# Disability-adjusted life years, years lived with disability, and years of life lost of diseases among children and adolescents in national and subnational levels of Iran, 1990–2021: A systematic analysis for the Global Burden of Disease 2021

**DOI:** 10.1371/journal.pone.0325085

**Published:** 2025-06-23

**Authors:** Bahareh Gholami, Mohammad-Mahdi Bastan, Samira Gholami, Azar Nejati, Sepehr Khosravi, Mohammad-Reza Malekpour, Nazila Rezaei, Sarvenaz Shahin, Ali Golestani

**Affiliations:** 1 Tehran University of Medical Sciences, Tehran, Iran; 2 Non-Communicable Diseases Research Center, Endocrinology and Metabolism Population Sciences Institute, Tehran University of Medical Sciences, Tehran, Iran; 3 Maternal and Childhood Obesity Research Center, Urmia University of Medical Sciences, Urmia, Iran; 4 Student Research Committee, Shahid Beheshti University of Medical Sciences, Tehran, Iran; 5 Endocrinology and Metabolism Research Center, Endocrinology and Metabolism Clinical Sciences Institute, Tehran University of Medical Sciences, Tehran, Iran; Addis Ababa University, ETHIOPIA

## Abstract

**Introduction:**

The global burden of disease (GBD) is a helpful measure that provides estimations regarding the effect of diseases and injuries on public health. This evidence is vital for making health policies and assessing the progress toward global health targets. Specifically, the burden of disease in children and adolescents can have a massive impact on a society’s health status. In this study, we aim to investigate the trends in years of life lost (YLL), years lost to disability (YLD), and disability-adjusted life years (DALYs) in children and adolescents under the age of 20 in Iran, based on GBD 2021 data.

**Methods:**

The Global Burden of Disease (GBD) classifies all causes of death and disability into four levels. Level 1 includes three main categories: communicable, maternal, neonatal, and nutritional diseases (CMNNDs), non-communicable diseases (NCDs), and injuries. Level 2 expands these categories into 22 clusters of causes. This study provides a descriptive analysis of the trends in YLLs, YLDs, and DALYs at levels 1 and 2 of the GBD hierarchy, focusing on children and adolescents under the age of 20 in Iran and its 31 provinces from 1990 to 2021.

**Results:**

All metrics show an overall decline. DALYs rate from all causes decreased by 79.8%, YLDs by 6.2%, and YLLs rate by 89.3%. This reduction was consistent across all metrics of CMNNDs, NCDs, and injuries, with the exception of YLD for NCDs, which showed a rise between 1990 and 2021; from 3733.4 (2742.7 to 5013.2) to 4036.1 (2941.5 to 5436.5) per 100,000 population. Mental disorders have significantly contributed to the upward trend of NCDs. NCDs, with a rate of 5666.2 per 100,000 population, exhibited the highest DALY rate in Iran in 2021. The trend of DALYs, YLLs, and YLDs of injuries and CMNNDs in Iran has been mostly downward. Differences between provinces have declined over thirty years. Following the COVID-19 pandemic, there have been changes in the trend of several diseases, especially the rise of mental disorders, respiratory infections and tuberculosis.

**Conclusion:**

Despite the overall decline in DALYs, YLLs, and YLDs for most causes of death, there has been an increase in YLDs from NCDs, underscoring the importance of addressing this issue. Reviewing YLLs to YLDs ratios for various diseases and injuries, combined with economic analyses of the cost-effectiveness of various health interventions, yields comprehensive evidence for health policy making.

## Introduction

The global burden of disease (GBD) is a helpful framework that provides estimates regarding the effect of diseases and injuries on public health [1]. The GBD Study has reported global health estimates categorized for different age groups, sexes, and locations since the early 1990s [2]. With the emergence of Coronavirus Disease 2019 (COVID-19) in late 2019, caused by severe acute respiratory syndrome coronavirus 2 (SARS-CoV-2), the GBD 2020 report was not published, making GBD 2021 the first available post-pandemic assessment [3].

Childhood and adolescence, defined as 0–9 and 10–19 years old, respectively, are vulnerable periods in life but also crucial for establishing lifelong health behaviors, including nutritional habits, physical activity, and disease prevention practices [4]. Their well-being is essential for the overall development of countries [5]. Regarding children and adolescent health, different global goals have been set over the past decades. From 1990 to 2019, GBD studies demonstrated improvements in overall health for the global population, largely due to widespread immunization programs, enhanced maternal and neonatal care, improved management of childhood illnesses, and public health initiatives focusing on sanitation, hygiene, and nutrition, all of which contributed to reducing under-5 mortality rates and overall child mortality [6,7]. In GBD 2021, for the first time in three decades, we have noticed an increase in age-standardized DALYs in 2020 and 2021, a new trend [8] which suggests that, despite improvements in reducing child mortality over the past twenty years, COVID-19 threatened these advancements [9].

Following the pandemic, the disease burden shifted, with COVID-19 replacing neonatal disorders as the primary cause of disability-adjusted life years (DALYs) across all age groups in 2021 [8]. While COVID-19 did not directly affect children as much as it did adults [10], the implementation of pandemic management measures such as social distancing and school closures interfered with children’s daily lives and had indirect impacts on their lives [11]. This led to a decrease in social interactions, heightened anxiety, and fewer opportunities for physical activities, suggesting that COVID-19 had both immediate and long-term impacts on the mental and physical health of children and adolescents [12].

Since understanding the burden of diseases in children and adolescents is crucial for effective prevention, treatment, and policymaking, it is necessary to have an up-to-date analysis of disease burden by cause, age, sex, and location. In 2020, at the peak of the pandemic, COVID-19 was an emergency requiring immediate attention. However, today, COVID-19 is viewed as a manageable infectious disease that countries must adapt to. The changing trend in GBD in the post-COVID era calls for the need to reassess health care system performance thoroughly. We need 2021 GBD estimates to adjust strategies, establish new priorities, and identify emerging challenges, given the importance of addressing the gap. By integrating DALY data into policymaking, healthcare systems can take a holistic approach to resource allocation. The objective of this study is to assess the trends and patterns in the all-cause burden of disease, measured through DALYs, years of life lost (YLLs), and years lived with disability (YLDs), among children and adolescents in Iran from 1990 to 2021. By analyzing changes across major disease categories—communicable, maternal, neonatal, and nutritional diseases (CMNNDs), injuries, and non-communicable diseases (NCDs)—and stratifying results by age group, gender, and province, the study aims to identify shifts in health priorities, evaluate the impact of public health interventions, and highlight regional disparities. This analysis seeks to provide evidence-based insights to inform targeted health policies that address the evolving needs of this population and reduce inequities across Iran.

## Method and material

### Overview

The Global Burden of Disease (GBD) 2021 study represents the most recent and thorough evaluation of the burden of 288 causes of death across 204 countries and territories from 1990 to 2021 [6]. The GBD study examines various parameters of global health and disease burden, including years of life lost (YLLs), years lived with disability (YLDs), healthy life expectancy (HALE), and disability-adjusted life years (DALYs). DALYs are computed as the sum of YLLs and YLDs. These metrics are categorized by age, sex, year, and location, providing a multidimensional perspective on global health trends. The details of the methodology have been previously discussed elsewhere [6,8]. The data employed in this study is accessible via the Global Health Data Exchange (https://ghdx.healthdata.org/gbd-2021) and the GBD Compare platform (https://vizhub.healthdata.org/gbd‐compare/).

### Estimation framework

The GBD 2021 study employs a complex, multi-faceted approach to data collection, processing, and estimation. The extensive GBD 2021 database incorporated a wide range of data types from vital registration systems, verbal autopsy data, surveys and censuses, surveillance systems, cancer registries, police records, open-source databases, minimally invasive tissue sampling, existing scientific reports on cohorts, registries, and population surveys, health system administrative data, and inpatient and outpatient claims data.. For Iran, these data sources are consistent and accessible via the Global Health Data Exchange (GHDx) at: https://ghdx.healthdata.org/geography/iran-islamic-republic. These data sources were standardized and adjusted to ensure comparability across various parameters. The GBD study utilizes several standardized tools to generate estimates by age, sex, location, and year. The Cause of Death Ensemble model (CODEm) was utilized to estimate cause-specific death rates for causes of death in our study. CODEm employs an ensemble of statistical models, systematically testing various combinations of covariates based on their out-of-sample predictive validity. This approach allows for the integration of diverse data sources, enhancing the robustness of mortality estimates. Specifically, CODEm was executed separately by sex and for countries and territories with and without extensive vital registration data, thereby reducing the risk of uncertainty inflation from heterogeneous data sources. To generate the ensemble model for each cause, multiple iterations of out-of-sample predictive validity were assessed, with models exhibiting the smallest root-mean-square error being weighted accordingly. For causes with unique epidemiological characteristics, significant changes in reporting practices, or limited data availability, customized modeling strategies were employed. These strategies included utilizing prevalence, incidence, case-fatality data, or data related to sub-causes to inform the cause of death estimates. Methodological improvements in this round of estimation focused on several key areas. Notably, cause-of-death data were updated to include age-specific data for younger age groups. Additionally, enhanced methods were implemented to account for stochastic variation in cause-of-death data, improving the estimation of small cause fractions associated with less common causes of death. By incorporating CODEm, we aim to provide more accurate and reliable estimates of mortality burden, which are essential for understanding health trends and informing public health interventions [6,8]. Spatiotemporal Gaussian process regression (ST-GPR) is used to synthesize data from multiple sources and fill in gaps where data are sparse [8].

These tools employed the GBD hierarchy to categorize diseases and injuries into a comprehensive four-level structure, encompassing both fatal and non-fatal causes. The Global Burden of Disease study organizes causes of death into a four-level hierarchy. Level 1 includes major categories such as communicable maternal, neonatal, and nutritional (CMNN) diseases; non-communicable diseases (NCDs); and injuries. Level 2 further divides these categories into 22 clusters. These clusters are subdivided into Level 3 and Level 4 causes, resulting in 288 estimated causes of death. To ensure comparability across causes, ages, sexes, locations, and time periods, the GBD study implements a set of data processing corrections. These include mapping different cause lists to the GBD cause hierarchy, age-sex splitting of aggregated data, redistribution of garbage codes (ill-defined or intermediate causes of death), noise reduction algorithms, and bias adjustments.

### Case definition

YLDs are calculated by multiplying the prevalence of a condition by its disability weight, which represents the severity of the condition and is derived from public surveys. YLLs are estimated by multiplying the number of deaths by the global standard life expectancy at the age of death. DALYs are determined by summing YLLs and YLDs for each health condition within a population.

This investigation is a descriptive analysis focusing on YLLs, YLDs, and DALYs at levels 1 and 2 in the GBD hierarchy among children and adolescents under 20 years old in Iran and its 31 provinces. The causes of death are categorized into the following age subgroups to clarify results: 0–4, 5–9, 10–14, and 15–19 years. The analysis includes sex-specific data to evaluate temporal trends.

### Statistical analysis

The Joinpoint regression models are linear statistical tools used for analyzing trends in different periods, implemented through the Joinpoint Regression Program (version 4.9.1.0 from the National Cancer Institute in Washington, DC). This program calculates the average annual percentage change (AAPC) and annual percentage change (APC) with a 95% confidence interval (CI) for the years 1990–2021. An APC greater than zero signifies an upward trend, while an APC less than zero indicates a downward trend during that period. Similarly, an AAPC greater than zero reflects an overall upward trend, and an AAPC less than zero shows a downward trend across the entire timeframe. When AAPC equals APC, it suggests a consistent increasing or decreasing trend. Point estimates were presented with 95% uncertainty intervals (UIs), derived by extracting the 2.5th and 97.5th percentiles from 500 samples drawn from the posterior distribution [8]. This approach enabled the generation of reliable estimates of uncertainty distribution essential for robust statistical evaluation. Statistical analyses were performed using R software (version 4.1.2, R Foundation for Statistical Computing, Vienna, Austria).

### Ethical considerations

This study was carried out in alignment with the principles set forth in the Declaration of Helsinki and was approved by the institutional review board of the Endocrinology and Metabolism Research Institute at Tehran University of Medical Sciences (IR.TUMS.EMRI.REC.1401.166). The results are based on estimates from the GBD 2021 study and adhere to all relevant guidelines and regulations. The authors also confirm that the data used in this research is publicly available.

## Results

### Overview

Table 1 provides the number and rate for DALYs, YLLs, and YLDs due to all-causes, communicable, maternal, neonatal, and nutritional diseases (CMNND), injuries and non-communicable diseases (NCD) for children and adolescent of both genders in Iran from 1990 to 2021.

**Table 1:** Number and rate of disability-adjusted life years (DALYs), years lived with disability (YLDs), and years of life lost (YLLs) of child and adolescents causes of death by sex in 1990 and 2021 and overall percent change over 1990–2021 in Iran.

Cause	Measure	Age, Metric	Year						% Change (1990–2021)		
			1990			2021					
			Both	Female	Male	Both	Female	Male	Both	Female	Male
All causes	DALYs	Number	15142212 (13874406 to 16518093)	6989459 (6436351 to 7621102)	8152753 (7401897 to 8975554)	2509670 (2117718 to 2956230)	1171786 (968317 to 1406379)	1337884 (1153072 to 1552447)	−83 (−86 to −81)	−83 (−86 to −80)	−84 (−86 to −81)
		Rate	48056 (44032 to 52423)	45243 (41662 to 49331)	50762 (46087 to 55885)	9707 (8191 to 11434)	9313 (7696 to 11177)	10081 (8688 to 11697)	−80 (−83 to −77)	−79 (−83 to −76)	−80 (−83 to −77)
	YLDs	Number	1727216 (1269070 to 2297237)	879317 (640867 to 1171500)	847898 (626753 to 1134549)	1328928 (966746 to 1781441)	703854 (514158 to 945182)	625074 (454809 to 840394)	−23 (−27 to −19)	−20 (−25 to −15)	−26 (−31 to −21)
		Rate	5482 (4028 to 7291)	5692 (4148 to 7583)	5279 (3902 to 7064)	5140 (3739 to 6890)	5594 (4086 to 7512)	4710 (3427 to 6332)	−6 (−11 to −1)	−2 (−7 to 4)	−11 (−17 to −4)
	YLLs	Number	13414996 (12275036 to 14567731)	6110142 (5588163 to 6628018)	7304855 (6622225 to 7990517)	1180742 (1094241 to 1273627)	467932 (428555 to 511114)	712810 (659415 to 764850)	−91 (−92 to −90)	−92 (−93 to −91)	−90 (−91 to −89)
		Rate	42574 (38957 to 46233)	39551 (36172 to 42903)	45483 (41232 to 49752)	4567 (4232 to 4926)	3719 (3406 to 4062)	5371 (4969 to 5763)	−89 (−90 to −88)	−91 (−92 to −89)	−88 (−90 to −87)
Communicable, maternal, neonatal, and nutritional diseases	DALYs	Number	6107170 (5257846 to 7417134)	2754865 (2368263 to 3465388)	3352305 (2862705 to 4086854)	615823 (528236 to 727564)	298892 (248963 to 361625)	316931 (272403 to 374319)	−90 (−92 to −88)	−89 (−92 to −87)	−91 (−93 to −88)
		Rate	19382 (16687 to 23539)	17832 (15330 to 22431)	20873 (17824 to 25446)	2382 (2043 to 2814)	2375 (1979 to 2874)	2388 (2053 to 2820)	−88 (−90 to −85)	−87 (−90 to −84)	−89 (−91 to −86)
	YLDs	Number	426356 (298518 to 591651)	211254 (143166 to 296744)	215102 (146585 to 304028)	246527 (171404 to 346075)	133759 (92458 to 191639)	112768 (76466 to 156416)	−42 (−50 to −29)	−37 (−48 to −21)	−48 (−58 to −32)
		Rate	1353 (947 to 1878)	1367 (927 to 1921)	1339 (913 to 1893)	954 (663 to 1339)	1063 (735 to 1523)	850 (576 to 1179)	−30 (−40 to −13)	−22 (−36 to −4)	−37 (−49 to −18)
	YLLs	Number	5680815 (4850418 to 6969759)	2543611 (2154138 to 3227952)	3137204 (2643971 to 3867150)	369296 (316917 to 429566)	165133 (139065 to 193761)	204163 (172129 to 236196)	−93 (−95 to −92)	−94 (−95 to −92)	−93 (−95 to −92)
		Rate	18029 (15393 to 22120)	16465 (13944 to 20894)	19533 (16462 to 24078)	1428 (1226 to 1661)	1312 (1105 to 1540)	1538 (1297 to 1780)	−92 (−94 to −90)	−92 (−94 to −90)	−92 (−94 to −90)
Injuries	DALYs	Number	4459823 (4148021 to 4809513)	2088048 (1926791 to 2262894)	2371775 (2189500 to 2599363)	417964 (392175 to 448597)	128056 (118457 to 140167)	289908 (270787 to 312313)	−91 (−92 to −90)	−94 (−95 to −93)	−88 (−89 to −86)
		Rate	14154 (13164 to 15264)	13516 (12472 to 14648)	14768 (13633 to 16185)	1617 (1517 to 1735)	1018 (941 to 1114)	2184 (2040 to 2353)	−89 (−90 to −87)	−92 (−93 to −91)	−85 (−87 to −84)
	YLDs	Number	124472 (92963 to 162287)	56689 (42332 to 73758)	67783 (50169 to 88937)	38882 (28344 to 51901)	17264 (12677 to 22971)	21618 (15746 to 29328)	−69 (−71 to −67)	−70 (−72 to −67)	−68 (−71 to −66)
		Rate	395 (295 to 515)	367 (274 to 477)	422 (312 to 554)	150 (110 to 201)	137 (101 to 183)	163 (119 to 221)	−62 (−65 to −59)	−63 (−66 to −59)	−61 (−64 to −59)
	YLLs	Number	4335351 (4015539 to 4675095)	2031359 (1870793 to 2198873)	2303992 (2115348 to 2526425)	379082 (353536 to 407862)	110792 (103081 to 122178)	268290 (248103 to 290269)	−91 (−92 to −90)	−95 (−95 to −94)	−88 (−90 to −87)
		Rate	13759 (12744 to 14837)	13149 (12110 to 14233)	14346 (13171 to 15730)	1466 (1367 to 1578)	881 (819 to 971)	2022 (1869 to 2187)	−89 (−91 to −88)	−93 (−94 to −92)	−86 (−88 to −84)
Non-communicable diseases	DALYs	Number	4575219 (3540429 to 5365055)	2146546 (1510800 to 2549714)	2428672 (1775800 to 2894482)	1464969 (1177225 to 1819299)	741582 (586275 to 937705)	723387 (584324 to 887293)	−68 (−75 to −56)	−65 (−73 to −49)	−70 (−76 to −57)
		Rate	14520 (11236 to 17027)	13895 (9779 to 16504)	15122 (11057 to 18022)	5666 (4553 to 7037)	5894 (4659 to 7452)	5451 (4403 to 6686)	−61 (−69 to −46)	−58 (−67 to −38)	−64 (−71 to −48)
	YLDs	Number	1176388 (864207 to 1579642)	611374 (448244 to 821998)	565014 (415552 to 755807)	1043519 (760513 to 1405597)	552831 (400645 to 748121)	490688 (357209 to 660126)	−11 (−14 to −8)	−10 (−13 to −7)	−13 (−16 to −10)
		Rate	3733 (2743 to 5013)	3957 (2901 to 5321)	3518 (2587 to 4706)	4036 (2942 to 5437)	4394 (3184 to 5946)	3697 (2691 to 4974)	8 (5 to 12)	11 (7 to 15)	5 (2 to 9)
	YLLs	Number	3398831 (2371530 to 4005293)	1535172 (910156 to 1834565)	1863659 (1233091 to 2248890)	421450 (377521 to 479136)	188751 (166970 to 217057)	232699 (204155 to 272553)	−88 (−90 to −81)	−88 (−90 to −78)	−88 (−90 to −79)
		Rate	10787 (7526 to 12711)	9937 (5891 to 11875)	11604 (7678 to 14002)	1630 (1460 to 1853)	1500 (1327 to 1725)	1753 (1538 to 2054)	−85 (−88 to −77)	−85 (−88 to −73)	−85 (−88 to −75)

Rates are expressed per 100,000 population

### All causes of death and disability

In 2021, the total burden of all-cause DALYs among Iranian children and adolescents was estimated at 2,509,670 (95% UI: 2,117,718–2,956,230), a figure that underscores a dramatic reduction in disease impact since 1990. The DALY rate dropped from 48,056 (95% UI: 44,032–52,423) per 100,000 population in 1990–9,707 (95% UI: 8,191–11,434) in 2021—a striking 80% decline (95% UI: −83% to −77%). This sharp drop likely reflects significant advancements in healthcare infrastructure, preventive measures, or socioeconomic conditions, benefiting both female and male subgroups equally as their DALY rates followed a similar downward trajectory. The YLD rate, however, showed a more modest decline of 6% (95% UI: −11% to −1%), falling from 5,482 (95% UI: 4,028–7,291) in 1990–5,140 (95% UI: 3,739–6,890) in 2021 per 100,000 population, with reductions observed across both genders. In contrast, the YLL rate saw a near-vertical drop from 42,574 (95% UI: 38,957–46,233) in 1990–4,567 (95% UI: 4,232–4,926) in 2021—a testament to substantial reductions in premature mortality. This disparity between YLDs and YLLs suggests a shift in burden from fatal outcomes to chronic conditions requiring sustained management (Table 1).

### Communicable, maternal, neonatal, and nutritional diseases (CMNNDs)

The burden of CMNNDs in 2021 totaled 615,823 DALYs (95% UI: 528,236–727,564, a sharp decrease from earlier decades. The DALY rate for these diseases decreased from 19,382 (95% UI: 16,687–23,539) per 100,000 population in 1990–2,382 (95% UI: 2,043–2,814) in 2021, reflecting an 88% reduction. This decline, consistent across both female and male subgroups, likely signals robust improvements in vaccination programs, maternal care, and nutritional support—interventions historically effective against CMNNDs. The YLD rate also decreased by 30% (95% UI: −40% to −13%), from 1,353 (95% UI: 947–1,878) in 1990–954 (95% UI: 663–1,339) in 2021 per 100,000 population, with parallel reductions in both genders. Even more striking, the YLL rate dropped by 92% (95% UI: −94% to −90%), from 18,029 (95% UI: 15,393–22,120) in 1990–1,428 (95% UI: 1,226–1,661) in 2021, underscoring a near-elimination of fatal outcomes from these conditions. These trends highlight a public health success, though the lingering YLD burden suggests some persistent morbidity challenges (Table 1).

### Injuries

Injuries contributed 417,964 DALYs (95% UI: 392,175–448,597) in 2021, a notable burden that has nonetheless diminished significantly over time. The DALY rate for injuries dropped from 14,154 (95% UI: 13,164–15,264) per 100,000 population in 1990–1,617 (95% UI: 1,517–1,735) in 2021—an 89% decline (95% UI: −90% to −87%) observed across both genders. This substantial reduction may reflect enhanced safety measures, such as road traffic regulations or injury prevention campaigns, which have decreased both incidence and severity. The YLD rate followed suit, decreasing by 62% (95% UI: −65% to −59%) from 395 (95% UI: 295–515) in 1990–150 (95% UI: 110–201) in 2021 per 100,000 population, with declines in both female and male subgroups. Similarly, the YLL rate fell by 89% (95% UI: −91% to −88%), from 13,759 (95% UI: 12,744–14,837) in 1990–1,466 (95% UI: 1,367–1,578) in 2021, reinforcing the success of mortality-focused interventions. The sharper drop in YLLs compared to YLDs suggests that while fatal injuries have been largely mitigated, non-fatal injuries continue to pose a residual challenge (Table 1).

### Non-communicable diseases (NCDs)

NCDs emerged as the dominant contributor to disease burden in 2021, with an estimated 1,464,969 DALYs (95% UI: 1,177,225–1,819,299). The DALY rate for NCDs decreased markedly from 14,520 (95% UI: 11,236–17,027) per 100,000 population in 1990–5,666 (95% UI: 4,553–7,036) in 2021—a 61% decline (95% UI: −69% to −46%) seen in both genders. This reduction likely comes from improved management of life-threatening NCDs, such as cancers, through early diagnosis and treatment. However, a contrasting trend emerged in the YLD rate, which rose by 8% (95% UI: 5% to 12%) from 3,733 (95% UI: 2,743–5,013) in 1990–4,036 (95% UI: 2,942–5,437) in 2021, with increases in both female and male subgroups. This uptick points to a growing prevalence of chronic, non-fatal conditions—such as mental disorders—posing new public health challenges. Meanwhile, the YLL rate for NCDs dropped dramatically by 85% (95% UI: −88% to −77%), from 10,787 (95% UI: 7,526–12,711) in 1990–1,630 (95% UI: 1,460–1,853) in 2021, reflecting a substantial reduction in premature deaths. The divergence between rising YLDs and falling YLLs underscores a shift toward managing long-term disability rather than mortality (Table 1).

### Estimates based on age-groups

**Fig 1:**
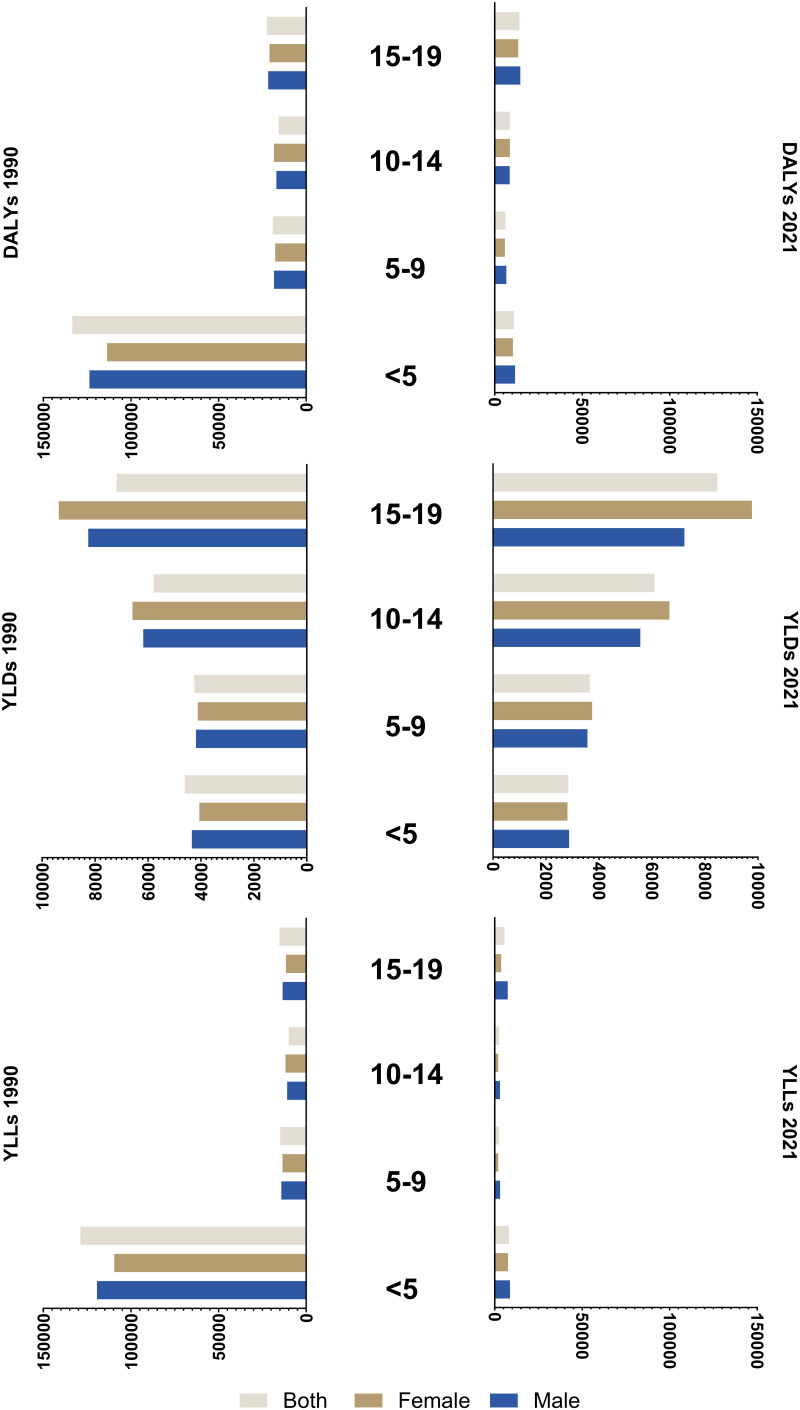
Rate of disability-adjusted life years (DALYs), years lived with disability (YLDs), and years of life lost (YLLs) of child and adolescents causes of death in Iran in 1990 and 2021, by sex and age.

Fig 1 illustrates the rates of DALYs, YLDs, and YLLs from all causes across age groups (<5, 5–9, 10–14, 15–19) and genders in 1990 and 2021. DALY rates declined universally across all age groups and both genders, with the most pronounced reduction in the < 5 age group—from 123,740.4 (95% UI: 111,940–135,701.1) in 1990 to a much lower burden by 2021. In contrast, the 15–19 age group exhibited the highest DALY rate in 2021 at 14,072.2 (95% UI: 11,620.8 to 16,865.4) and the smallest decline in DALYs, marking it as the age group with the greatest burden. This suggests a notable concentration of health challenges among older adolescents.

YLD rates decreased across most age groups except the 15–19 cohort, where they remained elevated at 8,258.5 in 1990 and 8,463.3 in 2021—the highest among all groups in both years. This persistence likely reflects adolescent-specific issues, such as mental health or injury-related disabilities. YLL rates, meanwhile, fell across all age groups, with the < 5 group showing the sharpest decline from 119,397.3 in 1990–8,177.6 in 2021, highlighting major gains in early childhood survival. (S2 Table).

### Provincial estimates

**Fig 2:**
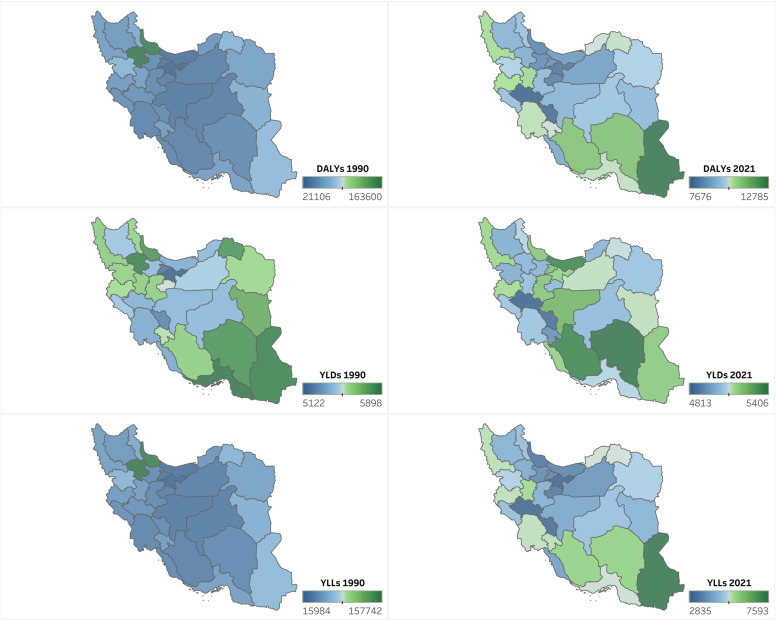
Geographical distribution of rate of disability-adjusted life years (DALYs), years lived with disability (YLDs), and years of life lost (YLLs) of child and adolescents causes of death among both sexes in 1990 and 2021 in Iran. (Contains information from OpenStreetMap and OpenStreetMap Foundation, which is made available under the Open Database License, https://www.openstreetmap.org/copyright).

**Fig 3:**
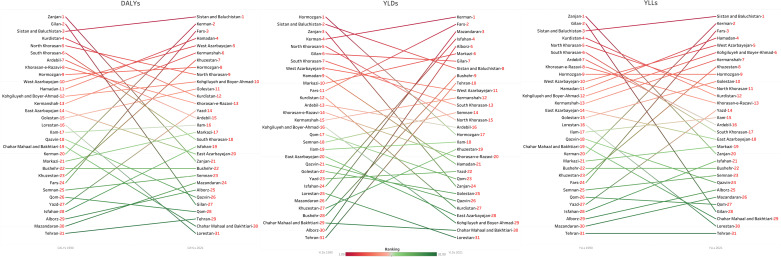
Ranking of rate of disability-adjusted life years (DALYs), years lived with disability (YLDs), and years of life lost (YLLs) of child and adolescents causes of death among both sexes in 1990 and 2021 in Iran.

From 1990 to 2021, Zanjan, Gilan, and Kurdistan led the nation in reducing DALY rates, with declines of 94.4% (95% UI: −95.3% to −93.3%), 94.7% (95% UI: −95.8% to −93.6%), and 85.2% (95% UI: −87.6% to −82.6%), respectively (S1 Table, Fig 2). Zanjan’s rank dropped from 1st to 21st, and Gilan and Kurdistan saw similar shifts (Fig 3), suggesting localized successes in health policy or infrastructure that could serve as models for other regions. For YLDs, Zanjan (−14.4%, 95% UI: −21.6% to −6.2%), Hormozgan (−13.7%, 95% UI: −21% to −5%), and North Khorasan (−12.1%, 95% UI: −20.3% to −3.8%) recorded the largest declines, with corresponding rank drops (Fig 3). In terms of YLLs, Zanjan (−97.3%, 95% UI: −97.7% to −97%) and Gilan (−97.9%, 95% UI: −98.2% to −97.6%) exhibited near-total reductions, reflecting exceptional progress in preventing premature mortality (S1 Table).

### DALYs, YLDs and YLLs by cause across all provinces

**Fig 4:**
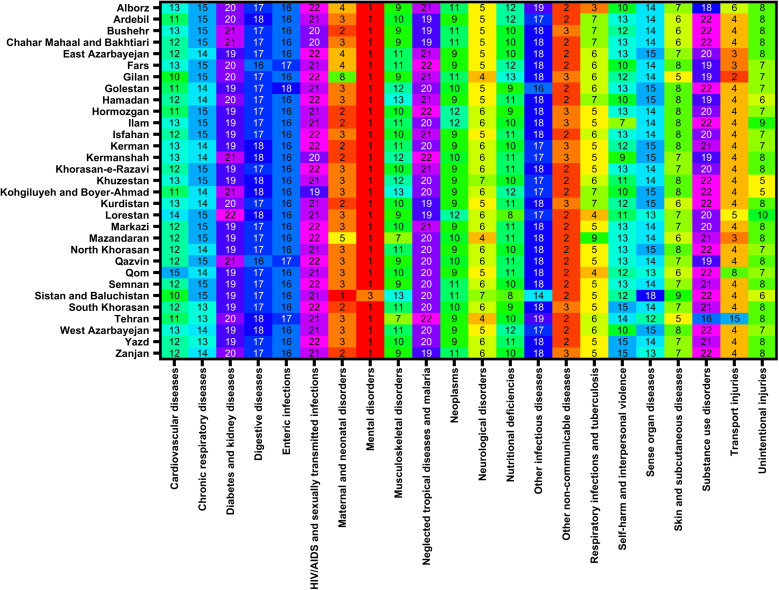
Ranking of rate of disability-adjusted life years (DALYs) of child and adolescents causes of death among both sexes in 2021 in Iran.

Fig 4 demonstrates the ranking of the top 22 causes of DALYs across all provinces of Iran in 2021. Mental disorders had the highest rank in almost all 32 provinces. Other non-communicable diseases and maternal and neonatal diseases have ranked second and third, respectively, in the majority of provinces (Fig 4). In both female and male subgroups, mental disorders held the highest rank, followed by other non-communicable diseases (S5,S6 Figs). Mental disorders were also the leading cause of YLDs in all provinces in 2021, and this trend was consistent in both male and female subgroups (S7–S9 Figs). Additionally, transport injuries and maternal and neonatal disorders ranked the highest among most provinces in terms of YLLs in 2021.

### Trend in burden of disease from 1990 to 2021

**Fig 5:**
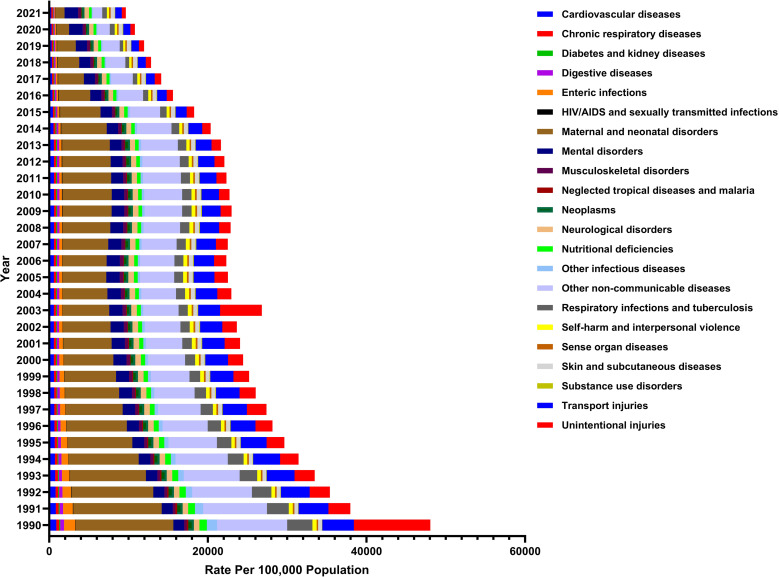
Time trend of rate of disability-adjusted life years (DALYs) of child and adolescents causes of death among both sexes in 1990-2021 in Iran.

Fig 5 illustrates the trend of the DALYs rate for children and adolescents in Iran, spanning all causes of death across both genders, from 1990 to 2021. Significant decline in DALYs (AAPC: −4.9%, CI [−5.7% to −4.2%]) was observed among both sexes from 1990 to 2021. Regarding the trend of DALYs in different periods, largest decline was reported in 2013−2021 (APC: −9.7% [95% CI: −10.6% to −8.8%]). In terms of YLDs, Significant decline (AAPC: −0.2%, CI [−0.4% to 0.0%]) was observed among both sexes from 1990 to 2021. However, a significant increase was observed in latest period (2018−2021) by 3.4% (95% CI: 2.7% to 4.2%). Regarding the trend of YLLs, Significant decline (AAPC: −6.4%, CI [−7.0% to −5.8%]) was observed among both sexes from 1990 to 2021 and largest decline was reported in 2014−2021 (APC: −15.9% [95% CI: −17.5% to −14.3%]) (S21 Fig).

The DALY rate for mental disorders followed a fluctuating pattern: rising from 1,352.7 (95% UI: 972.9 to 1,769.7) in 1990 to a peak of 1,692.6 (95% UI: 1,220.2 to 2,242.3) in 2004, dipping to 1,418.4 (95% UI: 1,020.6 to 1,861.1) by 2019, and climbing again to 1,711.4 (95% UI: 1,214.4 to 2,294.1) in 2021. This recent resurgence signals a growing burden of psychological health issues. Substance use disorders surged from 1990 to 2005 before steadily declining through 2021, while skin and subcutaneous diseases showed a consistent increase over the period. HIV/AIDS and sexually transmitted infections decreased from 1990 to 2000, spiked notably between 2000 and 2014, and then resumed a downward trend. Conversely, a broad range of conditions—including cardiovascular diseases, chronic respiratory diseases, digestive diseases, enteric infections, neoplasms, neurological disorders, nutritional deficiencies, respiratory infections and tuberculosis and, self-harm and interpersonal violence, transport and unintentional injuries—exhibited a consistent downward trend from 1990 to 2021. Notably, the DALY rate for nutritional deficiencies dropped steadily from 941.9 (95% UI: 708.6 to 1,259.1) to 338 (95% UI: 212.8 to 524.3) per 100,000, a decline that may reflect improvements in economic conditions and food security, offering a potential success story within the broader disease burden landscape (S4 Table).

## Discussion

This study depicts the trends in DALYs, YLLs, and YLDs for all causes of death, CMDNN, NCD, and injuries over the years from 1990 to 2021 among children and adolescents in Iran. The results show an overall decline in all the metrics. The DALYs rate from all causes decreased by 79.8%, the YLDs rate by 6.2%, and the YLLs rate by 89.3%. This reduction was consistent across all metrics from CMDNN, NCD, and injuries, with the exception of YLD for NCD, which showed a rise between 1990 and 2021.

Following the establishment of a national primary healthcare (PHC) network in 1985, several factors have contributed to the decline in these metrics in children and adolescent, including fundamental advances in public health and medical sciences, expansion of health facilities in remote areas, and increased coverage of health insurance [13]. There have been fundamental changes for children and adolescent care, including improved vaccination status, decreased mother and children’s mortality, and improved control for infectious diseases [14,15]. Analysis of these metrics at the provincial level has revealed variation in health improvement for children and adolescents, which may reflect differing levels of socioeconomic development in these regions. As an example, over thirty years, Zanjan, Gilan, and Kurdistan had the highest decline in the rate of DALYs. Also, Zanjan, Hormozgan, and North Khorasan experienced the highest decline in the rate of YLDs. Moreover, Zanjan and Gilan had a dramatic decline in terms of YLL rates. But differences in DALYs, YLDs, and YLLs rates between provinces have become much smaller over the last 30 years. This is because DALYs, YLDs, and YLLs rates have dropped more in provinces with higher rates than in provinces with lower rates.

In 2021, mental disorders overtook all other causes and ranked highest in terms of DALYs across almost all provinces with the exception of Sistan and Baluchistan province, where maternal and neonatal disorders ranked highest in terms of DALYs. The reason for this exception could be the high proportion of women giving birth at home and the high rate of consanguineous marriage among the population, both of which are predisposing factors to genetic disorders [16–19]. The alarming trend in mental disorders aligns with the global trend, which shows an increase in various mental disorders such as anxiety, major depressive disorders, and conduct disorders in childeren [20]. A cross-sectional study using data from the 2019 global burden of disease revealed that mental disorders caused one-fifth of all disease-related disability among individuals aged 5–24 years [21]. Additionally, globally, the COVID-19 pandemic had increased the DALYs for major depressive disorder, with an additional 137.1 DALYs (92·5–190·6) per 100,000 population, and this effect was more pronounced in younger age groups [22]. This is also consistent with our findings, which indicate a significant increase in mental disorders beginning in 2020. Our results show that the entire DALYs from mental disorders are comprised of YLDs, which indicate these disorders did not significantly contribute to mortality [23].

A 2017 global burden of disease study estimated that childhood cancers cause 11.5 million DALYs worldwide [24]. DALYs from neoplasms in Iran have shown a decline over thirty years. Likewise, DALYs from cancer have declined globally by 47.7% for children under five years [25]. The YLLs-to-YLDs ratio for neoplasms is high, indicating the lethal nature of childhood cancers; however, the YLLs have been primarily declining since 1994. Advances in available treatments and care for children with cancer are responsible for this decline [26].

The impact of the COVID-19 pandemic has increased susceptibility to NCDs. This has been due to changes in lifestyle, including low physical activity, an unhealthy sleep schedule, and an increased level of depressive symptoms and anxiety [27]. Additionally, the primary health care system experienced a significant decline in NCD prevention and control services during the COVID-19 pandemic. Although there has been a gradual compensation, more reinforcing policies are needed to make the compensation for enhancing NCD services in primary health care necessary [28].

The trend of DALYs, YLLs, and YLDs for injuries in Iran has been mostly downward. In terms of injury categories, transport injuries, unintentional injuries, self-harm, and interpersonal violence have all shown a decline over the past thirty years. Globally, transport injuries and unintentional injuries have also shown a decline from 1990 to 2019 [29]. In Iran, in 2003, there was a sudden peak in DALYs of unintentional injuries due to causalities related to the Kerman earthquake [30]. In 1990, the under-5 age group had the highest DALYs and YLLs from injuries. However, by 2021, this group exhibited the lowest DALYs and YLLs, while the 15–19 age group had the highest DALYs and YLLs.

From 1990 to 2021, transport injuries have consistently been among the leading causes of DALYs. Moreover, in 2021, transport injuries ranked among the top contributors of DALYs in most provinces of Iran. According to a WHO report, transport injuries are the leading cause of mortality among adolescents aged 15–19 worldwide [31]. In our analysis, we found that transport injuries ranked among the highest in terms of YLLs in most provinces, with males ranking higher than females. Furthermore, unintentional injuries have ranked higher among males than females, which is in line with a previous report on unintentional injuries in the south of Iran [32]. Transport, unintentional injuries, self-harm, and interpersonal violence all have a high YLLs to YLDs ratio, which indicates their improvement can have a significant impact on extending life expectancy. Improved safety measures for children of both genders have contributed to the decrease in DALYs from transport injuries since 1990.

DALYs, YLLs, and YLDs related to CMNND have significantly decreased over recent decades. In 1990, the under-5 age group had significantly higher rates of DALYs from CMNND compared to other age groups. However, the DALYs in this age group have experienced a greater decline than those in other age groups, which has led to a narrowing of the gap within this category. Despite this, the under-5 age group still has the highest DALYs.

The decline in DALYs and YLLs from maternal and neonatal disorders is consistent with the global trend, which showed a decline from 4198.5 per 100,000 population in 1990 to 2828.3 per 100,000 population in 2019. Despite this decline, maternal and neonatal disorders continue to contribute significantly to DALYs and YLLs in most provinces in Iran. Due to its high YLLs to YLDs ratio, further improvements in health care pertaining to maternal and neonatal disorders could have a significant impact on expanding life expectancy. Global estimates indicate that maternal hemorrhage causes about 24% of maternal disorders, and neonatal preterm birth causes 37% of neonatal disorders [33].

Up until 2014, the DALYs associated with HIV/AIDS and sexually transmitted infections showed a rising trend, but then began to decline. This decline was concurrent with the implementation of the Prevention of Mother-to-Child Transmission (PMTCT) program in Iran. This program, which incorporates early diagnosis and timely intervention for pregnant HIV-positive women, has demonstrated its effectiveness in reducing HIV transmission to infants from their HIV-positive mothers [34]. Additionally, the pilot study for the PMTCT program has shown the benefits of this program if integrated into primary health care (PHC) [35].

The trend for DALYs and YLLs from respiratory infections and tuberculosis has generally been decreasing; however, in 2019, there was an increase in DALYs and YLLs in this category. This rise can be attributed to the COVID-19 pandemic. The YLLs to YLDs ratio for this category was high, indicating its significant contribution to mortality.

Nutritional deficiencies have shown a decreasing trend over the past three decades in terms of DALYs, YLDs, and YLLs. This shows a significant shift over recent decades, which is primarily due to technological advancements, agricultural policy changes, and also economic, social, and lifestyle changes in Iran. Despite these improvements, there are still nutritional deficiencies in certain regions of Iran. Historically, health policies and educational programs in Iran have mostly focused on underweight and malnutrition in children. However, in the recent decade, there has been a pressing need to address obesity [36]. The ratio of YLDs to YLLs in nutritional deficiencies is high, which shows the importance of this category in enhancing healthy life expectancy compared to total life expectancy.

One key limitation of this study stems from its reliance on GBD estimates derived from medical records and registry data, which may be subject to incomplete registration, particularly in Iran. Insufficient coverage in rural provinces or under-reporting of age-specific conditions, such as neonatal disorders or adolescent mental health issues, could lead to underestimation of the true disease burden, potentially affecting the accuracy of our DALY, YLL, and YLD trends. While GBD estimation models have improved over time, these potential gaps highlight the need for enhanced primary data collection to validate and refine our findings. In this study, we focused on the trends in YLLs, YLDs, and DALYs among children and adolescents in Iran. While sociodemographic development has been a significant contributor to health gains over the past three decades, as highlighted by the GBD study, we did not include an analysis of the Socio-Demographic Index (SDI) or its impact on health outcomes in our current investigation. SDI as a composite measure of lag-distributed income per capita, average years of schooling, and the fertility rate in females younger than 25 years for a given location was not within the scope of this analysis. A detailed examination of the SDI ranking of individual provinces would require a separate and comprehensive investigation, which is beyond the objectives of our study. By maintaining a focused analysis on YLL, YLD, and DALYs, we aim to provide a clearer understanding of the health status and burden of disease among children and adolescents in Iran. Future studies that incorporate the SDI could provide valuable insights into how sociodemographic factors influence health outcomes, thereby enhancing our understanding of health disparities and informing targeted interventions.

## Conclusion

The establishment of the Primary Health Care (PHC) network marked a pivotal first step in reducing the disability-adjusted life years (DALYs) burden among children and adolescents in Iran. This foundation was reinforced by subsequent advancements in public health, including the expansion of health facilities into remote areas. These critical improvements transformed the landscape of disability across most provinces, shifting the predominant burden from maternal, neonatal, and communicable diseases to chronic conditions and non-communicable diseases (NCDs). This shift underlies the sharp decline in DALYs observed in vulnerable age groups, such as children under five and in provinces like Hormozgan and Zanjan, which previously bore the highest burdens. However, disparities persist, with provinces like Sistan and Balouchistan lagging behind, highlighting the need for further investment in primary care infrastructure to replicate successes seen elsewhere.

The emergence of COVID-19 from 2020 to 2021 contributed to a temporary rise in respiratory illnesses, though its impact was less pronounced in younger age groups like those under five. Instead, it amplified the growing challenge of NCDs in Iran, mirroring global trends. The increasing DALYs attributable to NCDs signal that current health policies and interventions are insufficient to address this evolving burden. Within the NCD category, mental disorders emerged as a particularly significant concern, raising an urgent alarm for targeted health strategies, especially for older children and adolescents who appear most affected. Policymakers should leverage insights from studies like this to develop tailored, evidence-based strategies that address the changing health needs of Iran’s youth, while simultaneously tackling persistent provincial inequalities to ensure equitable progress across the nation.

## Supporting information

S1 Fig Geographical distribution of rate of disability-adjusted life years (DALYs), years lived with disability (YLDs), and years of life lost (YLLs) of child and adolescents causes of death among females in 1990 and 2021 in Iran. (Contains information from OpenStreetMap and OpenStreetMap Foundation, which is made available under the Open Database License, https://www.openstreetmap.org/copyright).
(TIF)

S2 Fig Ranking of rate of disability-adjusted life years (DALYs), years lived with disability (YLDs), and years of life lost (YLLs) of child and adolescents causes of death among females in 1990 and 2021 in Iran. (TIF)

S3 Fig Geographical distribution of rate of disability-adjusted life years (DALYs), years lived with disability (YLDs), and years of life lost (YLLs) of child and adolescents causes of death among males in 1990 and 2021 in Iran. (Contains information from OpenStreetMap and OpenStreetMap Foundation, which is made available under the Open Database License, https://www.openstreetmap.org/copyright).
(TIF)

S4 Fig Ranking of rate of disability-adjusted life years (DALYs), years lived with disability (YLDs), and years of life lost (YLLs) of child and adolescents causes of death among males in 1990 and 2021 in Iran. (TIF)

S5 Fig Ranking of rate of disability-adjusted life years (DALYs) of child and adolescents causes of death among females in 2021 in Iran. (TIF)

S6 Fig Ranking of rate of disability-adjusted life years (DALYs) of child and adolescents causes of death among males in 2021 in Iran. (TIF)

S7 Fig Ranking of rate of years lived with disability (YLDs) of child and adolescents causes of death among both sexes in 2021 in Iran. (TIF)

S8 Fig Ranking of rate of years lived with disability (YLDs) of child and adolescents causes of death among females in 2021 in Iran. (TIF)

S9 Fig Ranking of rate of years lived with disability (YLDs) of child and adolescents causes of death among males in 2021 in Iran. (TIF)

S10 Fig Ranking of rate of years of life lost (YLLs) of child and adolescents causes of death among both sexes in 2021 in Iran. (TIF)

S11 Fig Ranking of rate of years of life lost (YLLs) of child and adolescents causes of death among females in 2021 in Iran. (TIF)

S12 Fig Ranking of rate of years of life lost (YLLs) of child and adolescents causes of death among males in 2021 in Iran. (TIF)

S13 Fig Time trend of rate of disability-adjusted life years (DALYs) of child and adolescents causes of death among females in 1990–2021 in Iran. (TIF)

S14 Fig Time trend of rate of disability-adjusted life years (DALYs) of child and adolescents causes of death among males in 1990–2021 in Iran. (TIF)

S15 Fig Time trend of rate of years lived with disability (YLDs) of child and adolescents causes of death among both sexes in 1990–2021 in Iran. (TIF)

S16 Fig Time trend of rate of years lived with disability (YLDs) of child and adolescents causes of death among females in 1990–2021 in Iran. (TIF)

S17 Fig Time trend of rate of years lived with disability (YLDs) of child and adolescents causes of death among males in 1990–2021 in Iran. (TIF)

S18 Fig Time trend of rate of years of life lost (YLLs) of child and adolescents causes of death among both sexes in 1990–2021 in Iran. (TIF)

S19 Fig Time trend of rate of years of life lost (YLLs) of child and adolescents causes of death among females in 1990–2021 in Iran. (TIF)

S20 Fig Time trend of rate of years of life lost (YLLs) of child and adolescents causes of death among males in 1990–2021 in Iran. (TIF)

S21 Fig Temporal trends of rate (per 100,000 population) of disability-adjusted life years (DALYs), years lived with disability (YLDs), and years of life lost (YLLs) of child and adolescents causes of death in Iran by sex from 1990 to 2021. (TIF)

S1 Table Subnational distribution of rate of disability-adjusted life years (DALYs), years lived with disability (YLDs), and years of life lost (YLLs) of child and adolescents causes of death by sex in 1990 and 2021 and overall percent change over 1990–2021 in Iran. (DOCX)

S2 Table Rate of disability-adjusted life years (DALYs), years lived with disability (YLDs), and years of life lost (YLLs) of child and adolescents causes of death in Iran in 1990 and 2021, by sex and age. (DOCX)

S3 Table Subnational rate of disability-adjusted life years (DALYs), years lived with disability (YLDs), and years of life lost (YLLs) of child and adolescents causes of death in 2021 in Iran by sex. (DOCX)

S4 Table Time trend of rate of disability-adjusted life years (DALYs), years lived with disability (YLDs), and years of life lost (YLLs) of child and adolescents causes of death in 1990–2021 in Iran by sex. (DOCX)

## Abbreviations

CODEm: cause of death ensemble model
COVID-19: coronavirus disease 2019
CMNNDs: communicable, maternal, neonatal, and nutritional diseases
DALY: disability-adjusted life years
GBD: Global Burden of Disease
HALE: healthy life expectancy
IMR: infant mortality rates
MDGs: Millennium Development Goals
NMR: neonatal mortality rates
NCDs: non-communicable diseases
PMTCT: prevention of mother-to-child transmission
PHC: primary health care
SARS-CoV-2: severe acute respiratory syndrome coronavirus 2
SDG: sustainable development goals
ST-GPR: spatiotemporal Gaussian process regression
U5MR: under-5-year mortality rates
UIs: uncertainty intervals
YLD: years lived with disability
YLL: years of life lost
