# Supplementary figures and images for: Disability-adjusted life years, years lived with disability, and years of life lost of diseases among children and adolescents in national and subnational levels of Iran, 1990–2021: A systematic analysis for the Global Burden of Disease 2021
Source: PLoS One. 2025 Jun 23;20(6):e0325085. doi: 10.1371/journal.pone.0325085 (PMC12184942; doi:10.1371/journal.pone.0325085)

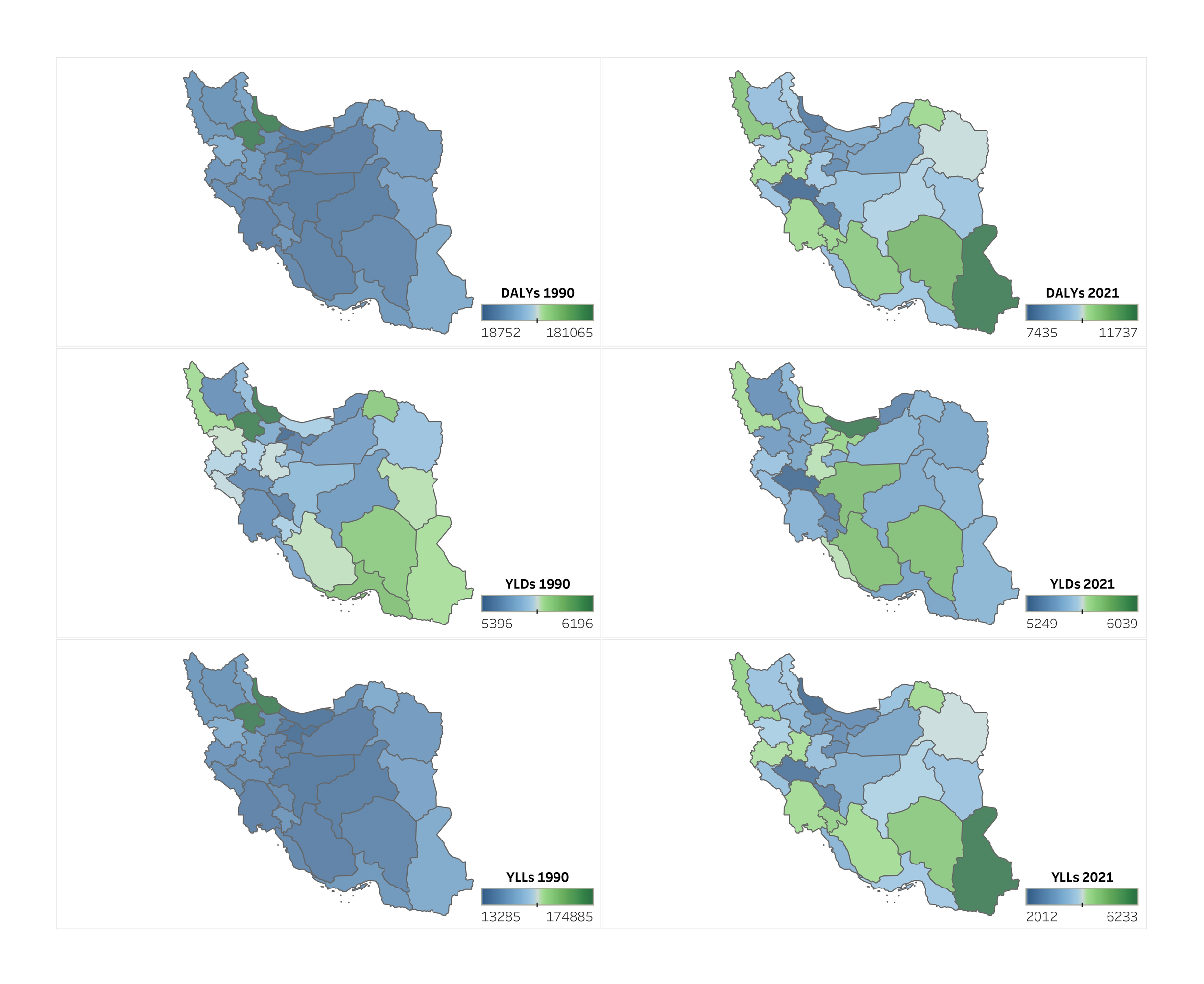

Supplement: S1 Fig — (Contains information from OpenStreetMap and OpenStreetMap Foundation, which is made available under the Open Database License, https://www.openstreetmap.org/copyright). (TIF) [file pone.0325085.s001.tif]

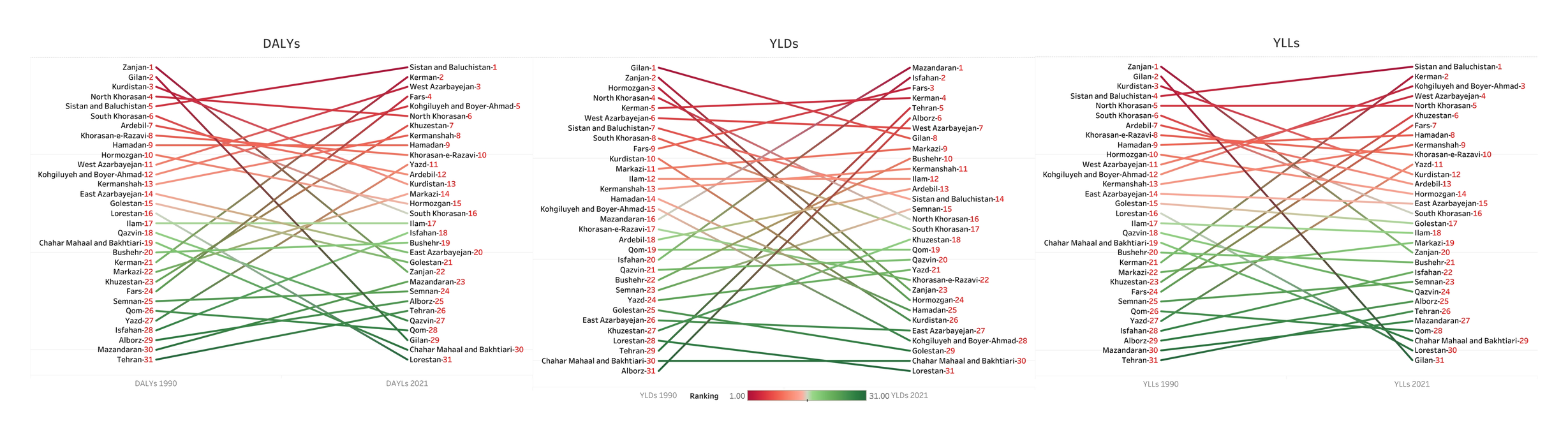

Supplement: S2 Fig — (TIF) [file pone.0325085.s002.tif]

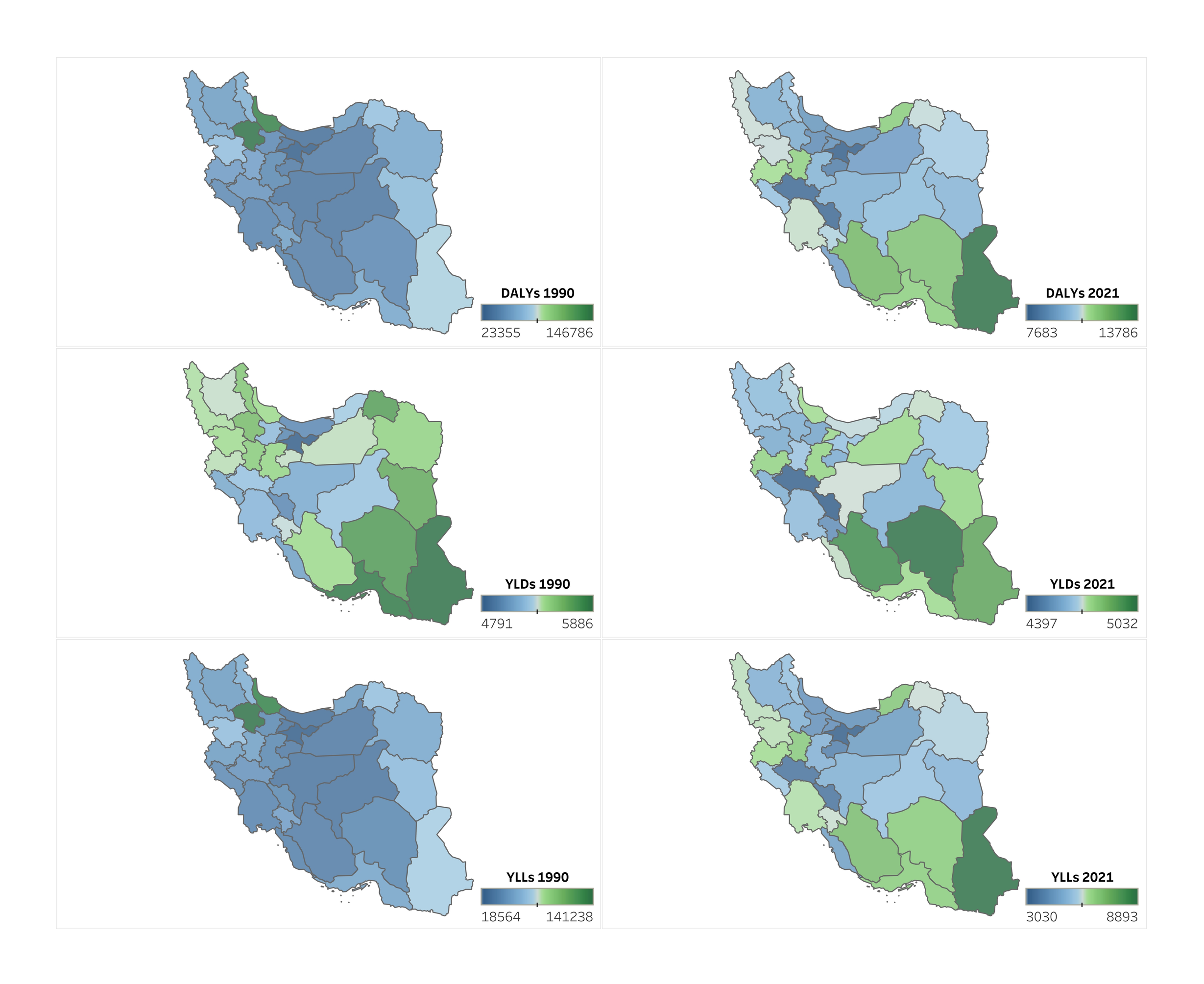

Supplement: S3 Fig — (Contains information from OpenStreetMap and OpenStreetMap Foundation, which is made available under the Open Database License, https://www.openstreetmap.org/copyright). (TIF) [file pone.0325085.s003.tif]

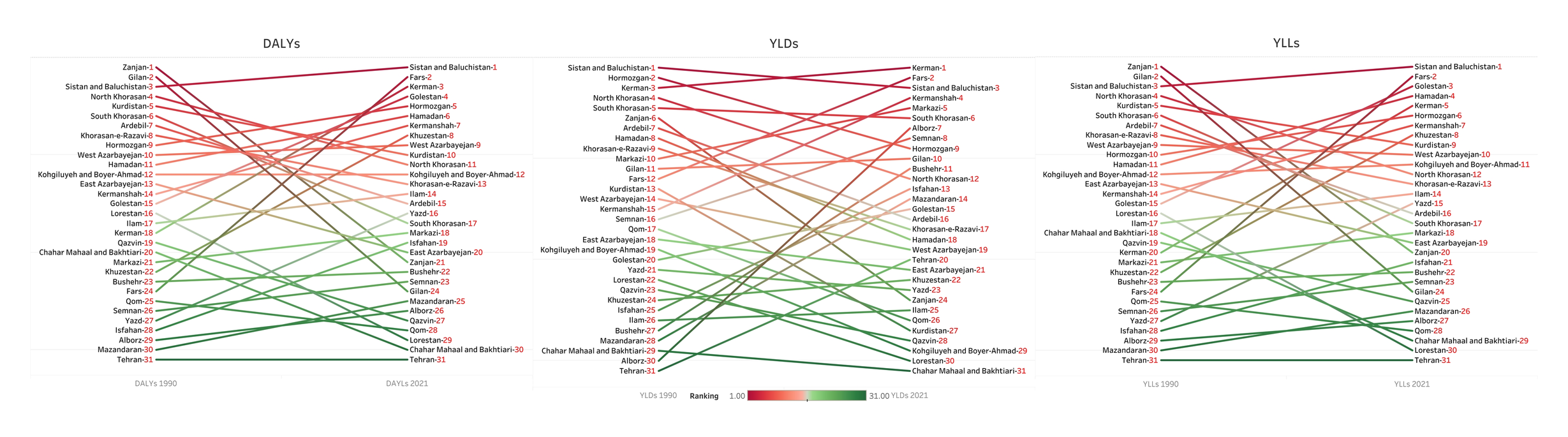

Supplement: S4 Fig — (TIF) [file pone.0325085.s004.tif]

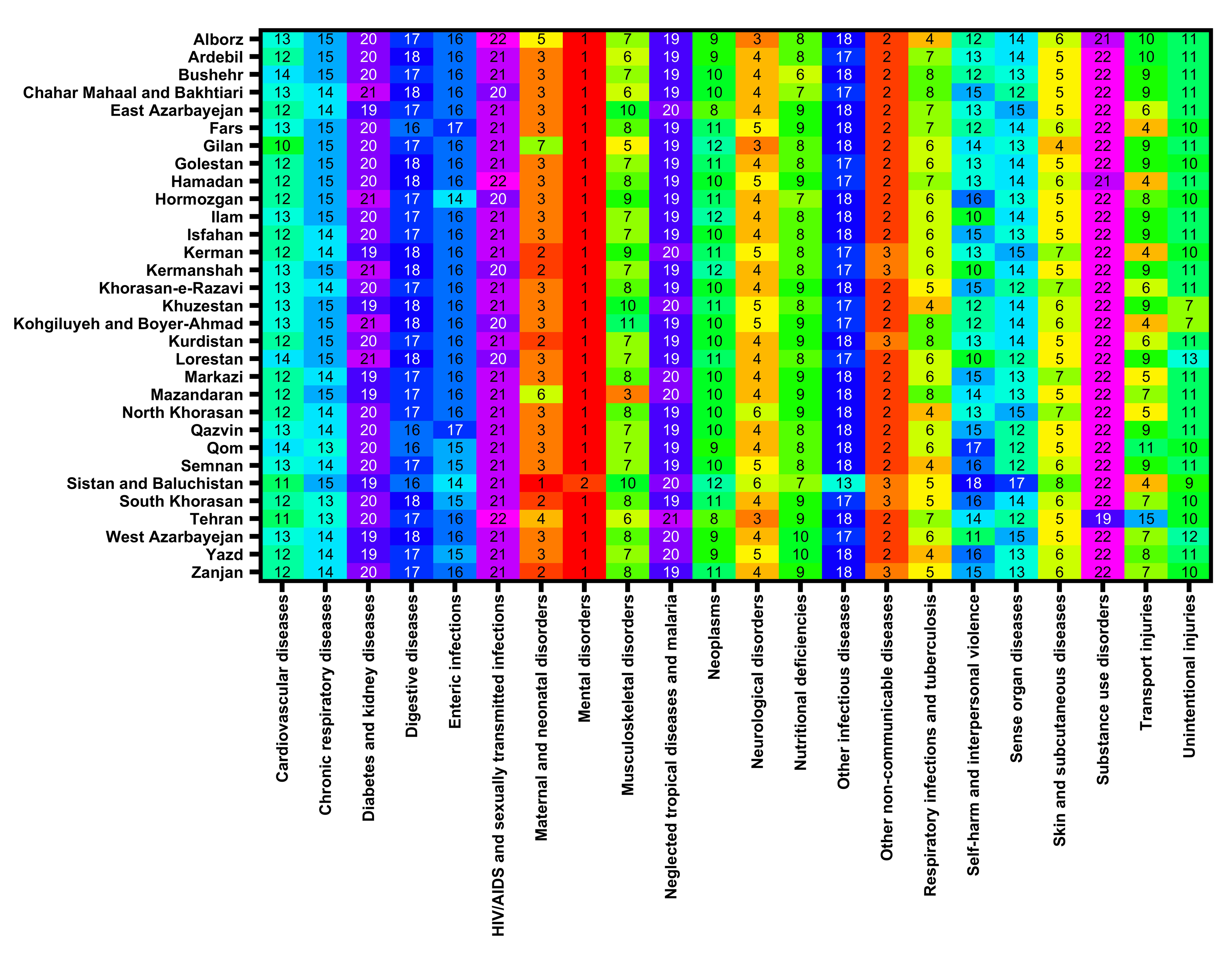

Supplement: S5 Fig — (TIF) [file pone.0325085.s005.tif]

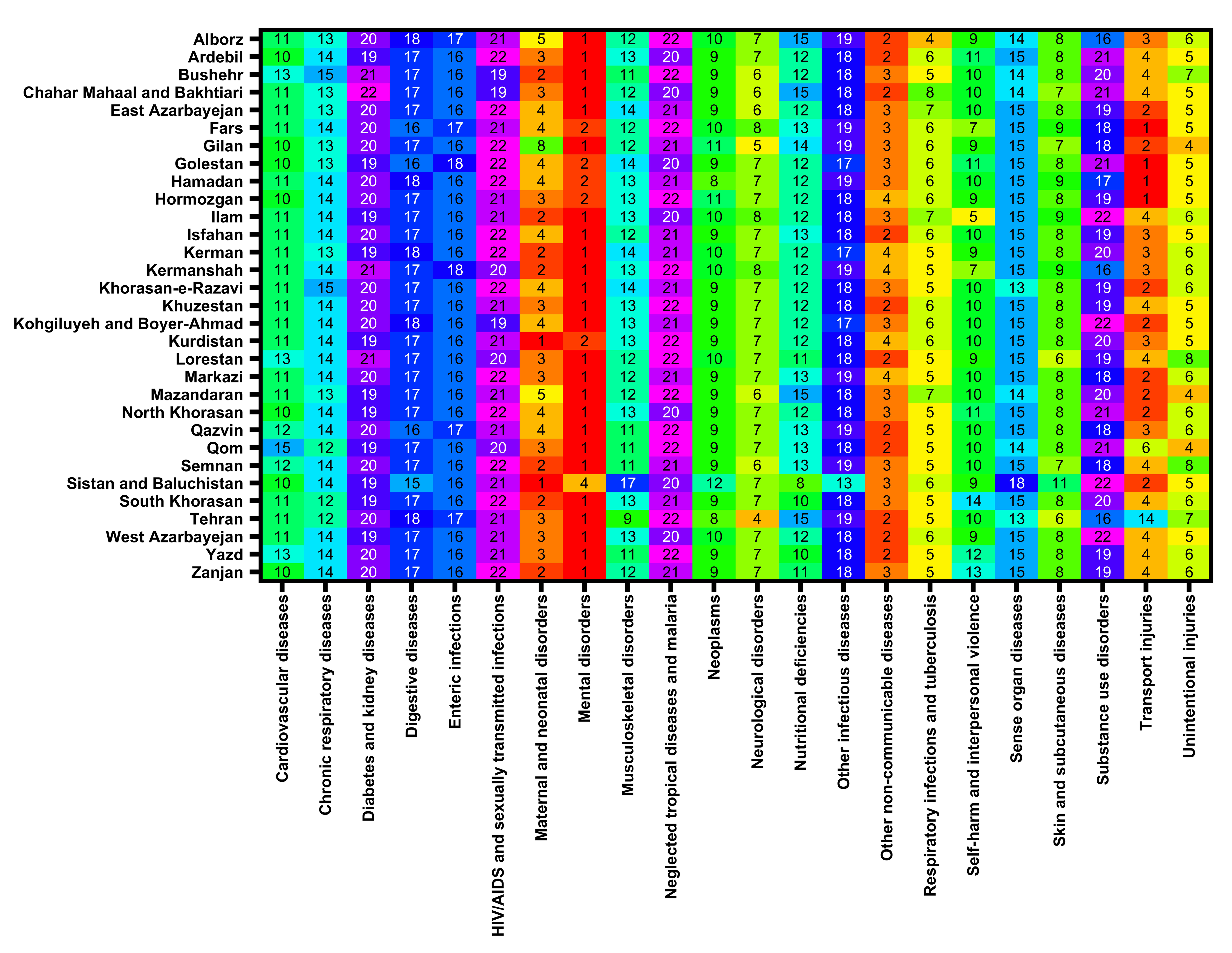

Supplement: S6 Fig — (TIF) [file pone.0325085.s006.tif]

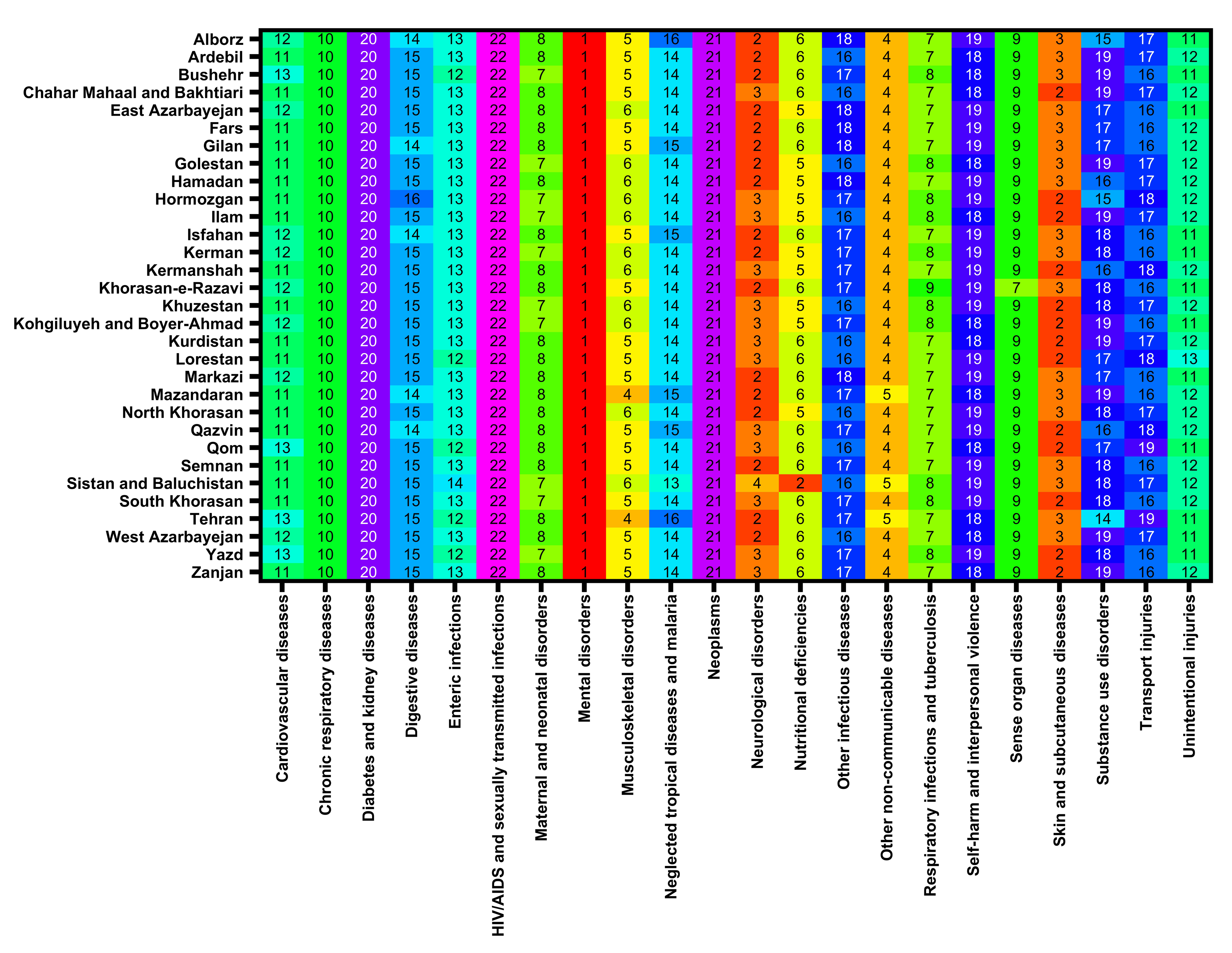

Supplement: S7 Fig — (TIF) [file pone.0325085.s007.tif]

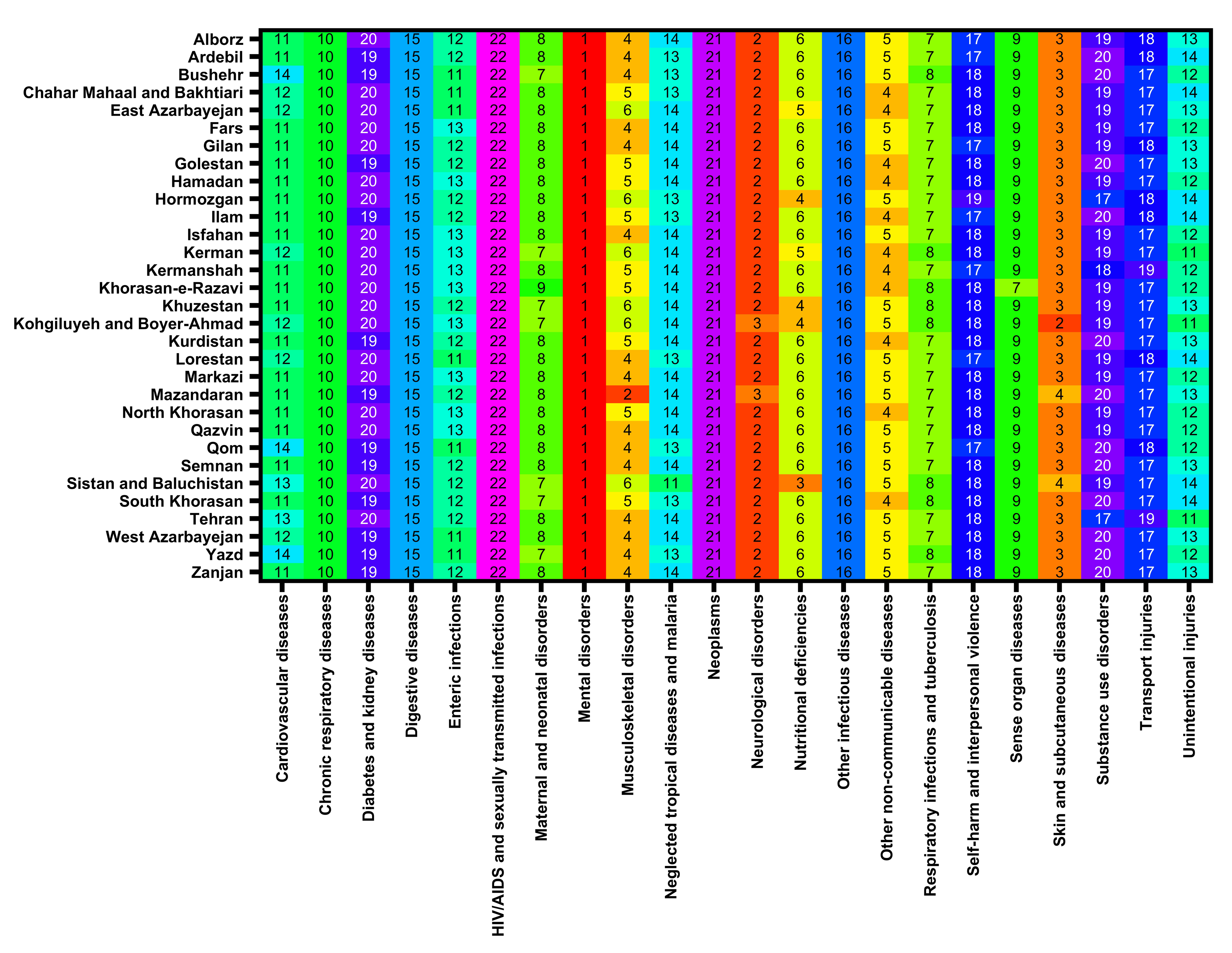

Supplement: S8 Fig — (TIF) [file pone.0325085.s008.tif]

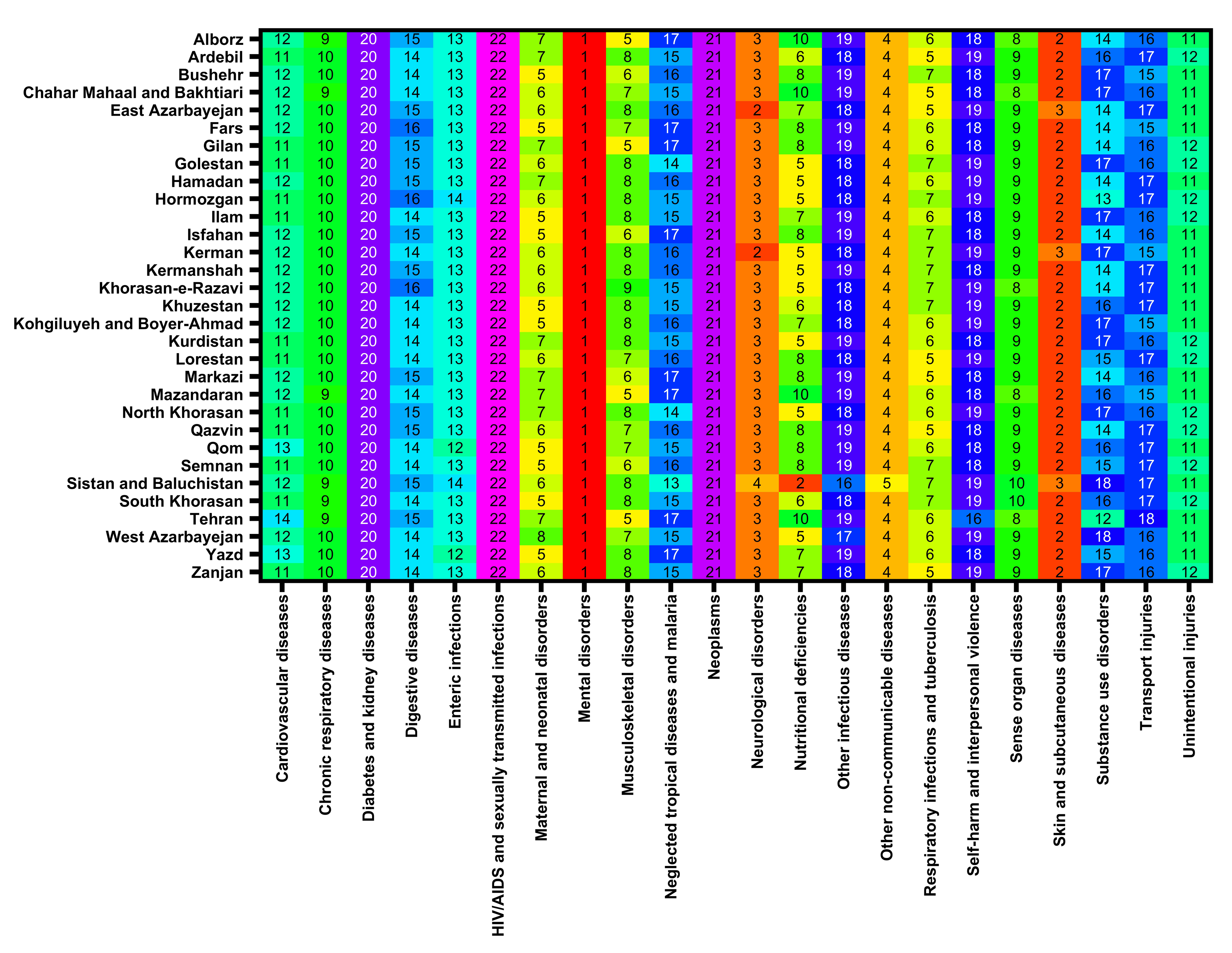

Supplement: S9 Fig — (TIF) [file pone.0325085.s009.tif]

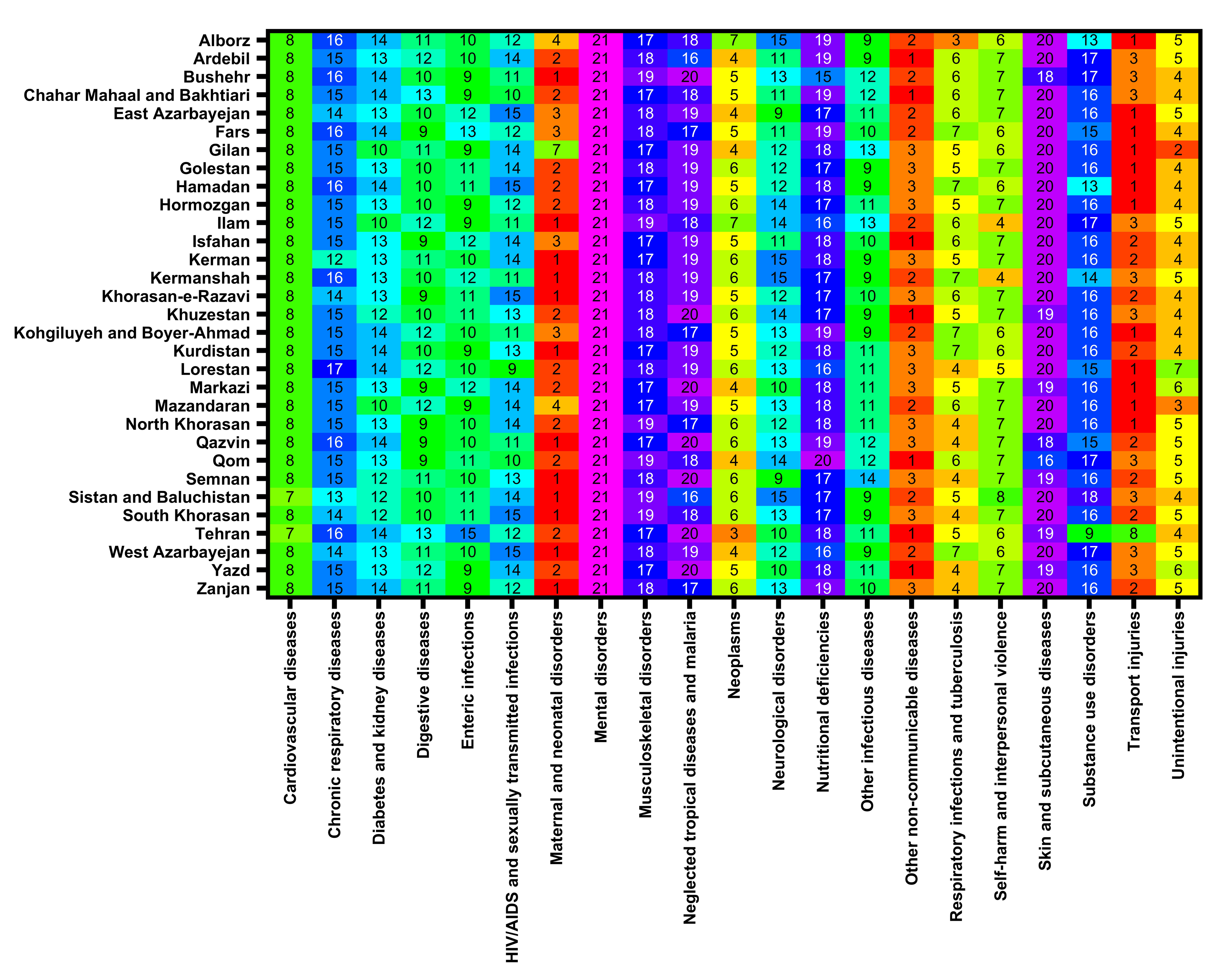

Supplement: S10 Fig — (TIF) [file pone.0325085.s010.tif]

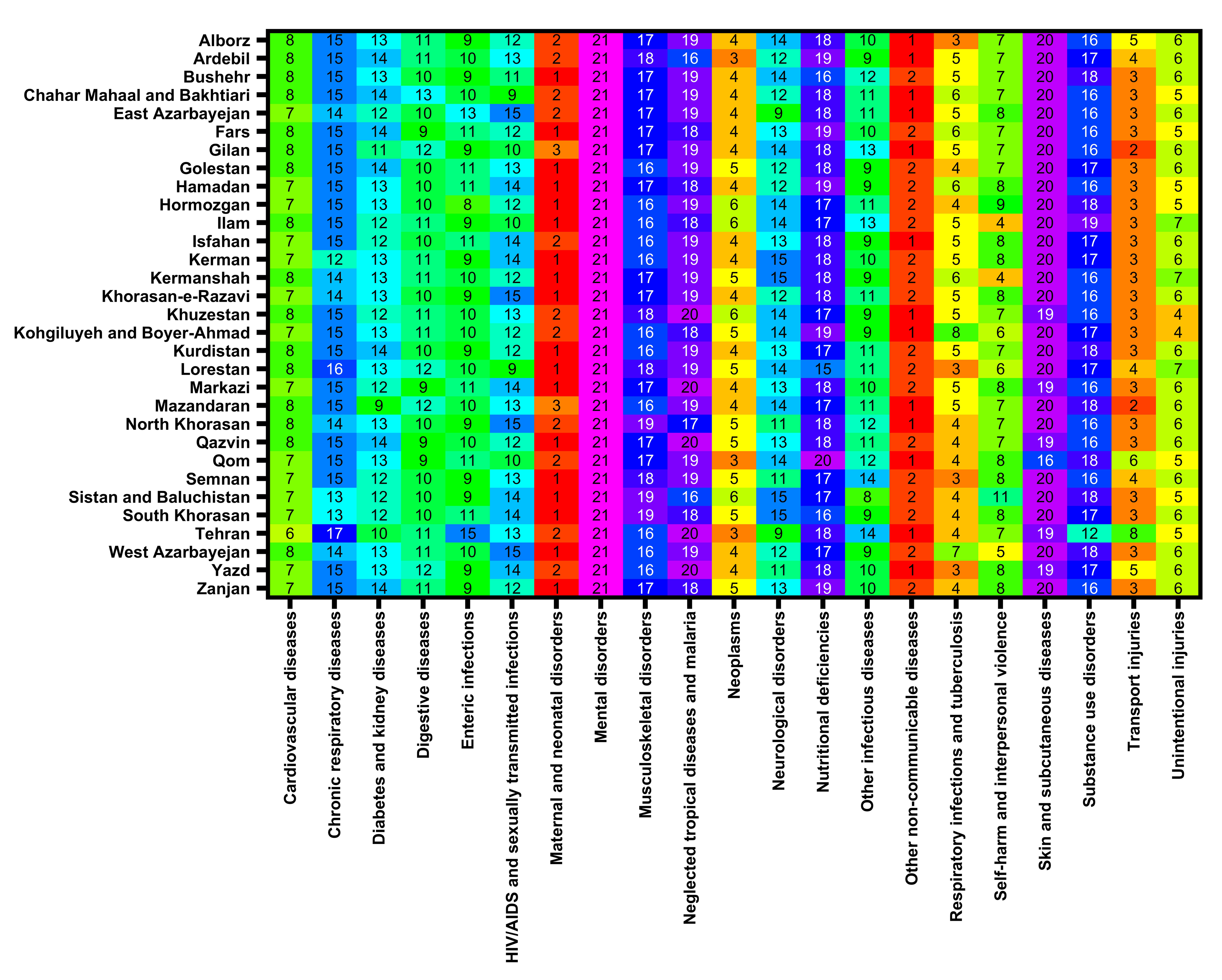

Supplement: S11 Fig — (TIF) [file pone.0325085.s011.tif]

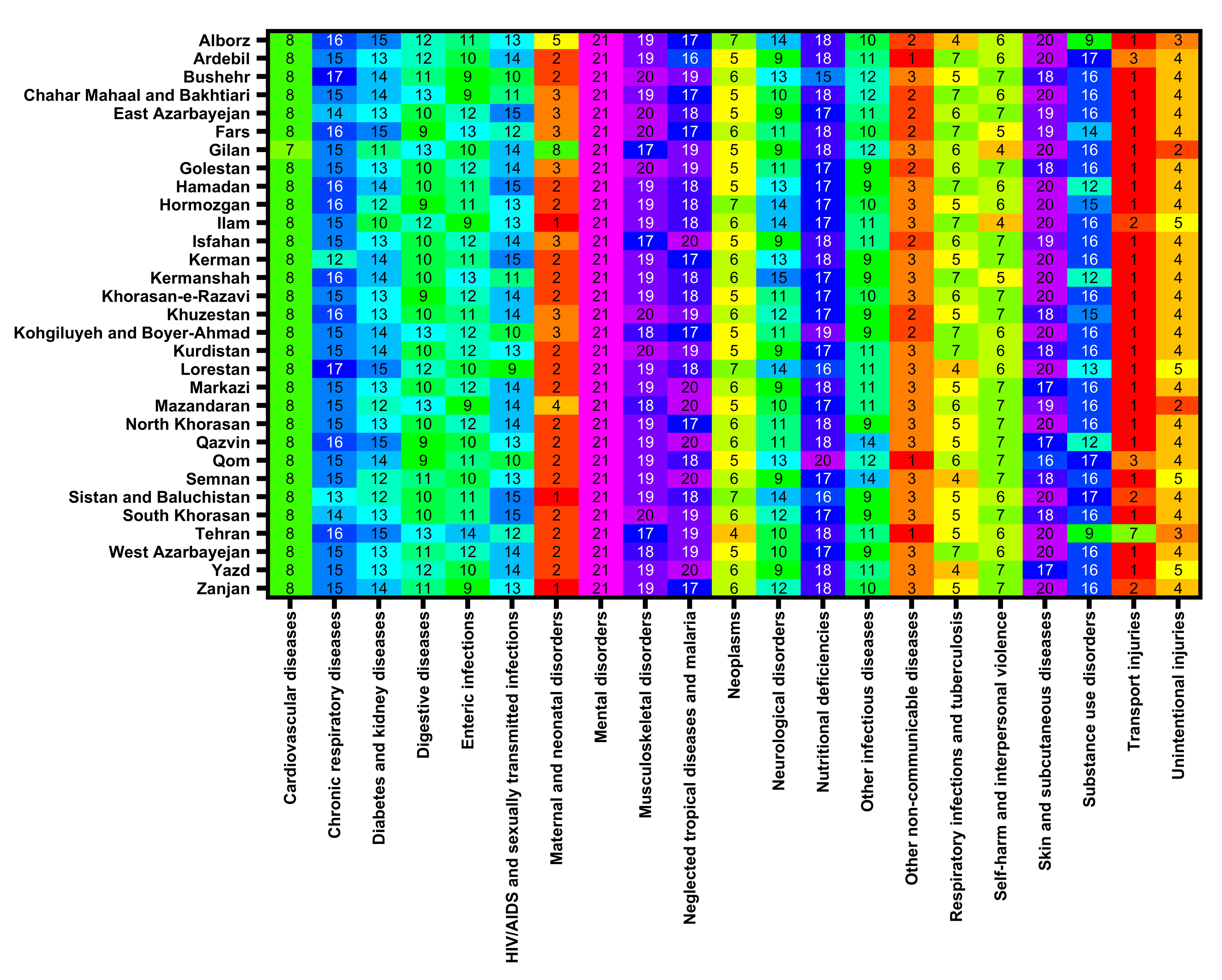

Supplement: S12 Fig — (TIF) [file pone.0325085.s012.tif]

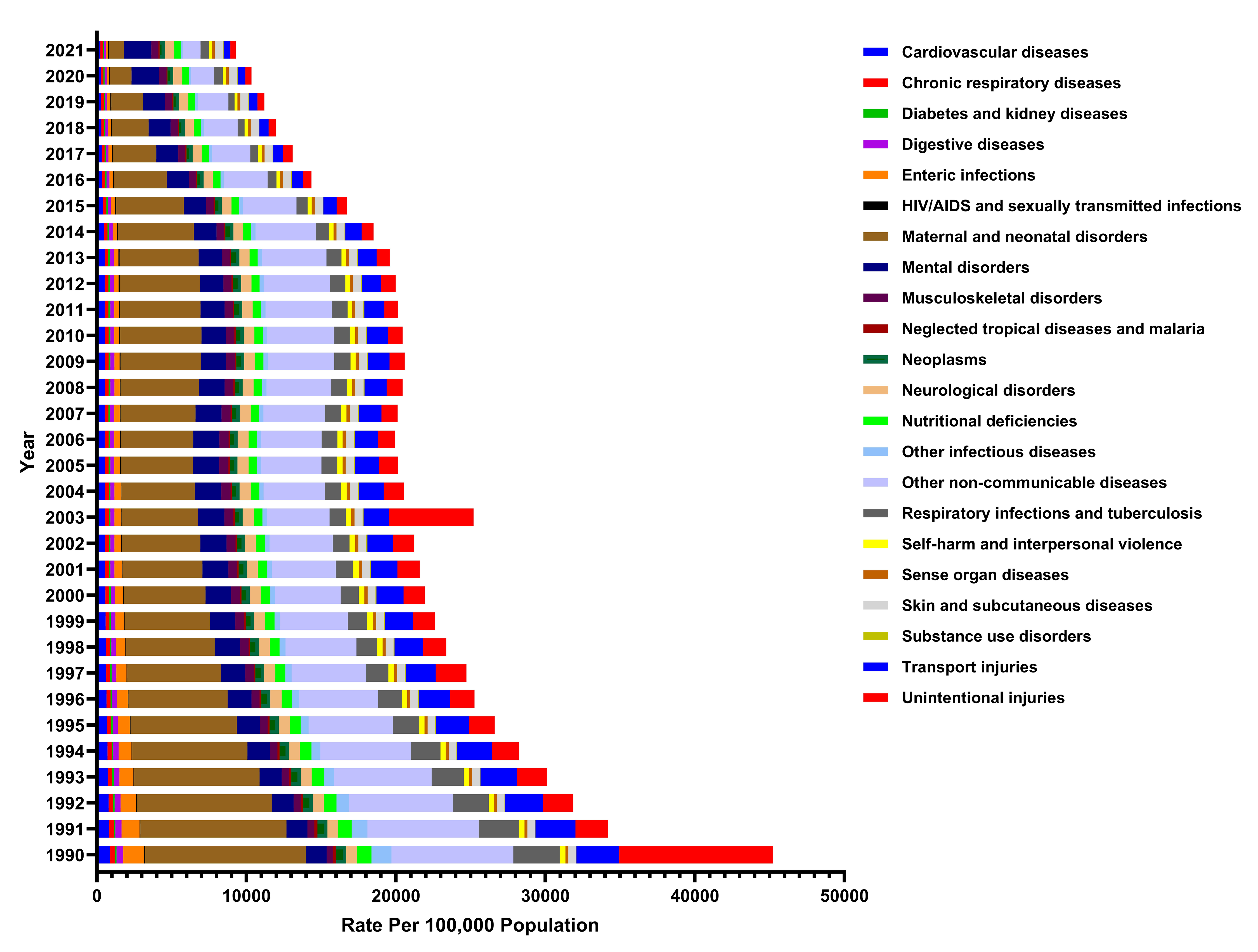

Supplement: S13 Fig — (TIF) [file pone.0325085.s013.tif]

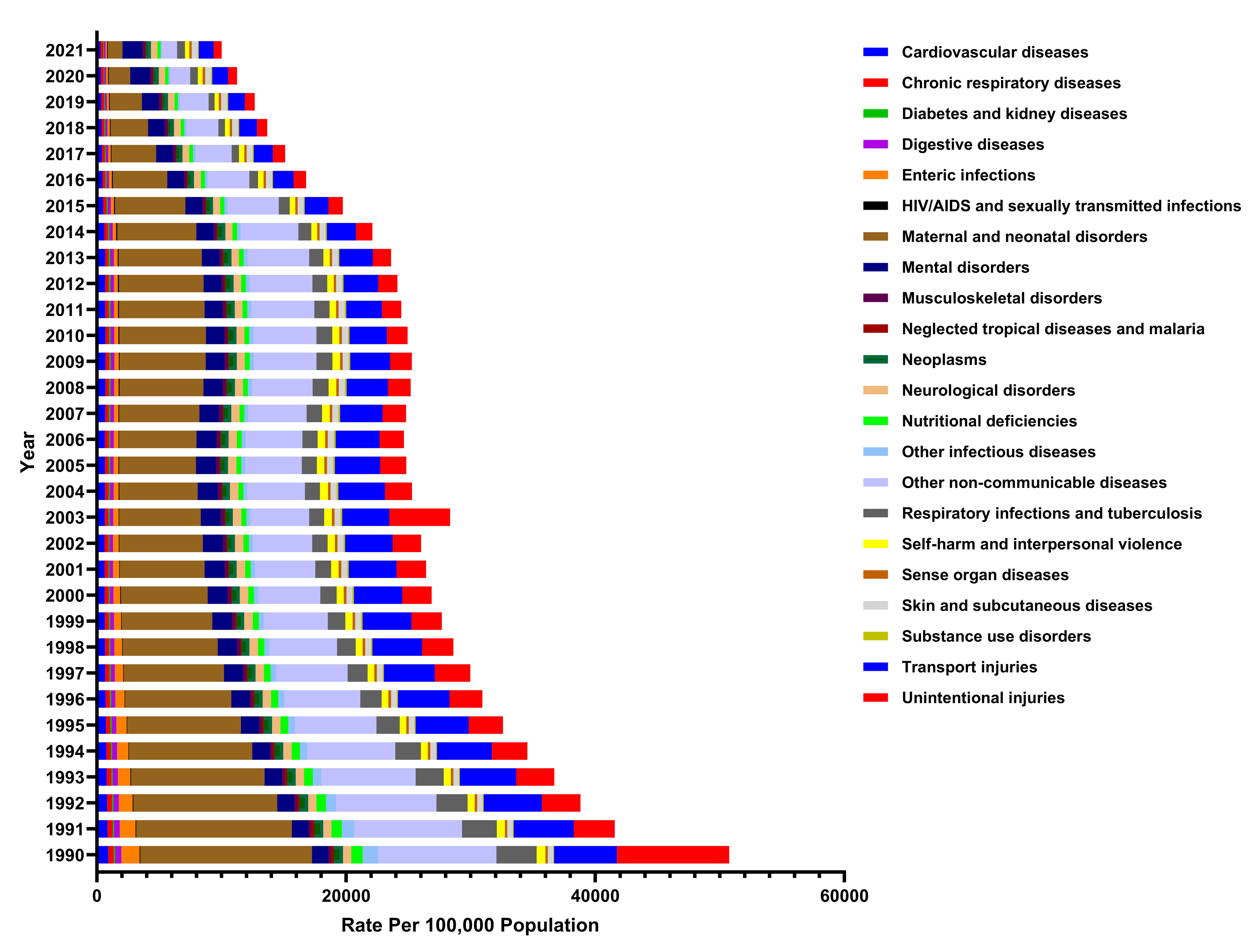

Supplement: S14 Fig — (TIF) [file pone.0325085.s014.tif]

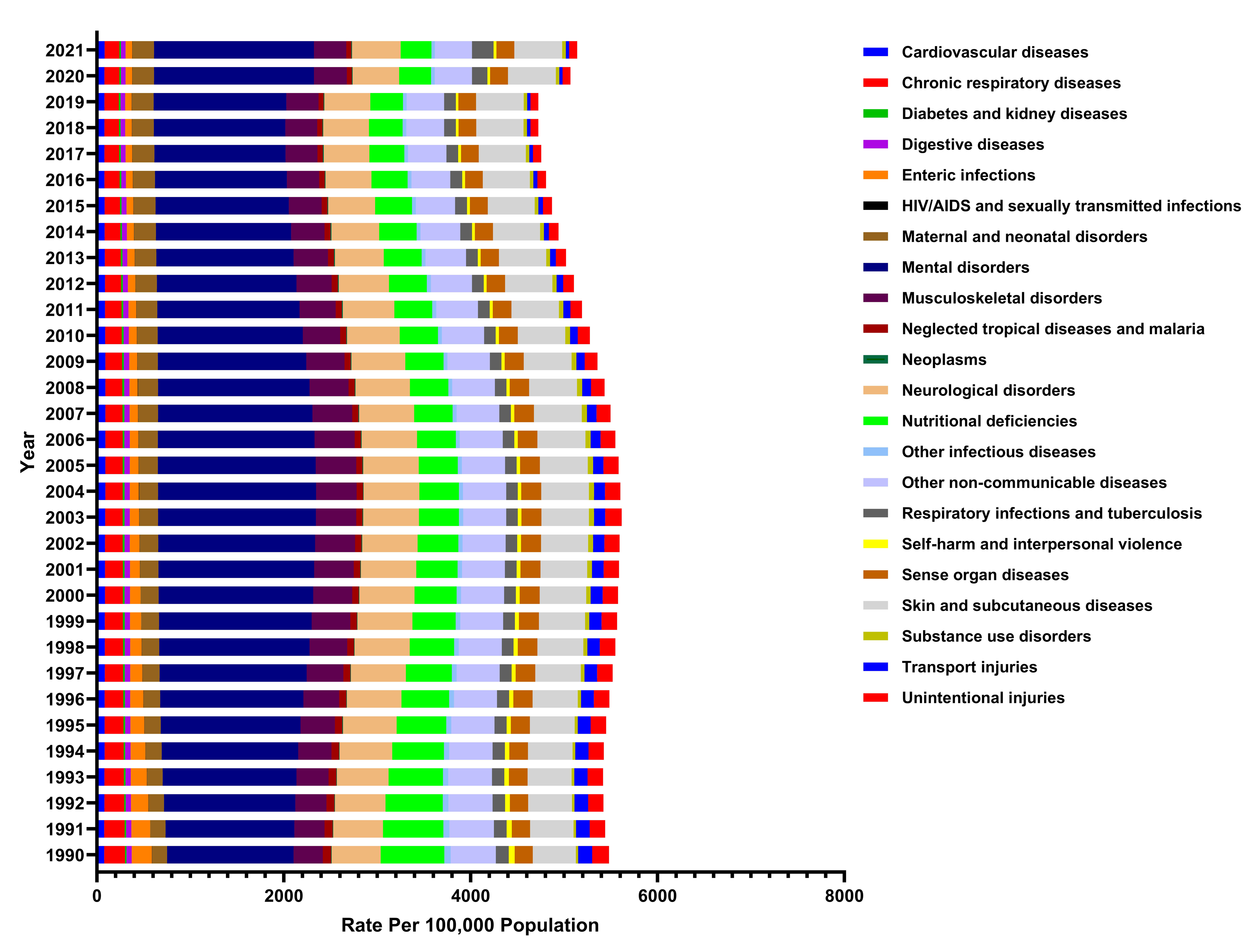

Supplement: S15 Fig — (TIF) [file pone.0325085.s015.tif]

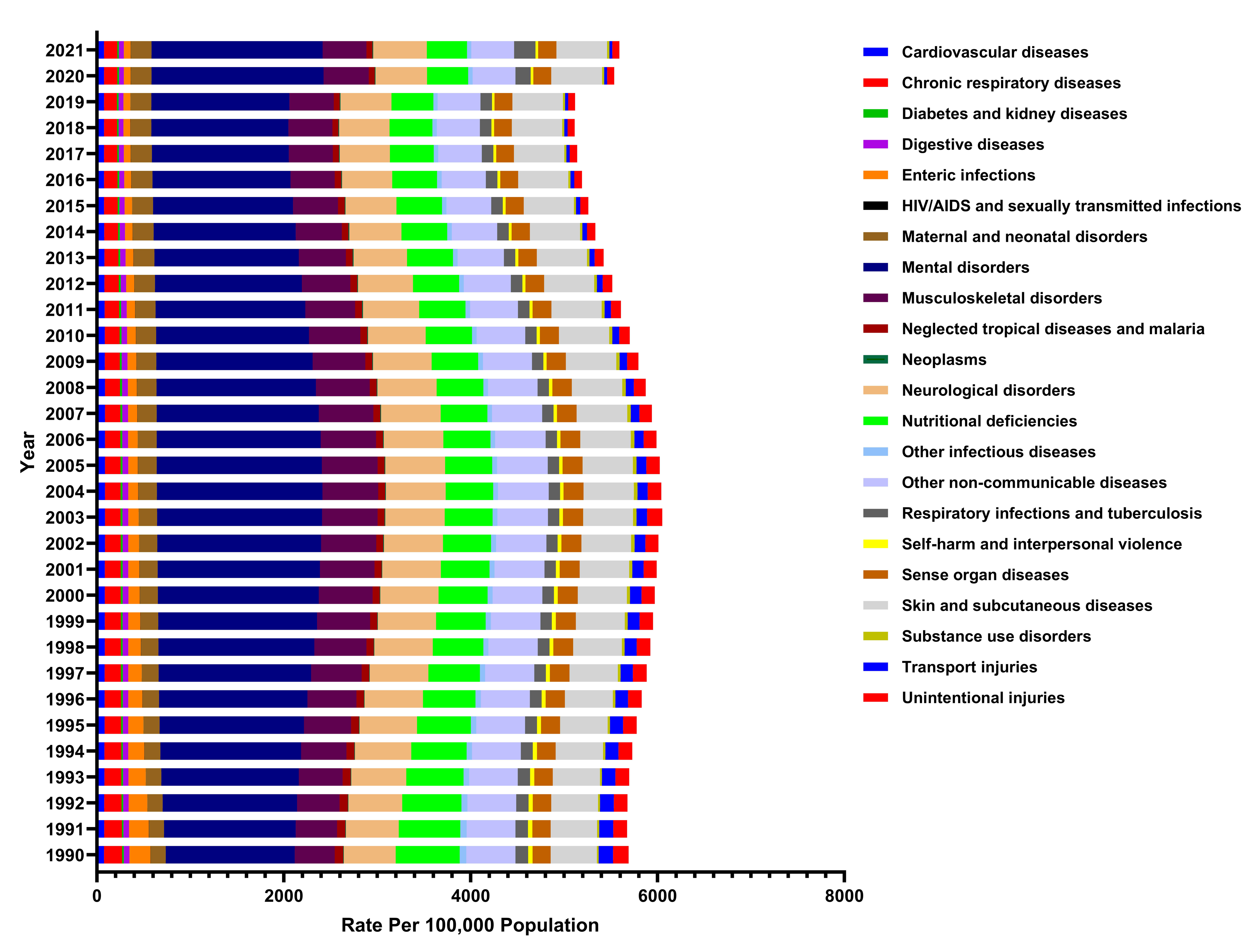

Supplement: S16 Fig — (TIF) [file pone.0325085.s016.tif]

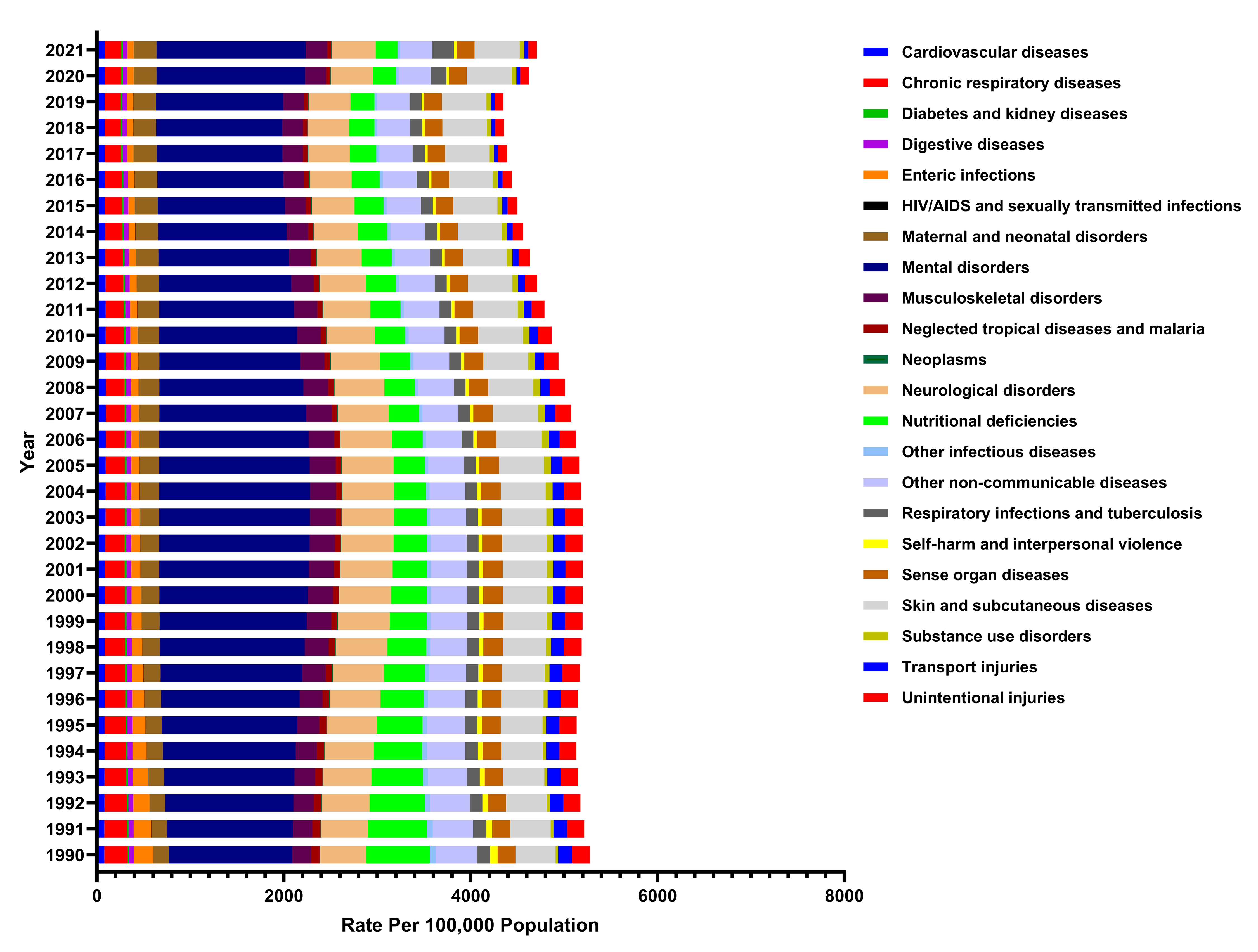

Supplement: S17 Fig — (TIF) [file pone.0325085.s017.tif]

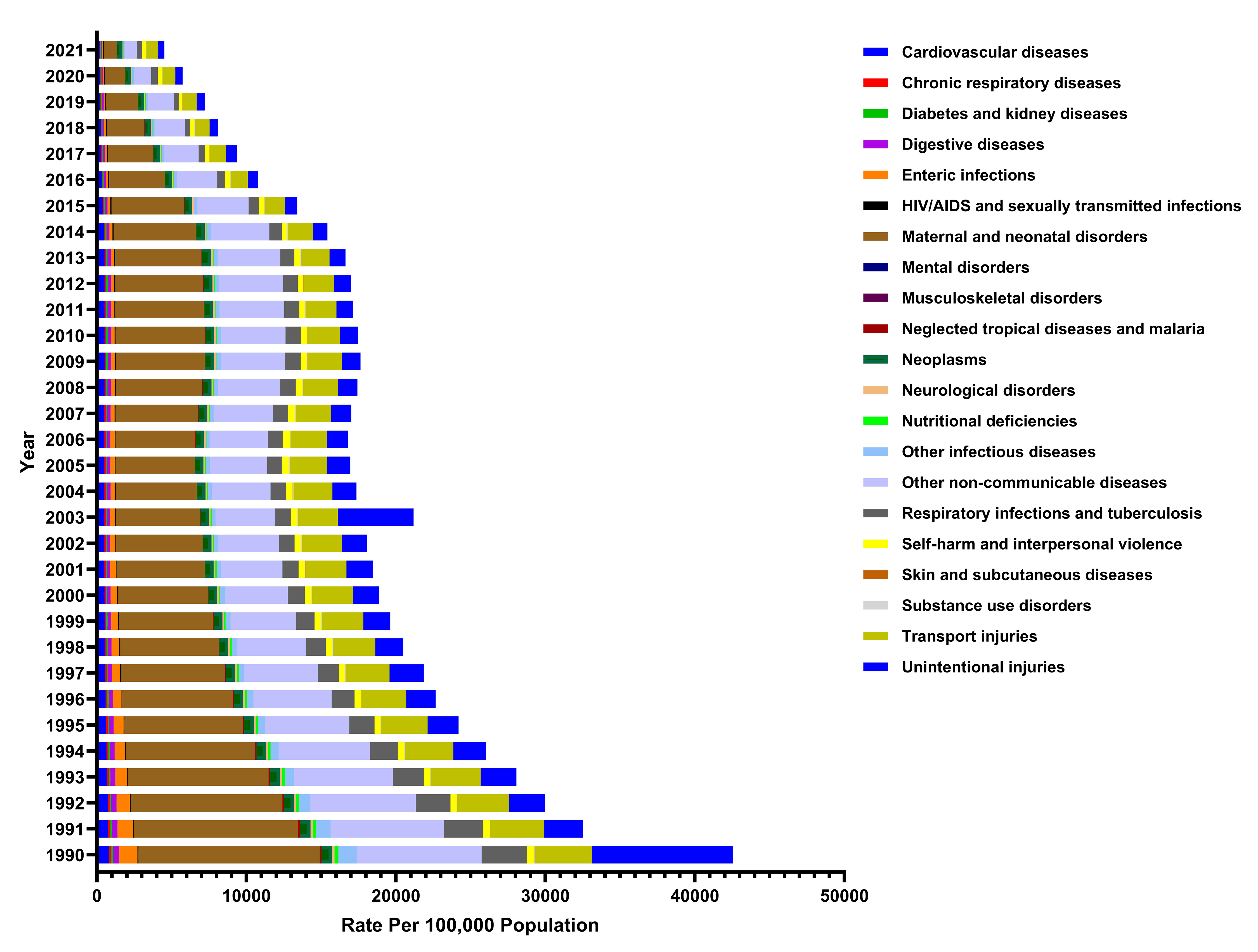

Supplement: S18 Fig — (TIF) [file pone.0325085.s018.tif]

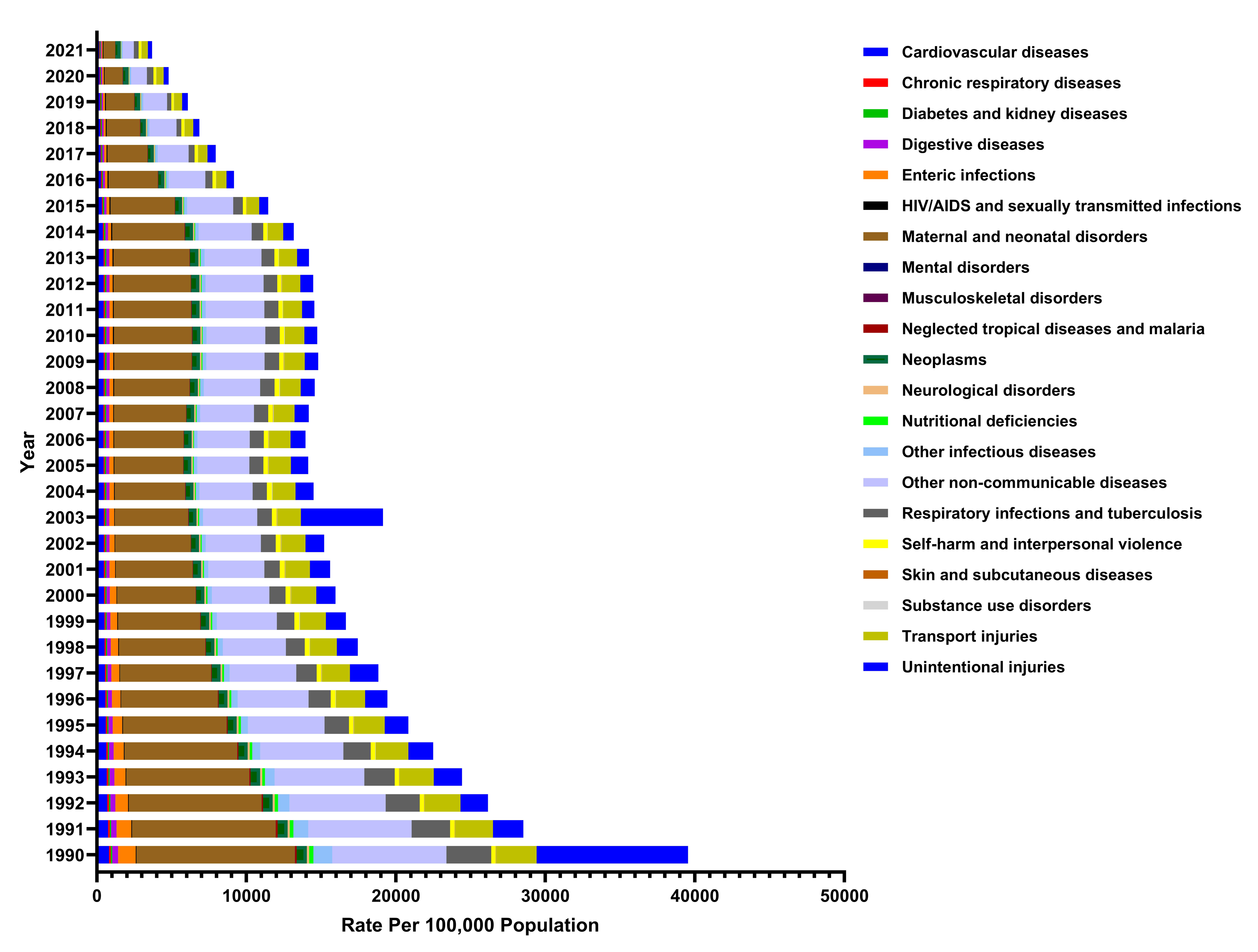

Supplement: S19 Fig — (TIF) [file pone.0325085.s019.tif]

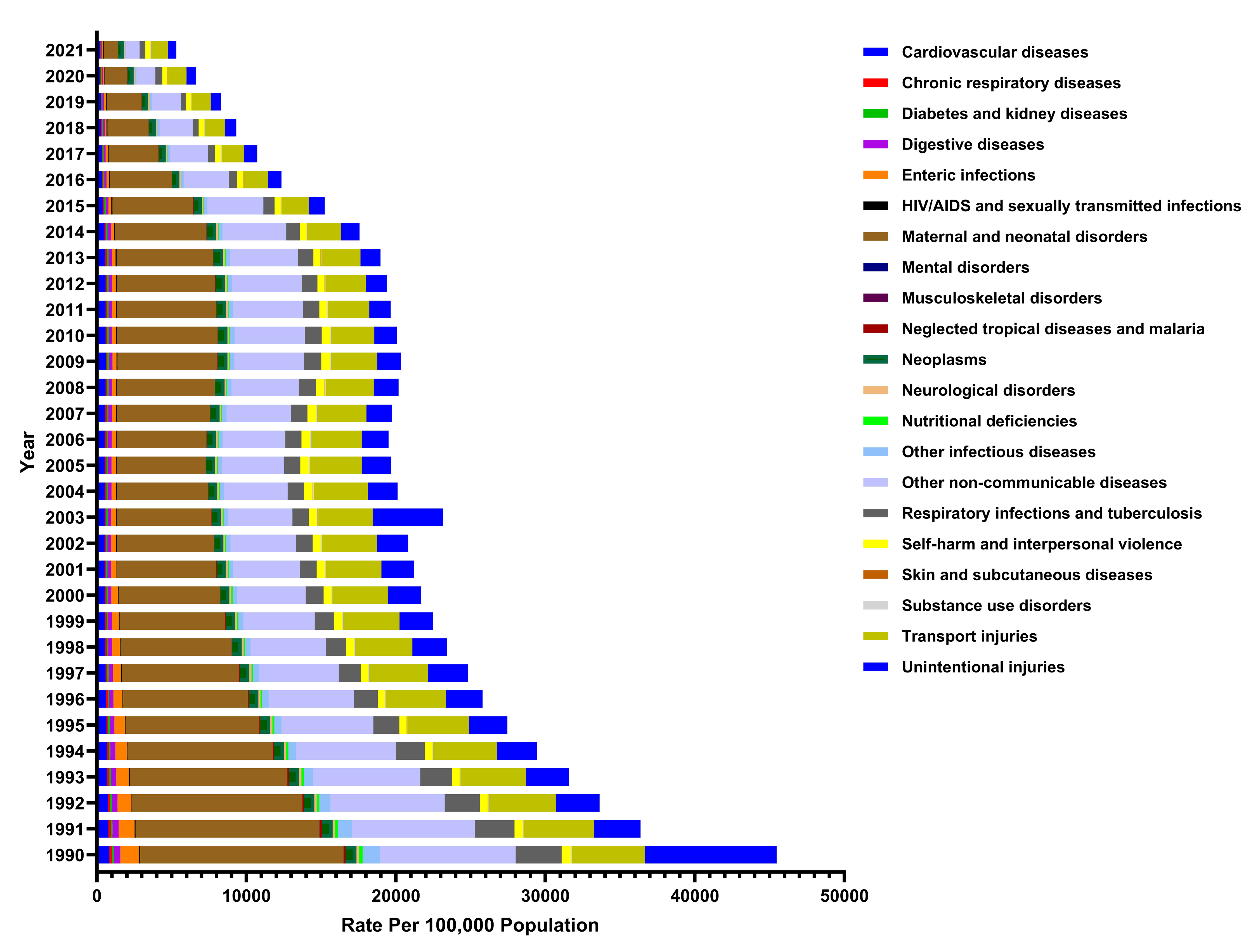

Supplement: S20 Fig — (TIF) [file pone.0325085.s020.tif]

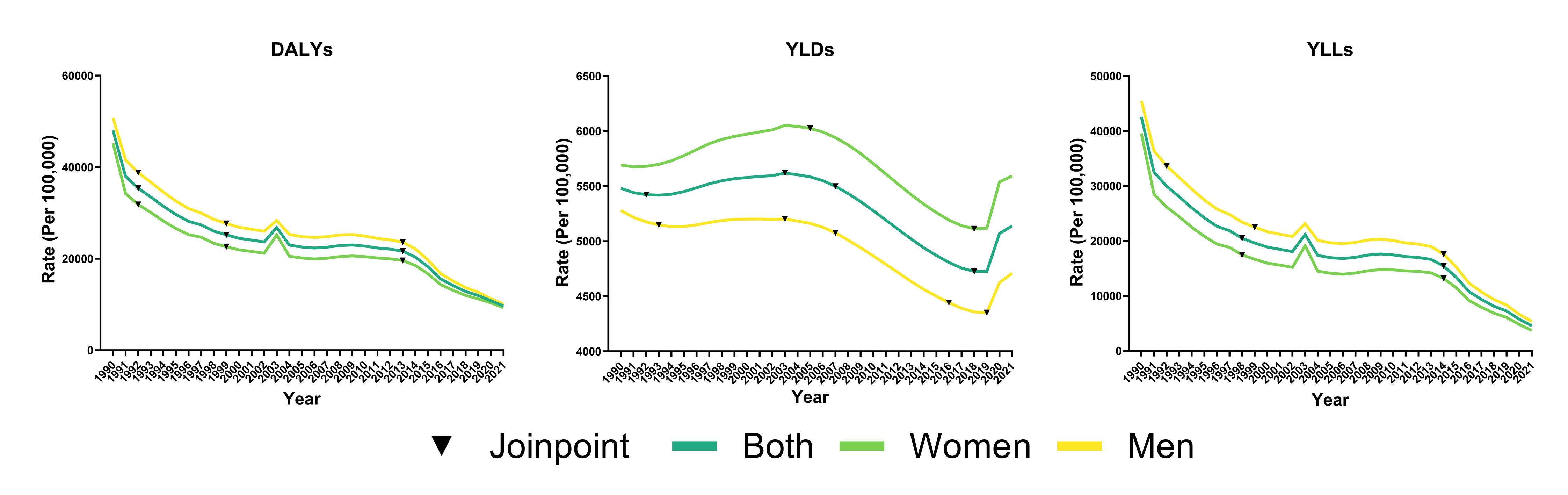

Supplement: S21 Fig — (TIF) [file pone.0325085.s021.tif]
