# Supplementary material for: Disability-adjusted life years, years lived with disability, and years of life lost of diseases among children and adolescents in national and subnational levels of Iran, 1990–2021: A systematic analysis for the Global Burden of Disease 2021
Source: PLoS One. 2025 Jun 23;20(6):e0325085. doi: 10.1371/journal.pone.0325085 (PMC12184942; doi:10.1371/journal.pone.0325085)
Supplement: S1 Table — (DOCX) [file pone.0325085.s022.docx]

**S1 Table.** Subnational distribution of rate of disability-adjusted life years (DALYs), years lived with disability (YLDs), and years of life lost (YLLs) of child and adolescents causes of death by sex in 1990 and 2021 and overall percent change over 1990–2021 in Iran

| Province | Cause | Measure | Metric | Year | | | | | | % Change (1990 to 2021) | | |
| --- | --- | --- | --- | --- | --- | --- | --- | --- | --- | --- | --- | --- |
|  |  |  |  | 1990 | | | 2021 | | |  |  |  |
|  |  |  |  | Both | Females | Males | Both | Females | Males | Both | Females | Males |
| Alborz | All causes | DALYs (Disability-Adjusted Life Years) | Number | 220097.7 (196959.2 to 246867.4) | 93099 (83077.7 to 104775.6) | 126998.7 (113231.1 to 141767.7) | 68127 (56603 to 82293) | 32074.7 (25862.8 to 39733.6) | 36052.3 (30223.6 to 42603.8) | -69 (-74.3 to -63.4) | -65.5 (-71.6 to -58.8) | -71.6 (-76.5 to -66.8) |
|  |  |  | Rate | 27883.4 (24952.1 to 31274.8) | 24122.8 (21526.2 to 27148.3) | 31481.1 (28068.3 to 35142.1) | 8647.4 (7184.7 to 10445.5) | 8383.7 (6760.1 to 10385.6) | 8896.4 (7458.1 to 10513.1) | -69 (-74.3 to -63.3) | -65.2 (-71.4 to -58.4) | -71.7 (-76.6 to -66.9) |
|  |  | YLDs (Years Lived with Disability) | Number | 40864.5 (29936.5 to 54821.3) | 20824.2 (15155.9 to 27735.7) | 20040.4 (14553.3 to 26703.8) | 41082.5 (29636.7 to 54734.7) | 21799.4 (15682.9 to 29162.8) | 19283.1 (13987.4 to 25639.7) | 0.5 (-7.2 to 10.5) | 4.7 (-4.9 to 15.4) | -3.8 (-14.5 to 8.4) |
|  |  |  | Rate | 5177 (3792.6 to 6945.1) | 5395.7 (3927 to 7186.6) | 4967.7 (3607.5 to 6619.5) | 5214.6 (3761.8 to 6947.5) | 5697.9 (4099.2 to 7622.6) | 4758.4 (3451.6 to 6326.9) | 0.7 (-7 to 10.7) | 5.6 (-4.1 to 16.4) | -4.2 (-14.9 to 7.9) |
|  |  | YLLs (Years of Life Lost) | Number | 179233.2 (157727.3 to 201311.9) | 72274.9 (63183.3 to 81682.7) | 106958.3 (93545.1 to 120031.5) | 27044.6 (24189 to 30035.9) | 10275.3 (9124.3 to 11509.7) | 16769.3 (14670.7 to 18904.5) | -84.9 (-87.3 to -82.1) | -85.8 (-88.1 to -82.9) | -84.3 (-87.1 to -81.2) |
|  |  |  | Rate | 22706.4 (19981.9 to 25503.5) | 18727.1 (16371.4 to 21164.8) | 26513.4 (23188.4 to 29754) | 3432.8 (3070.3 to 3812.5) | 2685.8 (2384.9 to 3008.4) | 4138 (3620.2 to 4664.9) | -84.9 (-87.3 to -82.1) | -85.7 (-88 to -82.8) | -84.4 (-87.1 to -81.3) |
|  | Communicable, maternal, neonatal, and nutritional diseases | DALYs (Disability-Adjusted Life Years) | Number | 100883.1 (84978.7 to 123590.9) | 42738.9 (35659.6 to 53916) | 58144.1 (48329.9 to 73094.1) | 14700.6 (12112 to 17996.7) | 7086.8 (5726.7 to 8956) | 7613.8 (6339.7 to 9275.2) | -85.4 (-88.9 to -81.3) | -83.4 (-87.8 to -78.3) | -86.9 (-90.4 to -83) |
|  |  |  | Rate | 12780.5 (10765.7 to 15657.3) | 11074.1 (9239.7 to 13970.2) | 14413.1 (11980.3 to 18119) | 1866 (1537.4 to 2284.3) | 1852.3 (1496.8 to 2340.9) | 1878.8 (1564.4 to 2288.8) | -85.4 (-88.9 to -81.3) | -83.3 (-87.7 to -78.1) | -87 (-90.5 to -83.1) |
|  |  | YLDs (Years Lived with Disability) | Number | 9176.9 (6249.9 to 12936.1) | 4665.5 (3102.5 to 6707.7) | 4511.4 (2842.5 to 6879.3) | 6795.1 (4659.5 to 9639.5) | 3754 (2524.9 to 5589.4) | 3041.1 (2101.7 to 4555.4) | -26 (-42.9 to 0.5) | -19.5 (-38.8 to 10.6) | -32.6 (-54.7 to -2.9) |
|  |  |  | Rate | 1162.6 (791.8 to 1638.8) | 1208.9 (803.9 to 1738) | 1118.3 (704.6 to 1705.3) | 862.5 (591.4 to 1223.6) | 981.2 (660 to 1461) | 750.4 (518.6 to 1124.1) | -25.8 (-42.7 to 0.7) | -18.8 (-38.2 to 11.6) | -32.9 (-54.9 to -3.4) |
|  |  | YLLs (Years of Life Lost) | Number | 91706.2 (76105.4 to 114007.3) | 38073.5 (31376.8 to 48508.5) | 53632.7 (44342.3 to 68712.4) | 7905.5 (6704.8 to 9170.1) | 3332.7 (2758.6 to 3935.4) | 4572.7 (3837.7 to 5359.8) | -91.4 (-93.6 to -89) | -91.2 (-94 to -88.5) | -91.5 (-94.1 to -88.8) |
|  |  |  | Rate | 11617.9 (9641.5 to 14443.2) | 9865.2 (8130 to 12569) | 13294.8 (10991.8 to 17032.8) | 1003.4 (851.1 to 1164) | 871.1 (721 to 1028.6) | 1128.4 (947 to 1322.6) | -91.4 (-93.6 to -89) | -91.2 (-93.9 to -88.4) | -91.5 (-94.1 to -88.9) |
|  | Injuries | DALYs (Disability-Adjusted Life Years) | Number | 36938.2 (31415.1 to 42154.5) | 12739.8 (10347.9 to 15147.9) | 24198.4 (20518.4 to 28251.8) | 11006.2 (9654.5 to 12369.4) | 3452.2 (3064.4 to 3891.4) | 7554 (6382.7 to 8745) | -70.2 (-75.6 to -63.6) | -72.9 (-78.4 to -65.9) | -68.8 (-75.2 to -60.3) |
|  |  |  | Rate | 4679.6 (3979.9 to 5340.4) | 3301 (2681.3 to 3925) | 5998.4 (5086.2 to 7003.2) | 1397 (1225.5 to 1570.1) | 902.3 (801 to 1017.1) | 1864.1 (1575 to 2158) | -70.1 (-75.6 to -63.6) | -72.7 (-78.2 to -65.6) | -68.9 (-75.4 to -60.5) |
|  |  | YLDs (Years Lived with Disability) | Number | 2506 (1835.1 to 3345.3) | 1105.4 (804.7 to 1482.5) | 1400.6 (1025.1 to 1858.2) | 1233.7 (896.4 to 1670) | 535.9 (387 to 718.7) | 697.8 (502.9 to 941.7) | -50.8 (-54.2 to -47.4) | -51.5 (-56 to -46.8) | -50.2 (-54.1 to -46.5) |
|  |  |  | Rate | 317.5 (232.5 to 423.8) | 286.4 (208.5 to 384.1) | 347.2 (254.1 to 460.6) | 156.6 (113.8 to 212) | 140.1 (101.2 to 187.9) | 172.2 (124.1 to 232.4) | -50.7 (-54.1 to -47.3) | -51.1 (-55.6 to -46.3) | -50.4 (-54.3 to -46.7) |
|  |  | YLLs (Years of Life Lost) | Number | 34432.2 (28858.3 to 39583.9) | 11634.4 (9257.2 to 14009.6) | 22797.7 (19043.5 to 26853.5) | 9772.6 (8446.4 to 11051.7) | 2916.3 (2538 to 3308.4) | 6856.2 (5679.1 to 8086.3) | -71.6 (-77.2 to -64.8) | -74.9 (-80.6 to -67.6) | -69.9 (-76.7 to -60.9) |
|  |  |  | Rate | 4362.1 (3656 to 5014.7) | 3014.6 (2398.6 to 3630) | 5651.2 (4720.6 to 6656.6) | 1240.4 (1072.1 to 1402.8) | 762.3 (663.4 to 864.7) | 1691.9 (1401.4 to 1995.4) | -71.6 (-77.1 to -64.7) | -74.7 (-80.4 to -67.3) | -70.1 (-76.9 to -61.1) |
|  | Non-communicable diseases | DALYs (Disability-Adjusted Life Years) | Number | 82276.4 (63049.6 to 97554.9) | 37620.3 (27547.6 to 45019) | 44656.2 (31687.8 to 53638.2) | 42155 (33012.4 to 53350.2) | 21478.9 (16645.2 to 27586.6) | 20676.1 (16203.2 to 25886.6) | -48.8 (-59.7 to -31.1) | -42.9 (-54.2 to -23.5) | -53.7 (-64.4 to -33.7) |
|  |  |  | Rate | 10423.3 (7987.5 to 12358.9) | 9747.8 (7137.8 to 11664.8) | 11069.6 (7854.9 to 13296.1) | 5350.8 (4190.3 to 6771.8) | 5614.2 (4350.7 to 7210.6) | 5102.1 (3998.3 to 6387.9) | -48.7 (-59.6 to -31) | -42.4 (-53.8 to -22.8) | -53.9 (-64.6 to -34) |
|  |  | YLDs (Years Lived with Disability) | Number | 29181.6 (21108.1 to 39206.5) | 15053.3 (10904.7 to 20085.4) | 14128.3 (10136.7 to 18988.6) | 33053.7 (23872.1 to 44328.6) | 17509.5 (12571.8 to 23582.6) | 15544.2 (11199.6 to 21004.8) | 13.3 (4.8 to 23.3) | 16.3 (7.7 to 26.5) | 10 (0.7 to 21) |
|  |  |  | Rate | 3696.9 (2674.1 to 4966.9) | 3900.5 (2825.5 to 5204.3) | 3502.2 (2512.7 to 4707) | 4195.5 (3030.1 to 5626.7) | 4576.6 (3286 to 6164) | 3835.7 (2763.7 to 5183.2) | 13.5 (5 to 23.6) | 17.3 (8.6 to 27.6) | 9.5 (0.2 to 20.5) |
|  |  | YLLs (Years of Life Lost) | Number | 53094.8 (34855.4 to 64652) | 22567 (14064.5 to 27709.1) | 30527.8 (18660.9 to 37931) | 9101.3 (7956.4 to 10320.6) | 3969.4 (3424.2 to 4707.2) | 5131.9 (4337.1 to 5999.3) | -82.9 (-86.8 to -72.2) | -82.4 (-86.5 to -69.1) | -83.2 (-87.5 to -69.3) |
|  |  |  | Rate | 6726.4 (4415.7 to 8190.5) | 5847.3 (3644.3 to 7179.7) | 7567.4 (4625.8 to 9402.5) | 1155.2 (1009.9 to 1310) | 1037.5 (895 to 1230.4) | 1266.4 (1070.2 to 1480.4) | -82.8 (-86.7 to -72.2) | -82.3 (-86.4 to -68.8) | -83.3 (-87.5 to -69.4) |
| Ardebil | All causes | DALYs (Disability-Adjusted Life Years) | Number | 415450.7 (373405.4 to 460170.7) | 186063.8 (165541.7 to 206828.1) | 229387 (205300.8 to 254807.2) | 36571.7 (30955.3 to 43053.8) | 16921.8 (13969.8 to 20246.3) | 19649.8 (16935.2 to 22729.8) | -91.2 (-92.7 to -89.7) | -90.9 (-92.6 to -89.1) | -91.4 (-92.9 to -89.9) |
|  |  |  | Rate | 59789.8 (53738.9 to 66225.8) | 54994.6 (48928.9 to 61131.9) | 64340.4 (57584.5 to 71470.5) | 9696.6 (8207.5 to 11415.2) | 9302.2 (7679.4 to 11129.7) | 10063.9 (8673.6 to 11641.4) | -83.8 (-86.6 to -81) | -83.1 (-86.3 to -79.8) | -84.4 (-87.1 to -81.6) |
|  |  | YLDs (Years Lived with Disability) | Number | 38832.6 (28542.3 to 51538.1) | 19233.3 (14187.6 to 25566.6) | 19599.3 (14318.3 to 26024.3) | 19189.8 (14029.4 to 25576.1) | 10024.4 (7349.2 to 13235.8) | 9165.4 (6702.5 to 12042.8) | -50.6 (-54.6 to -45.9) | -47.9 (-52.7 to -42.9) | -53.2 (-58.1 to -47.8) |
|  |  |  | Rate | 5588.6 (4107.7 to 7417.1) | 5684.8 (4193.4 to 7556.7) | 5497.4 (4016.1 to 7299.5) | 5088 (3719.7 to 6781.2) | 5510.6 (4040 to 7275.9) | 4694.2 (3432.8 to 6167.9) | -9 (-16.4 to -0.4) | -3.1 (-12.1 to 6.2) | -14.6 (-23.5 to -4.6) |
|  |  | YLLs (Years of Life Lost) | Number | 376618.1 (334905.5 to 417975.4) | 166830.5 (147691.8 to 186660.5) | 209787.6 (186373.6 to 232625.4) | 17381.9 (15582.1 to 19067.1) | 6897.4 (6239.3 to 7600.9) | 10484.5 (9256.2 to 11649.7) | -95.4 (-96.1 to -94.6) | -95.9 (-96.5 to -95.2) | -95 (-95.8 to -94.1) |
|  |  |  | Rate | 54201.2 (48198.1 to 60153.2) | 49309.8 (43653 to 55171) | 58843.1 (52275.7 to 65248.8) | 4608.6 (4131.4 to 5055.4) | 3791.6 (3429.8 to 4178.4) | 5369.8 (4740.7 to 5966.6) | -91.5 (-92.8 to -90.1) | -92.3 (-93.5 to -91.1) | -90.9 (-92.3 to -89.3) |
|  | Communicable, maternal, neonatal, and nutritional diseases | DALYs (Disability-Adjusted Life Years) | Number | 172536.9 (138184.3 to 224702.5) | 76544.8 (59833.7 to 105706.7) | 95992.1 (75124.7 to 122577.4) | 9083.7 (7662.5 to 10768.2) | 4340 (3606.8 to 5304.6) | 4743.7 (3909.1 to 5764.4) | -94.7 (-96.1 to -93.1) | -94.3 (-96.2 to -92.5) | -95.1 (-96.6 to -93.4) |
|  |  |  | Rate | 24830.8 (19886.9 to 32338.2) | 22624.2 (17685 to 31243.6) | 26924.7 (21071.6 to 34381.6) | 2408.4 (2031.6 to 2855.1) | 2385.8 (1982.7 to 2916) | 2429.5 (2002.1 to 2952.3) | -90.3 (-92.9 to -87.4) | -89.5 (-93 to -86) | -91 (-93.8 to -88) |
|  |  | YLDs (Years Lived with Disability) | Number | 8965.5 (6037.5 to 12936.6) | 4200.4 (2748 to 6112.8) | 4765.1 (3062.2 to 7039.4) | 3486.9 (2438.6 to 5041) | 1859 (1262.6 to 2733.1) | 1628 (1085 to 2389.1) | -61.1 (-69.9 to -48.7) | -55.7 (-66.2 to -40.5) | -65.8 (-76 to -48.5) |
|  |  |  | Rate | 1290.3 (868.9 to 1861.8) | 1241.5 (812.2 to 1806.8) | 1336.6 (858.9 to 1974.5) | 924.5 (646.6 to 1336.6) | 1021.9 (694.1 to 1502.5) | 833.8 (555.7 to 1223.6) | -28.3 (-44.5 to -5.4) | -17.7 (-37 to 10.7) | -37.6 (-56.2 to -5.9) |
|  |  | YLLs (Years of Life Lost) | Number | 163571.4 (129023.8 to 214416.3) | 72344.3 (55958.5 to 101754.5) | 91227 (70474.8 to 117932.5) | 5596.8 (4666.9 to 6630.1) | 2481.1 (1977.5 to 3000.9) | 3115.7 (2543.9 to 3706.2) | -96.6 (-97.7 to -95.5) | -96.6 (-98 to -95.4) | -96.6 (-97.7 to -95.5) |
|  |  |  | Rate | 23540.5 (18568.5 to 30857.8) | 21382.7 (16539.6 to 30075.4) | 25588.2 (19767.4 to 33078.7) | 1483.9 (1237.4 to 1757.9) | 1363.9 (1087.1 to 1649.6) | 1595.8 (1302.9 to 1898.2) | -93.7 (-95.8 to -91.7) | -93.6 (-96.2 to -91.5) | -93.8 (-95.9 to -91.7) |
|  | Injuries | DALYs (Disability-Adjusted Life Years) | Number | 71823.6 (59111.1 to 87034.3) | 28593.2 (23412.4 to 37192.2) | 43230.4 (34825.8 to 55993.6) | 5142.9 (4440.9 to 5850.7) | 1534 (1351.3 to 1768.7) | 3608.9 (3030 to 4205.6) | -92.8 (-94.4 to -91.1) | -94.6 (-96 to -93.2) | -91.7 (-94 to -89.4) |
|  |  |  | Rate | 10336.5 (8507 to 12525.6) | 8451.3 (6920 to 10992.8) | 12125.6 (9768.2 to 15705.6) | 1363.6 (1177.5 to 1551.2) | 843.3 (742.8 to 972.3) | 1848.3 (1551.9 to 2153.9) | -86.8 (-89.8 to -83.7) | -90 (-92.6 to -87.4) | -84.8 (-89 to -80.6) |
|  |  | YLDs (Years Lived with Disability) | Number | 2874.1 (2125.1 to 3815.6) | 1241.5 (919.1 to 1639) | 1632.6 (1195.8 to 2147.6) | 494.5 (363.6 to 660.4) | 214.8 (158.4 to 287) | 279.7 (203.7 to 377.4) | -82.8 (-84.3 to -81.2) | -82.7 (-84.4 to -80.7) | -82.9 (-84.7 to -81.3) |
|  |  |  | Rate | 413.6 (305.8 to 549.1) | 366.9 (271.7 to 484.4) | 457.9 (335.4 to 602.4) | 131.1 (96.4 to 175.1) | 118.1 (87.1 to 157.8) | 143.2 (104.3 to 193.3) | -68.3 (-71 to -65.4) | -67.8 (-70.9 to -64.1) | -68.7 (-72 to -65.9) |
|  |  | YLLs (Years of Life Lost) | Number | 68949.5 (56052.3 to 84201.3) | 27351.7 (22170.2 to 36079.8) | 41597.8 (33160.8 to 54368) | 4648.4 (3990.4 to 5363.7) | 1319.2 (1147.7 to 1545.8) | 3329.2 (2764.3 to 3935.6) | -93.3 (-94.9 to -91.6) | -95.2 (-96.5 to -93.8) | -92 (-94.3 to -89.7) |
|  |  |  | Rate | 9922.9 (8066.8 to 12117.9) | 8084.3 (6552.8 to 10664) | 11667.7 (9301.2 to 15249.6) | 1232.5 (1058 to 1422.1) | 725.2 (630.9 to 849.7) | 1705.1 (1415.8 to 2015.7) | -87.6 (-90.5 to -84.5) | -91 (-93.5 to -88.4) | -85.4 (-89.6 to -81.2) |
|  | Non-communicable diseases | DALYs (Disability-Adjusted Life Years) | Number | 171090.2 (127229.7 to 206750.7) | 80925.8 (49997.2 to 98633.9) | 90164.4 (62859.4 to 110803.4) | 22160.8 (17631.6 to 27496.6) | 10991.6 (8511.2 to 13948.1) | 11169.2 (9063.3 to 13602.8) | -87 (-90.3 to -81.1) | -86.4 (-90.1 to -77.3) | -87.6 (-90.9 to -80.2) |
|  |  |  | Rate | 24622.6 (18310.3 to 29754.7) | 23919.1 (14777.6 to 29153.1) | 25290.1 (17631.4 to 31079.1) | 5875.7 (4674.8 to 7290.4) | 6042.3 (4678.7 to 7667.5) | 5720.5 (4641.9 to 6966.9) | -76.1 (-82.1 to -65.2) | -74.7 (-81.6 to -57.8) | -77.4 (-83.3 to -63.9) |
|  |  | YLDs (Years Lived with Disability) | Number | 26993 (19932.2 to 36259.7) | 13791.4 (10114 to 18596.9) | 13201.6 (9791.6 to 17709.6) | 15208.4 (11010.1 to 20466) | 7950.7 (5705.2 to 10836) | 7257.7 (5340.8 to 9528.5) | -43.7 (-48.6 to -38.3) | -42.4 (-47.2 to -37.1) | -45 (-50 to -39.3) |
|  |  |  | Rate | 3884.7 (2868.5 to 5218.3) | 4076.3 (2989.4 to 5496.7) | 3702.9 (2746.4 to 4967.3) | 4032.3 (2919.2 to 5426.3) | 4370.6 (3136.3 to 5956.7) | 3717.1 (2735.4 to 4880.1) | 3.8 (-5.4 to 13.6) | 7.2 (-1.8 to 16.9) | 0.4 (-8.8 to 10.8) |
|  |  | YLLs (Years of Life Lost) | Number | 144097.2 (98553.4 to 178861.5) | 67134.4 (36020.9 to 84200.1) | 76962.8 (50324.7 to 97790.5) | 6952.4 (5777.4 to 8296.9) | 3040.9 (2500.6 to 3551.3) | 3911.5 (3157.1 to 4791.7) | -95.2 (-96.2 to -92.3) | -95.5 (-96.5 to -91) | -94.9 (-96.1 to -90.9) |
|  |  |  | Rate | 20737.8 (14183.4 to 25741) | 19842.8 (10646.6 to 24886.9) | 21587.2 (14115.5 to 27429.1) | 1843.4 (1531.8 to 2199.8) | 1671.6 (1374.6 to 1952.2) | 2003.3 (1616.9 to 2454.1) | -91.1 (-92.9 to -85.8) | -91.6 (-93.4 to -83.2) | -90.7 (-92.9 to -83.3) |
| Bushehr | All causes | DALYs (Disability-Adjusted Life Years) | Number | 150885.8 (134454.4 to 168531.3) | 69998.5 (62173.3 to 78326.9) | 80887.3 (70799.6 to 90636.4) | 40769.4 (34016.6 to 48594.5) | 19419.7 (15908.3 to 23656.3) | 21349.6 (17885.9 to 25065.2) | -73 (-78 to -68) | -72.3 (-77.5 to -66.9) | -73.6 (-78.3 to -68.8) |
|  |  |  | Rate | 37585.8 (33492.8 to 41981.4) | 35521.4 (31550.4 to 39747.7) | 39576.4 (34640.7 to 44346.4) | 9151.1 (7635.4 to 10907.5) | 9027.7 (7395.3 to 10997.2) | 9266.3 (7762.9 to 10879) | -75.7 (-80.2 to -71.2) | -74.6 (-79.4 to -69.7) | -76.6 (-80.7 to -72.3) |
|  |  | YLDs (Years Lived with Disability) | Number | 21430.9 (15604.7 to 28908.5) | 11042.7 (8014.4 to 14924.5) | 10388.3 (7705.4 to 14086.8) | 23063.4 (16774.8 to 30863.5) | 12184.5 (8784.5 to 16217.4) | 10878.9 (7978.6 to 14630.1) | 7.6 (-1.7 to 17.7) | 10.3 (0.4 to 22.1) | 4.7 (-6.5 to 16.6) |
|  |  |  | Rate | 5338.5 (3887.2 to 7201.1) | 5603.7 (4067 to 7573.6) | 5082.7 (3770.1 to 6892.4) | 5176.8 (3765.3 to 6927.6) | 5664.2 (4083.7 to 7539) | 4721.7 (3462.9 to 6349.8) | -3 (-11.4 to 6.1) | 1.1 (-8 to 11.9) | -7.1 (-17 to 3.4) |
|  |  | YLLs (Years of Life Lost) | Number | 129454.8 (113373.1 to 144488.3) | 58955.8 (51563.1 to 66313.7) | 70499 (61298.9 to 79220.1) | 17706 (15987.1 to 19526.3) | 7235.2 (6542.4 to 8013.6) | 10470.8 (9315.5 to 11679.7) | -86.3 (-88.5 to -83.8) | -87.7 (-89.7 to -85.2) | -85.1 (-87.6 to -82.1) |
|  |  |  | Rate | 32247.4 (28241.4 to 35992.2) | 29917.7 (26166.1 to 33651.5) | 34493.6 (29992.2 to 38760.7) | 3974.3 (3588.5 to 4382.9) | 3363.4 (3041.4 to 3725.3) | 4544.6 (4043.2 to 5069.3) | -87.7 (-89.6 to -85.4) | -88.8 (-90.6 to -86.4) | -86.8 (-89 to -84.1) |
|  | Communicable, maternal, neonatal, and nutritional diseases | DALYs (Disability-Adjusted Life Years) | Number | 73178.4 (62481.5 to 89063.2) | 34020.3 (28465.4 to 43175.1) | 39158.1 (32819.7 to 48138.9) | 10406.5 (8834.6 to 12415.3) | 5139.4 (4173.7 to 6243.6) | 5267.1 (4430.6 to 6368.1) | -85.8 (-89.3 to -82) | -84.9 (-89.3 to -81) | -86.5 (-90.1 to -82.9) |
|  |  |  | Rate | 18228.8 (15564.2 to 22185.8) | 17263.9 (14445 to 21909.6) | 19159.2 (16058 to 23553.3) | 2335.8 (1983 to 2786.7) | 2389.2 (1940.3 to 2902.5) | 2286.1 (1923 to 2763.9) | -87.2 (-90.3 to -83.7) | -86.2 (-90.2 to -82.5) | -88.1 (-91.2 to -84.8) |
|  |  | YLDs (Years Lived with Disability) | Number | 5453.8 (3839 to 7828.3) | 2759.3 (1849.7 to 4030) | 2694.5 (1807.5 to 3977.3) | 4308.7 (2976.4 to 6130.1) | 2397.2 (1587.4 to 3412.3) | 1911.6 (1328.4 to 2772.6) | -21 (-38.3 to 3.8) | -13.1 (-33.9 to 16.6) | -29.1 (-48.9 to -2.9) |
|  |  |  | Rate | 1358.5 (956.3 to 1950) | 1400.2 (938.6 to 2045) | 1318.4 (884.4 to 1946) | 967.1 (668.1 to 1376) | 1114.4 (737.9 to 1586.3) | 829.7 (576.6 to 1203.4) | -28.8 (-44.4 to -6.5) | -20.4 (-39.5 to 6.8) | -37.1 (-54.6 to -13.9) |
|  |  | YLLs (Years of Life Lost) | Number | 67724.6 (56710.4 to 82685.3) | 31261 (25900.5 to 40207.4) | 36463.6 (30239 to 45223.6) | 6097.7 (5173.1 to 7036.5) | 2742.2 (2249.8 to 3189) | 3355.6 (2784.1 to 3899) | -91 (-93.4 to -88.5) | -91.2 (-94.1 to -88.8) | -90.8 (-93.6 to -88.2) |
|  |  |  | Rate | 16870.3 (14126.6 to 20597) | 15863.7 (13143.4 to 20403.6) | 17840.9 (14795.3 to 22126.9) | 1368.7 (1161.2 to 1579.4) | 1274.8 (1045.9 to 1482.5) | 1456.4 (1208.4 to 1692.3) | -91.9 (-94.1 to -89.7) | -92 (-94.6 to -89.7) | -91.8 (-94.3 to -89.5) |
|  | Injuries | DALYs (Disability-Adjusted Life Years) | Number | 24567.1 (21229.1 to 27980.3) | 9658.9 (8077.5 to 11524.1) | 14908.2 (12716.1 to 17356.2) | 6102.1 (5369.5 to 6882.9) | 1958 (1748.5 to 2159.9) | 4144 (3515.6 to 4794) | -75.2 (-79.5 to -69.5) | -79.7 (-83.9 to -75.2) | -72.2 (-77.9 to -65.2) |
|  |  |  | Rate | 6119.7 (5288.2 to 6969.9) | 4901.5 (4099 to 5848) | 7294.2 (6221.7 to 8492) | 1369.7 (1205.2 to 1544.9) | 910.2 (812.8 to 1004.1) | 1798.6 (1525.9 to 2080.7) | -77.6 (-81.6 to -72.6) | -81.4 (-85.2 to -77.3) | -75.3 (-80.4 to -69.2) |
|  |  | YLDs (Years Lived with Disability) | Number | 1339.3 (991.1 to 1778.6) | 626.9 (462.6 to 829.8) | 712.5 (523.4 to 945.4) | 627.8 (457.3 to 845.2) | 288.1 (211.1 to 387.3) | 339.7 (246.2 to 461.8) | -53.1 (-56.8 to -49.4) | -54 (-58.6 to -49.1) | -52.3 (-56 to -48.3) |
|  |  |  | Rate | 333.6 (246.9 to 443) | 318.1 (234.8 to 421.1) | 348.6 (256.1 to 462.5) | 140.9 (102.6 to 189.7) | 133.9 (98.1 to 180) | 147.4 (106.8 to 200.4) | -57.8 (-61 to -54.4) | -57.9 (-62.1 to -53.4) | -57.7 (-61 to -54.1) |
|  |  | YLLs (Years of Life Lost) | Number | 23227.7 (19994.5 to 26745.1) | 9032 (7462.7 to 10956.6) | 14195.7 (12028.9 to 16639.9) | 5474.3 (4807.5 to 6234.8) | 1669.9 (1480.3 to 1858.4) | 3804.4 (3204.5 to 4456.2) | -76.4 (-80.9 to -70.6) | -81.5 (-85.8 to -76.8) | -73.2 (-79.1 to -66) |
|  |  |  | Rate | 5786.1 (4980.7 to 6662.2) | 4583.4 (3787 to 5560) | 6945.6 (5885.5 to 8141.6) | 1228.8 (1079.1 to 1399.5) | 776.3 (688.2 to 863.9) | 1651.2 (1390.8 to 1934.1) | -78.8 (-82.8 to -73.5) | -83.1 (-87 to -78.8) | -76.2 (-81.5 to -69.9) |
|  | Non-communicable diseases | DALYs (Disability-Adjusted Life Years) | Number | 53140.3 (40757.2 to 62539.1) | 26319.3 (18068.3 to 31613) | 26821 (19438.1 to 32418.2) | 24063.7 (18739.8 to 30092.6) | 12263.3 (9484.6 to 15627.7) | 11800.4 (9312.8 to 14630.6) | -54.7 (-64.5 to -37) | -53.4 (-64.4 to -31.2) | -56 (-66.1 to -35) |
|  |  |  | Rate | 13237.3 (10152.7 to 15578.6) | 13356 (9168.9 to 16042.3) | 13122.9 (9510.6 to 15861.5) | 5401.3 (4206.3 to 6754.6) | 5700.8 (4409.1 to 7264.9) | 5121.7 (4042 to 6350) | -59.2 (-68 to -43.2) | -57.3 (-67.4 to -36.9) | -61 (-69.9 to -42.3) |
|  |  | YLDs (Years Lived with Disability) | Number | 14637.8 (10572.1 to 19787.9) | 7656.5 (5495.4 to 10407.9) | 6981.3 (5054.7 to 9422.4) | 18126.8 (13077.3 to 24190.7) | 9499.2 (6803.4 to 12776.6) | 8627.6 (6228.2 to 11518.2) | 23.8 (14.7 to 34.1) | 24.1 (15.1 to 34.5) | 23.6 (13 to 35.1) |
|  |  |  | Rate | 3646.3 (2633.5 to 4929.2) | 3885.3 (2788.7 to 5281.6) | 3415.8 (2473.2 to 4610.2) | 4068.7 (2935.3 to 5429.8) | 4415.9 (3162.7 to 5939.5) | 3744.6 (2703.2 to 4999.2) | 11.6 (3.4 to 20.8) | 13.7 (5.4 to 23.2) | 9.6 (0.3 to 19.9) |
|  |  | YLLs (Years of Life Lost) | Number | 38502.5 (25576.2 to 46333.4) | 18662.9 (10627.5 to 22924.9) | 19839.7 (12533.6 to 24691.5) | 5936.8 (5067.8 to 7025.5) | 2764.1 (2341.2 to 3209) | 3172.8 (2634 to 4020.1) | -84.6 (-88.2 to -74.7) | -85.2 (-88.8 to -71.5) | -84 (-88.2 to -70.9) |
|  |  |  | Rate | 9591 (6371.1 to 11541.7) | 9470.6 (5393 to 11633.4) | 9707.1 (6132.4 to 12081) | 1332.6 (1137.5 to 1576.9) | 1284.9 (1088.4 to 1491.8) | 1377.1 (1143.2 to 1744.8) | -86.1 (-89.4 to -77.2) | -86.4 (-89.7 to -73.9) | -85.8 (-89.5 to -74.2) |
| Chahar Mahaal and Bakhtiari | All causes | DALYs (Disability-Adjusted Life Years) | Number | 174863.6 (155771.2 to 195222.3) | 78943.7 (69956.1 to 88438.5) | 95919.9 (85132.5 to 107585.7) | 26352.1 (21547.1 to 31965.8) | 12657.7 (10117.2 to 15754.5) | 13694.4 (11317.1 to 16359.2) | -84.9 (-87.9 to -81.8) | -84 (-87.3 to -80.4) | -85.7 (-88.5 to -82.9) |
|  |  |  | Rate | 40445.5 (36029.5 to 45154.4) | 37192.8 (32958.4 to 41666.1) | 43582.4 (38681 to 48882.9) | 7789 (6368.8 to 9448.3) | 7672.9 (6132.9 to 9550.1) | 7899.5 (6528.2 to 9436.7) | -80.7 (-84.5 to -76.7) | -79.4 (-83.7 to -74.8) | -81.9 (-85.4 to -78.3) |
|  |  | YLDs (Years Lived with Disability) | Number | 22553.1 (16530.4 to 30144.5) | 11601.6 (8402.4 to 15550) | 10951.5 (7967 to 14631.7) | 16367.5 (11842.1 to 21964.6) | 8745.5 (6306.9 to 11795.7) | 7622.1 (5550.2 to 10257.4) | -27.4 (-33.6 to -20.7) | -24.6 (-31.2 to -17) | -30.4 (-38.7 to -22.2) |
|  |  |  | Rate | 5216.5 (3823.4 to 6972.3) | 5465.9 (3958.6 to 7326.1) | 4976 (3619.9 to 6648.1) | 4837.8 (3500.2 to 6492.2) | 5301.3 (3823.1 to 7150.4) | 4396.7 (3201.6 to 5916.9) | -7.3 (-15.2 to 1.3) | -3 (-11.5 to 6.8) | -11.6 (-22.2 to -1.2) |
|  |  | YLLs (Years of Life Lost) | Number | 152310.5 (133193.3 to 170222.5) | 67342.1 (58960.9 to 76004.8) | 84968.4 (74262.3 to 94908.8) | 9984.6 (8808.4 to 11170.5) | 3912.3 (3450.6 to 4409) | 6072.3 (5292.3 to 6843.8) | -93.4 (-94.6 to -92.2) | -94.2 (-95.2 to -93) | -92.9 (-94.1 to -91.4) |
|  |  |  | Rate | 35229 (30807.2 to 39372) | 31726.9 (27778.3 to 35808.2) | 38606.5 (33742 to 43123) | 2951.2 (2603.5 to 3301.7) | 2371.5 (2091.7 to 2672.6) | 3502.8 (3052.8 to 3947.8) | -91.6 (-93 to -90) | -92.5 (-93.9 to -91) | -90.9 (-92.5 to -89.1) |
|  | Communicable, maternal, neonatal, and nutritional diseases | DALYs (Disability-Adjusted Life Years) | Number | 78424.6 (66022 to 96570.5) | 35502.6 (29088.8 to 45620.6) | 42922 (36285.5 to 53660.6) | 5980.3 (4843.9 to 7295.5) | 3045.7 (2370.3 to 3915) | 2934.6 (2348.6 to 3571.4) | -92.4 (-94.5 to -90.2) | -91.4 (-94.3 to -88.5) | -93.2 (-95.2 to -91.1) |
|  |  |  | Rate | 18139.4 (15270.7 to 22336.5) | 16726.3 (13704.6 to 21493.2) | 19502.2 (16486.7 to 24381.4) | 1767.6 (1431.7 to 2156.4) | 1846.2 (1436.8 to 2373.2) | 1692.8 (1354.8 to 2060.2) | -90.3 (-92.9 to -87.5) | -89 (-92.7 to -85.2) | -91.3 (-93.8 to -88.7) |
|  |  | YLDs (Years Lived with Disability) | Number | 5525.8 (3734.3 to 7848) | 2885.2 (1884.2 to 4234.7) | 2640.6 (1651.4 to 3996.5) | 3084.3 (2094.8 to 4345.9) | 1752.2 (1150.3 to 2553.8) | 1332.2 (893.8 to 1965.8) | -44.2 (-55.4 to -27.1) | -39.3 (-54.3 to -17.5) | -49.6 (-64.8 to -27.2) |
|  |  |  | Rate | 1278.1 (863.7 to 1815.2) | 1359.3 (887.7 to 1995.1) | 1199.8 (750.3 to 1815.9) | 911.6 (619.2 to 1284.5) | 1062.1 (697.3 to 1548.1) | 768.4 (515.6 to 1133.9) | -28.7 (-43 to -6.8) | -21.9 (-41.2 to 6.2) | -36 (-55.3 to -7.6) |
|  |  | YLLs (Years of Life Lost) | Number | 72898.8 (60672 to 90594.2) | 32617.4 (26206.8 to 42901.4) | 40281.4 (33256.5 to 50746.1) | 2896 (2329.1 to 3504.7) | 1293.5 (1010.9 to 1584.2) | 1602.4 (1249.1 to 1946.8) | -96 (-97.3 to -94.8) | -96 (-97.5 to -94.6) | -96 (-97.4 to -94.7) |
|  |  |  | Rate | 16861.3 (14033.3 to 20954.2) | 15367 (12346.8 to 20212.2) | 18302.4 (15110.5 to 23057.1) | 856 (688.4 to 1035.9) | 784.1 (612.8 to 960.3) | 924.4 (720.6 to 1123) | -94.9 (-96.5 to -93.3) | -94.9 (-96.8 to -93.1) | -94.9 (-96.7 to -93.2) |
|  | Injuries | DALYs (Disability-Adjusted Life Years) | Number | 32496.2 (27942 to 38293.5) | 12341.8 (10269.2 to 15255.8) | 20154.4 (17247.3 to 24499.5) | 3878.3 (3417.3 to 4369.6) | 1184.6 (1039.4 to 1328.9) | 2693.8 (2305.1 to 3095.6) | -88.1 (-90.4 to -85.4) | -90.4 (-92.7 to -88.1) | -86.6 (-89.6 to -83.4) |
|  |  |  | Rate | 7516.3 (6462.9 to 8857.2) | 5814.6 (4838.1 to 7187.5) | 9157.4 (7836.5 to 11131.7) | 1146.3 (1010.1 to 1291.6) | 718.1 (630 to 805.6) | 1553.9 (1329.7 to 1785.7) | -84.7 (-87.7 to -81.3) | -87.7 (-90.6 to -84.7) | -83 (-86.8 to -78.9) |
|  |  | YLDs (Years Lived with Disability) | Number | 1621.9 (1197.2 to 2144.8) | 702.4 (515.4 to 929.2) | 919.5 (679.7 to 1225.5) | 452.5 (331.2 to 615.2) | 198.1 (144.2 to 266.9) | 254.4 (185.6 to 349.7) | -72.1 (-74.6 to -69.6) | -71.8 (-74.6 to -68.7) | -72.3 (-75 to -69.8) |
|  |  |  | Rate | 375.1 (276.9 to 496.1) | 330.9 (242.8 to 437.8) | 417.8 (308.8 to 556.8) | 133.8 (97.9 to 181.8) | 120.1 (87.4 to 161.8) | 146.8 (107.1 to 201.7) | -64.3 (-67.5 to -61.2) | -63.7 (-67.3 to -59.8) | -64.9 (-68.2 to -61.6) |
|  |  | YLLs (Years of Life Lost) | Number | 30874.3 (26345.8 to 36524) | 11639.4 (9642.1 to 14497) | 19234.9 (16346.7 to 23548.1) | 3425.8 (2987.3 to 3909) | 986.5 (850.9 to 1125) | 2439.3 (2063.3 to 2844) | -88.9 (-91.1 to -86.1) | -91.5 (-93.7 to -89.2) | -87.3 (-90.4 to -84) |
|  |  |  | Rate | 7141.1 (6093.7 to 8447.9) | 5483.7 (4542.7 to 6830) | 8739.6 (7427.3 to 10699.4) | 1012.6 (883 to 1155.4) | 598 (515.8 to 682) | 1407.1 (1190.2 to 1640.5) | -85.8 (-88.7 to -82.2) | -89.1 (-91.9 to -86.1) | -83.9 (-87.8 to -79.7) |
|  | Non-communicable diseases | DALYs (Disability-Adjusted Life Years) | Number | 63942.8 (47623.4 to 75980.4) | 31099.3 (21198 to 37378.7) | 32843.5 (23170.8 to 40179.8) | 16386.1 (12659.1 to 21065.5) | 8394.3 (6398.1 to 10920.4) | 7991.8 (6237.5 to 10155.5) | -74.4 (-80.6 to -63.5) | -73 (-79.9 to -58) | -75.7 (-81.7 to -62.1) |
|  |  |  | Rate | 14789.8 (11015.2 to 17574.1) | 14651.8 (9987 to 17610.2) | 14922.8 (10527.9 to 18256.2) | 4843.3 (3741.7 to 6226.4) | 5088.5 (3878.4 to 6619.8) | 4610 (3598.1 to 5858.1) | -67.3 (-75.2 to -53.3) | -65.3 (-74.2 to -46) | -69.1 (-76.7 to -51.9) |
|  |  | YLDs (Years Lived with Disability) | Number | 15405.4 (11111 to 20673) | 8014 (5752.6 to 10768) | 7391.4 (5342.8 to 9924.5) | 12830.7 (9342.9 to 17292.8) | 6795.2 (4897.7 to 9257.8) | 6035.4 (4398.8 to 8066.9) | -16.7 (-23.1 to -10.2) | -15.2 (-22 to -8.3) | -18.3 (-24.9 to -11) |
|  |  |  | Rate | 3563.2 (2570 to 4781.6) | 3775.7 (2710.2 to 5073.2) | 3358.4 (2427.6 to 4509.3) | 3792.4 (2761.5 to 5111.3) | 4119.1 (2968.9 to 5611.9) | 3481.5 (2537.4 to 4653.4) | 6.4 (-1.7 to 14.8) | 9.1 (0.4 to 17.9) | 3.7 (-4.7 to 13) |
|  |  | YLLs (Years of Life Lost) | Number | 48537.4 (32343.3 to 58579.1) | 23085.3 (13182.8 to 28540.1) | 25452.1 (15721.1 to 31891.8) | 3555.4 (2917.8 to 4282) | 1599.1 (1306.9 to 1952.2) | 1956.4 (1546.1 to 2516.2) | -92.7 (-94.5 to -87.4) | -93.1 (-94.7 to -86.4) | -92.3 (-94.6 to -85.4) |
|  |  |  | Rate | 11226.6 (7480.9 to 13549.2) | 10876.2 (6210.8 to 13446.1) | 11564.5 (7143.1 to 14490.5) | 1050.9 (862.4 to 1265.6) | 969.3 (792.2 to 1183.4) | 1128.5 (891.9 to 1451.4) | -90.6 (-93 to -83.9) | -91.1 (-93.2 to -82.5) | -90.2 (-93.1 to -81.4) |
| East Azarbayejan | All causes | DALYs (Disability-Adjusted Life Years) | Number | 936707.3 (841146.1 to 1043365.6) | 415240.5 (372493 to 463765.7) | 521466.8 (467002.6 to 580654.9) | 113929.6 (96554.4 to 134491.1) | 53284.7 (43527.6 to 64515.5) | 60644.8 (52057 to 70635.6) | -87.8 (-89.9 to -85.6) | -87.2 (-89.4 to -84.7) | -88.4 (-90.4 to -86.3) |
|  |  |  | Rate | 49309.3 (44278.9 to 54924) | 44483.2 (39903.8 to 49681.5) | 53972.1 (48335.1 to 60098.1) | 9332.5 (7909.2 to 11016.8) | 9027 (7374 to 10929.6) | 9618.4 (8256.4 to 11203) | -81.1 (-84.4 to -77.6) | -79.7 (-83.3 to -75.8) | -82.2 (-85.3 to -79) |
|  |  | YLDs (Years Lived with Disability) | Number | 103217.5 (74961 to 138148.3) | 51565.7 (37445.2 to 68892.2) | 51651.8 (37311.7 to 69542.4) | 60971.5 (43983.3 to 80066.6) | 31724.7 (22912.5 to 42145.9) | 29246.8 (21035.4 to 38795.1) | -40.9 (-46.3 to -35.2) | -38.5 (-44.2 to -31.9) | -43.4 (-50.3 to -36.9) |
|  |  |  | Rate | 5433.5 (3946 to 7272.3) | 5524 (4011.4 to 7380.2) | 5346 (3861.8 to 7197.7) | 4994.4 (3602.9 to 6558.6) | 5374.5 (3881.6 to 7140) | 4638.6 (3336.3 to 6153) | -8.1 (-16.4 to 0.8) | -2.7 (-11.7 to 7.8) | -13.2 (-23.8 to -3.4) |
|  |  | YLLs (Years of Life Lost) | Number | 833489.8 (741067.5 to 931325.7) | 363674.8 (321724.5 to 406225.3) | 469815 (416151.5 to 525798.4) | 52958 (48517.7 to 57614.3) | 21560 (19501 to 23687.8) | 31398 (28145 to 34552.3) | -93.6 (-94.5 to -92.6) | -94.1 (-94.9 to -93.1) | -93.3 (-94.3 to -92.1) |
|  |  |  | Rate | 43875.9 (39010.6 to 49026.1) | 38959.2 (34465.2 to 43517.4) | 48626.1 (43071.9 to 54420.5) | 4338 (3974.3 to 4719.4) | 3652.5 (3303.7 to 4013) | 4979.8 (4463.9 to 5480.1) | -90.1 (-91.5 to -88.6) | -90.6 (-92 to -89) | -89.8 (-91.3 to -88) |
|  | Communicable, maternal, neonatal, and nutritional diseases | DALYs (Disability-Adjusted Life Years) | Number | 429730 (358864.8 to 534760.9) | 193737.8 (160446.7 to 253937.9) | 235992.2 (195674.9 to 295468.3) | 24316.3 (19945.9 to 29573.7) | 12076.3 (9770.3 to 15059.4) | 12240 (9810.1 to 15318) | -94.3 (-95.9 to -92.7) | -93.8 (-95.7 to -91.7) | -94.8 (-96.3 to -93.2) |
|  |  |  | Rate | 22621.5 (18891.1 to 28150.4) | 20754.4 (17188.1 to 27203.5) | 24425.3 (20252.5 to 30581.2) | 1991.9 (1633.9 to 2422.5) | 2045.8 (1655.2 to 2551.2) | 1941.3 (1555.9 to 2429.5) | -91.2 (-93.6 to -88.6) | -90.1 (-93.2 to -86.9) | -92.1 (-94.3 to -89.6) |
|  |  | YLDs (Years Lived with Disability) | Number | 25221.6 (16802.7 to 35978) | 11988.8 (7565.8 to 17668.9) | 13232.8 (8336.9 to 19810.1) | 11139.6 (7544 to 15884.5) | 5996.3 (4045.2 to 8951.8) | 5143.2 (3292.7 to 7553.4) | -55.8 (-65.8 to -41.3) | -50 (-62.6 to -32.7) | -61.1 (-72.3 to -44) |
|  |  |  | Rate | 1327.7 (884.5 to 1893.9) | 1284.3 (810.5 to 1892.8) | 1369.6 (862.9 to 2050.4) | 912.5 (618 to 1301.2) | 1015.8 (685.3 to 1516.5) | 815.7 (522.2 to 1198) | -31.3 (-46.8 to -8.7) | -20.9 (-40.8 to 6.4) | -40.4 (-57.5 to -14.1) |
|  |  | YLLs (Years of Life Lost) | Number | 404508.3 (333131.3 to 508741.2) | 181749 (148953.7 to 240257.1) | 222759.4 (182165.4 to 281666.6) | 13176.7 (10746.6 to 15797) | 6079.9 (4918.8 to 7519.7) | 7096.8 (5584.2 to 8451.4) | -96.7 (-97.8 to -95.7) | -96.7 (-97.9 to -95.6) | -96.8 (-97.8 to -95.8) |
|  |  |  | Rate | 21293.8 (17536.4 to 26780.7) | 19470.1 (15956.9 to 25737.9) | 23055.7 (18854.2 to 29152.7) | 1079.4 (880.3 to 1294) | 1030 (833.3 to 1273.9) | 1125.6 (885.7 to 1340.4) | -94.9 (-96.5 to -93.4) | -94.7 (-96.6 to -93) | -95.1 (-96.7 to -93.5) |
|  | Injuries | DALYs (Disability-Adjusted Life Years) | Number | 165601.3 (141640.8 to 195811.1) | 60503.5 (49453.3 to 76145.3) | 105097.8 (89178.2 to 123771.7) | 20275.6 (17799.3 to 22928.5) | 6360.8 (5597.9 to 7421.8) | 13914.9 (12034.9 to 16118.3) | -87.8 (-90.1 to -85) | -89.5 (-92 to -86.6) | -86.8 (-89.7 to -83.6) |
|  |  |  | Rate | 8717.4 (7456.1 to 10307.7) | 6481.5 (5297.8 to 8157.2) | 10877.7 (9230 to 12810.4) | 1660.9 (1458 to 1878.2) | 1077.6 (948.3 to 1257.3) | 2206.9 (1908.8 to 2556.4) | -80.9 (-84.6 to -76.7) | -83.4 (-87.4 to -78.8) | -79.7 (-84.2 to -74.9) |
|  |  | YLDs (Years Lived with Disability) | Number | 7147.2 (5283.5 to 9402.6) | 3229 (2380.2 to 4342.8) | 3918.2 (2906 to 5134.6) | 1812.7 (1318.2 to 2403.3) | 806.6 (592.9 to 1095.4) | 1006.1 (724.6 to 1345.2) | -74.6 (-76.9 to -72.4) | -75 (-77.7 to -72.2) | -74.3 (-76.8 to -72.1) |
|  |  |  | Rate | 376.2 (278.1 to 495) | 345.9 (255 to 465.2) | 405.5 (300.8 to 531.4) | 148.5 (108 to 196.9) | 136.7 (100.4 to 185.6) | 159.6 (114.9 to 213.4) | -60.5 (-64 to -57.1) | -60.5 (-64.7 to -56.1) | -60.7 (-64.4 to -57.3) |
|  |  | YLLs (Years of Life Lost) | Number | 158454.2 (133903.2 to 187672.7) | 57274.6 (46412.9 to 73008.7) | 101179.6 (85409.2 to 120028.2) | 18462.9 (16196.7 to 21105.8) | 5554.1 (4843.3 to 6585.8) | 12908.8 (11048.4 to 15153.8) | -88.3 (-90.7 to -85.5) | -90.3 (-92.7 to -87.4) | -87.2 (-90.2 to -84) |
|  |  |  | Rate | 8341.2 (7048.8 to 9879.3) | 6135.6 (4972.1 to 7821.2) | 10472.2 (8839.9 to 12423) | 1512.4 (1326.7 to 1728.9) | 940.9 (820.5 to 1115.7) | 2047.4 (1752.3 to 2403.4) | -81.9 (-85.6 to -77.5) | -84.7 (-88.4 to -80.1) | -80.4 (-85 to -75.5) |
|  | Non-communicable diseases | DALYs (Disability-Adjusted Life Years) | Number | 341376.1 (249483.1 to 404994.6) | 160999.2 (102499.8 to 192830) | 180376.9 (125731.3 to 218683.4) | 68802.6 (55443 to 84930.4) | 34689.5 (27279.6 to 43332.7) | 34113.1 (27460 to 42089.6) | -79.8 (-84.4 to -70.6) | -78.5 (-83.3 to -64.7) | -81.1 (-85.6 to -71.2) |
|  |  |  | Rate | 17970.4 (13133.1 to 21319.4) | 17247.3 (10980.4 to 20657.2) | 18669.1 (13013.3 to 22633.9) | 5635.9 (4541.6 to 6957) | 5876.8 (4621.5 to 7341) | 5410.4 (4355.2 to 6675.5) | -68.6 (-75.7 to -54.3) | -65.9 (-73.6 to -44.1) | -71 (-77.9 to -55.9) |
|  |  | YLDs (Years Lived with Disability) | Number | 70848.8 (51058.5 to 95280.2) | 36347.9 (26148.9 to 49115.9) | 34500.8 (24960.7 to 46246.5) | 48019.2 (34293.9 to 64062.3) | 24921.8 (17924 to 33278.7) | 23097.5 (16505.1 to 30652.6) | -32.2 (-38.8 to -25.9) | -31.4 (-38.1 to -24.6) | -33.1 (-40.5 to -26) |
|  |  |  | Rate | 3729.6 (2687.8 to 5015.7) | 3893.8 (2801.2 to 5261.6) | 3570.9 (2583.5 to 4786.5) | 3933.5 (2809.2 to 5247.6) | 4222 (3036.5 to 5637.8) | 3663.3 (2617.8 to 4861.6) | 5.5 (-4.8 to 15.2) | 8.4 (-2.1 to 19.2) | 2.6 (-8.9 to 13.4) |
|  |  | YLLs (Years of Life Lost) | Number | 270527.3 (180234.6 to 330140.6) | 124651.3 (67322.8 to 152805.3) | 145876 (91213.9 to 181479.6) | 20783.4 (17406.5 to 24328.4) | 9767.7 (8084.4 to 11382.4) | 11015.6 (8997 to 13435.2) | -92.3 (-94 to -87.1) | -92.2 (-93.9 to -84.3) | -92.4 (-94.3 to -86.2) |
|  |  |  | Rate | 14240.9 (9487.8 to 17379) | 13353.4 (7212 to 16369.5) | 15098.3 (9440.7 to 18783.3) | 1702.5 (1425.8 to 1992.8) | 1654.8 (1369.6 to 1928.3) | 1747.1 (1426.9 to 2130.9) | -88 (-90.7 to -79.9) | -87.6 (-90.4 to -75.2) | -88.4 (-91.3 to -78.9) |
| Fars | All causes | DALYs (Disability-Adjusted Life Years) | Number | 704911.3 (637797.5 to 783708.9) | 306396.1 (277049.7 to 341415.2) | 398515.2 (357661.3 to 444427.1) | 158624.1 (135154.1 to 185282.2) | 70780.4 (58429.5 to 84880) | 87843.7 (75723.6 to 100978.8) | -77.5 (-80.8 to -73.9) | -76.9 (-80.6 to -73) | -78 (-81.2 to -74.4) |
|  |  |  | Rate | 34515.4 (31229.2 to 38373.6) | 30621.3 (27688.4 to 34121.1) | 38255.7 (34333.9 to 42663.1) | 11120.2 (9474.8 to 12989) | 10206.5 (8425.5 to 12239.7) | 11984.6 (10331 to 13776.6) | -67.8 (-72.5 to -62.6) | -66.7 (-72 to -61) | -68.7 (-73.3 to -63.6) |
|  |  | YLDs (Years Lived with Disability) | Number | 114452.2 (84457.9 to 152767.6) | 58138.2 (42299.4 to 77962.1) | 56314 (41654 to 74934.7) | 76577.3 (55740 to 102547.2) | 40217.1 (29324.5 to 54277.5) | 36360.2 (26467.3 to 48773.7) | -33.1 (-38.8 to -27) | -30.8 (-36.5 to -24.1) | -35.4 (-42.7 to -28) |
|  |  |  | Rate | 5604 (4135.4 to 7480.1) | 5810.3 (4227.4 to 7791.5) | 5405.9 (3998.6 to 7193.4) | 5368.4 (3907.6 to 7189) | 5799.3 (4228.6 to 7826.8) | 4960.7 (3610.9 to 6654.2) | -4.2 (-12.4 to 4.5) | -0.2 (-8.4 to 9.5) | -8.2 (-18.6 to 2.3) |
|  |  | YLLs (Years of Life Lost) | Number | 590459.1 (526268.8 to 654991.5) | 248257.9 (221020.4 to 273288.9) | 342201.2 (302848.1 to 383550.6) | 82046.7 (73896.9 to 90437.4) | 30563.3 (27283.4 to 34122.3) | 51483.4 (45646.1 to 56800.5) | -86.1 (-88.1 to -83.8) | -87.7 (-89.6 to -85.6) | -85 (-87.2 to -82.3) |
|  |  |  | Rate | 28911.3 (25768.3 to 32071.1) | 24810.9 (22088.8 to 27312.5) | 32849.8 (29072.1 to 36819.2) | 5751.8 (5180.5 to 6340) | 4407.2 (3934.3 to 4920.4) | 7023.9 (6227.5 to 7749.3) | -80.1 (-82.9 to -76.8) | -82.2 (-85 to -79.3) | -78.6 (-81.8 to -74.9) |
|  | Communicable, maternal, neonatal, and nutritional diseases | DALYs (Disability-Adjusted Life Years) | Number | 304921.8 (262731.4 to 361702.2) | 138635.8 (118668.1 to 169786.3) | 166286 (140301.4 to 198552.5) | 35654.2 (29917.5 to 43187.7) | 17295.4 (14112.9 to 21360) | 18358.8 (15368 to 22171.2) | -88.3 (-90.8 to -85.3) | -87.5 (-90.7 to -84.2) | -89 (-91.5 to -86.2) |
|  |  |  | Rate | 14930.2 (12864.4 to 17710.4) | 13855.3 (11859.7 to 16968.5) | 15962.7 (13468.3 to 19060.2) | 2499.5 (2097.3 to 3027.6) | 2494 (2035.1 to 3080.1) | 2504.7 (2096.7 to 3024.8) | -83.3 (-86.9 to -79) | -82 (-86.6 to -77.2) | -84.3 (-87.9 to -80.4) |
|  |  | YLDs (Years Lived with Disability) | Number | 28609.5 (19591.4 to 40941.3) | 14517.3 (9808.9 to 21305) | 14092.2 (9213.1 to 21671.6) | 13715.2 (9483.4 to 19479.7) | 7580.7 (5057.3 to 11011.6) | 6134.6 (4145.5 to 8902.2) | -52.1 (-61.8 to -36.4) | -47.8 (-59.4 to -30.2) | -56.5 (-68.9 to -37.1) |
|  |  |  | Rate | 1400.8 (959.3 to 2004.7) | 1450.9 (980.3 to 2129.2) | 1352.8 (884.4 to 2080.4) | 961.5 (664.8 to 1365.6) | 1093.1 (729.3 to 1587.9) | 836.9 (565.6 to 1214.5) | -31.4 (-45.3 to -9) | -24.7 (-41.5 to 0.7) | -38.1 (-55.8 to -10.6) |
|  |  | YLLs (Years of Life Lost) | Number | 276312.2 (234730.8 to 328985.5) | 124118.5 (105541 to 153381.3) | 152193.8 (128092.1 to 184302.5) | 21939 (17957.8 to 26649.3) | 9714.8 (7739 to 11836) | 12224.2 (9966.5 to 14841.4) | -92.1 (-94.1 to -89.8) | -92.2 (-94.5 to -89.8) | -92 (-94 to -89.5) |
|  |  |  | Rate | 13529.4 (11493.4 to 16108.5) | 12404.4 (10547.8 to 15328.9) | 14609.9 (12296.3 to 17692.2) | 1538 (1258.9 to 1868.2) | 1400.9 (1116 to 1706.7) | 1667.8 (1359.7 to 2024.8) | -88.6 (-91.6 to -85.3) | -88.7 (-92.1 to -85.2) | -88.6 (-91.5 to -85.1) |
|  | Injuries | DALYs (Disability-Adjusted Life Years) | Number | 149645.7 (129176.2 to 168408.7) | 52007.2 (43312.1 to 60706.8) | 97638.4 (83764.2 to 110890.4) | 33987.4 (30573.9 to 37680.9) | 9740.6 (8731.8 to 10802.3) | 24246.9 (21151 to 27566.8) | -77.3 (-80.6 to -73.2) | -81.3 (-84.5 to -77.3) | -75.2 (-79.7 to -69.8) |
|  |  |  | Rate | 7327.3 (6325 to 8246) | 5197.6 (4328.6 to 6067) | 9372.9 (8041 to 10645) | 2382.7 (2143.4 to 2641.6) | 1404.6 (1259.1 to 1557.7) | 3308 (2885.6 to 3761) | -67.5 (-72.2 to -61.6) | -73 (-77.7 to -67.3) | -64.7 (-71.1 to -57.1) |
|  |  | YLDs (Years Lived with Disability) | Number | 8158.2 (6081 to 10792.9) | 3524.6 (2607.6 to 4635.3) | 4633.7 (3471.3 to 6065.1) | 2422.9 (1751 to 3235) | 1040.9 (762.3 to 1399.1) | 1382 (990.3 to 1857.8) | -70.3 (-73.1 to -67.7) | -70.5 (-73.2 to -67.6) | -70.2 (-73.4 to -67.4) |
|  |  |  | Rate | 399.5 (297.8 to 528.5) | 352.2 (260.6 to 463.3) | 444.8 (333.2 to 582.2) | 169.9 (122.8 to 226.8) | 150.1 (109.9 to 201.7) | 188.5 (135.1 to 253.5) | -57.5 (-61.5 to -53.8) | -57.4 (-61.4 to -53.2) | -57.6 (-62.2 to -53.7) |
|  |  | YLLs (Years of Life Lost) | Number | 141487.4 (121605.1 to 159370.4) | 48482.6 (39847.7 to 57228.4) | 93004.8 (79328.6 to 106339.7) | 31564.6 (28095.2 to 35130) | 8699.7 (7706.2 to 9703.3) | 22864.9 (19688.1 to 26179.6) | -77.7 (-81.2 to -73.4) | -82.1 (-85.3 to -77.8) | -75.4 (-80.1 to -69.9) |
|  |  |  | Rate | 6927.8 (5954.3 to 7803.4) | 4845.4 (3982.4 to 5719.4) | 8928.1 (7615.2 to 10208.1) | 2212.8 (1969.6 to 2462.7) | 1254.5 (1111.2 to 1399.2) | 3119.5 (2686.1 to 3571.7) | -68.1 (-73 to -61.9) | -74.1 (-78.8 to -68) | -65.1 (-71.7 to -57.3) |
|  | Non-communicable diseases | DALYs (Disability-Adjusted Life Years) | Number | 250343.8 (205329.3 to 292584.1) | 115753.1 (86357.8 to 138337.1) | 134590.8 (106880.7 to 157900.3) | 88322.1 (71191.7 to 110241) | 43541.7 (34350 to 55111.3) | 44780.4 (36313.9 to 55262.6) | -64.7 (-71.2 to -54) | -62.4 (-69.9 to -48.7) | -66.7 (-73.1 to -55.4) |
|  |  |  | Rate | 12257.9 (10053.8 to 14326.1) | 11568.4 (8630.6 to 13825.4) | 12920.1 (10260.1 to 15157.7) | 6191.7 (4990.8 to 7728.3) | 6278.7 (4953.3 to 7947) | 6109.4 (4954.3 to 7539.5) | -49.5 (-58.8 to -34.2) | -45.7 (-56.6 to -26) | -52.7 (-61.7 to -36.6) |
|  |  | YLDs (Years Lived with Disability) | Number | 77684.4 (56273 to 103719.4) | 40096.3 (28806.3 to 53220.1) | 37588.2 (27529.2 to 49784.3) | 60439.2 (42975 to 81788.1) | 31595.5 (22308.5 to 42880.6) | 28843.7 (20572.1 to 38708.2) | -22.2 (-28.3 to -15.8) | -21.2 (-27.1 to -14.5) | -23.3 (-30.2 to -16.1) |
|  |  |  | Rate | 3803.7 (2755.4 to 5078.5) | 4007.2 (2878.9 to 5318.8) | 3608.3 (2642.7 to 4779.1) | 4237 (3012.7 to 5733.7) | 4556.1 (3216.9 to 6183.4) | 3935.2 (2806.7 to 5281) | 11.4 (2.6 to 20.6) | 13.7 (5.2 to 23.4) | 9.1 (-0.7 to 19.2) |
|  |  | YLLs (Years of Life Lost) | Number | 172659.4 (129957.8 to 206704.3) | 75656.8 (48516.2 to 93985.6) | 97002.6 (71086.6 to 118224.7) | 27882.8 (24004.7 to 32698.3) | 11946.1 (10163.3 to 14061.9) | 15936.7 (13270.8 to 19185.1) | -83.9 (-87.3 to -76.4) | -84.2 (-87.8 to -72.8) | -83.6 (-87.3 to -74.6) |
|  |  |  | Rate | 8454.1 (6363.3 to 10121.1) | 7561.1 (4848.7 to 9392.9) | 9311.8 (6824 to 11349.1) | 1954.7 (1682.8 to 2292.3) | 1722.6 (1465.6 to 2027.7) | 2174.3 (1810.5 to 2617.4) | -76.9 (-81.8 to -66.2) | -77.2 (-82.4 to -60.7) | -76.7 (-82 to -63.9) |
| Gilan | All causes | DALYs (Disability-Adjusted Life Years) | Number | 1854922.4 (1712625.6 to 2009876.1) | 1032323.3 (951659.2 to 1120636.4) | 822599.2 (760032.6 to 891236.4) | 51453.5 (42243.5 to 62315.1) | 23125.5 (18168.1 to 28704.4) | 28328 (23456.6 to 33556.7) | -97.2 (-97.8 to -96.6) | -97.8 (-98.3 to -97.2) | -96.6 (-97.2 to -95.9) |
|  |  |  | Rate | 158538.9 (146376.9 to 171782.7) | 179076 (165083.3 to 194395.6) | 138592.3 (128051.1 to 150156.4) | 8373.3 (6874.5 to 10140.9) | 7689.8 (6041.3 to 9544.9) | 9028.5 (7475.9 to 10695) | -94.7 (-95.8 to -93.6) | -95.7 (-96.7 to -94.6) | -93.5 (-94.6 to -92.2) |
|  |  | YLDs (Years Lived with Disability) | Number | 67807.3 (50539.8 to 90234.6) | 35717 (26478.2 to 47185.8) | 32090.3 (23872.2 to 43507.3) | 31965 (23271.5 to 42607.4) | 17074.2 (12221.3 to 22706.8) | 14890.8 (10795.5 to 20063.1) | -52.9 (-56.5 to -48.7) | -52.2 (-56.2 to -48) | -53.6 (-57.9 to -48.5) |
|  |  |  | Rate | 5795.4 (4319.6 to 7712.3) | 6195.8 (4593.1 to 8185.3) | 5406.6 (4022 to 7330.2) | 5201.9 (3787.1 to 6933.7) | 5677.6 (4063.9 to 7550.5) | 4745.9 (3440.7 to 6394.4) | -10.2 (-17.2 to -2.4) | -8.4 (-16 to -0.2) | -12.2 (-20.3 to -2.6) |
|  |  | YLLs (Years of Life Lost) | Number | 1787115.1 (1648075.4 to 1943242.1) | 996606.3 (916397.6 to 1084480.3) | 790508.8 (728215 to 861619.7) | 19488.5 (17318.8 to 21569) | 6051.3 (5306.6 to 6851.1) | 13437.2 (11799.8 to 15182.7) | -98.9 (-99.1 to -98.8) | -99.4 (-99.5 to -99.3) | -98.3 (-98.6 to -98) |
|  |  |  | Rate | 152743.5 (140859.8 to 166087.5) | 172880.2 (158966.5 to 188123.6) | 133185.7 (122690.4 to 145166.6) | 3171.5 (2818.4 to 3510.1) | 2012.2 (1764.6 to 2278.2) | 4282.6 (3760.7 to 4838.9) | -97.9 (-98.2 to -97.6) | -98.8 (-99 to -98.7) | -96.8 (-97.3 to -96.3) |
|  | Communicable, maternal, neonatal, and nutritional diseases | DALYs (Disability-Adjusted Life Years) | Number | 137148.2 (105855.9 to 182481.8) | 62222.8 (48124.7 to 82999.7) | 74925.4 (56616.1 to 101618.8) | 9142.3 (7232.5 to 11469.6) | 4626.7 (3561.6 to 6108.7) | 4515.6 (3588 to 5771.3) | -93.3 (-95.4 to -90.7) | -92.6 (-95 to -89.4) | -94 (-95.9 to -91.4) |
|  |  |  | Rate | 11722 (9047.4 to 15596.6) | 10793.7 (8348.1 to 14397.9) | 12623.5 (9538.7 to 17120.8) | 1487.8 (1177 to 1866.5) | 1538.5 (1184.3 to 2031.3) | 1439.2 (1143.5 to 1839.4) | -87.3 (-91.3 to -82.2) | -85.7 (-90.4 to -79.8) | -88.6 (-92.3 to -83.7) |
|  |  | YLDs (Years Lived with Disability) | Number | 13389.4 (9090 to 19795.7) | 6619.8 (4268.5 to 9926.6) | 6769.5 (4314 to 10229.2) | 5410.2 (3682.5 to 7721.8) | 2954.4 (1970 to 4217.7) | 2455.8 (1651.6 to 3556.1) | -59.6 (-68.3 to -46.3) | -55.4 (-66.5 to -38.7) | -63.7 (-73.9 to -49.8) |
|  |  |  | Rate | 1144.4 (776.9 to 1691.9) | 1148.3 (740.4 to 1722) | 1140.5 (726.8 to 1723.4) | 880.4 (599.3 to 1256.6) | 982.4 (655.1 to 1402.5) | 782.7 (526.4 to 1133.4) | -23.1 (-39.7 to 2.3) | -14.4 (-35.8 to 17.6) | -31.4 (-50.7 to -4.9) |
|  |  | YLLs (Years of Life Lost) | Number | 123758.8 (92187.5 to 168611.1) | 55603 (41262.3 to 75703.4) | 68155.8 (50132.8 to 93897) | 3732.2 (3024.3 to 4534.2) | 1672.3 (1310.6 to 2064.2) | 2059.8 (1639.9 to 2490.9) | -97 (-98.1 to -95.6) | -97 (-98.1 to -95.5) | -97 (-98.1 to -95.5) |
|  |  |  | Rate | 10577.6 (7879.2 to 14411.1) | 9645.4 (7157.7 to 13132.2) | 11483 (8446.4 to 15819.9) | 607.4 (492.2 to 737.9) | 556.1 (435.8 to 686.4) | 656.5 (522.7 to 793.9) | -94.3 (-96.3 to -91.6) | -94.2 (-96.4 to -91.4) | -94.3 (-96.3 to -91.5) |
|  | Injuries | DALYs (Disability-Adjusted Life Years) | Number | 1592703.2 (1453528.3 to 1749619.6) | 910731.4 (829486.1 to 999946.2) | 681971.8 (623673.4 to 746872.2) | 10189.6 (8925.4 to 11428.7) | 2230.4 (1972.7 to 2515) | 7959.2 (6843.7 to 9189.5) | -99.4 (-99.5 to -99.3) | -99.8 (-99.8 to -99.7) | -98.8 (-99 to -98.6) |
|  |  |  | Rate | 136127.2 (124232 to 149538.7) | 157983.6 (143890 to 173459.6) | 114899.3 (105077.1 to 125833.8) | 1658.2 (1452.5 to 1859.9) | 741.6 (656 to 836.3) | 2536.7 (2181.2 to 2928.8) | -98.8 (-99 to -98.6) | -99.5 (-99.6 to -99.5) | -97.8 (-98.2 to -97.4) |
|  |  | YLDs (Years Lived with Disability) | Number | 10231.6 (7510 to 13731) | 5808.5 (4226.6 to 7973.7) | 4423.1 (3249.9 to 5884) | 903.1 (656.9 to 1221.5) | 386.3 (283.3 to 516.8) | 516.8 (373.1 to 715.3) | -91.2 (-92.8 to -89.4) | -93.3 (-94.8 to -91.7) | -88.3 (-90.2 to -86.4) |
|  |  |  | Rate | 874.5 (641.9 to 1173.6) | 1007.6 (733.2 to 1383.2) | 745.2 (547.5 to 991.3) | 147 (106.9 to 198.8) | 128.5 (94.2 to 171.8) | 164.7 (118.9 to 228) | -83.2 (-86.3 to -79.9) | -87.3 (-90 to -84) | -77.9 (-81.5 to -74.2) |
|  |  | YLLs (Years of Life Lost) | Number | 1582471.6 (1442525.6 to 1739826.8) | 904922.9 (824461.6 to 994253.3) | 677548.7 (618981.6 to 743191.4) | 9286.5 (8116.9 to 10523.9) | 1844 (1622.3 to 2101.6) | 7442.4 (6350 to 8671.8) | -99.4 (-99.5 to -99.3) | -99.8 (-99.8 to -99.8) | -98.9 (-99.1 to -98.7) |
|  |  |  | Rate | 135252.7 (123291.6 to 148701.8) | 156976 (143018.4 to 172472) | 114154.1 (104286.6 to 125213.6) | 1511.2 (1320.9 to 1712.6) | 613.2 (539.5 to 698.8) | 2372 (2023.8 to 2763.8) | -98.9 (-99.1 to -98.7) | -99.6 (-99.7 to -99.5) | -97.9 (-98.3 to -97.5) |
|  | Non-communicable diseases | DALYs (Disability-Adjusted Life Years) | Number | 125071 (98118 to 154263.8) | 59369 (44105 to 73944.9) | 65702 (48983.9 to 83236.1) | 31800.6 (24640.4 to 40426) | 16169 (12162.9 to 20898.6) | 15631.5 (12306.7 to 19632.8) | -74.6 (-80.6 to -66.5) | -72.8 (-79.6 to -62.8) | -76.2 (-82 to -66.5) |
|  |  |  | Rate | 10689.7 (8386.1 to 13184.8) | 10298.7 (7650.9 to 12827.1) | 11069.5 (8252.9 to 14023.7) | 5175.1 (4009.9 to 6578.8) | 5376.6 (4044.4 to 6949.3) | 4982 (3922.3 to 6257.2) | -51.6 (-63.1 to -36.3) | -47.8 (-61 to -28.7) | -55 (-66 to -36.6) |
|  |  | YLDs (Years Lived with Disability) | Number | 44186.3 (32386.8 to 58636.5) | 23288.6 (16989.6 to 31035.2) | 20897.7 (15344.8 to 27936.4) | 25651.8 (18411.1 to 34395.7) | 13733.5 (9779.6 to 18367.6) | 11918.3 (8630 to 15919.8) | -41.9 (-46 to -37.1) | -41 (-45.1 to -36) | -43 (-47.4 to -37.5) |
|  |  |  | Rate | 3776.6 (2768.1 to 5011.6) | 4039.8 (2947.2 to 5383.6) | 3520.9 (2585.3 to 4706.8) | 4174.5 (2996.1 to 5597.4) | 4566.7 (3251.9 to 6107.6) | 3798.5 (2750.5 to 5073.8) | 10.5 (2.8 to 19.7) | 13 (5.2 to 22.8) | 7.9 (-0.6 to 18.2) |
|  |  | YLLs (Years of Life Lost) | Number | 80884.7 (54803.2 to 104617.9) | 36080.4 (22183.6 to 48813.2) | 44804.3 (27922.5 to 59599.2) | 6148.8 (5343.8 to 7088.8) | 2435.6 (2062.7 to 2882.1) | 3713.2 (3137.9 to 4427.5) | -92.4 (-94.4 to -87.7) | -93.2 (-95.3 to -87.5) | -91.7 (-94.2 to -85.5) |
|  |  |  | Rate | 6913.2 (4684 to 8941.6) | 6258.8 (3848.2 to 8467.6) | 7548.7 (4704.4 to 10041.3) | 1000.6 (869.6 to 1153.6) | 809.9 (685.9 to 958.4) | 1183.5 (1000.1 to 1411.1) | -85.5 (-89.4 to -76.7) | -87.1 (-91 to -76) | -84.3 (-89.1 to -72.5) |
| Golestan | All causes | DALYs (Disability-Adjusted Life Years) | Number | 381106 (338675.7 to 429938.8) | 168989.3 (149157.4 to 191288.3) | 212116.6 (188671.2 to 238250.9) | 69118.6 (58956 to 81344.2) | 29667.4 (24393.5 to 35698) | 39451.2 (34464.2 to 45448.1) | -81.9 (-84.8 to -78.4) | -82.4 (-85.7 to -79) | -81.4 (-84.2 to -77.9) |
|  |  |  | Rate | 48573.7 (43165.8 to 54797.7) | 43453.3 (38353.7 to 49187.1) | 53606.3 (47681.1 to 60210.9) | 10234.1 (8729.4 to 12044.3) | 8959.2 (7366.6 to 10780.4) | 11460.5 (10011.8 to 13202.6) | -78.9 (-82.4 to -75) | -79.4 (-83.2 to -75.3) | -78.6 (-81.9 to -74.6) |
|  |  | YLDs (Years Lived with Disability) | Number | 42369.3 (31215.3 to 56634) | 21503.5 (15769 to 28679.1) | 20865.7 (15412.6 to 28292.2) | 33847 (24717.3 to 45311.6) | 17686.1 (12895.3 to 23912.4) | 16160.9 (11729.4 to 21463.6) | -20.1 (-26.6 to -12.2) | -17.8 (-25.3 to -9.2) | -22.5 (-31.8 to -12.6) |
|  |  |  | Rate | 5400.2 (3978.5 to 7218.3) | 5529.3 (4054.8 to 7374.4) | 5273.2 (3895.1 to 7150) | 5011.6 (3659.8 to 6709.1) | 5341 (3894.3 to 7221.3) | 4694.7 (3407.4 to 6235.1) | -7.2 (-14.7 to 2) | -3.4 (-12.2 to 6.7) | -11 (-21.6 to 0.5) |
|  |  | YLLs (Years of Life Lost) | Number | 338736.7 (297376.6 to 382899.1) | 147485.8 (129639.8 to 167068.3) | 191250.9 (168042.4 to 216192.6) | 35271.6 (31875.7 to 38499.8) | 11981.3 (10725.2 to 13234.7) | 23290.3 (21124.2 to 25563.5) | -89.6 (-91.1 to -87.8) | -91.9 (-93.2 to -90.3) | -87.8 (-89.6 to -85.6) |
|  |  |  | Rate | 43173.6 (37902 to 48802.3) | 37923.9 (33335.1 to 42959.3) | 48333.1 (42467.8 to 54636.3) | 5222.5 (4719.7 to 5700.5) | 3618.2 (3238.9 to 3996.7) | 6765.8 (6136.5 to 7426.1) | -87.9 (-89.6 to -85.9) | -90.5 (-92 to -88.7) | -86 (-88 to -83.5) |
|  | Communicable, maternal, neonatal, and nutritional diseases | DALYs (Disability-Adjusted Life Years) | Number | 174052.2 (144903.6 to 215235.2) | 76948.6 (62919.2 to 99813.5) | 97103.7 (80735.6 to 120919) | 17197.2 (14662 to 20338.1) | 8108 (6698.3 to 9935.8) | 9089.3 (7638.4 to 10720) | -90.1 (-92.5 to -87.5) | -89.5 (-92.4 to -86) | -90.6 (-93.3 to -87.9) |
|  |  |  | Rate | 22183.8 (18468.6 to 27432.7) | 19786.3 (16178.8 to 25665.7) | 24540.1 (20403.6 to 30558.7) | 2546.3 (2171 to 3011.4) | 2448.5 (2022.8 to 3000.5) | 2640.4 (2218.9 to 3114.1) | -88.5 (-91.3 to -85.4) | -87.6 (-91.1 to -83.5) | -89.2 (-92.3 to -86.1) |
|  |  | YLDs (Years Lived with Disability) | Number | 10550.8 (7227.4 to 14797.5) | 5077.1 (3292.9 to 7393.5) | 5473.7 (3531.2 to 8100.7) | 6734.9 (4640 to 9646.5) | 3577 (2401.2 to 5304.1) | 3157.9 (2013.6 to 4659) | -36.2 (-50.9 to -15.7) | -29.5 (-46.6 to -4) | -42.3 (-60 to -16) |
|  |  |  | Rate | 1344.7 (921.2 to 1886) | 1305.5 (846.7 to 1901.1) | 1383.3 (892.4 to 2047.2) | 997.2 (687 to 1428.3) | 1080.2 (725.1 to 1601.8) | 917.4 (585 to 1353.4) | -25.8 (-43 to -2.1) | -17.3 (-37.3 to 12.7) | -33.7 (-54 to -3.4) |
|  |  | YLLs (Years of Life Lost) | Number | 163501.4 (133935.6 to 204239.1) | 71871.4 (58638.9 to 94352.9) | 91630 (74301.3 to 114623.4) | 10462.3 (8952.5 to 12069.6) | 4530.9 (3791 to 5285.9) | 5931.4 (4998.8 to 6870.2) | -93.6 (-95.4 to -91.8) | -93.7 (-95.8 to -91.8) | -93.5 (-95.4 to -91.6) |
|  |  |  | Rate | 20839 (17070.7 to 26031.2) | 18480.7 (15078.2 to 24261.5) | 23156.8 (18777.5 to 28967.7) | 1549.1 (1325.6 to 1787.1) | 1368.3 (1144.8 to 1596.3) | 1723.1 (1452.1 to 1995.8) | -92.6 (-94.6 to -90.5) | -92.6 (-95.1 to -90.4) | -92.6 (-94.7 to -90.3) |
|  | Injuries | DALYs (Disability-Adjusted Life Years) | Number | 67615.1 (58091.2 to 79175.4) | 25289.3 (20575 to 30998.1) | 42325.8 (36139.2 to 50635.7) | 13278.3 (11881.2 to 14748.6) | 3015.9 (2676.7 to 3357.7) | 10262.4 (9016 to 11570.7) | -80.4 (-84 to -76.5) | -88.1 (-90.8 to -85.1) | -75.8 (-81.1 to -70.7) |
|  |  |  | Rate | 8617.9 (7404 to 10091.3) | 6502.8 (5290.6 to 7970.7) | 10696.6 (9133.1 to 12796.7) | 1966.1 (1759.2 to 2183.8) | 910.8 (808.3 to 1014) | 2981.2 (2619.1 to 3361.3) | -77.2 (-81.4 to -72.7) | -86 (-89.2 to -82.5) | -72.1 (-78.3 to -66.3) |
|  |  | YLDs (Years Lived with Disability) | Number | 2825.8 (2093.4 to 3730.4) | 1310.9 (972.9 to 1731.5) | 1514.9 (1114.4 to 1987.2) | 1001.9 (734 to 1330.2) | 420 (308.9 to 556.5) | 581.9 (422.2 to 783.4) | -64.5 (-67.1 to -61.8) | -68 (-70.8 to -65.1) | -61.6 (-64.8 to -58.7) |
|  |  |  | Rate | 360.2 (266.8 to 475.5) | 337.1 (250.2 to 445.2) | 382.9 (281.6 to 502.2) | 148.3 (108.7 to 197) | 126.8 (93.3 to 168.1) | 169 (122.6 to 227.6) | -58.8 (-61.8 to -55.7) | -62.4 (-65.7 to -59) | -55.8 (-59.5 to -52.5) |
|  |  | YLLs (Years of Life Lost) | Number | 64789.3 (55007.4 to 75952.4) | 23978.5 (19206.3 to 29616.5) | 40810.8 (34838.1 to 49148.5) | 12276.4 (10870.2 to 13721.3) | 2595.9 (2276.8 to 2930.2) | 9680.5 (8482.6 to 10963.8) | -81.1 (-84.7 to -77.2) | -89.2 (-91.9 to -86.3) | -76.3 (-81.8 to -71) |
|  |  |  | Rate | 8257.7 (7011 to 9680.5) | 6165.7 (4938.6 to 7615.5) | 10313.7 (8804.3 to 12420.8) | 1817.7 (1609.5 to 2031.7) | 783.9 (687.6 to 884.9) | 2812.2 (2464.2 to 3184.9) | -78 (-82.3 to -73.5) | -87.3 (-90.5 to -84) | -72.7 (-79.1 to -66.6) |
|  | Non-communicable diseases | DALYs (Disability-Adjusted Life Years) | Number | 139438.6 (106651.6 to 165886.9) | 66751.4 (45300.7 to 80796.1) | 72687.2 (51901.6 to 87538.4) | 38310.2 (30687.4 to 47364.9) | 18444 (14373.7 to 23504.9) | 19866.2 (16280.6 to 24278.6) | -72.5 (-78.4 to -61.8) | -72.4 (-79 to -57.2) | -72.7 (-78.7 to -58.6) |
|  |  |  | Rate | 17772.1 (13593.2 to 21143) | 17164.2 (11648.5 to 20775.6) | 18369.6 (13116.6 to 22122.8) | 5672.4 (4543.8 to 7013.1) | 5569.9 (4340.7 to 7098.2) | 5771.1 (4729.5 to 7052.9) | -68.1 (-75 to -55.6) | -67.5 (-75.3 to -49.8) | -68.6 (-75.6 to -52.5) |
|  |  | YLDs (Years Lived with Disability) | Number | 28992.7 (21100.4 to 38824.2) | 15115.6 (11054.5 to 20208.6) | 13877.1 (10036.8 to 18606.4) | 26110.1 (18733.7 to 35264.3) | 13689 (9821.9 to 18603.7) | 12421.1 (9000.8 to 16673.9) | -9.9 (-17 to -1.8) | -9.4 (-16.6 to -1) | -10.5 (-18.1 to -1.3) |
|  |  |  | Rate | 3695.3 (2689.3 to 4948.3) | 3886.8 (2842.5 to 5196.4) | 3507 (2536.5 to 4702.2) | 3866 (2773.8 to 5221.5) | 4133.9 (2966.1 to 5618.1) | 3608.3 (2614.7 to 4843.7) | 4.6 (-3.5 to 14) | 6.4 (-2.1 to 16.2) | 2.9 (-5.8 to 13.5) |
|  |  | YLLs (Years of Life Lost) | Number | 110446 (77438.8 to 135367.2) | 51635.9 (30104.1 to 64413.7) | 58810.1 (37820.4 to 72343.9) | 12200 (10774.4 to 14209.2) | 4754.9 (4115.2 to 5547.9) | 7445.1 (6388.7 to 8829.2) | -89 (-91.4 to -82.3) | -90.8 (-93.1 to -82.3) | -87.3 (-90.2 to -78) |
|  |  |  | Rate | 14076.8 (9869.9 to 17253.2) | 13277.5 (7740.8 to 16563.1) | 14862.5 (9558 to 18282.8) | 1806.4 (1595.3 to 2103.9) | 1435.9 (1242.7 to 1675.4) | 2162.8 (1855.9 to 2564.9) | -87.2 (-90 to -79.4) | -89.2 (-91.9 to -79.2) | -85.4 (-88.8 to -74.7) |
| Hamadan | All causes | DALYs (Disability-Adjusted Life Years) | Number | 482343.5 (428507.6 to 534370.1) | 217240.2 (193134.4 to 241030.3) | 265103.3 (235698.3 to 294535.8) | 51455.1 (44412.2 to 59910.7) | 22998.2 (19285.8 to 27444) | 28457 (24833.6 to 32567.9) | -89.3 (-91.1 to -87.6) | -89.4 (-91.3 to -87.6) | -89.3 (-91 to -87.5) |
|  |  |  | Rate | 52185.3 (46360.8 to 57814.1) | 48655.5 (43256.5 to 53983.8) | 55483.8 (49329.6 to 61643.8) | 10581.1 (9132.8 to 12319.9) | 9760.5 (8185 to 11647.3) | 11352.5 (9907 to 12992.5) | -79.7 (-83.1 to -76.5) | -79.9 (-83.5 to -76.5) | -79.5 (-82.8 to -76.3) |
|  |  | YLDs (Years Lived with Disability) | Number | 51835.5 (37919.4 to 68962.6) | 25690.7 (18743.5 to 34243.1) | 26144.8 (19174.1 to 34924.2) | 24499.1 (17574.1 to 33177.7) | 12814.5 (9180.1 to 17323.5) | 11684.6 (8413.5 to 15711) | -52.7 (-56.5 to -48.2) | -50.1 (-54.7 to -44.8) | -55.3 (-59.4 to -50.5) |
|  |  |  | Rate | 5608.1 (4102.5 to 7461.1) | 5754 (4198 to 7669.5) | 5471.9 (4013 to 7309.3) | 5037.9 (3613.9 to 6822.6) | 5438.5 (3896.1 to 7352.2) | 4661.4 (3356.4 to 6267.7) | -10.2 (-17.3 to -1.5) | -5.5 (-14.1 to 4.7) | -14.8 (-22.6 to -5.7) |
|  |  | YLLs (Years of Life Lost) | Number | 430508 (379725 to 478778.1) | 191549.5 (167989 to 216156.7) | 238958.5 (210804.6 to 266554.8) | 26956 (25061.4 to 28942.2) | 10183.7 (9420 to 11096.2) | 16772.3 (15336.8 to 18251) | -93.7 (-94.5 to -92.7) | -94.7 (-95.4 to -93.9) | -93 (-93.9 to -91.8) |
|  |  |  | Rate | 46577.2 (41082.9 to 51799.6) | 42901.5 (37624.7 to 48412.8) | 50011.9 (44119.6 to 55787.6) | 5543.2 (5153.6 to 5951.6) | 4322 (3997.9 to 4709.3) | 6691.1 (6118.4 to 7281) | -88.1 (-89.6 to -86.2) | -89.9 (-91.3 to -88.4) | -86.6 (-88.4 to -84.5) |
|  | Communicable, maternal, neonatal, and nutritional diseases | DALYs (Disability-Adjusted Life Years) | Number | 227626.3 (189875.4 to 283443.9) | 105365.6 (86942.1 to 137223.2) | 122260.7 (102124.3 to 151860.5) | 12706.8 (10944.4 to 15004.6) | 6180.8 (5177.9 to 7439.4) | 6526 (5560 to 7714.1) | -94.4 (-95.7 to -93) | -94.1 (-95.8 to -92.6) | -94.7 (-96 to -93.2) |
|  |  |  | Rate | 24627.2 (20542.8 to 30666.1) | 23598.8 (19472.5 to 30734) | 25588.1 (21373.7 to 31783.1) | 2613 (2250.6 to 3085.5) | 2623.1 (2197.5 to 3157.3) | 2603.5 (2218.1 to 3077.4) | -89.4 (-91.8 to -86.8) | -88.9 (-92 to -85.9) | -89.8 (-92.3 to -87.1) |
|  |  | YLDs (Years Lived with Disability) | Number | 12924.1 (8898.4 to 18378) | 6215 (4044 to 8932) | 6709.1 (4435.1 to 9885.2) | 4746 (3282.5 to 6706.1) | 2547.4 (1693.1 to 3715.5) | 2198.6 (1474.1 to 3283.9) | -63.3 (-70.8 to -52.3) | -59 (-68.5 to -44.9) | -67.2 (-75.8 to -53.8) |
|  |  |  | Rate | 1398.3 (962.7 to 1988.3) | 1392 (905.7 to 2000.5) | 1404.1 (928.2 to 2068.9) | 976 (675 to 1379) | 1081.1 (718.6 to 1576.9) | 877.1 (588.1 to 1310.1) | -30.2 (-44.5 to -9.4) | -22.3 (-40.4 to 4.4) | -37.5 (-53.8 to -12) |
|  |  | YLLs (Years of Life Lost) | Number | 214702.2 (176732.8 to 267826) | 99150.6 (80731.6 to 130626.7) | 115551.6 (95854.2 to 143396) | 7960.8 (6921.8 to 9158.4) | 3633.3 (3102.3 to 4222.2) | 4327.4 (3758.8 to 4968.1) | -96.3 (-97.2 to -95.3) | -96.3 (-97.4 to -95.3) | -96.3 (-97.3 to -95.3) |
|  |  |  | Rate | 23228.9 (19120.9 to 28976.4) | 22206.9 (18081.5 to 29256.6) | 24183.9 (20061.4 to 30011.5) | 1637 (1423.4 to 1883.3) | 1542 (1316.6 to 1791.9) | 1726.4 (1499.5 to 1981.9) | -93 (-94.7 to -91.1) | -93.1 (-95.1 to -91.1) | -92.9 (-94.8 to -91.1) |
|  | Injuries | DALYs (Disability-Adjusted Life Years) | Number | 94207.6 (81832.2 to 109165.4) | 35682.3 (28792.1 to 42697.3) | 58525.3 (50569.1 to 67656.7) | 10027.6 (9129 to 11078.1) | 2848.7 (2519.9 to 3169.3) | 7178.9 (6357.9 to 8100.3) | -89.4 (-91.2 to -87.3) | -92 (-93.7 to -90) | -87.7 (-90.2 to -85.1) |
|  |  |  | Rate | 10192.4 (8853.5 to 11810.7) | 7991.8 (6448.6 to 9563) | 12248.8 (10583.7 to 14160) | 2062 (1877.3 to 2278.1) | 1209 (1069.5 to 1345.1) | 2863.9 (2536.4 to 3231.5) | -79.8 (-83.3 to -75.9) | -84.9 (-88 to -81) | -76.6 (-81.3 to -71.6) |
|  |  | YLDs (Years Lived with Disability) | Number | 4101.3 (3048.6 to 5416.6) | 1763.8 (1288.1 to 2335.7) | 2337.6 (1749.6 to 3064.9) | 752.9 (548.8 to 1009.8) | 329.3 (240.2 to 444.6) | 423.5 (307.6 to 569.6) | -81.6 (-83.4 to -80) | -81.3 (-83.2 to -79.4) | -81.9 (-83.8 to -80) |
|  |  |  | Rate | 443.7 (329.8 to 586) | 395 (288.5 to 523.1) | 489.2 (366.2 to 641.5) | 154.8 (112.8 to 207.6) | 139.8 (101.9 to 188.7) | 169 (122.7 to 227.2) | -65.1 (-68.4 to -62) | -64.6 (-68.2 to -61) | -65.5 (-69.2 to -61.8) |
|  |  | YLLs (Years of Life Lost) | Number | 90106.2 (77759.5 to 105172.4) | 33918.5 (27017 to 41008.5) | 56187.7 (48110.3 to 65221.3) | 9274.7 (8356.4 to 10286) | 2519.4 (2215.3 to 2809.2) | 6755.3 (5943.9 to 7640.3) | -89.7 (-91.6 to -87.6) | -92.6 (-94.3 to -90.6) | -88 (-90.5 to -85.3) |
|  |  |  | Rate | 9748.7 (8412.9 to 11378.7) | 7596.8 (6051 to 9184.7) | 11759.6 (10069.1 to 13650.2) | 1907.2 (1718.4 to 2115.2) | 1069.2 (940.2 to 1192.3) | 2694.9 (2371.2 to 3048) | -80.4 (-84.1 to -76.5) | -85.9 (-89.1 to -82.1) | -77.1 (-81.8 to -72) |
|  | Non-communicable diseases | DALYs (Disability-Adjusted Life Years) | Number | 160509.7 (114754.1 to 193821.2) | 76192.3 (49721.8 to 94640.5) | 84317.4 (58315.6 to 102952.9) | 28563 (23040.5 to 35182.1) | 13919.7 (10979.5 to 17435.3) | 14643.3 (11998.5 to 17947.7) | -82.2 (-86.1 to -73.6) | -81.7 (-86 to -71.1) | -82.6 (-86.4 to -73.5) |
|  |  |  | Rate | 17365.7 (12415.4 to 20969.8) | 17064.9 (11136.2 to 21196.7) | 17646.9 (12205 to 21547.1) | 5873.6 (4738 to 7234.8) | 5907.6 (4659.8 to 7399.6) | 5841.7 (4786.6 to 7160) | -66.2 (-73.7 to -49.8) | -65.4 (-73.5 to -45.3) | -66.9 (-74.1 to -49.5) |
|  |  | YLDs (Years Lived with Disability) | Number | 34810.1 (24917 to 46257.1) | 17711.9 (12784 to 23505) | 17098.2 (12253.6 to 22677.2) | 19000.2 (13532.1 to 25605) | 9937.7 (7042 to 13371.2) | 9062.5 (6453.9 to 12191.8) | -45.4 (-49.9 to -41) | -43.9 (-48.4 to -39) | -47 (-51.8 to -42.2) |
|  |  |  | Rate | 3766.1 (2695.8 to 5004.6) | 3967 (2863.3 to 5264.4) | 3578.5 (2564.6 to 4746.1) | 3907.2 (2782.7 to 5265.3) | 4217.6 (2988.6 to 5674.8) | 3615.3 (2574.7 to 4863.7) | 3.7 (-4.7 to 12.2) | 6.3 (-2.2 to 15.5) | 1 (-8.2 to 10.1) |
|  |  | YLLs (Years of Life Lost) | Number | 125699.6 (82169.5 to 157047.1) | 58480.4 (31977.5 to 76630.9) | 67219.2 (41687.6 to 85143.8) | 9562.9 (8590.1 to 10834.3) | 3982 (3484.2 to 4543.5) | 5580.8 (4888.2 to 6472.4) | -92.4 (-94.1 to -87.7) | -93.2 (-94.8 to -86.6) | -91.7 (-93.6 to -85.4) |
|  |  |  | Rate | 13599.6 (8890 to 16991.1) | 13097.9 (7162 to 17163.1) | 14068.4 (8724.9 to 17819.8) | 1966.5 (1766.5 to 2227.9) | 1690 (1478.7 to 1928.3) | 2226.4 (1950.1 to 2582.1) | -85.5 (-88.7 to -76.6) | -87.1 (-90.2 to -74.7) | -84.2 (-87.8 to -72.2) |
| Hormozgan | All causes | DALYs (Disability-Adjusted Life Years) | Number | 280578.5 (251163.8 to 316938.1) | 125296.4 (110843.1 to 140941.3) | 155282.1 (138571.1 to 175929.9) | 73646.8 (62981.3 to 86093.6) | 31854.9 (26293.8 to 38591.5) | 41792 (36197.4 to 47625) | -73.8 (-78.1 to -69.3) | -74.6 (-79.2 to -69.7) | -73.1 (-77.3 to -68.6) |
|  |  |  | Rate | 53370.3 (47775.2 to 60286.5) | 48442.1 (42854.2 to 54490.7) | 58143.2 (51886 to 65874.5) | 10325.6 (8830.2 to 12070.6) | 9180.8 (7578 to 11122.3) | 11410 (9882.6 to 13002.5) | -80.7 (-83.9 to -77.4) | -81 (-84.5 to -77.4) | -80.4 (-83.5 to -77.1) |
|  |  | YLDs (Years Lived with Disability) | Number | 31004.7 (22664.5 to 41824.3) | 15392 (11211.4 to 20714.9) | 15612.8 (11237.3 to 20912.1) | 36288.8 (26400 to 49299.1) | 18881.3 (13652.3 to 25686.9) | 17407.5 (12684.6 to 23514.3) | 17 (7.2 to 28.8) | 22.7 (11.4 to 35.6) | 11.5 (-1.1 to 25.4) |
|  |  |  | Rate | 5897.6 (4311.1 to 7955.6) | 5950.8 (4334.5 to 8008.8) | 5846 (4207.7 to 7830.2) | 5087.8 (3701.4 to 6911.9) | 5441.7 (3934.7 to 7403.1) | 4752.6 (3463.1 to 6419.9) | -13.7 (-21 to -5) | -8.6 (-17 to 1.1) | -18.7 (-27.9 to -8.5) |
|  |  | YLLs (Years of Life Lost) | Number | 249573.7 (221233.1 to 283380.6) | 109904.4 (97034.1 to 124631.7) | 139669.3 (123954.4 to 159641.3) | 37358.1 (34053.5 to 40604.2) | 12973.6 (11720.6 to 14327.5) | 24384.5 (22090.7 to 26718.7) | -85 (-87.2 to -82.5) | -88.2 (-90 to -86.1) | -82.5 (-85.1 to -79.4) |
|  |  |  | Rate | 47472.7 (42081.9 to 53903.3) | 42491.2 (37515.3 to 48185.1) | 52297.2 (46413 to 59775.5) | 5237.7 (4774.4 to 5692.9) | 3739.1 (3378 to 4129.3) | 6657.4 (6031.2 to 7294.7) | -89 (-90.6 to -87.1) | -91.2 (-92.5 to -89.6) | -87.3 (-89.1 to -85) |
|  | Communicable, maternal, neonatal, and nutritional diseases | DALYs (Disability-Adjusted Life Years) | Number | 149794.5 (124958.8 to 186224.2) | 68228.5 (56100.7 to 86177.7) | 81566 (67688.7 to 101715.2) | 19461.5 (16666.6 to 22887.6) | 9195.1 (7711.4 to 11170.3) | 10266.4 (8749.5 to 12102.2) | -87 (-90.3 to -83.9) | -86.5 (-90.2 to -82.9) | -87.4 (-90.6 to -84.3) |
|  |  |  | Rate | 28493.2 (23769.1 to 35422.7) | 26378.5 (21689.6 to 33318) | 30541.3 (25345.1 to 38085.8) | 2728.6 (2336.7 to 3208.9) | 2650.1 (2222.5 to 3219.4) | 2802.9 (2388.8 to 3304.1) | -90.4 (-92.8 to -88.2) | -90 (-92.7 to -87.3) | -90.8 (-93.1 to -88.6) |
|  |  | YLDs (Years Lived with Disability) | Number | 9684.6 (6621.2 to 13815) | 4509 (3042.8 to 6424.4) | 5175.6 (3304.9 to 7511.6) | 7239.6 (4893 to 10135.5) | 3831.6 (2519 to 5643.5) | 3408 (2308 to 4905.3) | -25.2 (-39.6 to -2.7) | -15 (-34.2 to 12) | -34.2 (-51.7 to -9.8) |
|  |  |  | Rate | 1842.2 (1259.5 to 2627.8) | 1743.3 (1176.4 to 2483.8) | 1937.9 (1237.5 to 2812.6) | 1015 (686 to 1421) | 1104.3 (726 to 1626.5) | 930.4 (630.1 to 1339.2) | -44.9 (-55.5 to -28.3) | -36.7 (-50.9 to -16.5) | -52 (-64.8 to -34.2) |
|  |  | YLLs (Years of Life Lost) | Number | 140109.9 (115206.2 to 176772) | 63719.5 (51828 to 81100.4) | 76390.4 (62550.5 to 96927.2) | 12222 (10655.3 to 13866.7) | 5363.5 (4481.6 to 6171.2) | 6858.4 (6006.8 to 7847.8) | -91.3 (-93.6 to -89.1) | -91.6 (-94.3 to -89.3) | -91 (-93.4 to -88.8) |
|  |  |  | Rate | 26651 (21914 to 33624.7) | 24635.2 (20037.7 to 31355) | 28603.3 (23421.2 to 36293.1) | 1713.6 (1493.9 to 1944.2) | 1545.8 (1291.6 to 1778.6) | 1872.5 (1640 to 2142.6) | -93.6 (-95.3 to -92) | -93.7 (-95.7 to -92) | -93.5 (-95.2 to -91.9) |
|  | Injuries | DALYs (Disability-Adjusted Life Years) | Number | 49374.1 (41062.4 to 58236.4) | 19467.1 (15052.9 to 23671.5) | 29907.1 (24787.2 to 35797.6) | 13934.4 (12137.8 to 15630.6) | 3170.8 (2790.4 to 3557.3) | 10763.6 (9184.2 to 12251.6) | -71.8 (-77.1 to -64.8) | -83.7 (-87.1 to -78.6) | -64 (-71.4 to -55.3) |
|  |  |  | Rate | 9391.7 (7810.7 to 11077.5) | 7526.4 (5819.7 to 9151.9) | 11198.3 (9281.2 to 13403.9) | 1953.6 (1701.8 to 2191.5) | 913.8 (804.2 to 1025.2) | 2938.7 (2507.5 to 3344.9) | -79.2 (-83.2 to -74.1) | -87.9 (-90.4 to -84) | -73.8 (-79.2 to -67.4) |
|  |  | YLDs (Years Lived with Disability) | Number | 1939.8 (1428.7 to 2554) | 895.7 (660.5 to 1190) | 1044.2 (767.3 to 1379.2) | 973 (709.9 to 1298.9) | 412.8 (302 to 553.9) | 560.1 (406 to 753.8) | -49.8 (-54.2 to -45.6) | -53.9 (-59 to -48.5) | -46.4 (-51.3 to -41.5) |
|  |  |  | Rate | 369 (271.8 to 485.8) | 346.3 (255.4 to 460.1) | 391 (287.3 to 516.4) | 136.4 (99.5 to 182.1) | 119 (87 to 159.6) | 152.9 (110.8 to 205.8) | -63 (-66.2 to -59.9) | -65.6 (-69.4 to -61.6) | -60.9 (-64.5 to -57.4) |
|  |  | YLLs (Years of Life Lost) | Number | 47434.3 (38933.1 to 56022.5) | 18571.4 (14188.2 to 22708.3) | 28862.9 (23690 to 34785) | 12961.4 (11132.4 to 14556.8) | 2758 (2384 to 3134) | 10203.4 (8600.8 to 11748.3) | -72.7 (-78.1 to -65.5) | -85.1 (-88.5 to -80) | -64.6 (-72.2 to -55.8) |
|  |  |  | Rate | 9022.7 (7405.7 to 10656.3) | 7180.1 (5485.5 to 8779.5) | 10807.3 (8870.4 to 13024.8) | 1817.2 (1560.8 to 2040.9) | 794.9 (687.1 to 903.2) | 2785.7 (2348.2 to 3207.5) | -79.9 (-83.9 to -74.6) | -88.9 (-91.4 to -85.1) | -74.2 (-79.8 to -67.8) |
|  | Non-communicable diseases | DALYs (Disability-Adjusted Life Years) | Number | 81409.8 (57488.6 to 103288.1) | 37600.9 (26044.6 to 47732.1) | 43808.9 (29172.5 to 56692.5) | 39951.7 (31487.3 to 49766.7) | 19397 (14994.8 to 24940.4) | 20554.7 (16352.7 to 25016.1) | -50.9 (-62.7 to -26.1) | -48.4 (-61.3 to -20.8) | -53.1 (-65 to -24.3) |
|  |  |  | Rate | 15485.4 (10935.2 to 19647) | 14537.3 (10069.4 to 18454.2) | 16403.7 (10923.2 to 21227.7) | 5601.4 (4414.6 to 6977.5) | 5590.4 (4321.6 to 7188) | 5611.8 (4464.6 to 6829.9) | -63.8 (-72.5 to -45.5) | -61.5 (-71.1 to -41) | -65.8 (-74.5 to -44.8) |
|  |  | YLDs (Years Lived with Disability) | Number | 19380.3 (14182.5 to 25938.6) | 9987.3 (7284.9 to 13364.1) | 9393 (6869.7 to 12578.9) | 28076.2 (20101.7 to 38112.8) | 14636.9 (10401.7 to 20078.5) | 13439.4 (9626.9 to 17960.4) | 44.9 (33.6 to 58.4) | 46.6 (34.8 to 61.1) | 43.1 (31.1 to 57.3) |
|  |  |  | Rate | 3686.4 (2697.7 to 4933.9) | 3861.3 (2816.5 to 5166.8) | 3517.1 (2572.3 to 4710) | 3936.4 (2818.3 to 5343.6) | 4218.4 (2997.8 to 5786.8) | 3669.2 (2628.3 to 4903.5) | 6.8 (-1.5 to 16.7) | 9.2 (0.5 to 20.1) | 4.3 (-4.4 to 14.7) |
|  |  | YLLs (Years of Life Lost) | Number | 62029.5 (38901.1 to 82631.1) | 27613.6 (16515.9 to 36821.1) | 34415.9 (19934.4 to 47316) | 11875.5 (10554.4 to 13494.5) | 4760.1 (4060.5 to 5643.5) | 7115.3 (6196.6 to 8246.7) | -80.9 (-85.8 to -66.7) | -82.8 (-87.5 to -67.3) | -79.3 (-84.8 to -59.8) |
|  |  |  | Rate | 11799 (7399.6 to 15717.7) | 10676 (6385.4 to 14235.8) | 12886.6 (7464.2 to 17716.8) | 1665 (1479.8 to 1892) | 1371.9 (1170.3 to 1626.5) | 1942.6 (1691.8 to 2251.5) | -85.9 (-89.5 to -75.5) | -87.1 (-90.7 to -75.7) | -84.9 (-88.9 to -70.7) |
| Ilam | All causes | DALYs (Disability-Adjusted Life Years) | Number | 113451.6 (101405.5 to 125983.2) | 52310.5 (46300.5 to 58254.7) | 61141.1 (54193 to 67898.3) | 16674.3 (13796.9 to 19551.9) | 7603.5 (6092.4 to 9122.5) | 9070.8 (7672.2 to 10492.4) | -85.3 (-88.3 to -82.4) | -85.5 (-88.6 to -82.3) | -85.2 (-87.9 to -82.2) |
|  |  |  | Rate | 42714.5 (38179.2 to 47432.7) | 40325.3 (35692.3 to 44907.6) | 44995.5 (39882.2 to 49968.4) | 9660.7 (7993.6 to 11327.9) | 9126 (7312.3 to 10949.1) | 10159.7 (8593.2 to 11752) | -77.4 (-82 to -72.9) | -77.4 (-82.3 to -72.4) | -77.4 (-81.5 to -72.9) |
|  |  | YLDs (Years Lived with Disability) | Number | 14453.2 (10600.7 to 19448.4) | 7497.8 (5451.9 to 10039.9) | 6955.4 (5052.6 to 9194.7) | 8718 (6268.6 to 11642.7) | 4606.5 (3325 to 6190) | 4111.5 (2990 to 5490) | -39.7 (-44.6 to -33.8) | -38.6 (-43.6 to -32.5) | -40.9 (-47.4 to -33.6) |
|  |  |  | Rate | 5441.6 (3991.2 to 7322.3) | 5779.9 (4202.8 to 7739.6) | 5118.7 (3718.4 to 6766.6) | 5051 (3631.9 to 6745.5) | 5528.9 (3990.8 to 7429.5) | 4605 (3348.9 to 6149) | -7.2 (-14.8 to 1.8) | -4.3 (-12.2 to 5.1) | -10 (-20 to 1.1) |
|  |  | YLLs (Years of Life Lost) | Number | 98998.4 (87028.2 to 110472.4) | 44812.7 (39469.7 to 50391.4) | 54185.7 (47659.9 to 60575) | 7956.3 (6620.5 to 9352.9) | 2997 (2459.9 to 3621.2) | 4959.4 (4140.6 to 5742.1) | -92 (-93.7 to -89.9) | -93.3 (-94.9 to -91.3) | -90.8 (-92.7 to -88.6) |
|  |  |  | Rate | 37272.9 (32766.1 to 41592.9) | 34545.4 (30426.5 to 38845.8) | 39876.8 (35074.3 to 44578.9) | 4609.7 (3835.8 to 5418.8) | 3597.1 (2952.4 to 4346.4) | 5554.7 (4637.7 to 6431.4) | -87.6 (-90.2 to -84.5) | -89.6 (-92.1 to -86.4) | -86.1 (-88.9 to -82.7) |
|  | Communicable, maternal, neonatal, and nutritional diseases | DALYs (Disability-Adjusted Life Years) | Number | 52771.3 (44570.2 to 64711.6) | 24522.4 (20314.7 to 30916.5) | 28248.9 (23816.9 to 34907.4) | 4256 (3396.7 to 5139.4) | 2026.4 (1605 to 2527.8) | 2229.6 (1748.2 to 2689.6) | -91.9 (-94.1 to -89.4) | -91.7 (-94.2 to -88.9) | -92.1 (-94.2 to -89.7) |
|  |  |  | Rate | 19868.4 (16780.7 to 24363.9) | 18903.9 (15660.3 to 23833) | 20789.2 (17527.5 to 25689.4) | 2465.8 (1968 to 2977.6) | 2432.2 (1926.4 to 3033.9) | 2497.2 (1958.1 to 3012.4) | -87.6 (-90.9 to -83.6) | -87.1 (-90.9 to -82.8) | -88 (-91.2 to -84.3) |
|  |  | YLDs (Years Lived with Disability) | Number | 3693.7 (2518.7 to 5344.9) | 1911.1 (1254.8 to 2757.2) | 1782.5 (1160.6 to 2658.3) | 1634.7 (1104.4 to 2339.9) | 888.4 (588.5 to 1298.7) | 746.2 (508.3 to 1092.7) | -55.7 (-65.7 to -42.6) | -53.5 (-64.3 to -38.5) | -58.1 (-71.1 to -37.1) |
|  |  |  | Rate | 1390.7 (948.3 to 2012.3) | 1473.3 (967.3 to 2125.5) | 1311.8 (854.1 to 1956.3) | 947.1 (639.8 to 1355.7) | 1066.3 (706.3 to 1558.7) | 835.8 (569.3 to 1223.9) | -31.9 (-47.2 to -11.6) | -27.6 (-44.5 to -4.2) | -36.3 (-56 to -4.2) |
|  |  | YLLs (Years of Life Lost) | Number | 49077.6 (41096 to 60653.6) | 22611.3 (18484.2 to 28769.1) | 26466.3 (21936.9 to 32577.7) | 2621.3 (2013.1 to 3287.3) | 1138 (855.8 to 1444.7) | 1483.3 (1128.3 to 1869.9) | -94.7 (-96.4 to -92.5) | -95 (-96.6 to -92.8) | -94.4 (-96.2 to -92.2) |
|  |  |  | Rate | 18477.7 (15472.7 to 22836.1) | 17430.6 (14249.2 to 22177.6) | 19477.3 (16144 to 23974.9) | 1518.7 (1166.4 to 1904.6) | 1365.8 (1027.2 to 1734) | 1661.4 (1263.7 to 2094.4) | -91.8 (-94.4 to -88.5) | -92.2 (-94.8 to -88.7) | -91.5 (-94.2 to -88.1) |
|  | Injuries | DALYs (Disability-Adjusted Life Years) | Number | 20198.3 (16960.8 to 23542.3) | 8400.3 (6796.9 to 10270) | 11798 (9875.1 to 13677.3) | 2944.1 (2576.4 to 3337.5) | 861 (742.1 to 977.1) | 2083.1 (1783.1 to 2413.6) | -85.4 (-88.1 to -81.8) | -89.8 (-92.3 to -86.8) | -82.3 (-85.9 to -78.3) |
|  |  |  | Rate | 7604.7 (6385.8 to 8863.7) | 6475.6 (5239.6 to 7917) | 8682.5 (7267.4 to 10065.5) | 1705.7 (1492.7 to 1933.7) | 1033.4 (890.7 to 1172.7) | 2333.1 (1997.2 to 2703.3) | -77.6 (-81.7 to -72) | -84 (-88.1 to -79.5) | -73.1 (-78.6 to -67) |
|  |  | YLDs (Years Lived with Disability) | Number | 920.9 (682.1 to 1207.6) | 434.1 (319.5 to 576.3) | 486.9 (357 to 641) | 246.4 (179.3 to 329.9) | 106.7 (76.8 to 141.8) | 139.7 (100.2 to 188.8) | -73.2 (-75.3 to -71.2) | -75.4 (-77.8 to -73) | -71.3 (-73.5 to -69.1) |
|  |  |  | Rate | 346.7 (256.8 to 454.7) | 334.6 (246.3 to 444.3) | 358.3 (262.7 to 471.7) | 142.8 (103.9 to 191.1) | 128.1 (92.1 to 170.1) | 156.4 (112.2 to 211.4) | -58.8 (-61.9 to -55.7) | -61.7 (-65.4 to -58) | -56.3 (-59.7 to -52.9) |
|  |  | YLLs (Years of Life Lost) | Number | 19277.3 (16112 to 22589) | 7966.2 (6336.6 to 9850.7) | 11311.1 (9392.9 to 13228.3) | 2697.7 (2330 to 3080.2) | 754.3 (640.9 to 869) | 1943.4 (1656.4 to 2270.7) | -86 (-88.8 to -82.2) | -90.5 (-93.1 to -87.6) | -82.8 (-86.6 to -78.7) |
|  |  |  | Rate | 7257.9 (6066.2 to 8504.8) | 6141 (4884.8 to 7593.7) | 8324.2 (6912.5 to 9735.1) | 1563 (1350 to 1784.6) | 905.3 (769.3 to 1043) | 2176.7 (1855.3 to 2543.3) | -78.5 (-82.8 to -72.7) | -85.3 (-89.3 to -80.6) | -73.9 (-79.5 to -67.5) |
|  | Non-communicable diseases | DALYs (Disability-Adjusted Life Years) | Number | 40482 (32512.8 to 47173.2) | 19387.8 (13409.7 to 22945.1) | 21094.2 (15835.4 to 24987.3) | 9426.7 (7327.6 to 11616) | 4705.1 (3584.1 to 5957.6) | 4721.7 (3720.7 to 5764.5) | -76.7 (-82.2 to -67.5) | -75.7 (-82.2 to -63.7) | -77.6 (-83.1 to -67.7) |
|  |  |  | Rate | 15241.5 (12241.1 to 17760.7) | 14945.7 (10337.3 to 17688) | 15523.8 (11653.8 to 18388.8) | 5461.6 (4245.5 to 6730.1) | 5647.2 (4301.8 to 7150.5) | 5288.5 (4167.4 to 6456.5) | -64.2 (-72.7 to -50) | -62.2 (-72.2 to -43.5) | -65.9 (-74.2 to -50.9) |
|  |  | YLDs (Years Lived with Disability) | Number | 9838.5 (7267.5 to 13204.8) | 5152.6 (3754.2 to 6913.2) | 4686 (3449.8 to 6256.1) | 6836.9 (4898.7 to 9107.3) | 3611.4 (2561.7 to 4865.7) | 3225.6 (2319.2 to 4292) | -30.5 (-35.3 to -24.8) | -29.9 (-35.3 to -24.2) | -31.2 (-36.4 to -25.1) |
|  |  |  | Rate | 3704.2 (2736.2 to 4971.6) | 3972 (2894 to 5329.2) | 3448.5 (2538.8 to 4604.1) | 3961.2 (2838.2 to 5276.6) | 4334.5 (3074.7 to 5840) | 3612.8 (2597.6 to 4807.2) | 6.9 (-0.5 to 15.7) | 9.1 (0.8 to 18) | 4.8 (-3.3 to 13.9) |
|  |  | YLLs (Years of Life Lost) | Number | 30643.5 (22048.5 to 36383.2) | 14235.3 (8404.8 to 17598.5) | 16408.2 (11183.9 to 20152.3) | 2589.8 (2003.1 to 3244.3) | 1093.7 (850.9 to 1414.3) | 1496.1 (1158.1 to 1940.6) | -91.5 (-94.1 to -86.8) | -92.3 (-94.7 to -85) | -90.9 (-93.7 to -85) |
|  |  |  | Rate | 11537.3 (8301.3 to 13698.3) | 10973.7 (6479.1 to 13566.4) | 12075.3 (8230.6 to 14830.7) | 1500.5 (1160.6 to 1879.7) | 1312.7 (1021.3 to 1697.5) | 1675.7 (1297.1 to 2173.6) | -87 (-90.9 to -79.7) | -88 (-91.7 to -76.6) | -86.1 (-90.5 to -77.1) |
| Isfahan | All causes | DALYs (Disability-Adjusted Life Years) | Number | 612457.7 (548839.5 to 682568.2) | 260475.3 (233314.5 to 292869.7) | 351982.4 (314887.6 to 392284.3) | 141000.9 (115817.6 to 167953.6) | 66388.6 (53391.2 to 80382.6) | 74612.3 (62849.9 to 88090.3) | -77 (-80.8 to -73.1) | -74.5 (-79 to -70) | -78.8 (-82.2 to -75.4) |
|  |  |  | Rate | 30362.8 (27208.9 to 33838.6) | 26558.7 (23789.3 to 29861.7) | 33962.8 (30383.5 to 37851.6) | 9376.7 (7702 to 11169.1) | 9050.2 (7278.4 to 10957.9) | 9687.7 (8160.5 to 11437.7) | -69.1 (-74.3 to -63.9) | -65.9 (-71.9 to -59.8) | -71.5 (-76 to -66.9) |
|  |  | YLDs (Years Lived with Disability) | Number | 108771.2 (79199.9 to 145708.8) | 55617.3 (40334.6 to 73789.7) | 53153.9 (38451 to 71496.1) | 78879.7 (57638.9 to 104785.1) | 42570.3 (30259.7 to 56499.4) | 36309.4 (26725.6 to 48410.3) | -27.5 (-32.7 to -21.3) | -23.5 (-29.8 to -16) | -31.7 (-38.2 to -24.3) |
|  |  |  | Rate | 5392.4 (3926.4 to 7223.6) | 5670.9 (4112.6 to 7523.8) | 5128.8 (3710.1 to 6898.7) | 5245.6 (3833.1 to 6968.3) | 5803.2 (4125 to 7702.1) | 4714.4 (3470.1 to 6285.6) | -2.7 (-9.8 to 5.5) | 2.3 (-6.2 to 12.3) | -8.1 (-16.8 to 1.8) |
|  |  | YLLs (Years of Life Lost) | Number | 503686.5 (445409.8 to 563769.1) | 204858 (181457.2 to 227349.3) | 298828.5 (262315.9 to 335552.7) | 62121.2 (55798.7 to 69326.9) | 23818.3 (21125.8 to 27040.8) | 38302.9 (34113 to 43184.6) | -87.7 (-89.5 to -85.5) | -88.4 (-90.3 to -86.1) | -87.2 (-89.3 to -84.8) |
|  |  |  | Rate | 24970.5 (22081.4 to 27949.1) | 20887.8 (18501.8 to 23181.1) | 28834 (25310.9 to 32377.5) | 4131.1 (3710.7 to 4610.3) | 3246.9 (2879.9 to 3686.2) | 4973.3 (4429.3 to 5607.1) | -83.5 (-86 to -80.5) | -84.5 (-87 to -81.4) | -82.8 (-85.6 to -79.5) |
|  | Communicable, maternal, neonatal, and nutritional diseases | DALYs (Disability-Adjusted Life Years) | Number | 251794.8 (213842.9 to 303653.2) | 109501.5 (90348.1 to 139630.5) | 142293.3 (119122 to 170835.2) | 31676.4 (26138.9 to 38938.4) | 15581.7 (12587.6 to 19561.6) | 16094.6 (13232.6 to 19884.5) | -87.4 (-90.4 to -84) | -85.8 (-89.6 to -81.5) | -88.7 (-91.6 to -85.5) |
|  |  |  | Rate | 12482.8 (10601.3 to 15053.7) | 11165 (9212.1 to 14237) | 13729.9 (11494.1 to 16483.9) | 2106.5 (1738.3 to 2589.4) | 2124.1 (1716 to 2666.7) | 2089.7 (1718.1 to 2581.8) | -83.1 (-87.1 to -78.6) | -81 (-86.1 to -75.2) | -84.8 (-88.7 to -80.5) |
|  |  | YLDs (Years Lived with Disability) | Number | 26588.3 (18358.9 to 38059.2) | 13433.4 (8742.4 to 19393.8) | 13154.9 (8601.5 to 19974.7) | 14270.3 (9782.4 to 20490.4) | 7914.9 (5436.2 to 11570.1) | 6355.5 (4248.5 to 9380.4) | -46.3 (-56.8 to -30.9) | -41.1 (-55 to -22) | -51.7 (-64.3 to -31.7) |
|  |  |  | Rate | 1318.1 (910.2 to 1886.8) | 1369.7 (891.4 to 1977.4) | 1269.3 (830 to 1927.4) | 949 (650.5 to 1362.6) | 1079 (741.1 to 1577.2) | 825.2 (551.6 to 1218) | -28 (-42 to -7.3) | -21.2 (-39.8 to 4.2) | -35 (-52 to -8.1) |
|  |  | YLLs (Years of Life Lost) | Number | 225206.5 (187529.8 to 273241.8) | 96068.1 (78217.7 to 126153.7) | 129138.4 (106054.6 to 157821.2) | 17406 (13875.2 to 21826.5) | 7666.9 (5965.8 to 9641.6) | 9739.2 (7793.9 to 12189.8) | -92.3 (-94.5 to -89.9) | -92 (-94.6 to -89.4) | -92.5 (-94.5 to -90.1) |
|  |  |  | Rate | 11164.7 (9296.9 to 13546.1) | 9795.3 (7975.3 to 12862.9) | 12460.6 (10233.2 to 15228.2) | 1157.5 (922.7 to 1451.5) | 1045.2 (813.3 to 1314.4) | 1264.5 (1012 to 1582.7) | -89.6 (-92.6 to -86.4) | -89.3 (-92.7 to -85.8) | -89.9 (-92.6 to -86.6) |
|  | Injuries | DALYs (Disability-Adjusted Life Years) | Number | 113353.3 (99965.9 to 128435.3) | 35986.8 (30723.3 to 43517.9) | 77366.5 (67421.2 to 87752.1) | 23321.7 (20838.9 to 26341.4) | 6812.6 (5981.7 to 7758.6) | 16509.1 (14479.3 to 19024.6) | -79.4 (-82.6 to -75.9) | -81.1 (-85 to -76.6) | -78.7 (-82.4 to -74.5) |
|  |  |  | Rate | 5619.5 (4955.8 to 6367.2) | 3669.3 (3132.6 to 4437.2) | 7465.1 (6505.5 to 8467.2) | 1550.9 (1385.8 to 1751.7) | 928.7 (815.4 to 1057.7) | 2143.6 (1880 to 2470.2) | -72.4 (-76.7 to -67.7) | -74.7 (-79.9 to -68.7) | -71.3 (-76.3 to -65.6) |
|  |  | YLDs (Years Lived with Disability) | Number | 6904.5 (5078.7 to 9093.1) | 2974.6 (2207.6 to 3899.9) | 3929.9 (2881.8 to 5206.5) | 2413.6 (1724.8 to 3241.3) | 1058.2 (762 to 1420.5) | 1355.4 (957.8 to 1834.7) | -65 (-67.6 to -62.3) | -64.4 (-67.6 to -61.3) | -65.5 (-68.6 to -62.5) |
|  |  |  | Rate | 342.3 (251.8 to 450.8) | 303.3 (225.1 to 397.6) | 379.2 (278.1 to 502.4) | 160.5 (114.7 to 215.5) | 144.3 (103.9 to 193.7) | 176 (124.4 to 238.2) | -53.1 (-56.6 to -49.4) | -52.4 (-56.7 to -48.3) | -53.6 (-57.8 to -49.6) |
|  |  | YLLs (Years of Life Lost) | Number | 106448.8 (93298.8 to 121175.6) | 33012.2 (27725.5 to 39959.5) | 73436.6 (63614 to 83754.3) | 20908.1 (18600.2 to 23942.3) | 5754.4 (5038.4 to 6648.5) | 15153.7 (13230.9 to 17702.3) | -80.4 (-83.7 to -76.6) | -82.6 (-86.6 to -77.8) | -79.4 (-83.2 to -74.9) |
|  |  |  | Rate | 5277.2 (4625.3 to 6007.3) | 3366 (2827 to 4074.4) | 7085.9 (6138.1 to 8081.5) | 1390.4 (1236.9 to 1592.2) | 784.5 (686.8 to 906.3) | 1967.6 (1717.9 to 2298.5) | -73.7 (-78.1 to -68.6) | -76.7 (-82.1 to -70.3) | -72.2 (-77.4 to -66.2) |
|  | Non-communicable diseases | DALYs (Disability-Adjusted Life Years) | Number | 247309.6 (203074.1 to 287549.7) | 114987 (86746.8 to 136912.5) | 132322.6 (106183.9 to 155782.7) | 85378.8 (66582.4 to 107604) | 43813.7 (33538.1 to 55741) | 41565.1 (32998 to 52294.5) | -65.5 (-72.3 to -56.1) | -61.9 (-69.6 to -47.4) | -68.6 (-74.9 to -58) |
|  |  |  | Rate | 12260.5 (10067.5 to 14255.4) | 11724.3 (8844.9 to 13959.9) | 12767.8 (10245.7 to 15031.5) | 5677.8 (4427.8 to 7155.8) | 5972.7 (4572 to 7598.7) | 5396.8 (4284.5 to 6790) | -53.7 (-62.8 to -41.2) | -49.1 (-59.3 to -29.6) | -57.7 (-66.3 to -43.4) |
|  |  | YLDs (Years Lived with Disability) | Number | 75278.4 (55698.8 to 100682.6) | 39209.3 (28643.2 to 51979.4) | 36069.2 (26265.6 to 47973.7) | 62195.7 (44824.6 to 83326.4) | 33597.3 (23779.9 to 45453) | 28598.4 (20893.6 to 38392.4) | -17.4 (-23.5 to -10.4) | -14.3 (-20.9 to -6.8) | -20.7 (-27.4 to -13) |
|  |  |  | Rate | 3732 (2761.3 to 4991.4) | 3997.9 (2920.5 to 5299.9) | 3480.3 (2534.4 to 4629) | 4136.1 (2980.9 to 5541.3) | 4580 (3241.7 to 6196.2) | 3713.2 (2712.8 to 4984.9) | 10.8 (2.7 to 20.2) | 14.6 (5.7 to 24.6) | 6.7 (-2.3 to 17) |
|  |  | YLLs (Years of Life Lost) | Number | 172031.2 (131045 to 205520.1) | 75777.8 (48004.2 to 91345.4) | 96253.4 (68622.9 to 117542.8) | 23183.1 (19359.4 to 27449.4) | 10216.4 (8543 to 12119.7) | 12966.6 (10567.3 to 15859.2) | -86.5 (-89.3 to -80.4) | -86.5 (-89.2 to -77.2) | -86.5 (-89.6 to -79.1) |
|  |  |  | Rate | 8528.5 (6496.6 to 10188.7) | 7726.5 (4894.6 to 9313.8) | 9287.5 (6621.4 to 11341.7) | 1541.7 (1287.4 to 1825.4) | 1392.7 (1164.6 to 1652.2) | 1683.6 (1372.1 to 2059.2) | -81.9 (-85.7 to -73.8) | -82 (-85.6 to -69.6) | -81.9 (-86 to -71.9) |
| Kerman | All causes | DALYs (Disability-Adjusted Life Years) | Number | 425235.7 (382724.8 to 477980.3) | 187621 (168859.5 to 210383.1) | 237614.7 (212149 to 267437.6) | 130293.1 (110787.3 to 151876.9) | 60288.3 (50729.4 to 72223.3) | 70004.9 (60477.9 to 80909.9) | -69.4 (-74.1 to -64.5) | -67.9 (-73.4 to -62.6) | -70.5 (-75.1 to -65.6) |
|  |  |  | Rate | 39650.1 (35686.3 to 44568.1) | 35479.7 (31931.8 to 39784.1) | 43706.6 (39022.5 to 49192.2) | 11157.6 (9487.3 to 13006) | 10581.2 (8903.5 to 12675.9) | 11706.9 (10113.7 to 13530.6) | -71.9 (-76.2 to -67.4) | -70.2 (-75.3 to -65.3) | -73.2 (-77.4 to -68.7) |
|  |  | YLDs (Years Lived with Disability) | Number | 62231.3 (45533.9 to 82140.4) | 31262.5 (22739.1 to 41957.9) | 30968.7 (22532.6 to 41123.2) | 63125.8 (45486.2 to 83927.5) | 33034.4 (24003.4 to 44349.1) | 30091.4 (21721.6 to 40368.4) | 1.4 (-7.2 to 10.7) | 5.7 (-3 to 15.7) | -2.8 (-13.3 to 8.7) |
|  |  |  | Rate | 5802.6 (4245.7 to 7659) | 5911.8 (4300 to 7934.4) | 5696.4 (4144.6 to 7564.2) | 5405.8 (3895.2 to 7187.1) | 5797.9 (4212.8 to 7783.7) | 5032.2 (3632.5 to 6750.8) | -6.8 (-14.8 to 1.7) | -1.9 (-10 to 7.4) | -11.7 (-21.2 to -1.2) |
|  |  | YLLs (Years of Life Lost) | Number | 363004.4 (322194.6 to 402789.2) | 156358.5 (137768.6 to 174385.9) | 206646 (181407.2 to 230214.6) | 67167.4 (62011.2 to 73593) | 27253.9 (24986 to 29955.5) | 39913.5 (36302.6 to 43953.4) | -81.5 (-84.2 to -78.4) | -82.6 (-85.2 to -79.6) | -80.7 (-83.7 to -77.4) |
|  |  |  | Rate | 33847.5 (30042.3 to 37557.1) | 29567.9 (26052.4 to 32976.9) | 38010.2 (33367.8 to 42345.4) | 5751.9 (5310.3 to 6302.1) | 4783.3 (4385.3 to 5257.5) | 6674.7 (6070.9 to 7350.3) | -83 (-85.4 to -80.1) | -83.8 (-86.3 to -81.1) | -82.4 (-85.2 to -79.4) |
|  | Communicable, maternal, neonatal, and nutritional diseases | DALYs (Disability-Adjusted Life Years) | Number | 206052.2 (170210.7 to 256139.1) | 94305.9 (77354.6 to 118325.6) | 111746.3 (92501.9 to 138571.3) | 35635 (30637.5 to 41761.5) | 17217 (14571 to 20775.1) | 18417.9 (15744.2 to 21815.3) | -82.7 (-86.6 to -78.4) | -81.7 (-86.4 to -76.9) | -83.5 (-87.6 to -79.2) |
|  |  |  | Rate | 19212.8 (15870.9 to 23883.1) | 17833.5 (14628 to 22375.7) | 20554.5 (17014.7 to 25488.7) | 3051.6 (2623.6 to 3576.2) | 3021.8 (2557.3 to 3646.2) | 3080 (2632.9 to 3648.2) | -84.1 (-87.7 to -80.2) | -83.1 (-87.4 to -78.6) | -85 (-88.7 to -81.1) |
|  |  | YLDs (Years Lived with Disability) | Number | 17159 (12029 to 24666.9) | 8338.5 (5533 to 11891.2) | 8820.5 (5854.8 to 13071.7) | 12356.5 (8509.1 to 17569.4) | 6572.7 (4459.8 to 9619.3) | 5783.8 (3853.4 to 8688.3) | -28 (-41.6 to -7.2) | -21.2 (-38.8 to 2.8) | -34.4 (-52 to -8.7) |
|  |  |  | Rate | 1600 (1121.6 to 2300) | 1576.8 (1046.3 to 2248.7) | 1622.4 (1076.9 to 2404.4) | 1058.1 (728.7 to 1504.6) | 1153.6 (782.7 to 1688.3) | 967.2 (644.4 to 1452.9) | -33.9 (-46.4 to -14.8) | -26.8 (-43.2 to -4.6) | -40.4 (-56.4 to -17) |
|  |  | YLLs (Years of Life Lost) | Number | 188893.2 (154140 to 236987.4) | 85967.4 (69513 to 109841.1) | 102925.8 (84143.4 to 128844.6) | 23278.5 (20252.8 to 27124.8) | 10644.3 (9065.2 to 12551) | 12634.2 (11000.2 to 14823.1) | -87.7 (-90.9 to -84.1) | -87.6 (-91.3 to -84.1) | -87.7 (-91 to -84.1) |
|  |  |  | Rate | 17612.9 (14372.4 to 22097.3) | 16256.7 (13145.1 to 20771.3) | 18932.1 (15477.3 to 23699.5) | 1993.5 (1734.3 to 2322.8) | 1868.2 (1591 to 2202.8) | 2112.8 (1839.6 to 2478.9) | -88.7 (-91.7 to -85.4) | -88.5 (-91.9 to -85.2) | -88.8 (-91.8 to -85.5) |
|  | Injuries | DALYs (Disability-Adjusted Life Years) | Number | 84605.5 (72765.8 to 97403.1) | 30502.2 (24589.2 to 35630.9) | 54103.3 (46504.2 to 63001.5) | 23819.1 (21372.7 to 26358.9) | 7535.7 (6754.1 to 8402.9) | 16283.5 (14227.6 to 18487.7) | -71.8 (-76.9 to -66.2) | -75.3 (-79.7 to -69.1) | -69.9 (-76.1 to -62.8) |
|  |  |  | Rate | 7888.8 (6784.9 to 9082.1) | 5768.1 (4649.9 to 6737.9) | 9951.7 (8553.9 to 11588.4) | 2039.7 (1830.2 to 2257.2) | 1322.6 (1185.4 to 1474.8) | 2723.1 (2379.3 to 3091.7) | -74.1 (-78.8 to -69) | -77.1 (-81.2 to -71.3) | -72.6 (-78.3 to -66.2) |
|  |  | YLDs (Years Lived with Disability) | Number | 4313.3 (3224.6 to 5628.8) | 1934.2 (1455.1 to 2531.8) | 2379.1 (1788.6 to 3130) | 2170.7 (1651.2 to 2800.6) | 1007.9 (763.5 to 1294) | 1162.8 (880.4 to 1523.7) | -49.7 (-54.2 to -45.3) | -47.9 (-53.9 to -41.5) | -51.1 (-56.4 to -46.3) |
|  |  |  | Rate | 402.2 (300.7 to 524.8) | 365.8 (275.2 to 478.8) | 437.6 (329 to 575.7) | 185.9 (141.4 to 239.8) | 176.9 (134 to 227.1) | 194.5 (147.2 to 254.8) | -53.8 (-58 to -49.8) | -51.6 (-57.3 to -45.7) | -55.6 (-60.4 to -51.2) |
|  |  | YLLs (Years of Life Lost) | Number | 80292.2 (68684.3 to 93011.7) | 28568 (22673.3 to 33589.7) | 51724.2 (44145.7 to 60623.2) | 21648.4 (19316.5 to 23957.4) | 6527.8 (5766.7 to 7377) | 15120.6 (13170.3 to 17303.5) | -73 (-78 to -67.2) | -77.2 (-81.5 to -70.7) | -70.8 (-77 to -63.4) |
|  |  |  | Rate | 7486.7 (6404.3 to 8672.7) | 5402.3 (4287.6 to 6351.9) | 9514.1 (8120.1 to 11151) | 1853.9 (1654.2 to 2051.6) | 1145.7 (1012.1 to 1294.7) | 2528.6 (2202.5 to 2893.7) | -75.2 (-79.8 to -69.8) | -78.8 (-82.8 to -72.8) | -73.4 (-79.1 to -66.7) |
|  | Non-communicable diseases | DALYs (Disability-Adjusted Life Years) | Number | 134578 (102595.6 to 164578) | 62812.9 (44670.7 to 77776) | 71765.1 (52711.9 to 89515.2) | 70362.7 (56073 to 87032.4) | 35390.7 (27981.4 to 44174.1) | 34972.1 (27965.4 to 42968.1) | -47.7 (-59.7 to -27.8) | -43.7 (-55.7 to -19.5) | -51.3 (-62.9 to -28.7) |
|  |  |  | Rate | 12548.4 (9566.3 to 15345.7) | 11878.1 (8447.4 to 14707.7) | 13200.4 (9695.8 to 16465.3) | 6025.5 (4801.8 to 7453) | 6211.4 (4911 to 7753) | 5848.4 (4676.7 to 7185.6) | -52 (-63 to -33.7) | -47.7 (-58.9 to -25.3) | -55.7 (-66.2 to -35.2) |
|  |  | YLDs (Years Lived with Disability) | Number | 40759 (29645.9 to 54064.7) | 20989.8 (15176.9 to 28021.3) | 19769.2 (14332.6 to 26160.4) | 48598.6 (35347.8 to 64511.3) | 25453.8 (18325.2 to 34055.3) | 23144.8 (16740.5 to 30620.9) | 19.2 (9.5 to 29.7) | 21.3 (11.7 to 31.9) | 17.1 (5.4 to 28.2) |
|  |  |  | Rate | 3800.5 (2764.3 to 5041.1) | 3969.2 (2870 to 5298.9) | 3636.3 (2636.3 to 4811.9) | 4161.7 (3027 to 5524.4) | 4467.4 (3216.3 to 5977) | 3870.5 (2799.5 to 5120.7) | 9.5 (0.5 to 19.1) | 12.6 (3.7 to 22.5) | 6.4 (-4.1 to 16.5) |
|  |  | YLLs (Years of Life Lost) | Number | 93819 (62490.6 to 122474.8) | 41823 (25100 to 55764.6) | 51996 (33525.6 to 69090.8) | 21764.1 (19242.2 to 25043.5) | 9936.9 (8644.8 to 11649.3) | 11827.2 (10187.3 to 14246.3) | -76.8 (-82.6 to -61.3) | -76.2 (-82.3 to -55.9) | -77.3 (-83.2 to -60) |
|  |  |  | Rate | 8747.9 (5826.8 to 11419.9) | 7908.9 (4746.5 to 10545.3) | 9564.1 (6166.7 to 12708.5) | 1863.8 (1647.8 to 2144.6) | 1744 (1517.2 to 2044.6) | 1977.9 (1703.6 to 2382.4) | -78.7 (-84 to -64.5) | -77.9 (-83.6 to -59.1) | -79.3 (-84.7 to -63.6) |
| Kermanshah | All causes | DALYs (Disability-Adjusted Life Years) | Number | 479080.7 (428450.2 to 535247.7) | 214105.3 (191121.4 to 240296.7) | 264975.4 (236190.9 to 297819.5) | 56665.5 (48394.4 to 65630.8) | 25462.6 (21087.6 to 30155) | 31203 (27060.2 to 35703.3) | -88.2 (-90.2 to -86) | -88.1 (-90.4 to -85.9) | -88.2 (-90.2 to -86.1) |
|  |  |  | Rate | 50420.9 (45092.3 to 56332.2) | 46687.5 (41675.7 to 52398.8) | 53903.8 (48048.2 to 60585.3) | 10460.8 (8933.9 to 12115.9) | 9840.9 (8150 to 11654.5) | 11027.7 (9563.6 to 12618.2) | -79.3 (-82.9 to -75.5) | -78.9 (-83 to -74.9) | -79.5 (-83 to -75.8) |
|  |  | YLDs (Years Lived with Disability) | Number | 52796.7 (38941.9 to 70498.1) | 26436.7 (19359.3 to 35560.2) | 26360 (19356.1 to 35291.4) | 27880.7 (20164.7 to 37122.4) | 14373.7 (10402.2 to 19090.3) | 13507 (9738.9 to 17943.9) | -47.2 (-51.6 to -42) | -45.6 (-50.6 to -40.4) | -48.8 (-53.9 to -42.3) |
|  |  |  | Rate | 5556.6 (4098.4 to 7419.6) | 5764.8 (4221.5 to 7754.2) | 5362.4 (3937.6 to 7179.3) | 5147 (3722.5 to 6853) | 5555.2 (4020.3 to 7378.1) | 4773.6 (3441.9 to 6341.7) | -7.4 (-15.1 to 1.7) | -3.6 (-12.4 to 5.6) | -11 (-19.9 to 0.2) |
|  |  | YLLs (Years of Life Lost) | Number | 426284 (376450.8 to 477688.5) | 187668.6 (165530.1 to 211416.8) | 238615.4 (210611.7 to 268186.2) | 28784.8 (26105.7 to 31825.5) | 11088.8 (9974.8 to 12324.6) | 17696 (15972.6 to 19665.6) | -93.2 (-94.2 to -91.9) | -94.1 (-95 to -93) | -92.6 (-93.7 to -91.1) |
|  |  |  | Rate | 44864.3 (39619.6 to 50274.4) | 40922.8 (36095.3 to 46101.3) | 48541.4 (42844.6 to 54557) | 5313.9 (4819.3 to 5875.2) | 4285.7 (3855.1 to 4763.3) | 6254.1 (5645 to 6950.2) | -88.2 (-89.9 to -85.8) | -89.5 (-91.1 to -87.5) | -87.1 (-89.1 to -84.5) |
|  | Communicable, maternal, neonatal, and nutritional diseases | DALYs (Disability-Adjusted Life Years) | Number | 243646.7 (203071.5 to 297437.2) | 110698.4 (91696.6 to 140871.2) | 132948.3 (109786.4 to 164446) | 14997.3 (12819.5 to 17631.2) | 7128.2 (5944.5 to 8623.4) | 7869.1 (6607.9 to 9286.6) | -93.8 (-95.3 to -92.2) | -93.6 (-95.3 to -91.8) | -94.1 (-95.7 to -92.5) |
|  |  |  | Rate | 25642.6 (21372.3 to 31303.8) | 24138.8 (19995.2 to 30718.2) | 27045.6 (22333.8 to 33453.2) | 2768.6 (2366.6 to 3254.8) | 2754.9 (2297.5 to 3332.8) | 2781.1 (2335.4 to 3282) | -89.2 (-91.7 to -86.4) | -88.6 (-91.7 to -85.4) | -89.7 (-92.4 to -87) |
|  |  | YLDs (Years Lived with Disability) | Number | 13576.6 (9165.4 to 19544.8) | 6728.8 (4575.9 to 9876.6) | 6847.8 (4352.9 to 10138.9) | 5280.6 (3568.6 to 7489.3) | 2812.4 (1874.9 to 4137.4) | 2468.2 (1635.6 to 3693.6) | -61.1 (-68.9 to -47.9) | -58.2 (-68 to -44.6) | -64 (-74 to -46.9) |
|  |  |  | Rate | 1428.9 (964.6 to 2057) | 1467.3 (997.8 to 2153.7) | 1393 (885.5 to 2062.5) | 974.8 (658.8 to 1382.6) | 1086.9 (724.6 to 1599.1) | 872.3 (578 to 1305.4) | -31.8 (-45.4 to -8.7) | -25.9 (-43.2 to -1.9) | -37.4 (-54.8 to -7.8) |
|  |  | YLLs (Years of Life Lost) | Number | 230070.2 (189999.3 to 283728.2) | 103969.6 (85163.1 to 133810.4) | 126100.6 (103186.7 to 157067.1) | 9716.6 (8361.7 to 11371.2) | 4315.8 (3590.1 to 5110.7) | 5400.9 (4626.2 to 6327.4) | -95.8 (-96.9 to -94.6) | -95.8 (-97.1 to -94.7) | -95.7 (-96.9 to -94.6) |
|  |  |  | Rate | 24213.8 (19996.5 to 29861) | 22671.5 (18570.6 to 29178.5) | 25652.6 (20991.2 to 31952.1) | 1793.7 (1543.6 to 2099.2) | 1668 (1387.5 to 1975.2) | 1908.8 (1635 to 2236.2) | -92.6 (-94.5 to -90.6) | -92.6 (-94.9 to -90.6) | -92.6 (-94.6 to -90.6) |
|  | Injuries | DALYs (Disability-Adjusted Life Years) | Number | 79942.2 (68090 to 92381.3) | 30180.3 (24105.7 to 36511.2) | 49761.8 (42874.6 to 57310.8) | 10279 (9198.2 to 11374.3) | 3169.1 (2786.7 to 3517.9) | 7109.9 (6208.2 to 8063.8) | -87.1 (-89.5 to -84.4) | -89.5 (-91.5 to -87) | -85.7 (-88.6 to -82.4) |
|  |  |  | Rate | 8413.5 (7166.1 to 9722.7) | 6581.1 (5256.4 to 7961.6) | 10123 (8722 to 11658.7) | 1897.6 (1698.1 to 2099.8) | 1224.8 (1077 to 1359.6) | 2512.8 (2194.1 to 2849.9) | -77.4 (-81.5 to -72.6) | -81.4 (-85 to -76.9) | -75.2 (-80.3 to -69.4) |
|  |  | YLDs (Years Lived with Disability) | Number | 3483.7 (2567.7 to 4620.4) | 1583.1 (1170.9 to 2114.8) | 1900.6 (1385 to 2513.7) | 840.9 (623.8 to 1120.5) | 370.2 (278 to 494.4) | 470.7 (343.2 to 634) | -75.9 (-77.8 to -74) | -76.6 (-78.8 to -74.2) | -75.2 (-77.4 to -73.2) |
|  |  |  | Rate | 366.6 (270.2 to 486.3) | 345.2 (255.3 to 461.1) | 386.6 (281.7 to 511.4) | 155.2 (115.1 to 206.9) | 143.1 (107.4 to 191.1) | 166.4 (121.3 to 224.1) | -57.7 (-61 to -54.4) | -58.6 (-62.4 to -54.3) | -57 (-60.7 to -53.4) |
|  |  | YLLs (Years of Life Lost) | Number | 76458.5 (64531.6 to 88585.9) | 28597.2 (22589.5 to 34746.6) | 47861.2 (40878.2 to 55657.6) | 9438.1 (8496.6 to 10472.4) | 2798.9 (2450 to 3126.8) | 6639.2 (5768.5 to 7589.4) | -87.7 (-89.9 to -84.8) | -90.2 (-92.1 to -87.7) | -86.1 (-89.1 to -82.7) |
|  |  |  | Rate | 8046.9 (6791.6 to 9323.2) | 6235.9 (4925.8 to 7576.8) | 9736.4 (8315.8 to 11322.4) | 1742.3 (1568.5 to 1933.3) | 1081.7 (946.9 to 1208.5) | 2346.4 (2038.7 to 2682.2) | -78.3 (-82.3 to -73.3) | -82.7 (-86 to -78.2) | -75.9 (-81.1 to -70) |
|  | Non-communicable diseases | DALYs (Disability-Adjusted Life Years) | Number | 155491.8 (113953.2 to 190195.3) | 73226.6 (47253.3 to 91270.2) | 82265.2 (57251.9 to 102466) | 31130.7 (24910.7 to 38129.6) | 15087.5 (11780 to 18995.4) | 16043.2 (13031.5 to 19481.6) | -80 (-84.5 to -71.3) | -79.4 (-84.1 to -66.4) | -80.5 (-85.1 to -70) |
|  |  |  | Rate | 16364.8 (11993 to 20017.1) | 15967.7 (10304 to 19902.3) | 16735.2 (11646.7 to 20844.6) | 5746.9 (4598.7 to 7039) | 5831.1 (4552.8 to 7341.4) | 5670 (4605.6 to 6885.2) | -64.9 (-72.8 to -49.7) | -63.5 (-71.8 to -40.4) | -66.1 (-74.1 to -48) |
|  |  | YLDs (Years Lived with Disability) | Number | 35736.5 (26132.5 to 47869.4) | 18124.9 (13288.5 to 24180.5) | 17611.6 (12872.1 to 23781.1) | 21759.2 (15771.1 to 28954.6) | 11191.2 (8065 to 14915.5) | 10568 (7780.8 to 14024.7) | -39.1 (-43.7 to -34.2) | -38.3 (-43 to -33.1) | -40 (-45.2 to -34.6) |
|  |  |  | Rate | 3761.1 (2750.3 to 5038) | 3952.3 (2897.7 to 5272.8) | 3582.7 (2618.6 to 4837.8) | 4016.9 (2911.4 to 5345.2) | 4325.2 (3117 to 5764.6) | 3734.9 (2749.9 to 4956.6) | 6.8 (-1.3 to 15.5) | 9.4 (1.1 to 18.7) | 4.2 (-4.7 to 13.7) |
|  |  | YLLs (Years of Life Lost) | Number | 119755.3 (78009.2 to 153490.3) | 55101.7 (29325.5 to 71793.5) | 64653.6 (39948.1 to 83775.8) | 9371.5 (8258.2 to 10896.4) | 3896.3 (3359.3 to 4608.9) | 5475.2 (4638.9 to 6554.1) | -92.2 (-94.1 to -87.3) | -92.9 (-94.6 to -85.3) | -91.5 (-93.8 to -84.3) |
|  |  |  | Rate | 12603.7 (8210.1 to 16154.1) | 12015.4 (6394.7 to 15655.2) | 13152.4 (8126.6 to 17042.5) | 1730 (1524.5 to 2011.6) | 1505.9 (1298.3 to 1781.3) | 1935 (1639.5 to 2316.4) | -86.3 (-89.6 to -77.8) | -87.5 (-90.4 to -74) | -85.3 (-89.3 to -72.8) |
| Khorasan-e-Razavi | All causes | DALYs (Disability-Adjusted Life Years) | Number | 1492904.8 (1335033.2 to 1653598) | 677370 (606017.2 to 752558.2) | 815534.8 (723181.6 to 902948.1) | 231076.2 (194855.6 to 271833.8) | 107624.4 (87921.1 to 129154.9) | 123451.9 (106147.5 to 143360.3) | -84.5 (-87.1 to -82) | -84.1 (-87.1 to -81.3) | -84.9 (-87.2 to -82.6) |
|  |  |  | Rate | 55142.3 (49311.1 to 61077.7) | 50657.8 (45321.6 to 56280.8) | 59518.5 (52778.5 to 65898) | 9992.7 (8426.4 to 11755.3) | 9532.1 (7787 to 11439) | 10432.2 (8969.9 to 12114.6) | -81.9 (-84.9 to -79) | -81.2 (-84.7 to -77.9) | -82.5 (-85.2 to -79.8) |
|  |  | YLDs (Years Lived with Disability) | Number | 150856.7 (111413.9 to 202089.3) | 76279.9 (55549.7 to 101537.9) | 74576.8 (55142.5 to 100632.3) | 116747.2 (83916.1 to 156385.6) | 61558 (44272 to 83883.2) | 55189.3 (39592 to 73969.8) | -22.6 (-28.7 to -15) | -19.3 (-26.5 to -11) | -26 (-33.6 to -16.3) |
|  |  |  | Rate | 5572.1 (4115.2 to 7464.4) | 5704.7 (4154.3 to 7593.6) | 5442.7 (4024.4 to 7344.2) | 5048.6 (3628.9 to 6762.8) | 5452.1 (3921.1 to 7429.4) | 4663.7 (3345.7 to 6250.8) | -9.4 (-16.5 to -0.5) | -4.4 (-12.9 to 5.4) | -14.3 (-23.1 to -3.1) |
|  |  | YLLs (Years of Life Lost) | Number | 1342048.1 (1181220.8 to 1489109.4) | 601090.1 (529270.2 to 673969) | 740958 (651636.4 to 826439.6) | 114329 (98299 to 131138.2) | 46066.4 (38463.4 to 54284.8) | 68262.6 (59324.2 to 77833.8) | -91.5 (-92.6 to -90.2) | -92.3 (-93.6 to -90.9) | -90.8 (-92 to -89.4) |
|  |  |  | Rate | 49570.2 (43629.8 to 55002.1) | 44953.1 (39582 to 50403.4) | 54075.8 (47557.1 to 60314.4) | 4944.1 (4250.9 to 5671) | 4080 (3406.6 to 4807.9) | 5768.5 (5013.2 to 6577.3) | -90 (-91.4 to -88.5) | -90.9 (-92.4 to -89.3) | -89.3 (-90.8 to -87.8) |
|  | Communicable, maternal, neonatal, and nutritional diseases | DALYs (Disability-Adjusted Life Years) | Number | 732186.9 (622339.5 to 886357.3) | 341453 (287010.7 to 436490.3) | 390733.9 (330314.9 to 476174.2) | 58681.6 (48114.1 to 70438.9) | 28475.7 (22804.3 to 34861.8) | 30205.9 (24569.1 to 36976) | -92 (-93.9 to -90.1) | -91.7 (-94 to -89.4) | -92.3 (-94.3 to -90.4) |
|  |  |  | Rate | 27044.2 (22986.9 to 32738.7) | 25535.9 (21464.4 to 32643.3) | 28516.1 (24106.7 to 34751.7) | 2537.6 (2080.7 to 3046.1) | 2522 (2019.7 to 3087.6) | 2552.5 (2076.2 to 3124.6) | -90.6 (-92.8 to -88.4) | -90.1 (-92.9 to -87.4) | -91 (-93.3 to -88.9) |
|  |  | YLDs (Years Lived with Disability) | Number | 38970.6 (27054.2 to 55531.9) | 18607.7 (12386.1 to 27195) | 20362.9 (13234.8 to 29496) | 22263.9 (15010 to 31572.2) | 11867.9 (7903.4 to 17153.2) | 10396 (7139.8 to 15387.2) | -42.9 (-53.6 to -27.9) | -36.2 (-50.9 to -16.4) | -48.9 (-62.2 to -27.7) |
|  |  |  | Rate | 1439.4 (999.3 to 2051.1) | 1391.6 (926.3 to 2033.8) | 1486.1 (965.9 to 2152.6) | 962.8 (649.1 to 1365.3) | 1051.1 (700 to 1519.2) | 878.5 (603.3 to 1300.3) | -33.1 (-45.6 to -15.6) | -24.5 (-41.8 to -1) | -40.9 (-56.3 to -16.3) |
|  |  | YLLs (Years of Life Lost) | Number | 693216.4 (584434.5 to 850744.7) | 322845.4 (267527.3 to 414184.4) | 370371 (309398.2 to 456927.6) | 36417.7 (28394.8 to 44943.1) | 16607.8 (12594.5 to 20929.4) | 19809.9 (15305.1 to 24504) | -94.7 (-96.2 to -93.3) | -94.9 (-96.6 to -93.2) | -94.7 (-96.3 to -93.2) |
|  |  |  | Rate | 25604.8 (21586.8 to 31423.3) | 24144.3 (20007.3 to 30975.2) | 27030 (22580.2 to 33347) | 1574.9 (1227.9 to 1943.5) | 1470.9 (1115.5 to 1853.7) | 1674 (1293.4 to 2070.7) | -93.8 (-95.5 to -92.2) | -93.9 (-95.9 to -91.9) | -93.8 (-95.7 to -92.2) |
|  | Injuries | DALYs (Disability-Adjusted Life Years) | Number | 277510.7 (238410.3 to 319001.3) | 105107.5 (86142.7 to 124086.8) | 172403.2 (148761.5 to 199920) | 39512.5 (35155.9 to 44306.8) | 12308.4 (10694.1 to 14098) | 27204.2 (23488.7 to 31126.7) | -85.8 (-88.1 to -82.6) | -88.3 (-90.6 to -84.6) | -84.2 (-87.4 to -81) |
|  |  |  | Rate | 10250.2 (8806 to 11782.7) | 7860.6 (6442.3 to 9280) | 12582.2 (10856.8 to 14590.4) | 1708.7 (1520.3 to 1916) | 1090.1 (947.2 to 1248.6) | 2298.9 (1984.9 to 2630.3) | -83.3 (-86.1 to -79.6) | -86.1 (-88.8 to -81.8) | -81.7 (-85.4 to -78) |
|  |  | YLDs (Years Lived with Disability) | Number | 10667.5 (7894.8 to 14035.4) | 4905 (3636.4 to 6482.4) | 5762.5 (4242.6 to 7646.6) | 3402.6 (2456.6 to 4535.5) | 1528.7 (1108.8 to 2035.3) | 1873.9 (1351 to 2523.9) | -68.1 (-70.6 to -65.4) | -68.8 (-71.7 to -65.5) | -67.5 (-70.2 to -64.5) |
|  |  |  | Rate | 394 (291.6 to 518.4) | 366.8 (272 to 484.8) | 420.6 (309.6 to 558.1) | 147.1 (106.2 to 196.1) | 135.4 (98.2 to 180.3) | 158.4 (114.2 to 213.3) | -62.7 (-65.6 to -59.5) | -63.1 (-66.5 to -59.2) | -62.3 (-65.5 to -58.9) |
|  |  | YLLs (Years of Life Lost) | Number | 266843.2 (227746.8 to 306678.1) | 100202.5 (81188.7 to 118633.3) | 166640.8 (143131.1 to 194792.3) | 36109.9 (31696.2 to 41254.1) | 10779.6 (9280.1 to 12513.2) | 25330.3 (21699.8 to 29375.6) | -86.5 (-88.8 to -83.3) | -89.2 (-91.5 to -85.6) | -84.8 (-88 to -81.5) |
|  |  |  | Rate | 9856.2 (8412.1 to 11327.5) | 7493.7 (6071.8 to 8872.1) | 12161.6 (10445.8 to 14216.1) | 1561.5 (1370.7 to 1784) | 954.7 (821.9 to 1108.3) | 2140.5 (1833.7 to 2482.4) | -84.2 (-86.9 to -80.4) | -87.3 (-89.9 to -82.9) | -82.4 (-86.1 to -78.6) |
|  | Non-communicable diseases | DALYs (Disability-Adjusted Life Years) | Number | 483207.2 (360244.8 to 576413.1) | 230809.5 (144899.3 to 278806.4) | 252397.6 (179025.5 to 310221.9) | 131813.3 (105042.7 to 163254.3) | 66510.4 (52484.8 to 83126.4) | 65302.9 (52605.7 to 80758.6) | -72.7 (-79 to -60.2) | -71.2 (-77.9 to -53) | -74.1 (-80.4 to -60.6) |
|  |  |  | Rate | 17847.8 (13306.1 to 21290.5) | 17261.3 (10836.4 to 20850.8) | 18420.2 (13065.5 to 22640.3) | 5700.2 (4542.5 to 7059.8) | 5890.7 (4648.5 to 7362.3) | 5518.4 (4445.4 to 6824.5) | -68.1 (-75.4 to -53.4) | -65.9 (-73.8 to -44.3) | -70 (-77.3 to -54.4) |
|  |  | YLDs (Years Lived with Disability) | Number | 101218.7 (74926.2 to 136682.7) | 52767.3 (38742.5 to 71007.2) | 48451.4 (36019 to 65527.8) | 91080.7 (65207.1 to 121756) | 48161.3 (34300.6 to 64397.2) | 42919.4 (30837.5 to 57336.9) | -10 (-16.9 to -2.5) | -8.7 (-15.7 to -0.6) | -11.4 (-19.1 to -3.2) |
|  |  |  | Rate | 3738.6 (2767.5 to 5048.5) | 3946.3 (2897.4 to 5310.3) | 3536 (2628.7 to 4782.3) | 3938.7 (2819.8 to 5265.2) | 4265.5 (3037.9 to 5703.5) | 3626.9 (2605.9 to 4845.2) | 5.4 (-2.7 to 14.2) | 8.1 (-0.1 to 17.7) | 2.6 (-6.4 to 12.1) |
|  |  | YLLs (Years of Life Lost) | Number | 381988.5 (257154.3 to 465653.2) | 178042.3 (91990 to 222440.3) | 203946.2 (131356 to 260062.3) | 40732.6 (33606.9 to 50817.7) | 18349 (14557 to 22797.9) | 22383.5 (18398.7 to 28567.9) | -89.3 (-92 to -81.7) | -89.7 (-92.3 to -78.1) | -89 (-91.9 to -80.2) |
|  |  |  | Rate | 14109.2 (9498.3 to 17199.5) | 13315.1 (6879.6 to 16635.4) | 14884.2 (9586.5 to 18979.6) | 1761.4 (1453.3 to 2197.6) | 1625.1 (1289.3 to 2019.2) | 1891.5 (1554.8 to 2414.1) | -87.5 (-90.6 to -78.6) | -87.8 (-90.9 to -74.1) | -87.3 (-90.6 to -77.1) |
| Khuzestan | All causes | DALYs (Disability-Adjusted Life Years) | Number | 687775.2 (619097.7 to 764526.1) | 289559.9 (261523.5 to 320334.1) | 398215.3 (358201.9 to 443514.1) | 180533.3 (152575.7 to 210246.2) | 84138.9 (70275.3 to 100575.7) | 96394.4 (82244.1 to 110721) | -73.8 (-78 to -69.6) | -70.9 (-75.8 to -66.3) | -75.8 (-79.4 to -71.8) |
|  |  |  | Rate | 36303.2 (32678.1 to 40354.4) | 31239.9 (28215.2 to 34560.1) | 41153.2 (37018 to 45834.5) | 10353.5 (8750.2 to 12057.5) | 9903.3 (8271.5 to 11837.9) | 10781.4 (9198.7 to 12383.8) | -71.5 (-76.1 to -67) | -68.3 (-73.6 to -63.2) | -73.8 (-77.7 to -69.5) |
|  |  | YLDs (Years Lived with Disability) | Number | 101300.3 (73878.5 to 135649.9) | 51194 (37337.8 to 68865.1) | 50106.3 (36000.6 to 66778.4) | 88056.6 (64392.1 to 119622.4) | 46596.6 (33397.4 to 63048.1) | 41460 (30349.3 to 55898.4) | -13.1 (-20.1 to -4.8) | -9 (-16.7 to 0.5) | -17.3 (-25.7 to -7.6) |
|  |  |  | Rate | 5347 (3899.6 to 7160.1) | 5523.2 (4028.3 to 7429.7) | 5178.2 (3720.4 to 6901.1) | 5050 (3692.9 to 6860.3) | 5484.5 (3930.9 to 7420.9) | 4637.2 (3394.5 to 6252) | -5.6 (-13.2 to 3.4) | -0.7 (-9.2 to 9.7) | -10.4 (-19.6 to 0) |
|  |  | YLLs (Years of Life Lost) | Number | 586474.9 (522611.4 to 650372) | 238365.8 (213222.8 to 265113) | 348109 (308607 to 386040.6) | 92476.7 (84115.5 to 101254.4) | 37542.3 (34087.5 to 41526.5) | 54934.4 (49226.7 to 60571.4) | -84.2 (-86.5 to -81.6) | -84.3 (-86.5 to -81.6) | -84.2 (-86.5 to -81.5) |
|  |  |  | Rate | 30956.2 (27585.2 to 34328.9) | 25716.7 (23004.1 to 28602.4) | 35975 (31892.7 to 39895) | 5303.5 (4824 to 5806.9) | 4418.8 (4012.2 to 4887.7) | 6144.2 (5505.8 to 6774.7) | -82.9 (-85.3 to -80.1) | -82.8 (-85.2 to -79.9) | -82.9 (-85.4 to -79.9) |
|  | Communicable, maternal, neonatal, and nutritional diseases | DALYs (Disability-Adjusted Life Years) | Number | 313333.5 (266997.1 to 381176.8) | 134303.6 (112606.2 to 165308.9) | 179030 (151530.5 to 218094.9) | 45846.2 (38749.5 to 54425.1) | 22307.6 (18481 to 27368.1) | 23538.6 (19875.6 to 28021.6) | -85.4 (-88.3 to -81.6) | -83.4 (-87.3 to -78.7) | -86.9 (-90 to -83.3) |
|  |  |  | Rate | 16538.8 (14093 to 20119.8) | 14489.7 (12148.8 to 17834.8) | 18501.7 (15659.8 to 22538.8) | 2629.3 (2222.3 to 3121.3) | 2625.6 (2175.2 to 3221.3) | 2632.7 (2223 to 3134.1) | -84.1 (-87.3 to -80) | -81.9 (-86.2 to -76.7) | -85.8 (-89.1 to -81.9) |
|  |  | YLDs (Years Lived with Disability) | Number | 26340.6 (18168.1 to 36814.8) | 13295.3 (8811.3 to 19303.5) | 13045.3 (8451.3 to 19572.4) | 17513.2 (12047.6 to 24692.8) | 9670.8 (6464.5 to 14238.5) | 7842.4 (5264.3 to 11451.3) | -33.5 (-47.9 to -12.7) | -27.3 (-44.5 to -5.2) | -39.9 (-56.8 to -15.3) |
|  |  |  | Rate | 1390.3 (959 to 1943.2) | 1434.4 (950.6 to 2082.6) | 1348.2 (873.4 to 2022.7) | 1004.4 (690.9 to 1416.1) | 1138.3 (760.9 to 1675.9) | 877.1 (588.8 to 1280.8) | -27.8 (-43.3 to -5.1) | -20.6 (-39.4 to 3.4) | -34.9 (-53.3 to -8.4) |
|  |  | YLLs (Years of Life Lost) | Number | 286993 (239282.6 to 349932) | 121008.3 (100903.6 to 150753.4) | 165984.7 (139237.5 to 203503.5) | 28333 (23771.4 to 33482) | 12636.8 (10448.4 to 14954.6) | 15696.2 (12963.7 to 18571.7) | -90.1 (-92.6 to -87.2) | -89.6 (-92.4 to -86.3) | -90.5 (-93 to -87.6) |
|  |  |  | Rate | 15148.5 (12630.2 to 18470.6) | 13055.3 (10886.3 to 16264.4) | 17153.5 (14389.4 to 21030.9) | 1624.9 (1363.3 to 1920.2) | 1487.4 (1229.8 to 1760.2) | 1755.6 (1449.9 to 2077.2) | -89.3 (-91.9 to -86.1) | -88.6 (-91.8 to -85.1) | -89.8 (-92.4 to -86.6) |
|  | Injuries | DALYs (Disability-Adjusted Life Years) | Number | 124502.7 (106643.8 to 141687.6) | 41738.7 (33680.7 to 51201.2) | 82764 (71252.3 to 94641.5) | 32887.9 (29848 to 36319.1) | 10684.7 (9497.9 to 11971.1) | 22203.2 (19762.2 to 24918.9) | -73.6 (-77.9 to -68.3) | -74.4 (-79.4 to -66.8) | -73.2 (-77.6 to -67.8) |
|  |  |  | Rate | 6571.7 (5629 to 7478.8) | 4503.1 (3633.7 to 5524) | 8553.2 (7363.5 to 9780.6) | 1886.1 (1711.8 to 2082.9) | 1257.6 (1117.9 to 1409) | 2483.4 (2210.3 to 2787.1) | -71.3 (-76 to -65.5) | -72.1 (-77.5 to -63.8) | -71 (-75.7 to -65.2) |
|  |  | YLDs (Years Lived with Disability) | Number | 6935.7 (5211.9 to 9133.1) | 2811.6 (2100.4 to 3708.2) | 4124 (3083.9 to 5437.3) | 2507.7 (1836.5 to 3387.2) | 1128.2 (831.3 to 1517.7) | 1379.4 (998.3 to 1873.7) | -63.8 (-67.8 to -60.4) | -59.9 (-64 to -55.9) | -66.6 (-70.6 to -62.9) |
|  |  |  | Rate | 366.1 (275.1 to 482.1) | 303.3 (226.6 to 400.1) | 426.2 (318.7 to 561.9) | 143.8 (105.3 to 194.3) | 132.8 (97.8 to 178.6) | 154.3 (111.7 to 209.6) | -60.7 (-65 to -57) | -56.2 (-60.7 to -51.9) | -63.8 (-68.2 to -59.8) |
|  |  | YLLs (Years of Life Lost) | Number | 117567 (99759.6 to 134661.3) | 38927.1 (31141.7 to 47727.1) | 78640 (67026.9 to 90785.1) | 30380.2 (27529.1 to 33682.1) | 9556.5 (8370.6 to 10782.3) | 20823.8 (18405.3 to 23530.3) | -74.2 (-78.5 to -68.6) | -75.5 (-80.5 to -67.6) | -73.5 (-78.2 to -67.8) |
|  |  |  | Rate | 6205.6 (5265.7 to 7107.9) | 4199.7 (3359.8 to 5149.2) | 8127 (6926.8 to 9382.1) | 1742.3 (1578.8 to 1931.7) | 1124.8 (985.2 to 1269.1) | 2329.1 (2058.6 to 2631.8) | -71.9 (-76.6 to -65.8) | -73.2 (-78.7 to -64.6) | -71.3 (-76.4 to -65.1) |
|  | Non-communicable diseases | DALYs (Disability-Adjusted Life Years) | Number | 249938.9 (202396.1 to 290533.2) | 113517.6 (83805.4 to 133376.9) | 136421.3 (104360.2 to 159924) | 100974.6 (81074.2 to 125292.2) | 50897.2 (40686.3 to 64077.8) | 50077.4 (40418 to 61352.3) | -59.6 (-67.5 to -48.4) | -55.2 (-64 to -37.2) | -63.3 (-70.8 to -49.4) |
|  |  |  | Rate | 13192.6 (10683.2 to 15335.4) | 12247.2 (9041.6 to 14389.7) | 14098.3 (10785 to 16527.2) | 5790.9 (4649.6 to 7185.5) | 5990.7 (4788.8 to 7542) | 5601 (4520.6 to 6862) | -56.1 (-64.6 to -44) | -51.1 (-60.7 to -31.5) | -60.3 (-68.4 to -45.3) |
|  |  | YLDs (Years Lived with Disability) | Number | 68024.1 (48991 to 90926) | 35087.1 (25315.5 to 46961.7) | 32937 (23668.8 to 44067.4) | 68035.7 (49294.7 to 91642.3) | 35797.5 (25965.8 to 48867.1) | 32238.1 (23331.4 to 43421) | 0 (-8.4 to 8.7) | 2 (-6.3 to 11.2) | -2.1 (-11.1 to 7.1) |
|  |  |  | Rate | 3590.5 (2585.9 to 4799.4) | 3785.5 (2731.2 to 5066.6) | 3403.8 (2446 to 4554.1) | 3901.8 (2827 to 5255.7) | 4213.4 (3056.2 to 5751.7) | 3605.7 (2609.5 to 4856.5) | 8.7 (-0.5 to 18.1) | 11.3 (2.2 to 21.3) | 5.9 (-3.7 to 15.9) |
|  |  | YLLs (Years of Life Lost) | Number | 181914.8 (136131.5 to 215845.1) | 78430.5 (49494.5 to 95120.8) | 103484.4 (72400 to 123743.9) | 32938.9 (28399 to 38255.3) | 15099.7 (12925.2 to 17485.8) | 17839.2 (15240.9 to 21246.1) | -81.9 (-85.8 to -73.7) | -80.7 (-85.1 to -68.1) | -82.8 (-86.8 to -72.6) |
|  |  |  | Rate | 9602.1 (7185.5 to 11393.1) | 8461.7 (5339.8 to 10262.4) | 10694.5 (7482.1 to 12788.2) | 1889 (1628.7 to 2193.9) | 1777.3 (1521.3 to 2058.1) | 1995.3 (1704.6 to 2376.3) | -80.3 (-84.6 to -71.4) | -79 (-83.7 to -65.2) | -81.3 (-85.7 to -70.3) |
| Kohgiluyeh and Boyer-Ahmad | All causes | DALYs (Disability-Adjusted Life Years) | Number | 150296.3 (135401.4 to 166079.8) | 67847 (60722.8 to 75530) | 82449.3 (73781.9 to 91215.6) | 25017.6 (21376.7 to 29185.1) | 11819.9 (9974.2 to 14018) | 13197.7 (11365.7 to 15211.3) | -83.4 (-86 to -80.6) | -82.6 (-85.7 to -79.5) | -84 (-86.4 to -81.4) |
|  |  |  | Rate | 51185.2 (46112.5 to 56560.4) | 46997.8 (42062.9 to 52319.9) | 55234.8 (49428.3 to 61107.6) | 10248.8 (8757.2 to 11956) | 9977 (8419.1 to 11832.4) | 10505 (9046.8 to 12107.7) | -80 (-83.1 to -76.7) | -78.8 (-82.5 to -75) | -81 (-83.9 to -77.9) |
|  |  | YLDs (Years Lived with Disability) | Number | 16248 (12040.4 to 21593.1) | 8300 (6089.3 to 10994.9) | 7948 (5903.3 to 10647.7) | 12019.9 (8741 to 16199.4) | 6341.4 (4604.3 to 8549.1) | 5678.5 (4122.8 to 7541.2) | -26 (-32.5 to -18.3) | -23.6 (-30.1 to -14.4) | -28.6 (-36.3 to -19.5) |
|  |  |  | Rate | 5533.4 (4100.5 to 7353.8) | 5749.5 (4218.1 to 7616.2) | 5324.5 (3954.7 to 7133.1) | 4924.1 (3580.9 to 6636.3) | 5352.7 (3886.4 to 7216.1) | 4519.9 (3281.7 to 6002.5) | -11 (-18.8 to -1.7) | -6.9 (-14.8 to 4.3) | -15.1 (-24.3 to -4.3) |
|  |  | YLLs (Years of Life Lost) | Number | 134048.2 (118937.7 to 149816.9) | 59546.9 (52833.2 to 66998.5) | 74501.3 (65886.3 to 82686.6) | 12997.8 (11815.2 to 14176.4) | 5478.6 (4943.3 to 6026.7) | 7519.2 (6738.3 to 8317.3) | -90.3 (-91.6 to -88.6) | -90.8 (-92.2 to -89.3) | -89.9 (-91.3 to -88) |
|  |  |  | Rate | 45651.7 (40505.6 to 51021.9) | 41248.4 (36597.7 to 46410.1) | 49910.3 (44138.8 to 55393.8) | 5324.7 (4840.2 to 5807.5) | 4624.4 (4172.6 to 5087.1) | 5985.1 (5363.5 to 6620.3) | -88.3 (-89.9 to -86.3) | -88.8 (-90.5 to -86.9) | -88 (-89.7 to -85.8) |
|  | Communicable, maternal, neonatal, and nutritional diseases | DALYs (Disability-Adjusted Life Years) | Number | 64877.3 (54920.9 to 79265.6) | 29996.4 (24798.4 to 38412.6) | 34880.9 (29015.9 to 42884.3) | 5891.2 (4952.8 to 6953) | 2926.7 (2405.2 to 3552.4) | 2964.5 (2456.7 to 3609.6) | -90.9 (-93 to -88.7) | -90.2 (-93 to -87.3) | -91.5 (-93.7 to -89.2) |
|  |  |  | Rate | 22094.7 (18704 to 26994.8) | 20778.6 (17177.9 to 26608.6) | 23367.6 (19438.4 to 28729.3) | 2413.4 (2029 to 2848.4) | 2470.4 (2030.2 to 2998.5) | 2359.7 (1955.5 to 2873.1) | -89.1 (-91.6 to -86.4) | -88.1 (-91.4 to -84.5) | -89.9 (-92.5 to -87.1) |
|  |  | YLDs (Years Lived with Disability) | Number | 4238.6 (2977.9 to 6016.8) | 2157.4 (1445.2 to 3069.6) | 2081.2 (1378.1 to 3128) | 2373.2 (1626.8 to 3340.3) | 1326 (886.5 to 1952.8) | 1047.2 (704.7 to 1548.3) | -44 (-55 to -25.3) | -38.5 (-53 to -17.4) | -49.7 (-63.9 to -26.5) |
|  |  |  | Rate | 1443.5 (1014.2 to 2049.1) | 1494.4 (1001.1 to 2126.3) | 1394.2 (923.2 to 2095.5) | 972.2 (666.4 to 1368.4) | 1119.2 (748.3 to 1648.4) | 833.6 (560.9 to 1232.4) | -32.6 (-45.9 to -10.2) | -25.1 (-42.8 to 0.7) | -40.2 (-57.1 to -12.6) |
|  |  | YLLs (Years of Life Lost) | Number | 60638.7 (51070.3 to 74582.6) | 27839 (22620.4 to 36396.2) | 32799.7 (27104.9 to 40517.2) | 3518 (2928.5 to 4180.4) | 1600.7 (1313 to 1931.1) | 1917.3 (1561.6 to 2290.5) | -94.2 (-95.7 to -92.7) | -94.3 (-96.1 to -92.7) | -94.2 (-95.9 to -92.4) |
|  |  |  | Rate | 20651.2 (17392.6 to 25400) | 19284.1 (15669.2 to 25211.8) | 21973.3 (18158.2 to 27143.5) | 1441.2 (1199.7 to 1712.5) | 1351.2 (1108.3 to 1630) | 1526.1 (1243 to 1823.2) | -93 (-94.8 to -91.2) | -93 (-95.3 to -91) | -93.1 (-95.1 to -91) |
|  | Injuries | DALYs (Disability-Adjusted Life Years) | Number | 32044.7 (27490.7 to 37363) | 12827.3 (10698.8 to 15461.4) | 19217.4 (16407.1 to 22493.4) | 5253.4 (4781 to 5770.6) | 1929.8 (1726.6 to 2120.1) | 3323.6 (2893.8 to 3786.8) | -83.6 (-86.4 to -80.4) | -85 (-88 to -81.9) | -82.7 (-86.4 to -78.6) |
|  |  |  | Rate | 10913.2 (9362.3 to 12724.4) | 8885.5 (7411.1 to 10710.2) | 12874.2 (10991.5 to 15068.9) | 2152.1 (1958.6 to 2364) | 1628.9 (1457.4 to 1789.5) | 2645.5 (2303.4 to 3014.2) | -80.3 (-83.6 to -76.4) | -81.7 (-85.4 to -77.9) | -79.5 (-83.8 to -74.6) |
|  |  | YLDs (Years Lived with Disability) | Number | 1216.4 (895.1 to 1610.4) | 540.2 (397 to 713.5) | 676.2 (496.2 to 897.3) | 382.8 (275.4 to 514.8) | 178.7 (127.7 to 241.6) | 204.1 (147.1 to 278.6) | -68.5 (-71.5 to -65.8) | -66.9 (-70.2 to -63.5) | -69.8 (-73.1 to -67) |
|  |  |  | Rate | 414.2 (304.8 to 548.5) | 374.2 (275 to 494.2) | 453 (332.4 to 601.1) | 156.8 (112.8 to 210.9) | 150.8 (107.8 to 203.9) | 162.4 (117.1 to 221.8) | -62.1 (-65.7 to -58.9) | -59.7 (-63.7 to -55.6) | -64.1 (-68 to -60.8) |
|  |  | YLLs (Years of Life Lost) | Number | 30828.3 (26397.7 to 36037) | 12287.1 (10209.5 to 14897) | 18541.2 (15779.9 to 21836.5) | 4870.7 (4380.2 to 5391) | 1751.1 (1574 to 1935.8) | 3119.6 (2695.5 to 3578.8) | -84.2 (-87 to -80.9) | -85.7 (-88.6 to -82.6) | -83.2 (-86.9 to -78.9) |
|  |  |  | Rate | 10498.9 (8990.1 to 12272.8) | 8511.3 (7072.1 to 10319.2) | 12421.2 (10571.3 to 14628.8) | 1995.3 (1794.4 to 2208.5) | 1478.1 (1328.6 to 1634) | 2483.1 (2145.6 to 2848.6) | -81 (-84.3 to -77) | -82.6 (-86.2 to -78.8) | -80 (-84.4 to -75) |
|  | Non-communicable diseases | DALYs (Disability-Adjusted Life Years) | Number | 53374.3 (41446.5 to 63210.9) | 25023.3 (17911.6 to 29694) | 28351 (21081.4 to 34971.7) | 13803.1 (11067.2 to 17003.7) | 6942.7 (5464.8 to 8684.6) | 6860.5 (5503.5 to 8363) | -74.1 (-79.9 to -64.5) | -72.3 (-78.7 to -59.9) | -75.8 (-81.4 to -63.7) |
|  |  |  | Rate | 18177.2 (14115.1 to 21527.2) | 17333.7 (12407.4 to 20569.1) | 18993.1 (14123 to 23428.4) | 5654.6 (4533.8 to 6965.7) | 5860.2 (4612.8 to 7330.6) | 5460.7 (4380.6 to 6656.7) | -68.9 (-75.8 to -57.3) | -66.2 (-74 to -51.2) | -71.2 (-77.9 to -56.8) |
|  |  | YLDs (Years Lived with Disability) | Number | 10793.1 (7879.1 to 14576.1) | 5602.4 (4097.8 to 7516.9) | 5190.6 (3809.7 to 6967.7) | 9263.9 (6591.5 to 12433.4) | 4836.7 (3432.6 to 6549.6) | 4427.2 (3139.7 to 5875.5) | -14.2 (-20.6 to -5.3) | -13.7 (-20.4 to -4.3) | -14.7 (-22.5 to -5.3) |
|  |  |  | Rate | 3675.7 (2683.3 to 4964.1) | 3880.8 (2838.5 to 5207) | 3477.3 (2552.2 to 4667.8) | 3795.1 (2700.3 to 5093.5) | 4082.6 (2897.4 to 5528.4) | 3523.9 (2499.1 to 4676.7) | 3.2 (-4.5 to 13.9) | 5.2 (-3 to 16.6) | 1.3 (-7.9 to 12.5) |
|  |  | YLLs (Years of Life Lost) | Number | 42581.3 (30902.2 to 51661.1) | 19420.8 (12245.1 to 23995.3) | 23160.4 (15822.7 to 29426.1) | 4539.2 (3956.3 to 5243.3) | 2106 (1827.7 to 2409) | 2433.2 (2003.8 to 2961.2) | -89.3 (-91.7 to -83.9) | -89.2 (-91.7 to -81.6) | -89.5 (-92 to -82.4) |
|  |  |  | Rate | 14501.5 (10524.1 to 17593.8) | 13452.9 (8482.3 to 16621.6) | 15515.7 (10600 to 19713.3) | 1859.5 (1620.7 to 2148) | 1777.6 (1542.7 to 2033.4) | 1936.8 (1595 to 2357) | -87.2 (-90.1 to -80.6) | -86.8 (-89.8 to -77.6) | -87.5 (-90.5 to -79) |
| Kurdistan | All causes | DALYs (Disability-Adjusted Life Years) | Number | 484962.9 (432237 to 538103.1) | 223177.2 (197210.2 to 247372.6) | 261785.7 (231787.5 to 290316.3) | 49163.5 (41805.1 to 56956.4) | 22325.2 (18387.2 to 26477.7) | 26838.3 (23410.5 to 30640) | -89.9 (-91.5 to -88.1) | -90 (-91.8 to -88) | -89.7 (-91.3 to -88) |
|  |  |  | Rate | 67558 (60213 to 74960.8) | 63438.5 (56057.4 to 70316.1) | 71517.3 (63322.1 to 79311.5) | 9999.3 (8502.7 to 11584.3) | 9287.3 (7649.1 to 11014.8) | 10680.4 (9316.3 to 12193.3) | -85.2 (-87.6 to -82.6) | -85.4 (-88 to -82.5) | -85.1 (-87.3 to -82.5) |
|  |  | YLDs (Years Lived with Disability) | Number | 40170.7 (29299.4 to 53133) | 20420.1 (14724.1 to 27056.9) | 19750.6 (14210.4 to 26509.8) | 24556.9 (17951 to 32657.2) | 13017.6 (9413.4 to 17283.4) | 11539.3 (8420.6 to 15478.6) | -38.9 (-44.2 to -33.2) | -36.3 (-42 to -30.6) | -41.6 (-47.7 to -34.7) |
|  |  |  | Rate | 5596 (4081.6 to 7401.7) | 5804.4 (4185.4 to 7691) | 5395.7 (3882.1 to 7242.2) | 4994.6 (3651 to 6642.1) | 5415.3 (3916 to 7189.9) | 4592.1 (3351 to 6159.8) | -10.7 (-18.5 to -2.5) | -6.7 (-15.1 to 1.6) | -14.9 (-23.8 to -4.9) |
|  |  | YLLs (Years of Life Lost) | Number | 444792.2 (392697.2 to 495947.7) | 202757.1 (179028.6 to 227940.2) | 242035.1 (213361.9 to 268220.1) | 24606.6 (22568.6 to 26936.8) | 9307.6 (8433.1 to 10283.5) | 15299 (14015.9 to 16756.6) | -94.5 (-95.2 to -93.5) | -95.4 (-96.1 to -94.6) | -93.7 (-94.5 to -92.7) |
|  |  |  | Rate | 61962 (54704.9 to 69088.3) | 57634.1 (50889.2 to 64792.4) | 66121.6 (58288.4 to 73275.1) | 5004.7 (4590.2 to 5478.6) | 3872 (3508.2 to 4278) | 6088.3 (5577.7 to 6668.3) | -91.9 (-93 to -90.5) | -93.3 (-94.3 to -92.1) | -90.8 (-92 to -89.3) |
|  | Communicable, maternal, neonatal, and nutritional diseases | DALYs (Disability-Adjusted Life Years) | Number | 234736 (196404.2 to 290976.2) | 109500 (89766.4 to 144400.7) | 125236 (105275.3 to 154562.4) | 13154.1 (11330 to 15368.2) | 6354.1 (5359.8 to 7641.3) | 6800 (5822.4 to 7989.5) | -94.4 (-95.7 to -93) | -94.2 (-96 to -92.6) | -94.6 (-95.9 to -93.1) |
|  |  |  | Rate | 32700 (27360.2 to 40534.6) | 31125.6 (25516.3 to 41046.2) | 34213.2 (28760.2 to 42224.9) | 2675.4 (2304.4 to 3125.7) | 2643.3 (2229.7 to 3178.8) | 2706.1 (2317 to 3179.5) | -91.8 (-93.8 to -89.8) | -91.5 (-94.2 to -89.2) | -92.1 (-94 to -90) |
|  |  | YLDs (Years Lived with Disability) | Number | 9918 (6707.2 to 13877.8) | 4880.8 (3126.4 to 6928.8) | 5037.2 (3177.5 to 7613.9) | 4656.8 (3220.2 to 6575.4) | 2529 (1668.5 to 3704.7) | 2127.8 (1473.2 to 3060.8) | -53 (-63.3 to -40.5) | -48.2 (-60.5 to -31.1) | -57.8 (-69.8 to -41.1) |
|  |  |  | Rate | 1381.6 (934.3 to 1933.3) | 1387.4 (888.7 to 1969.5) | 1376.1 (868.1 to 2080.1) | 947.1 (654.9 to 1337.4) | 1052.1 (694.1 to 1541.2) | 846.8 (586.3 to 1218) | -31.4 (-46.4 to -13.1) | -24.2 (-42.2 to 0.8) | -38.5 (-56 to -14.2) |
|  |  | YLLs (Years of Life Lost) | Number | 224818 (186999.3 to 280100.5) | 104619.2 (85635 to 139499.9) | 120198.8 (99712.3 to 149775.7) | 8497.3 (7344.6 to 9791.3) | 3825.1 (3206.8 to 4440.3) | 4672.2 (3975.5 to 5444.1) | -96.2 (-97.2 to -95.3) | -96.3 (-97.6 to -95.3) | -96.1 (-97.2 to -95.1) |
|  |  |  | Rate | 31318.4 (26050 to 39019.6) | 29738.2 (24341.9 to 39653.1) | 32837.1 (27240.4 to 40917.2) | 1728.3 (1493.8 to 1991.4) | 1591.3 (1334 to 1847.2) | 1859.3 (1582.1 to 2166.5) | -94.5 (-95.9 to -93.1) | -94.6 (-96.5 to -93.1) | -94.3 (-95.9 to -92.8) |
|  | Injuries | DALYs (Disability-Adjusted Life Years) | Number | 96436.3 (82541.7 to 113118.5) | 39238.2 (31269.7 to 47705.5) | 57198 (48135.2 to 69193.9) | 8671.8 (7819.1 to 9651.5) | 2366.5 (2111.2 to 2601.8) | 6305.3 (5547 to 7155) | -91 (-92.7 to -89) | -94 (-95.3 to -92.2) | -89 (-91.5 to -86.5) |
|  |  |  | Rate | 13434.1 (11498.5 to 15758) | 11153.5 (8888.5 to 13560.4) | 15625.9 (13150.1 to 18903.1) | 1763.8 (1590.3 to 1963) | 984.5 (878.2 to 1082.3) | 2509.2 (2207.4 to 2847.3) | -86.9 (-89.4 to -84) | -91.2 (-93.2 to -88.6) | -83.9 (-87.6 to -80.3) |
|  |  | YLDs (Years Lived with Disability) | Number | 3242.7 (2394.1 to 4247.8) | 1502.8 (1091.9 to 1995.1) | 1739.9 (1300.5 to 2293.8) | 713.1 (515.5 to 953.3) | 305.7 (221.1 to 409.3) | 407.3 (293.1 to 548) | -78 (-79.9 to -76) | -79.7 (-81.6 to -77.3) | -76.6 (-78.9 to -74.5) |
|  |  |  | Rate | 451.7 (333.5 to 591.7) | 427.2 (310.4 to 567.1) | 475.3 (355.3 to 626.6) | 145 (104.8 to 193.9) | 127.2 (92 to 170.3) | 162.1 (116.6 to 218.1) | -67.9 (-70.6 to -65) | -70.2 (-73.1 to -66.8) | -65.9 (-69.2 to -62.8) |
|  |  | YLLs (Years of Life Lost) | Number | 93193.6 (79270.2 to 110188.1) | 37735.4 (29818.9 to 46188.1) | 55458.2 (46178.9 to 67501.4) | 7958.7 (7111.3 to 8873.6) | 2060.8 (1832.8 to 2285.6) | 5898 (5134.3 to 6737.7) | -91.5 (-93.1 to -89.5) | -94.5 (-95.9 to -92.9) | -89.4 (-91.9 to -86.9) |
|  |  |  | Rate | 12982.4 (11042.8 to 15349.8) | 10726.4 (8476.1 to 13129.1) | 15150.6 (12615.6 to 18440.7) | 1618.7 (1446.4 to 1804.8) | 857.3 (762.5 to 950.8) | 2347.1 (2043.2 to 2681.3) | -87.5 (-90 to -84.7) | -92 (-94 to -89.6) | -84.5 (-88.2 to -80.9) |
|  | Non-communicable diseases | DALYs (Disability-Adjusted Life Years) | Number | 153790.6 (105295 to 190918.5) | 74438.9 (43893.9 to 95332.2) | 79351.7 (50119.2 to 99026.8) | 27154.2 (21463.3 to 33579.1) | 13547.2 (10506.4 to 17211.9) | 13607 (10878.6 to 16443.9) | -82.3 (-86.7 to -72.1) | -81.8 (-86.4 to -68.3) | -82.9 (-87.2 to -71.4) |
|  |  |  | Rate | 21423.9 (14668.2 to 26596) | 21159.4 (12476.9 to 27098.4) | 21678.1 (13692.1 to 27053.1) | 5522.9 (4365.4 to 6829.6) | 5635.7 (4370.7 to 7160.2) | 5415 (4329.2 to 6543.9) | -74.2 (-80.6 to -59.3) | -73.4 (-80.1 to -53.6) | -75 (-81.3 to -58.3) |
|  |  | YLDs (Years Lived with Disability) | Number | 27010 (19604.3 to 36057.6) | 14036.5 (10074.8 to 18763.3) | 12973.5 (9443.6 to 17205.1) | 19187 (13831.5 to 25823.1) | 10182.9 (7246.4 to 13794) | 9004.2 (6507 to 11999.8) | -29 (-34.7 to -23.2) | -27.5 (-33.4 to -21.4) | -30.6 (-36.9 to -24.2) |
|  |  |  | Rate | 3762.6 (2731 to 5023) | 3989.9 (2863.8 to 5333.5) | 3544.2 (2579.9 to 4700.3) | 3902.4 (2813.2 to 5252.1) | 4236.1 (3014.5 to 5738.3) | 3583.2 (2589.5 to 4775.4) | 3.7 (-4.6 to 12.2) | 6.2 (-2.6 to 15) | 1.1 (-8.1 to 10.4) |
|  |  | YLLs (Years of Life Lost) | Number | 126780.6 (76886.1 to 161424.7) | 60402.4 (30669.1 to 80084.2) | 66378.1 (37323.1 to 86084.9) | 7967.2 (6962.1 to 9285.4) | 3364.4 (2896.2 to 3985.5) | 4602.8 (3947 to 5499.8) | -93.7 (-95.2 to -88.7) | -94.4 (-95.8 to -87.8) | -93.1 (-94.8 to -86.2) |
|  |  |  | Rate | 17661.2 (10710.7 to 22487.4) | 17169.5 (8717.7 to 22764.1) | 18133.8 (10196.3 to 23517.5) | 1620.4 (1416 to 1888.6) | 1399.6 (1204.8 to 1658) | 1831.7 (1570.7 to 2188.7) | -90.8 (-93.1 to -83.5) | -91.8 (-93.9 to -82.2) | -89.9 (-92.5 to -80) |
| Lorestan | All causes | DALYs (Disability-Adjusted Life Years) | Number | 413275.3 (368310.5 to 467152.2) | 181887.7 (160081.6 to 207361.2) | 231387.7 (206558.6 to 262255.1) | 41767.1 (33967.1 to 50414.5) | 19602.1 (15498.9 to 24456.9) | 22165 (18534 to 26383.1) | -89.9 (-91.8 to -87.8) | -89.2 (-91.5 to -86.8) | -90.4 (-92.1 to -88.6) |
|  |  |  | Rate | 45480.6 (40532.3 to 51409.7) | 41011.6 (36094.8 to 46755.3) | 49741.3 (44403.8 to 56376.9) | 7675.9 (6242.5 to 9265.2) | 7435.2 (5878.8 to 9276.7) | 7902.2 (6607.7 to 9406) | -83.1 (-86.3 to -79.6) | -81.9 (-85.7 to -77.8) | -84.1 (-86.9 to -81.1) |
|  |  | YLDs (Years Lived with Disability) | Number | 48808.5 (35446.7 to 65228) | 24458.7 (17639.5 to 32957) | 24349.8 (17709.6 to 32298.3) | 26191.1 (18814.9 to 35030.9) | 13837.4 (9898.1 to 18667.9) | 12353.7 (9024.2 to 16485.1) | -46.3 (-50.5 to -41.3) | -43.4 (-48.7 to -37.8) | -49.3 (-54.5 to -43) |
|  |  |  | Rate | 5371.3 (3900.9 to 7178.3) | 5514.9 (3977.3 to 7431.1) | 5234.5 (3807 to 6943.2) | 4813.4 (3457.8 to 6438) | 5248.6 (3754.4 to 7080.9) | 4404.3 (3217.3 to 5877.2) | -10.4 (-17.4 to -2) | -4.8 (-13.7 to 4.7) | -15.9 (-24.5 to -5.5) |
|  |  | YLLs (Years of Life Lost) | Number | 364466.8 (322052 to 415249.2) | 157429 (137528.5 to 180553.3) | 207037.8 (182613.7 to 236672.4) | 15576 (13803.4 to 17434.5) | 5764.7 (5083.2 to 6541.5) | 9811.3 (8549.3 to 11118.7) | -95.7 (-96.5 to -94.8) | -96.3 (-97 to -95.5) | -95.3 (-96.1 to -94.3) |
|  |  |  | Rate | 40109.3 (35441.5 to 45697.8) | 35496.7 (31009.6 to 40710.7) | 44506.9 (39256.4 to 50877.4) | 2862.6 (2536.8 to 3204.1) | 2186.6 (1928.1 to 2481.2) | 3497.9 (3048 to 3964) | -92.9 (-94.1 to -91.4) | -93.8 (-94.9 to -92.5) | -92.1 (-93.5 to -90.5) |
|  | Communicable, maternal, neonatal, and nutritional diseases | DALYs (Disability-Adjusted Life Years) | Number | 214022.6 (179089.3 to 265014.5) | 94064.3 (77932.8 to 121102.1) | 119958.3 (99447.5 to 147410.2) | 10170.4 (8265 to 12522.5) | 4949.6 (3896.3 to 6303.9) | 5220.8 (4315.8 to 6404.6) | -95.2 (-96.4 to -93.8) | -94.7 (-96.3 to -93) | -95.6 (-96.8 to -94.3) |
|  |  |  | Rate | 23553 (19708.6 to 29164.6) | 21209.4 (17572.1 to 27305.8) | 25787.4 (21378.2 to 31688.7) | 1869.1 (1518.9 to 2301.4) | 1877.4 (1477.9 to 2391.1) | 1861.3 (1538.6 to 2283.3) | -92.1 (-94 to -89.7) | -91.1 (-93.7 to -88.2) | -92.8 (-94.7 to -90.6) |
|  |  | YLDs (Years Lived with Disability) | Number | 12685.2 (8744.5 to 17508.3) | 6108.5 (4073.6 to 8744.3) | 6576.7 (4231.4 to 9549.4) | 5033.7 (3408.5 to 7352.8) | 2778.5 (1833.5 to 4112.9) | 2255.2 (1503.6 to 3398.4) | -60.3 (-67.9 to -46.9) | -54.5 (-65.2 to -38.6) | -65.7 (-75.1 to -50.8) |
|  |  |  | Rate | 1396 (962.3 to 1926.8) | 1377.3 (918.5 to 1971.6) | 1413.8 (909.6 to 2052.8) | 925.1 (626.4 to 1351.3) | 1053.9 (695.5 to 1560.1) | 804 (536.1 to 1211.6) | -33.7 (-46.4 to -11.3) | -23.5 (-41.5 to 3.2) | -43.1 (-58.7 to -18.4) |
|  |  | YLLs (Years of Life Lost) | Number | 201337.3 (166257.2 to 252576.8) | 87955.8 (71820.1 to 114920.6) | 113381.6 (92327.3 to 140278.3) | 5136.6 (4287.6 to 6088) | 2171 (1771.1 to 2591.4) | 2965.6 (2458.4 to 3532.5) | -97.4 (-98.2 to -96.7) | -97.5 (-98.4 to -96.7) | -97.4 (-98.1 to -96.6) |
|  |  |  | Rate | 22157 (18296.5 to 27795.9) | 19832.1 (16193.8 to 25912) | 24373.6 (19847.6 to 30155.6) | 944 (788 to 1118.8) | 823.5 (671.8 to 983) | 1057.3 (876.5 to 1259.4) | -95.7 (-97 to -94.5) | -95.8 (-97.3 to -94.5) | -95.7 (-96.9 to -94.3) |
|  | Injuries | DALYs (Disability-Adjusted Life Years) | Number | 70256.2 (59503.7 to 80831.5) | 25748.6 (19788.1 to 30922.8) | 44507.6 (37512.3 to 52992.2) | 6008.9 (5259 to 6689.6) | 1811.8 (1600.9 to 2029.9) | 4197.1 (3581.1 to 4777.3) | -91.4 (-93.1 to -89.6) | -93 (-94.5 to -90.7) | -90.6 (-92.8 to -88.4) |
|  |  |  | Rate | 7731.6 (6548.3 to 8895.4) | 5805.7 (4461.8 to 6972.4) | 9567.8 (8064 to 11391.7) | 1104.3 (966.5 to 1229.4) | 687.2 (607.2 to 770) | 1496.3 (1276.7 to 1703.2) | -85.7 (-88.5 to -82.6) | -88.2 (-90.7 to -84.3) | -84.4 (-88 to -80.7) |
|  |  | YLDs (Years Lived with Disability) | Number | 3400.3 (2549.1 to 4472.3) | 1479.3 (1100 to 1944.1) | 1921 (1436.1 to 2532.1) | 657.8 (476.2 to 882) | 298.3 (215 to 400.8) | 359.5 (260.3 to 484.2) | -80.7 (-82.8 to -78.7) | -79.8 (-82.1 to -77.5) | -81.3 (-83.7 to -79.1) |
|  |  |  | Rate | 374.2 (280.5 to 492.2) | 333.6 (248 to 438.4) | 413 (308.7 to 544.3) | 120.9 (87.5 to 162.1) | 113.1 (81.6 to 152) | 128.2 (92.8 to 172.6) | -67.7 (-71.3 to -64.4) | -66.1 (-69.8 to -62.2) | -69 (-73 to -65.3) |
|  |  | YLLs (Years of Life Lost) | Number | 66855.8 (56137.7 to 77724.8) | 24269.3 (18488.1 to 29391.8) | 42586.5 (35539.9 to 51212.8) | 5351.1 (4650.8 to 6039.6) | 1513.5 (1318.8 to 1706.7) | 3837.6 (3261.5 to 4402.3) | -92 (-93.7 to -90.1) | -93.8 (-95.2 to -91.4) | -91 (-93.2 to -88.8) |
|  |  |  | Rate | 7357.4 (6177.9 to 8553.5) | 5472.2 (4168.6 to 6627.2) | 9154.8 (7640 to 11009.2) | 983.4 (854.7 to 1110) | 574.1 (500.2 to 647.4) | 1368.2 (1162.8 to 1569.5) | -86.6 (-89.5 to -83.5) | -89.5 (-92 to -85.6) | -85.1 (-88.8 to -81.4) |
|  | Non-communicable diseases | DALYs (Disability-Adjusted Life Years) | Number | 128996.6 (91831.5 to 158133.8) | 62074.8 (41628.4 to 76797.5) | 66921.8 (44787.5 to 84335) | 25315.3 (19156.2 to 32546.2) | 12759 (9507.2 to 16515.6) | 12556.3 (9702.5 to 15895) | -80.4 (-85.3 to -70.6) | -79.4 (-85.1 to -67.7) | -81.2 (-85.9 to -70) |
|  |  |  | Rate | 14196 (10106 to 17402.5) | 13996.5 (9386.3 to 17316.1) | 14386.2 (9628 to 18129.5) | 4652.4 (3520.5 to 5981.3) | 4839.6 (3606.2 to 6264.5) | 4476.5 (3459.1 to 5666.8) | -67.2 (-75.5 to -50.8) | -65.4 (-74.9 to -45.7) | -68.9 (-76.6 to -50.3) |
|  |  | YLDs (Years Lived with Disability) | Number | 32722.9 (23669.2 to 43540.9) | 16870.9 (12194.3 to 22492.9) | 15852.1 (11525.8 to 21276.3) | 20499.6 (14589.1 to 27875.9) | 10760.6 (7561.2 to 14525.8) | 9739 (6995.5 to 13010.4) | -37.4 (-42 to -32.3) | -36.2 (-41.3 to -31) | -38.6 (-43.7 to -33.1) |
|  |  |  | Rate | 3601.1 (2604.8 to 4791.6) | 3804 (2749.5 to 5071.7) | 3407.7 (2477.7 to 4573.8) | 3767.4 (2681.2 to 5123) | 4081.6 (2868 to 5509.8) | 3472.1 (2494 to 4638.4) | 4.6 (-3.1 to 13.1) | 7.3 (-1.2 to 16.1) | 1.9 (-6.6 to 10.9) |
|  |  | YLLs (Years of Life Lost) | Number | 96273.6 (60259.8 to 122089.2) | 45203.9 (25985.5 to 58049.6) | 51069.7 (29391.9 to 68157) | 4815.7 (4156.7 to 5678) | 1998.4 (1695.2 to 2503.1) | 2817.2 (2356.8 to 3378.1) | -95 (-96.3 to -91.1) | -95.6 (-96.7 to -90.8) | -94.5 (-96.1 to -89) |
|  |  |  | Rate | 10594.8 (6631.5 to 13435.8) | 10192.5 (5859.2 to 13088.9) | 10978.4 (6318.4 to 14651.7) | 885 (763.9 to 1043.5) | 758 (643 to 949.4) | 1004.4 (840.2 to 1204.4) | -91.6 (-93.8 to -85.1) | -92.6 (-94.5 to -84.5) | -90.9 (-93.6 to -81.8) |
| Markazi | All causes | DALYs (Disability-Adjusted Life Years) | Number | 257021 (228452.3 to 288045.1) | 112221.9 (100139 to 126547.8) | 144799.1 (128591 to 161768.7) | 37466.3 (31427 to 44307.2) | 17751.7 (14728.6 to 21443.4) | 19714.6 (16565.6 to 23070.8) | -85.4 (-87.8 to -83) | -84.2 (-86.9 to -81.4) | -86.4 (-88.6 to -84.2) |
|  |  |  | Rate | 39180.4 (34825.4 to 43909.8) | 34827.8 (31077.9 to 39273.8) | 43382.3 (38526.4 to 48466.5) | 9524.8 (7989.5 to 11263.9) | 9254.6 (7678.6 to 11179.2) | 9781.9 (8219.5 to 11447.3) | -75.7 (-79.7 to -71.6) | -73.4 (-78.1 to -68.8) | -77.5 (-81.1 to -73.9) |
|  |  | YLDs (Years Lived with Disability) | Number | 36768 (26803.4 to 48949.1) | 18642.8 (13615.7 to 24685.4) | 18125.2 (13226.8 to 24471.4) | 20482.5 (14865.4 to 27279.9) | 10866.9 (7954.5 to 14575.5) | 9615.6 (6977.4 to 12866.4) | -44.3 (-48.8 to -39.2) | -41.7 (-47 to -36) | -46.9 (-52.4 to -40.9) |
|  |  |  | Rate | 5604.9 (4085.9 to 7461.8) | 5785.8 (4225.6 to 7661.1) | 5430.4 (3962.8 to 7331.7) | 5207.1 (3779.1 to 6935.2) | 5665.3 (4147 to 7598.7) | 4771 (3462 to 6384) | -7.1 (-14.7 to 1.4) | -2.1 (-11 to 7.5) | -12.1 (-21.2 to -2.1) |
|  |  | YLLs (Years of Life Lost) | Number | 220253 (194478.6 to 246211.7) | 93579.1 (82435.8 to 105155.9) | 126673.9 (111493.4 to 142138.5) | 16983.8 (15609.9 to 18393.8) | 6884.8 (6257.7 to 7555.1) | 10099 (9069.1 to 11136.5) | -92.3 (-93.4 to -91) | -92.6 (-93.7 to -91.4) | -92 (-93.3 to -90.6) |
|  |  |  | Rate | 33575.5 (29646.4 to 37532.6) | 29042.1 (25583.8 to 32634.9) | 37952 (33403.8 to 42585.2) | 4317.7 (3968.4 to 4676.1) | 3589.3 (3262.4 to 3938.8) | 5010.9 (4499.9 to 5525.7) | -87.1 (-89 to -85) | -87.6 (-89.5 to -85.5) | -86.8 (-88.9 to -84.5) |
|  | Communicable, maternal, neonatal, and nutritional diseases | DALYs (Disability-Adjusted Life Years) | Number | 118827.4 (99647 to 143833.9) | 53486.1 (44530.7 to 67428.9) | 65341.3 (54331.5 to 81138.9) | 8960.7 (7586.1 to 10699.8) | 4458.9 (3720.6 to 5463.8) | 4501.9 (3786.6 to 5338.7) | -92.5 (-94.1 to -90.5) | -91.7 (-93.8 to -89.4) | -93.1 (-94.8 to -91.2) |
|  |  |  | Rate | 18114.1 (15190.2 to 21926.1) | 16599.3 (13820 to 20926.4) | 19576.5 (16277.9 to 24309.5) | 2278 (1928.6 to 2720.1) | 2324.6 (1939.7 to 2848.5) | 2233.7 (1878.8 to 2648.9) | -87.4 (-90.2 to -84.2) | -86 (-89.6 to -82.3) | -88.6 (-91.4 to -85.4) |
|  |  | YLDs (Years Lived with Disability) | Number | 8562.3 (5972.1 to 12276.6) | 4251.3 (2824.3 to 6287.6) | 4311.1 (2751.8 to 6592.8) | 3613.8 (2522.4 to 5216.6) | 1984.2 (1364.2 to 2881.5) | 1629.6 (1060.7 to 2449.4) | -57.8 (-66.5 to -43.4) | -53.3 (-65.2 to -35) | -62.2 (-73 to -45.1) |
|  |  |  | Rate | 1305.2 (910.4 to 1871.5) | 1319.4 (876.5 to 1951.3) | 1291.6 (824.4 to 1975.2) | 918.7 (641.3 to 1326.2) | 1034.5 (711.2 to 1502.2) | 808.6 (526.3 to 1215.3) | -29.6 (-44.2 to -5.5) | -21.6 (-41.5 to 9.3) | -37.4 (-55.2 to -9.1) |
|  |  | YLLs (Years of Life Lost) | Number | 110265 (90537.7 to 136444.9) | 49234.8 (40161.5 to 62639.9) | 61030.2 (49854.8 to 76133.2) | 5346.9 (4610.7 to 6096) | 2474.6 (2074.9 to 2855.1) | 2872.3 (2439.9 to 3317.3) | -95.2 (-96.3 to -94) | -95 (-96.5 to -93.7) | -95.3 (-96.7 to -94) |
|  |  |  | Rate | 16808.9 (13801.6 to 20799.7) | 15279.9 (12464 to 19440.1) | 18284.9 (14936.7 to 22809.8) | 1359.3 (1172.1 to 1549.7) | 1290.1 (1081.7 to 1488.5) | 1425.2 (1210.6 to 1646) | -91.9 (-93.9 to -89.9) | -91.6 (-94.1 to -89.4) | -92.2 (-94.5 to -90.1) |
|  | Injuries | DALYs (Disability-Adjusted Life Years) | Number | 53442.7 (47598.3 to 60270.8) | 18115.7 (15332.3 to 21209.7) | 35327 (31093.9 to 40356.4) | 6406.9 (5669.9 to 7148) | 2028 (1819.8 to 2283) | 4378.9 (3758.3 to 4971.6) | -88 (-90.1 to -85.9) | -88.8 (-91 to -86.1) | -87.6 (-90.1 to -85) |
|  |  |  | Rate | 8146.8 (7255.9 to 9187.7) | 5622.2 (4758.3 to 6582.4) | 10584.1 (9315.9 to 12090.9) | 1628.8 (1441.4 to 1817.2) | 1057.3 (948.7 to 1190.2) | 2172.7 (1864.8 to 2466.8) | -80 (-83.4 to -76.4) | -81.2 (-85 to -76.7) | -79.5 (-83.6 to -75.2) |
|  |  | YLDs (Years Lived with Disability) | Number | 2910.1 (2172.4 to 3868.9) | 1248.6 (935.4 to 1642.8) | 1661.5 (1229.7 to 2185) | 603.2 (435.1 to 801.4) | 279.4 (198.8 to 373.7) | 323.7 (236.2 to 435.5) | -79.3 (-81.3 to -77.5) | -77.6 (-79.9 to -75.2) | -80.5 (-82.7 to -78.6) |
|  |  |  | Rate | 443.6 (331.2 to 589.8) | 387.5 (290.3 to 509.8) | 497.8 (368.4 to 654.6) | 153.3 (110.6 to 203.7) | 145.7 (103.6 to 194.8) | 160.6 (117.2 to 216.1) | -65.4 (-68.8 to -62.4) | -62.4 (-66.2 to -58.3) | -67.7 (-71.3 to -64.5) |
|  |  | YLLs (Years of Life Lost) | Number | 50532.6 (44822.1 to 57166.2) | 16867.1 (14195.1 to 19872.7) | 33665.5 (29574 to 38923.2) | 5803.7 (5054.2 to 6490.3) | 1748.6 (1550.6 to 1991.5) | 4055.1 (3424.2 to 4647) | -88.5 (-90.6 to -86.3) | -89.6 (-91.8 to -86.9) | -88 (-90.5 to -85.3) |
|  |  |  | Rate | 7703.2 (6832.7 to 8714.5) | 5234.7 (4405.4 to 6167.4) | 10086.3 (8860.5 to 11661.5) | 1475.4 (1284.9 to 1650) | 911.6 (808.4 to 1038.3) | 2012.1 (1699 to 2305.7) | -80.8 (-84.3 to -77.1) | -82.6 (-86.3 to -78) | -80.1 (-84.2 to -75.6) |
|  | Non-communicable diseases | DALYs (Disability-Adjusted Life Years) | Number | 84750.9 (65421.7 to 102280.3) | 40620.1 (28651.9 to 49612.2) | 44130.8 (32229.1 to 53591.6) | 21977.9 (17394.8 to 27727.6) | 11233.4 (8851.7 to 14250.2) | 10744.5 (8512.1 to 13397.4) | -74.1 (-79.4 to -64.2) | -72.3 (-77.9 to -60.2) | -75.7 (-81 to -64.9) |
|  |  |  | Rate | 12919.5 (9972.9 to 15591.7) | 12606.4 (8892 to 15397) | 13221.8 (9655.9 to 16056.3) | 5587.3 (4422.2 to 7049) | 5856.4 (4614.7 to 7429.2) | 5331.2 (4223.5 to 6647.5) | -56.8 (-65.6 to -40.2) | -53.5 (-62.9 to -33.1) | -59.7 (-68.6 to -41.9) |
|  |  | YLDs (Years Lived with Disability) | Number | 25295.6 (18431.5 to 34114.3) | 13142.9 (9566.7 to 17683.7) | 12152.6 (8838 to 16388.7) | 16265.4 (11769.3 to 21755) | 8603.2 (6264.4 to 11610.7) | 7662.2 (5554.4 to 10192) | -35.7 (-40.7 to -30.8) | -34.5 (-39.9 to -29) | -37 (-42.3 to -31) |
|  |  |  | Rate | 3856.1 (2809.7 to 5200.4) | 4078.9 (2969 to 5488.1) | 3641 (2647.9 to 4910.1) | 4135.1 (2992 to 5530.6) | 4485.2 (3265.9 to 6053.1) | 3801.8 (2756 to 5057) | 7.2 (-1.1 to 15.4) | 10 (1 to 19.2) | 4.4 (-4.4 to 14.3) |
|  |  | YLLs (Years of Life Lost) | Number | 59455.4 (40283.4 to 75395.9) | 27477.2 (16467.3 to 35380.3) | 31978.2 (20462 to 41819) | 5712.4 (5062.1 to 6567.5) | 2630.2 (2296.6 to 3100.6) | 3082.3 (2605.6 to 3666.9) | -90.4 (-92.6 to -84.6) | -90.4 (-92.7 to -82.2) | -90.4 (-92.8 to -83) |
|  |  |  | Rate | 9063.4 (6140.8 to 11493.4) | 8527.5 (5110.6 to 10980.2) | 9580.8 (6130.5 to 12529.1) | 1452.2 (1286.9 to 1669.6) | 1371.2 (1197.3 to 1616.5) | 1529.4 (1292.9 to 1819.4) | -84 (-87.7 to -74.3) | -83.9 (-87.7 to -70.1) | -84 (-88.1 to -71.9) |
| Mazandaran | All causes | DALYs (Disability-Adjusted Life Years) | Number | 366633.2 (324155.4 to 415366.9) | 155414.2 (137321.1 to 178171.3) | 211219 (186202.7 to 239864.6) | 78994 (64720.4 to 95862.9) | 38399.4 (30517 to 47487.2) | 40594.6 (34141.6 to 47901.8) | -78.5 (-82.3 to -74.6) | -75.3 (-79.8 to -71) | -80.8 (-84 to -77.2) |
|  |  |  | Rate | 26667.5 (23577.8 to 30212.2) | 22799.5 (20145.2 to 26138) | 30471.3 (26862.3 to 34603.8) | 8787.3 (7199.5 to 10663.8) | 8661.7 (6883.6 to 10711.6) | 8909.6 (7493.3 to 10513.4) | -67 (-73 to -61.2) | -62 (-68.9 to -55.3) | -70.8 (-75.7 to -65.3) |
|  |  | YLDs (Years Lived with Disability) | Number | 73677.5 (53786.5 to 97558.7) | 39159.5 (28492.8 to 51857.4) | 34517.9 (25073.7 to 46411.2) | 48212.6 (34719 to 64704.1) | 26773.5 (19147.2 to 36204.7) | 21439.1 (15404.7 to 28692.3) | -34.6 (-39.8 to -28) | -31.6 (-37.3 to -25) | -37.9 (-44.6 to -31.1) |
|  |  |  | Rate | 5359 (3912.2 to 7096.1) | 5744.8 (4179.9 to 7607.6) | 4979.7 (3617.2 to 6695.5) | 5363.2 (3862.2 to 7197.7) | 6039.2 (4319 to 8166.6) | 4705.4 (3381 to 6297.3) | 0.1 (-7.9 to 10.1) | 5.1 (-3.6 to 15.4) | -5.5 (-15.8 to 4.9) |
|  |  | YLLs (Years of Life Lost) | Number | 292955.7 (255303.4 to 333749.4) | 116254.7 (101275 to 132819.5) | 176701.1 (153659.2 to 200918.1) | 30781.5 (27980.8 to 33916.6) | 11625.9 (10434.5 to 12862.5) | 19155.6 (16978.5 to 21510.8) | -89.5 (-91.2 to -87.3) | -90 (-91.6 to -88.1) | -89.2 (-91 to -86.7) |
|  |  |  | Rate | 21308.5 (18569.8 to 24275.7) | 17054.7 (14857.2 to 19484.8) | 25491.6 (22167.5 to 28985.2) | 3424.1 (3112.6 to 3772.9) | 2622.4 (2353.7 to 2901.4) | 4204.2 (3726.4 to 4721.1) | -83.9 (-86.6 to -80.6) | -84.6 (-87.1 to -81.6) | -83.5 (-86.3 to -79.8) |
|  | Communicable, maternal, neonatal, and nutritional diseases | DALYs (Disability-Adjusted Life Years) | Number | 139521.4 (111395.5 to 170874.4) | 60787.2 (48160.1 to 79158.6) | 78734.2 (62422.3 to 98462.1) | 14572.5 (11763.4 to 18114) | 7360.3 (5739.5 to 9432.9) | 7212.2 (5827.5 to 8918.2) | -89.6 (-92.2 to -86.1) | -87.9 (-91.4 to -83.3) | -90.8 (-93.3 to -87.6) |
|  |  |  | Rate | 10148.3 (8102.5 to 12428.8) | 8917.6 (7065.2 to 11612.7) | 11358.5 (9005.3 to 14204.5) | 1621.1 (1308.6 to 2015) | 1660.2 (1294.6 to 2127.8) | 1582.9 (1279 to 1957.3) | -84 (-88.1 to -78.7) | -81.4 (-86.7 to -74.3) | -86.1 (-89.7 to -81.2) |
|  |  | YLDs (Years Lived with Disability) | Number | 15940.8 (11102.2 to 22286.3) | 8157.3 (5375.3 to 11849.6) | 7783.5 (5080.8 to 11667.9) | 7848.4 (5394.2 to 11391.5) | 4352.7 (2942.5 to 6272.6) | 3495.7 (2324.8 to 5120.2) | -50.8 (-61 to -35) | -46.6 (-59.3 to -27.5) | -55.1 (-67.3 to -34.9) |
|  |  |  | Rate | 1159.5 (807.5 to 1621) | 1196.7 (788.6 to 1738.4) | 1122.9 (733 to 1683.3) | 873.1 (600.1 to 1267.2) | 981.8 (663.7 to 1414.9) | 767.2 (510.2 to 1123.8) | -24.7 (-40.4 to -0.5) | -18 (-37.4 to 11.5) | -31.7 (-50.3 to -0.9) |
|  |  | YLLs (Years of Life Lost) | Number | 123580.5 (95667.1 to 154425) | 52629.9 (40322.1 to 69697.9) | 70950.7 (54885.4 to 90319) | 6724.1 (5564.4 to 8026.8) | 3007.6 (2433.8 to 3701.3) | 3716.5 (3028.2 to 4406.9) | -94.6 (-96 to -92.8) | -94.3 (-96.3 to -92.2) | -94.8 (-96.4 to -93.1) |
|  |  |  | Rate | 8988.8 (6958.5 to 11232.3) | 7720.9 (5915.3 to 10224.8) | 10235.6 (7918 to 13029.8) | 748 (619 to 892.9) | 678.4 (549 to 834.9) | 815.7 (664.6 to 967.2) | -91.7 (-93.9 to -89) | -91.2 (-94.3 to -88.1) | -92 (-94.5 to -89.5) |
|  | Injuries | DALYs (Disability-Adjusted Life Years) | Number | 70005.4 (60165.4 to 80391.9) | 21522.8 (17926.9 to 25772.2) | 48482.6 (41634.2 to 55567.4) | 14378.5 (12722.7 to 16145.8) | 4262.2 (3768.5 to 4809.2) | 10116.2 (8819.3 to 11680.2) | -79.5 (-82.9 to -75.1) | -80.2 (-83.9 to -75.7) | -79.1 (-83.1 to -74) |
|  |  |  | Rate | 5091.9 (4376.2 to 5847.4) | 3157.4 (2629.9 to 3780.8) | 6994.3 (6006.3 to 8016.4) | 1599.5 (1415.3 to 1796.1) | 961.4 (850 to 1084.8) | 2220.3 (1935.6 to 2563.5) | -68.6 (-73.8 to -61.9) | -69.6 (-75.3 to -62.6) | -68.3 (-74.3 to -60.4) |
|  |  | YLDs (Years Lived with Disability) | Number | 4806.2 (3541.2 to 6399.3) | 2050.4 (1487.2 to 2720.8) | 2755.8 (2051.6 to 3697.8) | 1389.6 (997.5 to 1850.6) | 632.2 (456.1 to 846) | 757.4 (545.3 to 1030.1) | -71.1 (-73.9 to -68.5) | -69.2 (-72.1 to -66.4) | -72.5 (-75.6 to -69.6) |
|  |  |  | Rate | 349.6 (257.6 to 465.5) | 300.8 (218.2 to 399.2) | 397.6 (296 to 533.5) | 154.6 (111 to 205.9) | 142.6 (102.9 to 190.8) | 166.2 (119.7 to 226.1) | -55.8 (-60.1 to -51.8) | -52.6 (-57.1 to -48.3) | -58.2 (-62.8 to -53.7) |
|  |  | YLLs (Years of Life Lost) | Number | 65199.2 (55790.7 to 75172) | 19472.4 (15962.8 to 23653.1) | 45726.8 (38859.5 to 53013.8) | 12988.8 (11381.4 to 14629.5) | 3630 (3163.5 to 4161.7) | 9358.8 (8064.1 to 10885.2) | -80.1 (-83.6 to -75.6) | -81.4 (-85.5 to -76.5) | -79.5 (-83.8 to -74.2) |
|  |  |  | Rate | 4742.3 (4058 to 5467.7) | 2856.6 (2341.8 to 3469.9) | 6596.7 (5606 to 7648) | 1444.9 (1266.1 to 1627.4) | 818.8 (713.6 to 938.7) | 2054.1 (1769.9 to 2389.1) | -69.5 (-75 to -62.7) | -71.3 (-77.7 to -63.9) | -68.9 (-75.3 to -60.7) |
|  | Non-communicable diseases | DALYs (Disability-Adjusted Life Years) | Number | 157106.4 (129751.9 to 186021.7) | 73104.2 (55066.4 to 88212.2) | 84002.2 (66158.6 to 102067) | 49625.1 (38316 to 62979.6) | 26646.1 (20386 to 34238.8) | 22979.1 (17981.2 to 28884.4) | -68.4 (-75.3 to -60.7) | -63.6 (-71.2 to -52.1) | -72.6 (-79.3 to -63.8) |
|  |  |  | Rate | 11427.3 (9437.7 to 13530.5) | 10724.5 (8078.3 to 12940.9) | 12118.5 (9544.3 to 14724.6) | 5520.3 (4262.3 to 7005.9) | 6010.5 (4598.4 to 7723.2) | 5043.4 (3946.5 to 6339.5) | -51.7 (-62.3 to -39.9) | -44 (-55.8 to -26.3) | -58.4 (-68.6 to -44.9) |
|  |  | YLDs (Years Lived with Disability) | Number | 52930.5 (38506 to 70333.1) | 28951.8 (21045.5 to 38451.1) | 23978.7 (17246.8 to 32066.4) | 38974.5 (27851.1 to 52485.1) | 21788.5 (15605.6 to 29431.7) | 17186 (12198.1 to 23000.6) | -26.4 (-31.5 to -21) | -24.7 (-30 to -18.8) | -28.3 (-34.6 to -22.3) |
|  |  |  | Rate | 3850 (2800.8 to 5115.8) | 4247.3 (3087.4 to 5640.8) | 3459.3 (2488.1 to 4626) | 4335.5 (3098.2 to 5838.5) | 4914.8 (3520.1 to 6638.8) | 3771.9 (2677.2 to 5048.1) | 12.6 (4.7 to 20.8) | 15.7 (7.7 to 24.8) | 9 (-0.5 to 18.2) |
|  |  | YLLs (Years of Life Lost) | Number | 104176 (79793.1 to 129429.9) | 44152.4 (28588.2 to 55646.5) | 60023.6 (43386.1 to 75889.7) | 10650.6 (8834.6 to 12458.8) | 4857.6 (3990.7 to 5623.2) | 5793.1 (4745 to 7053.8) | -89.8 (-92 to -85.5) | -89 (-91.6 to -82.1) | -90.3 (-92.8 to -84.3) |
|  |  |  | Rate | 7577.4 (5803.9 to 9414.2) | 6477.2 (4193.9 to 8163.4) | 8659.2 (6259 to 10948.1) | 1184.8 (982.8 to 1385.9) | 1095.7 (900.2 to 1268.4) | 1271.5 (1041.4 to 1548.1) | -84.4 (-87.8 to -77.9) | -83.1 (-87.1 to -72.4) | -85.3 (-89.1 to -76.2) |
| North Khorasan | All causes | DALYs (Disability-Adjusted Life Years) | Number | 233444.5 (208389.5 to 260950.5) | 105968.6 (93824.4 to 118849.5) | 127475.9 (113641.2 to 143532.3) | 30681.8 (25970 to 35872.7) | 14423.7 (12070.4 to 17308.1) | 16258.2 (13929.5 to 18760.3) | -86.9 (-89.2 to -84.4) | -86.4 (-88.9 to -83.7) | -87.2 (-89.4 to -85) |
|  |  |  | Rate | 67388.9 (60156.2 to 75329.1) | 61915.7 (54820.1 to 69441.8) | 72733.6 (64839.9 to 81894.9) | 10304.6 (8722.1 to 12048) | 9932.1 (8311.7 to 11918.3) | 10659.3 (9132.6 to 12299.8) | -84.7 (-87.4 to -81.8) | -84 (-86.9 to -80.8) | -85.3 (-87.8 to -82.7) |
|  |  | YLDs (Years Lived with Disability) | Number | 20091.6 (14679.6 to 26849.2) | 10128.5 (7408.5 to 13365.3) | 9963.1 (7266.1 to 13539.3) | 15186.9 (10922.2 to 20575.8) | 7988.3 (5799.6 to 10813.1) | 7198.5 (5151.2 to 9667) | -24.4 (-31.5 to -17.3) | -21.1 (-28.5 to -13.4) | -27.7 (-35.7 to -19.5) |
|  |  |  | Rate | 5799.9 (4237.6 to 7750.6) | 5917.9 (4328.6 to 7809.1) | 5684.6 (4145.8 to 7725.1) | 5100.6 (3668.3 to 6910.5) | 5500.8 (3993.6 to 7445.9) | 4719.6 (3377.3 to 6337.9) | -12.1 (-20.3 to -3.8) | -7 (-15.8 to 2) | -17 (-26.1 to -7.5) |
|  |  | YLLs (Years of Life Lost) | Number | 213352.9 (189364.2 to 240313.5) | 95840.1 (84546.3 to 108179) | 117512.8 (103634.5 to 132866.5) | 15495 (14149.7 to 16918.3) | 6435.3 (5833.9 to 7052.2) | 9059.6 (8186.9 to 10021.9) | -92.7 (-93.7 to -91.5) | -93.3 (-94.3 to -92.1) | -92.3 (-93.4 to -91) |
|  |  |  | Rate | 61589 (54664.2 to 69371.8) | 55997.8 (49399 to 63207.3) | 67049 (59130.5 to 75809.3) | 5204 (4752.2 to 5682.1) | 4431.4 (4017.2 to 4856.2) | 5939.7 (5367.5 to 6570.6) | -91.6 (-92.7 to -90.1) | -92.1 (-93.3 to -90.7) | -91.1 (-92.4 to -89.6) |
|  | Communicable, maternal, neonatal, and nutritional diseases | DALYs (Disability-Adjusted Life Years) | Number | 116707.7 (99564.8 to 145908.8) | 53201.2 (44219.4 to 68964.9) | 63506.6 (53525.7 to 80402.1) | 7700 (6545.7 to 9053) | 3731.2 (3102.9 to 4525.1) | 3968.9 (3343.7 to 4817.4) | -93.4 (-95.1 to -91.6) | -93 (-95.1 to -90.9) | -93.8 (-95.5 to -91.9) |
|  |  |  | Rate | 33690.3 (28741.6 to 42119.8) | 31084.6 (25836.7 to 40295.1) | 36234.8 (30540 to 45874.8) | 2586.1 (2198.4 to 3040.5) | 2569.3 (2136.7 to 3115.9) | 2602.1 (2192.2 to 3158.4) | -92.3 (-94.3 to -90.2) | -91.7 (-94.2 to -89.3) | -92.8 (-94.8 to -90.7) |
|  |  | YLDs (Years Lived with Disability) | Number | 5482.7 (3684.3 to 7762.8) | 2586.3 (1674.8 to 3694.5) | 2896.4 (1876.3 to 4220.2) | 2924.6 (1967.6 to 4157.8) | 1545.3 (1030.3 to 2281.8) | 1379.4 (894.4 to 2022.5) | -46.7 (-58 to -29.9) | -40.3 (-54.9 to -20.5) | -52.4 (-65.4 to -29.9) |
|  |  |  | Rate | 1582.7 (1063.6 to 2240.9) | 1511.2 (978.6 to 2158.6) | 1652.6 (1070.5 to 2407.9) | 982.2 (660.8 to 1396.4) | 1064.1 (709.5 to 1571.3) | 904.3 (586.4 to 1326) | -37.9 (-51.2 to -18.4) | -29.6 (-46.8 to -6.3) | -45.3 (-60.2 to -19.5) |
|  |  | YLLs (Years of Life Lost) | Number | 111225 (93427 to 140767) | 50614.8 (41833.1 to 66036.5) | 60610.2 (50738.9 to 77223.7) | 4775.4 (4075.5 to 5509) | 2185.9 (1847.7 to 2548.9) | 2589.5 (2156.7 to 3007.5) | -95.7 (-96.9 to -94.5) | -95.7 (-97.2 to -94.4) | -95.7 (-97.1 to -94.5) |
|  |  |  | Rate | 32107.6 (26969.8 to 40635.5) | 29573.4 (24442.4 to 38584.1) | 34582.2 (28950 to 44061.4) | 1603.8 (1368.8 to 1850.2) | 1505.2 (1272.3 to 1755.2) | 1697.8 (1414 to 1971.8) | -95 (-96.4 to -93.5) | -94.9 (-96.7 to -93.4) | -95.1 (-96.6 to -93.7) |
|  | Injuries | DALYs (Disability-Adjusted Life Years) | Number | 43079.4 (36949.9 to 51042.1) | 17276.6 (14173.6 to 21103.3) | 25802.9 (21647.4 to 30889.5) | 5719.5 (5156 to 6200.8) | 1897.8 (1692.6 to 2134.5) | 3821.7 (3364.3 to 4261.7) | -86.7 (-89.3 to -83.9) | -89 (-91.3 to -86) | -85.2 (-88.4 to -81.7) |
|  |  |  | Rate | 12435.8 (10666.4 to 14734.4) | 10094.4 (8281.4 to 12330.3) | 14722.3 (12351.3 to 17624.6) | 1920.9 (1731.7 to 2082.6) | 1306.8 (1165.5 to 1469.8) | 2505.6 (2205.7 to 2794.1) | -84.6 (-87.5 to -81.3) | -87.1 (-89.7 to -83.5) | -83 (-86.7 to -79) |
|  |  | YLDs (Years Lived with Disability) | Number | 1359.3 (999.7 to 1766.9) | 637.8 (464.2 to 843.6) | 721.6 (532.1 to 949.1) | 453.2 (324.8 to 604.1) | 206.8 (148 to 275) | 246.4 (175.9 to 330.1) | -66.7 (-69.6 to -63.9) | -67.6 (-70.7 to -64.2) | -65.9 (-68.6 to -63.2) |
|  |  |  | Rate | 392.4 (288.6 to 510.1) | 372.6 (271.2 to 492.9) | 411.7 (303.6 to 541.5) | 152.2 (109.1 to 202.9) | 142.4 (101.9 to 189.3) | 161.5 (115.3 to 216.4) | -61.2 (-64.6 to -58) | -61.8 (-65.5 to -57.8) | -60.8 (-63.9 to -57.7) |
|  |  | YLLs (Years of Life Lost) | Number | 41720.1 (35705.3 to 49629.7) | 16638.8 (13436.9 to 20549.7) | 25081.3 (21026.6 to 30117.7) | 5266.3 (4703.1 to 5800.9) | 1691 (1496.9 to 1911.9) | 3575.3 (3125.3 to 4038.7) | -87.4 (-90 to -84.6) | -89.8 (-92 to -87.1) | -85.7 (-89 to -82.2) |
|  |  |  | Rate | 12043.4 (10307.1 to 14326.7) | 9721.8 (7851 to 12006.9) | 14310.6 (11997.1 to 17184.2) | 1768.7 (1579.6 to 1948.3) | 1164.4 (1030.8 to 1316.6) | 2344.1 (2049.1 to 2647.9) | -85.3 (-88.3 to -82.1) | -88 (-90.5 to -84.8) | -83.6 (-87.4 to -79.6) |
|  | Non-communicable diseases | DALYs (Disability-Adjusted Life Years) | Number | 73657.3 (51468.5 to 88441.9) | 35490.8 (22615.4 to 43053.1) | 38166.5 (24470.5 to 47777.9) | 17146.2 (13505.2 to 21288.7) | 8761.5 (6879.6 to 10957.7) | 8384.7 (6657.1 to 10245.6) | -76.7 (-82.4 to -64.1) | -75.3 (-81.6 to -60.1) | -78 (-83.8 to -64) |
|  |  |  | Rate | 21262.8 (14857.5 to 25530.7) | 20736.7 (13213.8 to 25155.2) | 21776.6 (13962.1 to 27260.5) | 5758.6 (4535.8 to 7149.9) | 6033.1 (4737.2 to 7545.4) | 5497.2 (4364.6 to 6717.3) | -72.9 (-79.6 to -58.2) | -70.9 (-78.3 to -52.9) | -74.8 (-81.4 to -58.6) |
|  |  | YLDs (Years Lived with Disability) | Number | 13249.6 (9638.8 to 17526.8) | 6904.4 (4993.8 to 9162.8) | 6345.2 (4656.9 to 8369.8) | 11809.1 (8583 to 15846.7) | 6236.3 (4500.1 to 8341.4) | 5572.8 (4041.8 to 7464.8) | -10.9 (-18 to -2.9) | -9.7 (-17.3 to -1.3) | -12.2 (-19.9 to -3.9) |
|  |  |  | Rate | 3824.8 (2782.5 to 5059.5) | 4034.1 (2917.8 to 5353.7) | 3620.3 (2657.1 to 4775.6) | 3966.1 (2882.6 to 5322.2) | 4294.3 (3098.8 to 5743.8) | 3653.7 (2649.9 to 4894.1) | 3.7 (-4.6 to 13) | 6.4 (-2.5 to 16.3) | 0.9 (-8 to 10.4) |
|  |  | YLLs (Years of Life Lost) | Number | 60407.8 (37191.1 to 74181.8) | 28586.4 (15908.2 to 35607) | 31821.3 (18508 to 41128.2) | 5337 (4700.8 to 6160.4) | 2525.2 (2212.4 to 2941.1) | 2811.9 (2390.5 to 3404.6) | -91.2 (-93.2 to -84) | -91.2 (-93.3 to -82.9) | -91.2 (-93.4 to -83.1) |
|  |  |  | Rate | 17438 (10736 to 21414.2) | 16702.6 (9294.9 to 20804.6) | 18156.2 (10560.1 to 23466.4) | 1792.5 (1578.8 to 2069) | 1738.8 (1523.4 to 2025.3) | 1843.5 (1567.3 to 2232.1) | -89.7 (-92 to -81.4) | -89.6 (-92.1 to -79.9) | -89.8 (-92.5 to -80.6) |
| Qazvin | All causes | DALYs (Disability-Adjusted Life Years) | Number | 222502.3 (199852.9 to 246615.2) | 100454.3 (90394.4 to 111402.8) | 122048 (108864.6 to 136167.3) | 32575.7 (26769 to 39390.8) | 15273.3 (12254.2 to 18858.9) | 17302.4 (14530.3 to 20589.6) | -85.4 (-88 to -82.5) | -84.8 (-87.7 to -81.7) | -85.8 (-88.3 to -83) |
|  |  |  | Rate | 40972 (36801.3 to 45412.2) | 38100.5 (34285 to 42253) | 43681.6 (38963.2 to 48735) | 8539.3 (7017.1 to 10325.8) | 8245.4 (6615.5 to 10181.1) | 8816.7 (7404.2 to 10491.8) | -79.2 (-83 to -75) | -78.4 (-82.5 to -74) | -79.8 (-83.3 to -75.7) |
|  |  | YLDs (Years Lived with Disability) | Number | 29355.5 (21409.1 to 39128.3) | 14811.7 (10708.3 to 19804.3) | 14543.8 (10518.8 to 19532.4) | 19109.8 (13867.2 to 25693.8) | 10132.8 (7275 to 13740.4) | 8977 (6537.7 to 12140) | -34.9 (-40.3 to -29) | -31.6 (-37.7 to -24.2) | -38.3 (-44.6 to -30.9) |
|  |  |  | Rate | 5405.6 (3942.3 to 7205.2) | 5617.8 (4061.5 to 7511.4) | 5205.3 (3764.7 to 6990.8) | 5009.4 (3635.1 to 6735.3) | 5470.3 (3927.5 to 7417.8) | 4574.4 (3331.4 to 6186.1) | -7.3 (-15.1 to 1.1) | -2.6 (-11.4 to 7.9) | -12.1 (-21.2 to -1.6) |
|  |  | YLLs (Years of Life Lost) | Number | 193146.8 (171780.2 to 214385.1) | 85642.7 (76095.1 to 95351.4) | 107504.1 (94285 to 120409.3) | 13465.9 (11925.3 to 15233.6) | 5140.5 (4550.8 to 5802) | 8325.4 (7258 to 9399.1) | -93 (-94.2 to -91.7) | -94 (-94.9 to -92.8) | -92.3 (-93.6 to -90.7) |
|  |  |  | Rate | 35566.4 (31631.9 to 39477.3) | 32482.7 (28861.5 to 36165) | 38476.3 (33745.1 to 43095.1) | 3529.9 (3126.1 to 3993.3) | 2775.2 (2456.8 to 3132.2) | 4242.3 (3698.4 to 4789.5) | -90.1 (-91.7 to -88.1) | -91.5 (-92.8 to -89.8) | -89 (-91 to -86.7) |
|  | Communicable, maternal, neonatal, and nutritional diseases | DALYs (Disability-Adjusted Life Years) | Number | 99581 (84982 to 121725.1) | 44835.1 (37653.6 to 57951.1) | 54745.9 (46103.9 to 66899.9) | 7838.9 (6516.6 to 9502.3) | 3793.5 (3041.9 to 4730.9) | 4045.4 (3374.5 to 4931.9) | -92.1 (-94.1 to -89.8) | -91.5 (-94 to -88.8) | -92.6 (-94.6 to -90.5) |
|  |  |  | Rate | 18337 (15648.7 to 22414.7) | 17005.1 (14281.3 to 21979.8) | 19593.8 (16500.8 to 23943.8) | 2054.9 (1708.2 to 2490.9) | 2047.9 (1642.2 to 2554) | 2061.4 (1719.6 to 2513.2) | -88.8 (-91.6 to -85.5) | -88 (-91.4 to -84.1) | -89.5 (-92.3 to -86.4) |
|  |  | YLDs (Years Lived with Disability) | Number | 7308.3 (4905.5 to 10363.3) | 3575.4 (2360.4 to 5171.5) | 3732.8 (2400.3 to 5501.4) | 3490.6 (2417.3 to 4974.8) | 1900.5 (1281.3 to 2796.9) | 1590.1 (1053.3 to 2284.7) | -52.2 (-62.8 to -38) | -46.8 (-58.9 to -27.5) | -57.4 (-70.5 to -40.8) |
|  |  |  | Rate | 1345.8 (903.3 to 1908.3) | 1356.1 (895.2 to 1961.5) | 1336 (859.1 to 1969) | 915 (633.7 to 1304.1) | 1026 (691.7 to 1509.9) | 810.3 (536.7 to 1164.2) | -32 (-47 to -11.8) | -24.3 (-41.6 to 3.2) | -39.4 (-58.1 to -15.7) |
|  |  | YLLs (Years of Life Lost) | Number | 92272.7 (77630 to 114026.7) | 41259.7 (34140.8 to 54317.4) | 51013 (42213.7 to 63232.9) | 4348.3 (3637.2 to 5103.5) | 1893 (1538.6 to 2279.2) | 2455.3 (2031.2 to 2887.8) | -95.3 (-96.6 to -93.9) | -95.4 (-97 to -94) | -95.2 (-96.7 to -93.7) |
|  |  |  | Rate | 16991.3 (14294.9 to 20997.1) | 15649.1 (12949 to 20601.6) | 18257.8 (15108.5 to 22631.4) | 1139.9 (953.4 to 1337.8) | 1021.9 (830.6 to 1230.5) | 1251.2 (1035 to 1471.5) | -93.3 (-95.2 to -91.3) | -93.5 (-95.7 to -91.5) | -93.1 (-95.2 to -91.1) |
|  | Injuries | DALYs (Disability-Adjusted Life Years) | Number | 45138.3 (38788.6 to 52183.3) | 17829.1 (14377.4 to 21838.6) | 27309.1 (23377.9 to 32075.6) | 4915.1 (4220.6 to 5572.7) | 1432.3 (1258 to 1639.8) | 3482.7 (2892.9 to 4088.8) | -89.1 (-91.3 to -86.4) | -92 (-93.9 to -89.6) | -87.2 (-90.1 to -84) |
|  |  |  | Rate | 8311.8 (7142.6 to 9609.1) | 6762.3 (5453.1 to 8283) | 9774.1 (8367.1 to 11480) | 1288.4 (1106.4 to 1460.8) | 773.3 (679.2 to 885.3) | 1774.7 (1474.1 to 2083.5) | -84.5 (-87.6 to -80.6) | -88.6 (-91.3 to -85.2) | -81.8 (-86 to -77.2) |
|  |  | YLDs (Years Lived with Disability) | Number | 2113.3 (1578.1 to 2787.8) | 952 (708.9 to 1248.3) | 1161.2 (858.5 to 1539.5) | 539 (390.6 to 728.3) | 246.6 (178.5 to 332.5) | 292.3 (209.8 to 394.7) | -74.5 (-76.9 to -72.1) | -74.1 (-76.8 to -71.1) | -74.8 (-77.5 to -72.2) |
|  |  |  | Rate | 389.1 (290.6 to 513.3) | 361.1 (268.9 to 473.5) | 415.6 (307.2 to 551) | 141.3 (102.4 to 190.9) | 133.2 (96.4 to 179.5) | 149 (106.9 to 201.1) | -63.7 (-67.1 to -60.3) | -63.1 (-67 to -58.8) | -64.2 (-68 to -60.5) |
|  |  | YLLs (Years of Life Lost) | Number | 43025 (36715.3 to 49963.6) | 16877.1 (13520 to 20807.9) | 26147.9 (22227.6 to 30923.9) | 4376.1 (3736.7 to 5051.6) | 1185.7 (1024.2 to 1357.9) | 3190.4 (2626.9 to 3775.5) | -89.8 (-92.1 to -87.1) | -93 (-94.8 to -90.7) | -87.8 (-90.7 to -84.5) |
|  |  |  | Rate | 7922.7 (6760.8 to 9200.4) | 6401.2 (5127.9 to 7892.1) | 9358.5 (7955.4 to 11067.8) | 1147.1 (979.5 to 1324.2) | 640.1 (552.9 to 733.1) | 1625.7 (1338.6 to 1923.9) | -85.5 (-88.7 to -81.7) | -90 (-92.6 to -86.8) | -82.6 (-86.8 to -77.9) |
|  | Non-communicable diseases | DALYs (Disability-Adjusted Life Years) | Number | 77783 (59612.2 to 92309.2) | 37790.1 (26660.4 to 45039.4) | 39993 (28494.6 to 48331.8) | 19727.2 (15351.9 to 25108.9) | 10030.4 (7643.7 to 13012.1) | 9696.8 (7630.4 to 12222.9) | -74.6 (-80.3 to -65.2) | -73.5 (-79.8 to -60) | -75.8 (-81.6 to -63.7) |
|  |  |  | Rate | 14323.1 (10977.1 to 16998) | 14333.1 (10111.8 to 17082.6) | 14313.7 (10198.3 to 17298.2) | 5171.2 (4024.3 to 6582) | 5415 (4126.5 to 7024.7) | 4941.2 (3888.2 to 6228.4) | -63.9 (-72 to -50.5) | -62.2 (-71.3 to -43.1) | -65.5 (-73.9 to -48.3) |
|  |  | YLDs (Years Lived with Disability) | Number | 19933.9 (14272.5 to 26437.2) | 10284.2 (7477.9 to 13824.3) | 9649.7 (6951.5 to 12782.8) | 15080.2 (10868.5 to 20317.4) | 7985.6 (5687.2 to 10856.3) | 7094.6 (5141.4 to 9613.8) | -24.3 (-29.9 to -18.2) | -22.4 (-28.4 to -15.1) | -26.5 (-32.5 to -20.2) |
|  |  |  | Rate | 3670.7 (2628.2 to 4868.2) | 3900.6 (2836.3 to 5243.3) | 3453.7 (2488 to 4575) | 3953.1 (2849 to 5325.9) | 4311.1 (3070.3 to 5860.8) | 3615.2 (2619.9 to 4898.9) | 7.7 (-0.2 to 16.5) | 10.5 (1.9 to 20.8) | 4.7 (-3.9 to 13.6) |
|  |  | YLLs (Years of Life Lost) | Number | 57849.1 (39212.7 to 70618.8) | 27505.9 (16868.5 to 33510) | 30343.2 (18695.2 to 38452.4) | 4647 (3965.7 to 5591.1) | 2044.8 (1721.3 to 2475.6) | 2602.2 (2129.3 to 3267.3) | -92 (-94 to -86.8) | -92.6 (-94.4 to -85.8) | -91.4 (-93.7 to -83.8) |
|  |  |  | Rate | 10652.4 (7220.7 to 13003.9) | 10432.5 (6397.9 to 12709.7) | 10860 (6691.1 to 13762.3) | 1218.2 (1039.6 to 1465.6) | 1103.9 (929.2 to 1336.5) | 1326 (1085 to 1664.9) | -88.6 (-91.5 to -81.2) | -89.4 (-92 to -79.9) | -87.8 (-91.1 to -76.9) |
| Qom | All causes | DALYs (Disability-Adjusted Life Years) | Number | 135176.3 (119721.9 to 152422.5) | 59333 (52199.9 to 67115.2) | 75843.3 (66977 to 85389.3) | 37791.9 (31177.6 to 45611.4) | 18030.8 (14383 to 22498.2) | 19761.1 (16730.4 to 23379.7) | -72 (-77.2 to -66.7) | -69.6 (-75.5 to -63.2) | -73.9 (-78.7 to -68.8) |
|  |  |  | Rate | 32796.1 (29046.6 to 36980.3) | 29398.5 (25864.1 to 33254.4) | 36056 (31840.9 to 40594.1) | 8215.2 (6777.3 to 9914.9) | 7997.1 (6379.2 to 9978.5) | 8424.8 (7132.7 to 9967.5) | -75 (-79.6 to -70.1) | -72.8 (-78 to -67.1) | -76.6 (-80.9 to -72) |
|  |  | YLDs (Years Lived with Disability) | Number | 22715 (16552.2 to 29925.2) | 11457.6 (8335.6 to 15154.4) | 11257.4 (8171.1 to 14926.2) | 23134 (17032 to 31147.8) | 12354.2 (9036.5 to 16544.4) | 10779.8 (7972 to 14366.4) | 1.8 (-6.2 to 10.5) | 7.8 (-2.4 to 17.2) | -4.2 (-13.8 to 5.9) |
|  |  |  | Rate | 5511.1 (4015.9 to 7260.4) | 5677.1 (4130.2 to 7508.7) | 5351.8 (3884.5 to 7095.9) | 5028.8 (3702.4 to 6770.9) | 5479.4 (4007.9 to 7337.8) | 4595.8 (3398.7 to 6124.9) | -8.7 (-16 to -1) | -3.5 (-12.7 to 4.9) | -14.1 (-22.7 to -5.1) |
|  |  | YLLs (Years of Life Lost) | Number | 112461.3 (98223.2 to 128326.1) | 47875.4 (41473 to 54473.9) | 64585.9 (56352.9 to 73972.5) | 14657.9 (13034.1 to 16372.9) | 5676.6 (4977.8 to 6461.5) | 8981.3 (7935.4 to 10095) | -87 (-89.3 to -83.9) | -88.1 (-90.4 to -85.3) | -86.1 (-88.8 to -82.9) |
|  |  |  | Rate | 27285 (23830.6 to 31134.1) | 23721.4 (20549.2 to 26990.9) | 30704.2 (26790.2 to 35166.6) | 3186.3 (2833.3 to 3559.1) | 2517.7 (2207.8 to 2865.8) | 3829 (3383.1 to 4303.8) | -88.3 (-90.5 to -85.6) | -89.4 (-91.4 to -86.9) | -87.5 (-89.9 to -84.7) |
|  | Communicable, maternal, neonatal, and nutritional diseases | DALYs (Disability-Adjusted Life Years) | Number | 61564.3 (50954.9 to 74974.8) | 27360.9 (22650.6 to 34439.5) | 34203.4 (28327.7 to 42152.7) | 8849.8 (7241.2 to 10894.7) | 4409.9 (3563.9 to 5581.2) | 4439.9 (3625.1 to 5416.1) | -85.6 (-89.1 to -81.3) | -83.9 (-88.3 to -78.5) | -87 (-90.5 to -83) |
|  |  |  | Rate | 14936.5 (12362.5 to 18190.2) | 13556.8 (11223 to 17064.2) | 16260.3 (13467 to 20039.4) | 1923.8 (1574.1 to 2368.3) | 1955.9 (1580.7 to 2475.4) | 1892.9 (1545.5 to 2309.1) | -87.1 (-90.2 to -83.2) | -85.6 (-89.5 to -80.8) | -88.4 (-91.5 to -84.8) |
|  |  | YLDs (Years Lived with Disability) | Number | 5717.7 (3939 to 8194.7) | 2827.4 (1834.6 to 4167.6) | 2890.3 (1925.3 to 4274.1) | 4176 (2968.5 to 6056.5) | 2310.2 (1596.8 to 3411.1) | 1865.9 (1274.8 to 2712.5) | -27 (-42 to -6.3) | -18.3 (-38.2 to 9.3) | -35.4 (-52.4 to -9.8) |
|  |  |  | Rate | 1387.2 (955.7 to 1988.2) | 1400.9 (909 to 2065) | 1374 (915.3 to 2031.9) | 907.8 (645.3 to 1316.6) | 1024.6 (708.2 to 1512.9) | 795.5 (543.5 to 1156.4) | -34.6 (-48 to -16.1) | -26.9 (-44.7 to -2.1) | -42.1 (-57.3 to -19.1) |
|  |  | YLLs (Years of Life Lost) | Number | 55846.5 (45727.1 to 69109.2) | 24533.4 (20034.4 to 31353.3) | 31313.1 (25393.9 to 38930.9) | 4673.8 (3776 to 5558.9) | 2099.7 (1668.4 to 2542) | 2574 (2035.2 to 3066.5) | -91.6 (-94.1 to -88.8) | -91.4 (-94.3 to -88.5) | -91.8 (-94.4 to -88.9) |
|  |  |  | Rate | 13549.3 (11094.2 to 16767.1) | 12155.9 (9926.7 to 15535) | 14886.3 (12072.3 to 18507.8) | 1016 (820.8 to 1208.4) | 931.3 (740 to 1127.5) | 1097.4 (867.7 to 1307.3) | -92.5 (-94.7 to -90) | -92.3 (-94.9 to -89.7) | -92.6 (-95 to -90) |
|  | Injuries | DALYs (Disability-Adjusted Life Years) | Number | 22317.7 (19272.6 to 26377.1) | 7259.2 (6029.9 to 8677.4) | 15058.6 (12872 to 18102.1) | 4762.4 (4267.1 to 5401.1) | 1317.7 (1145.6 to 1577.6) | 3444.7 (3007.4 to 3928.3) | -78.7 (-82.4 to -74.3) | -81.8 (-85.9 to -76.8) | -77.1 (-81.9 to -72) |
|  |  |  | Rate | 5414.7 (4675.9 to 6399.5) | 3596.8 (2987.7 to 4299.5) | 7158.8 (6119.4 to 8605.8) | 1035.3 (927.6 to 1174.1) | 584.4 (508.1 to 699.7) | 1468.6 (1282.1 to 1674.7) | -80.9 (-84.3 to -76.9) | -83.8 (-87.4 to -79.2) | -79.5 (-83.7 to -74.9) |
|  |  | YLDs (Years Lived with Disability) | Number | 1718.2 (1282.9 to 2254.9) | 695 (519.7 to 919.9) | 1023.2 (759.3 to 1344.4) | 630.1 (448.4 to 858.3) | 272.1 (196.5 to 366) | 358 (252.6 to 489.1) | -63.3 (-67.9 to -59.1) | -60.8 (-65.6 to -56.6) | -65 (-70.1 to -60.4) |
|  |  |  | Rate | 416.9 (311.3 to 547.1) | 344.4 (257.5 to 455.8) | 486.4 (361 to 639.1) | 137 (97.5 to 186.6) | 120.7 (87.1 to 162.3) | 152.6 (107.7 to 208.5) | -67.1 (-71.2 to -63.4) | -65 (-69.2 to -61.2) | -68.6 (-73.2 to -64.5) |
|  |  | YLLs (Years of Life Lost) | Number | 20599.6 (17459.4 to 24464.5) | 6564.2 (5352.4 to 7901.6) | 14035.4 (11838.1 to 17078.3) | 4132.3 (3678.3 to 4681.3) | 1045.6 (898.2 to 1296) | 3086.7 (2656.7 to 3536) | -79.9 (-84 to -75.2) | -84.1 (-87.9 to -78.8) | -78 (-82.9 to -72.7) |
|  |  |  | Rate | 4997.8 (4235.9 to 5935.5) | 3252.4 (2652 to 3915.1) | 6672.4 (5627.8 to 8119.1) | 898.3 (799.6 to 1017.6) | 463.7 (398.4 to 574.8) | 1316 (1132.6 to 1507.5) | -82 (-85.7 to -77.8) | -85.7 (-89.2 to -81) | -80.3 (-84.7 to -75.5) |
|  | Non-communicable diseases | DALYs (Disability-Adjusted Life Years) | Number | 51294.3 (40764.4 to 60265.5) | 24713 (18307.3 to 29491.6) | 26581.4 (20057.4 to 31836) | 23982.3 (18813.6 to 30200.5) | 12244.5 (9486.9 to 15695.2) | 11737.8 (9321.6 to 14611.8) | -53.2 (-62.9 to -37.4) | -50.5 (-61.1 to -31.5) | -55.8 (-65.5 to -38.4) |
|  |  |  | Rate | 12444.9 (9890.1 to 14621.4) | 12244.9 (9070.9 to 14612.6) | 12636.8 (9535.3 to 15134.9) | 5213.3 (4089.7 to 6565) | 5430.7 (4207.7 to 6961.2) | 5004.2 (3974.1 to 6229.5) | -58.1 (-66.7 to -43.9) | -55.6 (-65.1 to -38.7) | -60.4 (-69.1 to -44.7) |
|  |  | YLDs (Years Lived with Disability) | Number | 15279.1 (11235 to 20260.3) | 7935.2 (5777.6 to 10529.3) | 7343.9 (5398.4 to 9704) | 18327.8 (13318.6 to 24535.7) | 9771.9 (7123.9 to 13071.2) | 8555.9 (6223.8 to 11434.5) | 20 (10.5 to 29.5) | 23.1 (13 to 34) | 16.5 (6.4 to 26.7) |
|  |  |  | Rate | 3707 (2725.8 to 4915.5) | 3931.8 (2862.7 to 5217.1) | 3491.3 (2566.4 to 4613.3) | 3984.1 (2895.2 to 5333.5) | 4334.1 (3159.6 to 5797.4) | 3647.7 (2653.4 to 4874.9) | 7.5 (-1 to 16) | 10.2 (1.1 to 19.9) | 4.5 (-4.6 to 13.6) |
|  |  | YLLs (Years of Life Lost) | Number | 36015.2 (25534.5 to 43359.7) | 16777.8 (10783.7 to 20574.3) | 19237.4 (13213.4 to 23913.3) | 5654.5 (4793.2 to 6646.1) | 2472.6 (1994.9 to 2905.1) | 3181.9 (2692 to 3875.5) | -84.3 (-87.9 to -75.2) | -85.3 (-88.9 to -74.4) | -83.5 (-87.7 to -72.1) |
|  |  |  | Rate | 8737.9 (6195.1 to 10519.8) | 8313.1 (5343.2 to 10194.2) | 9145.5 (6281.6 to 11368.4) | 1229.2 (1041.9 to 1444.7) | 1096.7 (884.8 to 1288.5) | 1356.5 (1147.7 to 1652.3) | -85.9 (-89.1 to -77.8) | -86.8 (-90 to -77.1) | -85.2 (-89 to -75) |
| Semnan | All causes | DALYs (Disability-Adjusted Life Years) | Number | 78342.9 (69159.6 to 89353.7) | 33877.4 (29794 to 38682.8) | 44465.5 (39482.2 to 50450.5) | 18675.8 (15305.9 to 22175.3) | 8721.1 (6951.6 to 10620.8) | 9954.7 (8198.1 to 11684.7) | -76.2 (-80.9 to -71.4) | -74.3 (-79.4 to -69.1) | -77.6 (-81.9 to -73.3) |
|  |  |  | Rate | 32941.1 (29079.8 to 37570.8) | 29615.5 (26045.9 to 33816.4) | 36022.9 (31985.8 to 40871.6) | 8890.6 (7286.4 to 10556.6) | 8546.4 (6812.4 to 10408.1) | 9215.8 (7589.5 to 10817.3) | -73 (-78.4 to -67.6) | -71.1 (-76.9 to -65.4) | -74.4 (-79.4 to -69.4) |
|  |  | YLDs (Years Lived with Disability) | Number | 12988 (9635.3 to 17264.2) | 6377.1 (4686.3 to 8555.5) | 6610.9 (4893.1 to 8867.7) | 10754 (7800.2 to 14248) | 5614.3 (4048.4 to 7510.1) | 5139.8 (3756 to 6815.8) | -17.2 (-23.8 to -10) | -12 (-19.7 to -4) | -22.3 (-30.9 to -13.5) |
|  |  |  | Rate | 5461.1 (4051.4 to 7259.1) | 5574.9 (4096.7 to 7479.2) | 5355.7 (3964 to 7184) | 5119.5 (3713.3 to 6782.8) | 5501.8 (3967.3 to 7359.7) | 4758.3 (3477.2 to 6309.9) | -6.3 (-13.7 to 2) | -1.3 (-10 to 7.6) | -11.2 (-21.1 to -1.1) |
|  |  | YLLs (Years of Life Lost) | Number | 65354.9 (57033.4 to 75208.8) | 27500.3 (23740 to 31923.1) | 37854.6 (33115.1 to 43264.6) | 7921.8 (6540.1 to 9416.9) | 3106.8 (2498.7 to 3727.5) | 4815 (3946.9 to 5707.4) | -87.9 (-90.8 to -84.7) | -88.7 (-91.6 to -85.4) | -87.3 (-90.2 to -84.2) |
|  |  |  | Rate | 27480 (23981 to 31623.3) | 24040.7 (20753.4 to 27907.1) | 30667.2 (26827.6 to 35050.1) | 3771.2 (3113.4 to 4482.9) | 3044.6 (2448.7 to 3652.8) | 4457.5 (3653.9 to 5283.7) | -86.3 (-89.6 to -82.7) | -87.3 (-90.6 to -83.7) | -85.5 (-88.9 to -81.9) |
|  | Communicable, maternal, neonatal, and nutritional diseases | DALYs (Disability-Adjusted Life Years) | Number | 35305.5 (29783.9 to 43024.6) | 15494.6 (12837.3 to 19792.4) | 19810.8 (16579.4 to 24470.9) | 4789.4 (3878.4 to 5749) | 2300.6 (1834.3 to 2848.5) | 2488.8 (2006.4 to 3031.6) | -86.4 (-90.3 to -82.4) | -85.2 (-89.6 to -80.5) | -87.4 (-91.1 to -83.8) |
|  |  |  | Rate | 14845 (12523.3 to 18090.7) | 13545.4 (11222.4 to 17302.5) | 16049.4 (13431.5 to 19824.7) | 2280 (1846.3 to 2736.8) | 2254.5 (1797.6 to 2791.4) | 2304.1 (1857.4 to 2806.5) | -84.6 (-89 to -80.1) | -83.4 (-88.4 to -78.1) | -85.6 (-89.8 to -81.4) |
|  |  | YLDs (Years Lived with Disability) | Number | 3016.9 (2070 to 4255.7) | 1449.6 (951.5 to 2097.4) | 1567.2 (996.3 to 2306.7) | 1933.9 (1342.2 to 2752.5) | 1048 (713.2 to 1524.1) | 885.9 (590.9 to 1274.5) | -35.9 (-49.9 to -18.1) | -27.7 (-45.6 to -5.5) | -43.5 (-60.2 to -18.6) |
|  |  |  | Rate | 1268.5 (870.4 to 1789.4) | 1267.3 (831.8 to 1833.6) | 1269.7 (807.1 to 1868.7) | 920.6 (639 to 1310.3) | 1027 (698.9 to 1493.5) | 820.1 (547 to 1179.9) | -27.4 (-43.3 to -7.2) | -19 (-39 to 6) | -35.4 (-54.5 to -6.9) |
|  |  | YLLs (Years of Life Lost) | Number | 32288.6 (26903.7 to 40118.3) | 14045 (11650.7 to 18188) | 18243.6 (15148.6 to 22706.6) | 2855.5 (2197.2 to 3573.1) | 1252.6 (959.8 to 1567.3) | 1602.9 (1220.2 to 2013.7) | -91.2 (-94.1 to -88) | -91.1 (-94.2 to -87.7) | -91.2 (-94.2 to -88) |
|  |  |  | Rate | 13576.5 (11312.3 to 16868.7) | 12278.1 (10185 to 15899.9) | 14779.7 (12272.4 to 18395.3) | 1359.4 (1046 to 1701) | 1227.5 (940.5 to 1535.9) | 1484 (1129.6 to 1864.3) | -90 (-93.3 to -86.4) | -90 (-93.5 to -86.2) | -90 (-93.4 to -86.3) |
|  | Injuries | DALYs (Disability-Adjusted Life Years) | Number | 14542.6 (12907.9 to 16534.5) | 4919.9 (4168.3 to 5788.6) | 9622.7 (8443.4 to 10990.7) | 2671.7 (2244.1 to 3147) | 801.3 (684.9 to 954.9) | 1870.3 (1535 to 2227.8) | -81.6 (-85.2 to -77.1) | -83.7 (-87.5 to -79.4) | -80.6 (-84.9 to -75.5) |
|  |  |  | Rate | 6114.8 (5427.4 to 6952.3) | 4300.9 (3643.9 to 5060.4) | 7795.7 (6840.2 to 8904) | 1271.9 (1068.3 to 1498.1) | 785.3 (671.2 to 935.8) | 1731.5 (1421 to 2062.4) | -79.2 (-83.3 to -74.1) | -81.7 (-86 to -76.9) | -77.8 (-82.7 to -72) |
|  |  | YLDs (Years Lived with Disability) | Number | 1004.3 (749.8 to 1331.1) | 410.5 (306.3 to 548.1) | 593.9 (443.3 to 786.3) | 299.7 (218.2 to 401.5) | 134.8 (97.9 to 181.2) | 164.8 (117.8 to 224.9) | -70.2 (-73.6 to -66.8) | -67.1 (-70.9 to -63.3) | -72.2 (-75.9 to -68.7) |
|  |  |  | Rate | 422.3 (315.3 to 559.7) | 358.9 (267.8 to 479.2) | 481.1 (359.1 to 637) | 142.7 (103.9 to 191.1) | 132.1 (95.9 to 177.6) | 152.6 (109.1 to 208.2) | -66.2 (-70.1 to -62.5) | -63.2 (-67.4 to -58.9) | -68.3 (-72.5 to -64.3) |
|  |  | YLLs (Years of Life Lost) | Number | 13538.3 (11897.3 to 15604.4) | 4509.4 (3765.4 to 5403.9) | 9028.9 (7867.2 to 10374.5) | 2372 (1971.1 to 2844) | 666.5 (545.9 to 807.7) | 1705.5 (1371.9 to 2069.2) | -82.5 (-86.3 to -77.7) | -85.2 (-89.1 to -80.6) | -81.1 (-85.6 to -75.8) |
|  |  |  | Rate | 5692.5 (5002.5 to 6561.2) | 3942.1 (3291.7 to 4724.1) | 7314.6 (6373.5 to 8404.7) | 1129.2 (938.3 to 1353.9) | 653.1 (535 to 791.5) | 1578.9 (1270.1 to 1915.6) | -80.2 (-84.5 to -74.8) | -83.4 (-87.7 to -78.2) | -78.4 (-83.6 to -72.4) |
|  | Non-communicable diseases | DALYs (Disability-Adjusted Life Years) | Number | 28494.8 (23056.3 to 33986.2) | 13462.9 (9944.1 to 16204.7) | 15031.9 (11412.6 to 18032.5) | 11173.7 (8675.7 to 14015.7) | 5615.3 (4345.1 to 7080.3) | 5558.4 (4325.7 to 6965.4) | -60.8 (-68.9 to -47.6) | -58.3 (-67.5 to -40.7) | -63 (-71.3 to -48.8) |
|  |  |  | Rate | 11981.3 (9694.6 to 14290.3) | 11769.2 (8693.1 to 14166.1) | 12177.9 (9245.7 to 14608.7) | 5319.2 (4130.1 to 6672.2) | 5502.8 (4258.1 to 6938.5) | 5145.8 (4004.6 to 6448.4) | -55.6 (-64.8 to -40.7) | -53.2 (-63.6 to -33.5) | -57.7 (-67.2 to -41.5) |
|  |  | YLDs (Years Lived with Disability) | Number | 8966.8 (6537.4 to 11996.9) | 4517 (3246 to 6053.4) | 4449.8 (3270.2 to 5975.4) | 8520.5 (6102.6 to 11352.1) | 4431.4 (3141.8 to 5897.9) | 4089.1 (2955.2 to 5466.3) | -5 (-11.8 to 2.5) | -1.9 (-9.1 to 5.8) | -8.1 (-15.5 to -0.1) |
|  |  |  | Rate | 3770.3 (2748.8 to 5044.4) | 3948.7 (2837.6 to 5291.8) | 3604.9 (2649.3 to 4840.9) | 4056.2 (2905.2 to 5404.2) | 4342.7 (3078.9 to 5779.8) | 3785.6 (2735.9 to 5060.5) | 7.6 (-0.1 to 16.1) | 10 (1.9 to 18.6) | 5 (-3.4 to 14.2) |
|  |  | YLLs (Years of Life Lost) | Number | 19528 (13868.8 to 23689.5) | 8945.9 (5552.9 to 10929.1) | 10582.1 (7085.4 to 13154.7) | 2653.2 (2048 to 3345) | 1183.9 (893.3 to 1522.2) | 1469.3 (1132.1 to 1898.5) | -86.4 (-90.3 to -78.1) | -86.8 (-91 to -74.4) | -86.1 (-90.3 to -75.8) |
|  |  |  | Rate | 8211 (5831.4 to 9960.8) | 7820.5 (4854.3 to 9554.2) | 8572.9 (5740.1 to 10657.1) | 1263 (974.9 to 1592.4) | 1160.2 (875.5 to 1491.8) | 1360.2 (1048 to 1757.5) | -84.6 (-89.1 to -75.2) | -85.2 (-90 to -71.3) | -84.1 (-88.9 to -72.4) |
| Sistan and Baluchistan | All causes | DALYs (Disability-Adjusted Life Years) | Number | 634066.7 (569308.9 to 704246.1) | 274869.8 (246694.9 to 305766.6) | 359196.9 (322584.3 to 399798.8) | 196237.5 (171542.9 to 224481.8) | 88025.7 (75489.1 to 102553) | 108211.9 (95738 to 122906.6) | -69.1 (-73.4 to -64.2) | -68 (-73.1 to -62.5) | -69.9 (-73.9 to -65.2) |
|  |  |  | Rate | 71049.3 (63793 to 78913.1) | 61834.1 (55496 to 68784.6) | 80195 (72020.8 to 89259.9) | 12784.5 (11175.7 to 14624.6) | 11736.7 (10065.2 to 13673.7) | 13785.6 (12196.5 to 15657.6) | -82 (-84.5 to -79.2) | -81 (-84 to -77.8) | -82.8 (-85.1 to -80.1) |
|  |  | YLDs (Years Lived with Disability) | Number | 52298.6 (38000.3 to 70538.4) | 25937 (18849.2 to 34795.1) | 26361.6 (18930.2 to 35505.9) | 79685.1 (58387.5 to 107629.8) | 41277.9 (30123.5 to 55878.7) | 38407.3 (28337.1 to 51932.4) | 52.4 (38.6 to 66.6) | 59.1 (44.6 to 75.2) | 45.7 (30.7 to 63.4) |
|  |  |  | Rate | 5860.2 (4258.1 to 7904.1) | 5834.7 (4240.3 to 7827.4) | 5885.5 (4226.4 to 7927.1) | 5191.3 (3803.8 to 7011.9) | 5503.7 (4016.5 to 7450.5) | 4892.9 (3610 to 6615.9) | -11.4 (-19.4 to -3.1) | -5.7 (-14.3 to 3.9) | -16.9 (-25.4 to -6.8) |
|  |  | YLLs (Years of Life Lost) | Number | 581768.1 (521231.6 to 647086.3) | 248932.7 (221315.4 to 278094.3) | 332835.3 (297120.6 to 371273) | 116552.4 (107759.5 to 125726.2) | 46747.8 (42820.7 to 51038.8) | 69804.6 (63808.5 to 75645) | -80 (-82.7 to -76.7) | -81.2 (-83.8 to -78.1) | -79 (-82.1 to -75.5) |
|  |  |  | Rate | 65189.1 (58405.8 to 72508.2) | 55999.4 (49786.7 to 62559.5) | 74309.5 (66335.7 to 82891.1) | 7593.2 (7020.3 to 8190.8) | 6233 (5709.4 to 6805.2) | 8892.7 (8128.9 to 9636.8) | -88.4 (-89.9 to -86.5) | -88.9 (-90.4 to -87) | -88 (-89.8 to -86) |
|  | Communicable, maternal, neonatal, and nutritional diseases | DALYs (Disability-Adjusted Life Years) | Number | 350306.5 (296995.3 to 424352.4) | 157741 (132359.4 to 192840.6) | 192565.5 (162454.6 to 234930.7) | 63051.4 (54505.5 to 72523.2) | 29301.7 (24900.3 to 33997.9) | 33749.7 (28969.1 to 39497.8) | -82 (-85.6 to -77.6) | -81.4 (-85.7 to -76.7) | -82.5 (-86.4 to -77.6) |
|  |  |  | Rate | 39253 (33279.3 to 47550.1) | 35485.1 (29775.3 to 43381) | 42992.6 (36269.9 to 52451.1) | 4107.7 (3550.9 to 4724.7) | 3906.9 (3320 to 4533) | 4299.5 (3690.5 to 5031.8) | -89.5 (-91.6 to -87) | -89 (-91.5 to -86.2) | -90 (-92.3 to -87.2) |
|  |  | YLDs (Years Lived with Disability) | Number | 15940.6 (11133.1 to 22038.1) | 7413.2 (4781.4 to 10502.8) | 8527.3 (5746.5 to 12129) | 18189.9 (12864.5 to 25101.5) | 9166.4 (6146 to 12788.3) | 9023.5 (5921.4 to 13157.3) | 14.1 (-7.4 to 45.2) | 23.6 (-3.3 to 58.3) | 5.8 (-22.7 to 47) |
|  |  |  | Rate | 1786.2 (1247.5 to 2469.4) | 1667.7 (1075.6 to 2362.7) | 1903.8 (1283 to 2707.9) | 1185 (838.1 to 1635.3) | 1222.2 (819.5 to 1705.1) | 1149.5 (754.4 to 1676.2) | -33.7 (-46.2 to -15.6) | -26.7 (-42.7 to -6.2) | -39.6 (-55.9 to -16.1) |
|  |  | YLLs (Years of Life Lost) | Number | 334366 (280421.2 to 405831.1) | 150327.8 (125344.7 to 184861.2) | 184038.2 (153819 to 226900.4) | 44861.5 (39134.5 to 51160.2) | 20135.3 (17082.7 to 23310.1) | 24726.3 (21353.8 to 28579.8) | -86.6 (-89.6 to -83) | -86.6 (-90.1 to -82.7) | -86.6 (-89.7 to -82.8) |
|  |  |  | Rate | 37466.8 (31422.1 to 45474.7) | 33817.4 (28197.3 to 41586) | 41088.7 (34341.9 to 50658.2) | 2922.6 (2549.5 to 3333) | 2684.7 (2277.7 to 3108) | 3150 (2720.4 to 3640.9) | -92.2 (-93.9 to -90.1) | -92.1 (-94.1 to -89.8) | -92.3 (-94.1 to -90.2) |
|  | Injuries | DALYs (Disability-Adjusted Life Years) | Number | 113424.2 (87386.3 to 134505.2) | 41847.1 (29702.1 to 52063.8) | 71577.1 (56688 to 85640.5) | 35673.9 (32258.5 to 39654.8) | 10869 (9687.9 to 12148.4) | 24805 (21900.3 to 28071.3) | -68.5 (-75.1 to -56.6) | -74 (-80.1 to -61.9) | -65.3 (-73.1 to -53.5) |
|  |  |  | Rate | 12709.6 (9791.9 to 15071.8) | 9413.8 (6681.7 to 11712.2) | 15980.4 (12656.3 to 19120.3) | 2324.1 (2101.6 to 2583.4) | 1449.2 (1291.7 to 1619.8) | 3160 (2790 to 3576.1) | -81.7 (-85.5 to -74.8) | -84.6 (-88.2 to -77.4) | -80.2 (-84.7 to -73.5) |
|  |  | YLDs (Years Lived with Disability) | Number | 3204 (2384.5 to 4209.3) | 1400.2 (1033.5 to 1845) | 1803.8 (1334.9 to 2371.1) | 2255.8 (1672.5 to 2974.7) | 999.5 (736.5 to 1332.7) | 1256.2 (921.3 to 1677.4) | -29.6 (-34.8 to -24.1) | -28.6 (-34.6 to -21.4) | -30.4 (-36.2 to -24.9) |
|  |  |  | Rate | 359 (267.2 to 471.7) | 315 (232.5 to 415) | 402.7 (298 to 529.4) | 147 (109 to 193.8) | 133.3 (98.2 to 177.7) | 160 (117.4 to 213.7) | -59.1 (-62.1 to -55.9) | -57.7 (-61.2 to -53.4) | -60.3 (-63.6 to -57.1) |
|  |  | YLLs (Years of Life Lost) | Number | 110220.1 (84444.6 to 131044.3) | 40446.9 (28232.6 to 50529.6) | 69773.3 (54719.8 to 84040.2) | 33418.2 (29940.3 to 37370.1) | 9869.5 (8712.4 to 11141.9) | 23548.7 (20623.9 to 26815.3) | -69.7 (-76.3 to -57.8) | -75.6 (-81.4 to -63.5) | -66.2 (-74.1 to -54.3) |
|  |  |  | Rate | 12350.5 (9462.3 to 14684) | 9098.8 (6351.1 to 11367) | 15577.7 (12216.8 to 18763) | 2177.1 (1950.6 to 2434.6) | 1315.9 (1161.7 to 1485.6) | 3000 (2627.4 to 3416.1) | -82.4 (-86.2 to -75.4) | -85.5 (-89 to -78.4) | -80.7 (-85.2 to -73.9) |
|  | Non-communicable diseases | DALYs (Disability-Adjusted Life Years) | Number | 170336 (114633.2 to 217200.9) | 75281.7 (48579.9 to 98422) | 95054.3 (59428.9 to 123667.9) | 96816.2 (78978.3 to 118207.3) | 47631.4 (38503.9 to 59166.8) | 49184.8 (40556.7 to 58780.1) | -43.2 (-57 to -11.7) | -36.7 (-52.7 to -0.3) | -48.3 (-61 to -11.6) |
|  |  |  | Rate | 19086.7 (12845 to 24338.1) | 16935.2 (10928.4 to 22140.8) | 21222 (13268.2 to 27610.3) | 6307.4 (5145.3 to 7701) | 6350.8 (5133.8 to 7888.9) | 6265.9 (5166.7 to 7488.3) | -67 (-75 to -48.7) | -62.5 (-72 to -40.9) | -70.5 (-77.7 to -49.6) |
|  |  | YLDs (Years Lived with Disability) | Number | 33154 (23984.1 to 44210.9) | 17123.6 (12341.4 to 22948.3) | 16030.4 (11615.5 to 21475.2) | 59239.5 (43039.1 to 80075.1) | 31111.9 (22662.8 to 42364.9) | 28127.5 (20538.3 to 37948.9) | 78.7 (64.1 to 94.6) | 81.7 (65.8 to 99.2) | 75.5 (60.1 to 92.1) |
|  |  |  | Rate | 3715 (2687.5 to 4954) | 3852.1 (2776.3 to 5162.4) | 3579 (2593.3 to 4794.6) | 3859.3 (2803.9 to 5216.7) | 4148.2 (3021.7 to 5648.6) | 3583.3 (2616.5 to 4834.5) | 3.9 (-4.6 to 13.1) | 7.7 (-1.7 to 18.1) | 0.1 (-8.6 to 9.6) |
|  |  | YLLs (Years of Life Lost) | Number | 137182 (80402.8 to 183628.6) | 58158.1 (32121.6 to 81439.9) | 79023.9 (43066.1 to 107417.1) | 37576.8 (32793.6 to 42857.7) | 16519.4 (14363.5 to 19205.1) | 21057.3 (18077 to 24986.7) | -72.6 (-79.4 to -51.9) | -71.6 (-79.2 to -43.2) | -73.4 (-80.4 to -45.3) |
|  |  |  | Rate | 15371.7 (9009.4 to 20576.2) | 13083.1 (7226 to 18320.5) | 17643 (9615 to 23982.1) | 2448.1 (2136.4 to 2792.1) | 2202.6 (1915.1 to 2560.7) | 2682.6 (2302.9 to 3183.2) | -84.1 (-88 to -72) | -83.2 (-87.7 to -66.4) | -84.8 (-88.8 to -68.8) |
| South Khorasan | All causes | DALYs (Disability-Adjusted Life Years) | Number | 241195.1 (216761.6 to 267112.8) | 105542.8 (94278.4 to 117439.7) | 135652.3 (121158.5 to 149782.1) | 30450.6 (25557.2 to 35873.8) | 14078.9 (11594.4 to 16962.2) | 16371.7 (13973.1 to 18924) | -87.4 (-89.7 to -85) | -86.7 (-89.3 to -84) | -87.9 (-90.1 to -85.7) |
|  |  |  | Rate | 63413.1 (56989.3 to 70227.2) | 56774.9 (50715.4 to 63174.6) | 69759.2 (62305.7 to 77025.4) | 9507.9 (7980 to 11201.2) | 9136.6 (7524.2 to 11007.6) | 9852.2 (8408.8 to 11388.1) | -85 (-87.8 to -82.2) | -83.9 (-87.1 to -80.7) | -85.9 (-88.4 to -83.3) |
|  |  | YLDs (Years Lived with Disability) | Number | 21755.2 (15833.7 to 29001.1) | 10816.8 (7839.5 to 14441.4) | 10938.4 (7846.1 to 14725) | 16401.1 (11812.2 to 22029.3) | 8475.8 (6115.8 to 11353.9) | 7925.3 (5768.1 to 10620.7) | -24.6 (-31.4 to -17.7) | -21.6 (-28.8 to -14.3) | -27.5 (-36.2 to -19) |
|  |  |  | Rate | 5719.7 (4162.9 to 7624.8) | 5818.7 (4217.1 to 7768.5) | 5625.1 (4034.8 to 7572.4) | 5121.1 (3688.2 to 6878.4) | 5500.4 (3968.9 to 7368.2) | 4769.3 (3471.2 to 6391.4) | -10.5 (-18.6 to -2.3) | -5.5 (-14.1 to 3.4) | -15.2 (-25.3 to -5.3) |
|  |  | YLLs (Years of Life Lost) | Number | 219439.9 (193917.5 to 244498.8) | 94726 (83716.8 to 106247.6) | 124713.9 (110352.9 to 139269) | 14049.6 (12802 to 15437) | 5603.1 (5070.3 to 6201.3) | 8446.5 (7688.5 to 9259.7) | -93.6 (-94.5 to -92.5) | -94.1 (-95 to -93.1) | -93.2 (-94.3 to -92) |
|  |  |  | Rate | 57693.4 (50983.3 to 64281.7) | 50956.2 (45034 to 57154) | 64134.1 (56749 to 71619.1) | 4386.8 (3997.3 to 4820.1) | 3636.1 (3290.4 to 4024.4) | 5082.9 (4626.8 to 5572.4) | -92.4 (-93.5 to -91.1) | -92.9 (-94 to -91.7) | -92.1 (-93.3 to -90.7) |
|  | Communicable, maternal, neonatal, and nutritional diseases | DALYs (Disability-Adjusted Life Years) | Number | 129442.1 (109439 to 157633.3) | 58283.9 (48202.9 to 74111.6) | 71158.2 (59699.3 to 87899.4) | 8309.3 (6976.3 to 9915) | 3977.3 (3302.6 to 4792.1) | 4332 (3642.2 to 5184.3) | -93.6 (-95.2 to -92) | -93.2 (-95 to -91.4) | -93.9 (-95.5 to -92.3) |
|  |  |  | Rate | 34031.9 (28772.8 to 41443.7) | 31352.8 (25929.9 to 39867) | 36593.1 (30700.4 to 45202.3) | 2594.5 (2178.3 to 3095.9) | 2581.1 (2143.2 to 3109.8) | 2606.9 (2191.8 to 3119.8) | -92.4 (-94.3 to -90.5) | -91.8 (-94 to -89.6) | -92.9 (-94.8 to -91) |
|  |  | YLDs (Years Lived with Disability) | Number | 5761.2 (3860.3 to 8208.6) | 2731.1 (1740.5 to 3965) | 3030 (1935.6 to 4495.8) | 3106.8 (2131 to 4388.4) | 1651.4 (1106.7 to 2391) | 1455.4 (962 to 2159.4) | -46.1 (-57.4 to -29.5) | -39.5 (-53.3 to -18.6) | -52 (-66.4 to -31.1) |
|  |  |  | Rate | 1514.7 (1014.9 to 2158.1) | 1469.2 (936.3 to 2132.9) | 1558.2 (995.4 to 2312) | 970.1 (665.4 to 1370.2) | 1071.7 (718.2 to 1551.7) | 875.9 (578.9 to 1299.5) | -36 (-49.4 to -16.3) | -27.1 (-43.7 to -1.9) | -43.8 (-60.7 to -19.4) |
|  |  | YLLs (Years of Life Lost) | Number | 123680.9 (102885.2 to 150707.4) | 55552.7 (45631.1 to 71116.7) | 68128.2 (56397 to 84734.3) | 5202.5 (4444.5 to 6107.9) | 2325.9 (1905.5 to 2746.3) | 2876.5 (2365.3 to 3386.8) | -95.8 (-96.9 to -94.7) | -95.8 (-97.2 to -94.7) | -95.8 (-97.1 to -94.6) |
|  |  |  | Rate | 32517.2 (27049.8 to 39622.8) | 29883.6 (24546.4 to 38256) | 35034.9 (29002.1 to 43574.6) | 1624.4 (1387.8 to 1907.1) | 1509.4 (1236.6 to 1782.2) | 1731.1 (1423.4 to 2038.1) | -95 (-96.3 to -93.7) | -94.9 (-96.6 to -93.6) | -95.1 (-96.6 to -93.6) |
|  | Injuries | DALYs (Disability-Adjusted Life Years) | Number | 44123.7 (36843.7 to 52348.6) | 17231.8 (12971.2 to 20976.6) | 26891.8 (22700.4 to 32536.2) | 4599.1 (4102.5 to 5095.5) | 1452.8 (1286.6 to 1624.2) | 3146.3 (2775.8 to 3568.4) | -89.6 (-91.8 to -86.9) | -91.6 (-93.4 to -88.5) | -88.3 (-91.1 to -85.1) |
|  |  |  | Rate | 11600.7 (9686.7 to 13763.1) | 9269.6 (6977.6 to 11284) | 13829.1 (11673.7 to 16731.7) | 1436 (1281 to 1591) | 942.8 (835 to 1054) | 1893.4 (1670.4 to 2147.4) | -87.6 (-90.3 to -84.4) | -89.8 (-92 to -86.2) | -86.3 (-89.6 to -82.5) |
|  |  | YLDs (Years Lived with Disability) | Number | 1418.6 (1048.4 to 1872.2) | 661.5 (481.5 to 883.8) | 757.1 (558.1 to 994.4) | 448.8 (325.5 to 597.2) | 194.8 (139.9 to 258.7) | 253.9 (184.3 to 343.2) | -68.4 (-70.7 to -65.9) | -70.5 (-73.6 to -67.5) | -66.5 (-69.3 to -63.6) |
|  |  |  | Rate | 373 (275.6 to 492.2) | 355.8 (259 to 475.4) | 389.3 (287 to 511.4) | 140.1 (101.6 to 186.5) | 126.4 (90.8 to 167.9) | 152.8 (110.9 to 206.5) | -62.4 (-65.2 to -59.5) | -64.5 (-68.1 to -60.8) | -60.7 (-64 to -57.4) |
|  |  | YLLs (Years of Life Lost) | Number | 42705.1 (35375.7 to 50845.6) | 16570.3 (12305.3 to 20169) | 26134.8 (21843.9 to 31765) | 4150.3 (3696 to 4631.9) | 1257.9 (1113 to 1415.1) | 2892.3 (2530.1 to 3318.9) | -90.3 (-92.4 to -87.5) | -92.4 (-94.2 to -89.5) | -88.9 (-91.6 to -85.7) |
|  |  |  | Rate | 11227.7 (9300.7 to 13367.9) | 8913.7 (6619.4 to 10849.6) | 13439.8 (11233.2 to 16335.2) | 1295.9 (1154 to 1446.3) | 816.3 (722.3 to 918.3) | 1740.6 (1522.6 to 1997.3) | -88.5 (-91 to -85.2) | -90.8 (-93 to -87.4) | -87 (-90.2 to -83.3) |
|  | Non-communicable diseases | DALYs (Disability-Adjusted Life Years) | Number | 67629.3 (46328.7 to 86693.7) | 30027 (19577.1 to 39603.7) | 37602.2 (23931.1 to 47794.8) | 17424.3 (13521.3 to 21614.8) | 8614.3 (6610.4 to 10841.7) | 8810 (6924.6 to 10827.9) | -74.2 (-81 to -60.2) | -71.3 (-78.7 to -54.5) | -76.6 (-83.1 to -61.5) |
|  |  |  | Rate | 17780.6 (12180.4 to 22792.8) | 16152.5 (10531.2 to 21304.1) | 19336.9 (12306.5 to 24578.5) | 5440.6 (4221.9 to 6749) | 5590.3 (4289.8 to 7035.7) | 5301.7 (4167.1 to 6516.1) | -69.4 (-77.4 to -52.8) | -65.4 (-74.3 to -45.1) | -72.6 (-80.2 to -54.9) |
|  |  | YLDs (Years Lived with Disability) | Number | 14575.4 (10522.9 to 19266.2) | 7424.1 (5384.9 to 9907.7) | 7151.3 (5177.9 to 9413.2) | 12845.5 (9293 to 17131.2) | 6629.6 (4747.2 to 8894.8) | 6215.9 (4524.1 to 8332.4) | -11.9 (-18.8 to -4.1) | -10.7 (-18 to -2.4) | -13.1 (-20.3 to -5.2) |
|  |  |  | Rate | 3832.1 (2766.6 to 5065.3) | 3993.7 (2896.7 to 5329.7) | 3677.6 (2662.8 to 4840.7) | 4010.9 (2901.6 to 5349) | 4302.3 (3080.7 to 5772.3) | 3740.6 (2722.5 to 5014.3) | 4.7 (-3.5 to 13.9) | 7.7 (-1.1 to 17.8) | 1.7 (-6.8 to 10.9) |
|  |  | YLLs (Years of Life Lost) | Number | 53053.8 (32560.4 to 71557.2) | 22602.9 (12772.2 to 31818.4) | 30450.9 (17399.5 to 40540.2) | 4578.9 (3967.5 to 5477.7) | 1984.7 (1697.1 to 2394.1) | 2594.1 (2205.6 to 3307.4) | -91.4 (-93.7 to -84.1) | -91.2 (-93.8 to -82) | -91.5 (-93.9 to -82.3) |
|  |  |  | Rate | 13948.5 (8560.5 to 18813.3) | 12158.9 (6870.6 to 17116.1) | 15659.4 (8947.7 to 20847.8) | 1429.7 (1238.8 to 1710.4) | 1288 (1101.4 to 1553.7) | 1561.1 (1327.3 to 1990.3) | -89.8 (-92.5 to -81.1) | -89.4 (-92.5 to -78.3) | -90 (-92.8 to -79.3) |
| Tehran | All causes | DALYs (Disability-Adjusted Life Years) | Number | 835390.1 (716625.5 to 978601.9) | 362712 (311923 to 421148) | 472678.1 (402623.1 to 555567.1) | 292797.8 (237423.3 to 360099.9) | 148244.4 (118977.6 to 183462.8) | 144553.4 (118089.7 to 175623) | -65 (-71.4 to -57.5) | -59.1 (-66.4 to -51.3) | -69.4 (-75.4 to -62.7) |
|  |  |  | Rate | 21106 (18105.4 to 24724.2) | 18752.5 (16126.7 to 21773.7) | 23355.2 (19893.7 to 27450.7) | 8009.6 (6494.8 to 9850.7) | 8356.2 (6706.5 to 10341.4) | 7682.8 (6276.3 to 9334.1) | -62.1 (-69 to -53.9) | -55.4 (-63.3 to -46.9) | -67.1 (-73.6 to -59.9) |
|  |  | YLDs (Years Lived with Disability) | Number | 202714.6 (148402.9 to 269268.6) | 105750.3 (77419.8 to 140834.3) | 96964.3 (70851.7 to 128165.7) | 189179.7 (136977.3 to 255204.9) | 101632.4 (73025.9 to 136149.8) | 87547.2 (63048.9 to 117936.7) | -6.7 (-14.3 to 1.5) | -3.9 (-12.4 to 4.8) | -9.7 (-18.3 to -0.4) |
|  |  |  | Rate | 5121.5 (3749.4 to 6803) | 5467.4 (4002.7 to 7281.2) | 4791 (3500.8 to 6332.7) | 5175.1 (3747.1 to 6981.2) | 5728.8 (4116.3 to 7674.5) | 4653 (3350.9 to 6268.1) | 1 (-7.2 to 9.9) | 4.8 (-4.5 to 14.3) | -2.9 (-12.1 to 7.2) |
|  |  | YLLs (Years of Life Lost) | Number | 632675.5 (527280.7 to 752203.9) | 256961.7 (214387.3 to 303473.8) | 375713.8 (309468.7 to 449483.9) | 103618.2 (86684 to 124673.9) | 46612 (38330.3 to 55706.1) | 57006.2 (45699.9 to 69662.9) | -83.6 (-87.1 to -79.4) | -81.9 (-85.5 to -77.1) | -84.8 (-88.6 to -80.6) |
|  |  |  | Rate | 15984.4 (13321.6 to 19004.3) | 13285.1 (11084 to 15689.8) | 18564.1 (15290.9 to 22209.1) | 2834.5 (2371.3 to 3410.5) | 2627.4 (2160.6 to 3140) | 3029.8 (2428.9 to 3702.5) | -82.3 (-86.1 to -77.7) | -80.2 (-84.2 to -75.1) | -83.7 (-87.7 to -79.1) |
|  | Communicable, maternal, neonatal, and nutritional diseases | DALYs (Disability-Adjusted Life Years) | Number | 386151.3 (315383.5 to 484306.2) | 168705.7 (137434 to 211079.1) | 217445.6 (171400.3 to 276196.6) | 64204.2 (50420.4 to 80288.1) | 31906.5 (24646.7 to 40842.6) | 32297.7 (25409.3 to 40435.8) | -83.4 (-87.7 to -77.2) | -81.1 (-86.3 to -74.2) | -85.1 (-89.3 to -79.5) |
|  |  |  | Rate | 9756 (7968.1 to 12235.9) | 8722.2 (7105.4 to 10913) | 10744 (8468.9 to 13647) | 1756.3 (1379.3 to 2196.3) | 1798.5 (1389.3 to 2302.2) | 1716.6 (1350.5 to 2149.1) | -82 (-86.7 to -75.3) | -79.4 (-85 to -71.8) | -84 (-88.4 to -78) |
|  |  | YLDs (Years Lived with Disability) | Number | 45836 (30846.8 to 62855.3) | 24445.6 (16196.3 to 35217.4) | 21390.4 (13888 to 30978.3) | 31270.8 (21804.5 to 43697.2) | 17514.4 (11842 to 25513.1) | 13756.4 (9563.6 to 19887) | -31.8 (-45.4 to -13) | -28.4 (-44.7 to -4.5) | -35.7 (-53.3 to -10.4) |
|  |  |  | Rate | 1158 (779.3 to 1588) | 1263.9 (837.4 to 1820.8) | 1056.9 (686.2 to 1530.6) | 855.4 (596.5 to 1195.4) | 987.2 (667.5 to 1438.1) | 731.1 (508.3 to 1057) | -26.1 (-40.8 to -5.8) | -21.9 (-39.7 to 4.1) | -30.8 (-49.8 to -3.7) |
|  |  | YLLs (Years of Life Lost) | Number | 340315.4 (267629.4 to 433457.7) | 144260.1 (113562.6 to 189436.8) | 196055.2 (149418.4 to 251984.3) | 32933.4 (23739.2 to 43762.6) | 14392.1 (10311.4 to 19304.9) | 18541.3 (13366.9 to 24882.4) | -90.3 (-93.4 to -85.9) | -90 (-93.3 to -85.3) | -90.5 (-93.8 to -86.4) |
|  |  |  | Rate | 8598 (6761.6 to 10951.2) | 7458.4 (5871.3 to 9794) | 9687.1 (7382.8 to 12450.6) | 900.9 (649.4 to 1197.1) | 811.2 (581.2 to 1088.2) | 985.4 (710.4 to 1322.5) | -89.5 (-92.8 to -84.8) | -89.1 (-92.7 to -84) | -89.8 (-93.4 to -85.3) |
|  | Injuries | DALYs (Disability-Adjusted Life Years) | Number | 74221.9 (62557.2 to 88257.7) | 24733 (20586.2 to 30568.5) | 49488.9 (40177 to 61388.1) | 26019.6 (22895.1 to 30413.4) | 10500.8 (9095.3 to 13018.5) | 15518.8 (13208.7 to 18501.1) | -64.9 (-70.9 to -56.7) | -57.5 (-65 to -47.3) | -68.6 (-75.3 to -60) |
|  |  |  | Rate | 1875.2 (1580.5 to 2229.8) | 1278.7 (1064.3 to 1580.4) | 2445.3 (1985.2 to 3033.2) | 711.8 (626.3 to 832) | 591.9 (512.7 to 733.8) | 824.8 (702 to 983.3) | -62 (-68.5 to -53.1) | -53.7 (-61.8 to -42.5) | -66.3 (-73.4 to -57) |
|  |  | YLDs (Years Lived with Disability) | Number | 10838.7 (7987.8 to 14355.9) | 4766 (3483.5 to 6350.1) | 6072.7 (4422.5 to 8144.5) | 5510 (3934.1 to 7622.1) | 2453.9 (1773.1 to 3341) | 3056.1 (2158.4 to 4281.4) | -49.2 (-53 to -46.1) | -48.5 (-53.2 to -44) | -49.7 (-53.9 to -46.2) |
|  |  |  | Rate | 273.8 (201.8 to 362.7) | 246.4 (180.1 to 328.3) | 300.1 (218.5 to 402.4) | 150.7 (107.6 to 208.5) | 138.3 (99.9 to 188.3) | 162.4 (114.7 to 227.5) | -45 (-49.1 to -41.6) | -43.9 (-48.9 to -38.9) | -45.9 (-50.4 to -42.1) |
|  |  | YLLs (Years of Life Lost) | Number | 63383.2 (51703.1 to 77260.6) | 19967 (15709.4 to 25410.9) | 43416.2 (34225.4 to 54905.7) | 20509.7 (17880.3 to 24335.2) | 8046.9 (6823.4 to 10556.7) | 12462.7 (10022.2 to 15262.7) | -67.6 (-74.3 to -58) | -59.7 (-68 to -46.7) | -71.3 (-78.2 to -61.6) |
|  |  |  | Rate | 1601.4 (1306.3 to 1952) | 1032.3 (812.2 to 1313.8) | 2145.2 (1691.1 to 2712.9) | 561 (489.1 to 665.7) | 453.6 (384.6 to 595.1) | 662.4 (532.7 to 811.2) | -65 (-72.2 to -54.5) | -56.1 (-65.1 to -41.9) | -69.1 (-76.5 to -58.7) |
|  | Non-communicable diseases | DALYs (Disability-Adjusted Life Years) | Number | 375016.9 (306174.6 to 454568.5) | 169273.3 (133985.1 to 205428.7) | 205743.6 (163861.9 to 254805.5) | 201088 (157771.3 to 256487.1) | 105370.9 (81832.7 to 135361.6) | 95717.1 (75487.8 to 119641.2) | -46.4 (-57.1 to -33.3) | -37.8 (-49.1 to -21.9) | -53.5 (-63.6 to -38.9) |
|  |  |  | Rate | 9474.7 (7735.4 to 11484.6) | 8751.6 (6927.1 to 10620.8) | 10165.8 (8096.5 to 12590) | 5500.8 (4315.9 to 7016.3) | 5939.5 (4612.7 to 7630) | 5087.2 (4012 to 6358.7) | -41.9 (-53.6 to -27.8) | -32.1 (-44.5 to -14.9) | -50 (-60.9 to -34.3) |
|  |  | YLDs (Years Lived with Disability) | Number | 146040 (106175.1 to 194899.6) | 76538.7 (55404.6 to 102480.3) | 69501.3 (50331.9 to 92293.5) | 152398.9 (109582.4 to 204882.8) | 81664.2 (57945.5 to 110202.8) | 70734.7 (51340.5 to 95888.1) | 4.4 (-3.6 to 12.7) | 6.7 (-1.6 to 15.4) | 1.8 (-6.3 to 10.1) |
|  |  |  | Rate | 3689.7 (2682.5 to 4924.1) | 3957.1 (2864.5 to 5298.3) | 3434.1 (2486.9 to 4560.2) | 4168.9 (2997.7 to 5604.6) | 4603.2 (3266.3 to 6211.9) | 3759.4 (2728.7 to 5096.3) | 13 (4.4 to 22) | 16.3 (7.3 to 25.8) | 9.5 (0.8 to 18.4) |
|  |  | YLLs (Years of Life Lost) | Number | 228976.9 (166087.7 to 291964.6) | 92734.6 (62222.7 to 117555.3) | 136242.3 (94472.8 to 179123.3) | 48689.1 (40206.2 to 58152.5) | 23706.7 (19590.6 to 27721.2) | 24982.4 (19595.8 to 30991.2) | -78.7 (-84.4 to -69) | -74.4 (-80.9 to -59.9) | -81.7 (-87.2 to -71.3) |
|  |  |  | Rate | 5785.1 (4196.2 to 7376.4) | 4794.5 (3217 to 6077.7) | 6731.8 (4667.9 to 8850.5) | 1331.9 (1099.9 to 1590.8) | 1336.3 (1104.3 to 1562.6) | 1327.8 (1041.5 to 1647.1) | -77 (-83.1 to -66.5) | -72.1 (-79.2 to -56.3) | -80.3 (-86.3 to -69.1) |
| West Azarbayejan | All causes | DALYs (Disability-Adjusted Life Years) | Number | 696728.5 (621949.4 to 781125.4) | 310273.4 (276054.1 to 350337.9) | 386455 (342044 to 433103.9) | 123257.8 (105153.5 to 143716.6) | 58491.7 (48477.6 to 69294.9) | 64766.1 (56119.9 to 74288.3) | -82.3 (-85.1 to -79.3) | -81.1 (-84.4 to -77.8) | -83.2 (-85.7 to -80.4) |
|  |  |  | Rate | 53017.7 (47327.4 to 59439.9) | 47889.9 (42608.2 to 54073.7) | 58004.3 (51338.5 to 65005.9) | 10501.7 (8959.2 to 12244.9) | 10275.7 (8516.5 to 12173.6) | 10714.6 (9284.2 to 12289.9) | -80.2 (-83.3 to -76.8) | -78.5 (-82.3 to -74.7) | -81.5 (-84.2 to -78.4) |
|  |  | YLDs (Years Lived with Disability) | Number | 73725.5 (53850.9 to 97491) | 37908 (27670.6 to 50868.3) | 35817.5 (26126.1 to 48623.4) | 60528.5 (43870 to 81191.3) | 32389.9 (23276 to 43313) | 28138.6 (20217.8 to 37629.7) | -17.9 (-24.2 to -10.9) | -14.6 (-22 to -6.7) | -21.4 (-29.3 to -11.8) |
|  |  |  | Rate | 5610.2 (4097.8 to 7418.6) | 5851 (4270.9 to 7851.4) | 5376 (3921.3 to 7298) | 5157.1 (3737.8 to 6917.6) | 5690.2 (4089.1 to 7609.2) | 4655.1 (3344.7 to 6225.3) | -8.1 (-15.1 to -0.2) | -2.7 (-11.2 to 6.2) | -13.4 (-22 to -2.8) |
|  |  | YLLs (Years of Life Lost) | Number | 623003 (548279.7 to 700846.8) | 272365.4 (239307.4 to 307013.1) | 350637.6 (307008.1 to 396085.8) | 62729.2 (57618.7 to 68173.8) | 26101.8 (23896.9 to 28700.9) | 36627.5 (33446.9 to 40179.6) | -89.9 (-91.2 to -88.2) | -90.4 (-91.9 to -88.6) | -89.6 (-91 to -87.7) |
|  |  |  | Rate | 47407.6 (41721.5 to 53331.1) | 42038.9 (36936.4 to 47386.6) | 52628.3 (46079.8 to 59449.8) | 5344.6 (4909.2 to 5808.5) | 4585.5 (4198.2 to 5042.1) | 6059.5 (5533.3 to 6647.1) | -88.7 (-90.2 to -86.8) | -89.1 (-90.7 to -87) | -88.5 (-90 to -86.4) |
|  | Communicable, maternal, neonatal, and nutritional diseases | DALYs (Disability-Adjusted Life Years) | Number | 324942 (272278.1 to 406178) | 149015.1 (123670.6 to 194412) | 175926.9 (146301.2 to 219842.6) | 29933 (25393.1 to 35617.5) | 14568.8 (12153.7 to 17227.8) | 15364.1 (12891.1 to 18602.3) | -90.8 (-93.1 to -88.1) | -90.2 (-93 to -87.3) | -91.3 (-93.8 to -88.6) |
|  |  |  | Rate | 24726.5 (20719.1 to 30908.2) | 23000.1 (19088.2 to 30007) | 26405.4 (21958.8 to 32996.9) | 2550.3 (2163.5 to 3034.7) | 2559.4 (2135.1 to 3026.6) | 2541.8 (2132.6 to 3077.5) | -89.7 (-92.3 to -86.7) | -88.9 (-92.1 to -85.6) | -90.4 (-93.1 to -87.5) |
|  |  | YLDs (Years Lived with Disability) | Number | 18001.9 (12476.8 to 26419.7) | 8974.1 (5982.6 to 13301.9) | 9027.8 (5862.4 to 13925.7) | 11021.3 (7535.3 to 15846.3) | 5901.8 (3885.1 to 8442.2) | 5119.4 (3362.2 to 7656.7) | -38.8 (-52.5 to -17.5) | -34.2 (-48.9 to -14.1) | -43.3 (-60.3 to -12.1) |
|  |  |  | Rate | 1369.9 (949.4 to 2010.4) | 1385.1 (923.4 to 2053.1) | 1355 (879.9 to 2090.2) | 939 (642 to 1350.1) | 1036.8 (682.5 to 1483.1) | 846.9 (556.2 to 1266.7) | -31.5 (-46.9 to -7.6) | -25.1 (-41.9 to -2.2) | -37.5 (-56.3 to -3.1) |
|  |  | YLLs (Years of Life Lost) | Number | 306940.1 (255105.1 to 387551.5) | 140041 (114331.6 to 184229.9) | 166899.1 (137755.7 to 210581.5) | 18911.7 (15867.9 to 22079.3) | 8667 (7096.6 to 10230.1) | 10244.7 (8268.5 to 12049.6) | -93.8 (-95.6 to -92.1) | -93.8 (-95.9 to -91.9) | -93.9 (-95.8 to -92.1) |
|  |  |  | Rate | 23356.7 (19412.3 to 29490.8) | 21615 (17646.8 to 28435.4) | 25050.4 (20676.2 to 31606.8) | 1611.3 (1352 to 1881.2) | 1522.6 (1246.7 to 1797.2) | 1694.8 (1367.9 to 1993.4) | -93.1 (-95.1 to -91.2) | -93 (-95.3 to -90.8) | -93.2 (-95.4 to -91.3) |
|  | Injuries | DALYs (Disability-Adjusted Life Years) | Number | 125607.9 (107285.7 to 148265.1) | 45233.2 (35298.4 to 55459.7) | 80374.7 (67918.7 to 95817.4) | 22149.9 (19835 to 24998.5) | 7385.9 (6503.5 to 8392) | 14764 (12850.6 to 16955) | -82.4 (-85.7 to -78.1) | -83.7 (-87.1 to -79.1) | -81.6 (-85.4 to -77) |
|  |  |  | Rate | 9558.2 (8163.9 to 11282.3) | 6981.6 (5448.2 to 8560.1) | 12063.7 (10194.1 to 14381.5) | 1887.2 (1690 to 2129.9) | 1297.5 (1142.5 to 1474.3) | 2442.5 (2125.9 to 2805) | -80.3 (-83.9 to -75.5) | -81.4 (-85.4 to -76.2) | -79.8 (-84 to -74.6) |
|  |  | YLDs (Years Lived with Disability) | Number | 5028.9 (3726 to 6560.6) | 2217.5 (1633.1 to 2910.7) | 2811.5 (2075 to 3688.7) | 1695.1 (1233.9 to 2243.4) | 751.9 (550.4 to 997.4) | 943.2 (691.5 to 1267.2) | -66.3 (-69.1 to -63.5) | -66.1 (-69.4 to -62.5) | -66.5 (-69.7 to -63.4) |
|  |  |  | Rate | 382.7 (283.5 to 499.2) | 342.3 (252.1 to 449.3) | 422 (311.4 to 553.7) | 144.4 (105.1 to 191.1) | 132.1 (96.7 to 175.2) | 156 (114.4 to 209.6) | -62.3 (-65.4 to -59.1) | -61.4 (-65.1 to -57.3) | -63 (-66.6 to -59.7) |
|  |  | YLLs (Years of Life Lost) | Number | 120579 (101926.7 to 143590.3) | 43015.7 (33002 to 53033.1) | 77563.3 (65144 to 92543.5) | 20454.8 (18161.2 to 23257.5) | 6634 (5810.3 to 7641.8) | 13820.8 (11985.2 to 15976.3) | -83 (-86.4 to -78.8) | -84.6 (-88.1 to -80) | -82.2 (-86.1 to -77.4) |
|  |  |  | Rate | 9175.5 (7756.1 to 10926.5) | 6639.4 (5093.8 to 8185.5) | 11641.7 (9777.7 to 13890.1) | 1742.8 (1547.4 to 1981.6) | 1165.5 (1020.7 to 1342.5) | 2286.4 (1982.8 to 2643) | -81 (-84.7 to -76.2) | -82.4 (-86.4 to -77.3) | -80.4 (-84.7 to -75.1) |
|  | Non-communicable diseases | DALYs (Disability-Adjusted Life Years) | Number | 246178.5 (183927.4 to 290523.7) | 116025.1 (72931.7 to 139392.7) | 130153.4 (91040.4 to 158453.1) | 70607.8 (56507.9 to 86653) | 36368.2 (29014.1 to 45350.9) | 34239.5 (27426.8 to 41617.6) | -71.3 (-77.9 to -59.7) | -68.7 (-76 to -49.2) | -73.7 (-79.5 to -60.6) |
|  |  |  | Rate | 18733 (13996 to 22107.5) | 17908.2 (11256.8 to 21514.9) | 19535.1 (13664.5 to 23782.7) | 6015.9 (4814.6 to 7383) | 6389.1 (5097.2 to 7967.2) | 5664.4 (4537.3 to 6885) | -67.9 (-75.2 to -54.9) | -64.3 (-72.7 to -42.2) | -71 (-77.4 to -56.6) |
|  |  | YLDs (Years Lived with Disability) | Number | 50694.6 (36987.5 to 67641.3) | 26716.4 (19376.7 to 36019.6) | 23978.2 (17544.9 to 32013.7) | 47812.2 (34113 to 63157.7) | 25736.2 (18321.8 to 34399.3) | 22076 (15862.3 to 29215.5) | -5.7 (-12.6 to 2.3) | -3.7 (-11.1 to 4.6) | -7.9 (-15.4 to 0.4) |
|  |  |  | Rate | 3857.6 (2814.6 to 5147.2) | 4123.6 (2990.7 to 5559.5) | 3599 (2633.4 to 4805) | 4073.7 (2906.5 to 5381.1) | 4521.3 (3218.7 to 6043.2) | 3652.1 (2624.2 to 4833.3) | 5.6 (-2.2 to 14.5) | 9.6 (1.1 to 19) | 1.5 (-6.7 to 10.7) |
|  |  | YLLs (Years of Life Lost) | Number | 195483.9 (133801.8 to 234860.5) | 89308.7 (46131 to 108640.8) | 106175.2 (68011 to 130377.6) | 22795.6 (19374.3 to 26780.3) | 10632.1 (9014.1 to 12419.9) | 12163.5 (10011.5 to 14939.3) | -88.3 (-91 to -81.2) | -88.1 (-91 to -76.4) | -88.5 (-91.6 to -79.2) |
|  |  |  | Rate | 14875.4 (10181.7 to 17871.8) | 13784.6 (7120.2 to 16768.4) | 15936.2 (10208 to 19568.8) | 1942.2 (1650.7 to 2281.7) | 1867.8 (1583.6 to 2181.9) | 2012.3 (1656.2 to 2471.5) | -86.9 (-90 to -79) | -86.4 (-89.7 to -73.1) | -87.4 (-90.7 to -77.1) |
| Yazd | All causes | DALYs (Disability-Adjusted Life Years) | Number | 119535.5 (107115.1 to 133990.1) | 53517.5 (47632.6 to 60257.4) | 66018 (58436.9 to 74298.2) | 39527.5 (33551.5 to 46465.1) | 18734.6 (15539.2 to 22373.7) | 20792.9 (17973.8 to 24191.3) | -66.9 (-72.5 to -61) | -65 (-71.1 to -58.3) | -68.5 (-73.9 to -62.5) |
|  |  |  | Rate | 31788.2 (28485.2 to 35632.1) | 29155.3 (25949.4 to 32827.1) | 34299.1 (30360.4 to 38601) | 9716.2 (8247.3 to 11421.6) | 9397.4 (7794.6 to 11222.8) | 10022.6 (8663.7 to 11660.7) | -69.4 (-74.6 to -64) | -67.8 (-73.4 to -61.6) | -70.8 (-75.8 to -65.3) |
|  |  | YLDs (Years Lived with Disability) | Number | 20297.1 (15057.5 to 26930.3) | 10202 (7449.7 to 13481.4) | 10095.1 (7410.2 to 13559.7) | 20460.1 (14966.1 to 27569.6) | 10896.9 (7970 to 14599.6) | 9563.2 (6964.2 to 12933.2) | 0.8 (-8.3 to 10) | 6.8 (-2.2 to 17.1) | -5.3 (-16.1 to 4.8) |
|  |  |  | Rate | 5397.6 (4004.2 to 7161.6) | 5557.8 (4058.5 to 7344.4) | 5244.8 (3849.9 to 7044.8) | 5029.3 (3678.8 to 6776.9) | 5466 (3997.8 to 7323.3) | 4609.7 (3356.9 to 6234) | -6.8 (-15.2 to 1.7) | -1.7 (-10 to 7.8) | -12.1 (-22.2 to -2.7) |
|  |  | YLLs (Years of Life Lost) | Number | 99238.5 (86883.4 to 112285.1) | 43315.5 (38160 to 48952.7) | 55922.9 (48608.9 to 63476.8) | 19067.3 (17172.2 to 21121.3) | 7837.7 (6900.6 to 8764.3) | 11229.6 (9956.4 to 12548.3) | -80.8 (-84 to -76.6) | -81.9 (-85.2 to -77.9) | -79.9 (-83.3 to -75.5) |
|  |  |  | Rate | 26390.6 (23105 to 29860.1) | 23597.5 (20788.9 to 26668.6) | 29054.3 (25254.3 to 32978.8) | 4686.9 (4221.1 to 5191.8) | 3931.5 (3461.4 to 4396.2) | 5412.9 (4799.2 to 6048.5) | -82.2 (-85.2 to -78.4) | -83.3 (-86.4 to -79.7) | -81.4 (-84.5 to -77.2) |
|  | Communicable, maternal, neonatal, and nutritional diseases | DALYs (Disability-Adjusted Life Years) | Number | 54133.1 (45545.2 to 65155) | 25017.9 (21048.8 to 31266.4) | 29115.2 (24016.7 to 34967.5) | 10725.1 (9047.1 to 12543.1) | 5233.4 (4309.1 to 6317.8) | 5491.7 (4708.9 to 6413.6) | -80.2 (-84.7 to -75.3) | -79.1 (-84.5 to -72.8) | -81.1 (-85.7 to -76.3) |
|  |  |  | Rate | 14395.7 (12111.9 to 17326.7) | 13629.3 (11467 to 17033.4) | 15126.6 (12477.7 to 18167.1) | 2636.3 (2223.9 to 3083.2) | 2625.1 (2161.5 to 3169.1) | 2647.1 (2269.8 to 3091.5) | -81.7 (-85.9 to -77.1) | -80.7 (-85.7 to -75) | -82.5 (-86.7 to -78) |
|  |  | YLDs (Years Lived with Disability) | Number | 5481.7 (3856.6 to 7762.1) | 2699.3 (1838.9 to 3871.4) | 2782.3 (1883.8 to 4205) | 4019.9 (2822.3 to 5703.7) | 2201.8 (1476.3 to 3241.9) | 1818.1 (1237.1 to 2598.9) | -26.7 (-40.4 to -6.2) | -18.4 (-35.5 to 5.9) | -34.7 (-52.5 to -11.6) |
|  |  |  | Rate | 1457.7 (1025.6 to 2064.2) | 1470.5 (1001.8 to 2109.1) | 1445.5 (978.7 to 2184.7) | 988.1 (693.7 to 1402) | 1104.4 (740.5 to 1626.1) | 876.3 (596.3 to 1252.7) | -32.2 (-44.9 to -13.3) | -24.9 (-40.6 to -2.5) | -39.4 (-55.9 to -18) |
|  |  | YLLs (Years of Life Lost) | Number | 48651.5 (40322.5 to 59217.4) | 22318.6 (18377 to 28566.6) | 26332.9 (21543.6 to 31959.2) | 6705.2 (5697 to 7798.2) | 3031.6 (2510.6 to 3620.5) | 3673.6 (3109.3 to 4269.5) | -86.2 (-89.8 to -82.6) | -86.4 (-90.6 to -82.2) | -86 (-89.9 to -82.2) |
|  |  |  | Rate | 12937.9 (10723 to 15747.7) | 12158.8 (10011.4 to 15562.6) | 13681 (11192.8 to 16604.1) | 1648.2 (1400.4 to 1916.9) | 1520.7 (1259.4 to 1816.1) | 1770.7 (1498.7 to 2058) | -87.3 (-90.5 to -83.9) | -87.5 (-91.4 to -83.6) | -87.1 (-90.7 to -83.5) |
|  | Injuries | DALYs (Disability-Adjusted Life Years) | Number | 20751.3 (18143.3 to 23625.2) | 7020.2 (5823.7 to 8507) | 13731.2 (11884 to 15795.3) | 5822 (5075 to 6671.8) | 1783.5 (1530.3 to 2133.8) | 4038.5 (3379.3 to 4744.3) | -71.9 (-77 to -65.9) | -74.6 (-80.1 to -67.3) | -70.6 (-76.8 to -64) |
|  |  |  | Rate | 5518.4 (4824.9 to 6282.7) | 3824.5 (3172.7 to 4634.5) | 7133.9 (6174.2 to 8206.3) | 1431.1 (1247.5 to 1640) | 894.6 (767.6 to 1070.3) | 1946.7 (1628.9 to 2286.8) | -74.1 (-78.7 to -68.5) | -76.6 (-81.7 to -69.9) | -72.7 (-78.5 to -66.6) |
|  |  | YLDs (Years Lived with Disability) | Number | 1445 (1077 to 1909) | 595.4 (446.5 to 796.4) | 849.6 (632.9 to 1124.2) | 608.5 (442.5 to 816) | 276.5 (196.9 to 370.3) | 332 (240.6 to 450.6) | -57.9 (-62.5 to -53.5) | -53.6 (-59 to -48.7) | -60.9 (-66 to -56.2) |
|  |  |  | Rate | 384.3 (286.4 to 507.7) | 324.3 (243.3 to 433.9) | 441.4 (328.8 to 584.1) | 149.6 (108.8 to 200.6) | 138.7 (98.8 to 185.8) | 160.1 (116 to 217.2) | -61.1 (-65.4 to -57) | -57.2 (-62.2 to -52.8) | -63.7 (-68.4 to -59.4) |
|  |  | YLLs (Years of Life Lost) | Number | 19306.4 (16730.6 to 22239.3) | 6424.8 (5250.2 to 7850.3) | 12881.6 (11042.1 to 14940.2) | 5213.5 (4495.4 to 6074.2) | 1507 (1279.5 to 1827.6) | 3706.5 (3065.3 to 4383.8) | -73 (-78.2 to -66.6) | -76.5 (-82.2 to -68.9) | -71.2 (-77.6 to -64.1) |
|  |  |  | Rate | 5134.2 (4449.2 to 5914.1) | 3500.1 (2860.2 to 4276.7) | 6692.5 (5736.8 to 7762) | 1281.5 (1105 to 1493.1) | 755.9 (641.8 to 916.7) | 1786.6 (1477.5 to 2113.1) | -75 (-79.9 to -69.2) | -78.4 (-83.6 to -71.4) | -73.3 (-79.2 to -66.7) |
|  | Non-communicable diseases | DALYs (Disability-Adjusted Life Years) | Number | 44651.1 (35521 to 52676.3) | 21479.4 (15372.7 to 25731.8) | 23171.7 (17774.4 to 27825.4) | 22877.3 (18248.4 to 28602.2) | 11703.1 (9186 to 14756) | 11174.2 (9037 to 13850.8) | -48.8 (-59 to -32.1) | -45.5 (-56.7 to -22) | -51.8 (-61.9 to -33.9) |
|  |  |  | Rate | 11874.1 (9446.1 to 14008.3) | 11701.6 (8374.8 to 14018.2) | 12038.6 (9234.5 to 14456.4) | 5623.4 (4485.6 to 7030.7) | 5870.3 (4607.8 to 7401.7) | 5386.2 (4356 to 6676.4) | -52.6 (-62.1 to -37.2) | -49.8 (-60.2 to -28.2) | -55.3 (-64.7 to -38.7) |
|  |  | YLDs (Years Lived with Disability) | Number | 13370.4 (9727.8 to 17791.4) | 6907.3 (5003.9 to 9196.5) | 6463.2 (4699.6 to 8570) | 15831.8 (11365.6 to 21492.1) | 8418.6 (6036.1 to 11414.7) | 7413.1 (5299.7 to 9976.9) | 18.4 (8.7 to 28.9) | 21.9 (11.9 to 32.7) | 14.7 (4.4 to 26) |
|  |  |  | Rate | 3555.6 (2586.9 to 4731.3) | 3763 (2726 to 5010.1) | 3357.9 (2441.7 to 4452.5) | 3891.6 (2793.8 to 5282.9) | 4222.9 (3027.8 to 5725.7) | 3573.3 (2554.5 to 4809) | 9.4 (0.4 to 19.2) | 12.2 (3.1 to 22.2) | 6.4 (-3.1 to 16.9) |
|  |  | YLLs (Years of Life Lost) | Number | 31280.6 (22163.7 to 37751.6) | 14572.1 (8600.4 to 17907.6) | 16708.5 (11339.2 to 20620.7) | 7045.5 (5895.9 to 8424.8) | 3284.4 (2690 to 3916.2) | 3761.1 (3068.6 to 4663.2) | -77.5 (-82.4 to -64.5) | -77.5 (-82.9 to -59.5) | -77.5 (-83 to -62.6) |
|  |  |  | Rate | 8318.5 (5894 to 10039.3) | 7938.6 (4685.4 to 9755.7) | 8680.8 (5891.2 to 10713.3) | 1731.9 (1449.3 to 2070.9) | 1647.5 (1349.3 to 1964.4) | 1812.9 (1479.1 to 2247.8) | -79.2 (-83.8 to -67.2) | -79.2 (-84.2 to -62.7) | -79.1 (-84.3 to -65.3) |
| Zanjan | All causes | DALYs (Disability-Adjusted Life Years) | Number | 860869 (800360.3 to 921807.2) | 467329.4 (433971.3 to 501256.8) | 393539.6 (366445.1 to 422706.7) | 28973.2 (24393.3 to 34255.2) | 13571.9 (11086.1 to 16440.6) | 15401.3 (13159 to 17890.5) | -96.6 (-97.2 to -96) | -97.1 (-97.7 to -96.5) | -96.1 (-96.7 to -95.4) |
|  |  |  | Rate | 163599.8 (152100.7 to 175180.5) | 181065 (168140.6 to 194210.1) | 146786.2 (136680.3 to 157665.3) | 9217.5 (7760.5 to 10897.9) | 8836.3 (7217.9 to 10704.1) | 9581.8 (8186.8 to 11130.4) | -94.4 (-95.3 to -93.3) | -95.1 (-96.1 to -94.1) | -93.5 (-94.6 to -92.4) |
|  |  | YLDs (Years Lived with Disability) | Number | 30824.5 (22489.3 to 41406.5) | 15950 (11597.3 to 21552.1) | 14874.5 (10658.9 to 20396.4) | 15765.9 (11467.7 to 21241.8) | 8363.2 (6021.3 to 11219.2) | 7402.7 (5380.6 to 9935.5) | -48.9 (-53.2 to -44) | -47.6 (-52.6 to -42.6) | -50.2 (-55.9 to -43.9) |
|  |  |  | Rate | 5857.9 (4273.9 to 7868.9) | 6179.8 (4493.3 to 8350.3) | 5548 (3975.6 to 7607.6) | 5015.8 (3648.3 to 6757.8) | 5445.1 (3920.3 to 7304.5) | 4605.5 (3347.5 to 6181.2) | -14.4 (-21.6 to -6.2) | -11.9 (-20.3 to -3.6) | -17 (-26.4 to -6.5) |
|  |  | YLLs (Years of Life Lost) | Number | 830044.4 (772465.7 to 890318.5) | 451379.3 (419101.3 to 485868.8) | 378665.1 (352519.1 to 406498.2) | 13207.3 (11716.3 to 14767.9) | 5208.7 (4576.3 to 5868.2) | 7998.6 (7067.8 to 8952.2) | -98.4 (-98.6 to -98.2) | -98.8 (-99 to -98.7) | -97.9 (-98.2 to -97.6) |
|  |  |  | Rate | 157741.9 (146799.6 to 169196.4) | 174885.3 (162379.2 to 188248.1) | 141238.2 (131486 to 151619.7) | 4201.8 (3727.4 to 4698.2) | 3391.2 (2979.5 to 3820.6) | 4976.2 (4397.1 to 5569.5) | -97.3 (-97.7 to -97) | -98.1 (-98.3 to -97.8) | -96.5 (-97 to -96) |
|  | Communicable, maternal, neonatal, and nutritional diseases | DALYs (Disability-Adjusted Life Years) | Number | 128970.6 (107172.1 to 158336.6) | 58644.8 (48623.5 to 75236.7) | 70325.7 (57471.5 to 86894.3) | 7931.5 (6674.4 to 9510.7) | 3789 (3141.1 to 4583.7) | 4142.5 (3472.5 to 5047.4) | -93.9 (-95.3 to -91.9) | -93.5 (-95.4 to -91.6) | -94.1 (-95.7 to -92.2) |
|  |  |  | Rate | 24509.6 (20367 to 30090.3) | 22721.7 (18839 to 29150.2) | 26230.8 (21436.3 to 32410.7) | 2523.3 (2123.4 to 3025.7) | 2466.9 (2045.1 to 2984.3) | 2577.2 (2160.4 to 3140.2) | -89.7 (-92.1 to -86.5) | -89.1 (-92.2 to -85.8) | -90.2 (-92.9 to -87) |
|  |  | YLDs (Years Lived with Disability) | Number | 6633.1 (4359.7 to 9646.3) | 3244.1 (2144 to 4671.8) | 3389 (2079.2 to 5298.9) | 2887.9 (1975.8 to 4250.8) | 1572.2 (1057.1 to 2328.7) | 1315.7 (857.7 to 2001.4) | -56.5 (-66.4 to -42.9) | -51.5 (-63.6 to -35) | -61.2 (-73.5 to -39.4) |
|  |  |  | Rate | 1260.5 (828.5 to 1833.2) | 1256.9 (830.7 to 1810.1) | 1264.1 (775.5 to 1976.4) | 918.7 (628.6 to 1352.3) | 1023.6 (688.3 to 1516.2) | 818.5 (533.6 to 1245.1) | -27.1 (-43.7 to -4.5) | -18.6 (-38.8 to 9.3) | -35.2 (-55.7 to 1) |
|  |  | YLLs (Years of Life Lost) | Number | 122337.5 (100629.2 to 151141.7) | 55400.8 (45308.5 to 72372.5) | 66936.7 (54005.1 to 83238) | 5043.6 (4260.2 to 5970.1) | 2216.8 (1844.6 to 2639.1) | 2826.8 (2359.5 to 3355.9) | -95.9 (-97 to -94.6) | -96 (-97.3 to -94.7) | -95.8 (-97 to -94.4) |
|  |  |  | Rate | 23249.1 (19123.6 to 28723) | 21464.8 (17554.6 to 28040.5) | 24966.7 (20143.4 to 31046.9) | 1604.6 (1355.3 to 1899.3) | 1443.3 (1201 to 1718.2) | 1758.7 (1467.9 to 2087.9) | -93.1 (-95 to -90.9) | -93.3 (-95.5 to -91.2) | -93 (-95 to -90.7) |
|  | Injuries | DALYs (Disability-Adjusted Life Years) | Number | 649346.1 (596025.5 to 709393.3) | 368314.6 (336267.4 to 403443.5) | 281031.5 (257460 to 305745) | 4324 (3864.8 to 4798) | 1348.6 (1191.7 to 1497.9) | 2975.5 (2580.1 to 3353.5) | -99.3 (-99.4 to -99.2) | -99.6 (-99.7 to -99.6) | -98.9 (-99.1 to -98.8) |
|  |  |  | Rate | 123401.9 (113268.9 to 134813.3) | 142702.1 (130285.6 to 156312.7) | 104821.9 (96029.9 to 114039.7) | 1375.6 (1229.6 to 1526.4) | 878 (775.9 to 975.3) | 1851.2 (1605.2 to 2086.4) | -98.9 (-99 to -98.7) | -99.4 (-99.5 to -99.3) | -98.2 (-98.5 to -97.9) |
|  |  | YLDs (Years Lived with Disability) | Number | 4794.9 (3535.4 to 6321.9) | 2680.5 (1944.2 to 3609.6) | 2114.4 (1536.5 to 2815.1) | 438.4 (319 to 590.9) | 199.3 (143.5 to 266.5) | 239.2 (173.6 to 324.4) | -90.9 (-92.4 to -89.1) | -92.6 (-94.1 to -90.8) | -88.7 (-90.4 to -86.9) |
|  |  |  | Rate | 911.2 (671.9 to 1201.4) | 1038.6 (753.3 to 1398.5) | 788.7 (573.1 to 1050) | 139.5 (101.5 to 188) | 129.7 (93.4 to 173.5) | 148.8 (108 to 201.9) | -84.7 (-87.3 to -81.7) | -87.5 (-90 to -84.6) | -81.1 (-84 to -78.1) |
|  |  | YLLs (Years of Life Lost) | Number | 644551.2 (591211.3 to 704983.2) | 365634.1 (333361.9 to 400434.6) | 278917.1 (255376.5 to 303643.4) | 3885.6 (3436.5 to 4335.9) | 1149.3 (1007.9 to 1278.6) | 2736.3 (2366.8 to 3130.2) | -99.4 (-99.5 to -99.3) | -99.7 (-99.7 to -99.6) | -99 (-99.2 to -98.8) |
|  |  |  | Rate | 122490.7 (112354 to 133975.2) | 141663.6 (129159.8 to 155146.9) | 104033.2 (95252.8 to 113255.9) | 1236.2 (1093.3 to 1379.4) | 748.3 (656.2 to 832.5) | 1702.4 (1472.5 to 1947.4) | -99 (-99.1 to -98.8) | -99.5 (-99.5 to -99.4) | -98.4 (-98.6 to -98.1) |
|  | Non-communicable diseases | DALYs (Disability-Adjusted Life Years) | Number | 82552.3 (57931.2 to 102971.4) | 40369.9 (25825.5 to 50331) | 42182.4 (27552.4 to 53737.4) | 16647.4 (13027.9 to 20721.2) | 8420.2 (6432.7 to 10758.8) | 8227.2 (6552.1 to 10139.2) | -79.8 (-84.8 to -70.1) | -79.1 (-84.1 to -65.6) | -80.5 (-85.3 to -68.3) |
|  |  |  | Rate | 15688.3 (11009.3 to 19568.7) | 15641.2 (10006 to 19500.6) | 15733.6 (10276.8 to 20043.5) | 5296.2 (4144.7 to 6592.2) | 5482.2 (4188.2 to 7004.7) | 5118.5 (4076.3 to 6308) | -66.2 (-74.5 to -50) | -65 (-73.3 to -42.2) | -67.5 (-75.5 to -47.1) |
|  |  | YLDs (Years Lived with Disability) | Number | 19396.5 (14000.6 to 25979.5) | 10025.4 (7264.1 to 13478.7) | 9371.1 (6793.8 to 12591.4) | 12439.6 (8929.5 to 16709.4) | 6591.8 (4738.5 to 8860.5) | 5847.9 (4227.9 to 7845.9) | -35.9 (-41 to -30.8) | -34.2 (-39.3 to -29) | -37.6 (-42.9 to -31.8) |
|  |  |  | Rate | 3686.1 (2660.7 to 4937.2) | 3884.3 (2814.5 to 5222.3) | 3495.3 (2534 to 4696.5) | 3957.5 (2840.8 to 5315.9) | 4291.7 (3085.1 to 5768.9) | 3638.2 (2630.3 to 4881.3) | 7.4 (-1.2 to 15.9) | 10.5 (1.9 to 19.3) | 4.1 (-4.7 to 13.8) |
|  |  | YLLs (Years of Life Lost) | Number | 63155.8 (38463.9 to 81912.5) | 30344.5 (16045.7 to 39978.6) | 32811.3 (18250.9 to 44189.9) | 4207.8 (3614.8 to 4944.3) | 1828.4 (1536.6 to 2224.3) | 2379.3 (2002.6 to 2873.3) | -93.3 (-94.9 to -88) | -94 (-95.6 to -86.5) | -92.7 (-94.7 to -85.4) |
|  |  |  | Rate | 12002.1 (7309.7 to 15566.7) | 11756.9 (6216.8 to 15489.6) | 12238.3 (6807.4 to 16482.4) | 1338.7 (1150 to 1573) | 1190.4 (1000.4 to 1448.2) | 1480.3 (1245.9 to 1787.6) | -88.8 (-91.5 to -79.9) | -89.9 (-92.5 to -77.4) | -87.9 (-91.1 to -75.6) |
