# Supplementary material for: Disability-adjusted life years, years lived with disability, and years of life lost of diseases among children and adolescents in national and subnational levels of Iran, 1990–2021: A systematic analysis for the Global Burden of Disease 2021
Source: PLoS One. 2025 Jun 23;20(6):e0325085. doi: 10.1371/journal.pone.0325085 (PMC12184942; doi:10.1371/journal.pone.0325085)
Supplement: S2 Table — (DOCX) [file pone.0325085.s023.docx]

**S2 Table.** Rate of disability-adjusted life years (DALYs), years lived with disability (YLDs), and years of life lost (YLLs) of child and adolescents causes of death in Iran in 1990 and 2021, by sex and age

| Cause | Measure | Year | Age Group | Sex | | |
| --- | --- | --- | --- | --- | --- | --- |
| All causes | DALYs (Disability-Adjusted Life Years) | 1990 |  | Both | Females | Males |
|  |  |  | <5 years | 123740.4 (111940 to 135701.1) | 113604.7 (102736.9 to 124645.2) | 133531.4 (120157.1 to 147713.3) |
|  |  |  | 5-9 years | 18408.1 (16978.9 to 20129.3) | 17680 (16309 to 19515.9) | 19108.3 (17538.7 to 21243) |
|  |  |  | 10-14 years | 17027.2 (15233.2 to 19322.6) | 18398.7 (16438.7 to 20813.9) | 15712 (14046.2 to 17966.7) |
|  |  |  | 15-19 years | 21711.6 (19447.7 to 24499) | 20947.9 (18303.3 to 24073.9) | 22444.9 (20466.7 to 24942.1) |
| All causes | DALYs (Disability-Adjusted Life Years) | 2021 |  | Both | Females | Males |
|  |  |  | <5 years | 11019.5 (9407.7 to 12732.1) | 10382.7 (8804 to 12034.8) | 11620.7 (9936.7 to 13411.4) |
|  |  |  | 5-9 years | 6226.6 (5250.1 to 7490.6) | 5784.2 (4709.8 to 7169.1) | 6645.6 (5678.9 to 7871) |
|  |  |  | 10-14 years | 8635.9 (6885.8 to 10966.3) | 8696.9 (6753.8 to 11149.4) | 8577.8 (6964.7 to 10552) |
|  |  |  | 15-19 years | 14072.2 (11620.8 to 16865.4) | 13469.3 (10759.3 to 16837) | 14644.8 (12334.5 to 17152.2) |
| All causes | YLDs (Years Lived with Disability) | 1990 |  | Both | Females | Males |
|  |  |  | <5 years | 4343.2 (3078.3 to 5901.9) | 4061 (2867.2 to 5568.2) | 4615.7 (3274.5 to 6279.9) |
|  |  |  | 5-9 years | 4187.9 (3078 to 5602.6) | 4120.4 (3002.4 to 5609.1) | 4252.8 (3048.4 to 5773.1) |
|  |  |  | 10-14 years | 6179.5 (4380.2 to 8556.9) | 6587.4 (4654.2 to 9006.5) | 5788.3 (4132.2 to 7965.5) |
|  |  |  | 15-19 years | 8258.5 (6020.5 to 10967.1) | 9373 (6802 to 12450.7) | 7188.3 (5267.6 to 9580) |
| All causes | YLDs (Years Lived with Disability) | 2021 |  | Both | Females | Males |
|  |  |  | <5 years | 2841.8 (2055.7 to 3914.3) | 2810.3 (1998.4 to 3903.9) | 2871.6 (2058.7 to 3968.6) |
|  |  |  | 5-9 years | 3649.2 (2625.3 to 4904.3) | 3741 (2707.7 to 5149.8) | 3562.3 (2576.3 to 4749.5) |
|  |  |  | 10-14 years | 6094.4 (4340.4 to 8356.1) | 6658.8 (4721.8 to 9119.6) | 5557.3 (3991.3 to 7552.7) |
|  |  |  | 15-19 years | 8463.3 (6135.9 to 11245.3) | 9771.8 (7018.5 to 13135.1) | 7220.4 (5230.7 to 9569.9) |
| All causes | YLLs (Years of Life Lost) | 1990 |  | Both | Females | Males |
|  |  |  | <5 years | 119397.3 (107666.1 to 131360.2) | 109543.7 (98558.2 to 120287.2) | 128915.7 (115118.7 to 142554.3) |
|  |  |  | 5-9 years | 14220.2 (13375.6 to 15130.5) | 13559.6 (12774.1 to 14389.5) | 14855.5 (13816.1 to 15925.4) |
|  |  |  | 10-14 years | 10847.7 (10350.5 to 11423.9) | 11811.3 (11127.7 to 12543.5) | 9923.7 (9460.8 to 10461) |
|  |  |  | 15-19 years | 13453.2 (12935.9 to 14021.5) | 11574.9 (11028.4 to 12182.1) | 15256.6 (14545.3 to 16038.2) |
| All causes | YLLs (Years of Life Lost) | 2021 |  | Both | Females | Males |
|  |  |  | <5 years | 8177.6 (6834.7 to 9617.4) | 7572.3 (6251.9 to 8881.6) | 8749.1 (7283.5 to 10409.7) |
|  |  |  | 5-9 years | 2577.4 (2367.4 to 2784) | 2043.1 (1890.2 to 2225.3) | 3083.3 (2810.6 to 3333) |
|  |  |  | 10-14 years | 2541.5 (2323.9 to 2702.1) | 2038.1 (1824.3 to 2220.2) | 3020.6 (2761.1 to 3197.6) |
|  |  |  | 15-19 years | 5608.8 (5063.7 to 5969.1) | 3697.5 (3458 to 3874.8) | 7424.4 (6515.5 to 8052.5) |
| Communicable, maternal, neonatal, and nutritional diseases | DALYs (Disability-Adjusted Life Years) | 1990 |  | Both | Females | Males |
|  |  |  | <5 years | 64681.1 (55318.4 to 78980.2) | 58754.2 (49983.1 to 74654.3) | 70406.3 (59804.2 to 86883.7) |
|  |  |  | 5-9 years | 2493.7 (1937.3 to 3298.8) | 2459.8 (1783.1 to 3441.4) | 2526.3 (1914.1 to 3521.1) |
|  |  |  | 10-14 years | 1532.9 (1172.3 to 2021.5) | 1625.8 (1199.4 to 2195) | 1443.7 (1048.5 to 2004.2) |
|  |  |  | 15-19 years | 1449.8 (1045.4 to 2335.6) | 1731 (1219.8 to 2788.6) | 1179.9 (852.5 to 2007.3) |
| Communicable, maternal, neonatal, and nutritional diseases | DALYs (Disability-Adjusted Life Years) | 2021 |  | Both | Females | Males |
|  |  |  | <5 years | 5869.1 (4941.8 to 6866) | 5583.4 (4663.4 to 6614.6) | 6138.8 (5148.2 to 7303.3) |
|  |  |  | 5-9 years | 1301.5 (976.3 to 1725.5) | 1336.3 (939 to 1949.5) | 1268.6 (975.2 to 1658.7) |
|  |  |  | 10-14 years | 1170.5 (880.1 to 1508.4) | 1225.3 (890.3 to 1651.7) | 1118.2 (868.5 to 1493.5) |
|  |  |  | 15-19 years | 1422.4 (1165.6 to 1747.1) | 1603.2 (1253 to 2072.7) | 1250.7 (1050.6 to 1540.3) |
| Communicable, maternal, neonatal, and nutritional diseases | YLDs (Years Lived with Disability) | 1990 |  | Both | Females | Males |
|  |  |  | <5 years | 2226.5 (1517.2 to 3175.7) | 2054.5 (1384.2 to 2913.1) | 2392.6 (1550.5 to 3478.8) |
|  |  |  | 5-9 years | 1214.5 (783.1 to 1787.7) | 1214.7 (739.5 to 1940.8) | 1214.4 (725.3 to 1990.7) |
|  |  |  | 10-14 years | 925.7 (596.6 to 1377.6) | 1007.3 (626.4 to 1570.9) | 847.5 (499.1 to 1371.2) |
|  |  |  | 15-19 years | 820.6 (525.8 to 1207.5) | 1040.5 (626.7 to 1666.6) | 609.5 (406.7 to 895.6) |
| Communicable, maternal, neonatal, and nutritional diseases | YLDs (Years Lived with Disability) | 2021 |  | Both | Females | Males |
|  |  |  | <5 years | 1220.4 (825.8 to 1762.5) | 1275.4 (851 to 1869.4) | 1168.5 (753.4 to 1748.4) |
|  |  |  | 5-9 years | 947.7 (643.1 to 1372.4) | 1050.7 (657.2 to 1651.3) | 850 (569.4 to 1245.3) |
|  |  |  | 10-14 years | 830.3 (560.9 to 1188.8) | 934.1 (607.8 to 1384.8) | 731.6 (490.2 to 1129.9) |
|  |  |  | 15-19 years | 815.4 (560.4 to 1149.8) | 1000 (648.4 to 1462.1) | 640.1 (450.5 to 953.5) |
| Communicable, maternal, neonatal, and nutritional diseases | YLLs (Years of Life Lost) | 1990 |  | Both | Females | Males |
|  |  |  | <5 years | 62454.6 (53120.2 to 76464.1) | 56699.7 (47950.2 to 72513.4) | 68013.7 (56974.7 to 84377.5) |
|  |  |  | 5-9 years | 1279.2 (939.7 to 1858) | 1245.1 (872.2 to 1825.8) | 1312 (974.6 to 1871.7) |
|  |  |  | 10-14 years | 607.1 (483.1 to 843) | 618.5 (483.9 to 863.7) | 596.2 (459.6 to 831.7) |
|  |  |  | 15-19 years | 629.2 (433.4 to 1516.5) | 690.5 (482.3 to 1543) | 570.4 (366.9 to 1510.8) |
| Communicable, maternal, neonatal, and nutritional diseases | YLLs (Years of Life Lost) | 2021 |  | Both | Females | Males |
|  |  |  | <5 years | 4648.7 (3825.9 to 5632.4) | 4308 (3506.4 to 5235.2) | 4970.4 (4044.7 to 5982.2) |
|  |  |  | 5-9 years | 353.8 (273 to 436.5) | 285.5 (218.8 to 377.8) | 418.5 (307.3 to 507.8) |
|  |  |  | 10-14 years | 340.1 (256.5 to 392.9) | 291.2 (204.3 to 374.8) | 386.6 (301.7 to 432.2) |
|  |  |  | 15-19 years | 607 (577.2 to 634.3) | 603.3 (573.6 to 636.2) | 610.5 (575.8 to 646) |
| Injuries | DALYs (Disability-Adjusted Life Years) | 1990 |  | Both | Females | Males |
|  |  |  | <5 years | 24490.8 (22225.5 to 27103.4) | 23123.3 (20798.4 to 25956.8) | 25811.8 (23049.9 to 29332.2) |
|  |  |  | 5-9 years | 10457.3 (9718.9 to 11273.4) | 10079.6 (9349.4 to 10866.5) | 10820.5 (9952.5 to 11824.7) |
|  |  |  | 10-14 years | 8834.9 (8304.8 to 9449) | 9757.2 (9026.5 to 10535.2) | 7950.5 (7493.3 to 8446.3) |
|  |  |  | 15-19 years | 11299.7 (10623.8 to 11949.5) | 9417.3 (8751.7 to 10084.4) | 13107.2 (12287.7 to 13902.1) |
| Injuries | DALYs (Disability-Adjusted Life Years) | 2021 |  | Both | Females | Males |
|  |  |  | <5 years | 689.6 (583.6 to 809.7) | 607.8 (492.3 to 734.5) | 766.8 (631.1 to 928.8) |
|  |  |  | 5-9 years | 1230.9 (1115.7 to 1358.3) | 902.7 (818.9 to 997.3) | 1541.6 (1379.5 to 1727.1) |
|  |  |  | 10-14 years | 1284.1 (1181.3 to 1403.3) | 880.1 (789.8 to 991.7) | 1668.5 (1537.3 to 1829.9) |
|  |  |  | 15-19 years | 3513.1 (3221.5 to 3750.8) | 1771.4 (1637.7 to 1958.2) | 5167.6 (4650.7 to 5546.6) |
| Injuries | YLDs (Years Lived with Disability) | 1990 |  | Both | Females | Males |
|  |  |  | <5 years | 223.9 (158.8 to 294.2) | 232 (163.3 to 306.8) | 216.1 (153.4 to 285.4) |
|  |  |  | 5-9 years | 311.2 (227.1 to 418.2) | 307.6 (225 to 412.1) | 314.7 (230.5 to 423.7) |
|  |  |  | 10-14 years | 456.2 (338.4 to 601.9) | 437 (329.3 to 573.6) | 474.7 (349.7 to 633.6) |
|  |  |  | 15-19 years | 695.5 (526.2 to 913.1) | 568.5 (433.4 to 735.1) | 817.4 (618.4 to 1095.5) |
| Injuries | YLDs (Years Lived with Disability) | 2021 |  | Both | Females | Males |
|  |  |  | <5 years | 48.4 (33.7 to 65.9) | 46.1 (32 to 62.7) | 50.6 (35.4 to 68.9) |
|  |  |  | 5-9 years | 98.6 (71.4 to 135.6) | 96 (69.3 to 131.2) | 101 (72.5 to 139.6) |
|  |  |  | 10-14 years | 171.1 (122.6 to 231.9) | 162 (115.7 to 220) | 179.6 (128.4 to 245) |
|  |  |  | 15-19 years | 304.4 (223.3 to 401.7) | 260.3 (192.8 to 343.8) | 346.3 (253.5 to 462.7) |
| Injuries | YLLs (Years of Life Lost) | 1990 |  | Both | Females | Males |
|  |  |  | <5 years | 24266.9 (21982.2 to 26850.2) | 22891.3 (20615.9 to 25731) | 25595.7 (22815.3 to 29073.9) |
|  |  |  | 5-9 years | 10146.1 (9402.4 to 10916.5) | 9772 (9065 to 10563.2) | 10505.8 (9659.7 to 11479.8) |
|  |  |  | 10-14 years | 8378.7 (7865.8 to 8946.8) | 9320.2 (8613.6 to 10072.9) | 7475.8 (7022.6 to 7958.3) |
|  |  |  | 15-19 years | 10604.3 (9981 to 11204.5) | 8848.8 (8210.2 to 9485.2) | 12289.9 (11465.2 to 13034.9) |
| Injuries | YLLs (Years of Life Lost) | 2021 |  | Both | Females | Males |
|  |  |  | <5 years | 641.1 (538.7 to 764) | 561.7 (448 to 686.6) | 716.2 (581.2 to 878.5) |
|  |  |  | 5-9 years | 1132.3 (1018.8 to 1254.1) | 806.7 (724.7 to 900) | 1440.5 (1278.2 to 1627.3) |
|  |  |  | 10-14 years | 1113 (1023.3 to 1226.4) | 718 (644.5 to 816.6) | 1488.9 (1364.1 to 1649.5) |
|  |  |  | 15-19 years | 3208.7 (2924 to 3417.9) | 1511.1 (1402.4 to 1677.5) | 4821.3 (4334.2 to 5187.6) |
| Non-communicable diseases | DALYs (Disability-Adjusted Life Years) | 1990 |  | Both | Females | Males |
|  |  |  | <5 years | 34568.5 (23208.7 to 41390.6) | 31727.1 (17941.8 to 38466.7) | 37313.3 (23790.8 to 45566.8) |
|  |  |  | 5-9 years | 5457.1 (4606.3 to 6515.7) | 5140.5 (4232.3 to 6132.3) | 5761.5 (4850.9 to 6873.5) |
|  |  |  | 10-14 years | 6659.4 (5261.6 to 8444.1) | 7015.7 (5469.8 to 8951) | 6317.8 (5027.4 to 7961) |
|  |  |  | 15-19 years | 8962 (7022.9 to 11378.4) | 9799.6 (7600.4 to 12585.3) | 8157.8 (6537 to 10198.7) |
| Non-communicable diseases | DALYs (Disability-Adjusted Life Years) | 2021 |  | Both | Females | Males |
|  |  |  | <5 years | 4460.8 (3708.5 to 5361.5) | 4191.4 (3427.1 to 5160.8) | 4715 (3892.3 to 5818.9) |
|  |  |  | 5-9 years | 3690.4 (2973.6 to 4613.5) | 3545.2 (2829.4 to 4458.9) | 3828 (3087.8 to 4742.4) |
|  |  |  | 10-14 years | 6157.3 (4642.4 to 8081.4) | 6591.5 (4912.3 to 8657.6) | 5744.2 (4394.3 to 7540.6) |
|  |  |  | 15-19 years | 8977.4 (6872.4 to 11528.5) | 9976.9 (7497 to 12961.7) | 8028 (6296.5 to 10103.3) |
| Non-communicable diseases | YLDs (Years Lived with Disability) | 1990 |  | Both | Females | Males |
|  |  |  | <5 years | 1892.8 (1371.5 to 2531.7) | 1774.5 (1272.9 to 2395.7) | 2007.1 (1461.7 to 2655) |
|  |  |  | 5-9 years | 2662.2 (1918.1 to 3538.1) | 2598.1 (1884.5 to 3500) | 2723.8 (1955.7 to 3619.3) |
|  |  |  | 10-14 years | 4797.5 (3340.9 to 6595.1) | 5143.1 (3583.1 to 7097) | 4466.2 (3124.9 to 6151.2) |
|  |  |  | 15-19 years | 6742.4 (4805.5 to 9160.4) | 7764 (5533 to 10557.2) | 5761.5 (4108.9 to 7763.8) |
| Non-communicable diseases | YLDs (Years Lived with Disability) | 2021 |  | Both | Females | Males |
|  |  |  | <5 years | 1573 (1146.8 to 2091.9) | 1488.8 (1079.3 to 1986.6) | 1652.5 (1215.4 to 2191.7) |
|  |  |  | 5-9 years | 2603 (1864.4 to 3491.5) | 2594.3 (1838.2 to 3520) | 2611.2 (1895.6 to 3486.7) |
|  |  |  | 10-14 years | 5093 (3544.9 to 7014.1) | 5562.7 (3875.3 to 7602.4) | 4646 (3263.4 to 6413.3) |
|  |  |  | 15-19 years | 7343.5 (5235.1 to 9929.2) | 8511.6 (6027.2 to 11528) | 6234 (4485 to 8287.6) |
| Non-communicable diseases | YLLs (Years of Life Lost) | 1990 |  | Both | Females | Males |
|  |  |  | <5 years | 32675.7 (21232.8 to 39376.9) | 29952.6 (16050.5 to 36626.6) | 35306.2 (21668.7 to 43417.9) |
|  |  |  | 5-9 years | 2794.9 (2386.4 to 3168) | 2542.4 (2062.8 to 2885.4) | 3037.7 (2496.4 to 3534.8) |
|  |  |  | 10-14 years | 1861.9 (1666.2 to 2040) | 1872.6 (1631.5 to 2054.8) | 1851.6 (1615.9 to 2079.8) |
|  |  |  | 15-19 years | 2219.7 (1895.4 to 2451.3) | 2035.7 (1667.6 to 2258.1) | 2396.3 (2084.9 to 2728.6) |
| Non-communicable diseases | YLLs (Years of Life Lost) | 2021 |  | Both | Females | Males |
|  |  |  | <5 years | 2887.8 (2284.4 to 3629.8) | 2702.7 (2098.1 to 3487.8) | 3062.5 (2364.4 to 4084.3) |
|  |  |  | 5-9 years | 1087.5 (940.4 to 1226.5) | 950.9 (811.4 to 1059.9) | 1216.8 (1026.8 to 1401.9) |
|  |  |  | 10-14 years | 1064.3 (960.1 to 1160.3) | 1028.8 (902.8 to 1129.1) | 1098.2 (951.2 to 1223.5) |
|  |  |  | 15-19 years | 1633.9 (1483.6 to 1743.4) | 1465.3 (1299.8 to 1583.7) | 1794 (1582 to 1972.1) |
