# Supplementary material for: Disability-adjusted life years, years lived with disability, and years of life lost of diseases among children and adolescents in national and subnational levels of Iran, 1990–2021: A systematic analysis for the Global Burden of Disease 2021
Source: PLoS One. 2025 Jun 23;20(6):e0325085. doi: 10.1371/journal.pone.0325085 (PMC12184942; doi:10.1371/journal.pone.0325085)
Supplement: S3 Table — (DOCX) [file pone.0325085.s024.docx]

**S3 Table.** Subnational rate of disability-adjusted life years (DALYs), years lived with disability (YLDs), and years of life lost (YLLs) of child and adolescents causes of death in 2021 in Iran by sex

| DALYs (Disability-Adjusted Life Years) | Both Sexes | Province | Causes |  |  |  |  |  |  |  |  |  |  |  |  |  |  |  |  |  |  |  |  |  |
| --- | --- | --- | --- | --- | --- | --- | --- | --- | --- | --- | --- | --- | --- | --- | --- | --- | --- | --- | --- | --- | --- | --- | --- | --- |
|  |  |  | Cardiovascular diseases | Chronic respiratory diseases | Diabetes and kidney diseases | Digestive diseases | Enteric infections | HIV/AIDS and sexually transmitted infections | Maternal and neonatal disorders | Mental disorders | Musculoskeletal disorders | Neglected tropical diseases and malaria | Neoplasms | Neurological disorders | Nutritional deficiencies | Other infectious diseases | Other non-communicable diseases | Respiratory infections and tuberculosis | Self-harm and interpersonal violence | Sense organ diseases | Skin and subcutaneous diseases | Substance use disorders | Transport injuries | Unintentional injuries |
|  |  | Alborz | 239.3 (197.5 to 287.9) | 179.8 (114.7 to 271.7) | 52.8 (44.1 to 61.8) | 95.7 (76.6 to 122.1) | 122.7 (86.4 to 166.6) | 45.7 (22.7 to 85.9) | 633.9 (509.8 to 752.3) | 1824.3 (1287.4 to 2494.1) | 375.1 (256.1 to 526.1) | 47.7 (29 to 74.6) | 346.9 (265.3 to 420.2) | 570.2 (173.2 to 1132.6) | 269.6 (154.9 to 450.2) | 80.4 (59.2 to 121.3) | 862.3 (702.3 to 1059.3) | 666 (541.7 to 878.9) | 368.7 (289.8 to 448.1) | 195.2 (132.3 to 277.7) | 525.6 (338 to 760.4) | 83.5 (60.7 to 112.4) | 546 (450.7 to 656.2) | 482.4 (413.5 to 549.6) |
|  |  | Ardebil | 328.4 (267.7 to 397.3) | 181.6 (117.8 to 273) | 62.4 (51.4 to 74) | 98.9 (78.3 to 126) | 136.6 (93.5 to 188.2) | 46.3 (19.7 to 96.2) | 1225.1 (1033.6 to 1421.7) | 1713.4 (1171.1 to 2326.9) | 346.1 (234.5 to 483) | 67.9 (37.4 to 182.9) | 392.7 (260 to 525.8) | 569.2 (217.3 to 1085) | 319.3 (186.8 to 516) | 105.6 (72.3 to 189) | 1446.1 (1207.1 to 1766.4) | 507.6 (396.3 to 708.3) | 296.6 (247.5 to 350.7) | 189.2 (127.7 to 267) | 509.3 (331.9 to 742.6) | 38.4 (26.8 to 51.2) | 624 (515.9 to 759.9) | 443 (364.4 to 563.6) |
|  |  | Bushehr | 194.8 (159.1 to 233.4) | 172.4 (106 to 266.9) | 47.8 (40.2 to 57.1) | 99.9 (79.7 to 130.4) | 131.8 (93.3 to 180.3) | 49.1 (25.4 to 94.8) | 1176.7 (1001.7 to 1368.1) | 1768.2 (1243.8 to 2363.9) | 351.6 (233.5 to 489.4) | 57.5 (37.3 to 88.1) | 291.1 (197.5 to 370.5) | 553.4 (186.5 to 1113.3) | 344.6 (211.7 to 555.6) | 77.7 (61 to 101.8) | 1170.5 (985 to 1430.4) | 498.4 (382.9 to 683.5) | 237.5 (202.6 to 279.3) | 192 (130.9 to 275.7) | 522.7 (344.6 to 756.2) | 36.8 (25.7 to 49.5) | 737.4 (626.7 to 852.9) | 394.8 (338.1 to 471.9) |
|  |  | Chahar Mahaal and Bakhtiari | 198.6 (160.5 to 239.8) | 176.4 (109.1 to 278.6) | 35.7 (29.6 to 43.2) | 71.3 (54.6 to 92) | 119.6 (77.8 to 170.9) | 44.6 (17.4 to 93.8) | 769.3 (619.4 to 922.9) | 1564.8 (1090.1 to 2148.2) | 325.4 (216.5 to 453.4) | 54.7 (34.2 to 85.9) | 261.6 (166.9 to 346.8) | 534.6 (170.5 to 1053.4) | 305.9 (174.4 to 499.1) | 64.8 (46.1 to 94.7) | 951.5 (760 to 1188.2) | 408.7 (299.4 to 591.5) | 197.9 (164.1 to 233.3) | 186.7 (127.1 to 266.2) | 508.2 (328.4 to 736) | 28.5 (19.1 to 39.1) | 583.4 (485.4 to 678.5) | 365.1 (308.3 to 444.8) |
|  |  | East Azarbayejan | 279.7 (235.6 to 328.5) | 212.4 (146.5 to 309.3) | 75.4 (62.7 to 87.2) | 117.9 (95.9 to 148.6) | 128.7 (91.7 to 174.5) | 42.4 (20.5 to 81.4) | 796.1 (644.2 to 961.1) | 1627.3 (1120 to 2193.3) | 303.2 (195.7 to 446.6) | 59.8 (36 to 96.6) | 444.3 (296.6 to 575.7) | 640.8 (263.2 to 1220.9) | 320.5 (189.3 to 518.7) | 100.4 (73.2 to 159.8) | 1157 (957.8 to 1422.4) | 544 (425.3 to 766.2) | 275.6 (224.7 to 335.2) | 194.2 (131.9 to 274.4) | 519.2 (337.1 to 750.1) | 64.5 (47.4 to 87.1) | 904.5 (762.3 to 1067.3) | 480.8 (415.3 to 569.8) |
|  |  | Fars | 318.6 (266.1 to 371) | 190.6 (126.7 to 285) | 71.1 (60.6 to 84.2) | 142.2 (110.2 to 182.9) | 132.2 (94.4 to 178.8) | 62.7 (39 to 106.4) | 1201.9 (957.1 to 1463.7) | 1867.5 (1318.1 to 2566.5) | 355.5 (239 to 493.1) | 62.2 (37.3 to 116.9) | 443.7 (313 to 580.7) | 604.3 (216.6 to 1174.6) | 344 (209.6 to 557.3) | 110.2 (86.1 to 161.6) | 1403.9 (1169 to 1693.7) | 586.3 (465.5 to 802.4) | 432.7 (355.7 to 531) | 191 (128.8 to 272.4) | 520.4 (341.7 to 749.6) | 82.8 (60 to 109.4) | 1364.8 (1164 to 1552) | 585.1 (514.4 to 676.5) |
|  |  | Gilan | 313 (254.6 to 380.3) | 186.3 (118.3 to 280.5) | 53.1 (44.7 to 64.3) | 83.9 (66.8 to 106.2) | 116.8 (78.7 to 167.3) | 33.7 (14.3 to 66.7) | 449.6 (341.2 to 561.1) | 1756.7 (1191.2 to 2384.1) | 386.1 (254.3 to 543.7) | 47 (29.3 to 71.3) | 288.9 (220.7 to 359.1) | 585.2 (185.2 to 1186.3) | 283 (168.5 to 460.4) | 67.3 (47.8 to 101.3) | 753.5 (610 to 931.8) | 490.3 (375.1 to 710.8) | 283.1 (235.7 to 341.6) | 188.5 (127.9 to 273.5) | 526.6 (336.9 to 762.1) | 53.3 (37.8 to 71) | 892.7 (746.6 to 1047.4) | 482.4 (408.8 to 573.9) |
|  |  | Golestan | 337.3 (281.8 to 397.1) | 196 (131.7 to 290.8) | 70.8 (59.7 to 82.1) | 144.6 (109.5 to 187.4) | 144.3 (105.5 to 193.9) | 49.2 (22.8 to 95.8) | 1188.7 (1010.4 to 1372.9) | 1554.6 (1078.3 to 2118.3) | 334.4 (223.7 to 466.3) | 63.1 (38.7 to 94.6) | 356.7 (284.6 to 448.6) | 589.2 (211.1 to 1129.5) | 372.5 (215.8 to 596.7) | 144.7 (101.6 to 235) | 1350.9 (1152.9 to 1603.2) | 583.8 (464.9 to 776.8) | 322 (266.1 to 385.2) | 188.5 (128.4 to 267.4) | 511 (335.4 to 737.4) | 38.6 (27.7 to 51.4) | 1129.1 (969.3 to 1285.7) | 514.9 (435.3 to 627.3) |
|  |  | Hamadan | 348.5 (295 to 408.4) | 195 (130.1 to 286.1) | 66.5 (56.8 to 77.6) | 139.5 (110.3 to 183.7) | 149.8 (109.9 to 200.4) | 46.6 (21.6 to 93.7) | 1327.7 (1142.2 to 1535.3) | 1589.8 (1098.2 to 2163.9) | 339.1 (223.3 to 474.5) | 61 (38.6 to 90.6) | 449.8 (314.7 to 580.7) | 586.6 (231.5 to 1118.9) | 355.1 (203.2 to 555.5) | 135.7 (103.7 to 195.8) | 1358.7 (1155.5 to 1624) | 537.1 (419.5 to 741) | 355.2 (277.3 to 429.6) | 188.2 (128.5 to 267.7) | 511 (335.9 to 749.9) | 101.2 (72.1 to 138.8) | 1160.4 (1004.4 to 1332.9) | 546.5 (470.8 to 641.2) |
|  |  | Hormozgan | 313.7 (264.2 to 372.7) | 180.2 (117.2 to 276.5) | 76.3 (56.6 to 91.2) | 134.8 (104.3 to 172.4) | 168.1 (128.9 to 221.1) | 70.3 (43.5 to 119.7) | 1318.6 (1115.6 to 1528.8) | 1655.9 (1146.1 to 2241) | 326.1 (222.4 to 448) | 65.8 (42.1 to 95.8) | 293.1 (235.1 to 359.7) | 554 (202 to 1082.3) | 403.2 (252.1 to 617.9) | 113.1 (87.3 to 163.4) | 1298.8 (1097.8 to 1549.3) | 589.4 (477.2 to 799.5) | 280.6 (228.2 to 340.7) | 186.4 (125.6 to 266.7) | 508.8 (331.5 to 742.2) | 73.3 (50.6 to 101) | 1129.3 (944.5 to 1307) | 543.7 (466.7 to 646.8) |
|  |  | Ilam | 256.7 (209.5 to 311.6) | 174.8 (108.2 to 273.8) | 69.4 (53.6 to 82.5) | 94.3 (73.2 to 120.8) | 133.8 (95.4 to 179.7) | 47.9 (20.1 to 101.7) | 1270.5 (957.9 to 1598.7) | 1704.6 (1170.6 to 2380.8) | 330.3 (224.9 to 468.4) | 57.5 (35.1 to 88.5) | 311.2 (212.6 to 426.1) | 526.3 (175.9 to 1029.3) | 333.7 (193.2 to 544.1) | 81.6 (60.9 to 113) | 1269.3 (978.4 to 1616.7) | 540.9 (421 to 729.5) | 511 (375.2 to 601.2) | 184.7 (124.8 to 264) | 510.6 (332.5 to 743.3) | 29.6 (20 to 40.4) | 739.1 (595.7 to 895.9) | 455.6 (376.4 to 545.5) |
|  |  | Isfahan | 258.3 (213.6 to 305.8) | 189.2 (123.9 to 285.7) | 64.4 (54 to 75.3) | 106.2 (86 to 134.1) | 124.6 (86.5 to 172.7) | 36.3 (16.5 to 72.4) | 905.2 (709.7 to 1121.6) | 1764.8 (1227.8 to 2361.9) | 368.4 (250.4 to 514.3) | 52.6 (34 to 80.4) | 397.9 (258.7 to 504.7) | 593.3 (205.6 to 1171.1) | 331.3 (197.4 to 550.3) | 92 (68.6 to 130.2) | 1163.8 (962.8 to 1434.6) | 564.5 (436 to 765.8) | 229.5 (192 to 277.4) | 193.2 (132.3 to 274.1) | 523.5 (340.1 to 767.1) | 54.7 (38.7 to 75) | 795.5 (668.9 to 947.6) | 526 (460.8 to 603.7) |
|  |  | Kerman | 302.7 (253.6 to 354.7) | 227.4 (159.8 to 325) | 73.1 (59.3 to 87.5) | 134.7 (106.3 to 173.9) | 172.2 (131.3 to 223.8) | 52.6 (28 to 99.6) | 1607.7 (1379.5 to 1863.1) | 1834.5 (1278.2 to 2499.1) | 344.3 (230.7 to 480.8) | 63.9 (42 to 95.2) | 388.1 (274.4 to 500.9) | 581.6 (201.8 to 1143) | 385.2 (230.4 to 621.4) | 143.1 (94.9 to 289) | 1385.8 (1171.5 to 1670.6) | 626.9 (504.7 to 816.4) | 305.5 (252.8 to 368.9) | 188.2 (127.6 to 266.5) | 512.2 (330.6 to 744.5) | 53 (37.3 to 72.4) | 1202.7 (1044.4 to 1356.2) | 531.6 (468.6 to 633.8) |
|  |  | Kermanshah | 289.2 (239 to 339.7) | 192.6 (129 to 282.7) | 65.6 (56 to 76.3) | 124.7 (99.1 to 162.6) | 135.5 (97.7 to 181.8) | 72.9 (48.2 to 118.2) | 1428.8 (1217.7 to 1671) | 1710.3 (1192.8 to 2307.1) | 340.5 (229.4 to 475.8) | 59.1 (36.4 to 87.5) | 399.7 (270.7 to 523) | 538.8 (171.3 to 1066.7) | 350.8 (205.6 to 554.3) | 131.2 (85.5 to 258.8) | 1292.9 (1075.3 to 1577.7) | 590.4 (475 to 825.3) | 473.5 (376.5 to 559.7) | 188.2 (126.8 to 267.5) | 510.9 (333.6 to 749.1) | 93.4 (68.4 to 126.6) | 914.9 (763.6 to 1083.1) | 509.3 (443.5 to 586) |
|  |  | Khorasan-e-Razavi | 256.1 (202.5 to 309.2) | 203.2 (140.3 to 298.7) | 70.3 (59 to 84.3) | 129.4 (101 to 171.4) | 142.3 (105.8 to 186.4) | 46.5 (22 to 88) | 1207 (935.1 to 1498.2) | 1586.2 (1087.2 to 2174.5) | 341.2 (231.4 to 471.6) | 62.6 (40.4 to 89.7) | 421.6 (268.5 to 578.2) | 571.1 (215.8 to 1060.7) | 341 (202.4 to 522.3) | 114.4 (86 to 169.3) | 1305.8 (1039.8 to 1652.9) | 623.9 (499.1 to 833.7) | 254.6 (215.5 to 306.3) | 236.8 (166 to 337.6) | 511.8 (334.2 to 742.5) | 66.9 (46.7 to 88.3) | 955.2 (782.5 to 1131.3) | 498.9 (421 to 574.6) |
|  |  | Khuzestan | 301 (252 to 354.9) | 174.3 (115.4 to 262.1) | 74 (63.4 to 86.2) | 121.7 (98.7 to 157.1) | 142.8 (105.8 to 192.4) | 55.2 (29.4 to 105) | 1228.8 (1011.6 to 1429.4) | 1656.7 (1151.4 to 2265.8) | 324.6 (217.2 to 453.8) | 60.1 (37.4 to 90.9) | 379.1 (290.1 to 469.9) | 550.1 (219 to 1049.4) | 362.8 (213.7 to 580.3) | 129.4 (91.5 to 232.6) | 1461.5 (1229.8 to 1757.2) | 650.3 (526.9 to 830.5) | 336.9 (286.3 to 398.4) | 182.3 (125.5 to 261.7) | 512.9 (334.4 to 749.4) | 52.6 (39.1 to 71.4) | 855.7 (726.5 to 993.5) | 693.5 (620 to 782.1) |
|  |  | Kohgiluyeh and Boyer-Ahmad | 355.3 (298.6 to 415.9) | 187.9 (120.2 to 289.6) | 61.3 (52.8 to 70.4) | 112.4 (93.3 to 141.8) | 146.3 (106.5 to 194.6) | 68.6 (29.1 to 138.5) | 1160.2 (955 to 1371.5) | 1596.9 (1112.6 to 2143.8) | 308.7 (206 to 436) | 63.2 (39.5 to 97.4) | 450.8 (306.6 to 585) | 532.6 (193.4 to 1030.2) | 340.5 (202.4 to 558.5) | 125.8 (98.4 to 162.5) | 1327.7 (1114.7 to 1606.6) | 508.8 (397 to 700.9) | 411.2 (320.5 to 491.9) | 181.1 (121.9 to 257.7) | 503.9 (324.1 to 734.3) | 36 (26.1 to 47.9) | 1081.8 (900.6 to 1240.8) | 659.2 (577.8 to 749.3) |
|  |  | Kurdistan | 262.9 (219.8 to 313.4) | 186.8 (122.6 to 283.6) | 56 (47 to 66) | 115.6 (92.3 to 150.8) | 143.6 (106.6 to 194.2) | 48.6 (22.7 to 96.3) | 1488 (1273.4 to 1719.7) | 1639.4 (1120.4 to 2248.6) | 338.7 (228.1 to 474.4) | 59.3 (37.9 to 90.5) | 383 (247.7 to 519.3) | 559.2 (207.3 to 1099.4) | 335.2 (194.9 to 538) | 98.9 (75.8 to 131.8) | 1254.9 (1034.1 to 1525.1) | 501.7 (390.9 to 686.3) | 305.1 (262.2 to 359.3) | 181.4 (122.3 to 256.5) | 508 (334.2 to 735.6) | 36.9 (26.2 to 49.1) | 960.8 (818.6 to 1107.8) | 497.9 (429.3 to 589.1) |
|  |  | Lorestan | 184.1 (151.4 to 219.1) | 172.8 (105.5 to 262.8) | 40 (33.3 to 48.3) | 81.8 (65 to 108.4) | 117 (81.1 to 162.3) | 52.7 (27.9 to 96.3) | 687.7 (550.9 to 821.5) | 1546.4 (1072.1 to 2099.3) | 330.7 (222.9 to 464.2) | 53.4 (34.2 to 82.8) | 253.5 (176 to 328.7) | 516.1 (172.9 to 1047.6) | 331.6 (205.2 to 536.2) | 81.8 (59.8 to 122.5) | 781.5 (625 to 985.5) | 544.9 (427.6 to 762.8) | 275.3 (200.7 to 336) | 186.5 (125.4 to 266.3) | 506.1 (324.5 to 746.5) | 53 (37.1 to 71.2) | 517.6 (433.2 to 608.1) | 311.4 (259.3 to 394.7) |
|  |  | Markazi | 240.9 (198.1 to 287.7) | 192.6 (129.7 to 287.8) | 66.2 (55.4 to 78.2) | 114 (91.2 to 148.4) | 123.4 (85.6 to 168) | 40.6 (18.9 to 79.7) | 1029 (867 to 1204.1) | 1780.4 (1270.1 to 2393.7) | 361 (238.7 to 506.1) | 53.9 (34 to 81.1) | 413.4 (263.6 to 540.7) | 585 (216.5 to 1134.7) | 319 (181.7 to 535) | 90.9 (72.9 to 116.2) | 1061.5 (873.1 to 1299.9) | 621.3 (493.4 to 829.9) | 224.3 (183.7 to 270.7) | 193 (130.8 to 273.5) | 519.4 (339.1 to 749.6) | 59.9 (41.7 to 81.9) | 949.9 (800.5 to 1084.3) | 454.6 (394.6 to 525.1) |
|  |  | Mazandaran | 258.6 (212.4 to 308.8) | 180.7 (116.3 to 274.4) | 57.8 (49.3 to 67.9) | 85.8 (68.2 to 108.9) | 119 (78.3 to 169.4) | 36.6 (15.9 to 74.5) | 577.5 (462.5 to 694.9) | 1838.3 (1257 to 2544.9) | 502.4 (338.8 to 695.2) | 46.8 (29.3 to 71.7) | 328.8 (230.4 to 410.7) | 580.3 (187.8 to 1160.1) | 277.8 (164.8 to 455.4) | 70.7 (53.3 to 100.7) | 928.8 (755.3 to 1135.1) | 492.7 (377.5 to 723.4) | 246.2 (210.7 to 292) | 194.3 (129.8 to 277.5) | 526.6 (339 to 768.1) | 37.9 (26.5 to 51.2) | 856.2 (730.3 to 992.4) | 497 (429.5 to 582.5) |
|  |  | North Khorasan | 310.2 (258.1 to 363.6) | 203.3 (138.7 to 296) | 76.2 (64.4 to 89.7) | 136.8 (109.3 to 177.2) | 152.5 (116 to 198.3) | 49.3 (23.1 to 98.2) | 1111.8 (950.7 to 1289.2) | 1673.7 (1165.8 to 2282.9) | 334.1 (222.1 to 466.1) | 73.9 (42.7 to 143.2) | 419.2 (292.5 to 542.3) | 582.2 (250.7 to 1100.7) | 367.5 (216 to 598.4) | 121.3 (95.7 to 169.5) | 1280.9 (1068.8 to 1542.5) | 709.8 (572.3 to 912.8) | 288.9 (240.2 to 340.5) | 187 (127.2 to 264.5) | 508 (331.1 to 742.2) | 46.9 (33.8 to 61.3) | 1092.4 (926.5 to 1240.1) | 539.6 (467.6 to 618.6) |
|  |  | Qazvin | 204.1 (162.3 to 252.1) | 177.8 (113.8 to 272.1) | 47.4 (39.3 to 55.4) | 129.8 (97.4 to 169.4) | 122.6 (84.1 to 170.8) | 41.5 (18.1 to 83.6) | 870.8 (706.1 to 1026.6) | 1641.1 (1144 to 2258.5) | 348.9 (230.5 to 480.9) | 52.1 (31.7 to 78.7) | 317.5 (205.6 to 414.1) | 551.1 (193.9 to 1104.5) | 312.1 (182.1 to 522) | 74.6 (58.1 to 96.5) | 980.1 (790 to 1224.7) | 581.2 (465.2 to 801.9) | 194.4 (163.5 to 231.7) | 190.8 (130.2 to 276.9) | 517.1 (339.4 to 747.8) | 65.5 (46.9 to 87.2) | 678.5 (555.6 to 809.1) | 415.5 (352.4 to 491.3) |
|  |  | Qom | 162.7 (128.9 to 199.1) | 183.6 (118.2 to 279.1) | 57.3 (47.4 to 67.7) | 107.6 (85 to 139) | 119.4 (82.9 to 163) | 54.4 (31.2 to 95.1) | 749.1 (601.3 to 895.4) | 1726.2 (1209.3 to 2328) | 339.4 (222.8 to 479.5) | 55.7 (33.6 to 91.5) | 375.8 (235.4 to 482.4) | 547.7 (183.3 to 1058.6) | 302.7 (174.8 to 503.5) | 76 (56.4 to 108.5) | 959.3 (784.5 to 1173.9) | 566.5 (448.7 to 761.9) | 194.2 (158.9 to 240.1) | 190.2 (129.1 to 268.4) | 524.6 (345.7 to 759.8) | 38.9 (27 to 52) | 392.9 (313.2 to 515.1) | 448.2 (359.9 to 529.9) |
|  |  | Semnan | 199.8 (162 to 247.8) | 180 (116.8 to 271.5) | 58.3 (47.7 to 69.6) | 97.2 (74.9 to 126.8) | 120.5 (81.8 to 164.7) | 38 (14.4 to 84.2) | 979.3 (741.9 to 1246.6) | 1712.7 (1209.3 to 2269.9) | 361.4 (238.5 to 500) | 52.5 (33.8 to 80.1) | 295.3 (207.4 to 394.2) | 590.1 (213.7 to 1136.8) | 312.2 (181.9 to 512) | 68.3 (51.9 to 91.7) | 1070.3 (832.2 to 1371.7) | 709.2 (583.2 to 924.7) | 187 (158.7 to 223.4) | 181.6 (124.5 to 259.2) | 523.2 (344 to 759.8) | 49.3 (35.2 to 67.3) | 713.1 (583.7 to 863.9) | 371.8 (297.1 to 448.2) |
|  |  | Sistan and Baluchistan | 421.2 (358.3 to 487.6) | 266.1 (197.6 to 363.1) | 122.3 (69.1 to 144.9) | 224 (155 to 284.3) | 232.9 (176.3 to 305.8) | 84.7 (41.3 to 158.3) | 2102.4 (1777.8 to 2428.6) | 1602.5 (1110.5 to 2203.9) | 307 (202.3 to 424.2) | 105.9 (65.3 to 148.3) | 374.7 (299.3 to 474.8) | 561.2 (240 to 1076.8) | 524.8 (330.4 to 805.9) | 306 (177.1 to 579.3) | 1689.7 (1398.5 to 2074.7) | 751 (627 to 934.1) | 339.1 (283.3 to 407.7) | 187 (128.6 to 266.6) | 490.6 (318.2 to 717.3) | 61.1 (46.6 to 79.5) | 1285.5 (1107.3 to 1452.8) | 699.4 (602.4 to 840.6) |
|  |  | South Khorasan | 226.7 (183.2 to 278.7) | 213.8 (146.8 to 310.2) | 66.6 (55.7 to 79.7) | 128.5 (97.8 to 167.6) | 137.5 (99.4 to 181) | 42.3 (17.1 to 87.3) | 1268 (1072.7 to 1474.9) | 1705.5 (1158 to 2271.1) | 337.5 (223.7 to 473.2) | 60.7 (39.1 to 92.8) | 341.5 (210.5 to 466) | 547.9 (187 to 1056.3) | 348.2 (208.8 to 567) | 122.7 (84.4 to 219.9) | 1125.4 (909.2 to 1409.5) | 615.1 (492.1 to 802.3) | 174.6 (150.4 to 200.7) | 188.5 (128.2 to 269) | 509.1 (330.5 to 740.2) | 49.6 (35 to 65.7) | 831.3 (705 to 955.9) | 430.1 (375.1 to 508.8) |
|  |  | Tehran | 197.4 (163.5 to 241.6) | 179.2 (114 to 275.9) | 63.8 (53 to 75.9) | 99.1 (79.1 to 122.8) | 112.4 (76.1 to 157.2) | 51.8 (28.4 to 102.2) | 677.2 (470.5 to 921.1) | 1798.3 (1251.6 to 2444.4) | 403.7 (276.5 to 562.8) | 41.5 (26.8 to 63.3) | 367.1 (262.8 to 445.6) | 598.4 (203.5 to 1128) | 267.3 (168.9 to 427.1) | 80.7 (59.4 to 120.8) | 960.8 (759.8 to 1213.5) | 525.2 (409.6 to 725.6) | 169.1 (141.8 to 218) | 185.4 (124.7 to 261.9) | 527.8 (343.2 to 760.4) | 119.9 (86.3 to 157.7) | 141.8 (109.5 to 237.5) | 400.9 (326 to 480.6) |
|  |  | West Azarbayejan | 307.5 (253.3 to 361.1) | 209 (144.8 to 308.6) | 66.6 (56.7 to 78.3) | 123.4 (100.9 to 157.1) | 151 (110.5 to 201.3) | 46.5 (21.6 to 91.5) | 1260.1 (1068.1 to 1467.9) | 1788.7 (1269.8 to 2354.9) | 345.8 (232 to 481.4) | 58.9 (36.9 to 88.2) | 456.1 (296.9 to 608.3) | 587.2 (225.7 to 1094.2) | 338.3 (194.6 to 541.9) | 126.4 (85.2 to 234.4) | 1399.1 (1178.4 to 1689.8) | 569.1 (452.6 to 782.1) | 428.2 (355.5 to 505.3) | 192.3 (129.3 to 268.3) | 508 (325.9 to 741.1) | 32.2 (22.9 to 43.1) | 938.2 (794.1 to 1093.1) | 520.8 (448.6 to 628.7) |
|  |  | Yazd | 198.1 (159 to 241.9) | 192.3 (127.7 to 290.9) | 65.4 (55.5 to 76.4) | 97.8 (79.5 to 126.1) | 131.8 (92.3 to 179.3) | 45.8 (22.8 to 88.3) | 1210.3 (1019.1 to 1422.6) | 1621 (1118.9 to 2238.9) | 349.3 (234.7 to 484) | 52.5 (33.5 to 79.8) | 439.8 (272.5 to 559.8) | 566.3 (192.9 to 1086.9) | 334.8 (205.3 to 551.1) | 86.7 (63.7 to 126.9) | 1341.6 (1123.9 to 1619.8) | 774.6 (642.6 to 964.4) | 170.1 (142.4 to 205.3) | 179.1 (123.2 to 256.9) | 523.5 (342.3 to 762.7) | 49.3 (35.2 to 66.6) | 805.7 (659.7 to 971) | 455.3 (390.8 to 524.3) |
|  |  | Zanjan | 232.4 (192.3 to 282.3) | 183.9 (118.9 to 280.8) | 48.1 (39.5 to 57.4) | 104.6 (81.5 to 137.4) | 141.5 (100.5 to 189.8) | 45.2 (20.1 to 89.3) | 1258.7 (1034.7 to 1500.8) | 1695.9 (1194.6 to 2308.4) | 336.5 (219.4 to 474.9) | 57.3 (36.5 to 89.2) | 303.8 (204 to 395.2) | 538.3 (178.6 to 1044.1) | 315.8 (188.7 to 555.1) | 96.7 (76.3 to 130.4) | 1114.5 (895.7 to 1372.9) | 608.2 (485.6 to 813.6) | 177.9 (151.8 to 209.9) | 188.4 (128.9 to 266.9) | 509 (334 to 741.5) | 40.6 (28.8 to 55.3) | 766.7 (660.2 to 881.5) | 431.1 (374.4 to 497.9) |
| DALYs (Disability-Adjusted Life Years) | Females | Province | Causes |  |  |  |  |  |  |  |  |  |  |  |  |  |  |  |  |  |  |  |  |  |
|  |  |  | Cardiovascular diseases | Chronic respiratory diseases | Diabetes and kidney diseases | Digestive diseases | Enteric infections | HIV/AIDS and sexually transmitted infections | Maternal and neonatal disorders | Mental disorders | Musculoskeletal disorders | Neglected tropical diseases and malaria | Neoplasms | Neurological disorders | Nutritional deficiencies | Other infectious diseases | Other non-communicable diseases | Respiratory infections and tuberculosis | Self-harm and interpersonal violence | Sense organ diseases | Skin and subcutaneous diseases | Substance use disorders | Transport injuries | Unintentional injuries |
|  |  | Alborz | 210 (167.7 to 260.4) | 160.5 (103.6 to 246.5) | 53.7 (42.6 to 65.5) | 94.5 (74 to 120.3) | 125.5 (86.6 to 172.5) | 40.8 (20.6 to 77.1) | 573.5 (450.6 to 694.8) | 1948.3 (1345.3 to 2675.5) | 514.3 (349.5 to 720.5) | 57.9 (34.2 to 93.7) | 360.2 (225.9 to 455.3) | 624.4 (161.8 to 1278.2) | 376.8 (205.9 to 652.2) | 84.8 (60 to 125.4) | 854.1 (673.6 to 1080.5) | 593 (471.6 to 814) | 257.3 (202.4 to 320.2) | 194.9 (133 to 278.1) | 556.6 (361.2 to 800.5) | 42.6 (29.1 to 59.3) | 327.1 (259.8 to 405.3) | 318 (271.7 to 367.3) |
|  |  | Ardebil | 276.5 (220.8 to 335.5) | 164.5 (103.2 to 245) | 61.5 (49.4 to 73.8) | 101.6 (78.4 to 130.3) | 136.7 (89.6 to 190) | 46.9 (21.2 to 94.7) | 1131.1 (929.8 to 1333.9) | 1816.6 (1225.1 to 2545.1) | 473.6 (317.9 to 668.9) | 73 (41.7 to 144.9) | 344.8 (217.5 to 459.2) | 612.3 (204.7 to 1235.7) | 410 (226.3 to 690.3) | 117.2 (78.8 to 211.1) | 1433.4 (1181.2 to 1789) | 470.9 (356.5 to 684) | 219.5 (176.3 to 266.1) | 190.2 (129.6 to 270.2) | 542.3 (353.5 to 789.5) | 24.8 (16.6 to 35.7) | 339.7 (264 to 451.4) | 284.1 (239.3 to 352.3) |
|  |  | Bushehr | 175.6 (143.3 to 213.6) | 156.9 (97.9 to 237.6) | 48.1 (39.8 to 57.6) | 103.2 (79.9 to 136.4) | 137.4 (98.2 to 187.6) | 45.2 (22.8 to 85.9) | 1105.8 (928.8 to 1283.4) | 1865.1 (1284 to 2499.7) | 477.8 (322.1 to 666.2) | 70.9 (43.4 to 110.6) | 286.2 (182 to 364.7) | 604.6 (179.2 to 1260.9) | 478.1 (273.3 to 756.7) | 89.2 (68.4 to 119.3) | 1215.5 (1013 to 1518.8) | 462.6 (350 to 654.7) | 231.9 (186.8 to 278) | 192.6 (132.4 to 274.9) | 552 (361.7 to 797.2) | 23.3 (15.3 to 33.7) | 395 (333 to 459.7) | 283.3 (241.6 to 337.6) |
|  |  | Chahar Mahaal and Bakhtiari | 178 (142.3 to 218) | 157.8 (96.5 to 249.2) | 35.4 (28.6 to 44.4) | 72.6 (54.7 to 94.8) | 116.4 (75.6 to 169.1) | 42 (16.1 to 89) | 722.2 (579.2 to 864.5) | 1648.5 (1110.3 to 2304.6) | 443.2 (288.8 to 623) | 68.2 (39.4 to 108.4) | 237.6 (153.4 to 314.7) | 581.1 (179.9 to 1189.3) | 440.1 (238.9 to 733) | 75.9 (52.5 to 110.2) | 987.6 (792.5 to 1273.3) | 381.4 (271.1 to 561.9) | 153.3 (114.2 to 191) | 187 (127.1 to 269.7) | 541.2 (349.3 to 793.8) | 18.5 (11.1 to 27.4) | 341 (276.1 to 399.1) | 223.8 (187 to 272.6) |
|  |  | East Azarbayejan | 255.1 (204.2 to 305.9) | 202.8 (135.8 to 294.3) | 80.9 (65.5 to 97.5) | 124.7 (98.6 to 156.8) | 134.9 (93.4 to 188.1) | 41.9 (20.7 to 79.4) | 761.6 (617 to 922.1) | 1696.1 (1138.2 to 2338.4) | 403 (257.4 to 589) | 68.7 (41.6 to 108.3) | 448 (282.7 to 575.8) | 690.7 (242.7 to 1378) | 415.7 (231.5 to 736.3) | 108.6 (77.8 to 175.4) | 1192.6 (974.5 to 1481.2) | 514.5 (396.8 to 735) | 208.7 (166.4 to 258.7) | 194.3 (131.4 to 279.2) | 550.3 (354.1 to 801.7) | 38.2 (26.2 to 53.4) | 523.4 (425.5 to 655.5) | 345.5 (295.6 to 421) |
|  |  | Fars | 278.8 (227.9 to 325.1) | 171.5 (109.6 to 261.5) | 67.8 (56.2 to 83.1) | 137.8 (106.1 to 179.7) | 132.9 (92 to 183.5) | 55.9 (33.4 to 98.5) | 1114.4 (882.2 to 1345) | 1936.2 (1354.7 to 2666.9) | 486.1 (328.3 to 687) | 73.2 (42.4 to 114.9) | 400.2 (254.7 to 514) | 658.2 (200.9 to 1314.5) | 463 (258.8 to 757.9) | 115.9 (84.9 to 171.9) | 1350.9 (1105.5 to 1692) | 538.7 (415.1 to 761.1) | 284.8 (227.4 to 353.4) | 191.7 (128.8 to 276.6) | 552.1 (358.1 to 799.4) | 47.5 (31.3 to 68.2) | 718.7 (583.9 to 840.4) | 401.1 (347.5 to 470.8) |
|  |  | Gilan | 244.2 (195.8 to 297) | 159.7 (98.8 to 240.6) | 49.7 (41.4 to 61.1) | 82.3 (62.8 to 104.4) | 117.1 (78.7 to 172.5) | 32.5 (14.9 to 63.6) | 435.2 (335.7 to 544.5) | 1893.9 (1256 to 2585.1) | 525.8 (346.9 to 742.6) | 57.3 (35.1 to 89.4) | 235.9 (169.3 to 298.1) | 637.1 (188.1 to 1347.4) | 375.2 (206.2 to 617.8) | 70.6 (46.7 to 108.4) | 774.8 (613.5 to 976) | 450.5 (331.9 to 683.5) | 174.6 (143.1 to 211.1) | 188 (125.4 to 267.4) | 557.2 (360.5 to 800.6) | 28.2 (18.3 to 39.7) | 328.5 (266.2 to 402.7) | 238.5 (199.6 to 297) |
|  |  | Golestan | 253.5 (205.4 to 301.6) | 167.4 (107.5 to 243.3) | 61.6 (51.5 to 73.8) | 131.9 (99.7 to 173.5) | 142.2 (98.5 to 194.4) | 46.2 (21.2 to 93.8) | 1080.9 (902.2 to 1267.9) | 1610.3 (1095.9 to 2224.9) | 454.4 (301.5 to 636.2) | 70.6 (44.7 to 112.2) | 266.7 (199.4 to 338.4) | 619.2 (195.9 to 1228.6) | 448.8 (254.7 to 772.8) | 139.5 (95.1 to 238.9) | 1250.6 (1034.9 to 1551.3) | 520.4 (400.2 to 720.2) | 240.1 (175.3 to 292.1) | 189.1 (129 to 270.3) | 542.1 (353.5 to 781.8) | 23.2 (15.5 to 33.2) | 390.9 (309.1 to 479.9) | 279.8 (234 to 355.5) |
|  |  | Hamadan | 285.7 (231.2 to 342.8) | 171.3 (113.2 to 250.7) | 63.4 (53.9 to 76.2) | 135.6 (103.6 to 176.3) | 149.2 (106.7 to 202.7) | 43.4 (20 to 87.3) | 1274.5 (1064.8 to 1514.9) | 1678.7 (1146 to 2323.8) | 464.1 (309.4 to 657.5) | 71.5 (44.1 to 111.8) | 389.2 (247 to 516.4) | 630.3 (233.2 to 1234.6) | 452.9 (250.7 to 720) | 139.3 (104.1 to 201) | 1307.3 (1067.4 to 1645.1) | 492.4 (368.3 to 694.5) | 227.1 (176.8 to 281.5) | 188.9 (129.4 to 272.8) | 542 (354.3 to 784.3) | 51.1 (34.8 to 71.3) | 648.8 (538.2 to 756.2) | 333.1 (282.2 to 398.6) |
|  |  | Hormozgan | 228.3 (188.5 to 272.2) | 157.3 (99.4 to 236.4) | 64.1 (50.7 to 76.4) | 125.8 (95.2 to 166.3) | 185.4 (133.5 to 257.2) | 69.1 (43.9 to 115.2) | 1178.3 (971.1 to 1378.1) | 1742.9 (1178.7 to 2408.7) | 442.4 (297.8 to 611) | 74.7 (46.7 to 115.5) | 242.5 (178.9 to 310) | 586.7 (182.1 to 1191.7) | 487 (278.3 to 812) | 117.8 (86.8 to 169.8) | 1234.6 (1010.8 to 1574.6) | 537.8 (425.7 to 757.1) | 140.6 (114.5 to 176.5) | 187.3 (125.6 to 272.5) | 539.1 (351.4 to 786) | 39.3 (24.6 to 57.5) | 451.4 (371.3 to 528.3) | 321.9 (268.4 to 392.6) |
|  |  | Ilam | 214 (172.7 to 266.8) | 156.2 (96.2 to 244.1) | 60.3 (47.6 to 75) | 95.3 (71.8 to 126) | 137.5 (94.8 to 187.7) | 47.4 (20.9 to 98.2) | 1144.7 (833.7 to 1439.3) | 1848.8 (1239.4 to 2571.7) | 449 (300.7 to 636.8) | 66.7 (39.6 to 105.7) | 264.8 (177.8 to 353.8) | 565.3 (170.4 to 1149.8) | 444.6 (249.9 to 754.7) | 83.8 (58.2 to 119.2) | 1247.4 (966 to 1633.8) | 507.6 (384.6 to 694.7) | 364.2 (250.5 to 441.6) | 185.4 (124.3 to 267.1) | 543.3 (354.4 to 789.3) | 17.5 (10.5 to 26.1) | 369.5 (286.2 to 468.9) | 299.7 (247.6 to 356.2) |
|  |  | Isfahan | 222.7 (176 to 275.3) | 169.6 (108 to 258) | 63.2 (51.4 to 76.2) | 108.1 (83.8 to 140.6) | 125.9 (86.2 to 176.6) | 34.7 (16.4 to 70.7) | 828.7 (647.1 to 1018.4) | 1955.7 (1330 to 2639.6) | 503.8 (341.9 to 695.8) | 65.1 (38.9 to 104.8) | 373.7 (227.3 to 471.4) | 641.9 (195 to 1303.8) | 448.2 (264.5 to 751.5) | 103.2 (75.6 to 146.8) | 1159.2 (953.3 to 1446.6) | 518.3 (390 to 728.4) | 154.2 (124.4 to 203) | 194 (132.5 to 277.4) | 553.9 (361.2 to 802.5) | 26.9 (17.8 to 37.2) | 430 (335.6 to 521.5) | 344.5 (297.8 to 401.7) |
|  |  | Kerman | 273.3 (226.1 to 325.9) | 211.3 (145.3 to 297.5) | 75 (62.7 to 89.9) | 134.5 (103.1 to 173.9) | 189.1 (138.6 to 250) | 50.4 (27.2 to 94.4) | 1498.7 (1283.8 to 1728.7) | 1922.8 (1322.1 to 2666.4) | 469.8 (317.3 to 656.3) | 73 (46.3 to 114.1) | 384.9 (248.7 to 485.4) | 612.7 (187.1 to 1256) | 470.6 (272.4 to 794) | 151.8 (99.2 to 301.5) | 1360.1 (1108.9 to 1741.9) | 588.2 (466.3 to 775) | 215.6 (176.2 to 269.4) | 189.3 (126.7 to 272.3) | 543.2 (352.9 to 794.1) | 34.6 (23.6 to 49.4) | 703.5 (570.1 to 810.3) | 403.5 (347.7 to 486.8) |
|  |  | Kermanshah | 246.5 (199.7 to 298.1) | 171.6 (111.5 to 254.6) | 63.2 (50.6 to 76.7) | 120.2 (93.1 to 157.9) | 143.5 (99.4 to 200.5) | 64 (40 to 109.2) | 1349.6 (1134.3 to 1598.6) | 1784.5 (1207.6 to 2407.8) | 468 (317.9 to 654.3) | 67.9 (41.7 to 108.9) | 349.8 (233.5 to 441.3) | 585.9 (167.5 to 1209.9) | 455.1 (256 to 733.6) | 137.4 (84.5 to 280.8) | 1257.1 (1009.7 to 1616.3) | 537.3 (416.2 to 763.4) | 398.4 (266.2 to 500.9) | 188.3 (129.2 to 267.9) | 541.2 (349.9 to 799) | 54.7 (38.2 to 76.7) | 450 (364.1 to 556) | 376.4 (315.1 to 446.5) |
|  |  | Khorasan-e-Razavi | 231 (185.3 to 283.3) | 188.2 (124.1 to 282.9) | 66.7 (53.5 to 82.5) | 133.1 (99.3 to 181.8) | 154 (107.5 to 206.2) | 45.6 (22.9 to 83.5) | 1123 (846 to 1431) | 1682.3 (1121.5 to 2321.4) | 465.5 (316.2 to 650.4) | 70.3 (44.5 to 104.8) | 395 (236.1 to 535.2) | 616 (197.8 to 1190.2) | 425.4 (245.7 to 688.9) | 121.3 (88 to 181.3) | 1288.5 (1029.7 to 1712.9) | 582.6 (454 to 798.5) | 181.8 (151.7 to 223.5) | 242.2 (166.3 to 346.4) | 543 (351.8 to 787.1) | 39.1 (26.6 to 56) | 559.4 (430.8 to 692.2) | 348.9 (293.4 to 417.6) |
|  |  | Khuzestan | 276.8 (229.1 to 331.7) | 155.4 (103.3 to 236.1) | 74.9 (62.2 to 91.6) | 125.8 (99.6 to 164.8) | 153.7 (109.6 to 209.5) | 52.6 (28.6 to 99.7) | 1111.5 (902.4 to 1310.7) | 1743.7 (1195.1 to 2437.5) | 442.8 (290.7 to 617.1) | 72.7 (45.5 to 114.2) | 361.7 (254 to 453.6) | 594 (207.2 to 1178.6) | 485 (277.3 to 782.3) | 141.8 (98.6 to 255.1) | 1459.6 (1220.3 to 1825.9) | 608.5 (485.4 to 803.1) | 288.5 (231.4 to 355.4) | 182.8 (125.2 to 267.2) | 543.7 (347.2 to 792.3) | 29.6 (19.8 to 41.1) | 478.9 (362.5 to 620.9) | 490.2 (428.1 to 570.6) |
|  |  | Kohgiluyeh and Boyer-Ahmad | 352.2 (288.8 to 424.5) | 171.9 (113.3 to 256.3) | 65.1 (54 to 78.1) | 114.7 (92.9 to 146.7) | 156.4 (109.6 to 212.5) | 66 (28.7 to 136.2) | 1074.9 (877.4 to 1272.4) | 1667.9 (1146.2 to 2292.4) | 420 (283.5 to 589.7) | 76.5 (47.5 to 118.6) | 438.1 (284.9 to 551.2) | 567.8 (171.2 to 1141.4) | 469.1 (272.1 to 774.5) | 136.8 (103 to 179.1) | 1317.2 (1089.1 to 1636.2) | 490.7 (377 to 699.8) | 385 (254.5 to 499.9) | 181.1 (121 to 261.6) | 538 (344.9 to 786.2) | 26.3 (17.2 to 37.3) | 725.4 (589.1 to 858.4) | 518.5 (444.5 to 610.4) |
|  |  | Kurdistan | 210.9 (172.1 to 253.6) | 164.7 (105.8 to 248.6) | 50.3 (41.2 to 60.7) | 112.8 (85.6 to 148.5) | 154 (108.8 to 208.9) | 44.8 (21.3 to 88.8) | 1378 (1160 to 1610.9) | 1748.4 (1157.3 to 2433.9) | 463.4 (303.5 to 655.4) | 68.7 (42.4 to 110.9) | 323.5 (197.4 to 424.6) | 590.3 (183.3 to 1239.1) | 434.5 (240.1 to 740.6) | 101.7 (76.1 to 141.6) | 1231.4 (982.4 to 1574.4) | 461.6 (354.1 to 654.8) | 201.1 (162.9 to 244.4) | 181.1 (122.5 to 258.1) | 538.8 (347.5 to 776.9) | 20.2 (13 to 29) | 486.5 (409.3 to 566.9) | 296.9 (249.4 to 349.2) |
|  |  | Lorestan | 156 (128.4 to 188.1) | 153.4 (94.3 to 240.2) | 37.3 (29.6 to 45.6) | 79.7 (61.4 to 104.7) | 113.9 (76.2 to 162.6) | 49.1 (25.6 to 87.6) | 623.2 (497.4 to 745.3) | 1617.4 (1119.2 to 2250.5) | 452.5 (298.9 to 637.7) | 65.7 (40.9 to 104.1) | 225.2 (152.9 to 289.5) | 566.9 (164.6 to 1179) | 445.3 (259.8 to 748.6) | 86.4 (61.1 to 130.6) | 796.7 (619.6 to 1050.4) | 493.8 (378.4 to 718.2) | 227.8 (140 to 282.4) | 187.8 (125.3 to 268) | 539.1 (345.6 to 792) | 27.6 (18.7 to 39.2) | 273.4 (223.8 to 325.1) | 186.1 (153.6 to 230) |
|  |  | Markazi | 215.6 (171.7 to 260.8) | 173.3 (115.5 to 265.1) | 67.1 (54 to 79.4) | 117.8 (93.5 to 151.3) | 125 (84.9 to 171.9) | 38 (17.6 to 74.9) | 990.5 (827.2 to 1164.3) | 1887.5 (1318.3 to 2541.7) | 493.9 (328.6 to 679.2) | 64.3 (38.7 to 101.5) | 398.6 (249.7 to 506.9) | 631.7 (204 to 1268.8) | 425.9 (231.5 to 736.3) | 98.9 (77 to 128.5) | 1094.9 (867.7 to 1387.3) | 581.9 (454.8 to 799.3) | 146.9 (119.7 to 194.2) | 193.4 (132.2 to 275.6) | 549.6 (356 to 788.2) | 32.8 (21.4 to 47.6) | 596.5 (506.3 to 696.2) | 313.9 (262.1 to 370.8) |
|  |  | Mazandaran | 236.4 (190.1 to 291) | 161 (103.4 to 249.5) | 61.2 (50 to 73.4) | 88 (69.4 to 111.7) | 119.4 (79.6 to 169.8) | 35.4 (16.2 to 70.4) | 533 (421 to 657.9) | 2027.1 (1364.1 to 2849.5) | 765 (514.2 to 1056.9) | 57.4 (36.1 to 90.4) | 312.5 (216.1 to 387.5) | 635.1 (190 to 1315.9) | 377.4 (214.5 to 636.1) | 78.6 (57.7 to 113.4) | 949.5 (757.1 to 1180.3) | 459.1 (338.1 to 695.3) | 185.8 (153.8 to 227.7) | 194.6 (130.4 to 278.5) | 555.8 (356 to 812.8) | 24.3 (16 to 34.2) | 495.3 (398.4 to 577.2) | 280.3 (236.8 to 340.4) |
|  |  | North Khorasan | 290.8 (237.5 to 344.8) | 190.8 (132 to 287.8) | 75.6 (63.1 to 90.7) | 143.3 (110 to 184.5) | 167.5 (122.9 to 223.4) | 49.6 (25.1 to 97) | 1039.1 (873.4 to 1211.2) | 1769.1 (1189.9 to 2444.5) | 455.7 (298.2 to 641.1) | 77.8 (46.9 to 128.3) | 409.8 (262.4 to 518.6) | 645.7 (260.5 to 1246.2) | 444.8 (253.6 to 772.2) | 122.4 (93.4 to 174.8) | 1292 (1052.8 to 1613) | 668 (538.1 to 885.9) | 265 (201.1 to 334) | 188.6 (127 to 266.6) | 539.9 (352.1 to 791.7) | 32 (21.5 to 42.7) | 654 (540.6 to 793.3) | 387.8 (324.3 to 473) |
|  |  | Qazvin | 177.7 (143.1 to 220.4) | 158.9 (101.3 to 238.2) | 46 (36.6 to 56.1) | 132.9 (101.1 to 172.9) | 124.3 (84.4 to 176.1) | 39 (17.3 to 77.9) | 794.9 (633 to 949.2) | 1759.7 (1188.9 to 2449.2) | 475.3 (317.5 to 660.5) | 62 (34.6 to 96.4) | 302.6 (187.8 to 381.2) | 602.8 (186.4 to 1201.4) | 416.8 (227.6 to 697.8) | 82.1 (62.5 to 111.6) | 988 (789 to 1259.4) | 528.9 (413.6 to 746.6) | 133.5 (110.5 to 162.1) | 191 (131.5 to 274.2) | 547.4 (353.2 to 798.6) | 32.5 (22.1 to 44.8) | 378.5 (308.6 to 454) | 261.2 (220.7 to 314) |
|  |  | Qom | 137.9 (109.6 to 168.4) | 162.3 (103.8 to 247.2) | 54.1 (43.9 to 65.7) | 105.6 (79.9 to 138.3) | 118.8 (79.4 to 166.7) | 51.9 (30.5 to 94.1) | 708.9 (569.8 to 854) | 1835.2 (1257.7 to 2504.2) | 463.2 (306.4 to 651.9) | 65.6 (38.9 to 104.1) | 336.1 (194.3 to 427) | 596.6 (176.1 to 1180) | 409.6 (223.8 to 696.6) | 80.7 (57.8 to 117.6) | 975.5 (788.8 to 1235.4) | 520.4 (403.7 to 716.3) | 100.8 (84.2 to 143.8) | 191 (129.1 to 271.2) | 551.8 (357.5 to 808.8) | 21.5 (13.6 to 30.2) | 216.8 (170.2 to 324.1) | 266.8 (224.1 to 322.7) |
|  |  | Semnan | 178.9 (142.2 to 223) | 162.1 (104.7 to 244.6) | 57.7 (47 to 70.5) | 95.8 (73.4 to 126.1) | 121 (81.8 to 166.6) | 36.4 (14.6 to 79.9) | 893.7 (683.6 to 1136.3) | 1751 (1211.7 to 2373.3) | 490.2 (322.5 to 687) | 63 (38 to 96.2) | 271.5 (183 to 350.2) | 638.4 (207.1 to 1281) | 411.7 (235.8 to 675.7) | 74.6 (53.6 to 102.5) | 1094.2 (844.9 to 1417.2) | 654.1 (531.7 to 866.2) | 120.7 (100.8 to 153.8) | 180.3 (123.2 to 257.1) | 553.5 (361.6 to 792.3) | 29.2 (18.7 to 41.1) | 405.4 (319.3 to 494.1) | 259.1 (212.2 to 321) |
|  |  | Sistan and Baluchistan | 360.9 (298.2 to 428) | 253.2 (177 to 361.5) | 120.3 (73.3 to 148.9) | 221 (168.1 to 285) | 268.2 (193.9 to 358.9) | 86.3 (46.5 to 155.5) | 1878 (1576.6 to 2169.7) | 1688 (1139.7 to 2328.3) | 420.2 (278.2 to 576.7) | 109.7 (69.1 to 154.8) | 328.4 (245.3 to 430.2) | 598.5 (230.4 to 1183.1) | 557.9 (326.3 to 869) | 307.2 (176 to 594.9) | 1598.8 (1293.6 to 2051.2) | 699.7 (571.1 to 888.2) | 189.3 (150 to 271.4) | 189.6 (128.9 to 271.8) | 523.2 (337.3 to 769.4) | 48.8 (32.6 to 67.2) | 754.2 (640.6 to 886) | 505.7 (426.7 to 612.5) |
|  |  | South Khorasan | 201.7 (162.5 to 248.2) | 190 (127.8 to 277.5) | 67 (53.7 to 82.9) | 133.3 (98.1 to 178.9) | 143.8 (99.2 to 193.3) | 40.8 (17 to 83.3) | 1177.9 (970.2 to 1381.1) | 1784.8 (1185.5 to 2428.4) | 458 (300.4 to 649.7) | 72.1 (45 to 112.6) | 309.5 (186.5 to 413.1) | 588.9 (177.3 to 1193) | 440.2 (256.1 to 724.6) | 134.3 (90.4 to 237.9) | 1097.5 (852.3 to 1459.3) | 571.9 (451.6 to 751.7) | 137.2 (113.1 to 166.6) | 187.9 (126.9 to 269.3) | 539.8 (348.3 to 793.8) | 31.9 (21.7 to 44.5) | 486.1 (396.7 to 567.3) | 319.4 (274.1 to 379.2) |
|  |  | Tehran | 196.2 (161.5 to 244.2) | 165.6 (107.2 to 251.4) | 74.4 (58.9 to 90.8) | 103.6 (81.4 to 128.3) | 116.2 (78.8 to 161.6) | 50.4 (28.1 to 96.2) | 597.1 (404.1 to 818.1) | 1999 (1351.7 to 2744.4) | 526.4 (362.7 to 737.4) | 52.6 (32.9 to 83.5) | 402.9 (280.2 to 486.5) | 649.7 (192.9 to 1277.3) | 389.3 (237.4 to 651.4) | 87.7 (62.2 to 132.4) | 989.4 (782.7 to 1262.6) | 505.2 (392.6 to 701.2) | 134 (108.7 to 195) | 185.9 (125.1 to 262.6) | 559.2 (362.3 to 811.7) | 87.1 (58.5 to 125.9) | 120.2 (83.9 to 268) | 337.7 (270.5 to 411.8) |
|  |  | West Azarbayejan | 291.3 (235.3 to 346.3) | 195.4 (131.6 to 288.2) | 70.2 (57.4 to 84.6) | 129.1 (101.3 to 165.5) | 165.9 (117.9 to 220.8) | 45 (21.5 to 86.8) | 1179.4 (989.4 to 1371) | 1995.5 (1377.1 to 2649.4) | 471.4 (316.8 to 654.9) | 67.9 (41 to 107.2) | 452 (279.3 to 591.1) | 632.1 (209.3 to 1215.1) | 426.2 (227.3 to 722.5) | 138.2 (94.1 to 245.6) | 1397.4 (1165.1 to 1734.5) | 536.8 (416.9 to 763.2) | 386.7 (295.6 to 485.2) | 193.8 (130.2 to 275.8) | 539 (345.3 to 788.5) | 21.9 (14.2 to 32) | 530.7 (429.8 to 657.2) | 380.2 (323.4 to 463.8) |
|  |  | Yazd | 181.2 (144.2 to 220.6) | 170.5 (113.7 to 249.8) | 64.3 (52.6 to 78.3) | 97.4 (77.9 to 124.6) | 136.3 (94 to 186.7) | 45.5 (24 to 85.4) | 1127.6 (920.8 to 1352.6) | 1729.2 (1155.8 to 2388.6) | 476.7 (319.2 to 660.6) | 64 (38.8 to 98) | 448.8 (257.1 to 557.1) | 600.4 (183.9 to 1191.9) | 438.6 (245.2 to 759.9) | 95.2 (67.9 to 138.5) | 1344.5 (1092.4 to 1644.1) | 718 (588.9 to 909.7) | 120.1 (97.1 to 152.6) | 178.4 (122.4 to 257.3) | 552.8 (363.1 to 809.6) | 26.2 (16.9 to 36.9) | 461 (368.3 to 568.4) | 313.6 (266.9 to 376.4) |
|  |  | Zanjan | 199.8 (160.7 to 244.9) | 165.2 (106.1 to 247.2) | 45.5 (37 to 57.4) | 103.5 (78.4 to 135.4) | 139.6 (97.2 to 192) | 44 (21.2 to 84.9) | 1153.3 (923.3 to 1383.2) | 1794.3 (1236.3 to 2439.4) | 456.8 (296.7 to 648.4) | 65.2 (40.6 to 101.2) | 271.3 (174.2 to 353.1) | 583.6 (175.4 to 1189.9) | 411.9 (225.7 to 697.5) | 99.6 (74.7 to 136.7) | 1108.3 (875.8 to 1441.3) | 553.3 (432.1 to 760.7) | 140.5 (116.7 to 167) | 189.2 (128.2 to 270.1) | 541.1 (351.8 to 796.9) | 23.6 (14.7 to 33.9) | 461.6 (387.8 to 536.8) | 275.9 (230 to 324.2) |
| DALYs (Disability-Adjusted Life Years) | Males | Province | Causes |  |  |  |  |  |  |  |  |  |  |  |  |  |  |  |  |  |  |  |  |  |
|  |  |  | Cardiovascular diseases | Chronic respiratory diseases | Diabetes and kidney diseases | Digestive diseases | Enteric infections | HIV/AIDS and sexually transmitted infections | Maternal and neonatal disorders | Mental disorders | Musculoskeletal disorders | Neglected tropical diseases and malaria | Neoplasms | Neurological disorders | Nutritional deficiencies | Other infectious diseases | Other non-communicable diseases | Respiratory infections and tuberculosis | Self-harm and interpersonal violence | Sense organ diseases | Skin and subcutaneous diseases | Substance use disorders | Transport injuries | Unintentional injuries |
|  |  | Alborz | 266.9 (213.2 to 334.2) | 198 (122.7 to 305.4) | 52 (41.9 to 64.8) | 96.8 (76.3 to 127.2) | 120.1 (84.8 to 166.8) | 50.2 (24.6 to 93.4) | 690.8 (548.6 to 822.7) | 1707.2 (1223.2 to 2330.8) | 243.8 (162.8 to 351.4) | 38.2 (22 to 67.2) | 334.4 (243.2 to 432) | 519.1 (171.9 to 1008.4) | 168.3 (81.3 to 327.8) | 76.1 (53.4 to 120) | 870.1 (706.6 to 1084.6) | 735 (606.8 to 940) | 473.9 (356.5 to 612.9) | 195.4 (131.3 to 279.5) | 496.3 (312.5 to 725.9) | 122.1 (84 to 173.7) | 752.6 (591.2 to 928.9) | 637.6 (532.9 to 746) |
|  |  | Ardebil | 376.8 (294 to 473.8) | 197.5 (124.2 to 308.5) | 63.1 (48 to 79.9) | 96.3 (72.2 to 126.8) | 136.6 (93.7 to 189) | 45.8 (18.1 to 97.5) | 1312.7 (1070 to 1544.9) | 1617.3 (1121.9 to 2135.6) | 227.3 (153.1 to 319.2) | 63.1 (28 to 217) | 437.3 (265.5 to 601.4) | 529.1 (222 to 985.6) | 234.8 (115.3 to 447.5) | 94.7 (61.2 to 172.8) | 1457.9 (1197.5 to 1816.2) | 541.7 (426.8 to 732.4) | 368.4 (286.8 to 461.1) | 188.3 (124.8 to 268.8) | 478.5 (308 to 695.6) | 51.1 (34 to 70.7) | 888.9 (706.4 to 1075.3) | 591 (467.7 to 785.5) |
|  |  | Bushehr | 212.8 (169.2 to 258.9) | 186.9 (113 to 294.8) | 47.6 (37.1 to 60.2) | 96.9 (74.8 to 129.1) | 126.6 (89.8 to 180.1) | 52.7 (27.3 to 101.1) | 1242.9 (1042.3 to 1471) | 1677.8 (1198.6 to 2294.6) | 233.7 (147.5 to 332.6) | 45 (28.3 to 71.2) | 295.6 (194.8 to 399.3) | 505.6 (188.5 to 993.8) | 220 (120.5 to 385.9) | 67 (50.9 to 89.8) | 1128.6 (939.1 to 1448) | 531.9 (415.2 to 724.3) | 242.6 (191.2 to 303.1) | 191.4 (129.7 to 278.2) | 495.2 (325.4 to 716.4) | 49.5 (33.2 to 68.7) | 1057.2 (869.8 to 1253.4) | 498.8 (414.5 to 625.4) |
|  |  | Chahar Mahaal and Bakhtiari | 218.3 (170.3 to 274.6) | 194.1 (118.7 to 308.2) | 35.9 (28.1 to 45.1) | 70.1 (52.2 to 93.9) | 122.7 (77.6 to 174.2) | 47.2 (18.2 to 97.5) | 814.1 (640.4 to 989.9) | 1485.3 (1056.7 to 2022.9) | 213.2 (138.2 to 303.2) | 41.8 (25.3 to 67.3) | 284.5 (154.7 to 408.3) | 490.4 (179 to 941.4) | 178.2 (87.5 to 351.9) | 54.2 (36.7 to 82.9) | 917.2 (737.4 to 1193) | 434.7 (325.1 to 626) | 240.4 (182 to 295.8) | 186.3 (122.7 to 265) | 476.8 (305.3 to 700.8) | 38 (24.2 to 53.6) | 814 (652.8 to 974) | 499.5 (413.8 to 625.9) |
|  |  | East Azarbayejan | 302.8 (244.1 to 364.4) | 221.4 (149.5 to 326) | 70.4 (54.8 to 88) | 111.6 (89.1 to 149.5) | 122.9 (86.7 to 167.4) | 42.9 (20 to 86) | 828.4 (655 to 1013.2) | 1562.9 (1084.7 to 2087.7) | 209.8 (133.1 to 318.3) | 51.4 (27.5 to 100.2) | 440.7 (286.8 to 605.3) | 594.1 (255.7 to 1092.1) | 231.5 (111.4 to 425.4) | 92.7 (63.6 to 150.9) | 1123.6 (905.3 to 1415.9) | 571.6 (449.3 to 800.8) | 338.3 (253.8 to 435.5) | 194 (130.8 to 274.8) | 490.1 (314.1 to 709.8) | 89 (62 to 128.3) | 1261.2 (1036.5 to 1510.3) | 607.4 (510.2 to 747.8) |
|  |  | Fars | 356.3 (290.6 to 439.8) | 208.8 (135.3 to 316) | 74.3 (56.6 to 93.3) | 146.3 (109.1 to 199.7) | 131.6 (94.6 to 178.9) | 69.1 (43.9 to 115.2) | 1284.8 (1018 to 1590.3) | 1802.5 (1266.9 to 2462) | 231.9 (151.3 to 326.4) | 51.7 (26.9 to 126.5) | 484.9 (317.9 to 688.5) | 553.3 (228.9 to 1040) | 231.5 (119.8 to 436.5) | 104.8 (77.5 to 150.3) | 1454.2 (1186.6 to 1837.4) | 631.2 (508.6 to 843.4) | 572.6 (441.2 to 741.2) | 190.4 (126.9 to 273.2) | 490.5 (321.7 to 718.7) | 116.2 (79 to 166.5) | 1976.1 (1647.8 to 2321.1) | 759.3 (640.5 to 906) |
|  |  | Gilan | 379 (302.4 to 473) | 211.8 (133.7 to 318.7) | 56.4 (44.9 to 71.4) | 85.4 (65.5 to 111) | 116.6 (79 to 164.4) | 34.8 (13.5 to 70.9) | 463.5 (350.5 to 575) | 1625.1 (1143.4 to 2194.2) | 252.3 (161.7 to 357.8) | 37.2 (21.8 to 61.4) | 339.7 (242.9 to 451) | 535.5 (181.3 to 1033.2) | 194.5 (105.1 to 349.2) | 64 (45.4 to 96.1) | 733 (584.9 to 912.3) | 528.5 (413 to 733.6) | 387.1 (305.2 to 495) | 189 (127.8 to 274.6) | 497.3 (313.5 to 729.5) | 77.4 (53.7 to 107.3) | 1433.4 (1167.3 to 1719.8) | 716.2 (591.8 to 880.2) |
|  |  | Golestan | 417.8 (343.4 to 503.7) | 223.5 (150.9 to 336.4) | 79.7 (62.6 to 97.5) | 156.9 (111.7 to 214.6) | 146.3 (108.3 to 195.6) | 52.2 (23.6 to 103.5) | 1292.5 (1080.6 to 1503.4) | 1501 (1069.8 to 2028.8) | 219 (143.5 to 310.5) | 55.8 (31.4 to 87.7) | 443.3 (329.2 to 586.8) | 560.4 (230.3 to 1014.1) | 299.1 (144.1 to 548.8) | 149.8 (104.3 to 243.6) | 1447.3 (1215.1 to 1810.9) | 644.7 (527.6 to 835.9) | 400.8 (314.4 to 516.4) | 187.8 (126 to 269.8) | 481 (314.8 to 699.1) | 53.5 (36.6 to 72.4) | 1839.3 (1549.2 to 2114.4) | 741.2 (616.3 to 914.3) |
|  |  | Hamadan | 407.6 (336.2 to 496) | 217.2 (142.6 to 321.1) | 69.4 (54.3 to 88.2) | 143.1 (110.3 to 200.2) | 150.4 (110.4 to 208.2) | 49.6 (22.9 to 98.6) | 1377.7 (1182.4 to 1597.4) | 1506.2 (1049 to 2033.3) | 221.5 (144.8 to 310.1) | 51.2 (29.8 to 85.2) | 506.7 (348.9 to 687.5) | 545.5 (229.9 to 1014.5) | 263.2 (133.8 to 502.9) | 132.3 (97.6 to 185.2) | 1407.1 (1180.1 to 1709) | 579 (456.2 to 788.3) | 475.6 (352.1 to 611.7) | 187.5 (125 to 269.2) | 481.8 (315.9 to 717) | 148.2 (98.9 to 216.1) | 1641.3 (1373.9 to 1930.9) | 747 (630.3 to 915) |
|  |  | Hormozgan | 394.5 (322.5 to 474.8) | 201.9 (130 to 313.3) | 87.9 (56.2 to 111.6) | 143.3 (104.4 to 190.7) | 151.8 (105.6 to 229.8) | 71.5 (42.6 to 124.6) | 1451.6 (1220.6 to 1697) | 1573.5 (1103.7 to 2101.1) | 215.8 (146 to 297.7) | 57.4 (34 to 88) | 341 (261.1 to 434.3) | 523 (221.3 to 983.4) | 323.7 (180.9 to 542.6) | 108.6 (82.4 to 157.5) | 1359.7 (1126.6 to 1682.3) | 638.3 (521.3 to 838) | 413.3 (315.7 to 522.3) | 185.6 (124.1 to 265.7) | 480.2 (311.4 to 691) | 105.5 (71 to 147) | 1771.5 (1441.1 to 2099.6) | 753.9 (644.7 to 904.8) |
|  |  | Ilam | 296.6 (236.1 to 370.4) | 192.2 (116.8 to 301) | 77.9 (50 to 101.5) | 93.4 (71.1 to 126.2) | 130.4 (92.8 to 175) | 48.4 (19.2 to 105.2) | 1387.9 (1033.1 to 1764.3) | 1570.1 (1095.2 to 2124) | 219.4 (143.7 to 310.1) | 48.9 (28.4 to 80.4) | 354.4 (227.2 to 510.4) | 489.8 (186.9 to 910.7) | 230.2 (115.3 to 455.3) | 79.5 (57.4 to 111.8) | 1289.7 (973.3 to 1687.9) | 572 (451.9 to 767.8) | 648.1 (485.4 to 816) | 184 (124.6 to 264.8) | 480 (308.5 to 691.9) | 40.9 (27.4 to 57.4) | 1084 (850.7 to 1329) | 601.1 (488.1 to 748.5) |
|  |  | Isfahan | 292.3 (232.8 to 352.3) | 207.8 (136.1 to 315.9) | 65.6 (51.3 to 80.3) | 104.4 (81.4 to 137.6) | 123.3 (85.3 to 172.5) | 37.8 (16.2 to 76.9) | 978.1 (749.3 to 1228.3) | 1582.9 (1125.9 to 2116.4) | 239.5 (158.3 to 336.3) | 40.8 (24.8 to 65.3) | 421 (268.7 to 575.6) | 547.1 (212 to 1049.8) | 220 (115.6 to 404.9) | 81.2 (57.6 to 119.2) | 1168.2 (930.4 to 1480.7) | 608.6 (481 to 816.3) | 301.1 (239.2 to 385.1) | 192.5 (130.1 to 277.4) | 494.4 (316.7 to 729.7) | 81.1 (55 to 113.7) | 1143.5 (943.8 to 1408.3) | 698.9 (587.7 to 839.1) |
|  |  | Kerman | 330.7 (262.8 to 403.9) | 242.8 (168.5 to 354.7) | 71.3 (54 to 88.5) | 134.8 (102.2 to 179.8) | 156.1 (112.7 to 210.1) | 54.8 (28.6 to 105.2) | 1711.6 (1466.3 to 2013.2) | 1750.4 (1246 to 2332.9) | 224.7 (149 to 317.6) | 55.2 (33.3 to 88.3) | 391.2 (272.6 to 546) | 552 (206.4 to 1070.8) | 303.8 (158.9 to 542.9) | 134.9 (83.2 to 282) | 1410.3 (1172.3 to 1731.4) | 663.7 (533.6 to 862) | 391.2 (300 to 512.3) | 187.1 (125.2 to 268.4) | 482.7 (310.2 to 700.7) | 70.5 (46.9 to 101.2) | 1678.3 (1425.6 to 1949.1) | 653.6 (559.8 to 805.5) |
|  |  | Kermanshah | 328.3 (264.6 to 395) | 211.8 (140.6 to 318.1) | 67.8 (52.3 to 84.1) | 128.8 (99.1 to 175.4) | 128.2 (92.1 to 171.3) | 81 (55.1 to 127) | 1501.1 (1269.3 to 1768.1) | 1642.4 (1157.9 to 2222.8) | 224 (148 to 317.8) | 51.1 (30.7 to 81.5) | 445.3 (280.5 to 628.3) | 495.7 (169.3 to 947.8) | 255.4 (127 to 478) | 125.5 (78.9 to 240.1) | 1325.6 (1087.6 to 1671.5) | 638.9 (511.5 to 876.8) | 542.1 (424.9 to 676) | 188.2 (124 to 270.6) | 483.2 (314.1 to 706.4) | 128.8 (85.4 to 188) | 1339.9 (1105.7 to 1627.3) | 630.7 (526.3 to 767.3) |
|  |  | Khorasan-e-Razavi | 280 (213.4 to 350.7) | 217.4 (148.4 to 332.7) | 73.7 (57 to 94.8) | 125.9 (94.8 to 178) | 131.2 (96.6 to 183.5) | 47.4 (20.7 to 92.9) | 1287.1 (986.8 to 1584.7) | 1494.4 (1048 to 2060.2) | 222.5 (147.3 to 316.1) | 55.4 (34 to 86.4) | 447 (276.8 to 656.9) | 528.3 (214.6 to 958.3) | 260.4 (135.8 to 489.8) | 107.8 (77.1 to 163.2) | 1322.2 (1043.1 to 1749.3) | 663.3 (532.8 to 881.5) | 324.1 (257.7 to 409.1) | 231.6 (158.1 to 331.4) | 482 (311.5 to 711.2) | 93.4 (62.5 to 130.9) | 1332.8 (1060.4 to 1602.2) | 642 (522.4 to 755.8) |
|  |  | Khuzestan | 324.1 (265.1 to 401.7) | 192.2 (124.3 to 295.1) | 73.1 (59.4 to 89.9) | 117.9 (90.7 to 160.7) | 132.4 (96.6 to 182) | 57.6 (30 to 110.1) | 1340.2 (1098.3 to 1576.5) | 1574.1 (1105.3 to 2144) | 212.3 (141.4 to 303.5) | 48.2 (28 to 81.1) | 395.5 (286.8 to 517.2) | 508.5 (208.9 to 944.4) | 246.7 (120.6 to 479.9) | 117.6 (80.6 to 209.9) | 1463.4 (1212.7 to 1803.5) | 689.9 (556.1 to 874.2) | 382.8 (296.4 to 489) | 181.9 (123.1 to 257.7) | 483.5 (318.7 to 710.8) | 74.6 (52.7 to 108.5) | 1213.8 (1007.9 to 1446.3) | 886.7 (759.7 to 1040.2) |
|  |  | Kohgiluyeh and Boyer-Ahmad | 358.2 (282.5 to 438) | 203 (126.1 to 311.1) | 57.8 (46.6 to 70) | 110.2 (86.9 to 146.9) | 136.9 (98.6 to 188.8) | 70.9 (28.8 to 143.3) | 1240.6 (1005.4 to 1487.1) | 1530 (1079.8 to 2045.1) | 203.7 (132.9 to 293.9) | 50.6 (29.8 to 99.6) | 462.9 (285.1 to 654) | 499.5 (197.7 to 938.7) | 219.3 (113.4 to 432.3) | 115.4 (86.4 to 154.4) | 1337.5 (1104.1 to 1722.9) | 525.9 (415.4 to 709.8) | 435.8 (338 to 548.3) | 181.1 (120.5 to 257.8) | 471.8 (302.1 to 686.7) | 45.1 (30.8 to 63.8) | 1417.8 (1135 to 1682.3) | 791.9 (670.4 to 933.4) |
|  |  | Kurdistan | 312.7 (255.7 to 380.3) | 207.9 (134.6 to 322.6) | 61.6 (47.7 to 78) | 118.3 (92.1 to 164.4) | 133.8 (96.4 to 184.7) | 52.3 (24.6 to 104.4) | 1593.3 (1335.5 to 1863.7) | 1535.1 (1078.4 to 2089.9) | 219.5 (145.6 to 309.7) | 50.3 (29.8 to 82.5) | 440 (264.7 to 639.6) | 529.5 (223.8 to 983.9) | 240.2 (114.9 to 453.4) | 96.1 (70.6 to 131) | 1277.3 (1038.9 to 1609) | 540.1 (426.9 to 728.2) | 404.6 (335.4 to 496.3) | 181.7 (119.6 to 258) | 478.5 (316.8 to 696.7) | 53 (35.7 to 71.8) | 1414.5 (1177.2 to 1673.1) | 690.2 (579.9 to 845.3) |
|  |  | Lorestan | 210.4 (168 to 253.3) | 191 (116 to 288) | 42.6 (33.3 to 53.7) | 83.7 (65 to 114.7) | 119.9 (82.9 to 168.7) | 56.1 (29.3 to 101) | 748.3 (592.8 to 910.1) | 1479.6 (1048.2 to 1986.7) | 216.2 (140.7 to 303.3) | 41.9 (24.5 to 70.7) | 280.2 (171.6 to 389.9) | 468.4 (166.2 to 894.5) | 224.8 (120.7 to 411.9) | 77.5 (53.9 to 118.3) | 767.2 (610.2 to 986.2) | 592.8 (470.7 to 799.6) | 320 (243.7 to 405.1) | 185.2 (124.5 to 261.6) | 475.1 (304.1 to 699.9) | 76.9 (50.4 to 108) | 747.1 (604.2 to 901.5) | 429.2 (349.9 to 578.2) |
|  |  | Markazi | 265 (207.5 to 328.2) | 210.9 (137.8 to 316.2) | 65.3 (51.1 to 81.2) | 110.4 (85.2 to 150.7) | 121.8 (84.5 to 172.5) | 43.1 (20.2 to 83) | 1065.7 (885.6 to 1261.5) | 1678.5 (1212.5 to 2260.4) | 234.5 (152.8 to 332.6) | 43.9 (25.8 to 73.2) | 427.4 (251.7 to 616.5) | 540.4 (223.6 to 999) | 217.2 (108 to 427.8) | 83.2 (64.1 to 107.8) | 1029.6 (821.3 to 1308.8) | 658.8 (523.3 to 858.2) | 298 (233.7 to 367.2) | 192.7 (127.7 to 276.2) | 490.7 (316.1 to 718.8) | 85.7 (56 to 123.9) | 1286.2 (1052.6 to 1532.6) | 588.6 (493.7 to 708.7) |
|  |  | Mazandaran | 280.2 (219.1 to 347.5) | 199.9 (126.4 to 307.3) | 54.4 (43.9 to 67.5) | 83.5 (64.3 to 110) | 118.6 (79.7 to 171.7) | 37.8 (15.3 to 77.2) | 620.9 (490.8 to 747) | 1654.5 (1156.4 to 2284.5) | 246.9 (162.5 to 349.9) | 36.4 (21.2 to 58.2) | 344.7 (230.3 to 470.3) | 527 (194.6 to 1024.7) | 180.8 (91.5 to 333.6) | 63.1 (46.7 to 91.8) | 908.8 (735.3 to 1131.1) | 525.3 (406.5 to 742.1) | 305 (243.8 to 379.7) | 194.1 (131.8 to 281.1) | 498.2 (320.8 to 726.2) | 51.1 (33.8 to 71.1) | 1207.3 (988.6 to 1438.7) | 707.9 (591.2 to 836.8) |
|  |  | North Khorasan | 328.6 (260.5 to 400.5) | 215.2 (141.4 to 318.4) | 76.8 (58.7 to 97.6) | 130.6 (100.5 to 178.9) | 138.1 (100.6 to 194.9) | 48.9 (20.5 to 100.4) | 1181.1 (984.6 to 1380.7) | 1582.9 (1122.7 to 2130.9) | 218.4 (145.8 to 305.8) | 70.2 (36.1 to 171.7) | 428.3 (285.6 to 588.5) | 521.8 (231.3 to 955.6) | 293.9 (145.6 to 552) | 120.2 (87.7 to 167.2) | 1270.3 (1042.4 to 1593.4) | 749.6 (595.5 to 952.1) | 311.6 (252 to 388.7) | 185.5 (126.7 to 263.8) | 477.7 (304.9 to 693.9) | 61.2 (41.5 to 84.2) | 1509.8 (1259.2 to 1775.9) | 684.2 (576 to 806.5) |
|  |  | Qazvin | 228.9 (179.5 to 288.4) | 195.6 (124.2 to 303.2) | 48.7 (38.7 to 59.1) | 127 (85.8 to 175.8) | 120.9 (82.5 to 171.6) | 43.9 (18.7 to 89.5) | 942.5 (760.6 to 1121.4) | 1529.1 (1089.1 to 2076.9) | 229.7 (146.7 to 326.4) | 42.9 (24.1 to 71.2) | 331.5 (206.2 to 466.9) | 502.3 (193.2 to 988.3) | 213.3 (98.1 to 409.9) | 67.5 (50.7 to 91.7) | 972.8 (775.2 to 1242.5) | 630.5 (511.5 to 841.4) | 251.9 (204.6 to 314.8) | 190.5 (129.2 to 279.4) | 488.5 (321.3 to 709.5) | 96.5 (65.6 to 136.3) | 961.7 (748.9 to 1192.6) | 561.1 (462.4 to 684) |
|  |  | Qom | 186.4 (139.6 to 236.4) | 204.2 (130.4 to 315.2) | 60.4 (48.7 to 73.7) | 109.5 (82.8 to 145.2) | 120 (82 to 168.3) | 56.7 (31.4 to 100.3) | 787.9 (621.7 to 954.9) | 1621.5 (1146.8 to 2173.1) | 220.5 (141.6 to 316.8) | 46.2 (25.5 to 93.9) | 413.9 (247.3 to 573.1) | 500.6 (185.3 to 956.5) | 200 (98.7 to 387.3) | 71.5 (50.5 to 105.1) | 943.8 (755.5 to 1215.8) | 610.7 (486.8 to 811.3) | 283.9 (223.8 to 365.6) | 189.5 (126.5 to 270.3) | 498.4 (331.6 to 716.5) | 55.6 (38.2 to 75.3) | 562.1 (428.1 to 740.5) | 622.6 (464.6 to 759.4) |
|  |  | Semnan | 219.6 (169 to 276.1) | 196.9 (125.7 to 304.6) | 58.9 (45.4 to 73.2) | 98.5 (73.4 to 133.8) | 120 (80.3 to 168.8) | 39.4 (14.2 to 89.2) | 1060.1 (780.2 to 1355.8) | 1676.5 (1200.5 to 2242.7) | 239.7 (154.3 to 344.9) | 42.6 (24.8 to 70.8) | 317.7 (198.1 to 449.4) | 544.6 (221.3 to 1027.6) | 218.3 (112.2 to 400.6) | 62.4 (45.3 to 86.8) | 1047.7 (792.2 to 1354.8) | 761.2 (625.6 to 976) | 249.6 (201.6 to 312.3) | 182.8 (124.9 to 259.1) | 494.7 (321.8 to 718.1) | 68.3 (46.6 to 98.1) | 1003.7 (789.9 to 1247) | 478.2 (376.6 to 607) |
|  |  | Sistan and Baluchistan | 478.8 (382 to 587.4) | 278.4 (202.7 to 387.4) | 124.2 (59.5 to 159.8) | 226.9 (134.1 to 307.9) | 199.1 (141.4 to 314.9) | 83.2 (35.2 to 165.9) | 2316.9 (1975.2 to 2699.1) | 1520.7 (1063.4 to 2055.4) | 198.9 (130.5 to 279.9) | 102.2 (59.9 to 153.4) | 418.9 (309.9 to 545.3) | 525.6 (241.5 to 975.3) | 493.1 (273.1 to 857.9) | 304.9 (166.5 to 568) | 1776.5 (1460.8 to 2238.8) | 800.1 (665 to 987.1) | 482.3 (381.7 to 601.3) | 184.5 (126.6 to 261.6) | 459.4 (298 to 670.2) | 72.9 (49.7 to 101.4) | 1793.2 (1507.7 to 2081.8) | 884.5 (734.1 to 1110.9) |
|  |  | South Khorasan | 249.8 (198.6 to 309.7) | 235.9 (161 to 353.3) | 66.2 (51.6 to 85.1) | 124.1 (91.9 to 170) | 131.6 (94.1 to 177.5) | 43.7 (17.1 to 93.8) | 1351.5 (1114.5 to 1594.6) | 1632 (1114.1 to 2146.4) | 225.7 (145.2 to 316.6) | 50.1 (30.2 to 82.3) | 371.2 (215.1 to 541.8) | 509.9 (190.5 to 939.2) | 262.9 (137.4 to 513.6) | 111.8 (73.8 to 212.9) | 1151.3 (904.5 to 1497) | 655.2 (524.6 to 836.4) | 209.3 (173.1 to 255.5) | 189 (128.5 to 269.2) | 480.5 (309.5 to 704.4) | 66.1 (44.6 to 92.6) | 1151.3 (952.2 to 1348.9) | 532.8 (445.1 to 672.5) |
|  |  | Tehran | 198.6 (153.8 to 252.8) | 192 (118.5 to 304.5) | 53.8 (41.8 to 67.3) | 94.8 (73.9 to 122.9) | 108.9 (72.5 to 154) | 53.3 (28.6 to 107.7) | 752.8 (530.5 to 1016.1) | 1608.9 (1140.1 to 2185.4) | 287.9 (192.9 to 415.1) | 31.1 (18.6 to 50.7) | 333.2 (218.4 to 446.4) | 550 (208.1 to 1009.6) | 152.3 (80.1 to 273.5) | 74.1 (51.6 to 117.8) | 933.9 (725.7 to 1205.5) | 544.1 (422.3 to 749.2) | 202.1 (165.8 to 254.5) | 184.9 (123.3 to 264.7) | 498.2 (323.4 to 719.2) | 150.9 (98.1 to 216.4) | 162.2 (117.7 to 230.5) | 460.5 (357.5 to 579.5) |
|  |  | West Azarbayejan | 322.7 (259.5 to 392.9) | 221.8 (149.3 to 335.4) | 63.2 (49.7 to 77.5) | 118.1 (91.7 to 157.9) | 136.9 (101.1 to 185.5) | 47.9 (21.1 to 96.9) | 1336.1 (1091.1 to 1572.2) | 1594 (1136.9 to 2104.5) | 227.5 (149 to 321.9) | 50.5 (28.5 to 84.3) | 460 (288.1 to 646.2) | 544.8 (225.7 to 992.8) | 255.5 (124 to 505.2) | 115.3 (73.3 to 216.6) | 1400.8 (1148.8 to 1763.6) | 599.6 (481 to 812.7) | 467.3 (368.4 to 576.5) | 190.9 (126.3 to 270.6) | 478.9 (309.2 to 699.7) | 41.9 (29 to 57.3) | 1321.8 (1084.6 to 1586.7) | 653.3 (541.4 to 838.3) |
|  |  | Yazd | 214.3 (167.8 to 273) | 213.2 (139 to 324.6) | 66.4 (52.4 to 81.6) | 98.2 (77.6 to 129.6) | 127.4 (89.9 to 177.6) | 46.1 (21.4 to 91.5) | 1289.7 (1066.7 to 1510.8) | 1516.9 (1080.2 to 2074) | 226.8 (150 to 324.3) | 41.4 (24.6 to 66.9) | 431.2 (263.3 to 607.6) | 533.7 (196.8 to 995.1) | 235 (126.1 to 433) | 78.4 (55.8 to 118.7) | 1338.8 (1105.2 to 1655) | 829 (684.5 to 1028) | 218.1 (172.7 to 277.2) | 179.8 (121.9 to 256.7) | 495.4 (321.4 to 710.7) | 71.5 (49.1 to 101.5) | 1137 (901.2 to 1383.7) | 591.6 (484 to 709.8) |
|  |  | Zanjan | 263.5 (212.1 to 322.9) | 201.8 (127.3 to 316.2) | 50.7 (38.3 to 61.6) | 105.7 (81.2 to 143.8) | 143.4 (103.4 to 194.2) | 46.4 (19.4 to 92.1) | 1359.5 (1108.7 to 1621.7) | 1601.9 (1141.7 to 2198.2) | 221.7 (144.5 to 316.6) | 49.6 (30 to 82.4) | 334.9 (216.8 to 462.6) | 495.1 (184.3 to 918.7) | 223.9 (104.1 to 483) | 93.8 (70.3 to 130.8) | 1120.5 (889.1 to 1441.1) | 660.7 (529.5 to 875) | 213.6 (175.1 to 269.7) | 187.6 (126.5 to 268.9) | 478.2 (307.5 to 695.5) | 56.9 (38.3 to 79.4) | 1058.2 (880.5 to 1258.5) | 579.4 (486.8 to 696.8) |
| YLDs (Years Lived with Disability) | Both Sexes | Province | Causes |  |  |  |  |  |  |  |  |  |  |  |  |  |  |  |  |  |  |  |  |  |
|  |  |  | Cardiovascular diseases | Chronic respiratory diseases | Diabetes and kidney diseases | Digestive diseases | Enteric infections | HIV/AIDS and sexually transmitted infections | Maternal and neonatal disorders | Mental disorders | Musculoskeletal disorders | Neglected tropical diseases and malaria | Neoplasms | Neurological disorders | Nutritional deficiencies | Other infectious diseases | Other non-communicable diseases | Respiratory infections and tuberculosis | Self-harm and interpersonal violence | Sense organ diseases | Skin and subcutaneous diseases | Substance use disorders | Transport injuries | Unintentional injuries |
|  |  | Alborz | 88.8 (55.5 to 129.3) | 157.4 (92.1 to 251.9) | 17.8 (11.4 to 25.8) | 50.1 (35.1 to 68.5) | 74.9 (44.2 to 116.4) | 1.9 (1 to 3.2) | 213.9 (149.1 to 287.4) | 1824.3 (1287.4 to 2494.1) | 367.1 (247.5 to 519.1) | 43.8 (26 to 70.4) | 8.8 (5.7 to 13.4) | 535.5 (129 to 1099.7) | 265.7 (150.8 to 446.1) | 32.5 (19.9 to 50.8) | 380.6 (270.2 to 536.3) | 229.8 (126.1 to 443.9) | 30 (21.9 to 38.5) | 195.2 (132.3 to 277.7) | 524.1 (336.3 to 758.7) | 46 (27.9 to 64.9) | 33.4 (23.5 to 45.6) | 93.2 (64.2 to 133.3) |
|  |  | Ardebil | 116.5 (75.8 to 174.5) | 155.4 (90.9 to 251.1) | 17 (11.2 to 24.1) | 48.8 (33.9 to 67.3) | 69.8 (41.4 to 107.8) | 1.5 (0.7 to 2.8) | 219.6 (152.7 to 287.5) | 1713.4 (1171.1 to 2326.9) | 340.9 (229.2 to 477.1) | 52.2 (32.4 to 80.7) | 7.9 (4.5 to 12.7) | 511.2 (162.5 to 1026.8) | 314.6 (181.9 to 511) | 36.1 (22.9 to 54.3) | 397.3 (285.4 to 560.8) | 230.8 (128.9 to 444.5) | 28.7 (21.3 to 37) | 189.2 (127.7 to 267) | 508.4 (331 to 741.6) | 26.4 (16.4 to 38.2) | 31.3 (22.5 to 42.9) | 71.1 (49.1 to 100.4) |
|  |  | Bushehr | 70.1 (44.6 to 104.3) | 155.7 (89.5 to 248.3) | 17.2 (11.8 to 25) | 49.3 (33.6 to 69.3) | 71.6 (42 to 110.3) | 1.6 (0.8 to 2.8) | 253.4 (179.3 to 339) | 1768.2 (1243.8 to 2363.9) | 346.9 (228.8 to 485.1) | 53.1 (33.5 to 83.7) | 5.9 (3.7 to 9.1) | 521.1 (155.8 to 1076.5) | 326.9 (194.3 to 538) | 36.6 (23.2 to 55.3) | 399 (285.9 to 558.4) | 223.9 (122.7 to 412.5) | 28.7 (21.3 to 36.8) | 192 (130.9 to 275.7) | 517.4 (339.4 to 750.5) | 26 (16.4 to 37.1) | 36.7 (26.2 to 50.3) | 75.5 (52.5 to 108) |
|  |  | Chahar Mahaal and Bakhtiari | 76.7 (49.8 to 113.3) | 160.4 (93.2 to 264) | 14.4 (9.6 to 20.9) | 49 (33.8 to 69.5) | 73.5 (42 to 117) | 1.4 (0.7 to 2.4) | 216.6 (151.7 to 286.5) | 1564.8 (1090.1 to 2148.2) | 323.8 (215.1 to 452.1) | 53.3 (33 to 84.7) | 6 (3.4 to 9.5) | 495.8 (138.7 to 1018.5) | 304.8 (173.4 to 498.2) | 34.7 (22 to 53.1) | 384.5 (270.2 to 532.6) | 227.4 (125.9 to 412.3) | 25.9 (19.4 to 33.5) | 186.7 (127.1 to 266.2) | 508.1 (328.3 to 735.9) | 22.3 (13.9 to 32.3) | 32.7 (23.1 to 45) | 75.2 (52 to 109.2) |
|  |  | East Azarbayejan | 78.2 (51.1 to 112.7) | 163.5 (97.5 to 264.1) | 17.7 (11.9 to 25.3) | 49.1 (34.3 to 68.3) | 70.4 (41.9 to 111) | 1.6 (0.8 to 3) | 211.7 (148.6 to 283.2) | 1627.3 (1120 to 2193.3) | 294.9 (185.6 to 438.5) | 52.6 (31.9 to 81.8) | 8.2 (5 to 12.4) | 557.2 (164.6 to 1147.9) | 309.6 (178.1 to 508.7) | 35.6 (22.2 to 54.1) | 390.8 (280.8 to 560.7) | 230.9 (125.1 to 449) | 28.9 (21.6 to 37.1) | 194.2 (131.9 to 274.4) | 515.3 (332.9 to 746) | 37.1 (23.9 to 53.1) | 37.9 (26.9 to 52.5) | 81.7 (56.7 to 115.6) |
|  |  | Fars | 89.8 (59.9 to 128.6) | 158 (92.7 to 255.6) | 16.8 (11 to 24.6) | 50.5 (36.1 to 70) | 72.3 (42.3 to 114.2) | 2.2 (1.3 to 3.6) | 227.8 (160 to 299.3) | 1867.5 (1318.1 to 2566.5) | 347 (231.4 to 484.5) | 52.3 (32.2 to 81.2) | 8.8 (5.4 to 14) | 543.7 (143.3 to 1111.5) | 340.3 (206 to 553.7) | 36.5 (23.5 to 56.5) | 403.9 (285.5 to 572) | 230 (124.8 to 435.7) | 30.2 (22.5 to 38.6) | 191 (128.8 to 272.4) | 517.8 (339.2 to 747.1) | 42.2 (26.8 to 58.9) | 50.4 (36 to 69.3) | 89.3 (61.7 to 127.7) |
|  |  | Gilan | 112.3 (74.1 to 166.2) | 162.5 (94.1 to 256.6) | 18 (11.9 to 26.1) | 49.4 (34.2 to 68) | 78.3 (46.5 to 124.5) | 1.7 (0.9 to 3.2) | 204.6 (143.6 to 269.3) | 1756.7 (1191.2 to 2384.1) | 380.7 (248.7 to 538.6) | 45.6 (27.8 to 69.8) | 6.6 (4.4 to 9.8) | 550.8 (147.3 to 1148.9) | 281.2 (166.6 to 458.9) | 33 (21.1 to 49.9) | 387.7 (274.9 to 542.2) | 236 (129 to 461.5) | 32.2 (23.9 to 41.2) | 188.5 (127.9 to 273.5) | 525.5 (335.9 to 760.9) | 35.9 (23.1 to 51.5) | 36.2 (25.2 to 50.9) | 78.6 (54 to 112.9) |
|  |  | Golestan | 100.1 (63.5 to 145.2) | 156 (91.7 to 251.1) | 17.1 (11.4 to 24.7) | 50.1 (34.6 to 70.2) | 68.5 (40.6 to 109) | 1.6 (0.9 to 2.9) | 233.6 (162.9 to 309.1) | 1554.6 (1078.3 to 2118.3) | 328.5 (217.9 to 461.3) | 58.1 (35 to 89.7) | 5.8 (3.9 to 8.6) | 519.5 (139.8 to 1048.6) | 363.2 (207.2 to 586.9) | 40 (24.8 to 62.2) | 413.7 (295.4 to 579.4) | 232.1 (125.9 to 422.7) | 28.7 (21.3 to 36.9) | 188.5 (128.4 to 267.4) | 506.7 (330.8 to 732.9) | 25.6 (16.4 to 36.7) | 38.3 (27.2 to 52.9) | 81.4 (57.1 to 115.6) |
|  |  | Hamadan | 89.1 (57 to 130.4) | 156.6 (91.4 to 251) | 16 (10.8 to 23.2) | 50.5 (34.8 to 69.8) | 70.3 (41.9 to 108.7) | 1.5 (0.8 to 2.6) | 225.1 (162.4 to 298.9) | 1589.8 (1098.2 to 2163.9) | 331.4 (214.4 to 467.1) | 55.9 (34 to 85.2) | 7.9 (5 to 11.9) | 516.3 (171 to 1047.2) | 349 (196.9 to 549.1) | 38.8 (24.2 to 57.9) | 404.6 (287.4 to 559.9) | 235.5 (126.9 to 450.2) | 29.4 (21.7 to 37.6) | 188.2 (128.5 to 267.7) | 508.4 (333 to 746.8) | 48.4 (30.3 to 66.6) | 41.3 (29.4 to 56.3) | 84.2 (58 to 120.1) |
|  |  | Hormozgan | 89.8 (57.5 to 133.3) | 154.7 (90.4 to 253) | 17.3 (11.5 to 24.7) | 49.4 (34.2 to 69.7) | 72.1 (43 to 111.8) | 2.3 (1.4 to 3.7) | 229.3 (156.2 to 303.8) | 1655.9 (1146.1 to 2241) | 316.4 (213.1 to 437.9) | 58.1 (35.3 to 88.6) | 5.7 (3.5 to 8.9) | 500.1 (150.7 to 1029.9) | 390 (239.1 to 606.4) | 38.1 (24.1 to 58) | 401.4 (283.6 to 562.2) | 225.2 (124.7 to 426.7) | 26.1 (19.4 to 33.4) | 186.4 (125.6 to 266.7) | 505.5 (328.4 to 738.2) | 53.8 (33.6 to 80.2) | 35.8 (25.4 to 49.1) | 74.5 (52 to 105.9) |
|  |  | Ilam | 90.6 (59.5 to 135.9) | 157.3 (90.8 to 253.5) | 18.7 (12.8 to 26.6) | 47.9 (33.4 to 66.6) | 70.2 (40.5 to 110.1) | 1.4 (0.7 to 2.6) | 231.6 (161.3 to 307.8) | 1704.6 (1170.6 to 2380.8) | 325.7 (220.5 to 463.2) | 52.9 (31 to 82.8) | 6.6 (4 to 10.4) | 491.9 (146 to 995.9) | 327.1 (187.2 to 537) | 36.6 (23.6 to 55.1) | 399.7 (278.2 to 568.9) | 227.3 (127 to 429.9) | 31 (23.2 to 39.8) | 184.7 (124.8 to 264) | 509.6 (331.2 to 742.3) | 23.8 (14.8 to 34.1) | 36.2 (25.3 to 49.6) | 75.5 (52.2 to 108.3) |
|  |  | Isfahan | 86.8 (55.2 to 126.4) | 158.1 (94.6 to 251.8) | 16.9 (11.3 to 24.4) | 49.7 (34.1 to 68) | 75.5 (45.4 to 117.4) | 1.6 (0.8 to 2.8) | 227.8 (160.3 to 314.4) | 1764.8 (1227.8 to 2361.9) | 358.6 (240.6 to 504.8) | 49.5 (31.1 to 77.1) | 8.8 (5.3 to 13.5) | 540.7 (153.3 to 1115.4) | 327 (193.3 to 545.8) | 35.6 (23 to 54.7) | 403 (291.2 to 563.2) | 231.9 (123.4 to 446.8) | 29.8 (22 to 38) | 193.2 (132.3 to 274.1) | 520.5 (337.1 to 764.7) | 35 (22.5 to 49.7) | 39.1 (27.6 to 53.9) | 91.7 (62 to 130.2) |
|  |  | Kerman | 87.2 (55.5 to 130.3) | 162.2 (96.5 to 253.7) | 17.7 (12 to 26) | 51.2 (35.7 to 70.6) | 76.5 (47.3 to 116.4) | 1.6 (0.9 to 2.7) | 278.8 (193.7 to 368.2) | 1834.5 (1278.2 to 2499.1) | 333.1 (218 to 469.5) | 55.8 (33.5 to 87.4) | 7 (4.3 to 10.6) | 532.3 (157.7 to 1088.6) | 375.7 (221.4 to 611.6) | 39.4 (24.4 to 60.9) | 407.7 (293.9 to 571.5) | 230.4 (124.6 to 437.8) | 28 (20.7 to 35.7) | 188.2 (127.6 to 266.5) | 509.4 (326.9 to 741.7) | 31.4 (19.8 to 45.8) | 45.7 (32 to 61.8) | 112.2 (84.2 to 147) |
|  |  | Kermanshah | 89.8 (56.7 to 132.5) | 157 (92.9 to 247.3) | 17.4 (11.8 to 25.4) | 49.9 (35.1 to 70.1) | 69 (41.4 to 106) | 2.4 (1.4 to 3.8) | 233.7 (163.4 to 311) | 1710.3 (1192.8 to 2307.1) | 331.5 (219.5 to 467.1) | 55.4 (33.1 to 84.5) | 7.3 (4.4 to 11.3) | 502.7 (137.5 to 1031.6) | 339.9 (195 to 541.8) | 39 (24.3 to 59.5) | 408.8 (292.5 to 573.4) | 235.4 (126.4 to 458.5) | 31.7 (23.6 to 40.8) | 188.2 (126.8 to 267.5) | 508.1 (330.9 to 745.4) | 45.8 (29 to 63.4) | 35.8 (25.3 to 50) | 87.7 (62.9 to 121.9) |
|  |  | Khorasan-e-Razavi | 81.7 (53.2 to 119.8) | 157.5 (94.2 to 255.7) | 19 (12.7 to 27.3) | 50.1 (34.2 to 69.8) | 68.5 (40.9 to 105.7) | 1.6 (0.8 to 2.7) | 235.4 (162.1 to 310.6) | 1586.2 (1087.2 to 2174.5) | 333.6 (223.6 to 464.5) | 56.2 (35.4 to 83.5) | 7 (4.2 to 11.1) | 512.1 (144.4 to 993) | 332.1 (193.3 to 514) | 37.4 (23.8 to 55.1) | 409.2 (290.6 to 570.9) | 231.5 (129.5 to 447.2) | 27.6 (20.7 to 35.5) | 236.8 (166 to 337.6) | 508.9 (330.8 to 739.3) | 36.8 (23.9 to 51.4) | 37.7 (26.7 to 51.5) | 81.8 (56.4 to 114.6) |
|  |  | Khuzestan | 83.5 (54.4 to 118.4) | 143.1 (83.9 to 231.1) | 17.3 (11.5 to 25.1) | 48.3 (33.8 to 65.9) | 72.1 (43.1 to 112.6) | 1.6 (0.9 to 2.7) | 252.9 (178.6 to 336.7) | 1656.7 (1151.4 to 2265.8) | 317.7 (211.2 to 447.3) | 56.4 (34.3 to 87) | 7.4 (4.8 to 11.1) | 499.3 (165.1 to 1000.9) | 352.1 (202.9 to 570.3) | 39 (24.5 to 60) | 407.9 (288 to 574.4) | 230.3 (131.4 to 428.2) | 27.6 (20.5 to 35.7) | 182.3 (125.5 to 261.7) | 507.4 (328.9 to 744.1) | 31 (19.4 to 44.2) | 36.3 (26 to 49.9) | 79.9 (55.1 to 115.6) |
|  |  | Kohgiluyeh and Boyer-Ahmad | 83.6 (54.3 to 121.3) | 161 (94.2 to 265.3) | 14.8 (9.8 to 21.4) | 47.6 (33.8 to 64.8) | 75.6 (44 to 120.6) | 1.4 (0.7 to 2.5) | 241.2 (167.4 to 320.3) | 1596.9 (1112.6 to 2143.8) | 299.7 (198.1 to 428.1) | 53.5 (32.1 to 82.9) | 10.1 (6.2 to 15.9) | 475.9 (139.6 to 973.8) | 337.3 (199.7 to 555.5) | 37.3 (24.4 to 57) | 398 (286.5 to 551.4) | 225.9 (126.5 to 423.3) | 25.2 (18.5 to 32.4) | 181.1 (121.9 to 257.7) | 502.1 (321.8 to 732.2) | 24.3 (15.4 to 34.9) | 39.8 (28.1 to 55.1) | 91.8 (63 to 130.4) |
|  |  | Kurdistan | 89 (57.8 to 131.9) | 156.6 (90.2 to 255.9) | 16.3 (10.8 to 23.7) | 49.9 (34.1 to 69.6) | 69.3 (40.8 to 108.3) | 1.5 (0.8 to 2.6) | 223 (158 to 294.9) | 1639.4 (1120.4 to 2248.6) | 330.9 (219.9 to 465.8) | 55.5 (34.8 to 85.9) | 7.2 (4.2 to 11.6) | 501.3 (151.5 to 1040.2) | 330 (190.2 to 532.7) | 36.9 (23.2 to 54.6) | 399 (280.3 to 563.8) | 230.9 (131.1 to 421.5) | 29.5 (21.7 to 38.1) | 181.4 (122.3 to 256.5) | 505 (330.7 to 730.9) | 26.4 (16.5 to 37.8) | 36.6 (25.8 to 50.4) | 78.9 (54.8 to 112.9) |
|  |  | Lorestan | 71.2 (47.5 to 101.9) | 159.1 (92 to 251.3) | 15.2 (10.1 to 22) | 47.8 (33.5 to 66.6) | 70.4 (41.5 to 110.4) | 1.6 (0.9 to 2.9) | 221.3 (154 to 287.4) | 1546.4 (1072.1 to 2099.3) | 327.2 (219.1 to 461.3) | 50.9 (31.5 to 80.7) | 5.8 (3.4 to 8.9) | 489.1 (139.8 to 1026) | 313.4 (185.5 to 520.6) | 35.9 (23.4 to 54.7) | 382.8 (269.4 to 537.7) | 231.6 (132.2 to 436.6) | 26.9 (19.8 to 34.8) | 186.5 (125.4 to 266.3) | 505.5 (323.8 to 745.9) | 30.7 (18.7 to 44.1) | 27.7 (19.6 to 38.4) | 66.2 (45.4 to 94.1) |
|  |  | Markazi | 84.1 (55.6 to 124.3) | 159.6 (95.9 to 259.3) | 18.6 (12.7 to 26.9) | 50.1 (34.6 to 69.5) | 71.3 (42.6 to 111.8) | 1.6 (0.8 to 2.8) | 209 (146.1 to 273.5) | 1780.4 (1270.1 to 2393.7) | 354.2 (233.1 to 499.5) | 51.8 (31.9 to 79) | 8.2 (4.8 to 12.8) | 529.4 (162.1 to 1079.2) | 315.3 (178.1 to 531.7) | 35.8 (22.3 to 55.2) | 403.4 (284.7 to 574.4) | 234 (131.8 to 442.6) | 28.9 (21.3 to 37.5) | 193 (130.8 to 273.5) | 516.5 (336 to 745.8) | 37.3 (22.6 to 52.9) | 40.1 (28.4 to 55.4) | 84.4 (58.4 to 120.3) |
|  |  | Mazandaran | 90.7 (58.6 to 134.1) | 159.7 (95.5 to 254.3) | 17.5 (11.6 to 25.6) | 48.4 (33.5 to 66.8) | 77.1 (45.1 to 120.6) | 1.6 (0.8 to 2.9) | 214.7 (146.7 to 285.6) | 1838.2 (1257 to 2544.9) | 495.5 (332.3 to 689.1) | 44.1 (26.9 to 69) | 8 (5.1 to 12.1) | 544.1 (153.7 to 1123.2) | 272.1 (158.7 to 449.1) | 32.3 (20.5 to 50.2) | 386.9 (274.6 to 539.5) | 231.2 (126 to 455.3) | 30.4 (22.5 to 39.1) | 194.3 (129.8 to 277.5) | 524.4 (336.2 to 765.8) | 27.7 (17.2 to 40.5) | 41.4 (29.2 to 57.1) | 82.8 (56.7 to 117.6) |
|  |  | North Khorasan | 93 (60.4 to 135.1) | 158.1 (95.4 to 247) | 18.8 (12.3 to 27.5) | 49.3 (34 to 68.5) | 69 (42.1 to 105.8) | 1.5 (0.8 to 2.5) | 224.4 (155.3 to 296.2) | 1673.4 (1165.7 to 2282.6) | 327.2 (214.9 to 459) | 58.5 (35.3 to 89.2) | 7.3 (4.4 to 11.1) | 505.3 (167.2 to 1023) | 357.1 (204.6 to 588.8) | 38.8 (24 to 59.1) | 412 (293.5 to 578.8) | 232.9 (133.8 to 445.8) | 27.3 (20.3 to 34.9) | 187 (127.2 to 264.5) | 504.9 (327.6 to 738.8) | 29.8 (19 to 42.2) | 38.5 (27.2 to 52.4) | 86.4 (59.4 to 120.3) |
|  |  | Qazvin | 89.3 (58.5 to 130.9) | 156.1 (91.7 to 251.4) | 16.9 (11.4 to 24.2) | 51.2 (35.8 to 70.4) | 69.9 (40.5 to 110.8) | 1.5 (0.8 to 2.6) | 219.6 (152.5 to 285.9) | 1641.1 (1144 to 2258.5) | 344.8 (227.4 to 476.8) | 51 (30.8 to 77.4) | 6.4 (3.9 to 9.8) | 513.3 (156.1 to 1064.9) | 309.2 (178.8 to 519.1) | 34.8 (22.1 to 54.2) | 392.9 (275.6 to 556.4) | 228.9 (124.9 to 444) | 28.3 (20.8 to 36.6) | 190.8 (130.2 to 276.9) | 513.7 (334.6 to 744.7) | 36.7 (22.7 to 52.4) | 32.6 (23 to 44.5) | 80.3 (55.6 to 116.3) |
|  |  | Qom | 55.9 (36.5 to 82.1) | 157.5 (93.3 to 252.8) | 18.2 (12.1 to 25.9) | 49.4 (34.6 to 69.4) | 69.7 (40.2 to 108.7) | 1.6 (0.9 to 2.7) | 225.4 (155.8 to 297.7) | 1726.2 (1209.3 to 2328) | 335.5 (218.7 to 475.7) | 50.3 (30.6 to 80.3) | 8.5 (4.8 to 12.9) | 511.1 (145.9 to 1023.2) | 300.5 (172.2 to 501.3) | 34.4 (22.2 to 52.5) | 390.9 (275.9 to 555.7) | 225.8 (124.4 to 433.9) | 27.5 (20.2 to 35.3) | 190.2 (129.1 to 268.4) | 512.7 (332.6 to 747.2) | 27.9 (17.4 to 40.2) | 24.3 (17.2 to 33.3) | 85.1 (58 to 122.5) |
|  |  | Semnan | 86.1 (56.4 to 123.8) | 155.2 (90.6 to 249.1) | 19.1 (12.7 to 27) | 50 (35.4 to 70.1) | 68 (39.8 to 105.9) | 1.6 (0.7 to 2.9) | 229 (164.1 to 300.9) | 1712.7 (1209.3 to 2269.9) | 358.4 (235.6 to 497.2) | 50.4 (31.5 to 78) | 6.3 (3.9 to 9.6) | 537.3 (163.5 to 1091.9) | 307.6 (177.1 to 507.8) | 34.7 (22 to 52.9) | 396.4 (282.4 to 555.1) | 229.3 (127 to 439.5) | 30.2 (22.3 to 39.1) | 181.6 (124.5 to 259.2) | 520.5 (341.3 to 757.7) | 32.8 (19.9 to 46.9) | 35.6 (25.2 to 48.1) | 76.9 (53 to 109.4) |
|  |  | Sistan and Baluchistan | 76.8 (50.8 to 113) | 169.5 (104.6 to 269.6) | 17.9 (11.9 to 25.5) | 56.2 (39.2 to 77.8) | 69.9 (43.6 to 106.2) | 1.8 (1.1 to 2.9) | 261.7 (182.8 to 338.5) | 1602.5 (1110.5 to 2203.9) | 297.7 (193 to 414.1) | 75.8 (48.3 to 112.9) | 4.7 (3 to 6.9) | 483.3 (160 to 1001.5) | 496.3 (303.4 to 778.6) | 50.2 (31.6 to 74.7) | 441.4 (317.6 to 624.3) | 229.3 (127.7 to 406.4) | 30 (22.5 to 38.3) | 187 (128.6 to 266.6) | 488.4 (315.6 to 715.4) | 34.1 (21.9 to 48) | 41.1 (29.5 to 55.9) | 75.9 (54 to 106) |
|  |  | South Khorasan | 94.9 (63 to 141.3) | 170 (102.8 to 269.5) | 21.5 (14.3 to 31.4) | 50.8 (35.2 to 70.8) | 69.1 (41.4 to 107) | 1.4 (0.7 to 2.7) | 246.4 (175.6 to 326) | 1705.5 (1157.9 to 2271.1) | 333.1 (219.5 to 469.5) | 55 (33.6 to 86.5) | 6.6 (3.7 to 10.4) | 502.9 (149 to 1008.5) | 332.6 (195.7 to 552.5) | 38.1 (24.2 to 58.9) | 398.4 (280.4 to 560.6) | 227.6 (129.9 to 404.4) | 27.2 (20.3 to 35.2) | 188.5 (128.2 to 269) | 506.2 (327.8 to 736.5) | 32.5 (20.6 to 46.1) | 38.4 (27.2 to 52.7) | 74.5 (51.5 to 106.4) |
|  |  | Tehran | 68.3 (46.3 to 96.6) | 159.4 (93.1 to 256.1) | 17.6 (11.8 to 25.8) | 49.5 (34.5 to 67.8) | 73.7 (42 to 116.6) | 1.9 (0.9 to 3.4) | 214.8 (149.7 to 285.1) | 1798.3 (1251.6 to 2444.4) | 387.7 (260.6 to 545.6) | 40.1 (25.6 to 62.2) | 10.6 (6.7 to 15.8) | 536.9 (141.3 to 1063.3) | 263.5 (164.9 to 422.9) | 30.4 (20.1 to 45.1) | 373.2 (267.6 to 523.2) | 231 (126.2 to 457.3) | 30.1 (22.1 to 38.8) | 185.4 (124.7 to 261.9) | 526.2 (341.4 to 758.6) | 55.9 (35.3 to 78.4) | 21.3 (15.1 to 29.7) | 99.4 (66.7 to 144.2) |
|  |  | West Azarbayejan | 74.9 (47.3 to 109.4) | 161.6 (95.7 to 262.7) | 16.2 (10.6 to 24.1) | 50 (34.6 to 70.3) | 72 (43.4 to 109.4) | 1.6 (0.8 to 2.7) | 215.5 (149.9 to 282) | 1788.7 (1269.8 to 2354.9) | 338.5 (224.5 to 473.8) | 55.3 (33.9 to 83.8) | 7.8 (4.7 to 11.9) | 516.4 (156 to 1021.4) | 327.8 (185.8 to 530.8) | 37.7 (23.1 to 57.8) | 397.8 (281 to 566) | 229.1 (128.7 to 439.4) | 30.5 (22.7 to 39.1) | 192.3 (129.3 to 268.3) | 505.7 (323.2 to 738.6) | 23.8 (14.8 to 34.1) | 36.6 (26.2 to 50.1) | 77.3 (53.4 to 109.8) |
|  |  | Yazd | 61.8 (38.7 to 93.7) | 157.6 (93.5 to 254.6) | 18 (11.8 to 25.7) | 48.9 (33.8 to 67.9) | 73.2 (43.1 to 113.4) | 1.6 (0.8 to 2.7) | 273.6 (194.7 to 366.5) | 1620.9 (1118.7 to 2238.7) | 339.9 (224.5 to 474.3) | 50.3 (31.5 to 77.1) | 9.3 (5.5 to 14.2) | 508.9 (132.8 to 1035.1) | 326.7 (197.1 to 543.8) | 35.4 (22.7 to 55.5) | 400.6 (284 to 566) | 227.4 (127.3 to 410) | 27.6 (20.4 to 35.6) | 179.1 (123.2 to 256.9) | 515.7 (334.3 to 753.2) | 31.1 (19.5 to 44.7) | 38.5 (27.4 to 52.5) | 83.5 (58.1 to 119.9) |
|  |  | Zanjan | 92.3 (61.3 to 140.6) | 155.5 (91.5 to 251) | 15.8 (10.4 to 23.1) | 49.2 (34.3 to 68.8) | 72 (43.1 to 112.6) | 1.5 (0.8 to 2.6) | 212.9 (147.4 to 283.6) | 1695.8 (1194.6 to 2308.4) | 332.7 (215.6 to 471.3) | 51.7 (31.2 to 84.8) | 6.5 (3.8 to 10.4) | 497.8 (141.6 to 1003.4) | 312.3 (185.4 to 551.2) | 35.7 (22.4 to 56.4) | 388.9 (271.6 to 551.9) | 232.6 (127.6 to 441.7) | 27.2 (20.3 to 35.3) | 188.4 (128.9 to 266.9) | 507.6 (332.3 to 740.3) | 27 (16.4 to 38.3) | 36 (25.4 to 49.7) | 76.3 (52.5 to 109) |
| YLDs (Years Lived with Disability) | Females | Province | Causes |  |  |  |  |  |  |  |  |  |  |  |  |  |  |  |  |  |  |  |  |  |
|  |  |  | Cardiovascular diseases | Chronic respiratory diseases | Diabetes and kidney diseases | Digestive diseases | Enteric infections | HIV/AIDS and sexually transmitted infections | Maternal and neonatal disorders | Mental disorders | Musculoskeletal disorders | Neglected tropical diseases and malaria | Neoplasms | Neurological disorders | Nutritional deficiencies | Other infectious diseases | Other non-communicable diseases | Respiratory infections and tuberculosis | Self-harm and interpersonal violence | Sense organ diseases | Skin and subcutaneous diseases | Substance use disorders | Transport injuries | Unintentional injuries |
|  |  | Alborz | 85.4 (54.7 to 123.5) | 140 (81 to 228.8) | 19 (12.5 to 26.9) | 51.9 (36 to 72.5) | 78.9 (45.3 to 126) | 1.2 (0.7 to 2) | 204.3 (142.2 to 272.5) | 1948.3 (1345.3 to 2675.5) | 501.5 (337.5 to 707) | 55 (31.9 to 91.2) | 8.5 (5 to 12.7) | 597.3 (135.9 to 1250) | 373.2 (201.7 to 649.3) | 41.1 (25 to 67.6) | 449.6 (319.7 to 648.9) | 227.5 (123.1 to 449.4) | 31.2 (22.3 to 42) | 194.9 (133 to 278.1) | 555.5 (360.1 to 799.2) | 24.8 (14.9 to 37.2) | 30.4 (21 to 41.6) | 78.5 (53.4 to 112.1) |
|  |  | Ardebil | 103.3 (65.7 to 152.9) | 137.9 (79.4 to 218.4) | 17.8 (11.4 to 25.3) | 51.4 (35.5 to 70.2) | 72.1 (41.9 to 114) | 1 (0.6 to 1.7) | 209.9 (146.4 to 274.8) | 1816.6 (1225.1 to 2545.1) | 466.6 (310.6 to 661.8) | 61.3 (36.8 to 98.2) | 6.7 (3.9 to 10.6) | 566.2 (161.5 to 1185.3) | 405.9 (222.1 to 686.4) | 43.6 (26.7 to 70.1) | 455.3 (324.3 to 630.9) | 228.1 (127.6 to 434.3) | 29.6 (21.1 to 39.3) | 190.2 (129.6 to 270.2) | 541.6 (352.8 to 788.7) | 16.9 (9.6 to 25.7) | 27.3 (19.3 to 37.8) | 61.2 (41.8 to 86.6) |
|  |  | Bushehr | 63.6 (40.8 to 94.9) | 138.3 (79.6 to 220.2) | 17.8 (11.9 to 25.3) | 51.7 (34.4 to 72.4) | 74.1 (42.7 to 115.9) | 1.1 (0.6 to 1.9) | 242.7 (169.2 to 325.2) | 1865.1 (1284 to 2499.7) | 470.5 (315.3 to 659.3) | 67.1 (40.1 to 107.1) | 5.1 (3.1 to 8.1) | 577.5 (154.9 to 1228.4) | 461.2 (254.6 to 739.1) | 47.2 (28 to 72.6) | 468.2 (334.5 to 658.9) | 221 (121.1 to 401.9) | 30.5 (22.1 to 40.9) | 192.6 (132.4 to 274.9) | 548.5 (358.6 to 794.1) | 17.1 (9.8 to 26.4) | 32.4 (22.5 to 44.7) | 71 (48.7 to 102.5) |
|  |  | Chahar Mahaal and Bakhtiari | 72.7 (47.5 to 104.6) | 141.7 (81.3 to 229) | 15.2 (10.1 to 22.1) | 51.1 (34.2 to 72.4) | 75.9 (42.2 to 122.1) | 0.9 (0.5 to 1.6) | 208.8 (147.7 to 280.5) | 1648.5 (1110.3 to 2304.6) | 441 (286.7 to 621) | 67.3 (38.4 to 107.6) | 6 (3.2 to 10) | 550.8 (143.1 to 1167.2) | 439.1 (238.1 to 731.8) | 45.3 (27.1 to 70.9) | 448.9 (318.6 to 617.5) | 224.7 (125.3 to 401.4) | 26.8 (19.1 to 36.4) | 187 (127.1 to 269.7) | 541 (349.1 to 793.6) | 15.3 (8.3 to 24.2) | 29.3 (20.2 to 41.2) | 64 (43.8 to 92.9) |
|  |  | East Azarbayejan | 73.4 (48.3 to 106) | 148.3 (88.5 to 237.3) | 18.6 (12.4 to 26.8) | 51.3 (35.2 to 71.4) | 73.7 (43.5 to 119.3) | 1.1 (0.7 to 1.9) | 202.2 (140.4 to 271.7) | 1696.1 (1138.2 to 2338.3) | 389.5 (244.1 to 573.8) | 62.5 (37.1 to 101.7) | 8.1 (4.8 to 12.3) | 614.3 (167.1 to 1289.3) | 404.9 (221 to 724.8) | 43.5 (25.8 to 71.9) | 458.6 (327.8 to 646.8) | 227.9 (125.3 to 439.2) | 29.9 (21.3 to 40.2) | 194.3 (131.4 to 279.2) | 547.5 (351.3 to 798.7) | 22.1 (12.9 to 33.8) | 33.4 (23.2 to 46.4) | 73.4 (51.4 to 104.2) |
|  |  | Fars | 85 (56.2 to 122) | 138.7 (81.6 to 224.7) | 17.9 (12 to 26.2) | 52.7 (35.6 to 72.6) | 74.5 (42.9 to 117.5) | 1.5 (0.9 to 2.5) | 218.5 (154.2 to 286.3) | 1936.2 (1354.7 to 2666.8) | 472.7 (315.3 to 668.6) | 65.4 (38.6 to 102.7) | 7.9 (4.7 to 12.3) | 608.1 (147.6 to 1265.7) | 459.4 (254.9 to 754) | 46.5 (27.9 to 73.1) | 468.3 (334.2 to 668.2) | 227.2 (122.6 to 426.9) | 30.5 (21.9 to 40.8) | 191.7 (128.8 to 276.6) | 550.7 (356.7 to 798.2) | 26.1 (15.4 to 39.7) | 43.9 (30.9 to 61.4) | 75.8 (52 to 108) |
|  |  | Gilan | 106.9 (68.5 to 155) | 141.3 (81.4 to 220.7) | 18.9 (12.5 to 27.7) | 52.3 (35.5 to 72.8) | 80.8 (46.4 to 128.9) | 1.2 (0.7 to 2.1) | 196.7 (140.9 to 259) | 1893.9 (1256 to 2585.1) | 518.2 (337.6 to 733.8) | 56.1 (34 to 88.5) | 5.3 (3.2 to 8.3) | 610.5 (154.2 to 1321.1) | 373.5 (204.5 to 616.3) | 41.2 (25.4 to 63.7) | 454.9 (320.6 to 635.9) | 232.8 (126.7 to 450.6) | 32.6 (23.4 to 43.2) | 188 (125.4 to 267.4) | 556.3 (359.6 to 799.8) | 20.3 (11.4 to 30) | 29.1 (20.4 to 40.5) | 66.8 (45.3 to 94.8) |
|  |  | Golestan | 92.4 (59.2 to 133.8) | 134.2 (78.5 to 211) | 17.5 (11.6 to 25.5) | 52.1 (35 to 72.3) | 69.3 (40.9 to 110.3) | 1.1 (0.6 to 1.8) | 224.8 (157.3 to 299.1) | 1610.3 (1095.9 to 2224.9) | 446.5 (293.4 to 627) | 66.4 (39.8 to 107.2) | 4.3 (2.7 to 6.5) | 568.1 (141.8 to 1186.8) | 442.1 (247.3 to 765.3) | 46.7 (28.7 to 75.9) | 464.1 (329.5 to 653) | 229.8 (121.2 to 423.2) | 29.4 (21.2 to 38.7) | 189.1 (129 to 270.3) | 539.5 (350.6 to 779.5) | 15.8 (8.9 to 25.3) | 30.4 (21.5 to 42) | 67.1 (47 to 95.6) |
|  |  | Hamadan | 86 (55.9 to 126.4) | 137.1 (80.4 to 220.7) | 16.7 (11.1 to 24.1) | 52.6 (35.8 to 73.2) | 71.9 (41.6 to 112.6) | 1 (0.6 to 1.7) | 213.2 (152.8 to 285.6) | 1678.7 (1146 to 2323.8) | 453 (293.1 to 647) | 66.5 (39.9 to 106.9) | 6.8 (4.1 to 10.4) | 569.9 (173.5 to 1179.7) | 448.3 (246 to 715.5) | 47.1 (28.6 to 72.6) | 458.2 (320.6 to 643.4) | 233.1 (124.6 to 435.1) | 29.8 (21.4 to 39.8) | 188.9 (129.4 to 272.8) | 541 (352.6 to 782.8) | 28.9 (17.4 to 42.4) | 37.1 (25.9 to 52.6) | 72.9 (49.9 to 104.1) |
|  |  | Hormozgan | 82.7 (54.1 to 120.9) | 135.1 (76.9 to 218.5) | 17.5 (11.6 to 25.6) | 50.9 (35.3 to 71.3) | 70 (41.8 to 111.6) | 1.9 (1.2 to 2.9) | 220.2 (151.1 to 292) | 1742.9 (1178.7 to 2408.7) | 430.2 (286.4 to 598.1) | 68 (40.3 to 109) | 3.9 (2.5 to 6) | 547.1 (139.9 to 1153.3) | 475.7 (267.6 to 800.5) | 45.9 (27.1 to 73.4) | 452.2 (322.2 to 641.4) | 222.6 (124.1 to 422.6) | 26.8 (19.1 to 36) | 187.3 (125.6 to 272.5) | 538 (350.1 to 785.1) | 30.7 (17.4 to 48.6) | 29.3 (20.6 to 40.2) | 62.9 (43.7 to 89.8) |
|  |  | Ilam | 86.7 (57.1 to 128) | 139.4 (78.2 to 223.7) | 18.7 (12.8 to 26.4) | 50.1 (34.6 to 69.4) | 71.2 (40.5 to 111.9) | 0.9 (0.5 to 1.6) | 219.3 (151.6 to 295) | 1848.8 (1239.4 to 2571.7) | 443.1 (295.9 to 630.7) | 64.5 (37.9 to 103.9) | 5.8 (3.2 to 9.6) | 542.7 (150 to 1124.9) | 440.4 (245.5 to 751.3) | 45.6 (27.3 to 72.1) | 455.7 (322.7 to 639.1) | 224.4 (122.6 to 427.2) | 32 (23.1 to 42.1) | 185.4 (124.3 to 267.1) | 542.5 (353.6 to 788.6) | 15.7 (8.8 to 24.1) | 31.8 (22.2 to 43.7) | 64.3 (43.4 to 90.8) |
|  |  | Isfahan | 81 (52.5 to 115.9) | 140.1 (82.9 to 226.7) | 18 (12.3 to 26.6) | 52 (35.2 to 71.4) | 78 (45.2 to 123.9) | 1.1 (0.6 to 1.9) | 218.2 (153.3 to 298.6) | 1955.7 (1330 to 2639.6) | 488.4 (325.7 to 680) | 62.7 (37.2 to 102.5) | 8.1 (4.7 to 12.9) | 599.3 (154.1 to 1264) | 443.9 (260.1 to 746.5) | 45.8 (29 to 72.2) | 472.3 (334.7 to 665.2) | 229.3 (122.3 to 433.2) | 30.6 (22 to 41.2) | 194 (132.5 to 277.4) | 552.2 (359.8 to 800.6) | 19.1 (11.2 to 29.7) | 33.9 (23.5 to 47.5) | 79.7 (53.9 to 113.5) |
|  |  | Kerman | 82.1 (55.3 to 120.8) | 146.2 (87 to 231.1) | 18.4 (12.2 to 27.3) | 52.9 (35.6 to 73.9) | 81.6 (49.7 to 125.3) | 1.1 (0.6 to 1.9) | 270.1 (187.3 to 360) | 1922.8 (1322.1 to 2666.4) | 452.7 (297.4 to 636.4) | 65.5 (38.8 to 105.4) | 6.6 (3.9 to 10.2) | 573.3 (148 to 1223.7) | 460.3 (262.8 to 783.1) | 47.1 (28.9 to 73.9) | 461.8 (327.7 to 648.2) | 227.8 (123.3 to 430.9) | 28.5 (20.4 to 38.1) | 189.3 (126.7 to 272.3) | 541.3 (351.3 to 792.1) | 20.1 (11.9 to 30.4) | 42 (29.3 to 56.8) | 106.3 (78.6 to 140.6) |
|  |  | Kermanshah | 85.8 (56.5 to 125.2) | 137.9 (81.9 to 215.5) | 18.3 (12.2 to 25.9) | 52.1 (36.4 to 74.9) | 70.7 (40.6 to 110.6) | 1.8 (1.1 to 2.8) | 223.1 (155.4 to 300.2) | 1784.5 (1207.6 to 2407.8) | 454.5 (303.7 to 640.4) | 65.8 (39.8 to 106.2) | 6.4 (3.8 to 10.1) | 559 (142.8 to 1187.5) | 444.9 (247.6 to 723.2) | 47.4 (28.8 to 72.7) | 467.4 (334.3 to 655) | 233.3 (124.2 to 462.1) | 32.1 (23.2 to 42.1) | 188.3 (129.2 to 267.9) | 540.2 (348.2 to 798) | 30.9 (17.4 to 47.1) | 30 (21.3 to 41.5) | 81 (58.6 to 114.1) |
|  |  | Khorasan-e-Razavi | 76.3 (48.1 to 109.4) | 140.4 (82.7 to 224) | 18.3 (12.1 to 26.1) | 52.3 (34.8 to 73.5) | 71.4 (40.9 to 111.8) | 1.2 (0.8 to 2) | 223.5 (152.5 to 299.2) | 1682.3 (1121.5 to 2321.4) | 453.9 (304.9 to 639.6) | 64.8 (39.7 to 99.2) | 6.4 (3.7 to 10.4) | 567.4 (141.7 to 1150.3) | 416.6 (236.1 to 679.4) | 44.4 (27.4 to 69.6) | 462.9 (332.1 to 656.2) | 229.3 (127.5 to 441.8) | 28.2 (20.2 to 37.7) | 242.2 (166.3 to 346.4) | 540.9 (350 to 785) | 22.3 (13.4 to 32.8) | 33.9 (23.9 to 46.4) | 73.3 (51 to 102.5) |
|  |  | Khuzestan | 80.9 (53.4 to 113.6) | 123.3 (72.6 to 203.8) | 17.9 (11.9 to 26) | 50.5 (34.4 to 70.3) | 75.8 (44.2 to 119.9) | 1.1 (0.7 to 1.8) | 241.5 (169.5 to 324.4) | 1743.6 (1194.9 to 2437.5) | 432.1 (280.6 to 606.1) | 69.4 (42.5 to 110.4) | 6.8 (4.3 to 10.4) | 549.9 (163.8 to 1134.8) | 473.8 (263.8 to 769.8) | 48.9 (29.5 to 76.6) | 466.9 (328.1 to 650.7) | 227.7 (129.1 to 421.5) | 29 (20.9 to 38.6) | 182.8 (125.2 to 267.2) | 540.4 (343.3 to 788.6) | 18.3 (10.8 to 26.9) | 32.3 (22.8 to 45.2) | 71.5 (49.3 to 103.4) |
|  |  | Kohgiluyeh and Boyer-Ahmad | 81 (53.8 to 120.6) | 142.4 (83.6 to 228.6) | 15.3 (9.9 to 22) | 49.5 (33.6 to 69.6) | 80.6 (48.5 to 129) | 0.9 (0.5 to 1.6) | 232.2 (161.5 to 313.8) | 1667.9 (1146.2 to 2292.4) | 406.8 (269.5 to 577.1) | 67.8 (41.5 to 109.5) | 9.5 (5.7 to 14.6) | 522.8 (133.9 to 1105.9) | 466.5 (269.1 to 771.8) | 47.9 (30.2 to 75) | 454.2 (325.1 to 638.3) | 223.3 (125.2 to 421.9) | 26.2 (18.7 to 35) | 181.1 (121 to 261.6) | 535.7 (342 to 783.2) | 16.2 (9.5 to 24.6) | 37.6 (26.3 to 52.6) | 87.1 (58.2 to 123.4) |
|  |  | Kurdistan | 83 (54.1 to 123.9) | 136.7 (80 to 216.7) | 16.8 (10.9 to 24.3) | 51.7 (34.8 to 72.4) | 70.6 (40 to 111.1) | 1.1 (0.6 to 1.8) | 213.1 (149.7 to 281.6) | 1748.4 (1157.3 to 2433.9) | 451.7 (292.3 to 646.8) | 65.6 (39.4 to 107.2) | 5.9 (3.2 to 8.9) | 552.6 (144.7 to 1200.7) | 429.1 (234.8 to 735.5) | 44.8 (27.5 to 72.1) | 454.5 (320.6 to 636.2) | 227.9 (130.2 to 416) | 29.1 (20.9 to 39) | 181.1 (122.5 to 258.1) | 537.9 (346.7 to 775.7) | 15.9 (9.3 to 24.5) | 31.9 (22.1 to 43.8) | 66.2 (45.7 to 94.1) |
|  |  | Lorestan | 68.9 (46 to 96.8) | 140.7 (82.4 to 226.9) | 15.9 (10.5 to 23.2) | 50.1 (34.5 to 70.4) | 72.4 (41.8 to 116) | 1.1 (0.6 to 1.9) | 213.3 (147.8 to 283.7) | 1617.4 (1119.2 to 2250.5) | 447.7 (294.4 to 633) | 63.2 (38.2 to 101.6) | 4.9 (2.8 to 7.5) | 547.3 (145.4 to 1159.9) | 429.9 (244.5 to 733.1) | 45.3 (28.4 to 71.6) | 444 (313.2 to 632) | 228.7 (129.4 to 437.9) | 28 (20 to 37.3) | 187.8 (125.3 to 268) | 538.8 (345.3 to 791.7) | 18 (10.5 to 27.6) | 24.3 (17.2 to 34.1) | 60.8 (42 to 87) |
|  |  | Markazi | 79.1 (52.4 to 113.8) | 141 (85.4 to 230.6) | 19.4 (12.9 to 28.2) | 52.8 (35 to 72.8) | 73.9 (43 to 118) | 1 (0.6 to 1.8) | 199.1 (140 to 261.4) | 1887.5 (1318.3 to 2541.7) | 483.1 (319.6 to 669.8) | 62.8 (37.2 to 100.1) | 7.6 (4.4 to 12.1) | 585.9 (157.3 to 1230.1) | 421.7 (227.2 to 731.4) | 44.3 (26.6 to 71) | 466 (333.5 to 655) | 231.6 (132.7 to 445.7) | 29.8 (21.1 to 40.5) | 193.4 (132.2 to 275.6) | 548 (353.8 to 786.7) | 21.3 (12.2 to 32.8) | 37.8 (26.6 to 52.4) | 78.1 (53.4 to 111.7) |
|  |  | Mazandaran | 90 (58.1 to 132) | 140.7 (83.3 to 223.9) | 18.5 (12.4 to 26.5) | 50.9 (35.7 to 71.1) | 80 (46.9 to 125.1) | 1.1 (0.6 to 1.9) | 206 (140 to 275.7) | 2027.1 (1364.1 to 2849.5) | 755 (505.2 to 1047.8) | 54.7 (33.6 to 87.4) | 7.2 (4.4 to 10.6) | 605.2 (158.1 to 1285) | 370.7 (208.1 to 628.8) | 40.7 (24.6 to 66.6) | 453 (320.9 to 642.3) | 228.6 (123.8 to 454.6) | 31.3 (22.8 to 42.1) | 194.6 (130.4 to 278.5) | 554.5 (354.5 to 811.4) | 18.1 (10.3 to 28.4) | 37.5 (26.5 to 52.2) | 73.8 (50.8 to 105) |
|  |  | North Khorasan | 87 (57.4 to 127) | 141.2 (85.4 to 226.2) | 18.3 (12.2 to 26.5) | 51.9 (35.9 to 72.4) | 72.1 (43.2 to 111.8) | 1 (0.6 to 1.7) | 214.8 (148.2 to 286.4) | 1768.4 (1189.6 to 2444) | 446.6 (288.6 to 632.3) | 66.2 (39.5 to 105.1) | 7 (4.1 to 10.9) | 566 (176.8 to 1155.9) | 434.5 (242.9 to 761.4) | 45.2 (27.7 to 72.2) | 462.1 (331.8 to 649.7) | 230.3 (130.8 to 443.2) | 28.2 (20.3 to 37.3) | 188.6 (127 to 266.6) | 538 (350.3 to 789.4) | 19.3 (11.3 to 28.5) | 34.6 (24.5 to 48.1) | 79.5 (53.4 to 112.8) |
|  |  | Qazvin | 82.8 (53.6 to 120.4) | 138.4 (81.7 to 220) | 17.7 (11.5 to 25.9) | 53.6 (36.5 to 75) | 72.1 (41.5 to 116.1) | 1.1 (0.6 to 1.8) | 209.4 (146.1 to 276.4) | 1759.7 (1188.9 to 2449.2) | 469 (311.2 to 654.3) | 61.3 (34 to 95.9) | 5.9 (3.3 to 9.1) | 572.4 (158.5 to 1166.3) | 413.2 (223.6 to 693.9) | 43 (25.5 to 68.2) | 454.9 (326.3 to 643.7) | 226.1 (122.3 to 440.9) | 29.1 (20.5 to 39.3) | 191 (131.5 to 274.2) | 545.7 (351.4 to 797.1) | 19.8 (11.4 to 30.5) | 29.3 (20.7 to 40.8) | 74.8 (51.3 to 107.2) |
|  |  | Qom | 52.2 (34.1 to 76.9) | 139 (82.7 to 224.2) | 18.8 (12.5 to 26.9) | 51.5 (34.7 to 72.2) | 71.9 (41.9 to 112.9) | 1.1 (0.7 to 1.9) | 216.7 (153.5 to 286.1) | 1835.2 (1257.7 to 2504.2) | 457.2 (300.6 to 647) | 61.4 (36.3 to 98.6) | 7.3 (3.9 to 11.7) | 568.3 (151.5 to 1152.6) | 407.2 (221.6 to 694.6) | 43 (25.8 to 69.5) | 451.7 (321.9 to 625.8) | 223.2 (121.2 to 434.3) | 28.3 (20.3 to 38.4) | 191 (129.1 to 271.2) | 545.3 (349.9 to 801.9) | 16.6 (9.1 to 24.9) | 21.3 (15.1 to 29.2) | 71.1 (48.7 to 101.6) |
|  |  | Semnan | 81.9 (53.6 to 119.1) | 138.3 (82 to 221.7) | 20 (13.2 to 28.1) | 51.8 (35.6 to 71.4) | 70.2 (41.7 to 112.5) | 1.1 (0.6 to 1.9) | 218.3 (151.8 to 290.8) | 1751 (1211.7 to 2373.3) | 486.4 (318.5 to 683.7) | 60.9 (35.8 to 94.1) | 5.5 (3.3 to 8.7) | 594.6 (167.4 to 1237.6) | 406.7 (230.7 to 671.1) | 43.1 (25.8 to 68) | 461.4 (327.8 to 647.2) | 226.8 (124.7 to 444.6) | 30.5 (21.6 to 40.9) | 180.3 (123.2 to 257.1) | 551.9 (359.9 to 790.9) | 19.5 (10.7 to 30) | 32.3 (22.6 to 45) | 69.3 (47.5 to 100.1) |
|  |  | Sistan and Baluchistan | 70.8 (47 to 101.6) | 153.8 (93.8 to 240.3) | 18.3 (12.2 to 26.2) | 55.9 (38.2 to 78.2) | 72.3 (45.1 to 111) | 1.4 (0.9 to 2.2) | 253.9 (177 to 334.3) | 1688 (1139.7 to 2328.3) | 407.1 (267.5 to 564.6) | 79.9 (48.2 to 121.5) | 4.4 (2.8 to 6.8) | 534.2 (167 to 1123.6) | 532 (300.5 to 839.2) | 54.6 (33.2 to 83.2) | 478.4 (344.7 to 667) | 228 (127.9 to 409.3) | 27.9 (20.2 to 36.7) | 189.6 (128.9 to 271.8) | 522.2 (336.4 to 768.7) | 25.5 (15.4 to 37.5) | 37 (26.4 to 50.3) | 68.4 (48.1 to 94.7) |
|  |  | South Khorasan | 85.1 (55.7 to 123) | 146.5 (88.7 to 230.3) | 23 (15.1 to 34) | 52.2 (35.8 to 73.8) | 72.3 (43.6 to 112.9) | 0.9 (0.5 to 1.7) | 238.8 (170.1 to 319.5) | 1784.8 (1185.5 to 2428.4) | 451.4 (293.9 to 643.3) | 64.3 (38.3 to 104.9) | 5.6 (3 to 8.8) | 554.8 (146.4 to 1156.2) | 424.8 (240.4 to 709.1) | 45.7 (28.9 to 72.3) | 453.1 (323.6 to 629.2) | 224.9 (124.9 to 392.5) | 28 (19.9 to 37.7) | 187.9 (126.9 to 269.3) | 538.1 (346.1 to 791.8) | 20 (12 to 29) | 34.2 (23.9 to 48.3) | 64.2 (43.4 to 91.8) |
|  |  | Tehran | 65.6 (43.4 to 93.1) | 142.5 (83.2 to 228.7) | 18.5 (12.3 to 27.2) | 51.2 (34.4 to 71.3) | 76.5 (42.5 to 119.5) | 1.2 (0.7 to 2) | 204.6 (139.7 to 271.9) | 1999 (1351.7 to 2744.4) | 498.7 (333.4 to 703.3) | 51.4 (31.8 to 82.7) | 11.2 (7.1 to 16.8) | 591.4 (136.4 to 1217.3) | 385.3 (233.2 to 647.6) | 39.1 (24.8 to 62.3) | 444.9 (317.8 to 628.1) | 229.2 (125.9 to 448.6) | 30.1 (21.5 to 40.6) | 185.9 (125.1 to 262.6) | 557.6 (360.6 to 809.9) | 36.7 (21.6 to 55) | 19.1 (13.2 to 26.7) | 89.2 (59.9 to 127.2) |
|  |  | West Azarbayejan | 70.7 (46.2 to 104.6) | 144.7 (86.3 to 234.2) | 16.9 (11.1 to 24.6) | 52.4 (35.7 to 72.2) | 76.2 (45.1 to 117.8) | 1.1 (0.7 to 1.8) | 207.5 (143.1 to 274.5) | 1995.5 (1377.1 to 2649.4) | 460.6 (305.5 to 644.4) | 64.2 (38.7 to 104.2) | 7.7 (4.6 to 11.8) | 570.9 (148.7 to 1148.5) | 416.1 (216.4 to 713.3) | 45.1 (26.9 to 70) | 454.9 (321.1 to 645.2) | 226.5 (126.7 to 435.6) | 31.1 (22.7 to 41.4) | 193.8 (130.2 to 275.8) | 537.1 (343.1 to 786.8) | 16.3 (9.3 to 25) | 31.9 (22.7 to 44.3) | 69.1 (47.8 to 98.8) |
|  |  | Yazd | 58.1 (36.4 to 86.4) | 140 (82.8 to 222.2) | 18.7 (11.9 to 27.1) | 50.9 (35.1 to 72.6) | 76.2 (44.9 to 119.3) | 1.1 (0.6 to 1.9) | 263.6 (187.7 to 353.8) | 1729 (1155.4 to 2388.4) | 461.5 (305.1 to 644.9) | 61.8 (36.5 to 95.7) | 9 (5 to 13.8) | 550.3 (139.7 to 1146.1) | 432.5 (239.4 to 753.7) | 44.2 (26.2 to 70.7) | 460.8 (327.6 to 654.7) | 225.1 (125.2 to 405.4) | 28.3 (20.3 to 37.9) | 178.4 (122.4 to 257.3) | 548.2 (357.3 to 805.7) | 18 (10.1 to 27.3) | 35 (24.6 to 48.1) | 75.4 (51.4 to 108) |
|  |  | Zanjan | 85.6 (56.4 to 127.1) | 138.1 (82.1 to 222.2) | 16.6 (10.9 to 24.1) | 51.2 (35.7 to 71.9) | 74 (41.8 to 118.2) | 1.1 (0.6 to 1.7) | 204.5 (143.7 to 273.8) | 1794.1 (1236.2 to 2439.2) | 452.7 (293.2 to 645) | 61.6 (36.9 to 97.7) | 5.7 (3.2 to 9.1) | 554.3 (142.1 to 1165.2) | 409.2 (223.1 to 695) | 43.6 (26.3 to 70.8) | 447.3 (311.9 to 629.6) | 229.6 (126.2 to 435.9) | 28.1 (20.2 to 37.9) | 189.2 (128.2 to 270.1) | 540.6 (351.2 to 796.4) | 16.3 (9 to 25) | 32.8 (22.7 to 45.5) | 68.9 (45.8 to 97.7) |
| YLDs (Years Lived with Disability) | Males | Province | Causes |  |  |  |  |  |  |  |  |  |  |  |  |  |  |  |  |  |  |  |  |  |
|  |  |  | Cardiovascular diseases | Chronic respiratory diseases | Diabetes and kidney diseases | Digestive diseases | Enteric infections | HIV/AIDS and sexually transmitted infections | Maternal and neonatal disorders | Mental disorders | Musculoskeletal disorders | Neglected tropical diseases and malaria | Neoplasms | Neurological disorders | Nutritional deficiencies | Other infectious diseases | Other non-communicable diseases | Respiratory infections and tuberculosis | Self-harm and interpersonal violence | Sense organ diseases | Skin and subcutaneous diseases | Substance use disorders | Transport injuries | Unintentional injuries |
|  |  | Alborz | 92.1 (57.2 to 137.1) | 173.8 (100.6 to 281.2) | 16.7 (10.6 to 24.7) | 48.4 (33.3 to 69.3) | 71.2 (41.1 to 109.9) | 2.4 (1.1 to 4.9) | 223 (155.2 to 304) | 1707.2 (1223.2 to 2330.8) | 240.1 (159.3 to 347.6) | 33.3 (18.9 to 59.1) | 9 (5.5 to 13.8) | 477.2 (130.5 to 963.8) | 164.2 (76.1 to 324.7) | 24.3 (14.1 to 41.5) | 315.4 (219.6 to 440.9) | 231.9 (128.3 to 439.7) | 28.8 (21 to 37.1) | 195.4 (131.3 to 279.5) | 494.4 (310.3 to 723.4) | 66 (39.5 to 94.2) | 36.3 (25.6 to 49.8) | 107.1 (74.2 to 152.4) |
|  |  | Ardebil | 128.7 (83.2 to 196.8) | 171.7 (100.7 to 285.2) | 16.2 (10.8 to 22.8) | 46.4 (31.4 to 65.2) | 67.7 (40.2 to 104.9) | 2 (0.7 to 4.2) | 228.6 (157.2 to 303) | 1617.3 (1121.9 to 2135.6) | 223.8 (150 to 316.8) | 43.6 (25.5 to 74.5) | 9 (4.9 to 14.8) | 459.9 (151.7 to 920.5) | 229.5 (110.7 to 442.3) | 29.2 (16.3 to 50.5) | 343.3 (241.2 to 476.2) | 233.3 (129.5 to 455.2) | 27.9 (20.8 to 35.8) | 188.3 (124.8 to 268.8) | 477.5 (307 to 694.6) | 35.2 (21.8 to 53.1) | 35 (25.1 to 48.6) | 80.3 (55.5 to 113.2) |
|  |  | Bushehr | 76.1 (47.8 to 114.8) | 172 (97.6 to 280.2) | 16.6 (11.1 to 24.6) | 47.1 (32.5 to 66.9) | 69.3 (39.5 to 109) | 2.2 (0.9 to 3.9) | 263.4 (183.5 to 348.4) | 1677.8 (1198.6 to 2294.6) | 231.4 (144.8 to 330.6) | 39.9 (23.6 to 66) | 6.7 (3.9 to 10.9) | 468.4 (153.2 to 950.8) | 201.5 (101.7 to 366.4) | 26.8 (16.2 to 42.7) | 334.3 (239.7 to 469) | 226.6 (124 to 413.4) | 26.9 (19.9 to 34.3) | 191.4 (129.7 to 278.2) | 488.4 (315.8 to 710.8) | 34.3 (21.2 to 49.4) | 40.8 (28.9 to 56) | 79.8 (55.5 to 114.7) |
|  |  | Chahar Mahaal and Bakhtiari | 80.5 (51.7 to 119.5) | 178.1 (104.5 to 294) | 13.6 (8.8 to 20) | 47 (32 to 68.2) | 71.3 (39.7 to 114.1) | 1.9 (0.7 to 3.7) | 224 (156.2 to 299.4) | 1485.3 (1056.7 to 2022.9) | 212.3 (137.5 to 302.2) | 39.9 (23.1 to 65.7) | 6 (3.3 to 9.8) | 443.5 (129 to 892) | 177.1 (86.4 to 350.8) | 24.5 (14.5 to 40.8) | 323.1 (224.2 to 463.9) | 229.9 (125.7 to 421.8) | 25.1 (18.5 to 32.4) | 186.3 (122.7 to 265) | 476.7 (305.2 to 700.7) | 29 (17.8 to 43.3) | 35.9 (25.6 to 49.5) | 85.7 (59.9 to 122.5) |
|  |  | East Azarbayejan | 82.8 (53.3 to 122.9) | 177.7 (105.1 to 286.3) | 16.8 (10.8 to 24.8) | 47.1 (32.7 to 68.6) | 67.4 (39.3 to 105.1) | 2.1 (0.8 to 4.4) | 220.7 (154.6 to 291.8) | 1562.9 (1084.7 to 2087.7) | 206.2 (129.5 to 314.7) | 43.4 (24 to 73.5) | 8.2 (4.9 to 13.3) | 503.8 (167.7 to 1003.4) | 220.3 (100.3 to 414.9) | 28.2 (15.8 to 46.4) | 327.3 (230 to 469.4) | 233.7 (126 to 455.4) | 27.9 (20.8 to 35.5) | 194 (130.8 to 274.8) | 485.2 (309.7 to 704.2) | 51.1 (32.3 to 74.5) | 42.1 (29.9 to 57.7) | 89.6 (61.3 to 125.9) |
|  |  | Fars | 94.3 (61.6 to 138.5) | 176.2 (103.6 to 284.7) | 15.8 (9.8 to 24) | 48.5 (33.9 to 68.1) | 70.3 (41.3 to 114.7) | 2.8 (1.5 to 5.1) | 236.6 (166.1 to 318.4) | 1802.5 (1266.9 to 2462) | 228.1 (147.7 to 322.8) | 39.9 (22.6 to 65.4) | 9.6 (5.5 to 15.8) | 482.6 (147.8 to 960.6) | 227.6 (116.1 to 432) | 27.1 (16.2 to 45.3) | 342.9 (240 to 486.8) | 232.6 (128.6 to 444.8) | 29.9 (22.3 to 38.3) | 190.4 (126.9 to 273.2) | 486.7 (316.9 to 714.1) | 57.3 (35.9 to 80.8) | 56.6 (40.2 to 77.8) | 102 (70.9 to 144.5) |
|  |  | Gilan | 117.5 (78.5 to 175.8) | 182.8 (105.4 to 287.7) | 17.1 (11.3 to 24.6) | 46.7 (33 to 66.1) | 76 (44.4 to 122.8) | 2.2 (0.9 to 4.8) | 212.2 (150 to 280.3) | 1625.1 (1143.4 to 2194.2) | 248.9 (158.1 to 353.8) | 35.5 (20.2 to 59.7) | 7.7 (4.9 to 11.9) | 493.6 (138.5 to 993) | 192.7 (103.1 to 347.4) | 25.1 (15.1 to 39.6) | 323.3 (226.3 to 461.2) | 239 (131.1 to 473.9) | 31.7 (23.2 to 41.3) | 189 (127.8 to 274.6) | 496 (312.1 to 728.3) | 50.9 (31 to 75.1) | 43.1 (29.8 to 60.7) | 89.9 (62.3 to 129.2) |
|  |  | Golestan | 107.6 (69.1 to 160.8) | 176.9 (105.1 to 291.9) | 16.7 (11 to 24.2) | 48.1 (32.2 to 68.1) | 67.7 (38.8 to 106.2) | 2.1 (0.9 to 4.2) | 242 (164.4 to 323.3) | 1501 (1069.8 to 2028.8) | 214.9 (139.6 to 306.2) | 50.2 (27 to 81.6) | 7.3 (4.6 to 11.5) | 472.8 (141.9 to 923.2) | 287.3 (132.8 to 538.4) | 33.6 (19.2 to 55.8) | 365.2 (250.5 to 513.4) | 234.4 (128 to 425) | 28 (20.4 to 35.6) | 187.8 (126 to 269.8) | 475.1 (307.8 to 692.9) | 35 (21.4 to 50.7) | 45.9 (32.7 to 63.6) | 95.2 (66.2 to 133.3) |
|  |  | Hamadan | 92.1 (58.6 to 140.5) | 174.9 (100.4 to 279.3) | 15.4 (10.3 to 22) | 48.4 (31.9 to 69.3) | 68.7 (41 to 108.8) | 2 (0.9 to 3.8) | 236.2 (168.5 to 319.4) | 1506.2 (1049 to 2033.3) | 217.1 (140.7 to 306) | 46 (26.4 to 78.8) | 8.9 (5.4 to 14) | 466 (162.6 to 943.4) | 255.6 (125.5 to 494.8) | 30.9 (18.2 to 52.4) | 354.2 (251 to 494.9) | 237.8 (130.4 to 463.9) | 29 (21.2 to 37) | 187.5 (125 to 269.2) | 477.8 (312 to 712.2) | 66.7 (41.9 to 94.3) | 45.1 (32 to 61.4) | 94.9 (66 to 133.6) |
|  |  | Hormozgan | 96.6 (62.3 to 145.7) | 173.2 (100.6 to 284.9) | 17.2 (11.4 to 24.1) | 48 (32.8 to 68.7) | 74.1 (44.8 to 114.6) | 2.7 (1.4 to 4.6) | 237.9 (162 to 315) | 1573.5 (1103.7 to 2101.1) | 208.5 (137.4 to 290.2) | 48.7 (27.6 to 80.3) | 7.4 (4.1 to 12.2) | 455.5 (156 to 911) | 308.8 (165.2 to 529.1) | 30.7 (18.1 to 48.5) | 353.3 (242.4 to 495.3) | 227.7 (127 to 435.1) | 25.4 (18.8 to 32.5) | 185.6 (124.1 to 265.7) | 474.7 (305.5 to 686.2) | 75.6 (47.6 to 110.9) | 42 (29.5 to 57.8) | 85.5 (59.7 to 121) |
|  |  | Ilam | 94.3 (61.6 to 144.7) | 174 (99.6 to 285.2) | 18.6 (12.4 to 26.9) | 46 (31.7 to 64.7) | 69.3 (39.4 to 110.1) | 1.8 (0.7 to 3.9) | 243.1 (169.7 to 327.1) | 1570.1 (1095.2 to 2124) | 216.1 (140.5 to 306.6) | 42 (23.1 to 72.8) | 7.4 (4.2 to 11.8) | 444.6 (141.5 to 871.2) | 221.4 (108.1 to 446) | 28.2 (16.9 to 48.5) | 347.4 (236.7 to 505.3) | 230 (128.6 to 434.2) | 30.2 (22.1 to 39.1) | 184 (124.6 to 264.8) | 478.9 (307.1 to 690.5) | 31.5 (18.7 to 46.8) | 40.3 (27.9 to 55.1) | 86 (59.8 to 123.3) |
|  |  | Isfahan | 92.3 (58.1 to 137.8) | 175.3 (104 to 282.8) | 15.9 (10.5 to 23.1) | 47.4 (32.5 to 65.7) | 73.2 (43.5 to 117.6) | 2.1 (0.9 to 4.1) | 236.9 (164.4 to 327.2) | 1582.9 (1125.9 to 2116.4) | 234.9 (153.5 to 331.9) | 37 (21.2 to 61.7) | 9.4 (5.4 to 14.8) | 485 (153.5 to 978.2) | 215.7 (111.8 to 400.9) | 25.8 (15.5 to 42.9) | 337 (233.8 to 476.3) | 234.5 (123.6 to 453.7) | 28.9 (20.9 to 37.2) | 192.5 (130.1 to 277.4) | 490.3 (312.4 to 725.4) | 50.2 (31.9 to 73.7) | 44 (30.8 to 61.9) | 103.1 (70 to 147.5) |
|  |  | Kerman | 92.1 (57.9 to 139.1) | 177.4 (105.2 to 284.6) | 17 (11.3 to 25.2) | 49.6 (34.3 to 69.5) | 71.7 (41.7 to 111) | 2.1 (0.9 to 3.9) | 287 (200.2 to 384.6) | 1750.4 (1246 to 2332.9) | 219.2 (142.2 to 311.8) | 46.5 (26.3 to 79.8) | 7.3 (4.2 to 11.8) | 493.2 (150.9 to 1009.6) | 295.1 (150.3 to 533.8) | 32 (18.6 to 52.7) | 356.1 (250.8 to 499.9) | 232.8 (125.8 to 444.4) | 27.4 (20.2 to 34.9) | 187.1 (125.2 to 268.4) | 479 (305.2 to 697.3) | 42.2 (25.4 to 62.4) | 49.1 (34.7 to 67.6) | 117.9 (88.8 to 157.6) |
|  |  | Kermanshah | 93.4 (57.7 to 138.6) | 174.4 (103.2 to 280.3) | 16.6 (10.9 to 24.6) | 48 (32.5 to 69) | 67.5 (39.1 to 105.4) | 3 (1.6 to 5.1) | 243.4 (168.3 to 322.5) | 1642.4 (1157.9 to 2222.8) | 219 (143.1 to 313) | 46 (25.7 to 75.4) | 8.2 (4.5 to 13) | 451.2 (130.5 to 905.2) | 243.9 (117 to 465.8) | 31.3 (18.3 to 51) | 355.3 (254.2 to 510.1) | 237.3 (127.4 to 456.4) | 31.4 (22.9 to 40.8) | 188.2 (124 to 270.6) | 478.8 (309.6 to 703.1) | 59.4 (37.3 to 83.9) | 41.2 (28.8 to 57.6) | 93.8 (66.2 to 131) |
|  |  | Khorasan-e-Razavi | 86.8 (55.3 to 130.8) | 173.8 (104.1 to 288) | 19.7 (13.3 to 29.1) | 48 (32.8 to 67.9) | 65.7 (39.4 to 102) | 2 (0.8 to 3.9) | 246.8 (170.8 to 321.6) | 1494.4 (1048 to 2060.2) | 218.8 (144.3 to 312.7) | 48 (27.3 to 79.6) | 7.6 (4.5 to 12.3) | 459.2 (142.7 to 886.7) | 251.4 (127.7 to 482.1) | 30.8 (18.2 to 50.9) | 358 (249.8 to 500.1) | 233.7 (130.5 to 450.2) | 27 (20.1 to 34.7) | 231.6 (158.1 to 331.4) | 478.3 (307.6 to 707) | 50.6 (31.7 to 72.6) | 41.4 (29.3 to 55.5) | 90 (61.9 to 127.9) |
|  |  | Khuzestan | 85.9 (55.9 to 126.3) | 161.8 (94.4 to 263.8) | 16.6 (11.2 to 24.1) | 46.3 (31.9 to 66.1) | 68.5 (39.6 to 106) | 2 (0.9 to 3.9) | 263.8 (185.3 to 352.8) | 1574.1 (1105.3 to 2144) | 208.9 (138.3 to 299.7) | 43.9 (23.9 to 76.1) | 8 (4.8 to 12.4) | 451.2 (154.2 to 890.9) | 236.5 (111.2 to 469.4) | 29.5 (16.8 to 49.9) | 351.9 (241.1 to 503.4) | 232.9 (132.8 to 430.8) | 26.2 (19.1 to 33.1) | 181.9 (123.1 to 257.7) | 476.1 (313 to 704.5) | 43 (25.9 to 63.6) | 40.1 (28.5 to 54.1) | 88 (60.8 to 125.5) |
|  |  | Kohgiluyeh and Boyer-Ahmad | 86 (55.6 to 126.8) | 178.6 (102.6 to 285.8) | 14.2 (9.4 to 20.3) | 45.8 (32.1 to 62.6) | 70.9 (41 to 113.7) | 1.9 (0.7 to 3.8) | 249.7 (169.6 to 331.8) | 1530 (1079.8 to 2045.1) | 198.8 (128 to 288.1) | 39.9 (23 to 71.6) | 10.6 (6.2 to 17.5) | 431.5 (135.4 to 866.5) | 215.5 (109.4 to 429.3) | 27.3 (16.4 to 47.8) | 345.1 (242.3 to 487.8) | 228.4 (128.5 to 419.2) | 24.3 (17.7 to 31.2) | 181.1 (120.5 to 257.8) | 470.4 (300.6 to 685.4) | 31.8 (19.9 to 48.4) | 41.9 (29.7 to 57.8) | 96.3 (66.3 to 136.3) |
|  |  | Kurdistan | 94.8 (60.2 to 147.1) | 175.7 (103.1 to 293) | 15.8 (10.4 to 23.4) | 48.1 (32.7 to 67) | 68.1 (40 to 108.7) | 2 (0.8 to 4) | 232.6 (167.6 to 308.1) | 1535.1 (1078.4 to 2089.9) | 215.4 (140.9 to 305.7) | 45.8 (25.2 to 77) | 8.5 (4.7 to 14.3) | 452.3 (149.2 to 913.1) | 235.2 (109.9 to 448.1) | 29.4 (16.8 to 48.3) | 345.9 (240 to 486.1) | 233.7 (133.5 to 426) | 29.9 (22.1 to 38.6) | 181.7 (119.6 to 258) | 473.6 (310.5 to 691.9) | 36.5 (21.9 to 53.6) | 41.2 (29.2 to 57) | 91.1 (63.4 to 129.3) |
|  |  | Lorestan | 73.5 (50 to 105.8) | 176.4 (101.6 to 275.8) | 14.6 (9.5 to 21) | 45.7 (31.1 to 66.3) | 68.6 (39.2 to 107.5) | 2 (0.9 to 4.2) | 228.8 (159.1 to 296.5) | 1479.6 (1048.2 to 1986.7) | 214.1 (138.5 to 301) | 39.4 (22.4 to 68.5) | 6.7 (3.7 to 10.6) | 434.4 (125.4 to 857.7) | 203.8 (99.4 to 392.1) | 27 (16.1 to 45.1) | 325.2 (223.7 to 454.4) | 234.4 (132.5 to 441.7) | 26 (19.1 to 33.1) | 185.2 (124.5 to 261.6) | 474.1 (302.9 to 698.9) | 42.7 (26.1 to 61.9) | 30.9 (22 to 42.9) | 71.3 (48.7 to 101.3) |
|  |  | Markazi | 88.9 (56 to 133) | 177.3 (105.4 to 283.3) | 17.9 (11.9 to 26.2) | 47.7 (32 to 67.7) | 68.8 (40.6 to 109.7) | 2.1 (0.8 to 4.2) | 218.3 (150.2 to 290.2) | 1678.5 (1212.5 to 2260.4) | 231.6 (149.9 to 329.2) | 41.3 (23.6 to 70.8) | 8.7 (4.7 to 14.1) | 475.6 (158 to 935.5) | 214 (104.3 to 425) | 27.8 (15.8 to 47.2) | 343.8 (238.2 to 491.5) | 236.3 (134.1 to 441.7) | 28 (20.6 to 36.2) | 192.7 (127.7 to 276.2) | 486.6 (310.8 to 712) | 52.6 (31.7 to 75.4) | 42.3 (29.7 to 58.5) | 90.3 (62.5 to 127.3) |
|  |  | Mazandaran | 91.5 (58.5 to 138.5) | 178.1 (105.5 to 286.7) | 16.6 (10.8 to 24.6) | 45.9 (30.6 to 65.7) | 74.2 (42.9 to 117.5) | 2.1 (0.9 to 4.5) | 223.1 (154.1 to 306) | 1654.5 (1156.4 to 2284.5) | 243 (159.1 to 345.5) | 33.9 (18.6 to 56.2) | 8.8 (5.1 to 14.2) | 484.6 (149.9 to 981.8) | 176.2 (86.3 to 328.9) | 24.1 (14.3 to 39.6) | 322.7 (223.8 to 456.3) | 233.7 (129 to 457) | 29.5 (21.7 to 38.5) | 194.1 (131.8 to 281.1) | 495.2 (317.9 to 723.4) | 36.9 (21.3 to 54.1) | 45.3 (32 to 62.5) | 91.5 (62.5 to 130.6) |
|  |  | North Khorasan | 98.7 (61.5 to 143.8) | 174.3 (105.7 to 273.6) | 19.2 (12.2 to 28.5) | 46.8 (31.4 to 65.7) | 66.1 (40.7 to 102.7) | 1.9 (0.8 to 3.8) | 233.6 (161.8 to 310) | 1582.9 (1122.7 to 2130.9) | 213.5 (140.8 to 300.2) | 51.2 (28.2 to 90.9) | 7.6 (4.5 to 12) | 447.6 (154.7 to 871.1) | 283.3 (134.1 to 540.6) | 32.8 (18.4 to 55) | 364.4 (251.4 to 529.7) | 235.4 (132.8 to 450.3) | 26.5 (19.7 to 34) | 185.5 (126.7 to 263.8) | 473.4 (298.3 to 688.4) | 39.9 (24.8 to 58.1) | 42.2 (29.5 to 57.1) | 92.8 (63.3 to 130.6) |
|  |  | Qazvin | 95.5 (61.6 to 143) | 172.8 (99.8 to 282) | 16.1 (10.5 to 22.9) | 48.9 (33.7 to 69.2) | 68 (38.9 to 108.3) | 2 (0.8 to 3.9) | 229.3 (156.2 to 299.1) | 1529.1 (1089.1 to 2076.9) | 227.5 (144 to 324.5) | 41.2 (22.5 to 68.4) | 6.9 (4.1 to 11.2) | 457.6 (148.8 to 945.1) | 211.1 (96.2 to 407.6) | 27.1 (15.3 to 44.4) | 334.3 (225.8 to 479.2) | 231.6 (126.5 to 449.8) | 27.6 (20.2 to 35.9) | 190.5 (129.2 to 279.4) | 483.4 (315.2 to 705) | 52.6 (32 to 76.3) | 35.8 (24.9 to 48.8) | 85.6 (59.3 to 122.6) |
|  |  | Qom | 59.5 (37.8 to 90.5) | 175.3 (102.6 to 285.4) | 17.5 (11.4 to 25.9) | 47.5 (32.4 to 65.9) | 67.7 (38.8 to 104.9) | 2 (0.9 to 4) | 233.8 (160.7 to 313.9) | 1621.5 (1146.8 to 2173.1) | 218.5 (139.6 to 314.1) | 39.6 (22.7 to 69) | 9.7 (5.6 to 14.9) | 456 (138 to 908.5) | 198 (97.1 to 385) | 26.2 (15.8 to 42.5) | 332.6 (231.7 to 462.8) | 228.2 (126.6 to 434) | 26.8 (19.5 to 34.2) | 189.5 (126.5 to 270.3) | 481.5 (312.7 to 694.7) | 38.7 (24.1 to 57.4) | 27.3 (18.8 to 37.8) | 98.6 (66.7 to 141.3) |
|  |  | Semnan | 90.1 (56.3 to 134.1) | 171.1 (99.6 to 277.6) | 18.2 (12.1 to 26.5) | 48.2 (32.9 to 68.5) | 66 (37.8 to 103.9) | 2 (0.7 to 4.4) | 239.2 (169.2 to 316.5) | 1676.5 (1200.5 to 2242.7) | 237.4 (151.9 to 342.1) | 40.4 (22.4 to 68.6) | 7 (4 to 11.1) | 483 (155.8 to 969) | 214.1 (107.7 to 396.3) | 26.8 (16.1 to 42.8) | 334.9 (238.2 to 478.2) | 231.6 (129.1 to 438) | 29.9 (21.5 to 38.5) | 182.8 (124.9 to 259.1) | 490.7 (317.7 to 714.9) | 45.5 (26.7 to 66.1) | 38.6 (27.5 to 52.9) | 84 (58 to 119.8) |
|  |  | Sistan and Baluchistan | 82.5 (53.5 to 125.4) | 184.6 (109.3 to 296.2) | 17.5 (11.6 to 24.9) | 56.4 (40.3 to 78.8) | 67.7 (40 to 102.7) | 2.2 (1.1 to 4) | 269 (187.2 to 353) | 1520.7 (1063.4 to 2055.4) | 193.2 (125.3 to 273.6) | 71.9 (42.4 to 117.3) | 5 (3.1 to 7.7) | 434.6 (150 to 883.4) | 462.2 (250.1 to 825.9) | 46.1 (27.2 to 77.8) | 406 (283.6 to 583.5) | 230.5 (128.4 to 407) | 32.1 (23.7 to 41.1) | 184.5 (126.6 to 261.6) | 456.1 (294.9 to 667.3) | 42.3 (26.2 to 60.1) | 44.9 (31.7 to 61.6) | 83 (58.8 to 116) |
|  |  | South Khorasan | 104 (67.2 to 159.1) | 191.9 (115.3 to 314.8) | 20.2 (13.3 to 29.2) | 49.5 (33.9 to 70.3) | 66.1 (38.8 to 104.4) | 1.9 (0.7 to 4.2) | 253.4 (175.3 to 338.8) | 1632 (1114.1 to 2146.4) | 223.4 (143 to 314.2) | 46.3 (26.1 to 79.2) | 7.5 (4.2 to 12.2) | 454.8 (139.9 to 887) | 247 (121.3 to 497) | 31 (17.6 to 52.3) | 347.6 (241.7 to 506.8) | 230.1 (129 to 416.4) | 26.5 (19.5 to 34.1) | 189 (128.5 to 269.2) | 476.6 (305.6 to 699.5) | 44.1 (27.5 to 64.6) | 42.2 (29.9 to 57.5) | 84.1 (58.5 to 118.8) |
|  |  | Tehran | 70.8 (48.4 to 101.5) | 175.4 (100.8 to 288.9) | 16.7 (10.7 to 24.3) | 47.8 (33.2 to 67.6) | 71.2 (40.7 to 112.3) | 2.5 (1 to 5) | 224.5 (154.4 to 301.9) | 1608.9 (1140.1 to 2185.4) | 283.2 (187.1 to 410.5) | 29.3 (16.6 to 48.7) | 10.2 (6 to 16) | 485.5 (140.4 to 930) | 148.7 (76.5 to 269.7) | 22.2 (13.7 to 35.3) | 305.7 (212.7 to 430.1) | 232.8 (128.2 to 462.8) | 30.1 (22.2 to 39.1) | 184.9 (123.3 to 264.7) | 496.6 (321.8 to 717.7) | 73.9 (47.5 to 105) | 23.3 (16.4 to 32.5) | 109 (71.9 to 158.6) |
|  |  | West Azarbayejan | 78.8 (50.4 to 118.5) | 177.5 (102.8 to 291.7) | 15.6 (10.3 to 22.8) | 47.7 (32 to 68.1) | 68 (41.4 to 107.7) | 2 (0.8 to 4) | 223 (150.8 to 293.3) | 1594 (1136.9 to 2104.5) | 223.5 (145 to 318.2) | 46.9 (25.8 to 80.9) | 8 (4.5 to 12.9) | 465.1 (154.8 to 914.5) | 244.6 (114.3 to 493.8) | 30.8 (17.7 to 53.8) | 344.1 (238.4 to 492.5) | 231.6 (130.6 to 442.9) | 29.9 (22.1 to 38.2) | 190.9 (126.3 to 270.6) | 476.2 (306.6 to 697.6) | 30.8 (19.1 to 44.6) | 41 (29.4 to 55.9) | 85.1 (59 to 119.5) |
|  |  | Yazd | 65.3 (39.3 to 101.6) | 174.5 (101.9 to 285.6) | 17.3 (11.3 to 25.3) | 46.9 (32 to 65.7) | 70.2 (40.8 to 110.1) | 2.1 (0.8 to 3.9) | 283.3 (199.8 to 382.2) | 1516.9 (1080.2 to 2074) | 223.1 (146.5 to 320.6) | 39.3 (22.7 to 65) | 9.6 (5.4 to 15.1) | 469.1 (136.3 to 931.3) | 225.1 (115.6 to 422) | 26.8 (16.1 to 44.1) | 342.7 (235.4 to 490.3) | 229.6 (128.8 to 415.2) | 26.9 (19.9 to 34.6) | 179.8 (121.9 to 256.7) | 484.4 (312.3 to 699) | 43.7 (26.2 to 64.8) | 41.9 (29.2 to 57.7) | 91.2 (63.9 to 129.1) |
|  |  | Zanjan | 98.6 (64 to 151.3) | 172.2 (98.7 to 287.5) | 15 (9.7 to 21.7) | 47.2 (32.1 to 66.1) | 70 (40.9 to 111.4) | 2 (0.8 to 3.8) | 221 (151 to 295) | 1601.9 (1141.7 to 2198.2) | 218.1 (141.1 to 312.3) | 42.3 (23 to 75.4) | 7.3 (4 to 12.3) | 443.7 (139.4 to 871.5) | 219.7 (99.5 to 479.1) | 28.3 (15.8 to 50.4) | 333.1 (228.2 to 478.9) | 235.4 (129.8 to 448.4) | 26.3 (19.5 to 34.1) | 187.6 (126.5 to 268.9) | 476.1 (305.4 to 693) | 37.3 (23.3 to 54.6) | 39.1 (27.2 to 53.8) | 83.4 (57.8 to 118.3) |
| YLLs (Years of Life Lost) | Both Sexes | Province | Causes |  |  |  |  |  |  |  |  |  |  |  |  |  |  |  |  |  |  |  |  |  |
|  |  |  | Cardiovascular diseases | Chronic respiratory diseases | Diabetes and kidney diseases | Digestive diseases | Enteric infections | HIV/AIDS and sexually transmitted infections | Maternal and neonatal disorders | Mental disorders | Musculoskeletal disorders | Neglected tropical diseases and malaria | Neoplasms | Neurological disorders | Nutritional deficiencies | Other infectious diseases | Other non-communicable diseases | Respiratory infections and tuberculosis | Self-harm and interpersonal violence | Skin and subcutaneous diseases | Substance use disorders | Transport injuries | Unintentional injuries | |
|  |  | Alborz | 150.4 (123 to 184.1) | 22.4 (17.7 to 29.1) | 35 (29.6 to 42.4) | 45.6 (35.7 to 60.8) | 47.8 (33.1 to 67.7) | 43.8 (20.8 to 84.3) | 419.9 (318 to 532.9) | 0 (0 to 0) | 8.1 (5.6 to 11.1) | 3.9 (1.5 to 18.4) | 338.2 (258.1 to 408.2) | 34.7 (26.3 to 51.3) | 3.9 (2.9 to 4.9) | 47.9 (32.2 to 87) | 481.7 (389.7 to 603.7) | 436.3 (372 to 491.1) | 338.7 (259.4 to 418.1) | 1.6 (1.2 to 2.2) | 37.5 (21 to 58.7) | 512.6 (417.5 to 623.3) | 389.2 (328.7 to 447.6) | |
|  |  | Ardebil | 211.9 (172.9 to 253.1) | 26.2 (20.2 to 35.9) | 45.4 (36.3 to 53.9) | 50 (38.6 to 68.1) | 66.9 (42 to 98.8) | 44.8 (17.7 to 95) | 1005.5 (815.4 to 1201.8) | 0 (0 to 0) | 5.2 (3.9 to 6.8) | 15.7 (1.6 to 130) | 384.9 (254.2 to 515.5) | 58.1 (43.1 to 72.2) | 4.8 (3.3 to 6.5) | 69.5 (41.6 to 156.7) | 1048.8 (851.4 to 1287.6) | 276.8 (230.8 to 329.2) | 267.8 (218.9 to 323.7) | 0.9 (0.6 to 1.2) | 12.1 (7.5 to 19) | 592.7 (483.4 to 728.6) | 371.9 (297.5 to 488.1) | |
|  |  | Bushehr | 124.8 (99 to 147.4) | 16.7 (13.1 to 22.9) | 30.7 (25.8 to 36.6) | 50.6 (36.3 to 70.9) | 60.2 (41.8 to 84.7) | 47.4 (23.8 to 93.4) | 923.3 (766.2 to 1101.6) | 0 (0 to 0) | 4.7 (3.7 to 6.1) | 4.5 (2 to 12.1) | 285.1 (193.6 to 363.2) | 32.4 (24.6 to 45.3) | 17.8 (12.4 to 23) | 41.1 (33 to 55.1) | 771.6 (643 to 967.7) | 274.5 (233.3 to 317.6) | 208.8 (174.9 to 247.8) | 5.2 (3.6 to 7.8) | 10.8 (6.6 to 18) | 700.7 (590.4 to 813.6) | 319.3 (269.7 to 394.8) | |
|  |  | Chahar Mahaal and Bakhtiari | 121.9 (98.3 to 147.5) | 16 (11.9 to 23.2) | 21.3 (17.6 to 25.9) | 22.3 (16.8 to 30.1) | 46.1 (25 to 73.3) | 43.2 (15.6 to 92.5) | 552.7 (424.9 to 694.4) | 0 (0 to 0) | 1.5 (1 to 3.1) | 1.4 (0.7 to 2.4) | 255.7 (163 to 338.9) | 38.8 (28.4 to 49.7) | 1.1 (0.7 to 1.5) | 30.1 (20.1 to 56.9) | 567.1 (448 to 732.7) | 181.4 (149.2 to 214.5) | 172 (138.6 to 206.1) | 0.1 (0.1 to 0.1) | 6.2 (3.6 to 10.3) | 550.7 (451.4 to 647.4) | 289.9 (238.6 to 362) | |
|  |  | East Azarbayejan | 201.5 (168.2 to 244.5) | 48.9 (38.4 to 65.7) | 57.7 (46.7 to 67.1) | 68.8 (53.6 to 91.5) | 58.3 (39.3 to 80.1) | 40.8 (18.7 to 79.6) | 584.4 (447.5 to 747.6) | 0 (0 to 0.1) | 8.3 (6.3 to 11) | 7.1 (2.1 to 41.1) | 436.1 (291.8 to 564.4) | 83.6 (39.8 to 112.8) | 11 (7.9 to 14.8) | 64.8 (44 to 123.9) | 766.2 (626.9 to 972.4) | 313.1 (265.8 to 359.1) | 246.8 (198.2 to 304.8) | 3.9 (2.8 to 5.4) | 27.4 (16.6 to 44.8) | 866.6 (722.5 to 1030.7) | 399 (339 to 478.8) | |
|  |  | Fars | 228.8 (189.8 to 268.2) | 32.7 (26.1 to 42.2) | 54.3 (45.1 to 64.2) | 91.6 (65.3 to 126.1) | 59.9 (40.9 to 84.1) | 60.5 (36.8 to 104.4) | 974.1 (748.5 to 1239.6) | 0 (0 to 0.1) | 8.4 (6.3 to 11.4) | 9.8 (2.4 to 63.8) | 434.9 (306.4 to 568.5) | 60.6 (36.7 to 79.3) | 3.7 (2.8 to 4.6) | 73.7 (54.1 to 125.2) | 1000.1 (813.7 to 1238) | 356.3 (303.4 to 418.4) | 402.5 (325.3 to 500.6) | 2.6 (1.9 to 3.8) | 40.6 (23.6 to 63.9) | 1314.4 (1118.3 to 1503.8) | 495.9 (423.8 to 579.8) | |
|  |  | Gilan | 200.7 (164.3 to 240.6) | 23.8 (19.1 to 30.1) | 35.2 (28.9 to 42.3) | 34.4 (27.1 to 45.1) | 38.5 (24.7 to 57) | 32 (12.5 to 64.9) | 245 (169.5 to 336.8) | 0 (0 to 0) | 5.4 (4.2 to 7.1) | 1.4 (0.8 to 2.1) | 282.3 (215.5 to 350.7) | 34.4 (27.4 to 42.9) | 1.8 (1.4 to 2.2) | 34.3 (22.2 to 67.3) | 365.8 (293.2 to 454.1) | 254.4 (213 to 291.2) | 250.9 (205.3 to 308.2) | 1.1 (0.8 to 1.5) | 17.4 (9.8 to 28.3) | 856.5 (709.2 to 1009.7) | 403.8 (340.4 to 494.9) | |
|  |  | Golestan | 237.1 (198.2 to 286.1) | 40 (31.7 to 50.8) | 53.7 (44.9 to 63.1) | 94.6 (63.3 to 130.9) | 75.8 (51.6 to 104.2) | 47.6 (21 to 94) | 955.2 (794.7 to 1121.9) | 0 (0 to 0) | 5.9 (4.5 to 8) | 4.9 (2.4 to 9.1) | 350.9 (280 to 441.6) | 69.7 (42.4 to 90.3) | 9.3 (6.9 to 12) | 104.7 (65.2 to 196.2) | 937.2 (795.6 to 1144.3) | 351.6 (297.5 to 403.8) | 293.3 (238.5 to 359) | 4.3 (3.1 to 5.9) | 13 (7.7 to 19.9) | 1090.8 (927.8 to 1250) | 433.6 (361.8 to 539.4) | |
|  |  | Hamadan | 259.4 (215.7 to 307) | 38.4 (30 to 50.9) | 50.4 (41.8 to 59.9) | 89 (64.8 to 123.8) | 79.5 (56.8 to 109.4) | 45.1 (20.3 to 92) | 1102.7 (932 to 1310.6) | 0 (0 to 0) | 7.7 (5.5 to 10.5) | 5.1 (2.5 to 9.8) | 441.9 (309.5 to 571) | 70.2 (48.2 to 91.1) | 6.1 (4.5 to 8) | 96.9 (70.6 to 150.4) | 954.1 (802.1 to 1175) | 301.6 (255.8 to 354.3) | 325.8 (249.6 to 402.7) | 2.6 (1.7 to 3.8) | 52.8 (30.6 to 82) | 1119.1 (961.3 to 1294.5) | 462.3 (392.5 to 559.7) | |
|  |  | Hormozgan | 223.8 (184.4 to 261.4) | 25.5 (20.4 to 34.4) | 59 (40.5 to 72) | 85.4 (57.7 to 116) | 96.1 (67 to 130) | 68 (41.5 to 117.7) | 1089.4 (918.6 to 1291.7) | 0 (0 to 0) | 9.7 (4.9 to 12.5) | 7.8 (3.1 to 20.1) | 287.4 (231 to 351.6) | 53.9 (38.9 to 67.9) | 13.2 (10.1 to 16.1) | 74.9 (55.3 to 131) | 897.4 (752.7 to 1101.4) | 364.2 (308 to 415.3) | 254.5 (203.9 to 315.3) | 3.3 (2.3 to 4.8) | 19.5 (11.5 to 31.3) | 1093.5 (903 to 1272.2) | 469.2 (393.2 to 568.9) | |
|  |  | Ilam | 166.1 (129.2 to 202.6) | 17.5 (13.5 to 24.2) | 50.7 (36.3 to 62.4) | 46.3 (33 to 65.3) | 63.5 (40.5 to 90.7) | 46.5 (18.6 to 100.1) | 1038.9 (747 to 1359.1) | 0 (0 to 0) | 4.6 (3.5 to 6.1) | 4.6 (1.7 to 16.7) | 304.6 (208.3 to 416.8) | 34.3 (24.1 to 48.9) | 6.6 (4.1 to 9) | 45 (30.7 to 71.5) | 869.6 (617.4 to 1184.7) | 313.6 (255.9 to 371.3) | 480 (345.3 to 572.3) | 1 (0.6 to 1.4) | 5.8 (3.2 to 10.1) | 702.9 (558.8 to 863.5) | 380.1 (309.7 to 470.8) | |
|  |  | Isfahan | 171.6 (138.4 to 200.7) | 31 (24.3 to 40.2) | 47.5 (38.7 to 55.3) | 56.5 (45.1 to 76.7) | 49.1 (33.5 to 71) | 34.7 (14.8 to 70.8) | 677.4 (499.4 to 890.7) | 0 (0 to 0) | 9.8 (5.6 to 13.4) | 3.1 (1.7 to 5.4) | 389.2 (253.7 to 494.5) | 52.6 (37.2 to 65.9) | 4.3 (3.1 to 5.7) | 56.4 (40 to 95.5) | 760.8 (596.5 to 963.1) | 332.6 (276 to 397) | 199.7 (162.1 to 248.7) | 3 (2.1 to 4.1) | 19.7 (10.6 to 33.1) | 756.4 (629.6 to 910.4) | 434.3 (372.5 to 506) | |
|  |  | Kerman | 215.4 (181.9 to 250.8) | 65.3 (51.5 to 83.2) | 55.4 (43.1 to 66) | 83.5 (59.5 to 111) | 95.6 (70.3 to 128) | 51 (26.4 to 98.3) | 1329 (1128.9 to 1571.1) | 0 (0 to 0) | 11.2 (6.7 to 15.2) | 8.1 (3.2 to 12.5) | 381.1 (269.1 to 490.6) | 49.3 (39.5 to 60.3) | 9.5 (7.1 to 12.1) | 103.7 (61 to 246.8) | 978.1 (803.9 to 1217.2) | 396.5 (338.2 to 452.4) | 277.5 (225.5 to 338) | 2.8 (2.1 to 3.9) | 21.6 (12.9 to 34.8) | 1157 (994.7 to 1305.5) | 419.3 (364.3 to 511.6) | |
|  |  | Kermanshah | 199.4 (162.4 to 236.4) | 35.6 (28.6 to 46.2) | 48.2 (40.4 to 57.7) | 74.8 (54.4 to 105.4) | 66.5 (46.1 to 93.6) | 70.5 (45.6 to 116) | 1195.1 (997.9 to 1421.6) | 0 (0 to 0) | 9 (6.4 to 12) | 3.7 (1.8 to 7.9) | 392.4 (266 to 512.4) | 36.1 (26.3 to 55.1) | 10.9 (7.8 to 13.3) | 92.2 (52.5 to 225.1) | 884.1 (717.7 to 1114.6) | 355 (299.9 to 404.3) | 441.7 (343.1 to 527) | 2.8 (1.9 to 4.4) | 47.6 (27.6 to 78.2) | 879 (726.8 to 1046.8) | 421.6 (361.2 to 493.5) | |
|  |  | Khorasan-e-Razavi | 174.4 (134.7 to 214.2) | 45.7 (35 to 65.1) | 51.2 (41.3 to 63.2) | 79.3 (57.1 to 116.1) | 73.8 (49.5 to 104.8) | 44.9 (20.6 to 86.4) | 971.5 (719.1 to 1268.1) | 0 (0 to 0) | 7.6 (5.6 to 10.5) | 6.4 (2.8 to 15) | 414.6 (264.3 to 570.5) | 59.1 (40.7 to 77.4) | 8.9 (5.9 to 12.1) | 77 (53.8 to 133.1) | 896.6 (669 to 1205.1) | 392.3 (325 to 454.4) | 227 (188.7 to 279.1) | 2.9 (2 to 4.2) | 30.1 (18.2 to 45.2) | 917.5 (745.4 to 1097.3) | 417 (345.5 to 489.9) | |
|  |  | Khuzestan | 217.6 (178.2 to 258.7) | 31.2 (24.8 to 40.8) | 56.7 (47.8 to 66.7) | 73.4 (56.3 to 102) | 70.7 (50.4 to 97.5) | 53.6 (27.6 to 103.4) | 975.8 (785 to 1192.1) | 0 (0 to 0.1) | 6.9 (5.1 to 9.3) | 3.8 (2.2 to 6.1) | 371.6 (285 to 459.7) | 50.9 (39.7 to 62.4) | 10.7 (7.6 to 14.6) | 90.4 (58 to 186.8) | 1053.6 (862.9 to 1291.3) | 419.9 (355.4 to 479.4) | 309.3 (260.1 to 368.9) | 5.4 (4 to 7.4) | 21.7 (12.7 to 34.7) | 819.4 (691.9 to 956) | 613.6 (542.8 to 693.6) | |
|  |  | Kohgiluyeh and Boyer-Ahmad | 271.7 (223.7 to 320.5) | 26.9 (20.8 to 35.8) | 46.6 (39.3 to 54.6) | 64.8 (52.6 to 85.2) | 70.8 (48.6 to 98.7) | 67.1 (28 to 137.2) | 919 (734.3 to 1136.8) | 0 (0 to 0) | 8.9 (6.5 to 11.5) | 9.7 (3.1 to 42.7) | 440.7 (300 to 571.2) | 56.8 (40.9 to 71.3) | 3.2 (2.2 to 4.2) | 88.5 (65.9 to 117.3) | 929.6 (774.5 to 1162.7) | 282.9 (230.8 to 345.6) | 386 (298.4 to 463.9) | 1.8 (1.4 to 2.5) | 11.7 (7.3 to 18.6) | 1042 (864.5 to 1204.1) | 567.4 (488.2 to 646.3) | |
|  |  | Kurdistan | 173.9 (141.7 to 210.9) | 30.2 (23 to 42.8) | 39.8 (32.5 to 47.9) | 65.7 (47.9 to 91.2) | 74.4 (52.5 to 102.5) | 47.1 (21.7 to 95.1) | 1265 (1057.7 to 1496.2) | 0 (0 to 0) | 7.8 (4.9 to 10.8) | 3.8 (1.9 to 5.8) | 375.8 (242.9 to 510.3) | 57.9 (39.6 to 74.3) | 5.2 (3.7 to 6.7) | 62 (45.6 to 95.1) | 855.9 (680.3 to 1095.4) | 270.9 (225.9 to 317.5) | 275.6 (233 to 330.9) | 3 (1.9 to 4.9) | 10.5 (6.1 to 17.1) | 924.1 (783.1 to 1072.1) | 419 (348.7 to 506.1) | |
|  |  | Lorestan | 112.8 (89.2 to 133.9) | 13.7 (10.7 to 19) | 24.8 (20.2 to 29.9) | 33.9 (25 to 47.8) | 46.5 (30.8 to 70.6) | 51.1 (26.1 to 94.8) | 466.4 (358.3 to 585.2) | 0 (0 to 0) | 3.5 (2.6 to 4.7) | 2.5 (1.3 to 3.7) | 247.7 (172.3 to 321.5) | 27 (19.5 to 40.1) | 18.3 (13.5 to 23.2) | 46 (30.3 to 87) | 398.7 (307.5 to 530.5) | 313.2 (259.8 to 366.2) | 248.4 (171.1 to 306.4) | 0.7 (0.4 to 1) | 22.3 (12.5 to 36.4) | 489.9 (404.7 to 578.4) | 245.2 (196.7 to 329.5) | |
|  |  | Markazi | 156.8 (125.6 to 187.3) | 33 (26.7 to 43.5) | 47.6 (39.5 to 56.9) | 63.9 (49.4 to 87.7) | 52.1 (34.2 to 75.5) | 39 (17.2 to 78) | 820.1 (682.8 to 987.8) | 0 (0 to 0) | 6.7 (5 to 9.1) | 2.1 (1.2 to 3.2) | 405.2 (257.5 to 530.2) | 55.6 (38.3 to 71.1) | 3.7 (2.8 to 4.6) | 55 (44.1 to 71.5) | 658.1 (530.1 to 832.2) | 387.3 (320.9 to 451.3) | 195.4 (156.2 to 242.1) | 2.9 (2.1 to 4) | 22.6 (12.5 to 38.6) | 909.8 (759.1 to 1043.4) | 370.3 (318.2 to 436.9) | |
|  |  | Mazandaran | 167.9 (133.8 to 202.9) | 21.1 (16.8 to 27.9) | 40.2 (34 to 48) | 37.4 (29.5 to 49.8) | 41.9 (27.7 to 62.9) | 34.9 (13.9 to 72.1) | 362.9 (266.4 to 468.8) | 0 (0 to 0) | 7 (5 to 9.3) | 2.6 (1.4 to 4.7) | 320.8 (224.1 to 401.7) | 36.2 (29.8 to 44.7) | 5.7 (4.3 to 7.3) | 38.4 (28.2 to 62.8) | 541.9 (421.2 to 667) | 261.5 (221.4 to 301.8) | 215.9 (180.4 to 260.5) | 2.2 (1.6 to 3) | 10.2 (5.9 to 16.1) | 814.8 (684.8 to 951.6) | 414.3 (351 to 496.2) | |
|  |  | North Khorasan | 217.2 (177.4 to 257.4) | 45.1 (35.8 to 62.3) | 57.5 (48.2 to 68.2) | 87.5 (65.1 to 118.6) | 83.4 (59.8 to 115.3) | 47.8 (21.6 to 97.2) | 887.4 (746.7 to 1056.8) | 0.3 (0 to 0.9) | 6.9 (5.1 to 9.2) | 15.4 (3.3 to 91.8) | 412 (288 to 533.4) | 76.9 (42.9 to 104.1) | 10.4 (7.7 to 13.3) | 82.4 (59.3 to 127.6) | 868.8 (721.6 to 1067.1) | 477 (390.5 to 554.1) | 261.6 (214.2 to 309.1) | 3.2 (2.3 to 4.5) | 17.1 (10.6 to 27.4) | 1053.9 (884.4 to 1202.5) | 453.3 (389.7 to 524) | |
|  |  | Qazvin | 114.8 (91.3 to 138.7) | 21.7 (17.2 to 29.1) | 30.5 (24.5 to 36.8) | 78.7 (50.8 to 112.6) | 52.6 (34.6 to 75.3) | 40 (16.6 to 82) | 651.2 (515.2 to 805.4) | 0 (0 to 0) | 4.1 (3.1 to 5.6) | 1.2 (0.6 to 2.4) | 311.1 (201.7 to 405.9) | 37.8 (29.8 to 48.4) | 2.9 (2.1 to 3.7) | 39.8 (30.7 to 52.8) | 587.3 (466.5 to 745.7) | 352.2 (297.4 to 405.3) | 166.1 (136.7 to 202.3) | 3.4 (2.4 to 5.1) | 28.8 (15.9 to 48) | 645.9 (520.1 to 774.3) | 335.1 (279.1 to 399.9) | |
|  |  | Qom | 106.7 (80.8 to 135.7) | 26.2 (21.3 to 33.4) | 39.1 (30.5 to 47.4) | 58.1 (41.9 to 79.7) | 49.7 (31.8 to 73.5) | 52.7 (29.4 to 93.7) | 523.7 (399.2 to 659.7) | 0 (0 to 0) | 4 (3 to 5.6) | 5.4 (1.4 to 35) | 367.2 (228.9 to 471.3) | 36.6 (27.9 to 49.5) | 2.2 (1.5 to 2.9) | 41.6 (29.3 to 68.1) | 568.4 (447.3 to 717.2) | 340.7 (279.9 to 406.9) | 166.6 (132.4 to 214.1) | 11.8 (8.5 to 16.1) | 11 (6.4 to 18.3) | 368.6 (289.2 to 492.6) | 363.1 (274.2 to 437.3) | |
|  |  | Semnan | 113.7 (88.9 to 141.2) | 24.8 (18.6 to 33.2) | 39.3 (30.8 to 47.7) | 47.2 (33.4 to 66.7) | 52.5 (32 to 80.6) | 36.4 (13.3 to 82.8) | 750.2 (514.2 to 1015.6) | 0 (0 to 0) | 3 (2.2 to 4.5) | 2.2 (1.2 to 3.3) | 289 (202.6 to 386.3) | 52.9 (33.8 to 73.5) | 4.6 (3 to 6.4) | 33.6 (23.6 to 47.4) | 673.9 (472.9 to 928.1) | 479.9 (407.2 to 553.1) | 156.8 (128.8 to 190.4) | 2.8 (1.9 to 4.1) | 16.5 (9.6 to 27.8) | 677.5 (550.5 to 826.9) | 294.9 (231.9 to 370.5) | |
|  |  | Sistan and Baluchistan | 344.4 (289.9 to 406.8) | 96.5 (67.1 to 129.4) | 104.4 (51.1 to 127.5) | 167.8 (102.3 to 224.4) | 162.9 (112 to 226.3) | 82.9 (39.6 to 156.3) | 1840.8 (1534.3 to 2151.7) | 0 (0 to 0) | 9.3 (6.2 to 12.5) | 30.1 (4.4 to 49.4) | 370 (295.6 to 468.7) | 77.9 (63.2 to 95.1) | 28.4 (21 to 36.8) | 255.8 (125.9 to 525.5) | 1248.3 (1020.2 to 1610.2) | 521.7 (448.8 to 592.8) | 309.1 (253 to 377.1) | 2.2 (1.6 to 3.2) | 27.1 (16.2 to 40.5) | 1244.5 (1070.3 to 1414.1) | 623.5 (531.3 to 765.5) | |
|  |  | South Khorasan | 131.8 (104.6 to 154.6) | 43.8 (34.7 to 59.4) | 45 (35.6 to 54.3) | 77.7 (50.6 to 111.9) | 68.4 (47.3 to 93.9) | 40.9 (15.7 to 86.2) | 1021.6 (844.3 to 1225.9) | 0 (0 to 0) | 4.4 (3.4 to 6.2) | 5.7 (2.2 to 9) | 334.9 (206.5 to 456.5) | 45 (35.4 to 54.3) | 15.7 (10.8 to 19.7) | 84.5 (51.2 to 178.6) | 727 (565.4 to 950.8) | 387.5 (324.1 to 450.6) | 147.4 (125.7 to 173.4) | 2.8 (2.1 to 4.1) | 17.1 (10.4 to 26.9) | 792.9 (661.6 to 912.8) | 355.6 (306.3 to 427.3) | |
|  |  | Tehran | 129.2 (102.7 to 165) | 19.7 (15.5 to 27) | 46.2 (37.5 to 54.1) | 49.6 (38.5 to 65.2) | 38.7 (23.5 to 60.2) | 50 (26.7 to 100.3) | 462.4 (273.5 to 695.8) | 0 (0 to 0) | 15.9 (6.3 to 21.6) | 1.5 (0.8 to 5) | 356.4 (255.5 to 431.4) | 61.5 (49.8 to 79.4) | 3.8 (2.7 to 5.3) | 50.4 (34 to 82.7) | 587.6 (414.4 to 776.5) | 294.2 (254.1 to 337.3) | 139 (113.7 to 186.2) | 1.6 (1.2 to 2.1) | 64.1 (37.5 to 102.3) | 120.5 (89.1 to 218.1) | 301.5 (228.3 to 367.4) | |
|  |  | West Azarbayejan | 232.6 (186.1 to 272.9) | 47.4 (38.4 to 62.6) | 50.3 (41.7 to 59.9) | 73.5 (57.3 to 99.3) | 79 (55 to 107.5) | 45 (20 to 90.4) | 1044.6 (865.9 to 1236.8) | 0 (0 to 0) | 7.3 (5.7 to 9.6) | 3.6 (2 to 5.2) | 448.3 (291.4 to 598.4) | 70.7 (46.5 to 93.1) | 10.5 (7.4 to 13.7) | 88.7 (53.4 to 193.3) | 1001.3 (823.6 to 1244.2) | 340 (287.8 to 405.3) | 397.7 (328.6 to 474.7) | 2.3 (1.7 to 3.2) | 8.4 (5 to 12.7) | 901.6 (753.5 to 1052.6) | 443.5 (373.6 to 551.5) | |
|  |  | Yazd | 136.3 (106.8 to 168) | 34.7 (28.1 to 43) | 47.3 (38.8 to 56.1) | 48.9 (39.3 to 65.8) | 58.6 (40.5 to 81.5) | 44.2 (21.1 to 86.6) | 936.7 (763.4 to 1139) | 0.1 (0 to 0.2) | 9.4 (5.6 to 12.3) | 2.2 (1.3 to 3.2) | 430.5 (267.3 to 548.3) | 57.5 (37.6 to 74.7) | 8 (6 to 10.3) | 51.3 (36.2 to 89.6) | 941 (776 to 1157.1) | 547.2 (472.3 to 616) | 142.5 (116.9 to 176.7) | 7.9 (5.8 to 10.8) | 18.2 (10.2 to 30.6) | 767.2 (618.1 to 931.4) | 371.9 (320.4 to 439.7) | |
|  |  | Zanjan | 140.1 (114.2 to 166) | 28.4 (22.7 to 37.7) | 32.3 (25.1 to 39.3) | 55.5 (39.5 to 78.3) | 69.5 (47.6 to 101.4) | 43.7 (18.4 to 87.7) | 1045.8 (847.6 to 1286.9) | 0.1 (0 to 0.2) | 3.8 (2.6 to 5.1) | 5.5 (2.3 to 8.7) | 297.3 (200.2 to 387) | 40.6 (32.2 to 51.4) | 3.5 (2.6 to 4.4) | 60.9 (44.9 to 92.8) | 725.6 (551.1 to 969.6) | 375.6 (316.7 to 430.4) | 150.7 (125.8 to 181.4) | 1.4 (0.9 to 2) | 13.6 (8.5 to 21.9) | 730.6 (622.5 to 845.3) | 354.8 (299.6 to 414.3) | |
| YLLs (Years of Life Lost) | Females | Province | Causes |  |  |  |  |  |  |  |  |  |  |  |  |  |  |  |  |  |  |  |  |  |
|  |  |  | Cardiovascular diseases | Chronic respiratory diseases | Diabetes and kidney diseases | Digestive diseases | Enteric infections | HIV/AIDS and sexually transmitted infections | Maternal and neonatal disorders | Mental disorders | Musculoskeletal disorders | Neglected tropical diseases and malaria | Neoplasms | Neurological disorders | Nutritional deficiencies | Other infectious diseases | Other non-communicable diseases | Respiratory infections and tuberculosis | Self-harm and interpersonal violence | Skin and subcutaneous diseases | Substance use disorders | Transport injuries | Unintentional injuries | |
|  |  | Alborz | 124.7 (98.4 to 154) | 20.5 (14.8 to 32) | 34.7 (27 to 44.2) | 42.7 (31.8 to 58.7) | 46.6 (30.8 to 69.1) | 39.6 (19.4 to 76.2) | 369.3 (268.4 to 485.5) | 0 (0 to 0) | 12.8 (8.1 to 18.2) | 2.9 (1.2 to 10.8) | 351.6 (220.7 to 441.5) | 27.1 (19.1 to 47.1) | 3.6 (2.5 to 5) | 43.7 (28.9 to 79.7) | 404.5 (308.4 to 576.8) | 365.5 (307.5 to 425.2) | 226.1 (171.4 to 288.6) | 1.1 (0.7 to 1.6) | 17.9 (9.8 to 32.2) | 296.7 (230 to 376.3) | 239.5 (203.1 to 282.5) | |
|  |  | Ardebil | 173.2 (138.7 to 207.2) | 26.6 (17.9 to 44.8) | 43.7 (33.7 to 54) | 50.2 (35.5 to 72.3) | 64.6 (35.8 to 98.8) | 45.9 (20.1 to 94) | 921.2 (732 to 1110.2) | 0 (0 to 0) | 7 (5 to 10.1) | 11.6 (1.9 to 81.6) | 338.1 (213 to 450) | 46.1 (30.2 to 65.3) | 4.1 (2.7 to 5.9) | 73.6 (40.7 to 170.5) | 978.1 (764.4 to 1222.1) | 242.8 (199.4 to 316.3) | 189.9 (147.2 to 234.4) | 0.7 (0.5 to 1.1) | 7.9 (4.3 to 13.2) | 312.4 (234.5 to 426.4) | 222.9 (184.8 to 288.6) | |
|  |  | Bushehr | 112 (84.6 to 135.7) | 18.6 (12.9 to 32.1) | 30.3 (24.4 to 37.2) | 51.4 (36.2 to 72.8) | 63.3 (39.1 to 95.6) | 44.2 (21.7 to 85) | 863 (694.7 to 1026.9) | 0 (0 to 0) | 7.3 (5.3 to 10.2) | 3.8 (1.9 to 8.5) | 281.1 (178.2 to 359) | 27.2 (18.4 to 40.8) | 16.9 (11.5 to 22.2) | 42 (32.4 to 57.8) | 747.3 (607.8 to 953.1) | 241.6 (200.7 to 292.2) | 201.3 (156.5 to 244.2) | 3.5 (2.3 to 5.6) | 6.2 (3.2 to 11) | 362.6 (297.7 to 425.9) | 212.3 (178.3 to 259.6) | |
|  |  | Chahar Mahaal and Bakhtiari | 105.3 (81.6 to 129.3) | 16 (10.6 to 29.4) | 20.2 (15.7 to 25.3) | 21.5 (15.9 to 28.9) | 40.5 (22.4 to 63.6) | 41 (15.2 to 87.9) | 513.4 (384.7 to 650) | 0 (0 to 0) | 2.2 (1.4 to 4.6) | 0.9 (0.4 to 2.1) | 231.6 (149.4 to 308.3) | 30.3 (17.6 to 43.9) | 1 (0.7 to 1.4) | 30.6 (19.3 to 57.7) | 538.7 (408.5 to 751.3) | 156.7 (129 to 203.3) | 126.5 (87.6 to 162.8) | 0.1 (0.1 to 0.2) | 3.2 (1.8 to 5.3) | 311.8 (244.7 to 368) | 159.7 (126.7 to 202.5) | |
|  |  | East Azarbayejan | 181.6 (144.7 to 224.2) | 54.5 (32.2 to 89.5) | 62.2 (48.2 to 77.3) | 73.4 (55 to 100.8) | 61.2 (38.5 to 92.7) | 40.8 (19.5 to 78.3) | 559.4 (428.6 to 718.6) | 0.1 (0 to 0.2) | 13.4 (9.3 to 19.5) | 6.2 (2.3 to 27.1) | 440 (276.9 to 564.8) | 76.4 (32.5 to 116.4) | 10.8 (7.5 to 15.2) | 65.1 (40.7 to 125.9) | 734 (573.9 to 953.7) | 286.5 (237.4 to 349.9) | 178.8 (136.6 to 226.5) | 2.8 (2 to 4.1) | 16.1 (8.9 to 27.8) | 490 (390.4 to 619.1) | 272.1 (230.3 to 338.1) | |
|  |  | Fars | 193.8 (152.1 to 235.4) | 32.8 (22.1 to 53.6) | 49.9 (40.3 to 62.9) | 85.2 (60.4 to 121.3) | 58.3 (36.2 to 85.6) | 54.4 (32 to 97.3) | 895.9 (683.3 to 1146.8) | 0 (0 to 0.1) | 13.4 (8.8 to 19.4) | 7.7 (2.4 to 40) | 392.3 (249.7 to 504.9) | 50 (29.7 to 69.5) | 3.5 (2.5 to 4.6) | 69.5 (47.5 to 125.7) | 882.5 (693.5 to 1157.5) | 311.6 (258.7 to 377.2) | 254.4 (198 to 322.1) | 1.4 (1 to 2.2) | 21.3 (11.2 to 40.5) | 674.9 (536.7 to 798.1) | 325.3 (278.8 to 387) | |
|  |  | Gilan | 137.3 (108.2 to 164.3) | 18.4 (12.9 to 29.4) | 30.8 (24.7 to 38) | 30 (22.4 to 41.1) | 36.3 (22.3 to 57.3) | 31.3 (13.4 to 62.4) | 238.5 (161 to 328.9) | 0 (0 to 0) | 7.6 (5.6 to 10.5) | 1.2 (0.7 to 1.8) | 230.5 (166.2 to 290.7) | 26.6 (17.9 to 39.9) | 1.7 (1.2 to 2.3) | 29.4 (16.7 to 64.6) | 319.9 (237.8 to 457.2) | 217.7 (182.7 to 266.4) | 141.9 (114 to 178.7) | 0.8 (0.5 to 1.3) | 7.9 (4.2 to 13.1) | 299.5 (235.1 to 370.3) | 171.8 (141.1 to 223.9) | |
|  |  | Golestan | 161.1 (128.7 to 193.8) | 33.2 (22.3 to 53) | 44.1 (35.8 to 54.2) | 79.8 (53.3 to 115.7) | 72.9 (43.6 to 108.6) | 45.1 (20.1 to 92.5) | 856 (693 to 1026.3) | 0 (0 to 0) | 7.9 (5.5 to 11.1) | 4.2 (1.9 to 7) | 262.4 (195.9 to 332.4) | 51.1 (30.3 to 71.3) | 6.6 (4.7 to 9) | 92.8 (56.2 to 186.6) | 786.5 (629.2 to 1012.2) | 290.7 (243.9 to 349.1) | 210.7 (147.4 to 260.1) | 2.5 (1.8 to 3.7) | 7.3 (3.9 to 12.8) | 360.5 (284 to 448.6) | 212.7 (173.5 to 280.8) | |
|  |  | Hamadan | 199.7 (154.9 to 240.1) | 34.3 (23.4 to 56.7) | 46.7 (38.2 to 57.8) | 83 (57.3 to 117) | 77.2 (50.4 to 112.8) | 42.4 (18.7 to 86.3) | 1061.3 (864.7 to 1275) | 0 (0 to 0) | 11.1 (7.3 to 17.1) | 4.9 (2.4 to 8.4) | 382.5 (242.6 to 506.7) | 60.4 (34.8 to 92.2) | 4.6 (3.1 to 6.4) | 92.2 (64.7 to 150.2) | 849 (665.1 to 1150.1) | 259.4 (211.8 to 322.9) | 197.3 (148.9 to 253.3) | 1 (0.7 to 1.6) | 22.3 (11.4 to 37.7) | 611.6 (506.1 to 721.1) | 260.3 (216.6 to 321.8) | |
|  |  | Hormozgan | 145.7 (116.9 to 170.8) | 22.2 (14.8 to 40.5) | 46.6 (35.2 to 57.8) | 74.9 (52.2 to 107.2) | 115.5 (74 to 160.2) | 67.2 (42.4 to 113.4) | 958.1 (769.1 to 1150.4) | 0 (0 to 0) | 12.2 (5.9 to 17.3) | 6.7 (2.9 to 14.2) | 238.6 (176.4 to 305.5) | 39.6 (27.3 to 54.8) | 11.3 (8.5 to 14.3) | 71.8 (50.6 to 126.2) | 782.4 (614.1 to 1060.3) | 315.2 (257.8 to 367.9) | 113.8 (88.1 to 145.7) | 1.1 (0.8 to 1.7) | 8.6 (4.7 to 14.8) | 422.1 (342.7 to 498.1) | 259 (214.1 to 324.7) | |
|  |  | Ilam | 127.2 (97.4 to 159.8) | 16.8 (11.1 to 30.6) | 41.6 (30.4 to 53.2) | 45.2 (30 to 66.8) | 66.2 (36.2 to 98.9) | 46.4 (20 to 97.2) | 925.4 (648.7 to 1217.5) | 0 (0 to 0) | 5.9 (4.2 to 8.4) | 2.2 (0.7 to 10) | 259 (173.9 to 346.6) | 22.6 (13.7 to 41.7) | 4.2 (2.7 to 5.7) | 38.2 (24.7 to 66.7) | 791.7 (553.5 to 1140.7) | 283.1 (222.8 to 344.2) | 332.2 (217.5 to 407.3) | 0.8 (0.5 to 1.2) | 1.8 (1.1 to 3.1) | 337.7 (254.4 to 435.9) | 235.4 (192.6 to 285.7) | |
|  |  | Isfahan | 141.8 (110.6 to 176.1) | 29.5 (20.5 to 47.8) | 45.2 (35.1 to 57.3) | 56 (41.4 to 79.7) | 47.9 (30.4 to 73.4) | 33.6 (14.7 to 69.8) | 610.5 (438.8 to 804.8) | 0 (0 to 0) | 15.4 (7.7 to 22.1) | 2.4 (1.3 to 3.8) | 365.7 (221.9 to 460.2) | 42.6 (28.9 to 58.6) | 4.3 (3.1 to 5.7) | 57.4 (39.9 to 103) | 686.9 (535 to 899.3) | 289 (236.9 to 353.1) | 123.6 (97.6 to 171.5) | 1.7 (1.2 to 2.7) | 7.9 (4 to 13.3) | 396.1 (305 to 489.4) | 264.8 (225.1 to 310.7) | |
|  |  | Kerman | 191.2 (155.6 to 229.6) | 65.1 (35.2 to 101.6) | 56.5 (44.8 to 70.6) | 81.6 (59.3 to 111.2) | 107.5 (71.4 to 152.1) | 49.3 (26.1 to 93.2) | 1228.6 (1015.2 to 1469.1) | 0 (0 to 0) | 17.2 (9.2 to 24.2) | 7.5 (2.8 to 12) | 378.2 (244.3 to 477.1) | 39.4 (26.7 to 56.1) | 10.4 (7.5 to 13.9) | 104.6 (59.9 to 248.3) | 898.3 (706 to 1211.8) | 360.4 (302.7 to 420.4) | 187 (148.6 to 242.6) | 1.9 (1.4 to 2.9) | 14.6 (8.5 to 23.7) | 661.4 (526.7 to 769.8) | 297.2 (253.7 to 377.1) | |
|  |  | Kermanshah | 160.7 (123.3 to 196) | 33.7 (22.9 to 56.7) | 44.9 (34.3 to 56.2) | 68.2 (48.1 to 97.8) | 72.9 (44.8 to 108.3) | 62.2 (38.2 to 107) | 1126.6 (916.8 to 1350.4) | 0 (0 to 0) | 13.5 (9.3 to 18.9) | 2.1 (1 to 4.3) | 343.4 (229.3 to 432.8) | 26.9 (17.2 to 50.5) | 10.3 (7.2 to 13) | 90 (47.2 to 236.6) | 789.8 (609.4 to 1072.6) | 304 (252.5 to 364.8) | 366.3 (235.4 to 467.1) | 1 (0.7 to 1.5) | 23.8 (12.5 to 40.3) | 420 (332.7 to 525.3) | 295.4 (239.6 to 356.6) | |
|  |  | Khorasan-e-Razavi | 154.8 (116.3 to 193.6) | 47.8 (29 to 85.1) | 48.4 (35.1 to 62.5) | 80.8 (53.5 to 122.7) | 82.6 (47.6 to 124) | 44.3 (21.8 to 82.3) | 899.5 (638.1 to 1193.7) | 0 (0 to 0) | 11.6 (7.9 to 17.2) | 5.5 (2.5 to 10.7) | 388.6 (231.9 to 525.8) | 48.6 (30.2 to 73.6) | 8.8 (5.7 to 12.4) | 76.9 (51.9 to 133.4) | 825.7 (595.1 to 1220.2) | 353.3 (288.6 to 420.3) | 153.6 (125.6 to 193.8) | 2.1 (1.3 to 3.2) | 16.7 (8.9 to 28.1) | 525.5 (396.8 to 661.8) | 275.6 (227.5 to 338.3) | |
|  |  | Khuzestan | 195.9 (155.2 to 242) | 32.1 (22.9 to 50.9) | 56.9 (44.8 to 71.9) | 75.3 (54.9 to 105.6) | 77.8 (51.3 to 114.6) | 51.4 (27.2 to 98.6) | 870 (703.3 to 1076.5) | 0.1 (0 to 0.2) | 10.7 (7.1 to 14.9) | 3.2 (1.9 to 5.1) | 354.9 (249 to 444.5) | 44.1 (31.6 to 61.3) | 11.2 (7.6 to 15.5) | 92.9 (56.7 to 198.3) | 992.6 (802.6 to 1265.1) | 380.8 (321.3 to 450.7) | 259.5 (203.9 to 327.1) | 3.3 (2.3 to 5) | 11.3 (5.7 to 19.4) | 446.6 (326.5 to 589.7) | 418.8 (360.5 to 488.1) | |
|  |  | Kohgiluyeh and Boyer-Ahmad | 271.2 (208.9 to 334.3) | 29.5 (20.1 to 47.5) | 49.8 (39.8 to 61.7) | 65.2 (50.6 to 88) | 75.8 (48.9 to 111.4) | 65.1 (27.6 to 135.5) | 842.7 (667.7 to 1037.7) | 0 (0 to 0) | 13.2 (8.7 to 17.9) | 8.7 (3.1 to 29.1) | 428.6 (277.3 to 539.4) | 44.9 (29.9 to 66.1) | 2.6 (1.9 to 3.5) | 88.9 (64.7 to 119.3) | 863 (689.2 to 1132.1) | 267.4 (214 to 356.7) | 358.8 (227 to 472) | 2.3 (1.6 to 3.3) | 10 (5.4 to 17.8) | 687.8 (548.7 to 823.2) | 431.5 (366 to 511.6) | |
|  |  | Kurdistan | 127.9 (97.3 to 153.4) | 28 (17.5 to 52.4) | 33.5 (25.6 to 41.3) | 61.1 (40.1 to 91.8) | 83.4 (49.2 to 120.7) | 43.7 (20.3 to 87.6) | 1164.9 (956.3 to 1387.1) | 0 (0 to 0) | 11.6 (6.3 to 17.3) | 3.1 (1.5 to 4.9) | 317.6 (194.3 to 415.8) | 37.7 (26.3 to 53.2) | 5.4 (3.8 to 7.2) | 56.9 (40.9 to 94) | 776.9 (586.5 to 1065.7) | 233.7 (190.6 to 292.2) | 172 (131.6 to 215) | 1 (0.7 to 1.5) | 4.3 (2.3 to 7.6) | 454.6 (375.1 to 532.7) | 230.7 (189.6 to 278.7) | |
|  |  | Lorestan | 87.1 (67.6 to 106.8) | 12.8 (8.6 to 22.4) | 21.4 (16.4 to 27.3) | 29.5 (20.3 to 42.1) | 41.4 (25.6 to 64.7) | 47.9 (24.5 to 86.5) | 409.9 (312.4 to 514.1) | 0 (0 to 0) | 4.8 (3.5 to 7.3) | 2.5 (1.3 to 4.2) | 220.3 (150.3 to 281.3) | 19.5 (12.6 to 33.4) | 15.5 (11.2 to 20) | 41.1 (25.8 to 84) | 352.6 (258.7 to 569.8) | 265.1 (219.3 to 322.5) | 199.8 (112.3 to 257.7) | 0.3 (0.2 to 0.5) | 9.6 (4.8 to 16.9) | 249.1 (199.1 to 300.3) | 125.2 (100.5 to 162.5) | |
|  |  | Markazi | 136.5 (106.5 to 169.4) | 32.3 (22.9 to 54) | 47.7 (37.1 to 58.6) | 65 (46.1 to 88.9) | 51.1 (31.1 to 75.5) | 37 (16.5 to 73.9) | 791.4 (638.1 to 954.2) | 0 (0 to 0) | 10.8 (7.3 to 15.2) | 1.6 (0.8 to 2.8) | 391 (245.6 to 498.1) | 45.8 (29.3 to 63.5) | 4.2 (3 to 5.5) | 54.6 (43.4 to 69.3) | 628.9 (486.2 to 869.8) | 350.3 (287.7 to 410.9) | 117.1 (92.9 to 164.1) | 1.6 (1.2 to 2.6) | 11.5 (6 to 19.7) | 558.7 (465.9 to 655.4) | 235.8 (194.9 to 287.1) | |
|  |  | Mazandaran | 146.4 (115.5 to 182.6) | 20.3 (14.1 to 32.2) | 42.7 (33.3 to 53.6) | 37.1 (28.2 to 51.2) | 39.4 (24.3 to 63.2) | 34.2 (15.1 to 69.1) | 327 (233.1 to 432.2) | 0 (0 to 0) | 10.1 (7 to 14.3) | 2.8 (1.4 to 4.6) | 305.3 (211.1 to 378.8) | 30 (22.4 to 43.6) | 6.7 (5 to 8.6) | 37.9 (26.6 to 64.6) | 496.5 (370.8 to 640.5) | 230.5 (194.9 to 289.9) | 154.5 (124.9 to 194.5) | 1.3 (0.9 to 1.9) | 6.1 (3.4 to 9.9) | 457.8 (362.1 to 540.6) | 206.5 (171.5 to 262) | |
|  |  | North Khorasan | 203.7 (159.1 to 243.8) | 49.6 (34.4 to 83.7) | 57.3 (44.2 to 70.5) | 91.4 (64.7 to 127.8) | 95.5 (59.9 to 141.1) | 48.6 (24.4 to 95.9) | 824.3 (673.3 to 983.9) | 0.7 (0.1 to 1.9) | 9 (6.5 to 13.1) | 11.6 (3.1 to 58) | 402.8 (257.6 to 508.5) | 79.6 (34.3 to 117.9) | 10.3 (7.4 to 13.6) | 77.3 (56.8 to 127.6) | 830 (652.7 to 1096.6) | 437.8 (359 to 530.5) | 236.8 (173.5 to 303.5) | 1.9 (1.4 to 3) | 12.8 (7.1 to 21.3) | 619.4 (502 to 754.5) | 308.2 (253.7 to 388.4) | |
|  |  | Qazvin | 94.9 (73.1 to 116.5) | 20.5 (13.9 to 33.8) | 28.3 (21.8 to 35.5) | 79.3 (55.9 to 111.7) | 52.3 (30.6 to 77.7) | 37.9 (16.1 to 77) | 585.5 (437.8 to 729.5) | 0 (0 to 0) | 6.3 (4.4 to 8.9) | 0.6 (0.2 to 2.2) | 296.7 (184.2 to 373.6) | 30.5 (21.3 to 43.1) | 3.6 (2.5 to 4.8) | 39.2 (29.7 to 53.2) | 533 (410 to 749.8) | 302.8 (257.8 to 356.7) | 104.4 (83.5 to 131.7) | 1.6 (1.1 to 2.4) | 12.7 (6.5 to 22.6) | 349.3 (279.6 to 424.3) | 186.4 (151.5 to 224.7) | |
|  |  | Qom | 85.7 (63.3 to 108) | 23.2 (15.6 to 39.2) | 35.3 (26.4 to 44.7) | 54.1 (36.7 to 77.5) | 46.9 (27 to 69.4) | 50.8 (29.7 to 93.3) | 492.1 (366.9 to 621.8) | 0 (0 to 0) | 6 (4.2 to 9) | 4.2 (1.4 to 20.3) | 328.8 (189.5 to 418.8) | 28.3 (19.4 to 40.7) | 2.3 (1.6 to 3.3) | 37.7 (25 to 64) | 523.9 (404.2 to 707.5) | 297.2 (241.2 to 361.3) | 72.5 (57 to 116.6) | 6.5 (4.5 to 9.4) | 4.9 (2.5 to 8.3) | 195.6 (147.2 to 304) | 195.7 (159.5 to 246.7) | |
|  |  | Semnan | 97 (73.3 to 118.9) | 23.8 (15.8 to 38.4) | 37.8 (29.5 to 48.1) | 44 (29.9 to 62.7) | 50.8 (29.1 to 81.4) | 35.3 (13.5 to 78.7) | 675.4 (471.3 to 908.4) | 0 (0 to 0) | 3.8 (2.5 to 6.9) | 2.1 (1.1 to 3.3) | 266 (179.5 to 343.1) | 43.7 (23.6 to 63.1) | 5 (3.3 to 7.2) | 31.5 (21.1 to 47.8) | 632.8 (436.6 to 927.4) | 427.3 (361.6 to 498.7) | 90.2 (71.9 to 123.4) | 1.6 (1.1 to 2.5) | 9.7 (5.3 to 16) | 373.2 (288.2 to 460.5) | 189.8 (151.7 to 250.5) | |
|  |  | Sistan and Baluchistan | 290.1 (229.3 to 349.5) | 99.4 (40 to 161.1) | 102 (54.7 to 128.7) | 165.1 (118.1 to 223.6) | 195.9 (128.4 to 274.4) | 84.9 (44.7 to 154.1) | 1624 (1324.6 to 1899.9) | 0 (0 to 0) | 13.1 (8.3 to 18.8) | 29.8 (3.5 to 50.9) | 324.1 (241.7 to 424.9) | 64.2 (46.8 to 88.3) | 25.9 (18.9 to 34.5) | 252.6 (124.2 to 531.3) | 1120.3 (864.3 to 1539.8) | 471.6 (392.6 to 549.8) | 161.4 (123.3 to 242.8) | 0.9 (0.7 to 1.4) | 23.3 (12.8 to 38.9) | 717.2 (603 to 845) | 437.3 (368.9 to 538.9) | |
|  |  | South Khorasan | 116.6 (87.8 to 142.7) | 43.5 (24.7 to 76.2) | 44 (32.2 to 57.1) | 81.1 (53.8 to 117.9) | 71.5 (41.8 to 105.6) | 39.9 (15.9 to 82.5) | 939.2 (741.5 to 1139.1) | 0 (0 to 0) | 6.7 (4.9 to 9.6) | 7.8 (2 to 13.1) | 303.9 (183.4 to 405.8) | 34.2 (24 to 48.6) | 15.4 (10.6 to 20.2) | 88.6 (49.9 to 188.3) | 644.4 (460.2 to 935) | 347 (281.8 to 412.7) | 109.1 (86.4 to 139.7) | 1.7 (1.2 to 2.7) | 11.9 (6.6 to 20.5) | 451.9 (365 to 530.6) | 255.3 (216.4 to 307.3) | |
|  |  | Tehran | 130.6 (101.9 to 168.3) | 23 (17.5 to 32.3) | 55.9 (43.2 to 69.3) | 52.4 (37.6 to 69.3) | 39.7 (24.6 to 61.6) | 49.1 (27.4 to 95.3) | 392.5 (215.3 to 615.8) | 0 (0 to 0) | 27.7 (9.1 to 39.6) | 1.1 (0.6 to 3.1) | 391.8 (272.8 to 474) | 58.3 (46.6 to 77.4) | 4 (2.7 to 5.9) | 48.6 (31.3 to 84) | 544.5 (379.7 to 736.8) | 276.1 (235.2 to 321.2) | 104 (81.7 to 163.4) | 1.6 (1.2 to 2.3) | 50.3 (27.1 to 83.3) | 101.1 (65.9 to 249.8) | 248.5 (185.8 to 311.3) | |
|  |  | West Azarbayejan | 220.6 (168.6 to 265.6) | 50.7 (33.7 to 84.4) | 53.3 (41.5 to 66.4) | 76.7 (56.6 to 106.1) | 89.7 (55.6 to 128.1) | 43.9 (20.2 to 85.8) | 971.9 (796 to 1163.4) | 0 (0 to 0) | 10.8 (8.1 to 15.3) | 3.6 (1.9 to 5.5) | 444.4 (273.6 to 583.4) | 61.2 (35.4 to 90.9) | 10.1 (6.7 to 13.5) | 93.1 (54.5 to 204.6) | 942.5 (758.6 to 1231.9) | 310.3 (257.9 to 395) | 355.6 (267.5 to 449.8) | 1.9 (1.4 to 3) | 5.6 (3 to 10) | 498.8 (397.1 to 624.4) | 311.1 (262.2 to 389.6) | |
|  |  | Yazd | 123.1 (94.3 to 154.6) | 30.6 (21.9 to 46.5) | 45.6 (35.5 to 57.2) | 46.4 (35.4 to 64.7) | 60 (38.4 to 88.7) | 44.4 (23 to 84.5) | 864.1 (672.7 to 1076.6) | 0.2 (0 to 0.5) | 15.2 (7.8 to 20.7) | 2.2 (1.3 to 3.3) | 439.8 (252.3 to 546.3) | 50 (30.3 to 66.8) | 6.1 (4.5 to 8) | 51 (34.4 to 92.6) | 883.7 (691.1 to 1118.6) | 492.9 (423.5 to 568.8) | 91.8 (71.7 to 122.6) | 4.6 (3.2 to 6.8) | 8.3 (4.2 to 13.9) | 426 (335 to 535.2) | 238.2 (203.4 to 300.5) | |
|  |  | Zanjan | 114.2 (90.2 to 137.2) | 27.1 (18.5 to 45.7) | 28.9 (22.1 to 36.6) | 52.3 (35 to 75.3) | 65.5 (39.5 to 96.9) | 42.9 (19.8 to 83.8) | 948.9 (740.9 to 1164.4) | 0.2 (0 to 0.4) | 4.1 (3 to 6.5) | 3.6 (1.9 to 6) | 265.6 (170.9 to 344.1) | 29.2 (20.5 to 42.3) | 2.8 (1.9 to 3.7) | 56 (39.6 to 89.8) | 661 (486.8 to 946.3) | 323.6 (270.8 to 379) | 112.4 (91.1 to 137.8) | 0.5 (0.4 to 0.8) | 7.3 (3.8 to 12.8) | 428.8 (355 to 505.1) | 207.1 (169.8 to 245.3) | |
| YLLs (Years of Life Lost) | Males | Province | Causes |  |  |  |  |  |  |  |  |  |  |  |  |  |  |  |  |  |  |  |  |  |
|  |  |  | Cardiovascular diseases | Chronic respiratory diseases | Diabetes and kidney diseases | Digestive diseases | Enteric infections | HIV/AIDS and sexually transmitted infections | Maternal and neonatal disorders | Mental disorders | Musculoskeletal disorders | Neglected tropical diseases and malaria | Neoplasms | Neurological disorders | Nutritional deficiencies | Other infectious diseases | Other non-communicable diseases | Respiratory infections and tuberculosis | Self-harm and interpersonal violence | Skin and subcutaneous diseases | Substance use disorders | Transport injuries | Unintentional injuries | |
|  |  | Alborz | 174.8 (131.8 to 231.9) | 24.2 (17.9 to 31.5) | 35.3 (27.9 to 46.1) | 48.4 (35.5 to 69.4) | 48.9 (31.4 to 74) | 47.8 (22.1 to 91.7) | 467.8 (349.4 to 589.5) | 0 (0 to 0) | 3.6 (1.8 to 5.2) | 4.8 (1.4 to 25.2) | 325.5 (236.3 to 421.7) | 41.8 (29.8 to 64.3) | 4.1 (2.9 to 5.6) | 51.9 (33.5 to 95.8) | 554.6 (439.4 to 723.8) | 503.1 (422.5 to 570.8) | 445 (327.3 to 585.7) | 2 (1.3 to 3) | 56.1 (27.7 to 97.9) | 716.4 (552.6 to 890.6) | 530.5 (430.6 to 642) | |
|  |  | Ardebil | 248 (188 to 311.5) | 25.8 (19.2 to 33.4) | 46.9 (34.2 to 61.5) | 49.8 (36.1 to 73.2) | 69 (43.7 to 103.3) | 43.8 (15.5 to 95.5) | 1084.1 (842.2 to 1320) | 0 (0 to 0) | 3.5 (1.8 to 4.9) | 19.5 (1.2 to 175.2) | 428.4 (259.8 to 588) | 69.2 (46.6 to 91.9) | 5.4 (3.4 to 7.6) | 65.6 (37 to 137.2) | 1114.6 (873.3 to 1442.3) | 308.5 (255.8 to 349) | 340.5 (260.8 to 435.6) | 1 (0.7 to 1.6) | 15.9 (8.1 to 28) | 853.9 (667.9 to 1042.8) | 510.7 (389.9 to 699.9) | |
|  |  | Bushehr | 136.7 (106.6 to 167) | 14.9 (11.5 to 18.8) | 31 (23.1 to 41.3) | 49.8 (34.1 to 75.3) | 57.2 (38.4 to 85.1) | 50.5 (25.7 to 98.9) | 979.5 (782 to 1188.2) | 0 (0 to 0) | 2.2 (1.4 to 3.4) | 5.1 (1.9 to 15.7) | 288.9 (189.9 to 389.2) | 37.2 (25.9 to 55.6) | 18.6 (12.5 to 26.6) | 40.2 (29.5 to 55.2) | 794.3 (638.3 to 1063.4) | 305.3 (256.4 to 352.1) | 215.7 (164.3 to 275.3) | 6.8 (4 to 10.9) | 15.2 (7.6 to 28.3) | 1016.4 (835.9 to 1211.3) | 419.1 (341.2 to 543.9) | |
|  |  | Chahar Mahaal and Bakhtiari | 137.8 (106.3 to 175.8) | 15.9 (11.4 to 20.5) | 22.3 (16.7 to 28.5) | 23.1 (16 to 35.6) | 51.4 (27.1 to 85.8) | 45.3 (16.3 to 96) | 590.1 (439.6 to 749.8) | 0 (0 to 0) | 0.9 (0.5 to 2.5) | 1.9 (0.8 to 3.1) | 278.6 (151.3 to 401.9) | 46.9 (31.6 to 62.3) | 1.1 (0.7 to 1.6) | 29.6 (17.7 to 56.7) | 594.1 (451.6 to 833.8) | 204.9 (167.2 to 233.9) | 215.3 (160.2 to 272.9) | 0.1 (0 to 0.1) | 9 (4.2 to 17) | 778 (618.5 to 940.2) | 413.8 (325.8 to 533.5) | |
|  |  | East Azarbayejan | 220 (171.7 to 275.1) | 43.6 (34.6 to 55.7) | 53.5 (39.6 to 67.8) | 64.6 (47.4 to 99.7) | 55.5 (36.5 to 83.5) | 40.8 (17.6 to 83.4) | 607.7 (443.9 to 787.5) | 0 (0 to 0) | 3.6 (2.2 to 5.1) | 8 (1.6 to 54.9) | 432.5 (281.5 to 594.2) | 90.2 (41 to 131.7) | 11.1 (7.9 to 15.4) | 64.5 (41 to 120.2) | 796.2 (626.4 to 1049.9) | 337.9 (279.6 to 385.7) | 310.4 (225.7 to 404.7) | 4.9 (3.3 to 7.5) | 37.9 (18.6 to 70.3) | 1219.2 (995.7 to 1466.1) | 517.8 (421.8 to 652) | |
|  |  | Fars | 261.9 (204.7 to 327.1) | 32.6 (25.8 to 41.3) | 58.5 (43.4 to 75.3) | 97.7 (65.2 to 147.2) | 61.3 (41.2 to 91) | 66.3 (41.5 to 112.6) | 1048.2 (793.5 to 1339) | 0 (0 to 0) | 3.7 (2.4 to 6.1) | 11.8 (2.1 to 86.4) | 475.2 (310.7 to 675.2) | 70.7 (36.2 to 102.1) | 3.8 (2.7 to 5.1) | 77.7 (53 to 122.4) | 1111.3 (885 to 1457.3) | 398.6 (336.5 to 466.8) | 542.7 (410.4 to 714.5) | 3.8 (2.4 to 6.1) | 58.9 (28.2 to 103.4) | 1919.5 (1583 to 2266.6) | 657.3 (541.5 to 794.3) | |
|  |  | Gilan | 261.5 (200.7 to 334.5) | 29.1 (22.1 to 38.2) | 39.3 (29.8 to 52.9) | 38.7 (28.3 to 54.1) | 40.6 (24.3 to 63.6) | 32.6 (11.9 to 68.5) | 251.3 (165.8 to 352.9) | 0 (0 to 0) | 3.4 (2 to 4.9) | 1.6 (0.8 to 2.5) | 332 (237.4 to 441.4) | 41.9 (31.5 to 56.5) | 1.9 (1.4 to 2.4) | 39 (25.1 to 70.9) | 409.7 (320.9 to 519.9) | 289.5 (243.1 to 323.1) | 355.4 (277.6 to 464.8) | 1.3 (0.9 to 2.1) | 26.6 (13.1 to 47.7) | 1390.3 (1123.6 to 1676.9) | 626.2 (510.9 to 785.9) | |
|  |  | Golestan | 310.2 (249.3 to 385.5) | 46.5 (35.3 to 60.3) | 63 (48.2 to 79.7) | 108.8 (68.4 to 160.8) | 78.6 (52.8 to 113.1) | 50.1 (21.9 to 101.4) | 1050.5 (853.4 to 1262.1) | 0 (0 to 0) | 4 (2.6 to 6.3) | 5.6 (2.4 to 11.2) | 436 (323.5 to 577.5) | 87.7 (48.9 to 120.7) | 11.8 (8.2 to 15.8) | 116.2 (68.6 to 204.6) | 1082.1 (886.5 to 1383.8) | 410.3 (345.1 to 469) | 372.8 (285.3 to 488.6) | 5.9 (4 to 9) | 18.5 (8.5 to 32) | 1793.4 (1499.6 to 2067.7) | 646 (520.8 to 820.4) | |
|  |  | Hamadan | 315.5 (252 to 396) | 42.3 (32.1 to 55.1) | 54 (40.5 to 71.8) | 94.7 (67.1 to 143.4) | 81.7 (53 to 121.7) | 47.6 (21 to 96.4) | 1141.5 (952.5 to 1343.6) | 0 (0 to 0) | 4.4 (2.5 to 6.6) | 5.3 (2.2 to 11.9) | 497.8 (341.6 to 676.1) | 79.5 (54.5 to 105.4) | 7.6 (5.4 to 10.2) | 101.4 (69.4 to 154.8) | 1052.9 (868.5 to 1342.9) | 341.2 (284.5 to 398.6) | 446.6 (320.2 to 585.4) | 4 (2.5 to 6.3) | 81.4 (40 to 140.1) | 1596.1 (1326.6 to 1882.7) | 652.2 (534.9 to 817.1) | |
|  |  | Hormozgan | 297.8 (233.4 to 365.2) | 28.7 (22.8 to 35.4) | 70.7 (39.2 to 93.2) | 95.3 (60.3 to 135.6) | 77.7 (44.8 to 141.1) | 68.8 (40.4 to 121.5) | 1213.7 (1004.6 to 1457) | 0 (0 to 0) | 7.3 (2.2 to 10) | 8.8 (2.8 to 26) | 333.6 (256.4 to 424.5) | 67.5 (45.3 to 89.9) | 15 (10.9 to 18.9) | 77.9 (54 to 128.6) | 1006.4 (831.7 to 1275.5) | 410.7 (348.7 to 473.9) | 387.9 (292.8 to 499.9) | 5.5 (3.6 to 8.2) | 29.9 (15.6 to 52.9) | 1729.5 (1397.6 to 2050.1) | 668.3 (548.3 to 828.4) | |
|  |  | Ilam | 202.3 (153.8 to 258.8) | 18.2 (13.8 to 23.6) | 59.3 (32.6 to 82.2) | 47.4 (33.6 to 73.3) | 61 (39.4 to 88.9) | 46.6 (17.8 to 103.7) | 1144.9 (795.4 to 1496.3) | 0 (0 to 0) | 3.3 (1.8 to 4.8) | 6.9 (2.2 to 23.4) | 347.1 (222 to 500.2) | 45.2 (30.9 to 62) | 8.8 (5.4 to 12.4) | 51.3 (32.4 to 80.5) | 942.3 (663.4 to 1345.2) | 341.9 (279.7 to 402.6) | 617.9 (456 to 782) | 1.1 (0.7 to 2) | 9.4 (4.5 to 17.7) | 1043.7 (806.5 to 1290.9) | 515.1 (405 to 670.7) | |
|  |  | Isfahan | 200 (148.6 to 250.3) | 32.5 (24 to 42.7) | 49.7 (36.9 to 62.5) | 57 (42.5 to 85.4) | 50.1 (33.5 to 77.1) | 35.7 (14.5 to 74.9) | 741.2 (540.3 to 1000.6) | 0 (0 to 0) | 4.5 (1.9 to 6.6) | 3.8 (1.8 to 7.1) | 411.6 (262.3 to 560.6) | 62.1 (40.4 to 82.5) | 4.3 (3 to 6.1) | 55.4 (36.7 to 90.3) | 831.2 (643 to 1112.1) | 374.1 (307.2 to 446.2) | 272.1 (211.8 to 355) | 4.1 (2.7 to 6.4) | 30.9 (14.6 to 56.3) | 1099.6 (899 to 1364) | 595.8 (476.4 to 728.8) | |
|  |  | Kerman | 238.5 (186.1 to 297.3) | 65.4 (53.3 to 81.9) | 54.3 (38.2 to 70) | 85.3 (56.9 to 120) | 84.4 (57.4 to 122.5) | 52.7 (26.2 to 103.2) | 1424.6 (1192.3 to 1703.2) | 0 (0 to 0) | 5.5 (2 to 7.9) | 8.7 (3 to 14.5) | 383.9 (267.7 to 535.2) | 58.8 (42.3 to 74.7) | 8.7 (6.2 to 11.2) | 102.9 (57.2 to 249) | 1054.2 (861 to 1335.2) | 430.9 (358.8 to 500.3) | 363.8 (272.5 to 485.6) | 3.7 (2.4 to 5.7) | 28.4 (13.7 to 50.7) | 1629.2 (1371.3 to 1898.2) | 535.7 (445.1 to 672) | |
|  |  | Kermanshah | 234.9 (184.4 to 293.8) | 37.4 (28.9 to 46.7) | 51.2 (37.1 to 66.1) | 80.9 (54.7 to 122.4) | 60.6 (39.9 to 90.6) | 78 (52.1 to 123.8) | 1257.7 (1030 to 1520) | 0 (0 to 0) | 4.9 (2.2 to 6.9) | 5.1 (2.2 to 11.3) | 437.2 (275.3 to 619.6) | 44.5 (30.6 to 68.8) | 11.5 (8 to 14.8) | 94.2 (52.2 to 211.7) | 970.3 (770.5 to 1285.8) | 401.6 (338.5 to 454.9) | 510.7 (394.3 to 651) | 4.4 (2.8 to 7.4) | 69.4 (33.1 to 126.4) | 1298.7 (1065.2 to 1586.2) | 536.9 (434.1 to 682.7) | |
|  |  | Khorasan-e-Razavi | 193.1 (141.6 to 248.7) | 43.6 (32.9 to 57.1) | 54 (38.9 to 73.3) | 77.9 (52.6 to 124.5) | 65.5 (42 to 100) | 45.4 (18.8 to 90.5) | 1040.3 (746.4 to 1333.4) | 0 (0 to 0) | 3.7 (2.1 to 5.6) | 7.3 (2.5 to 18.8) | 439.4 (271.6 to 646.1) | 69.1 (45.1 to 98.6) | 9 (5.9 to 12.7) | 77 (48.7 to 134.5) | 964.2 (729 to 1367.3) | 429.6 (349.1 to 506.2) | 297.1 (229.3 to 381.3) | 3.7 (2.3 to 6) | 42.8 (21 to 74.4) | 1291.5 (1017 to 1557.2) | 552 (442.3 to 670.9) | |
|  |  | Khuzestan | 238.2 (183.9 to 302.5) | 30.4 (23.2 to 40.7) | 56.5 (44.4 to 73.8) | 71.7 (50.7 to 109.2) | 63.9 (42.2 to 95.3) | 55.6 (27.9 to 108.5) | 1076.4 (851 to 1318.2) | 0 (0 to 0) | 3.4 (2.2 to 5.2) | 4.3 (2.2 to 7.6) | 387.5 (280.5 to 506) | 57.3 (42.4 to 74.7) | 10.2 (6.8 to 15.3) | 88.1 (54.1 to 181.3) | 1111.5 (894.3 to 1417.8) | 457.1 (381.5 to 525.8) | 356.6 (271.3 to 464.6) | 7.4 (5 to 11.3) | 31.5 (15.4 to 58.8) | 1173.7 (963.4 to 1398.1) | 798.7 (676.2 to 943.7) | |
|  |  | Kohgiluyeh and Boyer-Ahmad | 272.2 (205.2 to 346.4) | 24.4 (18 to 31.7) | 43.6 (33.5 to 55.6) | 64.4 (48.2 to 94.4) | 66 (45 to 97.5) | 69 (27.4 to 141.5) | 990.9 (780.2 to 1243.2) | 0 (0 to 0) | 4.9 (2.1 to 7) | 10.7 (2.6 to 55.2) | 452.2 (277.2 to 640.7) | 67.9 (43.9 to 93) | 3.8 (2.4 to 5.3) | 88.1 (59.9 to 124.6) | 992.5 (789.7 to 1328.5) | 297.5 (241.2 to 357.1) | 411.6 (315.3 to 524.2) | 1.4 (1 to 2.2) | 13.3 (6.6 to 25.8) | 1376 (1090.7 to 1643.3) | 695.5 (573.7 to 836) | |
|  |  | Kurdistan | 217.9 (172.9 to 279.8) | 32.2 (24.4 to 41.2) | 45.8 (33.8 to 61.5) | 70.2 (50.3 to 105.6) | 65.7 (42.2 to 96.5) | 50.3 (22.9 to 103) | 1360.7 (1122.7 to 1615.9) | 0 (0 to 0) | 4.1 (2 to 5.9) | 4.5 (1.9 to 7.4) | 431.5 (259.1 to 626.3) | 77.2 (47.3 to 108.1) | 4.9 (3.5 to 6.6) | 66.7 (46.4 to 98.3) | 931.4 (726.5 to 1247) | 306.5 (256.6 to 354.2) | 374.7 (305.5 to 466.4) | 4.9 (2.9 to 8.3) | 16.5 (7.5 to 29.9) | 1373.3 (1136.1 to 1634.3) | 599.1 (485.4 to 753.7) | |
|  |  | Lorestan | 137 (104 to 171.8) | 14.6 (10.8 to 18.8) | 28 (20.3 to 37.1) | 38.1 (27.4 to 58.4) | 51.3 (32.6 to 81.7) | 54.1 (27.2 to 99.6) | 519.5 (390.5 to 671.5) | 0 (0 to 0) | 2.2 (1.3 to 3.2) | 2.5 (1.2 to 3.9) | 273.5 (167.2 to 380.1) | 34 (22.6 to 53.1) | 20.9 (14.4 to 28.1) | 50.5 (31.7 to 91.7) | 442 (340.2 to 604.6) | 358.4 (291.2 to 418.1) | 294 (215.9 to 375.7) | 1 (0.6 to 1.7) | 34.2 (16.2 to 61.1) | 716.2 (574.5 to 871.3) | 357.9 (278.1 to 505.2) | |
|  |  | Markazi | 176.1 (133.2 to 225.2) | 33.6 (26 to 44.2) | 47.4 (35.6 to 62.6) | 62.8 (45.8 to 92.1) | 53 (34.7 to 80.4) | 41 (17.9 to 80.2) | 847.3 (683.3 to 1019.7) | 0 (0 to 0) | 2.9 (1.9 to 4.6) | 2.7 (1.3 to 4.1) | 418.7 (245.6 to 602.9) | 64.8 (41.1 to 87.2) | 3.3 (2.4 to 4.2) | 55.4 (39.9 to 74.7) | 685.8 (537.9 to 913.5) | 422.4 (349.8 to 499.3) | 270 (204.2 to 336.1) | 4.1 (2.8 to 6.3) | 33.1 (16 to 65.6) | 1243.8 (1010.6 to 1493) | 498.2 (406.9 to 608.8) | |
|  |  | Mazandaran | 188.8 (142.1 to 247.6) | 21.8 (16.5 to 28.6) | 37.8 (29.3 to 48.6) | 37.6 (27.9 to 57) | 44.3 (28.5 to 69.9) | 35.7 (13 to 75.4) | 397.8 (289.7 to 506.6) | 0 (0 to 0) | 4 (1.7 to 5.7) | 2.5 (1.1 to 5.5) | 335.8 (224.5 to 457.9) | 42.3 (32.2 to 55.3) | 4.7 (3.3 to 6.5) | 39 (26.6 to 62) | 586.1 (452.2 to 750.8) | 291.7 (242.6 to 325.8) | 275.6 (214.8 to 346.5) | 3.1 (1.9 to 4.8) | 14.2 (6.9 to 26) | 1162.1 (946.9 to 1396.1) | 616.4 (502.4 to 745.9) | |
|  |  | North Khorasan | 230 (182.8 to 290.9) | 40.9 (30.7 to 55.8) | 57.6 (42 to 77.8) | 83.8 (57 to 122.9) | 72 (46.3 to 111.2) | 47 (18.3 to 99.3) | 947.5 (748.4 to 1142.6) | 0 (0 to 0) | 4.9 (2 to 7.1) | 19 (3.1 to 120.6) | 420.7 (280.6 to 580) | 74.3 (45.6 to 102.4) | 10.5 (7.4 to 14.3) | 87.4 (57.1 to 132.4) | 905.8 (725.6 to 1193.2) | 514.2 (414.7 to 615.9) | 285.1 (226.2 to 361) | 4.3 (2.8 to 6.8) | 21.2 (10 to 38.6) | 1467.6 (1217.4 to 1733.8) | 591.4 (491.4 to 707.9) | |
|  |  | Qazvin | 133.5 (102.1 to 170) | 22.8 (17.6 to 29.4) | 32.6 (24.8 to 41.6) | 78.1 (40 to 121.7) | 53 (34.6 to 80.7) | 41.9 (17 to 87.9) | 713.3 (552.7 to 886.1) | 0 (0 to 0) | 2.1 (1.5 to 3.4) | 1.6 (0.8 to 3.1) | 324.7 (201.2 to 457.1) | 44.6 (31.4 to 62.4) | 2.2 (1.4 to 3.1) | 40.3 (28.7 to 55.4) | 638.5 (491.4 to 849.6) | 398.9 (335.1 to 450.8) | 224.3 (178.6 to 287.9) | 5.1 (3.3 to 8.3) | 44 (21.3 to 78.2) | 925.9 (713.9 to 1146.9) | 475.5 (379.6 to 602.9) | |
|  |  | Qom | 126.9 (92.8 to 167.1) | 28.9 (21.4 to 39) | 42.9 (31.8 to 54.9) | 62 (41 to 89.6) | 52.3 (33.5 to 81.4) | 54.7 (29.5 to 98.4) | 554.1 (411.5 to 715.5) | 0 (0 to 0) | 2 (1.4 to 4.1) | 6.6 (1.2 to 49.4) | 404.2 (241.9 to 561.5) | 44.6 (30 to 67.2) | 2 (1.3 to 2.9) | 45.3 (30.5 to 74) | 611.2 (464.1 to 818.5) | 382.5 (304.9 to 454.2) | 257.1 (195.9 to 341.2) | 16.9 (11.6 to 25.3) | 16.8 (8 to 30.9) | 534.8 (396.8 to 714.4) | 524 (370.9 to 655.3) | |
|  |  | Semnan | 129.5 (95.7 to 172.6) | 25.7 (19.1 to 34) | 40.7 (28.3 to 54) | 50.3 (33.7 to 77) | 54 (32.1 to 84.8) | 37.4 (12.6 to 87.2) | 820.9 (551.5 to 1114.8) | 0 (0 to 0) | 2.3 (1.3 to 3.5) | 2.2 (1.1 to 3.7) | 310.7 (194.3 to 437.4) | 61.5 (36.8 to 88.7) | 4.2 (2.7 to 6) | 35.6 (24.3 to 52.8) | 712.8 (492 to 1030.1) | 529.6 (447.4 to 604.4) | 219.7 (172 to 280.2) | 3.9 (2.5 to 6.2) | 22.9 (10.3 to 43.5) | 965.1 (751.4 to 1210.2) | 394.2 (304.8 to 522.8) | |
|  |  | Sistan and Baluchistan | 396.3 (308.4 to 494.3) | 93.8 (73.3 to 120.6) | 106.7 (43.2 to 141.8) | 170.5 (81.3 to 248.6) | 131.4 (80.1 to 237.5) | 81.1 (33.7 to 164.3) | 2047.9 (1714.5 to 2418.6) | 0 (0 to 0) | 5.7 (2.4 to 8.2) | 30.3 (4.7 to 55.7) | 413.9 (305.6 to 538.8) | 91 (69.2 to 115.3) | 30.9 (21.8 to 42.2) | 258.9 (122.5 to 529.1) | 1370.6 (1101.6 to 1795.8) | 569.6 (481.8 to 652.7) | 450.2 (344.9 to 568.7) | 3.4 (2.2 to 5.3) | 30.6 (14.3 to 55) | 1748.3 (1462.4 to 2039.7) | 801.5 (655.9 to 1021) | |
|  |  | South Khorasan | 145.8 (115 to 177.8) | 44 (34.4 to 56.2) | 46 (32.4 to 62.2) | 74.6 (43.8 to 114.4) | 65.5 (43.9 to 93.1) | 41.9 (15 to 92.3) | 1098.1 (862.7 to 1341) | 0 (0 to 0) | 2.3 (1.6 to 3.7) | 3.8 (1.8 to 6) | 363.6 (209.9 to 530.1) | 55.1 (40.4 to 72.6) | 15.9 (10.7 to 20.8) | 80.8 (46.5 to 175.1) | 803.7 (609.1 to 1125.6) | 425.1 (348.4 to 498.6) | 182.8 (147.1 to 227.7) | 3.9 (2.6 to 6) | 22 (10.7 to 40.5) | 1109.1 (904.4 to 1296.3) | 448.7 (364.4 to 589.8) | |
|  |  | Tehran | 127.8 (92.2 to 178.2) | 16.6 (11.7 to 23.7) | 37.1 (27.3 to 49) | 47 (33.3 to 66) | 37.7 (21.7 to 61.7) | 50.8 (26.3 to 104.8) | 528.4 (311.4 to 785.9) | 0 (0 to 0) | 4.8 (1.3 to 7.4) | 1.8 (0.7 to 7.1) | 323.1 (212.3 to 435.7) | 64.6 (48.2 to 90.7) | 3.6 (2.3 to 5.4) | 52 (33 to 89.1) | 628.2 (435.4 to 850.3) | 311.3 (256.6 to 368.9) | 172 (136.8 to 224.3) | 1.7 (1.1 to 2.4) | 77 (34.7 to 144.1) | 138.8 (95.3 to 207.6) | 351.5 (252.5 to 463.7) | |
|  |  | West Azarbayejan | 243.9 (183.9 to 300.7) | 44.3 (34 to 56.8) | 47.5 (36 to 61.2) | 70.4 (52.8 to 103.1) | 68.9 (45.4 to 102.1) | 45.9 (19.2 to 95.3) | 1113.1 (871.5 to 1335.5) | 0 (0 to 0) | 4 (2 to 5.5) | 3.6 (1.8 to 5.6) | 452 (283 to 634.5) | 79.7 (49 to 109.5) | 10.9 (7.3 to 14.6) | 84.5 (45.8 to 184) | 1056.7 (839.3 to 1394.1) | 367.9 (303.7 to 425.8) | 437.4 (340.6 to 545.3) | 2.6 (1.7 to 4.2) | 11.1 (5.6 to 19.6) | 1280.8 (1045.4 to 1543.3) | 568.2 (456.8 to 755.7) | |
|  |  | Yazd | 149 (111.4 to 196.4) | 38.6 (29 to 50.6) | 49 (36.3 to 62) | 51.3 (38.9 to 72.8) | 57.2 (39 to 82.9) | 44 (19.5 to 89.9) | 1006.5 (807.5 to 1219.5) | 0 (0 to 0) | 3.8 (1.9 to 5.2) | 2.1 (1.1 to 3.6) | 421.6 (256.7 to 594.4) | 64.6 (38.7 to 87.9) | 9.9 (6.9 to 13.1) | 51.6 (35.5 to 85.1) | 996.1 (801.4 to 1271.5) | 599.4 (503 to 679) | 191.2 (146 to 250.1) | 11 (7.6 to 16) | 27.8 (12.5 to 51.8) | 1095.1 (852.6 to 1345.2) | 500.4 (395.2 to 607.8) | |
|  |  | Zanjan | 164.9 (131.3 to 208.7) | 29.7 (23.2 to 38.1) | 35.6 (24.8 to 45.9) | 58.5 (41.4 to 90.5) | 73.4 (49.6 to 109.6) | 44.4 (17.4 to 89.9) | 1138.5 (904.8 to 1409.1) | 0 (0 to 0) | 3.6 (1.4 to 5) | 7.4 (2.1 to 12.2) | 327.5 (211.3 to 451) | 51.4 (38 to 66.5) | 4.2 (3 to 5.5) | 65.6 (45 to 98.3) | 787.4 (594.5 to 1081.9) | 425.3 (357.8 to 488.9) | 187.3 (149 to 241.4) | 2.2 (1.3 to 3.4) | 19.6 (9.7 to 34.4) | 1019.1 (843.3 to 1218.4) | 496 (405.3 to 611.1) | |
