# Supplementary material for: Disability-adjusted life years, years lived with disability, and years of life lost of diseases among children and adolescents in national and subnational levels of Iran, 1990–2021: A systematic analysis for the Global Burden of Disease 2021
Source: PLoS One. 2025 Jun 23;20(6):e0325085. doi: 10.1371/journal.pone.0325085 (PMC12184942; doi:10.1371/journal.pone.0325085)
Supplement: S4 Table — (DOCX) [file pone.0325085.s025.docx]

**S4 Table.** Time trend of rate of disability-adjusted life years (DALYs), years lived with disability (YLDs), and years of life lost (YLLs) of child and adolescents causes of death in 1990-2021 in Iran by sex

| DALYs (Disability-Adjusted Life Years) | Both Sexes | Causes | Year |  |  |  |  |  |  |  |  |  |  |  |  |  |  |  |  |  |  |  |  |  |  |  |  |  |  |  |  |  |  |  |
| --- | --- | --- | --- | --- | --- | --- | --- | --- | --- | --- | --- | --- | --- | --- | --- | --- | --- | --- | --- | --- | --- | --- | --- | --- | --- | --- | --- | --- | --- | --- | --- | --- | --- | --- |
|  |  |  | 1990 | 1991 | 1992 | 1993 | 1994 | 1995 | 1996 | 1997 | 1998 | 1999 | 2000 | 2001 | 2002 | 2003 | 2004 | 2005 | 2006 | 2007 | 2008 | 2009 | 2010 | 2011 | 2012 | 2013 | 2014 | 2015 | 2016 | 2017 | 2018 | 2019 | 2020 | 2021 |
|  |  | Cardiovascular diseases | 904 (782.4 to 1108.3) | 843.6 (739.1 to 1033.3) | 804.6 (707.7 to 986) | 767.9 (677.2 to 926.2) | 735.3 (648.5 to 862.4) | 703.7 (627.4 to 819.6) | 674.7 (603.3 to 775.9) | 652.6 (584.1 to 735) | 632.9 (571.5 to 702.6) | 616.5 (555.9 to 685.2) | 601.7 (545.3 to 664.6) | 589 (533 to 652.1) | 590.4 (535.5 to 653.2) | 595.2 (537 to 658.5) | 601.9 (541.5 to 670.8) | 597.2 (540.2 to 662.3) | 597.8 (542.4 to 663.4) | 602.2 (544.4 to 673.9) | 614.1 (556.5 to 682.3) | 620.4 (566.6 to 686.8) | 612.5 (561.6 to 677.2) | 606.5 (557.6 to 667) | 606 (559.3 to 659.7) | 601.1 (555.4 to 653.7) | 549.5 (510.3 to 594.6) | 471.4 (435.6 to 514.5) | 404.3 (360.9 to 445.9) | 369.9 (325.5 to 413.5) | 341.7 (293.8 to 385.2) | 322.2 (275.6 to 367) | 294.4 (255.6 to 334.5) | 271.6 (234.4 to 311) |
|  |  | Chronic respiratory diseases | 373.9 (272.6 to 503.3) | 357.4 (261.2 to 483.3) | 345.3 (255 to 469.2) | 334.2 (247.7 to 455.2) | 324.9 (242.4 to 441.4) | 316 (234.9 to 431.9) | 307.6 (228.9 to 420) | 300.8 (223.4 to 411.3) | 294.9 (217.9 to 399.8) | 290.1 (214.5 to 394.3) | 286.2 (211.1 to 388.9) | 282.4 (207.1 to 385.7) | 280.2 (204.5 to 381.7) | 279.4 (206 to 382.6) | 279.5 (206.5 to 381.4) | 278.1 (206 to 382.6) | 276.9 (205.4 to 380.2) | 275.2 (204.1 to 380.5) | 275 (205 to 380) | 273.8 (203.5 to 375.4) | 270.8 (200.7 to 369.4) | 268.2 (199 to 366.1) | 266.4 (198.2 to 361.9) | 263.2 (195 to 358.5) | 250.8 (184.4 to 342.9) | 235.4 (172.1 to 328.6) | 221.5 (157.6 to 312.8) | 212.7 (151 to 303.1) | 205.5 (143.8 to 295.8) | 201.1 (139.5 to 290.2) | 200.5 (137.6 to 293) | 194.9 (132.6 to 287.6) |
|  |  | Diabetes and kidney diseases | 119.5 (101.8 to 142.5) | 114.3 (96.7 to 134.5) | 111.4 (94.7 to 132.6) | 109.3 (92.9 to 129.9) | 106.9 (90.8 to 126.3) | 105 (90.5 to 123.7) | 103.9 (89.3 to 123.4) | 102.6 (88.1 to 121.8) | 101.5 (87.4 to 119.7) | 99.9 (86.8 to 119.4) | 99.8 (86.5 to 117.3) | 99.8 (87.7 to 116.1) | 101.4 (88.6 to 116) | 103.3 (90.6 to 117.6) | 107.2 (94.4 to 122) | 110.9 (96.9 to 125.1) | 114.5 (98.3 to 128.5) | 117.3 (99.2 to 132.9) | 120.9 (99.8 to 135.8) | 123.9 (100.8 to 139.9) | 125.2 (99.8 to 141) | 126.8 (99 to 141.4) | 127.3 (99.3 to 142) | 127.6 (99.6 to 140.8) | 119 (93.6 to 131.5) | 107.4 (85.2 to 120.7) | 95.6 (77.4 to 106.7) | 89.4 (74.3 to 100) | 84.4 (70.9 to 94.7) | 81.1 (69.2 to 91.9) | 73.9 (62.8 to 82.8) | 68.2 (58.7 to 76.6) |
|  |  | Digestive diseases | 476 (361.9 to 557.3) | 440.2 (347.5 to 517.3) | 418 (328.5 to 497.8) | 394.5 (310 to 464.6) | 379.1 (301.5 to 448) | 359.5 (282.9 to 421.4) | 337.2 (276 to 389.4) | 323.9 (269.3 to 372.4) | 311.7 (260.4 to 356.9) | 303.5 (249.8 to 349.6) | 289.2 (246.5 to 326.5) | 274.8 (235.7 to 309.2) | 271.1 (234 to 305.9) | 269.5 (227.9 to 305.3) | 278.6 (231.7 to 315.9) | 275.5 (229.2 to 311.4) | 274.4 (226.6 to 310.5) | 276.3 (225.9 to 312.5) | 281.1 (226.5 to 316.3) | 282.2 (227.6 to 319.4) | 278 (223.7 to 312) | 273.4 (221.3 to 306.5) | 269.6 (219.9 to 301.2) | 263.3 (216.3 to 293.7) | 245.8 (200.5 to 273.6) | 217 (173.1 to 245.9) | 190.2 (150 to 225.9) | 175.4 (137.2 to 212.7) | 163.4 (128.2 to 201.9) | 153.8 (119.8 to 193.4) | 135.7 (109 to 169.9) | 121 (98.3 to 151.9) |
|  |  | Enteric infections | 1430.5 (1029 to 2222.7) | 1234.5 (901.4 to 1892.2) | 1081.3 (801.4 to 1618.4) | 967.3 (731.3 to 1376.8) | 879.1 (664.1 to 1208.5) | 815.8 (627.4 to 1117.7) | 727.3 (561.3 to 981) | 676.3 (528.8 to 895.4) | 627.1 (486.5 to 817.6) | 585.8 (459 to 755.7) | 552.3 (439.8 to 700.8) | 489.5 (388.8 to 618.1) | 457.8 (365.5 to 573.9) | 430.4 (343 to 539.2) | 408.8 (326.6 to 514.1) | 392.4 (313.8 to 493) | 378.1 (304.9 to 477.3) | 366.8 (296.2 to 456.5) | 356.2 (288.1 to 440.3) | 343.8 (281.1 to 422.9) | 330.6 (269.6 to 404.8) | 323.2 (261.1 to 397) | 311.4 (249.7 to 379.4) | 304.3 (245.1 to 370.6) | 285.5 (234.4 to 349.6) | 268 (218.6 to 328.1) | 237.9 (193.2 to 293.4) | 218.3 (175.4 to 271.2) | 202.6 (159.3 to 253.2) | 190.8 (149 to 241.2) | 161.8 (125.1 to 209.1) | 139.4 (103 to 183.7) |
|  |  | HIV/AIDS and sexually transmitted infections | 65.6 (24.1 to 132.8) | 60.9 (22.8 to 125.4) | 57 (21.6 to 116.7) | 54.7 (20.6 to 113.3) | 52.9 (20.2 to 111.1) | 51.1 (19.6 to 106.9) | 49.6 (18.8 to 104.6) | 48.6 (18.9 to 100.6) | 48.1 (18.8 to 100.3) | 47.8 (19.2 to 99.5) | 47.9 (19.4 to 99.4) | 47.9 (19.5 to 101) | 48.6 (20.3 to 101.6) | 49.3 (21 to 102.2) | 50.3 (22.2 to 102) | 51.3 (22.8 to 103.1) | 53.8 (24.1 to 106.4) | 59.5 (27.2 to 115.5) | 64.4 (29.6 to 124.6) | 69.6 (32.1 to 136.6) | 73.2 (34 to 146.7) | 72.9 (32.9 to 148.8) | 75.9 (35.4 to 152.4) | 80.7 (40.3 to 155.2) | 80.7 (40.7 to 152.2) | 78.6 (40.6 to 147) | 71.5 (35.3 to 138.7) | 68.7 (34.7 to 134.2) | 64.6 (32.6 to 126.9) | 60.4 (30.7 to 117.4) | 55.6 (28.7 to 104.5) | 51.4 (26.8 to 95.2) |
|  |  | Maternal and neonatal disorders | 12291.9 (10534.6 to 14565.6) | 11143.7 (9566.6 to 13103) | 10314.5 (8834.2 to 12209.7) | 9574.4 (8131.5 to 11387.4) | 8817.6 (7489.4 to 10458.4) | 8132.2 (6888.9 to 9528.9) | 7589.4 (6500.2 to 8834.3) | 7168.4 (6094.2 to 8290.4) | 6807.9 (5784.8 to 7848) | 6486.8 (5549.7 to 7529) | 6222 (5364.7 to 7134.9) | 6091.6 (5326.8 to 6921.7) | 5982.4 (5259.9 to 6740.6) | 5839.3 (5171 to 6543.6) | 5619.3 (4982.7 to 6259.5) | 5495.1 (4809.8 to 6195.1) | 5543.2 (4831.1 to 6324.4) | 5744.2 (5003.4 to 6511.8) | 6001.7 (5166.6 to 6880.6) | 6164.9 (5292.2 to 7084.5) | 6202.4 (5380.2 to 7071.2) | 6140.2 (5391.3 to 6951.5) | 6099.3 (5432.8 to 6838) | 6002.3 (5425.2 to 6678.6) | 5723.7 (5176.1 to 6254.7) | 5096.7 (4678.7 to 5490.6) | 3954.3 (3643.5 to 4272.2) | 3241.7 (2942.9 to 3532.7) | 2732 (2449.6 to 3004.1) | 2351.2 (2071 to 2622.6) | 1580.3 (1408.1 to 1748.6) | 1093.3 (921.2 to 1267.7) |
|  |  | Mental disorders | 1352.7 (972.9 to 1769.7) | 1378.5 (989.9 to 1808.4) | 1404.7 (1010.9 to 1843.3) | 1432.3 (1030.4 to 1883.9) | 1462.3 (1047 to 1923.7) | 1496.5 (1074.4 to 1972.8) | 1533.8 (1100.1 to 2022.4) | 1573.1 (1132.4 to 2078.7) | 1608.1 (1158.2 to 2120.1) | 1635.1 (1173.9 to 2153.2) | 1653.5 (1188.1 to 2180.4) | 1667.5 (1195.9 to 2201.9) | 1679.3 (1208.7 to 2221.7) | 1688.5 (1212.4 to 2233.2) | 1692.6 (1220.2 to 2242.3) | 1689 (1215.1 to 2240) | 1675.7 (1206.7 to 2214.5) | 1652.6 (1190.7 to 2185.4) | 1622 (1169.8 to 2142.2) | 1588.6 (1147.7 to 2098.2) | 1554.2 (1117.9 to 2050.5) | 1522.1 (1098.7 to 2009.1) | 1493.6 (1076.1 to 1967.1) | 1469.1 (1058.5 to 1931.2) | 1446.5 (1044.3 to 1905.4) | 1427.2 (1026.4 to 1882.2) | 1411.4 (1013.8 to 1855.8) | 1404 (1009.7 to 1842.9) | 1406.4 (1010.7 to 1840.3) | 1418.4 (1020.6 to 1861.1) | 1711.6 (1219.6 to 2281.3) | 1711.4 (1214.4 to 2294.1) |
|  |  | Musculoskeletal disorders | 323.7 (219.2 to 448.5) | 333.3 (224 to 461.2) | 343.2 (232.2 to 476) | 353.3 (238.5 to 487.1) | 365 (245.8 to 504.2) | 378.2 (253 to 522) | 392 (262.2 to 543.8) | 404.4 (271.1 to 557.9) | 414.6 (278.3 to 573) | 422.3 (282.3 to 582.6) | 428.3 (285.4 to 596.2) | 433.4 (290.6 to 601.2) | 438.4 (297.7 to 608.8) | 442.7 (301.1 to 616) | 445.4 (304 to 617.4) | 445.2 (304 to 618.8) | 443.1 (303.6 to 616.6) | 438.2 (300.7 to 611.4) | 431.3 (293.2 to 598.7) | 422.5 (287.9 to 584.4) | 411.7 (281 to 572) | 401.4 (275.4 to 556) | 391.5 (268.4 to 539.5) | 382.4 (262.2 to 527.1) | 372.8 (255.7 to 511.8) | 364 (248.8 to 498.6) | 356.8 (242.9 to 491.2) | 353.2 (240.6 to 485.1) | 352.7 (239.4 to 488.5) | 355.7 (242.2 to 494.4) | 361.1 (242.3 to 499.6) | 354.7 (240.6 to 493.1) |
|  |  | Neglected tropical diseases and malaria | 198.9 (88.3 to 589.9) | 227.1 (87.1 to 978.1) | 170.1 (82.5 to 544.3) | 165.1 (78.9 to 589) | 142.1 (76.5 to 341.2) | 129.4 (73.6 to 295.8) | 123.9 (70.5 to 300.2) | 106.7 (66.6 to 213) | 99.7 (63.7 to 187.3) | 105.1 (63.1 to 221.1) | 90.6 (59.1 to 160.3) | 87.9 (58.8 to 151) | 87.5 (58.1 to 140.9) | 89.7 (57.3 to 147.1) | 82.7 (55.7 to 122.3) | 83.6 (56.6 to 120.7) | 81.6 (55.5 to 116.7) | 81.7 (56.2 to 115.3) | 81.7 (56.6 to 114.7) | 81.4 (57.2 to 113.8) | 81.6 (56.3 to 112.9) | 81.3 (56.9 to 112.6) | 81 (57 to 112.7) | 80.6 (57 to 112.5) | 79 (55.7 to 110.2) | 76.4 (52.4 to 108.1) | 72.9 (49.5 to 103.3) | 69.6 (47.3 to 99.3) | 66.7 (45.2 to 94.8) | 64.6 (44 to 92.6) | 61.6 (41.5 to 89.4) | 58.8 (39.5 to 84.8) |
|  |  | Neoplasms | 717 (560.5 to 907) | 695.5 (534.6 to 866.5) | 682.7 (537.1 to 831.2) | 670.7 (527.5 to 810.3) | 659.5 (529.9 to 789.9) | 646 (523.8 to 761.8) | 631.9 (517.6 to 735.8) | 619.3 (510.2 to 712.3) | 607 (500 to 697.4) | 595.8 (493.3 to 679.1) | 587.7 (495.9 to 666) | 580.1 (490 to 653) | 571.5 (485.9 to 641) | 565.6 (481.6 to 636.1) | 564.6 (473.2 to 636.8) | 564.1 (470.3 to 637.3) | 569 (469.4 to 641.9) | 576.6 (471.7 to 650.9) | 586.2 (474.3 to 665.7) | 593.3 (477.2 to 669.9) | 590.4 (467.7 to 667.2) | 594.5 (466.7 to 670.6) | 606.6 (475.3 to 685.9) | 618.3 (482.1 to 695.2) | 583.7 (452.2 to 656.4) | 515 (388.6 to 593.2) | 468 (345.4 to 550.7) | 446.3 (325.8 to 536.3) | 426.5 (306.7 to 523.8) | 411.4 (293.3 to 510.4) | 401.2 (285.4 to 488) | 380.2 (272 to 463.7) |
|  |  | Neurological disorders | 687.1 (357.5 to 1194.3) | 687.7 (356 to 1205.2) | 692.2 (351.7 to 1215.4) | 697.8 (351.5 to 1229.9) | 703.6 (349.9 to 1241) | 710.5 (344 to 1251.7) | 715.1 (337.8 to 1276.2) | 716.2 (334.9 to 1294.6) | 714 (329.4 to 1293) | 710 (327.5 to 1287.1) | 708.1 (327.9 to 1285) | 708.3 (326.2 to 1287.6) | 707.4 (325.9 to 1283.8) | 706.9 (324.1 to 1288.2) | 707.4 (321.3 to 1280.3) | 705.1 (321.6 to 1276.4) | 702.4 (323.2 to 1269.7) | 698.3 (318 to 1264) | 693 (317.1 to 1256.9) | 686 (313.7 to 1236.5) | 673.8 (310.6 to 1207.6) | 660.8 (310.7 to 1180.4) | 647.9 (309 to 1151.7) | 635.3 (306 to 1116.6) | 615.5 (297.7 to 1081.6) | 602.7 (292.5 to 1056.8) | 582.6 (273 to 1036.8) | 573.1 (262.3 to 1042.1) | 567.9 (252.4 to 1046.6) | 566.8 (242.9 to 1052) | 562.3 (228.1 to 1050) | 577 (231.2 to 1099.8) |
|  |  | Nutritional deficiencies | 941.9 (708.6 to 1259.1) | 865.9 (646.3 to 1159.3) | 807.7 (599.6 to 1083.9) | 757 (562.3 to 1016.3) | 711.9 (526.7 to 956.9) | 670.5 (489.5 to 901) | 632.8 (457.1 to 850.9) | 602.9 (434.8 to 817.5) | 576.9 (414.7 to 790.4) | 556.1 (399.7 to 763.2) | 538.6 (386.5 to 740.6) | 524.7 (375 to 724.4) | 510.7 (364.9 to 701.4) | 499.2 (355.6 to 686.6) | 487.5 (342.5 to 674.5) | 479.6 (331.7 to 663.3) | 473.5 (326 to 655.7) | 470.9 (324 to 650.9) | 471.4 (324.6 to 650.9) | 470.5 (323.9 to 653.3) | 467.7 (317.7 to 652.4) | 463 (315.1 to 653.7) | 459.7 (312.1 to 657) | 456.1 (312.3 to 658.9) | 446.9 (306.7 to 652.5) | 433.7 (291.6 to 636.9) | 415.8 (276.1 to 620.8) | 397.9 (260 to 600.2) | 380.5 (245.9 to 579.7) | 366.5 (235.6 to 562.9) | 353 (223.4 to 544.6) | 338 (212.8 to 524.3) |
|  |  | Other infectious diseases | 1281 (693.6 to 2475.8) | 1017.3 (587.1 to 1943.9) | 807.4 (510 to 1411.7) | 681.7 (449.7 to 1122.8) | 586.2 (415.5 to 943.4) | 519 (379.9 to 815) | 474.2 (351.1 to 716.3) | 431.4 (328.8 to 632.5) | 406.8 (305.4 to 594) | 372.8 (293.8 to 506.9) | 357.6 (286.8 to 485.3) | 335.1 (265.6 to 437.7) | 318.7 (255.3 to 419) | 308.1 (250.6 to 396.8) | 294.1 (236.4 to 384) | 290.9 (237.3 to 376.8) | 292.8 (237.3 to 383.9) | 299 (243.9 to 387.2) | 306.4 (249.4 to 394.2) | 314.8 (251.4 to 399.1) | 314.2 (253.4 to 416.2) | 314.2 (250.6 to 421.9) | 315.1 (253.6 to 410.1) | 317.3 (257.4 to 420.8) | 288.5 (223.9 to 397.2) | 258.6 (197.3 to 371.9) | 223 (164.1 to 322.2) | 201.9 (152.2 to 294.3) | 184.3 (139.9 to 251.5) | 168.2 (126.6 to 234.7) | 138.2 (103.5 to 212.3) | 114.5 (87.5 to 165.6) |
|  |  | Other non-communicable diseases | 8849.6 (5443.7 to 10750.6) | 8066.7 (5186.5 to 9711.5) | 7530.6 (5026.4 to 9072.8) | 7067.3 (4863.5 to 8564.3) | 6593.3 (4654 to 7935.3) | 6115 (4381.8 to 7420.6) | 5701 (4158.8 to 6905) | 5370.6 (4052.4 to 6453.8) | 5091.2 (3926.9 to 6096.4) | 4859.9 (3803.4 to 5806.6) | 4677.8 (3721 to 5571.3) | 4572.6 (3718.7 to 5453) | 4512.6 (3685.6 to 5360.5) | 4451.7 (3685.1 to 5248.6) | 4380.2 (3668.2 to 5101.2) | 4299.5 (3648.4 to 4980.7) | 4312 (3633.9 to 4947.8) | 4419.3 (3790 to 5105.3) | 4596.2 (3951.5 to 5266.2) | 4735.5 (4085 to 5391.2) | 4781.2 (4174.7 to 5434.2) | 4758.9 (4188.9 to 5399.7) | 4718.1 (4206.2 to 5338.1) | 4618.6 (4172.9 to 5185.2) | 4342.1 (3944.7 to 4845.8) | 3842.5 (3546.4 to 4279.7) | 3137.9 (2892 to 3489.7) | 2728.8 (2477.4 to 3057.4) | 2420.2 (2147.4 to 2760.4) | 2179 (1917.1 to 2529.9) | 1562.3 (1376.1 to 1821) | 1212.4 (1020.6 to 1445.9) |
|  |  | Respiratory infections and tuberculosis | 3172.1 (2652.5 to 4228.3) | 2745.4 (2259.2 to 3678.8) | 2448.3 (2008.8 to 3289.9) | 2207.1 (1829.1 to 2904.5) | 2000.5 (1641.3 to 2608.6) | 1816.8 (1521.4 to 2272.9) | 1662.1 (1406 to 2027.6) | 1541.5 (1309.3 to 1853.2) | 1436.5 (1218.9 to 1688.2) | 1344.8 (1151.7 to 1572.9) | 1262.8 (1090.2 to 1481.5) | 1203.3 (1041.8 to 1414.1) | 1172.9 (1010.9 to 1367.8) | 1152.8 (992.2 to 1340.5) | 1143.6 (973.5 to 1329.6) | 1132.7 (963.9 to 1327.8) | 1139.7 (972.7 to 1345.7) | 1158.4 (987.6 to 1363.7) | 1182.4 (1016.5 to 1380.8) | 1191.2 (1034.7 to 1386.5) | 1172.5 (1025 to 1348.7) | 1140.7 (1000.3 to 1306) | 1113 (992.2 to 1255.7) | 1070.1 (956.1 to 1190.7) | 969.9 (870.5 to 1079.4) | 817.7 (730 to 914.5) | 649.4 (553.3 to 739.2) | 557.9 (462.5 to 649.7) | 492.1 (404.6 to 579.2) | 444 (361.8 to 532.8) | 611.3 (513.9 to 726.6) | 586.6 (473.5 to 773.1) |
|  |  | Self-harm and interpersonal violence | 533.8 (474.6 to 578.4) | 505.8 (430.8 to 546.4) | 465.4 (409.1 to 505.8) | 459.6 (399.4 to 499.5) | 458.3 (405.7 to 496.5) | 439.1 (383.7 to 476.4) | 450.1 (394.6 to 487.3) | 454.4 (399.9 to 489.7) | 461.9 (411 to 496.8) | 480.4 (420.2 to 517.8) | 474.9 (418.2 to 509.5) | 495.4 (437.4 to 531.2) | 478.5 (417.7 to 511.2) | 491.9 (433.5 to 523.7) | 503.2 (450 to 537) | 482.3 (431.7 to 518) | 479.6 (432.1 to 513.1) | 479.7 (436.7 to 515.8) | 488 (448 to 523.6) | 478.1 (441.8 to 512) | 454.9 (421.7 to 485.9) | 424.2 (393.3 to 453.2) | 411.4 (382.2 to 438.1) | 410.7 (380.6 to 437.2) | 383.4 (359.5 to 406.7) | 361.2 (341.4 to 382.2) | 355.9 (335.9 to 376.1) | 336.3 (317.8 to 357.5) | 305.4 (285.4 to 325.6) | 266.6 (246.3 to 289.6) | 302.1 (281.6 to 322.9) | 283.8 (262.1 to 306.8) |
|  |  | Sense organ diseases | 192.7 (132.7 to 270.4) | 194.6 (133.8 to 273.4) | 196.3 (134.8 to 276.6) | 198.2 (136.3 to 278.8) | 200.3 (138 to 282.4) | 202.7 (139.6 to 285.7) | 205.4 (141.4 to 289.2) | 208.2 (143.6 to 292.6) | 210.7 (144.9 to 295.7) | 212.6 (146.4 to 297.6) | 213.8 (147.5 to 299.2) | 214.4 (147 to 301.5) | 214.3 (147.5 to 302.1) | 214.1 (147.7 to 302.4) | 213.5 (147.2 to 302.8) | 212.6 (146.6 to 302.7) | 211.2 (145.1 to 298.5) | 209.4 (144.1 to 296.4) | 207.1 (142.5 to 291.3) | 204.3 (141.1 to 285.8) | 201.5 (138.1 to 280.9) | 199 (136.8 to 276.2) | 196.9 (135.7 to 274.9) | 195.1 (134.3 to 273.5) | 193.6 (133.4 to 271.8) | 192.2 (132.6 to 270) | 190.7 (130.7 to 267.8) | 189.3 (130.4 to 266.4) | 188.6 (129.3 to 265.4) | 189.3 (130.2 to 267.4) | 191.5 (131.6 to 269.2) | 192.7 (131.7 to 271) |
|  |  | Skin and subcutaneous diseases | 471.6 (307.5 to 678.1) | 474.5 (309.6 to 681.8) | 477.5 (310.4 to 687.8) | 480.6 (313.7 to 690) | 484 (315.4 to 696.7) | 487.5 (319 to 704.3) | 491.2 (321.5 to 711.7) | 495.2 (324.7 to 717.2) | 498.7 (328 to 723.3) | 501.8 (330 to 725.5) | 504.5 (333.2 to 729.7) | 507.2 (335 to 735.2) | 510.4 (336.4 to 740.3) | 513.5 (339.2 to 746.4) | 516.2 (340.9 to 750.7) | 518.1 (341.3 to 755.1) | 519.2 (342.7 to 760.3) | 519.6 (341.6 to 760.4) | 519.2 (343.3 to 758.3) | 518.3 (341.4 to 756.6) | 516.8 (340.5 to 754.1) | 515.2 (339.9 to 752) | 513.6 (337.1 to 749.7) | 512.1 (336.1 to 747.9) | 510.4 (334.8 to 745.3) | 509 (332.4 to 744.8) | 508.2 (332 to 743.4) | 509.1 (331.6 to 743.3) | 510.8 (333 to 745.9) | 513.1 (335.9 to 751.5) | 514.9 (337.7 to 752.1) | 515.8 (336.6 to 747.7) |
|  |  | Substance use disorders | 52.2 (41.5 to 64.1) | 53.8 (43 to 65.7) | 56.3 (45.5 to 68.7) | 58.1 (46.4 to 70.6) | 61.2 (49.9 to 74.7) | 65.7 (53 to 79.5) | 71.4 (57.8 to 86.9) | 78 (62.3 to 93.8) | 84 (67.8 to 101.3) | 89.4 (72.6 to 107.4) | 94 (75.9 to 113.1) | 98.1 (79.6 to 118.4) | 101.9 (82.3 to 123.8) | 105.1 (84.9 to 126.3) | 107.4 (87.4 to 131.9) | 108.6 (88.4 to 131.7) | 110 (89.5 to 135.5) | 109.1 (89.1 to 132.7) | 106 (86.1 to 128.8) | 100.5 (81.6 to 122.6) | 94.6 (75.9 to 115.2) | 88.9 (71.4 to 107.9) | 83.9 (67.9 to 100.9) | 78.3 (62.9 to 94) | 74 (59.5 to 88.9) | 69.2 (55.4 to 83.7) | 66.9 (53.5 to 80.3) | 66.3 (53.1 to 79.8) | 65.6 (51.7 to 78.7) | 65.8 (51.8 to 80) | 65.7 (51.9 to 79.1) | 66.1 (51.9 to 80) |
|  |  | Transport injuries | 3977.2 (3482.9 to 4647) | 3770.4 (3302.2 to 4303.9) | 3630.2 (3234.8 to 4141.2) | 3497.5 (3097 to 3959.7) | 3381.6 (3010.7 to 3809.8) | 3250.8 (2907.5 to 3674.7) | 3137.3 (2824.5 to 3530.7) | 3055.5 (2772.8 to 3355.1) | 2975 (2726 to 3254) | 2917.7 (2681.9 to 3182.4) | 2859.4 (2631.8 to 3088.7) | 2817.3 (2586 to 3047) | 2790.7 (2571.6 to 3019.8) | 2758.8 (2542.1 to 2982.4) | 2714.5 (2517.8 to 2935.1) | 2630.1 (2432.4 to 2846) | 2544 (2360.3 to 2751.5) | 2472.6 (2294.6 to 2673.5) | 2415.6 (2245.1 to 2620.9) | 2342.7 (2182.9 to 2543.7) | 2206.2 (2060.6 to 2393.7) | 2117.1 (1985.8 to 2292.3) | 2053.3 (1930.2 to 2207) | 1986.6 (1883.9 to 2126.5) | 1731.2 (1657.1 to 1842.8) | 1401.7 (1323.6 to 1496.8) | 1200.7 (1122.9 to 1291.9) | 1102.4 (1015.2 to 1190.9) | 1021 (928.4 to 1110.5) | 955.2 (859.5 to 1045.2) | 898.1 (819.1 to 977.3) | 825.8 (744 to 908) |
|  |  | Unintentional injuries | 9642.8 (8913.5 to 10454.7) | 2757.1 (2330.4 to 3155.6) | 2550 (2182.8 to 2952.2) | 2553.5 (2189 to 2912) | 2341.8 (2001.1 to 2686.2) | 2237.6 (1914.3 to 2559.7) | 2142.2 (1832 to 2441.5) | 2466.3 (2184 to 2740.8) | 2035 (1747.2 to 2283.8) | 1969.8 (1690.4 to 2194.6) | 1904.9 (1635.6 to 2104.3) | 1944.7 (1684.4 to 2153) | 1842.7 (1595.1 to 2037.7) | 5253.8 (4886.9 to 5687.7) | 1775.5 (1563.6 to 1952.1) | 1704.2 (1506.4 to 1873.4) | 1551.7 (1372.7 to 1709) | 1490.6 (1326.3 to 1636) | 1450.9 (1300.8 to 1583.3) | 1388.5 (1255.3 to 1513.1) | 1330.2 (1208 to 1455.8) | 1254.3 (1145.5 to 1359.7) | 1255.3 (1158.3 to 1354.3) | 1183.5 (1091.6 to 1277.3) | 1071.4 (1004 to 1151.2) | 929.7 (877 to 998.2) | 793.8 (740.5 to 865.2) | 818.8 (767.3 to 891.2) | 666 (610.9 to 734.8) | 636.4 (581.6 to 708) | 577.6 (531 to 642.6) | 507 (458.8 to 570.6) |
| DALYs (Disability-Adjusted Life Years) | Females | Causes | Year |  |  |  |  |  |  |  |  |  |  |  |  |  |  |  |  |  |  |  |  |  |  |  |  |  |  |  |  |  |  |  |
|  |  |  | 1990 | 1991 | 1992 | 1993 | 1994 | 1995 | 1996 | 1997 | 1998 | 1999 | 2000 | 2001 | 2002 | 2003 | 2004 | 2005 | 2006 | 2007 | 2008 | 2009 | 2010 | 2011 | 2012 | 2013 | 2014 | 2015 | 2016 | 2017 | 2018 | 2019 | 2020 | 2021 |
|  |  | Cardiovascular diseases | 890.5 (756.8 to 1162) | 826.3 (712.2 to 1054.4) | 784.6 (680.4 to 1001.6) | 746.8 (649.5 to 943.7) | 712 (625.8 to 869.2) | 679.7 (596.6 to 815.1) | 650.7 (576.6 to 767.3) | 627.4 (557.6 to 729) | 606.3 (538.6 to 696.2) | 587.2 (521.7 to 665.3) | 572.2 (512.2 to 641.5) | 559.1 (499.2 to 629.6) | 555.8 (500.6 to 623.9) | 554 (499.1 to 621.4) | 552.8 (493.2 to 627.7) | 542.6 (483.1 to 614.3) | 538 (480.9 to 604.1) | 534.9 (475.6 to 600.2) | 541.8 (485.1 to 608.9) | 546.9 (492.8 to 609.2) | 541 (487.1 to 600.9) | 538.3 (487.7 to 598) | 537.3 (491.9 to 590.7) | 533 (488.7 to 584.3) | 486.3 (449.5 to 527.1) | 419.5 (384.6 to 456.8) | 356.9 (317.3 to 397.4) | 325.3 (282.8 to 363.2) | 302.8 (258.4 to 340.7) | 286 (240.5 to 324) | 261.5 (223.3 to 301.1) | 240.7 (204.9 to 279.1) |
|  |  | Chronic respiratory diseases | 318.1 (211.4 to 445.5) | 306.9 (205.5 to 427) | 299.1 (205.8 to 418.4) | 291.4 (203.9 to 403.4) | 285.1 (199.9 to 394.6) | 278.8 (197.8 to 384.8) | 272.9 (196.6 to 376.3) | 268.2 (194.9 to 369.2) | 264 (190.9 to 359.4) | 260.8 (190.4 to 354.6) | 258.3 (186.6 to 351.5) | 255.4 (185.4 to 347.8) | 254.4 (186.3 to 346) | 253.4 (187.2 to 343) | 252.9 (188.5 to 341.8) | 251.1 (186.8 to 341.5) | 249.5 (186.3 to 339.1) | 247.2 (184.5 to 337.6) | 246.8 (184.2 to 338) | 246.1 (182.7 to 333.4) | 243.8 (182 to 328.4) | 241.9 (182.2 to 325.8) | 239.4 (179.7 to 322.1) | 235.6 (177.2 to 319.8) | 224.4 (166.7 to 305.9) | 212.4 (153 to 295.1) | 200.6 (141.9 to 283.3) | 193.7 (137.1 to 273.9) | 188.6 (131.2 to 266.6) | 185 (127.8 to 262.8) | 183.6 (125.7 to 268.2) | 177.5 (121.5 to 260.7) |
|  |  | Diabetes and kidney diseases | 112.5 (92.1 to 140.2) | 107.6 (87.8 to 133.4) | 105.1 (85.6 to 130.5) | 103.5 (84 to 128.4) | 101.6 (83.6 to 127.7) | 100.5 (83.5 to 124.3) | 100.2 (82.7 to 123.4) | 99.6 (82.4 to 121.8) | 99.1 (82.9 to 120.5) | 97.9 (82.2 to 118.9) | 98.2 (83.8 to 117.8) | 98.4 (83.8 to 116.2) | 99.9 (86 to 118.1) | 101.6 (87.6 to 118.9) | 105.3 (91.9 to 121.8) | 108.5 (94.9 to 125.8) | 112.2 (98.2 to 129.5) | 114.2 (99.6 to 131.4) | 116.8 (101.7 to 134.7) | 119.1 (102.9 to 136.8) | 119.6 (103.4 to 136.5) | 120.5 (104 to 136.3) | 120.9 (104 to 136.8) | 121.4 (103.9 to 136.7) | 113.2 (98.1 to 127.2) | 102.1 (88.4 to 113.6) | 92.2 (80.5 to 103) | 87.1 (75.7 to 97.8) | 82.9 (71.2 to 93.6) | 80.1 (68.7 to 90.4) | 74 (63.7 to 83.7) | 68.7 (58.7 to 77.8) |
|  |  | Digestive diseases | 450 (351.7 to 551.7) | 416.6 (322.9 to 512) | 396.4 (304.5 to 497.3) | 374.7 (298.4 to 460) | 361.6 (283.5 to 446.3) | 344.3 (274.1 to 418.3) | 324.1 (269.6 to 380.9) | 312.9 (259.4 to 366.1) | 302.1 (254.3 to 349) | 295.1 (245.9 to 346.7) | 281.4 (240.5 to 324.2) | 266.9 (231.3 to 306) | 263.5 (225.8 to 303.7) | 261.6 (224.2 to 300.1) | 270.8 (235.4 to 309.8) | 266.7 (231.2 to 305.3) | 263.9 (227.7 to 300.8) | 264.9 (229.2 to 303.3) | 268.4 (232.2 to 307.6) | 269 (233.1 to 309) | 265.5 (233.2 to 302.4) | 261.6 (230.1 to 297.9) | 257.1 (227 to 291.9) | 249.8 (221.1 to 282.3) | 234.2 (210.6 to 262.8) | 210.1 (184 to 241.1) | 187.1 (154.8 to 222.6) | 173.8 (140.6 to 211.8) | 162.8 (128.2 to 200.8) | 154 (120.7 to 193.1) | 137.1 (108.8 to 171.1) | 122 (98.2 to 151.4) |
|  |  | Enteric infections | 1404.4 (999.9 to 2264.5) | 1205.4 (875 to 1918.4) | 1047.6 (769.2 to 1646.7) | 935.5 (695 to 1430.9) | 855 (634.6 to 1252.9) | 801.2 (608.6 to 1128.7) | 724.2 (556.5 to 998.7) | 682.3 (531.6 to 916.7) | 639.4 (503.8 to 837) | 599.6 (475.5 to 768.8) | 563.6 (453.1 to 707.5) | 498.1 (398.7 to 627.8) | 464.1 (372.3 to 584.1) | 435.3 (352.8 to 543.5) | 414.4 (335.4 to 516.6) | 399.7 (324.2 to 496.3) | 386.8 (312.4 to 476.1) | 373.6 (304.8 to 461.1) | 358.4 (292.5 to 440) | 342.3 (280.6 to 421.5) | 327 (269 to 403.7) | 318.9 (263.4 to 396) | 308.1 (252.9 to 380.7) | 301.9 (249.3 to 366.4) | 285.9 (236.5 to 349.6) | 273.2 (222.8 to 335.9) | 250.9 (197.8 to 309.7) | 233.3 (179.4 to 295.2) | 217.7 (164 to 276.4) | 206.1 (153.6 to 260.7) | 173.3 (129.5 to 224.7) | 147 (107.2 to 194) |
|  |  | HIV/AIDS and sexually transmitted infections | 59.9 (21.4 to 124.9) | 55.5 (20.1 to 114.9) | 51.9 (18.9 to 106.9) | 49.7 (18.4 to 103.1) | 48.1 (18 to 101) | 46.3 (17.7 to 97.6) | 45 (17.1 to 94.1) | 44.2 (17 to 91.9) | 43.7 (17 to 92) | 43.5 (17.1 to 90.9) | 43.5 (17.3 to 88.6) | 43.8 (17.5 to 90.9) | 43.5 (17.1 to 90.2) | 44.7 (18.6 to 91.3) | 45.8 (20 to 92.8) | 46.8 (20.2 to 94.7) | 49.1 (21.2 to 99.9) | 56.2 (25.9 to 108.8) | 61.1 (28.8 to 117.5) | 66.1 (31.6 to 126.9) | 69.5 (33 to 135.3) | 66.2 (29.1 to 133.7) | 69.3 (31.7 to 136.7) | 76 (38.3 to 143.9) | 77.4 (40.4 to 140.5) | 77 (41.4 to 138.3) | 68.8 (35.2 to 127.8) | 66 (34.6 to 125.4) | 62.1 (32.6 to 117.8) | 58 (30.7 to 112.1) | 53.4 (28.6 to 98.8) | 49.3 (26.5 to 90.6) |
|  |  | Maternal and neonatal disorders | 10755.1 (9098.1 to 13342.6) | 9762.7 (8272.9 to 12296.2) | 9044.6 (7628.4 to 11346.2) | 8388.8 (7047.1 to 10458) | 7715.5 (6471.1 to 9607.5) | 7111.6 (5988.3 to 8761.3) | 6639.6 (5568.4 to 8184.5) | 6273.7 (5274 to 7618.9) | 5959.6 (4976 to 7218.8) | 5680.4 (4857.7 to 6797.4) | 5450.9 (4705.8 to 6386.8) | 5339.7 (4631.6 to 6148.8) | 5243.8 (4570.1 to 5990) | 5114.8 (4511.8 to 5817.6) | 4915.8 (4329.6 to 5584.3) | 4805 (4248.8 to 5431.8) | 4844.7 (4233.2 to 5516.4) | 5018.4 (4373.1 to 5798.1) | 5244.5 (4477.8 to 6089.8) | 5391.2 (4637 to 6249.3) | 5431.2 (4740 to 6295.8) | 5389.8 (4699.6 to 6181.1) | 5368.3 (4732.9 to 6111.3) | 5297.8 (4707.2 to 5970.6) | 5062.9 (4519.4 to 5587.9) | 4521.1 (4113.8 to 4947.8) | 3517.1 (3188.9 to 3826.9) | 2895.4 (2583 to 3171.7) | 2449.6 (2167.9 to 2700.2) | 2115.9 (1851.4 to 2353.6) | 1438.2 (1269.7 to 1604.9) | 1004.7 (836.9 to 1184.3) |
|  |  | Mental disorders | 1381.7 (983.5 to 1817.7) | 1409.1 (1004.7 to 1862) | 1438.3 (1022.9 to 1893.7) | 1470 (1045.6 to 1945.8) | 1505.6 (1069.7 to 1992.7) | 1545.8 (1098.8 to 2048.4) | 1588.5 (1129 to 2107.6) | 1632.3 (1160.4 to 2161) | 1670.4 (1189.2 to 2215.5) | 1699.9 (1204.8 to 2261.7) | 1720.8 (1220.2 to 2287.5) | 1737.3 (1230.9 to 2304) | 1752.1 (1240.6 to 2331.4) | 1764.7 (1248.8 to 2345.1) | 1771 (1256.1 to 2354.5) | 1768.4 (1253.1 to 2343.8) | 1756.2 (1248.2 to 2339.7) | 1734.9 (1234.9 to 2307.7) | 1706.3 (1214.3 to 2272.3) | 1673.1 (1191.8 to 2226.2) | 1636.8 (1161.9 to 2173) | 1602.1 (1142.1 to 2146.2) | 1571.5 (1111.2 to 2098.8) | 1544.9 (1097.2 to 2059.5) | 1519.9 (1074 to 2026.9) | 1496.7 (1056.6 to 1999.1) | 1476.4 (1045.3 to 1962.4) | 1465.6 (1040.5 to 1945.7) | 1465.9 (1042.3 to 1937.6) | 1478.4 (1052 to 1956.1) | 1842.1 (1294.2 to 2459.8) | 1831.6 (1289.2 to 2460.6) |
|  |  | Musculoskeletal disorders | 442 (299.6 to 612.3) | 455.3 (305.3 to 630.3) | 469.1 (317 to 649.7) | 483.9 (326.4 to 668.9) | 500.5 (336.5 to 692.7) | 519 (349.4 to 717.2) | 538.2 (364.1 to 745.7) | 555.7 (376.2 to 768.3) | 570.6 (387.2 to 789) | 581.8 (393.2 to 807.1) | 590.6 (398.2 to 819.8) | 597.5 (405.1 to 831.6) | 604.2 (413.8 to 842.9) | 609.7 (418.3 to 854.2) | 613.1 (420.3 to 858.8) | 612.6 (422.2 to 856.7) | 610.3 (420.5 to 852.2) | 603.9 (415.4 to 844.6) | 594.6 (407.9 to 829.4) | 582.6 (397.7 to 807.9) | 567.6 (385.2 to 786.3) | 553.3 (379.7 to 769.1) | 539.2 (369.5 to 743.8) | 526.8 (361.3 to 728.2) | 513.1 (354.3 to 712.2) | 501.1 (344.9 to 690.5) | 491 (335.5 to 677.3) | 486.5 (332 to 673.5) | 485.7 (329.9 to 673.1) | 489.7 (332.8 to 675.4) | 496.7 (336.9 to 683.4) | 483.1 (328.1 to 668.3) |
|  |  | Neglected tropical diseases and malaria | 186.3 (83.3 to 548) | 214.3 (83.5 to 929.4) | 164.3 (78.4 to 544) | 159.8 (75.2 to 545.9) | 140.3 (75.4 to 337.1) | 128.8 (72.4 to 289.2) | 124.1 (70.6 to 289.8) | 108 (67.1 to 189.8) | 101.9 (65.5 to 163.9) | 108.2 (65.1 to 216) | 93.7 (61.9 to 145.2) | 91.7 (61.2 to 139.8) | 92.2 (61.9 to 133.7) | 94.9 (61.7 to 147.8) | 88.2 (59.6 to 124.3) | 89.7 (60.6 to 127.5) | 87.8 (59.9 to 122.3) | 87.9 (60.1 to 122.4) | 87.9 (61.3 to 122.6) | 87.7 (60.3 to 122.4) | 87.9 (61.1 to 125.5) | 87.7 (61.2 to 124.3) | 87.5 (60.6 to 123.6) | 87 (61.4 to 122.9) | 85.6 (59.8 to 121.2) | 83.4 (57.1 to 119.5) | 80.5 (55.2 to 116.7) | 77.7 (52.7 to 113.8) | 75.2 (50.5 to 111.2) | 73.5 (49.8 to 109) | 71.1 (47.6 to 105.6) | 68.6 (45.8 to 101.9) |
|  |  | Neoplasms | 688.3 (490.8 to 843.8) | 666 (464.2 to 807.8) | 653.5 (475.1 to 787.5) | 640.8 (464.6 to 755) | 629.4 (462 to 746.6) | 616 (459 to 716) | 601.6 (455.2 to 693.5) | 589 (451.5 to 671.2) | 576.1 (449.1 to 660.1) | 563.8 (438.6 to 644.9) | 554 (436.5 to 621.4) | 544.1 (428.8 to 614.7) | 533.2 (425.9 to 600.9) | 523.9 (421 to 588.6) | 519.4 (416.9 to 585.9) | 514.8 (410.5 to 580.7) | 517.9 (408.9 to 582.2) | 523 (404.8 to 589) | 530.3 (405.8 to 596) | 537.5 (404.3 to 603) | 536.8 (397.8 to 604.6) | 542.5 (396 to 605.3) | 554.1 (398 to 618.4) | 565.7 (402.6 to 632.3) | 531.6 (376.5 to 593.1) | 470.8 (335.9 to 536.6) | 427.6 (300.2 to 502.4) | 408.6 (282.5 to 489.3) | 391.7 (267.1 to 475.8) | 378.8 (256.6 to 463.6) | 375.4 (256.7 to 452.4) | 362.2 (244.7 to 436.3) |
|  |  | Neurological disorders | 714.7 (358.9 to 1277.9) | 716.4 (349.7 to 1290.2) | 722 (345 to 1298.5) | 729.2 (339.1 to 1318.4) | 736.7 (330.3 to 1356.5) | 745.1 (324.6 to 1390) | 749.7 (329.2 to 1410.8) | 748.4 (331.2 to 1402.5) | 743.4 (328 to 1390.5) | 737 (329.8 to 1374) | 735.3 (325.4 to 1368.2) | 737.8 (323.9 to 1380.3) | 739.2 (316.6 to 1389.8) | 741.5 (318.4 to 1401.3) | 743.8 (319.1 to 1407.5) | 741.7 (319.5 to 1403.9) | 740.5 (325.5 to 1392) | 738.4 (316.2 to 1390.1) | 736 (311.2 to 1387.2) | 730.5 (305.2 to 1366.4) | 717.7 (298.7 to 1339.6) | 703.3 (301 to 1309.8) | 689.4 (300.9 to 1269.1) | 674.8 (296.4 to 1243.5) | 653.7 (287 to 1210) | 634.8 (272.4 to 1171.5) | 616 (257.2 to 1155.5) | 608.4 (246.9 to 1149.5) | 606.3 (236.9 to 1170.7) | 607.2 (230.6 to 1190.2) | 605.7 (216.8 to 1183.4) | 622.7 (212 to 1240.6) |
|  |  | Nutritional deficiencies | 969.2 (703.9 to 1331.9) | 900.5 (649.3 to 1232.7) | 847.9 (607.9 to 1161.4) | 802.1 (565.7 to 1102) | 761.5 (528.6 to 1051.4) | 724.7 (495 to 1005.6) | 692.6 (471 to 951.4) | 667.6 (455.1 to 924.2) | 646.3 (438.2 to 905.7) | 629.7 (427.5 to 888) | 615.5 (419.4 to 880.8) | 604.8 (408.8 to 864.8) | 593.9 (401.6 to 848.9) | 584.7 (392.6 to 841.9) | 574.2 (378.5 to 833.4) | 566.9 (374.7 to 826.6) | 560.9 (371.6 to 811.2) | 558.2 (370.4 to 799.5) | 557.8 (370.3 to 795) | 557.3 (369.6 to 799.4) | 554 (368.7 to 794.4) | 549.8 (367.8 to 792.8) | 546.8 (365.6 to 797.5) | 543.1 (362.3 to 796.9) | 534.5 (355.2 to 788) | 522.9 (342 to 773.2) | 507 (330.9 to 759.1) | 491.7 (318.8 to 749.4) | 476.9 (309.9 to 735.3) | 464.8 (302.8 to 724.9) | 452.8 (293.6 to 708.7) | 438.3 (278.6 to 688.9) |
|  |  | Other infectious diseases | 1330.5 (678.2 to 2615.8) | 1051.6 (568.9 to 2114.8) | 827.4 (500 to 1490.2) | 693.6 (443 to 1185.8) | 591 (401.8 to 1000.3) | 519.1 (371 to 827.7) | 473.2 (339.9 to 726.1) | 428.1 (322.5 to 653.8) | 403.5 (295.6 to 614.8) | 368 (284.7 to 520.3) | 353.7 (276.7 to 496.3) | 331.9 (258.9 to 443.7) | 314.6 (248.3 to 434.3) | 303.1 (240.5 to 396.7) | 287.4 (224.7 to 379.6) | 282.1 (224.9 to 377.6) | 283.2 (223.6 to 381.8) | 287.6 (230.4 to 389.6) | 294.1 (230.7 to 390.3) | 303.2 (238.3 to 393.5) | 303.7 (236.6 to 420.6) | 305.6 (235.6 to 415.1) | 307.1 (239.8 to 407.9) | 309.9 (244.6 to 422.8) | 286.9 (219.7 to 395.5) | 260.2 (196.2 to 380.3) | 227.3 (168.4 to 333) | 207.4 (151.3 to 303.2) | 190.9 (142.4 to 268.6) | 175.2 (128.8 to 251.8) | 146 (105.6 to 224.4) | 121.5 (90.4 to 175.8) |
|  |  | Other non-communicable diseases | 8162.3 (3833.5 to 10171.3) | 7446.8 (3555.7 to 9234.2) | 6956.6 (3519.7 to 8685) | 6527.7 (3387.7 to 8084.7) | 6090.7 (3300.4 to 7474.4) | 5652.9 (3181.2 to 6934.4) | 5278.4 (3059.3 to 6474.3) | 4986.4 (2992.1 to 6124.8) | 4741.1 (2937 to 5854.5) | 4540.1 (2874.2 to 5614.3) | 4380.4 (2890.7 to 5434) | 4287.9 (2878.7 to 5316) | 4234.6 (2912.1 to 5177.2) | 4177.1 (2968.3 to 5006.8) | 4108.5 (2978.3 to 4883.2) | 4030.6 (2965.1 to 4805.4) | 4038 (2996.5 to 4814) | 4134.2 (3203.4 to 4940.2) | 4294 (3357.2 to 5091.4) | 4420.2 (3479 to 5218) | 4460 (3568.3 to 5227.1) | 4436.3 (3607.1 to 5193.9) | 4394.8 (3649.2 to 5080.2) | 4296.9 (3638.2 to 4979.3) | 4034.5 (3561.2 to 4635.1) | 3566.3 (3210.7 to 4103.6) | 2920.7 (2623.2 to 3353.1) | 2546.8 (2273.3 to 2967.3) | 2265.6 (2009.3 to 2686.8) | 2047.6 (1787.2 to 2469.3) | 1519.5 (1314 to 1848.8) | 1203.1 (1003.4 to 1485.8) |
|  |  | Respiratory infections and tuberculosis | 3126.8 (2518.7 to 4350.6) | 2700.9 (2150.7 to 3791.4) | 2405.9 (1934.3 to 3425.1) | 2162.5 (1756.9 to 3007.2) | 1952 (1578.1 to 2680.1) | 1764.4 (1439.2 to 2338.6) | 1606.5 (1335.8 to 2075.3) | 1484.3 (1245.5 to 1863.8) | 1378.4 (1157.3 to 1727.1) | 1287.5 (1083.9 to 1590.3) | 1206.9 (1024.1 to 1475.8) | 1147.9 (974.6 to 1406.8) | 1115.4 (947.1 to 1353.3) | 1091.1 (925.8 to 1321.6) | 1074.7 (901.5 to 1304.5) | 1058 (882 to 1278.1) | 1060.9 (881.9 to 1277.9) | 1074.3 (900.8 to 1298.4) | 1094 (927.8 to 1320.9) | 1102.1 (940.1 to 1318) | 1085.8 (931.2 to 1287.3) | 1057.9 (917.3 to 1239.1) | 1032 (906.8 to 1197.2) | 994.2 (885.2 to 1134.2) | 897.6 (805.3 to 1017.7) | 755.3 (673.8 to 854.1) | 600 (511.2 to 692.8) | 516.5 (427.3 to 605.8) | 456.7 (373.4 to 543.4) | 413.4 (334.5 to 493.3) | 593.2 (499.7 to 701.8) | 546 (433.8 to 744.4) |
|  |  | Self-harm and interpersonal violence | 351.9 (273.3 to 395.7) | 361.5 (276.9 to 406.8) | 347.9 (273.6 to 393.1) | 347.3 (266.6 to 389.8) | 347.7 (271.3 to 391.6) | 343.5 (265 to 389.8) | 353.5 (272.9 to 398.9) | 361.5 (282.3 to 403.1) | 366.3 (288.1 to 409.2) | 381 (298.1 to 422.7) | 374.5 (298.7 to 414.9) | 383.4 (303.9 to 424.2) | 370.3 (288.9 to 408.9) | 368.3 (288.3 to 403) | 367.7 (293.7 to 402.3) | 353.9 (286.7 to 386.1) | 344.4 (284.9 to 376.4) | 350.8 (297.1 to 383.8) | 349.5 (299.3 to 383.2) | 337.9 (291.7 to 369.5) | 325 (285.4 to 355.2) | 312.5 (277.2 to 342.5) | 307.6 (276.8 to 335.4) | 307.9 (277.4 to 338.1) | 292.4 (266.6 to 316.6) | 270.4 (248.3 to 290.4) | 259.2 (239.7 to 276.7) | 244.7 (225.8 to 263.7) | 223.5 (204.8 to 242.4) | 193 (174.4 to 211.6) | 214.2 (194.1 to 233.7) | 208 (189 to 229.6) |
|  |  | Sense organ diseases | 194.5 (134.4 to 274.2) | 196 (135.3 to 277.3) | 197.5 (135.9 to 279.5) | 199.3 (137.7 to 281.7) | 201.4 (139.8 to 284.8) | 203.7 (141.5 to 286.6) | 206.4 (143 to 290.7) | 209.3 (145.3 to 291.9) | 211.9 (145.4 to 296.6) | 213.9 (147.7 to 298.7) | 215 (148.7 to 297.4) | 215.5 (148.9 to 299.4) | 215.2 (149 to 300.2) | 214.6 (148.4 to 299.5) | 213.9 (147.9 to 298.9) | 212.9 (147.1 to 299) | 211.8 (146.7 to 295.9) | 210.3 (145.8 to 296.2) | 208.3 (144 to 290.9) | 205.9 (142.1 to 287.5) | 203.4 (140.8 to 286.1) | 201.1 (139.3 to 281.9) | 199.2 (138.4 to 281) | 197.5 (136.7 to 277.9) | 196.1 (135.6 to 276.8) | 194.6 (134.5 to 276.3) | 192.8 (132 to 271.3) | 190.7 (131.7 to 270) | 189.5 (130.5 to 267.9) | 189.8 (130.8 to 268.6) | 192 (133.3 to 271) | 193.8 (133.3 to 275) |
|  |  | Skin and subcutaneous diseases | 501.9 (327 to 728.6) | 504.7 (328.7 to 730.3) | 507.5 (328.5 to 732.6) | 510.5 (334.2 to 734.7) | 513.7 (335.1 to 739.4) | 516.8 (337.3 to 744) | 520.1 (338.8 to 751.2) | 523.7 (342.3 to 754.3) | 526.8 (344.3 to 758.4) | 529.6 (348.8 to 760.1) | 532 (350.7 to 761.2) | 534.5 (351.9 to 769.6) | 537.6 (353 to 773.3) | 540.8 (355.5 to 781.8) | 543.5 (357.9 to 788.7) | 545.4 (358.6 to 793.1) | 546.6 (359.9 to 798.1) | 547 (359.8 to 795.2) | 546.7 (360.1 to 794.3) | 546 (357.7 to 795.2) | 544.8 (357.8 to 790.6) | 543.9 (357.7 to 791.4) | 542.7 (357.2 to 788.2) | 541.7 (354.8 to 789.1) | 540.6 (353 to 789.6) | 539.5 (351.2 to 789.9) | 539.2 (350.6 to 789.5) | 540.5 (351 to 788.6) | 542.3 (352.4 to 793.1) | 544.8 (356.3 to 796.7) | 546.9 (357.1 to 798.5) | 546.9 (356.2 to 793.8) |
|  |  | Substance use disorders | 38.1 (29.9 to 47.9) | 38.8 (29.8 to 48.6) | 40.6 (31.8 to 50.5) | 41.9 (33 to 52.5) | 44.2 (34.4 to 55) | 47.3 (36.9 to 58.4) | 50.7 (39.4 to 63) | 54.5 (42.8 to 67.3) | 57.7 (45.1 to 71.8) | 60.5 (47.2 to 75.3) | 63 (48.8 to 77.9) | 65.3 (51.4 to 81.1) | 67.5 (53.7 to 83.6) | 69.6 (54.8 to 86.6) | 70.4 (55.3 to 86.6) | 70.8 (56.4 to 87) | 72.9 (58.3 to 89.8) | 73.5 (59.5 to 89.9) | 71.3 (57.8 to 86.7) | 67.1 (53.8 to 81.7) | 62.2 (49.7 to 76.6) | 57.7 (45.9 to 71.6) | 54.8 (44 to 67.7) | 51.7 (42 to 63.1) | 48.3 (39.1 to 58.8) | 44 (35.4 to 53.8) | 42.2 (33.7 to 52) | 41.8 (33.1 to 51.6) | 40.4 (31.8 to 50) | 40.1 (31.6 to 49.5) | 40.8 (32.2 to 50.1) | 41.2 (32.5 to 52.1) |
|  |  | Transport injuries | 2863.5 (2355.8 to 3383.6) | 2676.7 (2244.6 to 3113.6) | 2547.9 (2158.9 to 3008.5) | 2431.5 (2076.4 to 2849.2) | 2326.8 (2007.6 to 2688.3) | 2206.6 (1913 to 2536.3) | 2094.1 (1838.5 to 2420.9) | 2006 (1788.2 to 2272.6) | 1924.1 (1712.7 to 2168.2) | 1865.1 (1676.1 to 2084.6) | 1811 (1620 to 2010.1) | 1769.2 (1592.6 to 1969.4) | 1731.8 (1555.2 to 1924.9) | 1692.5 (1521.8 to 1878.7) | 1655.7 (1491.6 to 1852.2) | 1601.4 (1440 to 1777.9) | 1534.9 (1372.8 to 1705.1) | 1502 (1349.6 to 1664.8) | 1481.7 (1331.3 to 1643.1) | 1453.8 (1321.8 to 1611.8) | 1386.7 (1267.9 to 1522.3) | 1338.1 (1229.4 to 1468.6) | 1302.6 (1203.6 to 1425) | 1264.2 (1158.3 to 1370.8) | 1097.1 (1030.9 to 1202.2) | 884.7 (826.5 to 972.3) | 729.7 (676.3 to 797.1) | 655.2 (595.7 to 727.2) | 611.2 (544.4 to 687.4) | 567.4 (501.3 to 647.4) | 515.8 (454.9 to 578.6) | 457.6 (399.7 to 513.6) |
|  |  | Unintentional injuries | 10300.5 (9478.1 to 11256.7) | 2187.5 (1775.1 to 2663.1) | 1989.5 (1606.4 to 2471.1) | 2033.8 (1661 to 2466.5) | 1810.2 (1467.7 to 2189.8) | 1717.1 (1407.7 to 2073.9) | 1633.1 (1346.3 to 1924.3) | 2058 (1792.2 to 2327.2) | 1546.1 (1286.5 to 1794.1) | 1483.2 (1215.4 to 1712.1) | 1424.1 (1173.7 to 1634.6) | 1491.8 (1238.7 to 1683.2) | 1388.1 (1147.6 to 1559.1) | 5658.1 (5235.3 to 6153.1) | 1356.2 (1159.5 to 1519.2) | 1293.7 (1118 to 1434.1) | 1134.5 (975.2 to 1265) | 1083 (933.2 to 1203.2) | 1058.4 (917.9 to 1175.4) | 1015.9 (889.6 to 1127.1) | 984 (866.4 to 1085.3) | 934.1 (824.2 to 1027.7) | 955.1 (858.5 to 1038.3) | 889.9 (804.4 to 970) | 799.9 (734.7 to 864.6) | 690.3 (641.5 to 747.7) | 581.2 (535 to 641.4) | 636.9 (592.7 to 703.6) | 482.1 (435.4 to 541.9) | 465 (416.3 to 529.7) | 409.9 (370.9 to 462.7) | 352 (316.4 to 401.8) |
| DALYs (Disability-Adjusted Life Years) | Males | Causes | Year |  |  |  |  |  |  |  |  |  |  |  |  |  |  |  |  |  |  |  |  |  |  |  |  |  |  |  |  |  |  |  |
|  |  |  | 1990 | 1991 | 1992 | 1993 | 1994 | 1995 | 1996 | 1997 | 1998 | 1999 | 2000 | 2001 | 2002 | 2003 | 2004 | 2005 | 2006 | 2007 | 2008 | 2009 | 2010 | 2011 | 2012 | 2013 | 2014 | 2015 | 2016 | 2017 | 2018 | 2019 | 2020 | 2021 |
|  |  | Cardiovascular diseases | 916.9 (785.5 to 1089.6) | 860.2 (745.9 to 1015.7) | 823.8 (720.6 to 973.7) | 788.3 (690.9 to 923.8) | 757.7 (664.2 to 874.4) | 727 (642.1 to 831.5) | 697.9 (615.2 to 792.6) | 677 (598.1 to 756.4) | 658.6 (590 to 730.3) | 644.8 (580.3 to 715.4) | 630.1 (571.2 to 695.4) | 617.8 (560.1 to 682.3) | 623.5 (563.9 to 689.3) | 634.8 (572.5 to 704.4) | 649 (584.4 to 726.8) | 649.6 (582.3 to 732.6) | 655.2 (594.4 to 739.4) | 666.8 (605.9 to 751.4) | 683.5 (626.2 to 763.1) | 691 (633.8 to 771.6) | 681.1 (624.8 to 756.9) | 672 (614.7 to 743.4) | 671.8 (615.4 to 737.5) | 666.3 (614.4 to 731.4) | 609.9 (564.4 to 663.2) | 520.9 (479.7 to 573.7) | 449.4 (400.1 to 501) | 412.4 (360.8 to 463.5) | 378.8 (326.2 to 429.3) | 356.6 (304.5 to 409.3) | 325.6 (282.2 to 371.3) | 300.8 (258.5 to 348.8) |
|  |  | Chronic respiratory diseases | 427.7 (322.5 to 575.4) | 406 (307.2 to 552.4) | 389.8 (294.8 to 532.7) | 375.5 (282.1 to 517.2) | 363.4 (274.3 to 501.5) | 352 (264.6 to 486.5) | 341.2 (258 to 469.9) | 332.3 (248.3 to 455.9) | 324.6 (242.5 to 443.1) | 318.3 (235.6 to 437.9) | 313 (231 to 428.9) | 308.3 (226.2 to 425) | 305 (223 to 423.2) | 304.3 (223.4 to 419.7) | 305.1 (225.1 to 420.7) | 303.9 (224.4 to 418.5) | 303.1 (224.7 to 417.2) | 302.1 (222.1 to 421) | 302 (224.5 to 419.1) | 300.5 (223.4 to 415.8) | 296.6 (219.5 to 408.8) | 293.6 (216.3 to 404.6) | 292.4 (216.6 to 400.1) | 289.6 (213.9 to 396.9) | 276.1 (201.8 to 381.3) | 257.2 (184.9 to 363.5) | 241.4 (172.4 to 348.1) | 230.7 (162.5 to 333.3) | 221.6 (155 to 322.9) | 216.4 (149.2 to 318.9) | 216.4 (145.9 to 320.2) | 211.4 (139.1 to 314.8) |
|  |  | Diabetes and kidney diseases | 126.2 (99.9 to 151.8) | 120.6 (96.3 to 143.6) | 117.4 (94.4 to 142) | 114.8 (92.9 to 138.6) | 111.9 (91.5 to 135) | 109.4 (89.7 to 132.5) | 107.5 (88.2 to 130.9) | 105.5 (87.3 to 127.9) | 103.7 (87 to 125.3) | 101.7 (86.2 to 122) | 101.3 (86.1 to 120.2) | 101.2 (85.4 to 117.5) | 102.9 (84.7 to 117.9) | 105 (86.8 to 120.1) | 109 (88.2 to 124) | 113.2 (89 to 128.8) | 116.8 (88.9 to 131.4) | 120.3 (90.7 to 135.9) | 124.9 (92.3 to 142.1) | 128.6 (93.2 to 147.3) | 130.5 (92.8 to 149.8) | 132.7 (91.7 to 150.3) | 133.4 (90.6 to 150.4) | 133.5 (90.7 to 148.7) | 124.5 (85.4 to 138.6) | 112.4 (80.1 to 131.4) | 98.8 (72.6 to 115.3) | 91.6 (68.2 to 108.9) | 85.8 (65.7 to 102) | 82 (63.6 to 98.1) | 73.9 (59.5 to 86.7) | 67.8 (54.2 to 79.7) |
|  |  | Digestive diseases | 501.1 (328.6 to 598.9) | 462.9 (307.9 to 552.5) | 438.8 (297.5 to 526.5) | 413.7 (281.8 to 489.5) | 396.2 (276.8 to 471.9) | 374.2 (267.9 to 447.1) | 349.9 (254.9 to 411.8) | 334.5 (247.7 to 395.1) | 320.9 (239.2 to 380.8) | 311.5 (232.3 to 366.6) | 296.8 (227.4 to 343) | 282.3 (218.4 to 324.4) | 278.5 (214.3 to 318.6) | 277 (212.6 to 315.3) | 286 (208.1 to 327.9) | 284 (202.8 to 323.5) | 284.4 (202.4 to 323.8) | 287.2 (202.5 to 326.3) | 293.2 (202.8 to 334) | 294.8 (202.3 to 337.4) | 289.9 (196.6 to 332.1) | 284.6 (193.1 to 322.8) | 281.6 (191 to 319.2) | 276.2 (188.8 to 311.1) | 256.9 (176.9 to 289.7) | 223.5 (153.6 to 255.7) | 193.2 (138.5 to 234.9) | 177 (128.6 to 221.4) | 163.9 (120.1 to 210.6) | 153.5 (113.7 to 201) | 134.5 (104 to 173.6) | 120.1 (96 to 156.5) |
|  |  | Enteric infections | 1455.6 (897.9 to 2401.7) | 1262.5 (803.3 to 2010.4) | 1113.7 (729.9 to 1721.8) | 998.1 (663 to 1513.6) | 902.4 (599.8 to 1338.2) | 830 (562.7 to 1195.1) | 730.2 (494.5 to 1043.5) | 670.5 (461.1 to 950.5) | 615.2 (421.1 to 860.8) | 572.6 (400 to 796.1) | 541.4 (388.1 to 732.8) | 481.4 (343.2 to 653.1) | 451.7 (325.4 to 608.1) | 425.6 (312.4 to 567.3) | 403.4 (296.5 to 535.1) | 385.4 (283.4 to 509.8) | 369.8 (273.2 to 484.7) | 360.3 (270.5 to 468.8) | 354.1 (270.1 to 455.2) | 345.1 (262.1 to 439.1) | 334 (258.6 to 423.2) | 327.2 (254 to 413.7) | 314.7 (241.8 to 397.7) | 306.7 (235.8 to 389.4) | 285.2 (217.9 to 363.1) | 263 (201 to 335) | 225.5 (171.9 to 288.9) | 204.1 (154.6 to 265.6) | 188.2 (142 to 249.2) | 176.2 (130.4 to 236.5) | 150.9 (111.7 to 202) | 132.2 (96 to 180.3) |
|  |  | HIV/AIDS and sexually transmitted infections | 71.1 (26.1 to 145.7) | 66.2 (24.6 to 134.9) | 61.9 (23.5 to 125.1) | 59.5 (23 to 121.2) | 57.7 (22.5 to 120) | 55.7 (21.9 to 115.7) | 54 (21.2 to 112.5) | 53 (21.1 to 110.6) | 52.4 (21.1 to 109.7) | 52 (21 to 110.3) | 52 (21 to 107.3) | 51.9 (21.1 to 108.3) | 53.4 (22.9 to 110.1) | 53.7 (22.9 to 110.4) | 54.7 (24.3 to 111.7) | 55.6 (24.7 to 112.9) | 58.3 (26.4 to 117.3) | 62.6 (27.9 to 125.7) | 67.6 (30.2 to 136.3) | 72.9 (32.9 to 148.1) | 76.8 (34.7 to 155.7) | 79.4 (36.5 to 164.3) | 82.1 (38.8 to 165.8) | 85.1 (42.2 to 169.4) | 83.8 (41.3 to 161.5) | 80.1 (39.6 to 153.2) | 74 (35.3 to 147.6) | 71.2 (34.7 to 142.5) | 67 (32.8 to 132.9) | 62.6 (30.9 to 124.6) | 57.6 (28.6 to 109.9) | 53.3 (26.5 to 101.5) |
|  |  | Maternal and neonatal disorders | 13770.3 (11612 to 16578.6) | 12472.4 (10503.2 to 15016.5) | 11537.2 (9778.3 to 13761.4) | 10719.3 (9017 to 12787.3) | 9885.5 (8321.7 to 11767) | 9122.1 (7672.5 to 10825.5) | 8509.6 (7186.7 to 10007.1) | 8033.5 (6770.4 to 9399) | 7626.3 (6368.4 to 8890.5) | 7263.2 (6150.1 to 8492.9) | 6963.3 (5895.6 to 8086.5) | 6813.2 (5856.1 to 7913.2) | 6690.7 (5733.5 to 7688.4) | 6534.1 (5621 to 7479.9) | 6294.2 (5383 to 7189.3) | 6157.1 (5274.1 to 7099.4) | 6213.3 (5301.3 to 7149) | 6440.7 (5496.2 to 7443.5) | 6728.5 (5693.8 to 7826.2) | 6907.8 (5850.2 to 8066.9) | 6942.7 (6001 to 7980.5) | 6860 (5981.5 to 7863.2) | 6799.8 (6037.3 to 7630.9) | 6676.4 (5963.3 to 7405.9) | 6355 (5731.7 to 6953) | 5645.6 (5152 to 6108.9) | 4370.6 (3991.2 to 4743.7) | 3571.1 (3173.5 to 3929.2) | 3000.3 (2624.2 to 3313.6) | 2574.6 (2230.5 to 2915) | 1715.1 (1505.6 to 1916.8) | 1177.2 (987.2 to 1387.4) |
|  |  | Mental disorders | 1324.8 (967.2 to 1726.8) | 1349 (983.9 to 1761.1) | 1372.3 (1004.8 to 1786.6) | 1395.9 (1016.1 to 1830.7) | 1420.4 (1033.6 to 1854.3) | 1448.6 (1053.5 to 1898.5) | 1480.8 (1075.4 to 1942.7) | 1515.9 (1103.4 to 1998.5) | 1548 (1129.1 to 2036.5) | 1572.7 (1143.6 to 2063) | 1588.9 (1152.5 to 2082.3) | 1600.5 (1163.1 to 2105.2) | 1609.4 (1172.4 to 2115.5) | 1615.5 (1169.1 to 2124.9) | 1617.4 (1171.9 to 2122.8) | 1612.8 (1170.4 to 2118.4) | 1598.4 (1163.5 to 2099.6) | 1573.7 (1144.7 to 2064) | 1541.1 (1120.8 to 2015.8) | 1507.3 (1097.9 to 1974.9) | 1474.8 (1071.2 to 1928.4) | 1445.3 (1054.7 to 1892.2) | 1419 (1040.2 to 1857.4) | 1396.6 (1023.1 to 1829.1) | 1376.5 (1008.7 to 1791.9) | 1360.9 (996.4 to 1769.7) | 1349.6 (988.1 to 1759.9) | 1345.3 (982.9 to 1757.8) | 1349.8 (986.7 to 1765.5) | 1361.4 (993.7 to 1781.8) | 1587.8 (1138.6 to 2101.8) | 1597.5 (1149.9 to 2132.2) |
|  |  | Musculoskeletal disorders | 209.9 (138.6 to 292.2) | 216.1 (142.8 to 301.8) | 221.9 (147.3 to 310) | 227.2 (150.1 to 315.8) | 233.8 (153.7 to 325.1) | 241.7 (159 to 339.8) | 250.4 (163.7 to 350.4) | 258 (169.1 to 360.3) | 264 (172.1 to 371.3) | 268.7 (175.2 to 379.9) | 272.2 (178.2 to 386.5) | 276 (181.2 to 386.4) | 279.4 (185.4 to 392.7) | 282.5 (186.7 to 397.7) | 284.5 (188 to 397.3) | 284.5 (188.8 to 398.1) | 282.7 (187.6 to 396.6) | 279.2 (186 to 389.6) | 274.5 (181 to 384) | 268.7 (177.6 to 372.1) | 262 (173.8 to 363.8) | 255.6 (171.3 to 354) | 249.9 (166.4 to 347.3) | 244.3 (162.9 to 338.9) | 238.8 (159.1 to 330.4) | 233.3 (155.8 to 325) | 228.9 (150.9 to 316.2) | 226.5 (149.7 to 314.4) | 226.3 (151.6 to 313.5) | 228.5 (152.4 to 317.5) | 232.4 (153.5 to 324.2) | 233 (154.6 to 326.8) |
|  |  | Neglected tropical diseases and malaria | 211 (86.2 to 633.4) | 239.4 (85.4 to 1018.1) | 175.6 (78.8 to 551.9) | 170.1 (74.7 to 628.5) | 143.8 (74.2 to 358.5) | 130 (67 to 322.9) | 123.7 (66.4 to 302) | 105.4 (59.8 to 234.6) | 97.6 (59.7 to 209.4) | 102.1 (56.5 to 229) | 87.7 (54 to 184.9) | 84.3 (53 to 167.7) | 83.1 (52.3 to 153.9) | 84.6 (51.7 to 155.2) | 77.3 (50 to 130.7) | 77.9 (49.3 to 125.2) | 75.7 (48.7 to 115.9) | 75.7 (49.6 to 112.2) | 75.6 (49.9 to 109.4) | 75.4 (50.2 to 107.4) | 75.5 (50.4 to 107) | 75.3 (51.2 to 104.7) | 74.9 (50.4 to 104.3) | 74.4 (51.1 to 102.3) | 72.6 (49.7 to 100) | 69.7 (47 to 96.8) | 65.7 (43.9 to 91.7) | 62 (40.6 to 87.4) | 58.6 (38.8 to 83.9) | 56.1 (36.6 to 80.8) | 52.6 (34.2 to 77.2) | 49.5 (31.8 to 71.9) |
|  |  | Neoplasms | 744.6 (468.4 to 988.2) | 723.8 (476.9 to 947.5) | 710.9 (471.8 to 915.6) | 699.5 (468.6 to 887.4) | 688.6 (466.8 to 859) | 675 (467.5 to 830.4) | 661.2 (469.5 to 801.4) | 648.5 (462 to 781.9) | 636.8 (466.7 to 761.8) | 626.6 (460.7 to 747.2) | 620 (459.9 to 729.4) | 614.7 (461.1 to 717.5) | 608.3 (458.8 to 707) | 605.6 (457.1 to 704.3) | 608 (460.2 to 703.1) | 611.4 (457.9 to 708.3) | 617.9 (459.9 to 715.5) | 628.1 (462.1 to 726.1) | 639.9 (461.9 to 740.9) | 646.9 (464.5 to 746.6) | 641.8 (458.5 to 747.8) | 644.3 (457.8 to 743.7) | 657 (460.9 to 762.7) | 668.7 (472.3 to 774.4) | 633.6 (450.7 to 731.4) | 557.2 (400.7 to 659.3) | 506.4 (360.6 to 616.4) | 482.1 (337.5 to 601.7) | 459.5 (316.5 to 584.7) | 442.3 (298.7 to 576.4) | 425.8 (291.8 to 545.2) | 397.2 (273.7 to 510.2) |
|  |  | Neurological disorders | 660.6 (359.9 to 1105.2) | 660.1 (354.2 to 1120.6) | 663.5 (356.1 to 1121.7) | 667.4 (355.2 to 1137.1) | 671.5 (354.7 to 1156) | 677.1 (353.1 to 1171.9) | 681.6 (348.8 to 1188.9) | 685 (342.7 to 1194.8) | 685.7 (335.5 to 1188) | 683.9 (330.7 to 1201.6) | 681.9 (330.3 to 1210.9) | 680 (333.6 to 1198.9) | 676.9 (339.8 to 1180.1) | 673.8 (337.8 to 1178.6) | 672.4 (333 to 1179.6) | 670 (331.2 to 1175.3) | 665.9 (325.5 to 1165.3) | 659.9 (321.3 to 1138.6) | 651.8 (318 to 1117.3) | 643.4 (319.9 to 1100.6) | 631.7 (318.9 to 1090.4) | 620 (314.7 to 1063.7) | 608.1 (308.9 to 1034.4) | 597.5 (306 to 1018.5) | 579 (301.1 to 993.5) | 572.1 (298 to 969.8) | 550.8 (276.9 to 945) | 539.6 (264.7 to 935.9) | 531.5 (259.7 to 925.9) | 528.4 (247.7 to 931.1) | 521.2 (237.6 to 924.7) | 533.6 (235.1 to 973.5) |
|  |  | Nutritional deficiencies | 915.6 (665.8 to 1261.3) | 832.6 (590.5 to 1154.1) | 769 (543.3 to 1073.4) | 713.5 (500.1 to 999.6) | 663.8 (462 to 932.7) | 617.9 (426.4 to 878.2) | 575 (396.6 to 824.3) | 540.3 (374.8 to 778.7) | 509.9 (348.5 to 735.4) | 485.3 (328.5 to 699.6) | 464.6 (310 to 671.4) | 447.8 (296.2 to 646.2) | 430.9 (286.8 to 627.5) | 417.1 (278.3 to 601.8) | 404.4 (266.2 to 587.7) | 395.8 (257.6 to 579.9) | 389.8 (256.7 to 567.9) | 387.2 (255.7 to 562.2) | 388.4 (253.1 to 561) | 387.2 (251.3 to 567.8) | 384.9 (245.4 to 564.6) | 379.7 (241.1 to 560.3) | 376.3 (238.2 to 560.3) | 372.8 (233.4 to 554.9) | 363.3 (225.6 to 536.4) | 348.6 (211.7 to 520.7) | 328.9 (197.8 to 498.1) | 308.6 (183.3 to 477.5) | 288.9 (170.2 to 451.4) | 273.2 (161.6 to 436.1) | 258.4 (150.4 to 425.2) | 242.9 (141.5 to 405.6) |
|  |  | Other infectious diseases | 1233.5 (674.2 to 2353.1) | 984.2 (584.2 to 1777.4) | 788.1 (499.4 to 1370.8) | 670.1 (443.9 to 1076.2) | 581.5 (415.5 to 869.6) | 519 (377.4 to 782) | 475.1 (353.6 to 692.3) | 434.7 (332.3 to 618.7) | 410 (310.9 to 576) | 377.5 (292.9 to 509.7) | 361.3 (289 to 482.8) | 338.1 (269.4 to 442.5) | 322.6 (260.3 to 419.9) | 312.8 (254.3 to 403.6) | 300.6 (246.7 to 377.2) | 299.2 (248.4 to 381.2) | 301.9 (246.9 to 382) | 309.8 (251.9 to 396.2) | 318.1 (254.4 to 409.7) | 326 (257.5 to 414.8) | 324.3 (260.1 to 421.4) | 322.4 (259.4 to 419.2) | 322.7 (260.5 to 415.7) | 324.5 (257.7 to 424.6) | 290 (218.1 to 397.2) | 257.1 (187.6 to 359.4) | 218.9 (152.9 to 308) | 196.6 (142.7 to 280.5) | 178 (129.6 to 247.3) | 161.6 (115.7 to 226.4) | 130.8 (94.6 to 209.2) | 107.9 (80.6 to 161.6) |
|  |  | Other non-communicable diseases | 9510.8 (5593.5 to 11811) | 8663 (5257.4 to 10748.9) | 8083.3 (5032.8 to 9985.8) | 7588.4 (4879.6 to 9431.1) | 7080.2 (4618 to 8892.9) | 6563.1 (4422.6 to 8196.5) | 6110.5 (4283.9 to 7577.9) | 5742 (4146.8 to 7049.5) | 5429 (4018.5 to 6651.7) | 5167.9 (3928.5 to 6277.3) | 4963.7 (3853.6 to 6001.5) | 4845.8 (3830.2 to 5871.2) | 4779.1 (3820.9 to 5807.3) | 4715.1 (3804.4 to 5712.3) | 4640.8 (3793.3 to 5479.2) | 4557.4 (3806.4 to 5335.9) | 4574.8 (3863.6 to 5340.3) | 4692.8 (3960.5 to 5455.3) | 4886.3 (4120.3 to 5633.1) | 5038.2 (4263.8 to 5807.6) | 5089.4 (4376.4 to 5812.3) | 5068.5 (4373.2 to 5736.5) | 5028 (4381.1 to 5718.6) | 4926.4 (4387.7 to 5555.9) | 4636 (4143.9 to 5253.9) | 4105.9 (3716.4 to 4606.7) | 3344.6 (2999.3 to 3830.3) | 2902 (2571 to 3408.1) | 2567.1 (2226 to 3069.2) | 2303.8 (1966.8 to 2790.7) | 1602.8 (1385.8 to 1941.1) | 1221.3 (1027.5 to 1524.1) |
|  |  | Respiratory infections and tuberculosis | 3215.6 (2683.4 to 4184.3) | 2788.2 (2289.1 to 3621.7) | 2489.2 (2025.9 to 3228.2) | 2250.2 (1828.8 to 2908.2) | 2047.4 (1668.6 to 2614.3) | 1867.7 (1539.3 to 2318.1) | 1716 (1429 to 2076.4) | 1596.8 (1332.6 to 1935.1) | 1492.5 (1256.8 to 1779.4) | 1400 (1180.5 to 1656.6) | 1316.4 (1125.1 to 1551.5) | 1256.5 (1074.3 to 1479.4) | 1227.9 (1053.4 to 1435.4) | 1211.9 (1040.5 to 1411.8) | 1209.8 (1037.3 to 1408.3) | 1204.5 (1029.5 to 1398.4) | 1215.3 (1043 to 1415.9) | 1239.1 (1061.7 to 1449) | 1267.3 (1097.1 to 1473.7) | 1276.7 (1119.4 to 1494.4) | 1255.7 (1102.5 to 1468.6) | 1220.1 (1072.5 to 1404) | 1190.5 (1054 to 1347.7) | 1142.8 (1023.8 to 1280.1) | 1039.1 (937.2 to 1163.3) | 877.2 (781.4 to 986.4) | 696.4 (589.2 to 803.3) | 597.3 (486.9 to 698.3) | 525.7 (422.6 to 623.9) | 473 (375.2 to 566.8) | 628.5 (519.3 to 757.1) | 625 (507.2 to 809.8) |
|  |  | Self-harm and interpersonal violence | 708.8 (638.7 to 768.9) | 644.6 (559 to 697.4) | 578.6 (511 to 632.2) | 568.1 (496.7 to 619.8) | 565.6 (504.3 to 613.9) | 531.9 (467.4 to 580.9) | 543.8 (483.6 to 591.8) | 544.2 (480 to 590.5) | 554 (493.6 to 600.6) | 576.2 (514.5 to 623) | 571.3 (511.5 to 617.6) | 602.8 (541.1 to 652.1) | 582.3 (515 to 626.9) | 610.5 (542 to 653.9) | 633.1 (570.6 to 680.7) | 605.6 (545.3 to 654.2) | 609.3 (550.8 to 657.2) | 603.5 (545.8 to 652.2) | 621 (563.3 to 670.5) | 612.7 (559.8 to 661) | 579.6 (526.4 to 624.5) | 531.4 (484.2 to 574.1) | 510.7 (465.3 to 550) | 509.1 (466.6 to 545.5) | 470.3 (436.3 to 503.5) | 447.7 (415.9 to 477.3) | 448 (415.9 to 477.6) | 423.4 (391.3 to 455.5) | 383.2 (350.1 to 416.1) | 336.4 (304.4 to 371.5) | 385.4 (354.1 to 416.7) | 355.6 (320.8 to 388.6) |
|  |  | Sense organ diseases | 191 (130.5 to 267.3) | 193.3 (131.7 to 271.3) | 195.2 (133.8 to 275.3) | 197.1 (134.4 to 277.9) | 199.3 (136 to 279.9) | 201.7 (137.5 to 284.6) | 204.4 (139.3 to 288.7) | 207.1 (140.9 to 292.9) | 209.5 (143 to 295.9) | 211.3 (144.4 to 296.4) | 212.6 (145.4 to 297.5) | 213.3 (144.6 to 299.2) | 213.5 (146 to 301.6) | 213.5 (145.4 to 303.3) | 213.1 (145.7 to 301.2) | 212.2 (144.3 to 301.7) | 210.7 (142.9 to 300.5) | 208.6 (141.8 to 295.6) | 205.8 (140.9 to 291.3) | 202.7 (138.6 to 286.9) | 199.6 (135.2 to 278.9) | 197 (134.3 to 277) | 194.8 (132.9 to 272.1) | 192.8 (130.9 to 273) | 191.2 (130 to 269.5) | 190 (129.4 to 267.9) | 188.7 (128.9 to 266.1) | 187.9 (128.5 to 265.6) | 187.8 (127.7 to 265.9) | 188.8 (128.8 to 268.3) | 191.1 (128.9 to 269.7) | 191.7 (130 to 272) |
|  |  | Skin and subcutaneous diseases | 442.4 (288 to 634.9) | 445.4 (289.1 to 641) | 448.5 (292.4 to 646.6) | 451.8 (294.3 to 650.7) | 455.3 (296.9 to 658) | 459.1 (300.7 to 665.5) | 463.1 (303 to 670.1) | 467.7 (307.8 to 678.8) | 471.5 (310.7 to 684.5) | 475 (312.6 to 685.1) | 478.1 (314.5 to 692.8) | 480.9 (316.2 to 698.3) | 484.3 (318.2 to 702.5) | 487.4 (321.3 to 708.4) | 490.1 (324.1 to 712.8) | 491.9 (325.1 to 718.7) | 493 (326.1 to 723) | 493.4 (324.3 to 721.9) | 492.8 (324.2 to 719.2) | 491.6 (322.8 to 714) | 489.8 (321.8 to 714.1) | 487.7 (321.3 to 708.8) | 485.7 (319.2 to 707) | 483.7 (316.6 to 707.2) | 481.6 (316.1 to 699.4) | 480 (314.7 to 700.6) | 478.7 (312.8 to 700.8) | 479.2 (313.6 to 700.8) | 480.9 (313.2 to 704.2) | 483 (314.8 to 708.9) | 484.5 (318.1 to 710.8) | 486.3 (317.9 to 709.1) |
|  |  | Substance use disorders | 65.8 (51.4 to 83.1) | 68.1 (53.8 to 85.3) | 71.4 (57.7 to 87) | 73.7 (58.6 to 90.6) | 77.8 (62.4 to 96.6) | 83.7 (67.2 to 101.7) | 91.5 (72.4 to 111.8) | 100.7 (79.8 to 123.3) | 109.5 (87.7 to 132.9) | 117.2 (93 to 141.5) | 123.8 (97.7 to 151.2) | 129.6 (102.7 to 159.2) | 134.8 (108.8 to 164.9) | 139.3 (111.9 to 169.5) | 143 (115.6 to 177.5) | 144.8 (116.5 to 175.3) | 145.6 (118.3 to 181.1) | 143.3 (116.4 to 174.9) | 139.3 (112.6 to 168.8) | 132.5 (107.3 to 162) | 125.7 (101 to 155.3) | 118.8 (95.1 to 144.8) | 111.8 (89.3 to 134.9) | 103.8 (83.8 to 124.9) | 98.6 (78.8 to 117.8) | 93.2 (74.3 to 112.8) | 90.4 (71.9 to 108.3) | 89.7 (70.4 to 108) | 89.6 (70.4 to 108.2) | 90.2 (71.3 to 110.4) | 89.3 (70.1 to 109.1) | 89.7 (70.4 to 109) |
|  |  | Transport injuries | 5048.6 (4410.1 to 5998) | 4822.5 (4202.7 to 5629.3) | 4672.3 (4124.6 to 5421.2) | 4526.8 (4011.3 to 5211.1) | 4403.6 (3890.5 to 5030.8) | 4263.6 (3800.2 to 4847.9) | 4148.1 (3714.8 to 4681.2) | 4070.3 (3668.5 to 4504.2) | 3988.9 (3632.4 to 4371.3) | 3931.2 (3609.4 to 4281.4) | 3867.3 (3548.3 to 4178.6) | 3823.2 (3511.3 to 4141.6) | 3806.2 (3517.3 to 4131.1) | 3781.6 (3509.7 to 4086.5) | 3730.2 (3485 to 4033.7) | 3616.9 (3378.2 to 3932.7) | 3512.1 (3279.2 to 3806.2) | 3404.1 (3184.3 to 3701.6) | 3312.2 (3088.3 to 3594.4) | 3196.3 (2990.5 to 3476.9) | 2992.7 (2804.5 to 3263.7) | 2864.4 (2686.7 to 3103.5) | 2772.7 (2601.5 to 2995.5) | 2677.9 (2527.2 to 2855) | 2337 (2224.9 to 2483.1) | 1894.7 (1778.9 to 2029) | 1649.2 (1528.7 to 1764) | 1527.7 (1406.3 to 1649.1) | 1410.4 (1278.8 to 1538.2) | 1323.3 (1196.5 to 1449.7) | 1260.7 (1153.6 to 1370.6) | 1174.9 (1060.3 to 1295.7) |
|  |  | Unintentional injuries | 9010.2 (8277.3 to 9764.8) | 3305 (2794 to 3827.9) | 3089.7 (2640.2 to 3572.9) | 3055.4 (2602.6 to 3497.5) | 2856.8 (2434.2 to 3257.6) | 2742.5 (2331.5 to 3130.9) | 2635.4 (2254 to 2998.6) | 2861.2 (2472.3 to 3203.4) | 2506.7 (2152.8 to 2818.9) | 2438.4 (2081.7 to 2732.1) | 2367.2 (2017.7 to 2622.4) | 2379.4 (2032.6 to 2638.6) | 2278.8 (1942.7 to 2510.8) | 4866.1 (4489.6 to 5248.3) | 2177.8 (1887 to 2392.4) | 2097.9 (1837.9 to 2318.1) | 1951.9 (1712.2 to 2161.2) | 1881.6 (1657.7 to 2071.2) | 1827.8 (1617.9 to 2014.5) | 1746.3 (1552.8 to 1923.8) | 1662.6 (1496.1 to 1834.7) | 1561.4 (1410.4 to 1727.8) | 1542.9 (1405.4 to 1703.4) | 1464.4 (1337.4 to 1631.4) | 1330.7 (1226.8 to 1480.9) | 1158 (1073.4 to 1281.2) | 996.2 (915.2 to 1108.5) | 991.8 (914.7 to 1103.1) | 840.6 (759 to 948) | 799.1 (717.6 to 905.5) | 736.7 (671.4 to 831.9) | 653.9 (586.7 to 752.4) |
| YLDs (Years Lived with Disability) | Both Sexes | Causes | Year |  |  |  |  |  |  |  |  |  |  |  |  |  |  |  |  |  |  |  |  |  |  |  |  |  |  |  |  |  |  |  |
|  |  |  | 1990 | 1991 | 1992 | 1993 | 1994 | 1995 | 1996 | 1997 | 1998 | 1999 | 2000 | 2001 | 2002 | 2003 | 2004 | 2005 | 2006 | 2007 | 2008 | 2009 | 2010 | 2011 | 2012 | 2013 | 2014 | 2015 | 2016 | 2017 | 2018 | 2019 | 2020 | 2021 |
|  |  | Cardiovascular diseases | 78.5 (53.2 to 110.1) | 79.5 (53.6 to 111.5) | 80.5 (54.8 to 113.2) | 81.4 (55.8 to 115.1) | 82.4 (56.2 to 116.8) | 83.3 (57.2 to 118.1) | 84.2 (57.6 to 119.4) | 85 (58.2 to 120.4) | 85.8 (58.4 to 121.6) | 86.3 (58.6 to 122) | 86.7 (58.7 to 122.7) | 87.4 (59.1 to 123.3) | 88.4 (59.9 to 125.6) | 89.7 (60.8 to 127.8) | 90.8 (61.4 to 128.8) | 91.5 (62.2 to 130.6) | 91.8 (62 to 131.1) | 91.9 (61.9 to 130.8) | 91.5 (61.5 to 131.3) | 90.8 (61.3 to 129.5) | 89.7 (60.3 to 127.9) | 88.2 (59 to 126.4) | 86.7 (58 to 124.1) | 85.3 (57.4 to 121.5) | 84.1 (56.2 to 119.6) | 83.1 (55.7 to 118) | 82.2 (55.3 to 117.1) | 81.5 (54.3 to 115.7) | 81.1 (54.1 to 115.7) | 81.4 (54.3 to 115.6) | 82 (54.3 to 116.1) | 82.5 (54.3 to 118) |
|  |  | Chronic respiratory diseases | 222.8 (136.9 to 345.4) | 217.5 (133.8 to 337.1) | 212.5 (129.2 to 330.1) | 208.1 (127.1 to 324.2) | 204.5 (124 to 316.6) | 201.7 (121.7 to 311.1) | 199.2 (119.8 to 306.6) | 196.8 (118.4 to 304.5) | 194.7 (116.7 to 299.5) | 193 (115.5 to 298.4) | 191.7 (115.3 to 296.5) | 190.4 (114.5 to 295.1) | 189 (113.2 to 290.8) | 187.6 (113.5 to 289.5) | 186.3 (112.7 to 287.5) | 185.3 (112.5 to 287.2) | 184.3 (111.5 to 284.1) | 182.9 (109.9 to 283.8) | 180.9 (109.1 to 280.8) | 179 (107.2 to 277.5) | 177.3 (105.9 to 275.2) | 175.4 (104.7 to 272.3) | 173 (103.3 to 269.1) | 170.1 (102.3 to 267.4) | 167.3 (99.6 to 262.1) | 164.8 (99 to 259.7) | 162.1 (96.8 to 255.7) | 158.8 (95.5 to 250.5) | 156.2 (93.2 to 246.4) | 155.2 (92.9 to 244.9) | 159.2 (94.2 to 251.7) | 158.5 (94.2 to 251.6) |
|  |  | Diabetes and kidney diseases | 16.2 (10.9 to 23.4) | 16.5 (11.1 to 23.8) | 16.8 (11.3 to 24.2) | 17.1 (11.5 to 24.7) | 17.4 (11.6 to 25) | 17.8 (11.9 to 25.4) | 18.2 (12 to 26.2) | 18.5 (12.4 to 26.6) | 18.9 (12.5 to 27.1) | 19.1 (12.7 to 27.7) | 19.2 (12.8 to 27.8) | 19.3 (12.9 to 27.9) | 19.3 (13 to 27.7) | 19.3 (13 to 27.7) | 19.3 (13 to 27.6) | 19.3 (13 to 27.5) | 19.4 (13.1 to 27.7) | 19.6 (13.4 to 28.1) | 19.8 (13.5 to 28.3) | 19.8 (13.5 to 28.2) | 19.6 (13.4 to 28) | 19.3 (13.1 to 27.3) | 18.8 (12.8 to 26.5) | 18.3 (12.4 to 26) | 17.8 (12.3 to 25.2) | 17.5 (11.9 to 24.8) | 17.2 (11.5 to 24.5) | 16.9 (11.4 to 24) | 16.7 (11.3 to 23.9) | 16.7 (11.2 to 23.9) | 17 (11.5 to 24.5) | 17.5 (11.9 to 25.1) |
|  |  | Digestive diseases | 56.3 (40 to 77.4) | 56.3 (39.8 to 77.8) | 56.1 (39.5 to 78.6) | 55.9 (39.7 to 77.1) | 55.8 (39.5 to 76.9) | 56 (40 to 77) | 56.2 (40.5 to 76.9) | 56.5 (39.9 to 77.7) | 56.7 (40.8 to 77.3) | 56.8 (40.4 to 78.2) | 56.9 (40.3 to 78.4) | 56.9 (40.6 to 78.7) | 56.9 (40.2 to 78.5) | 57 (40.4 to 78.3) | 56.9 (40.4 to 78.2) | 56.8 (40 to 78.2) | 56.8 (40.1 to 78.3) | 56.7 (39.7 to 78.3) | 56.6 (39.8 to 78) | 56.3 (39.5 to 78.1) | 55.9 (39.2 to 77.7) | 55.2 (38.8 to 76.1) | 54.2 (38.2 to 75) | 53.3 (37.2 to 73.8) | 52.4 (36.6 to 72.3) | 51.8 (36.2 to 71.2) | 51.2 (35.7 to 70.6) | 50.8 (35.3 to 70.4) | 50.5 (34.9 to 68.7) | 50.4 (34.8 to 68.8) | 50.3 (35 to 69.1) | 50 (34.9 to 68.7) |
|  |  | Enteric infections | 215.7 (139.1 to 313) | 200.4 (128.7 to 293.3) | 186.4 (119.7 to 273.1) | 173.6 (110.8 to 256.5) | 162 (102.7 to 239.9) | 151.4 (95.2 to 225) | 141.7 (88.5 to 211.9) | 132.7 (82.7 to 197.1) | 124.8 (77.5 to 186.6) | 118.1 (73.1 to 176.3) | 112.7 (69.1 to 168.7) | 108.1 (66.7 to 162.9) | 104 (63.7 to 156.4) | 100.4 (61.5 to 152.1) | 97.2 (60.1 to 148.5) | 94.6 (58.7 to 144.2) | 92.5 (56.7 to 140) | 90.9 (55.8 to 137.1) | 89.4 (54.4 to 135.5) | 87.9 (53.2 to 133.1) | 86.1 (52 to 130.1) | 83.9 (50.9 to 126.2) | 81.6 (50 to 122.6) | 79.2 (48.7 to 118.8) | 76.9 (47.3 to 114.8) | 75 (45.9 to 111.8) | 73.2 (44.4 to 110) | 71.6 (43.4 to 107.1) | 70.7 (42.5 to 106.2) | 70.8 (42.6 to 106.6) | 71.5 (42.9 to 108.1) | 72.2 (43.8 to 109.9) |
|  |  | HIV/AIDS and sexually transmitted infections | 1.3 (0.8 to 2.1) | 1.3 (0.7 to 2.2) | 1.3 (0.8 to 2.2) | 1.4 (0.8 to 2.2) | 1.4 (0.8 to 2.2) | 1.5 (0.8 to 2.4) | 1.6 (0.9 to 2.5) | 1.7 (1 to 2.7) | 1.8 (1 to 2.9) | 1.9 (1.1 to 3) | 1.9 (1.1 to 3.1) | 2 (1.2 to 3.2) | 2.1 (1.2 to 3.4) | 2.2 (1.3 to 3.5) | 2.2 (1.3 to 3.6) | 2.3 (1.3 to 3.7) | 2.3 (1.4 to 3.6) | 2.2 (1.3 to 3.5) | 2.2 (1.3 to 3.5) | 2.1 (1.3 to 3.5) | 2.1 (1.2 to 3.3) | 2 (1.2 to 3.2) | 1.9 (1.2 to 3.1) | 1.9 (1.2 to 2.9) | 1.8 (1.1 to 2.9) | 1.8 (1.1 to 2.7) | 1.7 (1.1 to 2.7) | 1.7 (1 to 2.6) | 1.7 (1 to 2.6) | 1.7 (1 to 2.7) | 1.7 (1 to 2.6) | 1.7 (1 to 2.7) |
|  |  | Maternal and neonatal disorders | 161.7 (111.3 to 226.5) | 163.5 (111.9 to 227.4) | 165.7 (114.4 to 230.2) | 168 (115 to 233.9) | 170.4 (115.6 to 235.6) | 173 (119.1 to 238.9) | 176.2 (121.9 to 242.2) | 180.6 (125.1 to 249.9) | 185.3 (127.2 to 254.6) | 189.6 (130 to 260) | 193 (131.5 to 264.3) | 195.5 (134.9 to 268) | 197.6 (135.7 to 271.4) | 199.7 (138.2 to 276.6) | 201.8 (140.2 to 277.9) | 204.2 (141.5 to 281.5) | 207 (142.5 to 286.1) | 210.6 (145.6 to 293.3) | 214.1 (148.5 to 298.3) | 217.6 (150.4 to 303.3) | 220.7 (151.8 to 306.9) | 223.8 (155.5 to 310.7) | 226.9 (155.3 to 313.7) | 229.7 (157.2 to 318.3) | 232.3 (160.1 to 321) | 233.8 (161.3 to 320.7) | 234.4 (163.2 to 315.3) | 234.2 (166.6 to 308) | 233.3 (169.1 to 302.6) | 232.5 (168.8 to 300.3) | 231.3 (165.8 to 296) | 230.6 (166.2 to 292.4) |
|  |  | Mental disorders | 1352.7 (972.9 to 1769.7) | 1378.5 (989.9 to 1808.4) | 1404.7 (1010.9 to 1843.3) | 1432.3 (1030.4 to 1883.9) | 1462.3 (1047 to 1923.7) | 1496.5 (1074.4 to 1972.8) | 1533.8 (1100.1 to 2022.4) | 1573.1 (1132.4 to 2078.7) | 1608.1 (1158.2 to 2120.1) | 1635.1 (1173.9 to 2153.2) | 1653.5 (1188.1 to 2180.4) | 1667.5 (1195.9 to 2201.9) | 1679.3 (1208.7 to 2221.7) | 1688.5 (1212.4 to 2233.2) | 1692.6 (1220.2 to 2242.3) | 1689 (1215.1 to 2240) | 1675.7 (1206.7 to 2214.5) | 1652.6 (1190.7 to 2185.4) | 1622 (1169.8 to 2142.2) | 1588.6 (1147.7 to 2098.2) | 1554.2 (1117.9 to 2050.5) | 1522.1 (1098.7 to 2009.1) | 1493.6 (1076.1 to 1967.1) | 1469.1 (1058.5 to 1931.2) | 1446.5 (1044.3 to 1905.4) | 1427.2 (1026.4 to 1882.2) | 1411.4 (1013.8 to 1855.8) | 1403.9 (1009.7 to 1842.8) | 1406.4 (1010.7 to 1840.3) | 1418.4 (1020.6 to 1861.1) | 1711.6 (1219.6 to 2281.3) | 1711.4 (1214.4 to 2294.1) |
|  |  | Musculoskeletal disorders | 313.5 (208 to 438.5) | 323.4 (213.2 to 451.2) | 333.2 (221.2 to 466.9) | 343.4 (227.8 to 477.2) | 355.1 (235.4 to 494) | 368.2 (243.1 to 512.7) | 381.9 (251.9 to 533.6) | 394.3 (260.9 to 547.8) | 404.5 (267.8 to 562.4) | 412.4 (272.2 to 572.7) | 418.4 (275 to 586.6) | 423.6 (280.4 to 591.6) | 428.4 (287.3 to 598.5) | 432.3 (290.3 to 605.2) | 434.4 (293.1 to 607) | 433.7 (291.9 to 607.5) | 430.7 (290.3 to 604.8) | 425.4 (287 to 598.6) | 418.1 (279.5 to 585.9) | 409 (273.5 to 571.3) | 398.1 (266.9 to 558.5) | 387.4 (260.9 to 541.9) | 377.1 (253.4 to 525) | 367.5 (246.1 to 512) | 358.9 (241 to 497.6) | 351.3 (235.4 to 485.8) | 345.4 (231.1 to 479.6) | 342.5 (229.6 to 474.1) | 342.6 (229.4 to 478.2) | 346 (232.5 to 484.8) | 351.9 (233.5 to 490) | 346.1 (232.1 to 484.4) |
|  |  | Neglected tropical diseases and malaria | 89 (56.9 to 137) | 86.8 (56.7 to 131.2) | 84.3 (55.2 to 126.1) | 82 (54.2 to 120.9) | 79.9 (52.4 to 116.7) | 78.2 (51.4 to 113.8) | 76.1 (50 to 110.6) | 73.8 (48.4 to 105.9) | 71.9 (47.8 to 103.3) | 70.3 (46.4 to 102.4) | 69.2 (45.8 to 100.9) | 68.3 (45.3 to 98.8) | 67.5 (44.9 to 98.1) | 66.9 (44.3 to 97.4) | 66.3 (44 to 96.8) | 66 (43.7 to 96.5) | 65.8 (43.3 to 96.4) | 65.8 (43.5 to 96.4) | 65.8 (43.3 to 96.2) | 65.8 (42.7 to 96.3) | 65.8 (42.7 to 96.5) | 65.3 (42.5 to 95.1) | 64.5 (42.2 to 94.3) | 63.4 (41.5 to 93) | 62.2 (40.2 to 91.9) | 61 (39.2 to 90.1) | 59.4 (38 to 88.3) | 57.4 (36.7 to 85.4) | 55.5 (35.3 to 83.5) | 54.3 (34.3 to 82.4) | 53.7 (34.2 to 82.6) | 52.5 (33.7 to 79.5) |
|  |  | Neoplasms | 6.3 (4.4 to 9.1) | 6.4 (4.5 to 9) | 6.6 (4.6 to 9.2) | 6.7 (4.7 to 9.2) | 6.8 (4.7 to 9.2) | 6.7 (4.7 to 9.1) | 6.7 (4.7 to 9) | 6.7 (4.8 to 9) | 6.8 (4.8 to 9.1) | 6.9 (4.8 to 9.2) | 7 (4.9 to 9.3) | 7.1 (5 to 9.6) | 7.3 (5.2 to 9.8) | 7.5 (5.3 to 10) | 7.6 (5.3 to 10.1) | 7.6 (5.3 to 10.2) | 7.7 (5.4 to 10.4) | 8 (5.6 to 10.8) | 8.4 (5.9 to 11.5) | 8.8 (6.1 to 12.2) | 9.1 (6.3 to 12.7) | 9.5 (6.5 to 13.3) | 10 (6.8 to 14.2) | 10.6 (7.1 to 15) | 10.4 (6.8 to 15.1) | 9.5 (6.2 to 13.9) | 8.9 (5.7 to 13.6) | 8.9 (5.7 to 13.5) | 8.8 (5.5 to 13.5) | 8.7 (5.4 to 13.4) | 8.3 (5.3 to 12.7) | 7.8 (5 to 11.7) |
|  |  | Neurological disorders | 524 (207.7 to 1012.1) | 533.4 (207.2 to 1033.1) | 542.4 (207.8 to 1056.7) | 552.5 (207.4 to 1088.2) | 563 (205.2 to 1111.1) | 574.6 (207.5 to 1141.1) | 583.5 (210.4 to 1151.1) | 588.1 (215.4 to 1160.2) | 589.4 (216.2 to 1156.9) | 588.7 (218.5 to 1158.6) | 589.6 (218.3 to 1161.6) | 592.3 (220.2 to 1159.1) | 594.1 (215.3 to 1163.8) | 595.9 (216.3 to 1168.9) | 596.6 (219 to 1170.8) | 595.6 (218.1 to 1168.5) | 593.2 (221.3 to 1168.3) | 589.1 (218.6 to 1162.6) | 583 (216.7 to 1158.2) | 575.2 (214.7 to 1140.7) | 563.9 (212.3 to 1115.9) | 551 (210.7 to 1085.9) | 537.7 (206.2 to 1050.4) | 523.2 (200.3 to 1020.6) | 510.9 (191.5 to 995.7) | 500.7 (185.6 to 969.3) | 492.5 (182.9 to 958.1) | 488.2 (177.9 to 958.5) | 488.4 (174.6 to 966.9) | 491.5 (168.7 to 976.1) | 498 (165.1 to 987.1) | 521 (171 to 1046.8) |
|  |  | Nutritional deficiencies | 681.7 (447.5 to 975.5) | 644.8 (425.3 to 924.7) | 611.7 (400.6 to 867.4) | 581.6 (378.3 to 826.3) | 554.9 (358.8 to 799.9) | 531.3 (344.9 to 769.4) | 511.1 (329.4 to 737.3) | 492.5 (314.4 to 709.3) | 476.4 (303.6 to 689.3) | 462.8 (296 to 670.4) | 451.9 (291 to 656) | 443.1 (287.2 to 638.7) | 435.6 (284.1 to 628.4) | 429 (279.5 to 620.2) | 423 (276 to 613.1) | 418.4 (271.4 to 605.8) | 414.7 (269.6 to 600.9) | 411.9 (267.9 to 596.6) | 410.2 (269.1 to 592.4) | 409.3 (266.8 to 591.6) | 408.5 (263.9 to 591.6) | 407 (263.9 to 597.3) | 406.3 (264.6 to 603.5) | 404.5 (264.2 to 607.6) | 400.9 (258.4 to 606.8) | 396.1 (252.1 to 600.4) | 387.4 (246.1 to 592.5) | 374.6 (236 to 577.3) | 360.7 (227.3 to 559.3) | 349.4 (218.7 to 546.4) | 341.3 (212.6 to 533.2) | 329.4 (204.5 to 515.2) |
|  |  | Other infectious diseases | 67.1 (44.8 to 96.3) | 63.6 (42.8 to 90.4) | 59.9 (40.7 to 84.9) | 57.2 (39.1 to 80.7) | 55 (37.5 to 77.8) | 53.3 (36.2 to 74.9) | 51.9 (35.5 to 73.1) | 50.4 (34.2 to 71.1) | 49.1 (33.4 to 69.2) | 48.1 (32.6 to 67.9) | 47.3 (32.3 to 66.4) | 46.4 (31.7 to 65.5) | 45.5 (30.9 to 64.4) | 44.5 (29.9 to 62.1) | 43.6 (29.3 to 61) | 43.1 (29.2 to 60.1) | 42.7 (28.8 to 59.7) | 42.3 (28.3 to 59.9) | 42.1 (28.1 to 59.4) | 42 (27.9 to 59.7) | 41.9 (27.6 to 60.2) | 41.8 (27.7 to 59.9) | 41.7 (27.8 to 60.1) | 41.5 (27.6 to 60.6) | 41.2 (27.1 to 60.6) | 40.9 (27 to 60.6) | 40.4 (26.6 to 59.8) | 39.7 (26.1 to 58.8) | 38.8 (25.6 to 57.7) | 38.1 (25.1 to 57) | 37.5 (24.4 to 55.4) | 36.6 (23.9 to 54.2) |
|  |  | Other non-communicable diseases | 484.1 (340.6 to 664.4) | 478.2 (336.8 to 658) | 472.9 (332.6 to 651.1) | 468.4 (329.9 to 646.7) | 464.9 (328 to 644) | 462.2 (326.9 to 643.5) | 460.8 (326.3 to 641.7) | 460.3 (327.1 to 639.8) | 460.1 (326.5 to 639) | 460.1 (327.2 to 638.2) | 459.9 (328.2 to 638.5) | 460 (328.1 to 638) | 460.7 (327.9 to 637.8) | 461.7 (328.2 to 638.5) | 462.1 (329.3 to 640.2) | 461.5 (328.5 to 637.8) | 460.1 (326 to 637.6) | 458.2 (324.4 to 635.9) | 455.9 (321.8 to 630.2) | 453.5 (320 to 626.5) | 451.3 (319.2 to 625.2) | 447.2 (317 to 621.4) | 440.7 (312.5 to 612.7) | 433.1 (306.8 to 603.3) | 425.9 (303.2 to 595.4) | 420.7 (298.1 to 589.5) | 416.1 (295.2 to 584.3) | 410.6 (291.2 to 576.1) | 405.4 (287.5 to 570.9) | 401.9 (286.1 to 566.9) | 399.4 (285.4 to 562.6) | 398.6 (285 to 561.6) |
|  |  | Respiratory infections and tuberculosis | 136.6 (86.3 to 204.3) | 135.1 (85.3 to 202.9) | 133.7 (84.1 to 199.8) | 132.3 (82.9 to 198.8) | 131 (81.7 to 197.1) | 129.8 (80.9 to 195.6) | 128.6 (79.8 to 194) | 127.4 (79 to 192.1) | 126.2 (78.3 to 191.4) | 125.3 (77.4 to 190.4) | 124.7 (77.4 to 188.5) | 124.2 (76.8 to 188.3) | 123.8 (76.9 to 187.8) | 123.6 (76.9 to 187.1) | 123.4 (76.8 to 186.2) | 123.4 (77.1 to 186.1) | 123.3 (76.6 to 186.5) | 123.3 (76.6 to 186.1) | 123.4 (76.5 to 185.9) | 123.6 (76.3 to 186.6) | 124 (76.5 to 187.1) | 124.7 (77.3 to 187.8) | 125.4 (77.3 to 189.1) | 126 (77.6 to 190.1) | 126.4 (78.4 to 191) | 126.7 (78.3 to 191.3) | 126.9 (78.1 to 191.6) | 126.9 (78 to 192.4) | 126.6 (78.2 to 191.2) | 126.1 (77.7 to 191.3) | 165.4 (102.4 to 257.6) | 230.5 (129.2 to 420.1) |
|  |  | Self-harm and interpersonal violence | 64.5 (47.8 to 84.8) | 56.9 (41.9 to 74.5) | 51.8 (38 to 67.6) | 49.3 (36 to 64.4) | 48 (35.3 to 62.3) | 46.5 (34.2 to 59.8) | 45.9 (34 to 58.7) | 45.1 (33.5 to 57.3) | 44.2 (33 to 55.9) | 43.8 (32.3 to 55.2) | 42.4 (31.4 to 53.5) | 41.6 (30.6 to 52.9) | 40 (29.6 to 51) | 39.3 (29 to 49.8) | 38.5 (28.5 to 49) | 37.2 (27.4 to 47.2) | 36.4 (26.9 to 46.4) | 36.4 (26.9 to 46.5) | 36.3 (26.8 to 46.3) | 35.7 (26.5 to 45.5) | 34.9 (25.8 to 44.5) | 33.7 (25 to 43) | 32.6 (24.2 to 41.6) | 31.6 (23.5 to 40.5) | 30.6 (22.7 to 39.3) | 30 (22.2 to 38.3) | 29.9 (22.2 to 38.4) | 29.6 (21.9 to 37.8) | 28.6 (21.3 to 36.5) | 27.2 (20.3 to 34.7) | 28.6 (21.3 to 36.6) | 29 (21.7 to 37) |
|  |  | Sense organ diseases | 192.7 (132.7 to 270.4) | 194.6 (133.8 to 273.4) | 196.3 (134.8 to 276.6) | 198.2 (136.3 to 278.8) | 200.3 (138 to 282.4) | 202.7 (139.6 to 285.7) | 205.4 (141.4 to 289.2) | 208.2 (143.6 to 292.6) | 210.7 (144.9 to 295.7) | 212.6 (146.4 to 297.6) | 213.8 (147.5 to 299.2) | 214.4 (147 to 301.5) | 214.3 (147.5 to 302.1) | 214.1 (147.7 to 302.4) | 213.5 (147.2 to 302.8) | 212.6 (146.6 to 302.7) | 211.2 (145.1 to 298.5) | 209.4 (144.1 to 296.4) | 207.1 (142.5 to 291.3) | 204.3 (141.1 to 285.8) | 201.5 (138.1 to 280.9) | 199 (136.8 to 276.2) | 196.9 (135.7 to 274.9) | 195.1 (134.3 to 273.5) | 193.6 (133.4 to 271.8) | 192.2 (132.6 to 270) | 190.7 (130.7 to 267.8) | 189.3 (130.4 to 266.4) | 188.6 (129.3 to 265.4) | 189.3 (130.2 to 267.4) | 191.5 (131.6 to 269.2) | 192.7 (131.7 to 271) |
|  |  | Skin and subcutaneous diseases | 461.4 (298.7 to 669.9) | 465 (300.5 to 673.2) | 468.4 (302.3 to 679.5) | 471.9 (306.2 to 682.3) | 475.7 (307.9 to 689.7) | 479.5 (312.3 to 697.6) | 483.6 (315 to 705.3) | 487.9 (318.5 to 711) | 491.6 (321.5 to 717) | 494.9 (323.3 to 719) | 497.8 (326.5 to 723.4) | 500.7 (328.1 to 729.1) | 503.8 (330.2 to 734.2) | 507 (332.5 to 740.3) | 509.8 (334.2 to 744.7) | 511.7 (334.7 to 749.1) | 512.8 (336 to 754.2) | 513 (334.8 to 754) | 512.4 (336.2 to 751.6) | 511.4 (334.2 to 750) | 509.9 (333.7 to 747.5) | 508.3 (332.4 to 745.2) | 506.7 (330 to 742.9) | 505.2 (328.6 to 741.3) | 503.9 (327.8 to 738.8) | 502.8 (325.9 to 738.4) | 502.9 (326.7 to 737.9) | 504.3 (326.6 to 738.6) | 506.4 (328.1 to 741.2) | 509 (332 to 747.1) | 511.5 (334 to 748.8) | 512.9 (333.3 to 744.9) |
|  |  | Substance use disorders | 24.8 (16 to 33.9) | 26 (17 to 36.2) | 27.2 (17.8 to 37.5) | 28.5 (18.2 to 39.3) | 30.2 (19.3 to 41.4) | 32.6 (20.6 to 44.8) | 35.8 (23.4 to 49.5) | 39.3 (25.1 to 54.5) | 42.8 (27.3 to 59.6) | 45.9 (29.4 to 63.9) | 48.4 (30.9 to 67.9) | 50.9 (32.8 to 71.8) | 53.2 (34.4 to 74.3) | 55.3 (35.1 to 77.5) | 56.9 (36.2 to 79.8) | 57.6 (37 to 80.5) | 57.5 (36.9 to 80) | 56.1 (35.7 to 76.9) | 54.2 (34.8 to 74.9) | 52 (33.7 to 72) | 49.9 (32.1 to 69.3) | 47.5 (30.5 to 66.5) | 44.9 (28.8 to 61.9) | 42.3 (27.3 to 58.3) | 40 (26.1 to 55.3) | 38.1 (24.5 to 52.3) | 36.9 (23.9 to 50.6) | 36.5 (23.7 to 49.7) | 36.5 (23.5 to 49.8) | 36.7 (23.5 to 49.8) | 37 (23.9 to 50.7) | 37.2 (24.3 to 51) |
|  |  | Transport injuries | 150.6 (108.9 to 201.3) | 148 (106.7 to 198.4) | 145.4 (105.2 to 195.3) | 142.9 (103.5 to 192.4) | 140.6 (101.8 to 189.5) | 138.6 (100.3 to 187.1) | 136.9 (99.2 to 184.7) | 135.1 (98 to 181.9) | 133.2 (96.5 to 179.9) | 130.9 (95.2 to 176.3) | 128.5 (93.7 to 173.1) | 125.8 (91.7 to 169.8) | 122.9 (88.7 to 166.6) | 119.6 (86.6 to 161.9) | 115.9 (84.1 to 157.4) | 111.7 (80.7 to 151.5) | 106.9 (76.8 to 145.9) | 101.3 (73 to 138.2) | 95.3 (68.8 to 129.5) | 89 (64.1 to 121.2) | 82.7 (59.5 to 112.6) | 75.8 (54.6 to 103.8) | 68.5 (49.4 to 93.6) | 61.3 (44.2 to 83.6) | 54.7 (39.4 to 74.7) | 49.3 (35.6 to 67.2) | 44.6 (32.2 to 60.8) | 40.2 (29 to 54.8) | 36.9 (26.6 to 50.7) | 35.6 (25.5 to 48.9) | 35.8 (25.6 to 49) | 35.9 (25.4 to 49.1) |
|  |  | Unintentional injuries | 180 (131.1 to 238.6) | 166.2 (122.4 to 222.6) | 165.8 (121.9 to 222.2) | 166.2 (121.5 to 223.5) | 165.8 (121.5 to 223.9) | 166 (121.3 to 224.8) | 166.4 (120.6 to 226.1) | 168.5 (121.9 to 229.7) | 167.3 (120.9 to 228.4) | 166.3 (119.8 to 228.1) | 164.9 (118.9 to 226.8) | 163.6 (116.9 to 225.7) | 161.9 (115.6 to 222.6) | 178 (129.8 to 240.8) | 165.6 (121.9 to 225.8) | 162.3 (118.6 to 221.9) | 157.6 (114.8 to 215.7) | 152 (110.7 to 208.6) | 145.6 (105.8 to 200.4) | 138.3 (99.2 to 190.8) | 130.6 (93.4 to 181.6) | 123.7 (88.1 to 172.4) | 116.5 (83 to 162.8) | 108.8 (77.2 to 152.9) | 102.2 (72.9 to 143.6) | 96.8 (68.9 to 136) | 92.4 (65.5 to 130.1) | 89 (63 to 124.9) | 85.8 (60.6 to 121.1) | 84.6 (59.6 to 119.4) | 85 (59.6 to 120.5) | 85.5 (59.5 to 121.2) |
| YLDs (Years Lived with Disability) | Females | Causes | Year |  |  |  |  |  |  |  |  |  |  |  |  |  |  |  |  |  |  |  |  |  |  |  |  |  |  |  |  |  |  |  |
|  |  |  | 1990 | 1991 | 1992 | 1993 | 1994 | 1995 | 1996 | 1997 | 1998 | 1999 | 2000 | 2001 | 2002 | 2003 | 2004 | 2005 | 2006 | 2007 | 2008 | 2009 | 2010 | 2011 | 2012 | 2013 | 2014 | 2015 | 2016 | 2017 | 2018 | 2019 | 2020 | 2021 |
|  |  | Cardiovascular diseases | 77.9 (54 to 108.8) | 78.9 (54.4 to 109.3) | 79.8 (55.4 to 110.8) | 80.8 (56.1 to 112.6) | 81.7 (56.5 to 114.3) | 82.6 (57 to 115.8) | 83.5 (58.5 to 117.1) | 84.3 (58.5 to 118.2) | 85.1 (59.8 to 118.4) | 85.6 (59.6 to 119.7) | 86 (59.7 to 119.9) | 86.4 (59.2 to 120.7) | 87 (60.2 to 122) | 87.7 (61.1 to 122.8) | 88.3 (61.1 to 125) | 88.4 (61.4 to 124.2) | 88.1 (60.1 to 123.4) | 87.7 (60.8 to 122.1) | 86.8 (59.1 to 123.3) | 85.9 (57.9 to 121.4) | 84.6 (57 to 119.4) | 83.1 (56.2 to 117.4) | 81.7 (55.1 to 116.2) | 80.3 (54 to 113.1) | 79.1 (53.1 to 112.2) | 78.2 (52.8 to 110.6) | 77.3 (51.8 to 110) | 76.6 (51.7 to 108.6) | 76.4 (50.6 to 108.5) | 76.7 (51 to 108) | 77.3 (52.1 to 109.3) | 77.9 (51.1 to 110.5) |
|  |  | Chronic respiratory diseases | 193.7 (118.6 to 297.1) | 189.6 (115.6 to 288.9) | 185.5 (112.1 to 283) | 182.2 (110.6 to 278.5) | 179.6 (107.6 to 273.2) | 177.7 (106.7 to 270.1) | 176.1 (105.8 to 267.9) | 174.5 (104 to 265.7) | 173.1 (103.7 to 262.2) | 171.9 (103.8 to 262.2) | 171.1 (103.3 to 263.3) | 170 (102.5 to 259.6) | 168.8 (101.2 to 256.6) | 167.5 (101 to 254.8) | 166.4 (100.3 to 253.9) | 165.5 (99.7 to 254) | 164.8 (99.1 to 251.5) | 163.6 (98.5 to 251.2) | 162 (97.8 to 250.6) | 160.5 (96.1 to 246.9) | 159 (95.5 to 243.9) | 156.9 (95 to 241.1) | 154.4 (93.1 to 238.3) | 151.1 (91.4 to 235.6) | 148.2 (88.4 to 231.7) | 145.9 (87.6 to 230.2) | 143.9 (85.7 to 225.9) | 141.8 (85 to 222.8) | 140.1 (83.1 to 218.4) | 139.3 (83.3 to 217.1) | 142 (84.1 to 223.7) | 140.5 (82.9 to 221.7) |
|  |  | Diabetes and kidney diseases | 17.2 (11.7 to 24.4) | 17.4 (11.9 to 24.9) | 17.6 (12 to 24.9) | 17.8 (12.2 to 25.3) | 18 (12.3 to 25.4) | 18.3 (12.4 to 26) | 18.7 (12.7 to 26.8) | 19.1 (13 to 27.2) | 19.5 (13.3 to 27.5) | 19.7 (13.4 to 28.1) | 19.9 (13.5 to 28.1) | 20 (13.6 to 28.3) | 20 (13.7 to 28.4) | 20.1 (13.9 to 28.3) | 20.1 (14 to 28.5) | 20.2 (13.9 to 28.5) | 20.4 (14 to 29) | 20.6 (14.2 to 29.3) | 20.7 (14.1 to 29.5) | 20.7 (14.2 to 29.2) | 20.5 (14.1 to 29.3) | 20 (13.8 to 28.5) | 19.5 (13.4 to 27.6) | 18.8 (12.9 to 26.9) | 18.3 (12.5 to 26.3) | 17.9 (12.2 to 25.8) | 17.6 (11.8 to 25.2) | 17.4 (11.7 to 24.7) | 17.3 (11.6 to 24.5) | 17.4 (11.7 to 24.9) | 17.7 (12.1 to 25.3) | 18.1 (12.2 to 25.9) |
|  |  | Digestive diseases | 59.5 (41.8 to 81.4) | 59.1 (41.5 to 81.3) | 58.6 (40.9 to 81.5) | 58.1 (40.3 to 79.4) | 57.9 (40.2 to 79.1) | 57.9 (41.1 to 79.2) | 58.2 (40.7 to 80.4) | 58.9 (40.7 to 81.4) | 59.6 (41.1 to 82.1) | 60.1 (42 to 82.9) | 60.6 (42.2 to 83.6) | 60.9 (42 to 84.4) | 61.2 (42.5 to 84.9) | 61.5 (42.5 to 85.1) | 61.6 (42.5 to 84.9) | 61.6 (42.4 to 84.1) | 61.8 (42.1 to 84.4) | 61.8 (42.4 to 85.2) | 61.7 (42.2 to 84.6) | 61.5 (42.6 to 84.4) | 61 (41.7 to 83.9) | 60 (41 to 82.2) | 58.6 (39.5 to 80.6) | 57.1 (39.5 to 78.4) | 55.6 (37.9 to 76.3) | 54.5 (37.4 to 74.7) | 53.5 (36.9 to 73.4) | 52.6 (36 to 72.1) | 52 (35.5 to 71.1) | 51.9 (35.4 to 71.3) | 52.2 (35.5 to 71.8) | 51.9 (35.6 to 71) |
|  |  | Enteric infections | 227.5 (146.8 to 330.9) | 212 (135.3 to 311.3) | 197.8 (127 to 291.4) | 184.7 (117.4 to 272.6) | 172.8 (108 to 254.6) | 162 (101.2 to 240) | 152.4 (95.1 to 227.5) | 143.4 (89.3 to 212.7) | 135.4 (84.9 to 202.9) | 128.7 (80.1 to 192.7) | 123.1 (75.6 to 182.3) | 118.2 (73 to 177.1) | 113.9 (70.4 to 171.1) | 110 (67.5 to 166.7) | 106.4 (66 to 162.3) | 103.5 (64.1 to 157.4) | 101.2 (61.7 to 152.6) | 99.3 (60.9 to 150.3) | 97.6 (59.3 to 146.1) | 95.8 (57.5 to 142.7) | 93.6 (56.2 to 140) | 90.9 (54.6 to 137) | 88.2 (53.8 to 132.2) | 85.3 (52.5 to 128.3) | 82.6 (50.5 to 124.4) | 80.1 (49.2 to 120.9) | 77.7 (47.1 to 117.4) | 75.3 (45.2 to 113.8) | 73.7 (44.2 to 111.8) | 73.5 (44 to 111.3) | 74.2 (44.7 to 113.8) | 74.8 (45 to 114.6) |
|  |  | HIV/AIDS and sexually transmitted infections | 0.9 (0.5 to 1.5) | 0.9 (0.5 to 1.5) | 0.9 (0.5 to 1.5) | 0.9 (0.5 to 1.6) | 0.9 (0.5 to 1.6) | 1 (0.5 to 1.7) | 1 (0.6 to 1.8) | 1.1 (0.6 to 2) | 1.2 (0.7 to 2.1) | 1.3 (0.7 to 2.2) | 1.4 (0.8 to 2.4) | 1.5 (0.8 to 2.5) | 1.5 (0.9 to 2.6) | 1.6 (0.9 to 2.8) | 1.7 (1 to 2.9) | 1.8 (1 to 2.9) | 1.8 (1 to 2.9) | 1.7 (1 to 2.8) | 1.7 (1 to 2.8) | 1.6 (1 to 2.7) | 1.6 (0.9 to 2.6) | 1.5 (0.9 to 2.5) | 1.5 (0.9 to 2.4) | 1.4 (0.9 to 2.3) | 1.4 (0.9 to 2.2) | 1.3 (0.8 to 2.1) | 1.3 (0.8 to 2) | 1.2 (0.8 to 1.9) | 1.2 (0.7 to 1.9) | 1.2 (0.7 to 1.9) | 1.2 (0.7 to 1.9) | 1.2 (0.7 to 1.9) |
|  |  | Maternal and neonatal disorders | 160.4 (109.5 to 225) | 162.2 (109.8 to 227.2) | 164.5 (112.4 to 232.4) | 166.7 (114.2 to 235.6) | 169.1 (114.9 to 239) | 171.8 (117.8 to 239.7) | 175.5 (119.5 to 244.7) | 180 (124.2 to 253.2) | 184.9 (127.3 to 260.4) | 189.2 (131.1 to 262.9) | 192.2 (131.9 to 268) | 194.3 (134.3 to 269.7) | 195.6 (134 to 270.2) | 196.9 (135.5 to 271.6) | 198.1 (136.9 to 273.7) | 199.8 (138.1 to 274.8) | 202.2 (139.2 to 279.4) | 205.4 (142 to 284.1) | 208.5 (144.3 to 286.3) | 211.4 (146 to 292.7) | 214.1 (147.2 to 294.7) | 216.8 (151.3 to 297) | 219.2 (152.1 to 300.1) | 221.5 (152.9 to 302.5) | 223.5 (154.2 to 304) | 224.8 (154.6 to 305.2) | 225.1 (158.9 to 300.8) | 224.3 (159.7 to 292.9) | 223.3 (161.7 to 289.2) | 222.4 (161.3 to 288.4) | 221.2 (159.1 to 284) | 220.9 (159.2 to 281.9) |
|  |  | Mental disorders | 1381.7 (983.5 to 1817.7) | 1409.1 (1004.7 to 1862) | 1438.3 (1022.9 to 1893.7) | 1470 (1045.6 to 1945.8) | 1505.6 (1069.7 to 1992.7) | 1545.8 (1098.8 to 2048.4) | 1588.5 (1129 to 2107.6) | 1632.3 (1160.4 to 2161) | 1670.4 (1189.2 to 2215.5) | 1699.9 (1204.8 to 2261.7) | 1720.8 (1220.2 to 2287.5) | 1737.3 (1230.9 to 2304) | 1752.1 (1240.6 to 2331.4) | 1764.7 (1248.8 to 2345.1) | 1771 (1256.1 to 2354.5) | 1768.4 (1253.1 to 2343.8) | 1756.2 (1248.2 to 2339.7) | 1734.9 (1234.9 to 2307.7) | 1706.3 (1214.3 to 2272.3) | 1673.1 (1191.8 to 2226.2) | 1636.8 (1161.9 to 2173) | 1602.1 (1142.1 to 2146.2) | 1571.5 (1111.2 to 2098.8) | 1544.9 (1097.2 to 2059.5) | 1519.9 (1074 to 2026.9) | 1496.7 (1056.6 to 1999) | 1476.3 (1045.2 to 1962.4) | 1465.6 (1040.5 to 1945.7) | 1465.9 (1042.2 to 1937.6) | 1478.4 (1051.9 to 1956.1) | 1842 (1294.2 to 2459.8) | 1831.6 (1289.1 to 2460.6) |
|  |  | Musculoskeletal disorders | 428.5 (285.5 to 599.4) | 441.9 (293.3 to 616.4) | 455.6 (304.3 to 637.3) | 470.2 (313 to 654.5) | 486.7 (324.2 to 678) | 504.9 (335.5 to 703.4) | 523.9 (349.5 to 731.7) | 541.2 (361.7 to 754.8) | 556.1 (372.8 to 775.4) | 567.4 (379.1 to 794.1) | 576.2 (383.9 to 806.5) | 583.2 (391.1 to 818.9) | 589.6 (399.5 to 829.1) | 594.6 (403.2 to 840) | 597.4 (405.5 to 843.2) | 596.2 (406.2 to 840.7) | 592.3 (402.9 to 833.7) | 585.3 (396.2 to 825.1) | 575.6 (389.7 to 810) | 563.2 (379.1 to 787.9) | 548.3 (366.2 to 766.2) | 533.6 (359.1 to 750) | 518.8 (348.3 to 723.8) | 505.6 (339.7 to 706.5) | 493.3 (333.3 to 691.9) | 482.8 (326.1 to 672.8) | 474.3 (318.4 to 660.5) | 470.5 (316.5 to 657.4) | 470.6 (314.7 to 657.9) | 475.1 (319.1 to 660.4) | 482.6 (322.5 to 669.2) | 469.6 (314.5 to 654.1) |
|  |  | Neglected tropical diseases and malaria | 89.4 (54.4 to 142.6) | 88.3 (54.4 to 139.5) | 86.8 (53.6 to 136.5) | 85.4 (54.2 to 131.4) | 84.1 (53.7 to 127.6) | 83.1 (53.1 to 125.1) | 81.7 (52.2 to 121.3) | 79.8 (51 to 118.7) | 78.5 (49.7 to 116.2) | 77.3 (48.6 to 114.7) | 76.5 (48.7 to 113.7) | 76 (48.5 to 112.6) | 75.6 (49 to 111.9) | 75.2 (48.8 to 111.5) | 74.9 (48.6 to 110.7) | 74.7 (48.2 to 110.7) | 74.7 (48.4 to 110) | 74.7 (48.5 to 110.5) | 74.8 (48.7 to 110.5) | 74.7 (48.7 to 110.6) | 74.7 (48.8 to 110.8) | 74.3 (48.9 to 110.4) | 73.5 (48.2 to 110.1) | 72.4 (47.5 to 108) | 71.3 (46.9 to 106.2) | 70.3 (46.1 to 105.5) | 69 (44.9 to 103.7) | 67.2 (43.8 to 102.2) | 65.6 (42.9 to 100.6) | 64.6 (42.5 to 99.9) | 64.1 (42.5 to 98) | 63 (41.8 to 97) |
|  |  | Neoplasms | 6.1 (4.1 to 8.5) | 6.1 (4.2 to 8.7) | 6.3 (4.3 to 8.7) | 6.4 (4.4 to 8.8) | 6.4 (4.5 to 8.9) | 6.4 (4.5 to 8.8) | 6.4 (4.5 to 8.6) | 6.4 (4.5 to 8.6) | 6.4 (4.5 to 8.6) | 6.5 (4.5 to 8.7) | 6.6 (4.6 to 8.9) | 6.7 (4.7 to 9) | 6.8 (4.8 to 9.2) | 6.9 (4.9 to 9.3) | 7 (4.9 to 9.4) | 7 (4.9 to 9.3) | 7.2 (5.1 to 9.6) | 7.4 (5.2 to 10.1) | 7.8 (5.4 to 10.6) | 8.2 (5.6 to 11.3) | 8.4 (5.8 to 11.8) | 8.8 (6.1 to 12.3) | 9.3 (6.3 to 13.1) | 9.9 (6.7 to 13.6) | 9.6 (6.5 to 13.7) | 8.8 (5.7 to 12.8) | 8.2 (5.3 to 12.1) | 8.1 (5.3 to 12.2) | 8 (5.1 to 12.3) | 7.9 (4.9 to 12.3) | 7.7 (4.8 to 11.6) | 7.3 (4.5 to 10.7) |
|  |  | Neurological disorders | 556.2 (198.4 to 1088.7) | 566.9 (199.9 to 1115.2) | 577.5 (198.5 to 1139.8) | 589.4 (197.8 to 1178.3) | 601.9 (196.2 to 1216.9) | 615.2 (200.2 to 1255.1) | 624.5 (201.2 to 1275) | 627 (208.5 to 1265.5) | 625.6 (208.5 to 1255.1) | 622.6 (213.6 to 1244.8) | 623.4 (215.1 to 1252) | 627.9 (212.6 to 1261.7) | 632.1 (212.6 to 1279.7) | 636.7 (213.4 to 1301.9) | 639.8 (214.2 to 1298.3) | 639.3 (212.9 to 1308) | 638.3 (216.9 to 1296.3) | 636.8 (217 to 1297.1) | 633.8 (210.5 to 1289.4) | 627.6 (209.8 to 1272.9) | 615.9 (204.3 to 1243.6) | 601.5 (205.8 to 1207.9) | 587 (201.2 to 1172.1) | 570.8 (192.7 to 1146.4) | 557.1 (190 to 1123.7) | 545.6 (184 to 1091.4) | 537.9 (181 to 1083.3) | 536 (177 to 1085.6) | 539.2 (176.4 to 1107.4) | 544.1 (172.1 to 1128.5) | 551.4 (164.6 to 1131.9) | 575.6 (166.3 to 1196.7) |
|  |  | Nutritional deficiencies | 685.2 (421 to 1020.9) | 658.1 (401.6 to 981.6) | 633.8 (384.5 to 955.7) | 611.7 (370.9 to 923) | 592.5 (364.2 to 883.6) | 576 (353.3 to 852.2) | 562.7 (342.8 to 833.3) | 550.7 (336.2 to 819.5) | 540.4 (329.6 to 808.4) | 531.9 (321.7 to 797.6) | 525 (319.7 to 793.7) | 519.9 (318.4 to 776.1) | 515.5 (318.1 to 777.1) | 512 (314.9 to 777.4) | 508.5 (310.1 to 773) | 505.7 (308.7 to 770.6) | 503.1 (308 to 760.1) | 500.6 (305.6 to 751.5) | 498.8 (307.5 to 746.6) | 497.7 (309.2 to 746.7) | 496.7 (310.4 to 739) | 495.2 (310.7 to 735.6) | 494.6 (311.1 to 745.6) | 493.1 (310.7 to 745.8) | 490.3 (307.2 to 745.8) | 486.7 (307 to 740.1) | 479.9 (303.1 to 733.9) | 469.5 (297.4 to 729.5) | 458.1 (291.4 to 716.6) | 448.5 (286.4 to 708.5) | 441.5 (281.7 to 697.2) | 430.1 (270.1 to 681) |
|  |  | Other infectious diseases | 69.4 (46.1 to 100.1) | 66.4 (43.7 to 96.3) | 63.1 (41.3 to 91.6) | 60.8 (40 to 88.7) | 59.1 (38.6 to 86.7) | 57.9 (37.4 to 84.9) | 56.9 (36.6 to 83.1) | 56 (36.1 to 80.8) | 55.1 (35.4 to 79.5) | 54.4 (34.7 to 78.9) | 53.9 (34.3 to 77.9) | 53.2 (33.7 to 76.5) | 52.6 (33.8 to 75.5) | 51.8 (33.2 to 75.1) | 51.1 (32.8 to 73.9) | 50.7 (32.2 to 74.3) | 50.4 (32.3 to 72.4) | 50 (32.5 to 73) | 49.8 (32.3 to 71.9) | 49.7 (32.3 to 72.4) | 49.5 (32.3 to 72.2) | 49.4 (32.6 to 71.7) | 49.2 (32.5 to 72.1) | 49 (32.5 to 72.3) | 48.8 (32 to 72.7) | 48.6 (31.7 to 71.9) | 48.2 (31.6 to 72.2) | 47.7 (31.2 to 72.2) | 46.9 (30.7 to 71.7) | 46.3 (30.3 to 71.1) | 45.8 (29.6 to 69.9) | 44.8 (29 to 68.1) |
|  |  | Other non-communicable diseases | 527.1 (366.8 to 738.7) | 524.6 (366.8 to 735.8) | 522.3 (365.2 to 730.1) | 520.9 (365.7 to 723.9) | 520.7 (364.9 to 726.9) | 522 (366.7 to 730.4) | 524 (370.5 to 732.7) | 526.7 (372.4 to 735.9) | 528.8 (376.1 to 737.2) | 530.7 (376.4 to 740.2) | 532.3 (377.8 to 741.9) | 534.9 (380.3 to 746.4) | 538.2 (381.7 to 749.4) | 541.7 (384.7 to 752.9) | 543.9 (384.7 to 755.7) | 543.8 (385 to 753.4) | 541.4 (384.1 to 748.4) | 537.2 (381.9 to 746.6) | 531.8 (378.7 to 738.9) | 526 (371.7 to 728.3) | 520.7 (367.7 to 724.8) | 513.8 (363.8 to 716.6) | 504.8 (357.8 to 703) | 494.7 (349.5 to 691.2) | 485.6 (344.9 to 682) | 479 (341.4 to 670.3) | 473.2 (337.7 to 664.9) | 466.8 (333 to 654.5) | 461.4 (330.9 to 647.6) | 458.9 (328.3 to 644.3) | 458.9 (330.3 to 644.1) | 459.1 (328.1 to 645.4) |
|  |  | Respiratory infections and tuberculosis | 134.7 (84.7 to 199.7) | 133.1 (84.1 to 197.9) | 131.6 (83 to 196.2) | 130.1 (81.5 to 195.3) | 128.7 (80.2 to 193.5) | 127.4 (79.9 to 191.5) | 126.2 (78.6 to 190.2) | 124.9 (78.1 to 188.6) | 123.7 (77 to 187.5) | 122.8 (76.4 to 185.7) | 122.1 (76.3 to 184.8) | 121.7 (76.2 to 184.4) | 121.4 (75.8 to 183.6) | 121.2 (76 to 182.6) | 121 (76.1 to 182.2) | 121.1 (75.9 to 182.5) | 121.1 (76.2 to 183.5) | 121 (76.4 to 182.4) | 121 (75.2 to 181.8) | 121.2 (74.9 to 183) | 121.6 (75.1 to 184) | 122.2 (76.2 to 184.9) | 122.8 (76.1 to 185.3) | 123.3 (76.6 to 186.4) | 123.6 (76.9 to 186.2) | 123.9 (77.1 to 187) | 124 (76.7 to 187.6) | 123.8 (76.6 to 187.2) | 123.4 (76.5 to 185.9) | 122.9 (75.9 to 186.8) | 162.4 (100.9 to 255.8) | 228.1 (127.9 to 418.1) |
|  |  | Self-harm and interpersonal violence | 47.5 (34.9 to 62) | 47.1 (34.6 to 61.5) | 45.9 (33.7 to 59.8) | 45.1 (33 to 58.7) | 44.3 (32.5 to 56.8) | 43.4 (31.9 to 55.9) | 43.2 (31.8 to 55.5) | 42.8 (31.4 to 55.4) | 42.4 (31 to 54.5) | 42.2 (30.8 to 54.4) | 41.3 (30.2 to 53.6) | 40.9 (29.8 to 53) | 39.8 (28.9 to 52.1) | 39.1 (28.4 to 51.7) | 38.3 (27.9 to 50.9) | 37 (26.9 to 49.7) | 36.2 (26.3 to 48.8) | 36.3 (26.4 to 48.6) | 36 (26.2 to 48.2) | 35.3 (25.6 to 47) | 34.5 (25.1 to 45.9) | 33.5 (24.4 to 44.5) | 32.6 (23.6 to 43.3) | 31.7 (23 to 41.9) | 30.9 (22.4 to 40.8) | 30.2 (21.9 to 39.8) | 30 (21.8 to 39.3) | 29.7 (21.4 to 39.1) | 29 (20.9 to 38.4) | 28 (20.2 to 37.7) | 29 (20.8 to 38.8) | 29.5 (21.2 to 39.5) |
|  |  | Sense organ diseases | 194.5 (134.4 to 274.2) | 196 (135.3 to 277.3) | 197.5 (135.9 to 279.5) | 199.3 (137.7 to 281.7) | 201.4 (139.8 to 284.8) | 203.7 (141.5 to 286.6) | 206.4 (143 to 290.7) | 209.3 (145.3 to 291.9) | 211.9 (145.4 to 296.6) | 213.9 (147.7 to 298.7) | 215 (148.7 to 297.4) | 215.5 (148.9 to 299.4) | 215.2 (149 to 300.2) | 214.6 (148.4 to 299.5) | 213.9 (147.9 to 298.9) | 212.9 (147.1 to 299) | 211.8 (146.7 to 295.9) | 210.3 (145.8 to 296.2) | 208.3 (144 to 290.9) | 205.9 (142.1 to 287.5) | 203.4 (140.8 to 286.1) | 201.1 (139.3 to 281.9) | 199.2 (138.4 to 281) | 197.5 (136.7 to 277.9) | 196.1 (135.6 to 276.8) | 194.6 (134.5 to 276.3) | 192.8 (132 to 271.3) | 190.7 (131.7 to 270) | 189.5 (130.5 to 267.9) | 189.8 (130.8 to 268.6) | 192 (133.3 to 271) | 193.8 (133.3 to 275) |
|  |  | Skin and subcutaneous diseases | 494.7 (319.4 to 723) | 498.1 (321.8 to 725.4) | 501.2 (321.9 to 726.6) | 504.4 (328.2 to 728.9) | 507.9 (329.1 to 733.8) | 511.3 (332.4 to 739.3) | 514.8 (333.5 to 746.1) | 518.6 (337.2 to 749.4) | 521.9 (339.4 to 753.4) | 524.8 (343.2 to 754.9) | 527.3 (345.3 to 756.6) | 530 (347 to 765.2) | 533 (348.1 to 769) | 536.3 (350.6 to 777.6) | 539 (352.8 to 784.5) | 541 (353.8 to 789) | 542.1 (355.5 to 793.8) | 542.4 (355 to 790.8) | 542 (355.3 to 789.8) | 541.4 (353.1 to 790.5) | 540.2 (353.4 to 786.1) | 539.2 (352.1 to 786.9) | 538.1 (352.1 to 783.5) | 537.2 (349.9 to 784.6) | 536.2 (348.4 to 785.2) | 535.5 (346.8 to 786) | 535.8 (346.7 to 785.7) | 537.5 (347.6 to 785.2) | 539.5 (349.4 to 790.6) | 542.3 (353.6 to 794.4) | 544.8 (354.2 to 795.8) | 545 (354.1 to 791.6) |
|  |  | Substance use disorders | 20.4 (12.6 to 28.4) | 21 (13.6 to 29.5) | 21.7 (14 to 30.3) | 22.4 (14.1 to 31.8) | 23.5 (15.1 to 33) | 25 (15.8 to 35) | 26.8 (17.1 to 37.5) | 28.6 (18.3 to 39.5) | 30.2 (18.9 to 42.1) | 31.6 (20.1 to 44.2) | 32.9 (21.1 to 46.3) | 34.3 (22.1 to 48.8) | 35.8 (22.9 to 49.6) | 37.4 (23.6 to 52.8) | 38.4 (23.5 to 54.8) | 38.8 (24.3 to 54.7) | 38.4 (24.2 to 54.1) | 37 (23.7 to 51) | 35.4 (22.5 to 48.6) | 33.4 (21.4 to 46.7) | 31.7 (20.1 to 44.2) | 30.2 (19 to 43.2) | 28.3 (18.1 to 39.6) | 26.8 (17.3 to 36.7) | 25.3 (16.3 to 35.5) | 24.2 (15.8 to 33.5) | 23.4 (15.1 to 32.1) | 23 (15.4 to 31.8) | 22.9 (14.9 to 31.9) | 22.9 (15.1 to 31.7) | 23.1 (14.9 to 32) | 23.2 (15 to 31.9) |
|  |  | Transport injuries | 154.1 (111 to 210.3) | 150.8 (108.1 to 206.3) | 147.5 (106.1 to 200.1) | 144.2 (103.7 to 195.9) | 141.2 (101.6 to 191.6) | 138.3 (99.5 to 187.8) | 135.6 (97.7 to 184.5) | 132.7 (95.6 to 180.5) | 129.8 (93 to 176.2) | 126.5 (90.4 to 170.8) | 123.4 (89.1 to 167.3) | 120 (86.2 to 161.6) | 116.5 (83.2 to 157.9) | 112.5 (80.1 to 152.4) | 108.4 (77.7 to 146.5) | 103.9 (73.8 to 140.7) | 99 (70.1 to 134.6) | 93.6 (66.6 to 126.9) | 87.8 (62.5 to 119) | 81.7 (58.2 to 110.9) | 75.7 (54 to 102.7) | 69.4 (49.5 to 94.5) | 62.6 (44.7 to 84.9) | 55.9 (39.8 to 76) | 49.9 (35.6 to 68.1) | 44.8 (32.1 to 61.5) | 40.4 (28.9 to 55.2) | 36.2 (25.8 to 49.5) | 33 (23.6 to 45.1) | 31.6 (22.5 to 43.3) | 31.7 (22.4 to 43.7) | 31.7 (22.7 to 43.8) |
|  |  | Unintentional injuries | 165.3 (121.3 to 221.3) | 148.1 (111 to 196.1) | 147.3 (109.5 to 196.3) | 147.5 (108.9 to 197) | 146.7 (108.4 to 196.4) | 146.2 (107.1 to 196.2) | 145.9 (106.5 to 196.8) | 147.9 (108.1 to 199.8) | 145.5 (105.8 to 195.6) | 143.9 (103.5 to 194) | 142 (101.8 to 192.4) | 140.9 (101.5 to 191) | 139.1 (100.1 to 188.9) | 162.9 (119.5 to 217.9) | 147.5 (108.7 to 196.8) | 144.1 (106.1 to 192.9) | 139.2 (103.1 to 186.9) | 133.5 (98.7 to 180.3) | 127.2 (93.2 to 172.9) | 119.9 (86.7 to 164) | 113 (81.1 to 155.3) | 107.2 (76.7 to 147.7) | 101.6 (72.6 to 140.8) | 95.5 (68.1 to 132.3) | 90.4 (64.5 to 126) | 86.1 (61.3 to 120.3) | 82.4 (58.5 to 116) | 79.8 (56.6 to 111.7) | 76.8 (54.1 to 107.7) | 75.6 (53.4 to 106.3) | 75.5 (53 to 106.7) | 76 (52.8 to 107.4) |
| YLDs (Years Lived with Disability) | Males | Causes | Year |  |  |  |  |  |  |  |  |  |  |  |  |  |  |  |  |  |  |  |  |  |  |  |  |  |  |  |  |  |  |  |
|  |  |  | 1990 | 1991 | 1992 | 1993 | 1994 | 1995 | 1996 | 1997 | 1998 | 1999 | 2000 | 2001 | 2002 | 2003 | 2004 | 2005 | 2006 | 2007 | 2008 | 2009 | 2010 | 2011 | 2012 | 2013 | 2014 | 2015 | 2016 | 2017 | 2018 | 2019 | 2020 | 2021 |
|  |  | Cardiovascular diseases | 79.1 (52.6 to 112.3) | 80.1 (53 to 113.5) | 81.1 (54.5 to 114) | 82.1 (55.2 to 117.1) | 83.1 (55.7 to 118.1) | 83.9 (56.1 to 119.9) | 84.8 (57 to 121.1) | 85.7 (57.5 to 122.6) | 86.4 (58.1 to 123.1) | 87.1 (58.2 to 124.5) | 87.5 (58.5 to 124.8) | 88.3 (59.4 to 126) | 89.8 (61.2 to 128.7) | 91.6 (61.9 to 130.8) | 93.3 (62.8 to 134.1) | 94.5 (63.6 to 135.8) | 95.3 (64.2 to 138.5) | 95.8 (64.5 to 138.5) | 95.9 (63.8 to 138.3) | 95.6 (64.3 to 137.1) | 94.6 (64 to 135.9) | 93.1 (62.7 to 134.5) | 91.6 (61.9 to 132.2) | 90.1 (60.7 to 129.5) | 88.9 (60.1 to 127.5) | 87.8 (59.4 to 125.6) | 87 (59 to 124.5) | 86.1 (57.8 to 123.3) | 85.6 (57.7 to 123.6) | 85.8 (58 to 123.5) | 86.5 (57.2 to 123.6) | 86.9 (57 to 126.9) |
|  |  | Chronic respiratory diseases | 250.9 (153.5 to 394.5) | 244.4 (149.2 to 383.7) | 238.4 (144.2 to 375.5) | 233.1 (141.3 to 368.4) | 228.7 (137.2 to 358.8) | 225 (134.4 to 353.2) | 221.6 (132.2 to 344.3) | 218.4 (131 to 340.7) | 215.6 (129.3 to 336.5) | 213.3 (127.5 to 334.2) | 211.5 (127.4 to 330.1) | 210 (125.7 to 329.4) | 208.3 (124.9 to 324.4) | 206.9 (124.2 to 323.2) | 205.4 (123.5 to 319.7) | 204.3 (123.6 to 319) | 203 (122.7 to 315.8) | 201.4 (120.8 to 314.5) | 199.1 (120.3 to 309.7) | 196.8 (117.8 to 307.5) | 194.9 (115.4 to 306.1) | 193.1 (114.1 to 302.4) | 190.9 (113.5 to 298.9) | 188.3 (113.4 to 296.8) | 185.5 (110.9 to 291.2) | 182.8 (109.9 to 287.7) | 179.4 (107.3 to 285.2) | 175.1 (105.3 to 278.7) | 171.5 (103 to 275) | 170.3 (101.9 to 273.8) | 175.4 (103.6 to 279.3) | 175.4 (104.5 to 280.5) |
|  |  | Diabetes and kidney diseases | 15.2 (10.1 to 21.8) | 15.7 (10.5 to 22.6) | 16.1 (10.7 to 23.3) | 16.5 (10.9 to 24.1) | 16.8 (10.9 to 24.4) | 17.2 (11.2 to 25.2) | 17.6 (11.6 to 25.6) | 18 (11.8 to 26.2) | 18.3 (12.1 to 26.7) | 18.5 (12.1 to 26.8) | 18.7 (12.4 to 27) | 18.7 (12.4 to 27.2) | 18.6 (12.3 to 27) | 18.5 (12.4 to 26.8) | 18.4 (12.1 to 26.6) | 18.4 (12.2 to 26.5) | 18.5 (12.2 to 26.6) | 18.7 (12.5 to 26.8) | 18.9 (12.7 to 27.4) | 18.9 (12.9 to 27.3) | 18.8 (12.5 to 27.1) | 18.5 (12.3 to 26.5) | 18.1 (12.1 to 26.1) | 17.7 (11.9 to 25.3) | 17.4 (11.8 to 24.8) | 17.1 (11.5 to 24.5) | 16.8 (11.4 to 24.1) | 16.4 (11.1 to 23.4) | 16.1 (10.8 to 23) | 16.1 (10.7 to 22.8) | 16.4 (10.9 to 23.7) | 16.8 (11.3 to 24.3) |
|  |  | Digestive diseases | 53.3 (37.2 to 74.2) | 53.6 (37.7 to 74.2) | 53.8 (37.7 to 75.2) | 53.9 (38.2 to 74.5) | 53.9 (38 to 74.3) | 54.1 (38.3 to 74.9) | 54.3 (38.7 to 74.9) | 54.2 (38 to 74.6) | 53.9 (38.3 to 73.9) | 53.6 (38.1 to 74.2) | 53.3 (37.8 to 74) | 53.1 (37.7 to 73.9) | 52.8 (37.4 to 73) | 52.6 (37.1 to 73.2) | 52.4 (37.2 to 72.6) | 52.2 (37 to 72.4) | 52 (37 to 72.4) | 51.8 (36.2 to 72.4) | 51.6 (36.3 to 71.8) | 51.4 (36.1 to 72) | 51.1 (36 to 72.4) | 50.6 (35.7 to 70.7) | 50.1 (35.4 to 70) | 49.8 (34.9 to 68.4) | 49.4 (34.5 to 69) | 49.2 (34.2 to 67.6) | 49.1 (34.2 to 67.6) | 49 (34.2 to 67.5) | 49 (34 to 67) | 48.9 (33.8 to 67) | 48.4 (33.6 to 67.3) | 48.1 (33.8 to 67) |
|  |  | Enteric infections | 204.4 (131.9 to 293.3) | 189.2 (122.3 to 273.6) | 175.5 (112.3 to 255.4) | 162.9 (104.3 to 238.3) | 151.4 (97.5 to 223.3) | 141 (88.9 to 209) | 131.4 (82.4 to 194.8) | 122.4 (76.6 to 182.5) | 114.6 (71.6 to 170.9) | 108 (67.4 to 161.2) | 102.6 (63.1 to 152.7) | 98.4 (60.2 to 147.5) | 94.5 (57.8 to 141.6) | 91.3 (55.8 to 137.1) | 88.4 (54 to 133.2) | 86 (52.6 to 129.4) | 84.3 (51.3 to 127.5) | 82.8 (50.7 to 124.7) | 81.6 (50 to 122.9) | 80.4 (49.3 to 121.5) | 78.8 (48.1 to 119.2) | 77.1 (47.2 to 116.3) | 75.2 (46 to 114) | 73.3 (44.4 to 110.9) | 71.5 (43.5 to 106.8) | 70.1 (42.1 to 104.7) | 69 (41.7 to 104.7) | 68.2 (41.3 to 102.4) | 67.9 (40.8 to 102.7) | 68.3 (40.8 to 102.7) | 69 (41.5 to 103.8) | 69.6 (42.2 to 107.8) |
|  |  | HIV/AIDS and sexually transmitted infections | 1.7 (0.9 to 2.9) | 1.7 (0.9 to 2.9) | 1.8 (0.9 to 3) | 1.8 (0.9 to 3.1) | 1.8 (1 to 3.1) | 1.9 (1 to 3.3) | 2 (1.1 to 3.5) | 2.2 (1.2 to 3.7) | 2.3 (1.2 to 4.1) | 2.4 (1.3 to 4.1) | 2.5 (1.3 to 4.2) | 2.5 (1.4 to 4.4) | 2.6 (1.4 to 4.5) | 2.7 (1.4 to 4.6) | 2.7 (1.4 to 4.8) | 2.8 (1.5 to 4.8) | 2.8 (1.5 to 4.7) | 2.7 (1.4 to 4.6) | 2.7 (1.5 to 4.6) | 2.6 (1.4 to 4.6) | 2.5 (1.4 to 4.4) | 2.5 (1.3 to 4.1) | 2.4 (1.3 to 4.1) | 2.3 (1.3 to 3.9) | 2.3 (1.3 to 3.7) | 2.2 (1.2 to 3.7) | 2.2 (1.2 to 3.7) | 2.1 (1.2 to 3.6) | 2.1 (1.2 to 3.6) | 2.2 (1.2 to 3.6) | 2.2 (1.2 to 3.5) | 2.2 (1.2 to 3.7) |
|  |  | Maternal and neonatal disorders | 162.9 (112.5 to 228) | 164.8 (113.5 to 230.5) | 166.9 (114.3 to 231.8) | 169.3 (115.6 to 237.1) | 171.7 (116.5 to 239.8) | 174.1 (119 to 245.4) | 177 (121.5 to 246.2) | 181.2 (123.8 to 252) | 185.7 (127.4 to 259.7) | 189.9 (128.8 to 262.8) | 193.7 (131.5 to 267.9) | 196.6 (134.5 to 270.6) | 199.6 (136.4 to 276.3) | 202.4 (139.9 to 283.4) | 205.4 (143.2 to 284.8) | 208.4 (144.9 to 288.2) | 211.6 (146 to 296.1) | 215.6 (148.1 to 302.7) | 219.5 (152.1 to 308.4) | 223.6 (153.3 to 313.3) | 227.1 (155.3 to 314.7) | 230.6 (159 to 322.7) | 234.3 (159.5 to 327.9) | 237.6 (161.9 to 331.2) | 240.6 (164 to 335.8) | 242.4 (165.3 to 337.7) | 243.2 (169 to 328.8) | 243.5 (174.2 to 323) | 242.9 (176.5 to 316.8) | 242.1 (173.8 to 310.2) | 240.8 (171.5 to 309.6) | 239.7 (173.2 to 307.5) |
|  |  | Mental disorders | 1324.8 (967.2 to 1726.8) | 1349 (983.9 to 1761.1) | 1372.3 (1004.8 to 1786.6) | 1395.9 (1016.1 to 1830.7) | 1420.4 (1033.6 to 1854.3) | 1448.6 (1053.5 to 1898.5) | 1480.8 (1075.4 to 1942.7) | 1515.9 (1103.4 to 1998.5) | 1548 (1129.1 to 2036.5) | 1572.7 (1143.6 to 2063) | 1588.9 (1152.5 to 2082.3) | 1600.5 (1163.1 to 2105.2) | 1609.4 (1172.4 to 2115.5) | 1615.5 (1169.1 to 2124.9) | 1617.4 (1171.9 to 2122.8) | 1612.8 (1170.4 to 2118.4) | 1598.4 (1163.5 to 2099.6) | 1573.7 (1144.7 to 2064) | 1541.1 (1120.8 to 2015.8) | 1507.3 (1097.9 to 1974.9) | 1474.8 (1071.2 to 1928.4) | 1445.3 (1054.7 to 1892.2) | 1419 (1040.2 to 1857.4) | 1396.6 (1023.1 to 1829.1) | 1376.5 (1008.7 to 1791.9) | 1360.9 (996.4 to 1769.7) | 1349.6 (988.1 to 1759.9) | 1345.3 (982.9 to 1757.8) | 1349.8 (986.7 to 1765.5) | 1361.4 (993.7 to 1781.8) | 1587.8 (1138.6 to 2101.8) | 1597.5 (1149.9 to 2132.2) |
|  |  | Musculoskeletal disorders | 202.9 (132.1 to 285.2) | 209.4 (136.4 to 295.3) | 215.4 (140.9 to 303.7) | 220.9 (143.9 to 309.6) | 227.6 (148 to 319.1) | 235.6 (153.1 to 333.2) | 244.4 (157.5 to 345) | 252.2 (163.2 to 354.9) | 258.2 (166.5 to 365.8) | 263 (169.6 to 374.3) | 266.6 (172.8 to 380.6) | 270.5 (176.2 to 380.7) | 273.9 (180.5 to 386.9) | 276.6 (181 to 390.4) | 278.1 (181.9 to 390.7) | 277.7 (182.3 to 390.2) | 275.7 (180.4 to 388.7) | 272 (178.9 to 381.4) | 267 (173.3 to 376) | 260.9 (169.5 to 364.5) | 253.9 (165.5 to 355.7) | 247.3 (162.6 to 344.6) | 241.2 (157.6 to 338.5) | 235.3 (153.7 to 329.1) | 230.5 (150.6 to 321.3) | 226 (148.1 to 316.9) | 222.6 (144.4 to 309.8) | 220.7 (143.6 to 308.3) | 221 (145.9 to 308.5) | 223.5 (147.1 to 312.4) | 227.9 (148.7 to 319.7) | 229 (150.3 to 322.4) |
|  |  | Neglected tropical diseases and malaria | 88.6 (52.3 to 137.9) | 85.3 (50.8 to 132.8) | 81.9 (49 to 125.7) | 78.8 (47.9 to 120.5) | 75.8 (46.6 to 115.8) | 73.5 (45.9 to 111.7) | 70.7 (44.5 to 106.8) | 67.9 (43.2 to 102.1) | 65.5 (41.8 to 97.2) | 63.5 (40.2 to 93.7) | 62.1 (39.3 to 92.2) | 60.9 (38.5 to 89.6) | 59.8 (37.5 to 88.1) | 58.9 (36.7 to 86.8) | 58.1 (35.7 to 86.3) | 57.6 (35.5 to 84.9) | 57.3 (35 to 84.5) | 57.2 (34.7 to 83.9) | 57.2 (34.8 to 84.2) | 57.3 (34.7 to 84.1) | 57.2 (34.5 to 84.2) | 56.7 (34.2 to 83.9) | 55.9 (33.8 to 83) | 54.7 (33.1 to 80.5) | 53.4 (32 to 79.8) | 52 (31 to 77) | 50.3 (29.9 to 75.2) | 48 (28.2 to 71.9) | 45.8 (27.6 to 69) | 44.4 (26.7 to 68) | 43.7 (26.4 to 67.1) | 42.6 (26.1 to 65) |
|  |  | Neoplasms | 6.6 (4.3 to 9.7) | 6.7 (4.3 to 9.6) | 6.8 (4.5 to 9.7) | 7 (4.6 to 9.9) | 7.1 (4.6 to 9.9) | 7.1 (4.7 to 9.8) | 7 (4.7 to 9.7) | 7.1 (4.8 to 9.8) | 7.1 (4.9 to 9.8) | 7.2 (5 to 10) | 7.4 (5 to 10.1) | 7.6 (5.1 to 10.5) | 7.7 (5.2 to 10.7) | 7.9 (5.4 to 11) | 8.1 (5.5 to 11.2) | 8.2 (5.5 to 11.4) | 8.3 (5.6 to 11.4) | 8.6 (5.7 to 11.8) | 9 (6 to 12.5) | 9.4 (6.2 to 13.2) | 9.7 (6.3 to 13.9) | 10.2 (6.6 to 14.6) | 10.7 (6.9 to 15.4) | 11.3 (7.2 to 16.3) | 11.1 (7 to 16.4) | 10.2 (6.4 to 15.1) | 9.7 (5.9 to 14.6) | 9.6 (5.8 to 14.7) | 9.5 (5.6 to 14.7) | 9.5 (5.6 to 14.9) | 9 (5.5 to 14.2) | 8.3 (5.1 to 12.9) |
|  |  | Neurological disorders | 493 (210.6 to 938.1) | 501.2 (214.1 to 956.4) | 508.6 (210.2 to 974.4) | 516.9 (209.6 to 999.6) | 525.4 (207.8 to 1017.4) | 535.2 (209.1 to 1034.9) | 543.8 (215.2 to 1054.7) | 550.4 (220.6 to 1068.5) | 554.5 (220.9 to 1081.2) | 556 (216.9 to 1095) | 557.2 (219.5 to 1095.2) | 558.2 (222.5 to 1096.2) | 557.7 (221.1 to 1084.5) | 556.7 (223.3 to 1079) | 555.2 (225.2 to 1065.7) | 553.6 (223.1 to 1054.2) | 550 (224.3 to 1044) | 543.3 (217.3 to 1029) | 534.1 (215.4 to 1017.8) | 524.8 (208.5 to 998.9) | 513.9 (203.6 to 978.5) | 502.5 (204.8 to 948) | 490.4 (204 to 921.3) | 477.6 (199.8 to 903.9) | 466.8 (193.7 to 876.2) | 458 (184.8 to 859.3) | 449.3 (181.4 to 852.3) | 442.8 (177.2 to 840.9) | 440.2 (174.9 to 835.4) | 441.6 (169.3 to 844) | 447.3 (165.5 to 854.3) | 469.2 (169.7 to 916.2) |
|  |  | Nutritional deficiencies | 678.4 (426.6 to 1028) | 632 (394.8 to 945.7) | 590.4 (364.9 to 888.2) | 552.5 (338.1 to 837.2) | 518.5 (316 to 789.5) | 487.9 (298.3 to 748.3) | 461.2 (279.8 to 706.5) | 436.2 (264.8 to 669.2) | 414.7 (249.5 to 639.2) | 396.3 (240.7 to 609.9) | 381.6 (230.6 to 585.5) | 369.5 (224.1 to 566.3) | 358.9 (216.3 to 552.8) | 349.4 (211 to 532) | 341 (205.7 to 520.8) | 334.6 (201.1 to 515.6) | 330 (197.5 to 507.6) | 326.8 (192.3 to 503.2) | 325.2 (189.2 to 499.4) | 324.5 (187.2 to 503.9) | 323.8 (185.1 to 506.6) | 322.4 (184 to 506.2) | 321.7 (183.4 to 506.6) | 319.7 (179.9 to 501) | 315.6 (176 to 489.3) | 309.8 (173 to 481.4) | 299.3 (167.5 to 465.8) | 284.2 (159.6 to 452) | 268.2 (151.2 to 430.1) | 255.3 (143.8 to 414.6) | 246.2 (137.9 to 412.7) | 234 (132.3 to 396.1) |
|  |  | Other infectious diseases | 64.9 (42.1 to 95.7) | 61 (39.4 to 90) | 56.8 (36.9 to 84) | 53.7 (34.9 to 78.2) | 51 (33.2 to 74.5) | 48.8 (31.7 to 71.7) | 47 (30.6 to 68.9) | 45.1 (29.6 to 66.3) | 43.4 (28.4 to 64.2) | 42 (27.5 to 61.6) | 40.9 (26.9 to 60.8) | 39.8 (25.8 to 59) | 38.7 (25.1 to 57) | 37.5 (24.1 to 55.3) | 36.4 (23.3 to 53.2) | 35.8 (22.8 to 52.2) | 35.3 (22.6 to 51.3) | 34.9 (22.1 to 51) | 34.8 (21.7 to 51.3) | 34.6 (21.4 to 51.1) | 34.5 (21.3 to 51.1) | 34.5 (21.2 to 51.1) | 34.4 (21.2 to 50.9) | 34.2 (21 to 51.3) | 33.9 (20.7 to 50.5) | 33.6 (20.4 to 49.8) | 33 (19.9 to 49) | 32.1 (19.5 to 48.3) | 31 (18.9 to 46.7) | 30.2 (18.5 to 46) | 29.7 (18.3 to 45.2) | 28.7 (17.7 to 43.7) |
|  |  | Other non-communicable diseases | 442.7 (310.5 to 618.5) | 433.6 (304.8 to 607.9) | 425.2 (299.4 to 596.4) | 417.7 (294.2 to 588.4) | 410.8 (290 to 583.8) | 404.3 (285 to 575.9) | 399.5 (281.9 to 568.9) | 396 (277.5 to 563.8) | 393.7 (276.6 to 561.7) | 392.2 (276.3 to 557.8) | 390.3 (276 to 555.6) | 388.2 (275.8 to 550) | 386.4 (272.5 to 543.1) | 384.9 (272.8 to 541.3) | 383.5 (271.9 to 537.7) | 382.5 (271 to 534.2) | 382.1 (270.1 to 531.6) | 382.3 (272 to 533) | 382.9 (273.5 to 532.6) | 383.9 (274.2 to 533.4) | 384.7 (273.7 to 535.1) | 383.4 (274.3 to 533.4) | 379.3 (271.4 to 531) | 374.2 (268.7 to 525.5) | 368.9 (264 to 520.6) | 365 (259.8 to 517.7) | 361.6 (256.8 to 513.2) | 357.1 (254.1 to 506.7) | 352.1 (250.8 to 501.6) | 347.7 (247.2 to 494) | 342.9 (243.7 to 487.6) | 341.4 (242.1 to 484.3) |
|  |  | Respiratory infections and tuberculosis | 138.4 (87.6 to 208.2) | 137 (86.2 to 207.4) | 135.7 (85 to 203.8) | 134.5 (84.3 to 202) | 133.3 (82.7 to 200.7) | 132.2 (82.1 to 199.6) | 130.9 (81 to 198.4) | 129.7 (80 to 196.1) | 128.6 (79.1 to 195.5) | 127.8 (78.5 to 195.3) | 127.1 (78.6 to 193.5) | 126.7 (77.4 to 193) | 126.2 (78 to 192.8) | 125.9 (77.9 to 190.2) | 125.6 (77.7 to 189.9) | 125.6 (78.3 to 189.8) | 125.5 (77.7 to 188.8) | 125.5 (77.3 to 189.9) | 125.6 (77.8 to 190) | 125.8 (77.5 to 190.1) | 126.4 (77.8 to 191.8) | 127.2 (78 to 191.4) | 127.9 (78.4 to 192.6) | 128.6 (78.9 to 195.1) | 129.1 (79.3 to 195.6) | 129.4 (79.2 to 195.8) | 129.7 (79.4 to 196.4) | 129.8 (79.4 to 198.4) | 129.6 (79.7 to 196.6) | 129.2 (79.5 to 196.5) | 168.2 (103.6 to 260.6) | 232.8 (130.6 to 422) |
|  |  | Self-harm and interpersonal violence | 80.8 (58.6 to 107.1) | 66.2 (48.2 to 87.2) | 57.4 (41.8 to 75.7) | 53.4 (38.7 to 70) | 51.6 (37.2 to 67.8) | 49.5 (36.2 to 65.1) | 48.6 (35.6 to 64) | 47.3 (34.8 to 61.5) | 46 (33.7 to 59.2) | 45.3 (32.9 to 58) | 43.4 (31.6 to 54.7) | 42.3 (30.7 to 53.2) | 40.3 (29.4 to 50.8) | 39.5 (29 to 50) | 38.8 (28.3 to 49) | 37.3 (27.5 to 47.2) | 36.5 (26.8 to 46.2) | 36.6 (27.2 to 46.4) | 36.7 (27.1 to 46.6) | 36.1 (26.8 to 45.8) | 35.2 (26.2 to 44.7) | 33.9 (25.1 to 43.3) | 32.6 (24 to 41.6) | 31.6 (23.1 to 40.2) | 30.4 (22.3 to 38.9) | 29.8 (21.8 to 38.1) | 29.9 (21.7 to 38.3) | 29.5 (21.5 to 38) | 28.2 (20.8 to 36) | 26.4 (19.5 to 33.5) | 28.2 (20.6 to 36.2) | 28.6 (21.1 to 36.5) |
|  |  | Sense organ diseases | 191 (130.5 to 267.3) | 193.3 (131.7 to 271.3) | 195.2 (133.8 to 275.3) | 197.1 (134.4 to 277.9) | 199.3 (136 to 279.9) | 201.7 (137.5 to 284.6) | 204.4 (139.3 to 288.7) | 207.1 (140.9 to 292.9) | 209.5 (143 to 295.9) | 211.3 (144.4 to 296.4) | 212.6 (145.4 to 297.5) | 213.3 (144.6 to 299.2) | 213.5 (146 to 301.6) | 213.5 (145.4 to 303.3) | 213.1 (145.7 to 301.2) | 212.2 (144.3 to 301.7) | 210.7 (142.9 to 300.5) | 208.6 (141.8 to 295.6) | 205.8 (140.9 to 291.3) | 202.7 (138.6 to 286.9) | 199.6 (135.2 to 278.9) | 197 (134.3 to 277) | 194.8 (132.9 to 272.1) | 192.8 (130.9 to 273) | 191.2 (130 to 269.5) | 190 (129.4 to 267.9) | 188.7 (128.9 to 266.1) | 187.9 (128.5 to 265.6) | 187.8 (127.7 to 265.9) | 188.8 (128.8 to 268.3) | 191.1 (128.9 to 269.7) | 191.7 (130 to 272) |
|  |  | Skin and subcutaneous diseases | 429.4 (276.4 to 623.2) | 433.2 (276.4 to 630.1) | 436.8 (281.6 to 636.6) | 440.5 (284.7 to 640.9) | 444.4 (287.5 to 649.3) | 448.7 (290.5 to 657.1) | 453.3 (292.2 to 661.9) | 458.3 (298.5 to 670.5) | 462.3 (301.1 to 676.4) | 466 (303.1 to 677) | 469.4 (305.1 to 685) | 472.5 (307.1 to 690.8) | 475.8 (309.2 to 695.1) | 479 (312.3 to 700.9) | 481.7 (315 to 705) | 483.6 (316.6 to 710.3) | 484.6 (317.5 to 713.6) | 484.8 (315.4 to 713.6) | 484 (314.8 to 710.7) | 482.6 (313.2 to 705.3) | 480.8 (312.2 to 705.3) | 478.7 (311.7 to 699.7) | 476.7 (309.5 to 698) | 474.7 (307.2 to 698.2) | 473 (306.9 to 690.8) | 471.7 (306.4 to 691.5) | 471.6 (305.3 to 692.7) | 472.7 (307 to 693.3) | 475 (307.1 to 698.3) | 477.5 (308.7 to 702.7) | 479.9 (313.1 to 705.4) | 482.4 (313.3 to 704.6) |
|  |  | Substance use disorders | 29 (19.1 to 40) | 30.8 (20.1 to 43) | 32.6 (21.4 to 45.1) | 34.3 (21.8 to 47.1) | 36.7 (22.8 to 50.6) | 40 (24.6 to 55.5) | 44.5 (28.2 to 62.1) | 49.7 (31.4 to 68.8) | 55 (35.6 to 77.1) | 59.6 (38.4 to 83.2) | 63.4 (40.7 to 90.2) | 66.8 (42.7 to 93.9) | 69.9 (44.5 to 97) | 72.5 (46.7 to 101.2) | 74.7 (48.2 to 104.7) | 75.7 (49.2 to 105.6) | 75.8 (49.4 to 106.4) | 74.3 (47.5 to 103) | 72.3 (46.5 to 100.1) | 69.9 (44.9 to 97.7) | 67.3 (43.3 to 93.8) | 64.2 (41.1 to 89.6) | 60.7 (39.1 to 84) | 57.2 (37.4 to 78.9) | 54.1 (35.1 to 74.1) | 51.5 (33.2 to 70.5) | 49.8 (31.8 to 68.5) | 49.3 (31.9 to 67.9) | 49.4 (32 to 67.7) | 49.8 (31.8 to 68.3) | 50.2 (32.8 to 68.9) | 50.6 (33 to 69.3) |
|  |  | Transport injuries | 147.2 (107 to 197.4) | 145.3 (104.9 to 195.2) | 143.4 (104.1 to 192.9) | 141.6 (102.8 to 191) | 140 (101.7 to 189) | 138.9 (101.4 to 188.1) | 138.2 (100.7 to 187.1) | 137.4 (100.5 to 185.6) | 136.5 (99.8 to 184.2) | 135.1 (98.6 to 183) | 133.3 (98 to 180.4) | 131.3 (96.2 to 177.8) | 129.1 (94.5 to 175.6) | 126.3 (91.8 to 171.8) | 123 (89.6 to 167.4) | 119.1 (86.5 to 161.7) | 114.5 (83 to 155.3) | 108.8 (79 to 148.4) | 102.6 (74.5 to 139.3) | 96 (69.5 to 130.7) | 89.3 (64.7 to 122) | 82.1 (59.2 to 112.7) | 74.1 (53.6 to 101.5) | 66.4 (47.7 to 90.5) | 59.2 (42.6 to 81.1) | 53.5 (38.2 to 72.9) | 48.6 (34.7 to 66.3) | 44 (31.5 to 60.3) | 40.6 (29.1 to 55.8) | 39.3 (27.9 to 54.1) | 39.7 (28.3 to 54.4) | 39.8 (28.1 to 54.3) |
|  |  | Unintentional injuries | 194.1 (140.6 to 259.9) | 183.6 (132.8 to 247.8) | 183.7 (132.4 to 248.6) | 184.2 (132.4 to 249.9) | 184.3 (133 to 250.5) | 185.2 (133.6 to 252.1) | 186.4 (133.7 to 254.8) | 188.4 (135.2 to 259.2) | 188.3 (135.5 to 258.8) | 187.9 (134.4 to 258) | 186.9 (133.5 to 256.4) | 185.5 (132.5 to 255.1) | 183.7 (130.8 to 253.2) | 192.4 (138.9 to 263.7) | 183 (131.3 to 251.5) | 179.7 (129.1 to 246.8) | 175.2 (125.2 to 241.5) | 169.7 (121.9 to 233.7) | 163.4 (117 to 225.9) | 155.9 (111.4 to 216.6) | 147.6 (105.2 to 205.1) | 139.6 (99 to 194.6) | 130.7 (93.2 to 182.7) | 121.5 (85.9 to 170.7) | 113.3 (80.4 to 159.1) | 107 (76.3 to 149.9) | 101.8 (72.3 to 142.8) | 97.7 (69.3 to 137.5) | 94.3 (66.6 to 132.7) | 93.2 (65.6 to 130.5) | 94 (66 to 133.1) | 94.6 (65.9 to 134.3) |
| YLLs (Years of Life Lost) | Both Sexes | Causes | Year |  |  |  |  |  |  |  |  |  |  |  |  |  |  |  |  |  |  |  |  |  |  |  |  |  |  |  |  |  |  |  |
|  |  |  | 1990 | 1991 | 1992 | 1993 | 1994 | 1995 | 1996 | 1997 | 1998 | 1999 | 2000 | 2001 | 2002 | 2003 | 2004 | 2005 | 2006 | 2007 | 2008 | 2009 | 2010 | 2011 | 2012 | 2013 | 2014 | 2015 | 2016 | 2017 | 2018 | 2019 | 2020 | 2021 |
|  |  | Cardiovascular diseases | 825.5 (702.2 to 1030.6) | 764.1 (656.4 to 949.1) | 724.1 (633.2 to 904.1) | 686.5 (596.9 to 849.2) | 652.8 (569.9 to 785.1) | 620.4 (548.6 to 733.8) | 590.5 (526.3 to 685.7) | 567.6 (502.5 to 644.7) | 547.1 (491.8 to 614.9) | 530.2 (477.6 to 587.7) | 515 (464.6 to 569.3) | 501.7 (456.5 to 548.5) | 501.9 (456 to 552.6) | 505.5 (461.5 to 560.5) | 511.1 (463.4 to 568.8) | 505.7 (456.4 to 562.8) | 506 (455.2 to 564.7) | 510.4 (461.6 to 569.3) | 522.6 (477.5 to 582) | 529.6 (484.2 to 587.3) | 522.8 (480.4 to 576.1) | 518.3 (477.2 to 567.4) | 519.2 (481.4 to 562) | 515.8 (481.8 to 554.2) | 465.4 (439.5 to 497) | 388.3 (360.7 to 418) | 322.1 (287.4 to 349.3) | 288.4 (251.4 to 315.8) | 260.6 (224.1 to 289.4) | 240.8 (205 to 270.7) | 212.4 (185.1 to 232.5) | 189.1 (164.2 to 208.1) |
|  |  | Chronic respiratory diseases | 151.1 (113.7 to 187.7) | 139.9 (108.3 to 169.8) | 132.8 (104.5 to 160.3) | 126.1 (101 to 150) | 120.3 (97 to 143) | 114.3 (94.3 to 135.1) | 108.4 (90.1 to 128.3) | 104 (88.2 to 121.4) | 100.1 (85.7 to 116.8) | 97.1 (84.7 to 112) | 94.5 (82.8 to 108.9) | 92 (80.9 to 105.5) | 91.2 (80.2 to 104.6) | 91.8 (81.3 to 105.3) | 93.2 (82.7 to 105.5) | 92.8 (82.6 to 105) | 92.6 (83.4 to 104.4) | 92.3 (83 to 103.7) | 94.1 (85 to 105.3) | 94.9 (86 to 106) | 93.5 (84.5 to 104.6) | 92.9 (85 to 104) | 93.4 (85.2 to 103.9) | 93.1 (85.8 to 102.4) | 83.6 (77.8 to 91.1) | 70.6 (64.5 to 80.4) | 59.4 (54.1 to 68.5) | 53.8 (48.6 to 63.9) | 49.3 (43.7 to 59.6) | 45.9 (40.1 to 56.4) | 41.3 (36.6 to 50.1) | 36.5 (31.7 to 44.5) |
|  |  | Diabetes and kidney diseases | 103.3 (85 to 123.7) | 97.7 (82 to 117.2) | 94.6 (78.9 to 115.2) | 92.1 (77.4 to 112.3) | 89.4 (75.2 to 108.4) | 87.3 (73.8 to 106.8) | 85.7 (72.3 to 105.3) | 84 (71.2 to 102.4) | 82.6 (71.5 to 100.4) | 80.7 (70.1 to 97.1) | 80.5 (70.9 to 94.8) | 80.5 (71.1 to 93.4) | 82.1 (73.3 to 94.6) | 84.1 (74.2 to 96.7) | 87.9 (77.7 to 101) | 91.6 (78.6 to 104.7) | 95.1 (80.6 to 108) | 97.7 (80.2 to 110.5) | 101.2 (81.6 to 113.8) | 104.1 (81.9 to 116.4) | 105.5 (81.4 to 117.7) | 107.5 (81.5 to 119.9) | 108.5 (82.9 to 119.8) | 109.3 (83.2 to 120) | 101.2 (77.9 to 110) | 89.9 (69.8 to 101.4) | 78.4 (63 to 87.3) | 72.5 (59.8 to 81.2) | 67.7 (56.8 to 76.2) | 64.4 (54.4 to 72.2) | 56.9 (47.9 to 62.6) | 50.8 (43 to 56.3) |
|  |  | Digestive diseases | 419.7 (298.7 to 499.7) | 383.9 (288.3 to 460.1) | 361.9 (269.1 to 438.4) | 338.6 (253.3 to 409.6) | 323.3 (242.1 to 397.6) | 303.5 (231 to 367.5) | 281 (217.8 to 328.9) | 267.4 (210.2 to 314) | 255 (203.7 to 297.2) | 246.7 (194.7 to 290.8) | 232.3 (191.1 to 266.5) | 217.9 (184.2 to 247.4) | 214.2 (180.2 to 244) | 212.5 (175.3 to 242.4) | 221.7 (179.5 to 253.1) | 218.7 (176.7 to 249.9) | 217.6 (172.9 to 246.2) | 219.6 (170.1 to 248.5) | 224.5 (173.5 to 255.9) | 225.8 (171.6 to 257) | 222.1 (166.5 to 250.4) | 218.1 (164.4 to 244.4) | 215.4 (164.6 to 241.2) | 210 (161.6 to 231.7) | 193.4 (149 to 211.8) | 165.2 (123.7 to 185.6) | 139 (102.7 to 165.4) | 124.7 (89.9 to 153.3) | 112.9 (80.9 to 142.9) | 103.4 (72.7 to 135.4) | 85.5 (63.6 to 109.1) | 71 (54.3 to 91.8) |
|  |  | Enteric infections | 1214.8 (814.9 to 2012.2) | 1034.1 (696.2 to 1641.2) | 894.8 (610.3 to 1426.4) | 793.7 (557.5 to 1204.7) | 717.1 (514.1 to 1050.4) | 664.4 (483.5 to 944.2) | 585.5 (424.8 to 828.2) | 543.6 (396.9 to 756.4) | 502.3 (363.5 to 681.9) | 467.7 (343.1 to 625.5) | 439.6 (330.9 to 576.4) | 381.4 (287.3 to 506) | 353.7 (266.1 to 464) | 329.9 (248.2 to 428.8) | 311.5 (237.2 to 406.4) | 297.8 (227.3 to 391.2) | 285.5 (216.5 to 371.3) | 275.9 (212.1 to 355.6) | 266.8 (207.1 to 339.3) | 255.8 (199.7 to 323.2) | 244.5 (192.8 to 303.8) | 239.3 (189.6 to 298.6) | 229.8 (181.3 to 288.5) | 225.1 (179.3 to 284.2) | 208.6 (165.6 to 264.4) | 193 (153.7 to 243.5) | 164.6 (133.5 to 207.3) | 146.7 (116.7 to 187) | 131.9 (101.2 to 170.5) | 120 (92.3 to 155.9) | 90.3 (67.9 to 116.2) | 67.2 (49.4 to 88.7) |
|  |  | HIV/AIDS and sexually transmitted infections | 64.3 (22.8 to 131.4) | 59.6 (21.4 to 123.9) | 55.7 (20.4 to 115.5) | 53.3 (19.6 to 112.1) | 51.5 (19 to 109.7) | 49.6 (18.5 to 105.6) | 48 (17.8 to 103.1) | 47 (17.7 to 99) | 46.4 (17.2 to 98.9) | 46 (17.6 to 98.2) | 45.9 (17.7 to 97.6) | 45.9 (17.6 to 99.3) | 46.5 (18.4 to 99.7) | 47.1 (19 to 99.8) | 48.1 (19.9 to 99.6) | 49 (20.3 to 100.8) | 51.5 (21.2 to 104.3) | 57.2 (24.7 to 113.4) | 62.2 (27.2 to 122.5) | 67.5 (29.7 to 134.7) | 71.1 (32 to 145.3) | 70.9 (31.1 to 147.3) | 73.9 (33.5 to 150.3) | 78.8 (38.1 to 153.5) | 78.8 (38.9 to 150.4) | 76.8 (38.8 to 145.3) | 69.8 (33.8 to 137.4) | 67 (33 to 132.7) | 63 (30.8 to 125.5) | 58.7 (29.2 to 115.7) | 53.9 (26.9 to 102.8) | 49.6 (24.8 to 94) |
|  |  | Maternal and neonatal disorders | 12130.2 (10371.5 to 14365.9) | 10980.2 (9408.1 to 12919.6) | 10148.8 (8680.4 to 12031.8) | 9406.4 (7956.9 to 11221.9) | 8647.2 (7315.2 to 10292.3) | 7959.3 (6746.9 to 9367.7) | 7413.1 (6298 to 8612) | 6987.8 (5926 to 8097.2) | 6622.5 (5585.5 to 7649.4) | 6297.2 (5372.1 to 7357.7) | 6029.1 (5185.3 to 6927.4) | 5896.1 (5133.6 to 6721.7) | 5784.7 (5077.7 to 6548) | 5639.5 (4964.8 to 6334.5) | 5417.5 (4787.8 to 6073.8) | 5290.9 (4606.1 to 6001.1) | 5336.2 (4616.6 to 6107.8) | 5533.6 (4767.1 to 6327.6) | 5787.6 (4949.3 to 6646.4) | 5947.3 (5080.5 to 6848.7) | 5981.7 (5179.6 to 6846.9) | 5916.3 (5165.5 to 6698) | 5872.4 (5217.8 to 6602.2) | 5772.6 (5217.1 to 6422.2) | 5491.4 (4962 to 6032) | 4862.9 (4451.6 to 5243.5) | 3719.9 (3415.8 to 4020.6) | 3007.5 (2720.5 to 3289.6) | 2498.7 (2220.7 to 2770.5) | 2118.7 (1855.8 to 2389.3) | 1349 (1197.6 to 1520.7) | 862.7 (708.8 to 1042.9) |
|  |  | Mental disorders | 0 (0 to 0) | 0 (0 to 0) | 0 (0 to 0) | 0 (0 to 0) | 0 (0 to 0) | 0 (0 to 0) | 0 (0 to 0) | 0 (0 to 0) | 0 (0 to 0) | 0 (0 to 0) | 0 (0 to 0) | 0 (0 to 0) | 0 (0 to 0) | 0 (0 to 0) | 0 (0 to 0) | 0 (0 to 0) | 0 (0 to 0) | 0 (0 to 0) | 0 (0 to 0) | 0 (0 to 0) | 0 (0 to 0) | 0 (0 to 0) | 0 (0 to 0) | 0 (0 to 0) | 0 (0 to 0) | 0 (0 to 0) | 0 (0 to 0) | 0 (0 to 0) | 0 (0 to 0) | 0 (0 to 0) | 0 (0 to 0) | 0 (0 to 0) |
|  |  | Musculoskeletal disorders | 10.2 (8.4 to 12.8) | 9.9 (8 to 12.3) | 10 (8.2 to 12.2) | 10 (8.2 to 12.2) | 10 (8.3 to 12.4) | 10 (8.4 to 12.4) | 10.1 (8.6 to 12.5) | 10.1 (8.6 to 12.8) | 10.1 (8.6 to 12.8) | 9.9 (8.6 to 12.4) | 9.9 (8.6 to 13) | 9.8 (8.4 to 12.9) | 10 (8.6 to 12.8) | 10.4 (8.9 to 13) | 11 (9.5 to 13.3) | 11.5 (10 to 13.5) | 12.4 (10.6 to 14.1) | 12.8 (10.8 to 14.7) | 13.2 (10.8 to 15) | 13.5 (10.7 to 15.5) | 13.6 (10.3 to 15.4) | 13.9 (10.2 to 15.6) | 14.4 (10.2 to 16.3) | 14.9 (10.1 to 16.9) | 13.9 (9.3 to 15.8) | 12.7 (8.2 to 14.6) | 11.4 (7.4 to 12.9) | 10.8 (7 to 12.3) | 10.1 (6.6 to 11.7) | 9.7 (6.5 to 11.4) | 9.2 (6.2 to 10.7) | 8.7 (5.9 to 10.2) |
|  |  | Neglected tropical diseases and malaria | 109.9 (20.1 to 510) | 140.3 (19.9 to 893.1) | 85.8 (16.7 to 468) | 83 (16.6 to 508.6) | 62.2 (16.2 to 259.8) | 51.2 (14 to 219.5) | 47.8 (13.1 to 217.5) | 32.9 (12.2 to 133) | 27.9 (11.4 to 117.4) | 34.8 (10.8 to 143.2) | 21.5 (10.2 to 96.1) | 19.6 (9.8 to 82.4) | 20 (9.8 to 74.6) | 22.8 (9.7 to 75.8) | 16.3 (9.3 to 55.8) | 17.7 (9.7 to 52.7) | 15.8 (9.9 to 47.7) | 15.9 (10.1 to 44.7) | 15.8 (10.7 to 41.3) | 15.6 (9.9 to 38.5) | 15.8 (8.5 to 37.4) | 16 (8.6 to 36.4) | 16.5 (8.6 to 36.6) | 17.2 (8.4 to 36.7) | 16.8 (8.1 to 34.7) | 15.4 (7.2 to 31.9) | 13.5 (6.2 to 27.9) | 12.3 (5.3 to 25.6) | 11.2 (5.3 to 23.5) | 10.3 (4.6 to 21.5) | 8 (3.2 to 18.7) | 6.3 (2.7 to 17.5) |
|  |  | Neoplasms | 710.6 (554 to 900.6) | 689 (529.3 to 858.7) | 676.2 (532.2 to 824.6) | 664 (522.1 to 802.3) | 652.7 (524.4 to 782.5) | 639.2 (518.3 to 755.5) | 625.1 (512.3 to 729) | 612.5 (504.5 to 705.2) | 600.3 (494.5 to 690.5) | 588.9 (488.4 to 673) | 580.7 (488.8 to 657.9) | 573 (484.7 to 644.5) | 564.2 (478.6 to 633.1) | 558.1 (473.8 to 628.1) | 557.1 (467.6 to 627) | 556.5 (463.7 to 628.8) | 561.2 (463.9 to 631.9) | 568.6 (465.7 to 641) | 577.8 (468.2 to 655.6) | 584.5 (470.8 to 660.5) | 581.3 (459.9 to 658) | 585 (459.6 to 661.3) | 596.6 (467.2 to 674.2) | 607.7 (474.3 to 685.7) | 573.4 (444.7 to 644.7) | 505.5 (381.5 to 581.8) | 459 (337.5 to 541.6) | 437.4 (319.1 to 525.9) | 417.7 (301 to 512.7) | 402.7 (287.4 to 498.8) | 392.9 (279.5 to 478.6) | 372.4 (266.1 to 455) |
|  |  | Neurological disorders | 163.1 (90.7 to 210.4) | 154.3 (86 to 194.7) | 149.8 (84.4 to 187.5) | 145.2 (82.4 to 179) | 140.6 (82.8 to 174) | 135.9 (80.4 to 166.6) | 131.6 (79.2 to 161.5) | 128.1 (77.7 to 155.1) | 124.6 (76.9 to 150.4) | 121.3 (76.2 to 145.7) | 118.5 (75.1 to 141) | 116 (73.9 to 137.8) | 113.3 (73.1 to 135.1) | 111.1 (73.1 to 132.4) | 110.7 (72.4 to 131.5) | 109.5 (71.8 to 128.7) | 109.2 (73 to 128.1) | 109.2 (72.8 to 128.5) | 110.1 (73.5 to 128.3) | 110.9 (74.1 to 129.6) | 110 (74.3 to 128.1) | 109.8 (76.3 to 126.9) | 110.2 (76 to 125.8) | 112.1 (78.5 to 127.9) | 104.6 (75.3 to 117.9) | 102 (76.1 to 113.4) | 90 (69 to 101.6) | 84.9 (66.2 to 97.2) | 79.5 (61.1 to 91.5) | 75.2 (57.7 to 87.4) | 64.3 (49.9 to 73.8) | 56 (44 to 63.4) |
|  |  | Nutritional deficiencies | 260.2 (211.1 to 349) | 221.1 (179.3 to 298.8) | 196 (160.6 to 269) | 175.4 (143.9 to 236.6) | 157 (128 to 207.5) | 139.2 (113.4 to 178) | 121.7 (99.5 to 155) | 110.4 (91.5 to 138.7) | 100.5 (84 to 124) | 93.3 (77.4 to 114.3) | 86.7 (72.2 to 104.9) | 81.6 (67.7 to 98.1) | 75.1 (62 to 90.6) | 70.2 (56.8 to 85.2) | 64.5 (52 to 78.3) | 61.2 (49.6 to 73.6) | 58.8 (48.2 to 69.9) | 59 (48.6 to 70) | 61.2 (50.6 to 72.8) | 61.2 (51.8 to 71.8) | 59.3 (50.5 to 70) | 56 (48.5 to 64.7) | 53.4 (46.4 to 61.3) | 51.6 (45.2 to 58.5) | 46 (40.9 to 52.2) | 37.5 (32.9 to 42.7) | 28.4 (22.9 to 32.4) | 23.3 (18.1 to 27) | 19.8 (15.1 to 23.2) | 17.2 (12.9 to 20.5) | 11.7 (9.1 to 13.7) | 8.5 (6.6 to 10) |
|  |  | Other infectious diseases | 1213.9 (623.1 to 2406.2) | 953.6 (522.7 to 1879.6) | 747.5 (448.9 to 1355.8) | 624.5 (394.3 to 1071.7) | 531.1 (367.2 to 882.2) | 465.8 (328.2 to 751.1) | 422.3 (304.2 to 670.5) | 381 (282.9 to 581.5) | 357.7 (259.9 to 547.3) | 324.7 (251.2 to 462.5) | 310.3 (243.7 to 432.4) | 288.7 (221.3 to 394.6) | 273.2 (214.5 to 369.2) | 263.6 (209.3 to 349.3) | 250.5 (199 to 339.5) | 247.7 (197.4 to 327.4) | 250.1 (197.4 to 331.8) | 256.6 (206.2 to 345.5) | 264.2 (209.5 to 351.2) | 272.8 (211.6 to 350.7) | 272.4 (213 to 378.6) | 272.4 (212 to 382.8) | 273.4 (214.2 to 363.8) | 275.9 (217.7 to 378) | 247.3 (186.7 to 358) | 217.7 (159.1 to 329.4) | 182.6 (129.2 to 276.9) | 162.2 (115.3 to 246.3) | 145.5 (102.9 to 214.8) | 130.1 (93.3 to 194.3) | 100.7 (71.1 to 179.5) | 77.9 (56.1 to 125.2) |
|  |  | Other non-communicable diseases | 8365.6 (4938.7 to 10271.8) | 7588.5 (4648.9 to 9244) | 7057.7 (4486.2 to 8604.4) | 6598.9 (4338.4 to 8101.7) | 6128.4 (4093.2 to 7539.3) | 5652.7 (3839.6 to 7020.2) | 5240.3 (3662.9 to 6444.8) | 4910.3 (3545.8 to 5974.1) | 4631.1 (3447.7 to 5637.8) | 4399.8 (3361.2 to 5323.1) | 4217.9 (3293.6 to 5133.9) | 4112.6 (3245.9 to 5020.8) | 4051.8 (3245.4 to 4918.2) | 3990 (3230.7 to 4803.9) | 3918.1 (3212.3 to 4676.1) | 3838 (3184 to 4526) | 3851.9 (3193.9 to 4512.3) | 3961.1 (3355.6 to 4601.7) | 4140.3 (3545.1 to 4853.5) | 4282 (3673 to 4930.4) | 4329.9 (3744.6 to 4967.6) | 4311.7 (3750.9 to 4909.7) | 4277.4 (3797.4 to 4880.7) | 4185.5 (3769.9 to 4717.3) | 3916.2 (3552.5 to 4430.8) | 3421.9 (3167.5 to 3865.7) | 2721.8 (2519 to 3063.2) | 2318.2 (2102.2 to 2615.3) | 2014.9 (1781.6 to 2305.3) | 1777.2 (1544.7 to 2084.1) | 1162.9 (1020.8 to 1356.4) | 813.8 (686.7 to 981.8) |
|  |  | Respiratory infections and tuberculosis | 3035.5 (2520.7 to 4080.9) | 2610.3 (2130.3 to 3521.7) | 2314.7 (1892.6 to 3157.3) | 2074.8 (1691.1 to 2776.7) | 1869.5 (1517.6 to 2458.5) | 1687 (1376.2 to 2152.2) | 1533.5 (1271.6 to 1898.5) | 1414.2 (1185.6 to 1718.9) | 1310.3 (1094.9 to 1566) | 1219.5 (1026.2 to 1453.3) | 1138.1 (966.7 to 1351.7) | 1079 (925.9 to 1280.6) | 1049 (892.2 to 1245.1) | 1029.2 (875.1 to 1229.5) | 1020.3 (860 to 1210.2) | 1009.4 (851.1 to 1194.9) | 1016.4 (859.7 to 1188) | 1035.1 (876 to 1216.6) | 1059 (905.6 to 1240.7) | 1067.7 (924 to 1242.9) | 1048.5 (912.2 to 1216) | 1016 (892.8 to 1167.3) | 987.6 (873.5 to 1126.8) | 944.2 (843.4 to 1061.4) | 843.5 (762.4 to 938) | 691 (620.1 to 771.1) | 522.5 (437.4 to 590.3) | 431.1 (345.5 to 498.6) | 365.5 (287.5 to 427) | 317.8 (247.6 to 378.5) | 445.9 (371.2 to 522.7) | 356 (308.4 to 403.3) |
|  |  | Self-harm and interpersonal violence | 469.4 (410.3 to 508.8) | 448.9 (375.1 to 486.8) | 413.7 (357.5 to 453.9) | 410.3 (349.6 to 448.3) | 410.3 (357.1 to 444.9) | 392.6 (337.5 to 429.1) | 404.2 (347.3 to 440.3) | 409.3 (353.1 to 443.6) | 417.6 (365 to 451.8) | 436.7 (375.1 to 470.9) | 432.5 (376.9 to 465.2) | 453.8 (392.8 to 488.7) | 438.5 (377.5 to 471.4) | 452.6 (394.2 to 483.5) | 464.7 (409.1 to 494.7) | 445.2 (392.9 to 475.9) | 443.2 (397.4 to 474) | 443.3 (401.2 to 474.5) | 451.7 (412.5 to 483.8) | 442.4 (404.7 to 472.6) | 420.1 (388.1 to 448.2) | 390.5 (359 to 417.8) | 378.8 (351.4 to 404.4) | 379.1 (350.8 to 405.2) | 352.8 (332 to 375.8) | 331.2 (312.3 to 350.9) | 326 (307.3 to 344.9) | 306.7 (289 to 326) | 276.8 (258.8 to 297.1) | 239.4 (221.8 to 260.7) | 273.5 (256.6 to 292.8) | 254.7 (234.6 to 276.5) |
|  |  | Skin and subcutaneous diseases | 10.2 (6.2 to 13.6) | 9.5 (6 to 12.4) | 9.1 (6.1 to 11.8) | 8.7 (6 to 11.2) | 8.4 (5.9 to 10.6) | 8 (5.8 to 10) | 7.6 (5.5 to 9.4) | 7.3 (5.5 to 9) | 7.1 (5.4 to 8.7) | 6.9 (5.5 to 8.3) | 6.7 (5.4 to 8.1) | 6.5 (5.2 to 7.8) | 6.5 (5.2 to 7.9) | 6.5 (5.2 to 7.9) | 6.5 (5.2 to 7.9) | 6.4 (5.2 to 7.7) | 6.5 (5.2 to 7.8) | 6.6 (5.4 to 7.9) | 6.8 (5.5 to 8.1) | 6.9 (5.7 to 8.3) | 6.9 (5.7 to 8.2) | 6.9 (5.9 to 8) | 6.9 (6 to 7.9) | 6.8 (6 to 7.7) | 6.5 (5.8 to 7.3) | 6.2 (5.5 to 7) | 5.3 (4.5 to 6.2) | 4.8 (3.9 to 5.9) | 4.4 (3.5 to 5.5) | 4.1 (3.2 to 5.3) | 3.4 (2.7 to 4.3) | 2.9 (2.4 to 3.7) |
|  |  | Substance use disorders | 27.4 (21.4 to 34.3) | 27.8 (20.3 to 34.3) | 29.1 (23.4 to 36) | 29.6 (23.1 to 36.4) | 31 (25 to 38.3) | 33.1 (27.1 to 40.3) | 35.6 (29.4 to 44.2) | 38.7 (32.1 to 47.6) | 41.3 (34.2 to 50.4) | 43.5 (36.4 to 52.7) | 45.6 (38.2 to 54.7) | 47.2 (39.7 to 55.6) | 48.6 (41.2 to 57.4) | 49.8 (42.2 to 58.5) | 50.5 (43 to 58.8) | 51 (43.4 to 59.9) | 52.6 (45.2 to 61.2) | 53.1 (46.1 to 61.8) | 51.7 (45.1 to 59.4) | 48.4 (42.3 to 55.5) | 44.7 (39.2 to 50.8) | 41.3 (36.4 to 47) | 39 (34.3 to 44.4) | 36 (31.5 to 40.6) | 34 (29.6 to 38.5) | 31 (27.1 to 35.3) | 30 (26.3 to 34.5) | 29.8 (25.7 to 35.1) | 29.1 (24.9 to 34.3) | 29.1 (24.3 to 34.6) | 28.7 (23.8 to 34.3) | 28.9 (23.4 to 35.6) |
|  |  | Transport injuries | 3826.6 (3330.5 to 4468.1) | 3622.4 (3162.2 to 4169.3) | 3484.8 (3085.8 to 3966.8) | 3354.6 (2968.3 to 3804.9) | 3241 (2874.8 to 3650.6) | 3112.2 (2783.8 to 3528.9) | 3000.4 (2696.1 to 3377.1) | 2920.4 (2640.2 to 3225.6) | 2841.8 (2596.4 to 3123.9) | 2786.8 (2555.2 to 3047) | 2731 (2506.1 to 2977.1) | 2691.5 (2475.5 to 2918.5) | 2667.8 (2454 to 2893.8) | 2639.2 (2432.5 to 2852) | 2598.6 (2404.3 to 2819.6) | 2518.4 (2329.5 to 2740.2) | 2437.1 (2255.1 to 2653.7) | 2371.3 (2193.9 to 2582.4) | 2320.3 (2152.3 to 2515.3) | 2253.7 (2098.7 to 2451.9) | 2123.5 (1981.9 to 2312.5) | 2041.3 (1911.1 to 2205.4) | 1984.8 (1865 to 2133.5) | 1925.3 (1826.5 to 2061.7) | 1676.6 (1600.9 to 1784.3) | 1352.4 (1275.8 to 1448.4) | 1156.1 (1075.1 to 1241) | 1062.2 (976.9 to 1151) | 984.1 (890.5 to 1076.8) | 919.6 (827.7 to 1013.8) | 862.3 (781.2 to 941.4) | 790 (708 to 874.9) |
|  |  | Unintentional injuries | 9462.9 (8726.2 to 10288) | 2590.9 (2174.3 to 2999.1) | 2384.1 (2019.6 to 2777.9) | 2387.3 (2034.5 to 2738.4) | 2176 (1841.6 to 2519.9) | 2071.6 (1747.2 to 2385.6) | 1975.7 (1671.2 to 2267.4) | 2297.9 (2015.7 to 2571.7) | 1867.7 (1582.2 to 2105) | 1803.5 (1529.8 to 2017.3) | 1740 (1478.1 to 1949.3) | 1781.1 (1524.8 to 1985.8) | 1680.8 (1441.2 to 1872.5) | 5075.8 (4704.8 to 5480.7) | 1609.9 (1397.5 to 1786) | 1541.9 (1351.6 to 1714.5) | 1394.1 (1221.7 to 1549.4) | 1338.6 (1177.2 to 1475.9) | 1305.3 (1160.9 to 1432.9) | 1250.2 (1114.7 to 1376.8) | 1199.6 (1078.5 to 1319.9) | 1130.5 (1026.8 to 1229.6) | 1138.8 (1043.2 to 1227.3) | 1074.7 (992.7 to 1159.4) | 969.2 (902.1 to 1042.2) | 832.9 (786.9 to 897.1) | 701.5 (654.2 to 762.8) | 729.9 (683.2 to 792.8) | 580.2 (529.5 to 647.2) | 551.8 (499.5 to 615.9) | 492.7 (454.4 to 547.5) | 421.5 (380.5 to 472.6) |
| YLLs (Years of Life Lost) | Females | Causes | Year |  |  |  |  |  |  |  |  |  |  |  |  |  |  |  |  |  |  |  |  |  |  |  |  |  |  |  |  |  |  |  |
|  |  |  | 1990 | 1991 | 1992 | 1993 | 1994 | 1995 | 1996 | 1997 | 1998 | 1999 | 2000 | 2001 | 2002 | 2003 | 2004 | 2005 | 2006 | 2007 | 2008 | 2009 | 2010 | 2011 | 2012 | 2013 | 2014 | 2015 | 2016 | 2017 | 2018 | 2019 | 2020 | 2021 |
|  |  | Cardiovascular diseases | 812.6 (683.2 to 1070.7) | 747.4 (632.4 to 985) | 704.8 (602.1 to 917.7) | 666 (574.2 to 853.8) | 630.3 (546.2 to 780.7) | 597.1 (520.3 to 730.4) | 567.2 (497.6 to 680) | 543.1 (478.3 to 641) | 521.2 (462.1 to 604.9) | 501.6 (447.8 to 570.6) | 486.2 (436.4 to 547.4) | 472.7 (426.6 to 530.4) | 468.9 (422.4 to 526.7) | 466.3 (417.9 to 525.9) | 464.5 (417.1 to 528.7) | 454.2 (405.3 to 512.7) | 449.9 (402 to 504.6) | 447.2 (399.7 to 504) | 455 (407.6 to 511.3) | 461 (416.4 to 517.7) | 456.4 (412.3 to 506.4) | 455.2 (413.5 to 506.3) | 455.6 (419 to 501.6) | 452.7 (417.6 to 496.4) | 407.2 (381.6 to 443.1) | 341.3 (315.6 to 374.3) | 279.6 (249.1 to 307.7) | 248.6 (214.4 to 276.3) | 226.4 (192.8 to 253) | 209.3 (176.8 to 235.7) | 184.2 (157.2 to 205.9) | 162.8 (139.1 to 183) |
|  |  | Chronic respiratory diseases | 124.4 (67.6 to 191.7) | 117.4 (68.5 to 166.5) | 113.5 (68.8 to 161.1) | 109.3 (70.4 to 151) | 105.5 (69.6 to 142.4) | 101.2 (69.8 to 134.7) | 96.9 (68.9 to 124.1) | 93.7 (69.3 to 117.9) | 90.9 (68.9 to 113.2) | 88.8 (69.6 to 108.9) | 87.2 (70.6 to 105.4) | 85.4 (70.5 to 104.3) | 85.6 (71 to 103.3) | 85.9 (72.3 to 101.6) | 86.6 (72.9 to 101.6) | 85.7 (72.4 to 100.4) | 84.7 (73.1 to 98.9) | 83.6 (72.7 to 96.2) | 84.8 (73.5 to 97.9) | 85.6 (74 to 98.9) | 84.9 (74.9 to 97.4) | 84.9 (75.1 to 98) | 85 (75.5 to 96.9) | 84.5 (74.8 to 95.9) | 76.2 (68.1 to 86.8) | 66.5 (57.6 to 83.5) | 56.7 (47.8 to 74.8) | 51.9 (42.8 to 72.6) | 48.5 (39.1 to 70.1) | 45.7 (35.9 to 67.8) | 41.6 (32.5 to 59.9) | 37 (27.5 to 54.2) |
|  |  | Diabetes and kidney diseases | 95.2 (76.8 to 124.6) | 90.2 (72.2 to 115) | 87.6 (70.5 to 112.7) | 85.7 (69.1 to 110.8) | 83.6 (67.4 to 106.6) | 82.2 (67.2 to 107.2) | 81.4 (67.1 to 105.8) | 80.5 (66.9 to 103.7) | 79.7 (66.7 to 102.1) | 78.2 (66.3 to 99.3) | 78.4 (67.3 to 97.7) | 78.4 (66.8 to 95.4) | 79.9 (69 to 96.3) | 81.5 (70.6 to 97.4) | 85.1 (74.2 to 101.3) | 88.3 (77.8 to 103.9) | 91.8 (79.6 to 105.5) | 93.6 (80.8 to 108.1) | 96.1 (83.1 to 110.8) | 98.3 (84.9 to 112.7) | 99.1 (85.8 to 112.7) | 100.5 (85.3 to 112.9) | 101.5 (86.5 to 113.3) | 102.5 (87.6 to 114.4) | 94.9 (81.4 to 104.8) | 84.2 (72.8 to 93.9) | 74.6 (64.6 to 82.5) | 69.7 (59.7 to 77.5) | 65.6 (55.7 to 74.1) | 62.7 (52.5 to 70.9) | 56.3 (48 to 63) | 50.6 (43.4 to 57.2) |
|  |  | Digestive diseases | 390.5 (294.6 to 489.1) | 357.5 (269.9 to 443.9) | 337.9 (251.2 to 432.8) | 316.6 (239 to 398.9) | 303.7 (227.4 to 385) | 286.4 (221.6 to 359) | 265.8 (213.2 to 318.5) | 254 (208.7 to 300.7) | 242.6 (202.3 to 283.3) | 235 (195.2 to 279.1) | 220.8 (186.3 to 256.3) | 206 (177.9 to 239.2) | 202.3 (172.5 to 235.5) | 200.1 (171.9 to 233.3) | 209.2 (180.2 to 243.5) | 205.1 (175.6 to 235.2) | 202.1 (173.2 to 233) | 203.2 (174.4 to 233.1) | 206.7 (179.7 to 236.4) | 207.5 (182 to 237.9) | 204.5 (180.5 to 232.2) | 201.6 (179.3 to 228.1) | 198.5 (176.5 to 224.6) | 192.8 (172.5 to 217.4) | 178.6 (161.7 to 195.9) | 155.6 (136.5 to 173.8) | 133.6 (109.4 to 157.4) | 121.2 (95.9 to 148.6) | 110.7 (85.3 to 139.7) | 102.2 (77.2 to 132.1) | 84.8 (66.3 to 109.1) | 70.1 (54.8 to 89.8) |
|  |  | Enteric infections | 1177 (791.9 to 2010.5) | 993.4 (674.2 to 1685.5) | 849.8 (582.8 to 1417.7) | 750.8 (516.9 to 1207.4) | 682.2 (473.6 to 1064) | 639.1 (460.3 to 957.9) | 571.8 (414.6 to 838.9) | 538.9 (398.7 to 762.7) | 504 (370.8 to 687.6) | 471 (348.2 to 627.8) | 440.5 (334 to 569.8) | 379.8 (292.6 to 491.8) | 350.2 (267.8 to 456.3) | 325.3 (249.4 to 423.9) | 307.9 (237.9 to 396.5) | 296.2 (230.4 to 380.5) | 285.6 (220.3 to 366.2) | 274.3 (211.4 to 349.3) | 260.8 (204.9 to 332.5) | 246.6 (195.5 to 314.7) | 233.4 (187.7 to 296.5) | 228 (182.5 to 291) | 219.9 (176.6 to 280.8) | 216.6 (175.4 to 270.2) | 203.4 (166.8 to 253.5) | 193.1 (155 to 243.9) | 173.1 (131 to 222) | 158 (114.1 to 204.2) | 144 (101.2 to 187.7) | 132.7 (89.2 to 176.2) | 99.1 (67.8 to 129) | 72.2 (48.5 to 98.6) |
|  |  | HIV/AIDS and sexually transmitted infections | 59 (20.7 to 124) | 54.6 (19.4 to 114.2) | 51 (18.2 to 106) | 48.8 (17.6 to 102.6) | 47.1 (17.3 to 100.4) | 45.4 (16.9 to 96.2) | 43.9 (16.2 to 93) | 43 (16.1 to 91) | 42.5 (15.9 to 90.9) | 42.2 (15.9 to 89.8) | 42.2 (16.1 to 87.6) | 42.3 (16.4 to 89.5) | 42 (16 to 88.6) | 43 (17.1 to 89.8) | 44.1 (17.8 to 90.8) | 45 (18.1 to 92.6) | 47.3 (19.2 to 98.5) | 54.5 (24.1 to 107.2) | 59.4 (26.9 to 115.9) | 64.5 (29.9 to 125.7) | 67.9 (31.5 to 134.2) | 64.7 (27.8 to 132.2) | 67.9 (30.4 to 135.1) | 74.6 (36.9 to 142.7) | 76 (39.2 to 139.5) | 75.7 (40.1 to 137.1) | 67.5 (34.1 to 126.8) | 64.8 (33.3 to 124.5) | 60.9 (31.4 to 116.7) | 56.8 (29.3 to 110.9) | 52.2 (27.2 to 97.5) | 48.1 (25 to 89.3) |
|  |  | Maternal and neonatal disorders | 10594.7 (8932.5 to 13176.2) | 9600.5 (8092.2 to 12108.9) | 8880.1 (7470.4 to 11155.3) | 8222.1 (6875.9 to 10320.5) | 7546.4 (6304.6 to 9453.1) | 6939.8 (5810.8 to 8587) | 6464.1 (5381.8 to 7992.7) | 6093.7 (5108.3 to 7447.1) | 5774.7 (4809.7 to 7050.4) | 5491.2 (4662.3 to 6606.4) | 5258.6 (4500 to 6185.9) | 5145.4 (4452.5 to 5944.1) | 5048.2 (4387.2 to 5798.5) | 4917.9 (4321.4 to 5623.5) | 4717.7 (4158.6 to 5362.9) | 4605.2 (4039.3 to 5222.6) | 4642.5 (4022.4 to 5323.3) | 4813 (4155.5 to 5590.6) | 5036.1 (4271.8 to 5879.2) | 5179.8 (4414.2 to 6037.9) | 5217 (4526 to 6082.2) | 5173 (4489.7 to 5955.2) | 5149 (4523.5 to 5879.8) | 5076.3 (4494.9 to 5722.5) | 4839.3 (4319.7 to 5347.8) | 4296.2 (3905.7 to 4714.8) | 3292 (2982.5 to 3588.6) | 2671 (2358 to 2934.2) | 2226.3 (1949.8 to 2469.2) | 1893.5 (1632.2 to 2125.7) | 1216.9 (1052.4 to 1366.5) | 783.8 (633.6 to 949.4) |
|  |  | Mental disorders | 0 (0 to 0) | 0 (0 to 0) | 0 (0 to 0) | 0 (0 to 0) | 0 (0 to 0) | 0 (0 to 0) | 0 (0 to 0) | 0 (0 to 0) | 0 (0 to 0) | 0 (0 to 0) | 0 (0 to 0) | 0 (0 to 0) | 0 (0 to 0) | 0 (0 to 0) | 0 (0 to 0) | 0 (0 to 0) | 0 (0 to 0) | 0 (0 to 0) | 0 (0 to 0) | 0 (0 to 0) | 0 (0 to 0) | 0 (0 to 0) | 0 (0 to 0) | 0 (0 to 0) | 0 (0 to 0.1) | 0 (0 to 0.1) | 0 (0 to 0.1) | 0 (0 to 0.1) | 0 (0 to 0.1) | 0 (0 to 0.1) | 0 (0 to 0.1) | 0 (0 to 0.1) |
|  |  | Musculoskeletal disorders | 13.5 (11 to 17.3) | 13.3 (10.8 to 17.1) | 13.6 (11.1 to 17.1) | 13.7 (11.1 to 17) | 13.9 (11.4 to 17.4) | 14.1 (11.4 to 17.4) | 14.3 (11.9 to 17.6) | 14.5 (12 to 17.7) | 14.5 (11.8 to 17.7) | 14.4 (12 to 17.5) | 14.4 (12 to 18) | 14.3 (11.6 to 17.5) | 14.6 (12 to 17.8) | 15.1 (12.5 to 17.8) | 15.8 (13.2 to 18.4) | 16.5 (13.9 to 19.1) | 18 (14.5 to 20.7) | 18.6 (14.2 to 21.6) | 19 (14 to 22) | 19.4 (13.7 to 22.2) | 19.3 (13.2 to 22) | 19.7 (13 to 22.4) | 20.4 (12.7 to 23.3) | 21.2 (12.5 to 24.1) | 19.8 (11.6 to 22.6) | 18.3 (10.6 to 21.3) | 16.7 (9.6 to 19.2) | 16 (9 to 18.9) | 15.1 (8.8 to 18.1) | 14.6 (8.5 to 17.8) | 14.1 (8.3 to 17.1) | 13.6 (8 to 16.5) |
|  |  | Neglected tropical diseases and malaria | 96.8 (16.9 to 470.1) | 126 (16.8 to 840.6) | 77.5 (14 to 460) | 74.4 (13.8 to 453.6) | 56.2 (13.7 to 245.4) | 45.7 (11.6 to 208.1) | 42.5 (10.8 to 210.7) | 28.2 (9.9 to 103.8) | 23.5 (9.3 to 83.8) | 30.9 (9.3 to 140.3) | 17.2 (8.7 to 62.9) | 15.8 (8.2 to 54) | 16.6 (8.3 to 50.6) | 19.7 (8 to 64.5) | 13.4 (7.6 to 37.7) | 14.9 (8.1 to 38.8) | 13.1 (8.1 to 32.5) | 13.2 (8.4 to 30.5) | 13.2 (8.9 to 29.2) | 13 (8.1 to 27.4) | 13.2 (8 to 26.5) | 13.4 (7.8 to 25.9) | 14 (7.4 to 26.8) | 14.6 (7.7 to 26.8) | 14.3 (6.9 to 25.3) | 13 (6.5 to 22.5) | 11.5 (5.8 to 19.6) | 10.4 (5 to 18.1) | 9.6 (4.7 to 17.1) | 8.8 (4.1 to 15.6) | 7 (3.2 to 13.2) | 5.5 (2.6 to 11.8) |
|  |  | Neoplasms | 682.2 (485.2 to 836) | 659.9 (459.2 to 799.6) | 647.2 (469.7 to 780.3) | 634.4 (459.1 to 748.2) | 622.9 (456.2 to 738.1) | 609.6 (453.6 to 709.1) | 595.2 (449.4 to 685.6) | 582.7 (445.8 to 664) | 569.7 (442.1 to 650.4) | 557.3 (432.2 to 638.3) | 547.5 (430.7 to 613.9) | 537.4 (422.7 to 606.9) | 526.4 (420.4 to 593) | 517 (415.1 to 581.2) | 512.4 (410.7 to 578.2) | 507.8 (404.7 to 573) | 510.8 (402.3 to 574.5) | 515.5 (399 to 580.8) | 522.5 (399.8 to 588.4) | 529.4 (397.2 to 594.1) | 528.4 (391.1 to 595.5) | 533.7 (388.7 to 595.8) | 544.7 (391 to 607.5) | 555.8 (395.6 to 622.2) | 522 (368.7 to 581.7) | 462 (329.3 to 526.3) | 419.4 (293.7 to 492.1) | 400.5 (276 to 479.1) | 383.7 (260.5 to 463.7) | 370.9 (250.8 to 454.4) | 367.7 (250.6 to 444) | 355 (239.6 to 425.7) |
|  |  | Neurological disorders | 158.5 (67.9 to 226.9) | 149.4 (65.6 to 208.3) | 144.5 (65.6 to 198.9) | 139.8 (62.8 to 188.6) | 134.8 (61.8 to 179.9) | 129.9 (61.6 to 171.4) | 125.3 (61.4 to 163.4) | 121.4 (61.5 to 157.9) | 117.8 (59.5 to 151) | 114.4 (58.8 to 146.3) | 111.9 (58 to 141.4) | 109.9 (56.3 to 139.4) | 107.1 (55.6 to 137.2) | 104.8 (55.2 to 133.4) | 104 (56.3 to 131.6) | 102.4 (56.6 to 126.7) | 102.2 (58.3 to 124.9) | 101.6 (58.9 to 122.4) | 102.2 (60 to 124.1) | 102.8 (60 to 124.6) | 101.8 (60 to 123.3) | 101.8 (61.4 to 123.1) | 102.4 (62.6 to 124.4) | 103.9 (64.8 to 125.9) | 96.6 (62.7 to 115) | 89.2 (62.4 to 104.4) | 78.1 (56.5 to 93.3) | 72.4 (51.2 to 87.8) | 67.1 (47.5 to 82.6) | 63.1 (45 to 78.4) | 54.3 (39.1 to 66.8) | 47.1 (34 to 57.5) |
|  |  | Nutritional deficiencies | 284.1 (224 to 386.7) | 242.4 (193 to 332.2) | 214.1 (170.4 to 290.7) | 190.4 (151.7 to 257.1) | 169 (135.9 to 227.3) | 148.7 (118.6 to 194.9) | 129.9 (104.2 to 168.5) | 117 (94.9 to 150.9) | 106 (85.6 to 136.7) | 97.8 (78.6 to 127.8) | 90.5 (72.9 to 116.2) | 85 (67.7 to 107.3) | 78.4 (61.9 to 98.2) | 72.7 (56.5 to 91) | 65.7 (50.9 to 82.2) | 61.2 (48 to 77.3) | 57.8 (45.5 to 73.3) | 57.6 (45.6 to 72.5) | 59 (47.6 to 73.2) | 59.5 (48.9 to 72) | 57.3 (47.9 to 70) | 54.6 (46.6 to 64.9) | 52.2 (44.7 to 61.5) | 50 (43.1 to 57.5) | 44.2 (38.5 to 50.8) | 36.2 (31.6 to 41.6) | 27.2 (22.1 to 31.3) | 22.2 (17.3 to 25.8) | 18.8 (14.4 to 22.2) | 16.3 (12.3 to 19.4) | 11.3 (8.8 to 13.4) | 8.2 (6.4 to 9.7) |
|  |  | Other infectious diseases | 1261 (607.9 to 2546) | 985.2 (508.5 to 2053.5) | 764.3 (434 to 1432.1) | 632.8 (383.1 to 1126.5) | 531.9 (348.5 to 935.8) | 461.2 (319 to 768.3) | 416.3 (291.1 to 668.3) | 372.2 (267.1 to 596.8) | 348.4 (245.4 to 552.7) | 313.6 (231.4 to 464.1) | 299.9 (222.2 to 430.2) | 278.7 (206 to 389.6) | 262 (198.4 to 370) | 251.3 (192.9 to 342.4) | 236.2 (178.6 to 332.9) | 231.4 (175.8 to 322.5) | 232.9 (174 to 324.2) | 237.6 (182.6 to 332.3) | 244.3 (186 to 336.4) | 253.5 (188.7 to 341.6) | 254.2 (193.7 to 375.3) | 256.1 (192.3 to 371.6) | 257.9 (196.1 to 359) | 260.8 (197.9 to 370.6) | 238.2 (174.5 to 353.9) | 211.7 (151.5 to 334.4) | 179.1 (124.6 to 280.7) | 159.8 (108.7 to 247.6) | 144 (98.6 to 221.3) | 128.9 (87.9 to 198.2) | 100.2 (68.1 to 180.6) | 76.7 (51.6 to 125.7) |
|  |  | Other non-communicable diseases | 7635.2 (3280.7 to 9553.5) | 6922.2 (3014.1 to 8664.1) | 6434.3 (2938 to 8110.5) | 6006.8 (2813.6 to 7601.5) | 5570.1 (2722.5 to 6925.7) | 5130.9 (2625.5 to 6366.4) | 4754.4 (2434.3 to 5900.9) | 4459.7 (2386.6 to 5530.9) | 4212.3 (2312.8 to 5255.9) | 4009.4 (2264.5 to 4993.9) | 3848.1 (2282.3 to 4876.6) | 3753 (2292.7 to 4752.1) | 3696.4 (2357.4 to 4607.3) | 3635.4 (2409.8 to 4512.7) | 3564.6 (2426.3 to 4383.8) | 3486.8 (2418 to 4260.6) | 3496.5 (2451.7 to 4263.1) | 3597 (2606.3 to 4352.3) | 3762.1 (2780.7 to 4529.4) | 3894.2 (2909.8 to 4709.8) | 3939.4 (2972.4 to 4698.3) | 3922.4 (3039.1 to 4631) | 3890 (3096.4 to 4570.5) | 3802.2 (3131.1 to 4441.4) | 3548.9 (3062 to 4177.4) | 3087.3 (2752.9 to 3642.4) | 2447.5 (2187.7 to 2868.8) | 2079.9 (1853.2 to 2454.9) | 1804.2 (1576.8 to 2188.7) | 1588.7 (1370 to 1970.6) | 1060.6 (912.4 to 1324.7) | 744 (608.2 to 964.7) |
|  |  | Respiratory infections and tuberculosis | 2992.2 (2379.7 to 4200.2) | 2567.8 (2016 to 3666.6) | 2274.3 (1796.2 to 3314.1) | 2032.5 (1618 to 2891.7) | 1823.4 (1440.8 to 2516.4) | 1636.9 (1302.8 to 2223.2) | 1480.3 (1208 to 1938.4) | 1359.4 (1119.8 to 1751.5) | 1254.7 (1033.9 to 1580.5) | 1164.7 (967.6 to 1471.3) | 1084.8 (907.8 to 1350.5) | 1026.1 (857.4 to 1277.7) | 994.1 (834.1 to 1242.9) | 969.9 (808.4 to 1207.6) | 953.7 (790 to 1193.5) | 936.9 (769.8 to 1147.5) | 939.9 (768.6 to 1148.4) | 953.2 (786.5 to 1159.8) | 973 (810.6 to 1175.8) | 981 (825.7 to 1180.1) | 964.2 (822.2 to 1138.8) | 935.7 (804.4 to 1089.8) | 909.3 (795.1 to 1044.4) | 871 (776.4 to 999.3) | 774 (702.3 to 879.2) | 631.5 (564.7 to 713.2) | 476 (393.7 to 546.6) | 392.8 (310.3 to 458.3) | 333.3 (260.4 to 396.5) | 290.5 (224.5 to 349.6) | 430.8 (362.3 to 496.2) | 317.9 (275.6 to 371) |
|  |  | Self-harm and interpersonal violence | 304.4 (224.8 to 349.9) | 314.4 (230.5 to 356.8) | 302 (228.1 to 346.6) | 302.2 (221.7 to 344.9) | 303.4 (230.2 to 345.4) | 300 (222.8 to 346.9) | 310.3 (229.6 to 355) | 318.7 (239.4 to 360.7) | 324 (246.2 to 368.8) | 338.8 (255 to 380.2) | 333.2 (257 to 371.6) | 342.5 (262.7 to 381.4) | 330.5 (249.7 to 367.1) | 329.2 (247.9 to 362.7) | 329.5 (256.3 to 363) | 316.8 (246.5 to 345.3) | 308.1 (249.3 to 337.5) | 314.5 (259.5 to 341.7) | 313.4 (261.4 to 341.7) | 302.6 (256.7 to 331.7) | 290.5 (248.4 to 319.6) | 278.9 (241.8 to 307.9) | 275.1 (241.8 to 303.4) | 276.2 (245.8 to 304.9) | 261.5 (238.1 to 284.2) | 240.2 (220.4 to 259.4) | 229.2 (209.9 to 246.6) | 215 (198.1 to 231.7) | 194.5 (178.2 to 210.8) | 165 (148.4 to 181.7) | 185.2 (168.7 to 203.3) | 178.5 (161.3 to 196.6) |
|  |  | Skin and subcutaneous diseases | 7.2 (4 to 10.4) | 6.7 (4 to 9.6) | 6.4 (4.1 to 9.1) | 6.1 (4.1 to 8.6) | 5.8 (4 to 7.9) | 5.5 (3.9 to 7.6) | 5.3 (3.8 to 7) | 5.1 (3.8 to 6.6) | 4.9 (3.8 to 6.3) | 4.8 (3.8 to 6.1) | 4.7 (3.6 to 5.9) | 4.6 (3.5 to 5.7) | 4.6 (3.5 to 5.7) | 4.5 (3.4 to 5.6) | 4.5 (3.4 to 5.6) | 4.5 (3.4 to 5.5) | 4.5 (3.5 to 5.5) | 4.5 (3.5 to 5.5) | 4.6 (3.7 to 5.6) | 4.7 (3.8 to 5.7) | 4.7 (3.9 to 5.6) | 4.6 (3.9 to 5.5) | 4.6 (4 to 5.4) | 4.5 (4 to 5.2) | 4.3 (3.9 to 4.9) | 4 (3.6 to 4.7) | 3.4 (2.9 to 4.3) | 3 (2.5 to 4) | 2.8 (2.3 to 3.7) | 2.6 (2.1 to 3.5) | 2.1 (1.7 to 2.9) | 1.8 (1.5 to 2.4) |
|  |  | Substance use disorders | 17.7 (13.4 to 22.3) | 17.9 (12.1 to 22.5) | 19 (15.3 to 24.2) | 19.5 (14.9 to 24.7) | 20.7 (16.4 to 26.5) | 22.2 (17.7 to 28.9) | 23.9 (19.1 to 30.5) | 25.9 (20.8 to 33.5) | 27.5 (22 to 35) | 28.8 (23 to 37.6) | 30.1 (23.9 to 38.7) | 30.9 (25 to 39) | 31.7 (25.9 to 39.8) | 32.2 (26.3 to 39.9) | 32 (26.1 to 39.8) | 32 (26.2 to 39.2) | 34.6 (27.6 to 42.8) | 36.5 (30.1 to 44.6) | 35.9 (29.7 to 43.2) | 33.7 (28.1 to 40.4) | 30.5 (25.7 to 36.5) | 27.6 (23 to 33.3) | 26.4 (21.9 to 31.6) | 24.9 (20.3 to 30) | 23 (19.1 to 27.6) | 19.8 (16.4 to 23.9) | 18.8 (15.4 to 23.3) | 18.8 (15.3 to 23.6) | 17.5 (14.3 to 22) | 17.2 (14 to 21.8) | 17.8 (14.2 to 22.6) | 18 (14.2 to 23.4) |
|  |  | Transport injuries | 2709.4 (2189.4 to 3203.1) | 2525.9 (2092.5 to 2960.8) | 2400.4 (2016.6 to 2829.3) | 2287.3 (1945.9 to 2691.3) | 2185.6 (1875.9 to 2523) | 2068.3 (1795.1 to 2396.3) | 1958.5 (1723 to 2278.6) | 1873.3 (1665.9 to 2133.9) | 1794.3 (1602.2 to 2041.4) | 1738.6 (1558 to 1965.3) | 1687.6 (1507.5 to 1887.5) | 1649.2 (1474.1 to 1845.4) | 1615.4 (1446.3 to 1808.1) | 1580 (1415.5 to 1768.4) | 1547.3 (1383.4 to 1740.3) | 1497.5 (1337.6 to 1687) | 1435.9 (1278.5 to 1613.5) | 1408.5 (1255.2 to 1577.1) | 1393.9 (1245.7 to 1561.4) | 1372.1 (1242.3 to 1528.2) | 1311 (1196.8 to 1439.7) | 1268.8 (1163.6 to 1392.6) | 1240 (1144.6 to 1360.8) | 1208.3 (1103.2 to 1315.5) | 1047.3 (978 to 1152) | 839.9 (781.8 to 923.7) | 689.2 (635.4 to 757.1) | 619 (561.1 to 686.6) | 578.1 (511.3 to 656.3) | 535.8 (471.9 to 615.5) | 484.2 (421.2 to 546.8) | 425.9 (367.6 to 478.9) |
|  |  | Unintentional injuries | 10135.2 (9328.5 to 11076) | 2039.4 (1624 to 2518.3) | 1842.2 (1458.5 to 2328.6) | 1886.2 (1509 to 2334.3) | 1663.6 (1316.3 to 2047.6) | 1570.8 (1267.2 to 1929) | 1487.2 (1203.3 to 1778.6) | 1910 (1647.2 to 2190.4) | 1400.6 (1131.3 to 1652.1) | 1339.3 (1072.2 to 1567.6) | 1282 (1026.9 to 1488.4) | 1351 (1092.4 to 1534.3) | 1249 (1006 to 1420) | 5495.1 (5064.8 to 5978.5) | 1208.7 (1007.4 to 1377.1) | 1149.6 (971.1 to 1303.3) | 995.3 (832.8 to 1125.6) | 949.5 (801.9 to 1071.5) | 931.2 (797.6 to 1043) | 896 (773.5 to 993.2) | 871 (756.5 to 965.6) | 826.9 (725.7 to 907.1) | 853.4 (760.4 to 934.9) | 794.4 (710.5 to 869.7) | 709.5 (649.5 to 767.1) | 604.2 (558.9 to 647.9) | 498.8 (456.7 to 546.9) | 557.1 (514.5 to 607.7) | 405.3 (361.6 to 455.9) | 389.4 (347.5 to 442.7) | 334.4 (303.8 to 374.4) | 276.1 (249.2 to 317.1) |
| YLLs (Years of Life Lost) | Males | Causes | Year |  |  |  |  |  |  |  |  |  |  |  |  |  |  |  |  |  |  |  |  |  |  |  |  |  |  |  |  |  |  |  |
|  |  |  | 1990 | 1991 | 1992 | 1993 | 1994 | 1995 | 1996 | 1997 | 1998 | 1999 | 2000 | 2001 | 2002 | 2003 | 2004 | 2005 | 2006 | 2007 | 2008 | 2009 | 2010 | 2011 | 2012 | 2013 | 2014 | 2015 | 2016 | 2017 | 2018 | 2019 | 2020 | 2021 |
|  |  | Cardiovascular diseases | 837.8 (711 to 1007.3) | 780.1 (661.7 to 940.8) | 742.7 (637.2 to 899) | 706.2 (606.8 to 848.7) | 674.7 (584.2 to 790.5) | 643 (560.7 to 743.3) | 613.1 (537.9 to 698.4) | 591.3 (519.3 to 666.4) | 572.2 (509 to 637.1) | 557.7 (498.5 to 619.7) | 542.6 (486.9 to 601.3) | 529.5 (478 to 587.1) | 533.7 (481.7 to 590.2) | 543.2 (490.2 to 600.1) | 555.7 (499.7 to 623.2) | 555 (496.6 to 623.7) | 559.9 (507.2 to 631.7) | 571 (517.8 to 641) | 587.6 (539.4 to 657.2) | 595.4 (545.8 to 663.6) | 586.5 (538.1 to 648.8) | 578.9 (531.5 to 639.6) | 580.2 (536.7 to 631.7) | 576.2 (535 to 625.7) | 521 (487.6 to 565) | 433.1 (400.3 to 472.5) | 362.5 (321.9 to 398.3) | 326.3 (284.2 to 359.9) | 293.2 (250.8 to 327.8) | 270.8 (230.3 to 306.2) | 239.1 (209 to 263) | 214 (186.1 to 237.6) |
|  |  | Chronic respiratory diseases | 176.7 (147.4 to 213.7) | 161.7 (135.5 to 192.7) | 151.4 (128.5 to 178.4) | 142.4 (121.4 to 167.9) | 134.7 (113.9 to 158.5) | 127 (108.9 to 149.7) | 119.6 (103.4 to 141.6) | 113.9 (99.4 to 134.7) | 109 (95 to 127.8) | 105.1 (92.4 to 122.2) | 101.5 (90.4 to 117.7) | 98.3 (87.9 to 113.2) | 96.7 (86.6 to 109.7) | 97.5 (87.4 to 110.5) | 99.6 (89.9 to 112.1) | 99.6 (89.8 to 111.5) | 100 (90 to 111.5) | 100.7 (90.5 to 112.6) | 103 (92.7 to 115.1) | 103.7 (93.2 to 116.7) | 101.7 (91.8 to 114.4) | 100.4 (91.3 to 111.6) | 101.5 (92.3 to 112.3) | 101.3 (92.1 to 111.5) | 90.6 (83.3 to 99.4) | 74.4 (67.5 to 85.2) | 62 (54.8 to 70.6) | 55.6 (48.6 to 64.1) | 50 (42.9 to 58) | 46.1 (39.4 to 53.7) | 41 (35.9 to 47) | 36 (31.4 to 41.5) |
|  |  | Diabetes and kidney diseases | 111 (84.7 to 137.5) | 105 (81.9 to 128.1) | 101.3 (78.7 to 124.2) | 98.4 (76.6 to 122.5) | 95.1 (74.1 to 116.9) | 92.2 (73.4 to 113) | 89.9 (71.3 to 111.6) | 87.5 (69.8 to 108.8) | 85.4 (68.5 to 105.4) | 83.2 (67.8 to 103.7) | 82.6 (67.4 to 100) | 82.4 (67.4 to 96.7) | 84.3 (68.5 to 98.1) | 86.5 (68.9 to 100.1) | 90.6 (70.3 to 103.2) | 94.8 (71.2 to 109) | 98.3 (71.9 to 112.3) | 101.6 (73.4 to 115.8) | 106 (74.4 to 120.8) | 109.7 (75.5 to 124.3) | 111.7 (74.8 to 128) | 114.2 (74.1 to 130.7) | 115.3 (74.8 to 130.7) | 115.8 (75.2 to 130.2) | 107.1 (70.1 to 119.2) | 95.3 (64.1 to 113.1) | 82 (57.7 to 96.7) | 75.2 (55.3 to 90.3) | 69.7 (51.7 to 83.9) | 66 (49.7 to 79.7) | 57.5 (44.5 to 68.4) | 51 (39.7 to 60.7) |
|  |  | Digestive diseases | 447.8 (273.8 to 547.6) | 409.3 (251 to 498.3) | 385.1 (238.6 to 469.6) | 359.8 (230.7 to 433.9) | 342.3 (226.5 to 417.6) | 320.1 (215.2 to 390.2) | 295.6 (204 to 354.7) | 280.4 (195.6 to 336.9) | 267 (187.7 to 319.5) | 258 (181.5 to 309) | 243.4 (175.2 to 286.1) | 229.3 (165.8 to 266.9) | 225.6 (163.1 to 262.7) | 224.4 (160.5 to 260.7) | 233.7 (158.9 to 272.1) | 231.8 (156.7 to 270.9) | 232.4 (154.3 to 268.6) | 235.4 (150.9 to 272.6) | 241.6 (152.6 to 280.2) | 243.4 (150 to 280.7) | 238.9 (144.9 to 274.9) | 234 (142.8 to 268.3) | 231.5 (140.9 to 263) | 226.4 (140.6 to 256.2) | 207.5 (128.2 to 234.4) | 174.3 (106.1 to 203.6) | 144.1 (89.6 to 179.4) | 128 (81.1 to 167.4) | 115 (72.1 to 154.3) | 104.6 (66.5 to 143.8) | 86 (58.6 to 116.6) | 72 (51.4 to 99) |
|  |  | Enteric infections | 1251.2 (704.4 to 2186.2) | 1073.3 (616 to 1838.2) | 938.2 (557.7 to 1541.3) | 835.2 (506.9 to 1331.6) | 751 (459.7 to 1150.9) | 689 (425.7 to 1051.7) | 598.8 (367.6 to 912) | 548.1 (341.8 to 820) | 500.7 (308.2 to 747.6) | 464.6 (291.9 to 673.3) | 438.8 (286.7 to 621.4) | 382.9 (249.2 to 543.8) | 357.2 (234.9 to 509.8) | 334.3 (223.2 to 469.9) | 315 (211.6 to 448.1) | 299.3 (198.7 to 422) | 285.5 (190.1 to 398.6) | 277.4 (186.1 to 383) | 272.5 (187.6 to 371.3) | 264.8 (185.4 to 360.7) | 255.1 (182.3 to 344.9) | 250.1 (179.9 to 336.5) | 239.4 (172.5 to 322.9) | 233.3 (169.3 to 312.5) | 213.6 (153.6 to 288.5) | 192.9 (140.1 to 262) | 156.5 (112.3 to 214.2) | 135.9 (97.8 to 187.2) | 120.3 (84 to 167.2) | 108 (73.7 to 152.9) | 81.9 (56.4 to 117) | 62.6 (42.9 to 90.3) |
|  |  | HIV/AIDS and sexually transmitted infections | 69.4 (24.5 to 144.5) | 64.4 (23.1 to 133.4) | 60.2 (21.9 to 123.6) | 57.7 (21.4 to 119) | 55.8 (20.8 to 118.3) | 53.8 (20.2 to 114.1) | 52 (19.2 to 110.7) | 50.8 (19 to 108.5) | 50.1 (18.8 to 107.8) | 49.6 (18.9 to 108.3) | 49.6 (18.9 to 105.6) | 49.4 (18.6 to 105.6) | 50.8 (20.5 to 107.5) | 51 (20.5 to 107.1) | 51.9 (21.4 to 107.7) | 52.8 (22 to 109.8) | 55.5 (23 to 114.1) | 59.9 (25 to 122.8) | 64.9 (27.5 to 133.1) | 70.3 (30.1 to 146.4) | 74.3 (32.1 to 152.9) | 76.9 (34.2 to 161.4) | 79.8 (36.2 to 163.2) | 82.8 (39 to 167.1) | 81.5 (38.8 to 159) | 77.9 (37 to 150.7) | 71.9 (33.1 to 145.4) | 69 (32.8 to 140.3) | 64.9 (30.8 to 131) | 60.5 (29.1 to 122.4) | 55.5 (26.8 to 108.4) | 51.1 (24.5 to 99.4) |
|  |  | Maternal and neonatal disorders | 13607.3 (11441.8 to 16423.2) | 12307.6 (10327.6 to 14856.4) | 11370.3 (9637.1 to 13601.7) | 10550.1 (8868.6 to 12624.2) | 9713.8 (8144.6 to 11607.2) | 8948.1 (7483.9 to 10608.4) | 8332.6 (6998.9 to 9834.2) | 7852.3 (6585.4 to 9204.3) | 7440.5 (6210.1 to 8684.6) | 7073.4 (5960.5 to 8305.1) | 6769.7 (5707.7 to 7886.6) | 6616.6 (5652.6 to 7703.3) | 6491.1 (5555.4 to 7506.3) | 6331.7 (5423.4 to 7292.7) | 6088.9 (5187.6 to 7023.4) | 5948.7 (5049 to 6896.9) | 6001.7 (5095.3 to 6915.4) | 6225.1 (5272.1 to 7255.3) | 6509 (5492.5 to 7589) | 6684.2 (5625.5 to 7817.1) | 6715.6 (5807.3 to 7766.4) | 6629.4 (5758.6 to 7620.2) | 6565.5 (5804.3 to 7385.4) | 6438.8 (5759.2 to 7170.4) | 6114.4 (5481.4 to 6683) | 5403.2 (4918.1 to 5851.9) | 4127.3 (3756.8 to 4502.3) | 3327.6 (2937.6 to 3663.1) | 2757.4 (2395.8 to 3102.4) | 2332.5 (2000.6 to 2677.5) | 1474.3 (1272.3 to 1668.7) | 937.5 (754.3 to 1132.2) |
|  |  | Mental disorders | 0 (0 to 0) | 0 (0 to 0) | 0 (0 to 0) | 0 (0 to 0) | 0 (0 to 0) | 0 (0 to 0) | 0 (0 to 0) | 0 (0 to 0) | 0 (0 to 0) | 0 (0 to 0) | 0 (0 to 0) | 0 (0 to 0) | 0 (0 to 0) | 0 (0 to 0) | 0 (0 to 0) | 0 (0 to 0) | 0 (0 to 0) | 0 (0 to 0) | 0 (0 to 0) | 0 (0 to 0) | 0 (0 to 0) | 0 (0 to 0) | 0 (0 to 0) | 0 (0 to 0) | 0 (0 to 0) | 0 (0 to 0) | 0 (0 to 0) | 0 (0 to 0) | 0 (0 to 0) | 0 (0 to 0) | 0 (0 to 0) | 0 (0 to 0) |
|  |  | Musculoskeletal disorders | 7 (4.4 to 11.3) | 6.7 (4.1 to 10.7) | 6.5 (4.1 to 10.5) | 6.3 (4.1 to 10.2) | 6.2 (4.1 to 10) | 6.1 (4 to 10) | 6 (4 to 10) | 5.9 (4 to 9.7) | 5.8 (3.9 to 9.7) | 5.6 (3.8 to 9.5) | 5.6 (3.7 to 9.6) | 5.5 (3.7 to 9.5) | 5.5 (3.7 to 9.6) | 5.9 (3.9 to 9.6) | 6.5 (4 to 9.9) | 6.8 (4 to 9.9) | 7 (4 to 9.9) | 7.2 (4 to 9.8) | 7.6 (3.9 to 9.9) | 7.9 (3.9 to 10) | 8.1 (3.8 to 10.1) | 8.4 (3.7 to 10.4) | 8.7 (3.7 to 10.5) | 8.9 (3.6 to 10.7) | 8.3 (3.3 to 9.9) | 7.4 (3 to 9.3) | 6.3 (2.8 to 7.8) | 5.8 (2.6 to 7.3) | 5.3 (2.4 to 6.7) | 5 (2.3 to 6.5) | 4.5 (2.1 to 5.7) | 4.1 (2 to 5.2) |
|  |  | Neglected tropical diseases and malaria | 122.4 (21.3 to 550.7) | 154 (19.6 to 932.7) | 93.7 (17.5 to 489.9) | 91.4 (17.2 to 541.7) | 68 (16.8 to 274.1) | 56.6 (14.5 to 242.1) | 52.9 (13.4 to 231.7) | 37.5 (12.5 to 168.1) | 32.1 (11.6 to 142) | 38.6 (11.4 to 162) | 25.6 (11.1 to 128.1) | 23.4 (10 to 110.4) | 23.2 (10.4 to 97.6) | 25.7 (10.6 to 93.6) | 19.2 (10 to 73.6) | 20.3 (10.8 to 68.1) | 18.4 (10.7 to 62.5) | 18.5 (11 to 58.2) | 18.4 (11.5 to 53.8) | 18.1 (10.9 to 49.6) | 18.3 (9.2 to 47.5) | 18.5 (9 to 45.5) | 19 (9.1 to 45.2) | 19.6 (8.9 to 45.6) | 19.2 (8.3 to 43) | 17.6 (7.3 to 39.6) | 15.4 (6.4 to 35.2) | 14 (5.8 to 33) | 12.8 (5.3 to 30.2) | 11.7 (4.5 to 27.4) | 8.9 (3.1 to 24.8) | 6.9 (2.4 to 23) |
|  |  | Neoplasms | 738 (463.2 to 979.8) | 717.1 (472 to 940.7) | 704.1 (467.1 to 907.1) | 692.5 (464.1 to 877.8) | 681.5 (461.2 to 851.4) | 667.9 (462.4 to 821.4) | 654.1 (462.7 to 794.9) | 641.4 (456.5 to 775.6) | 629.7 (461 to 753.7) | 619.4 (454.7 to 739.4) | 612.6 (454.3 to 720.2) | 607.1 (454.6 to 709.1) | 600.5 (452.8 to 698.4) | 597.6 (450.5 to 696.6) | 599.9 (454.3 to 694.5) | 603.2 (451.2 to 698.6) | 609.7 (452.4 to 706.3) | 619.5 (455 to 715) | 630.9 (455 to 730.2) | 637.5 (455.8 to 736.1) | 632 (451.1 to 737.2) | 634.1 (449.4 to 733.6) | 646.3 (452.8 to 749.4) | 657.3 (463.2 to 762.1) | 622.4 (442.5 to 718.3) | 547 (393.3 to 648.3) | 496.8 (354.1 to 605) | 472.6 (331.9 to 589.4) | 450 (310.6 to 570.3) | 432.9 (292 to 563) | 416.8 (284.4 to 535.9) | 389 (267.2 to 500.2) |
|  |  | Neurological disorders | 167.6 (90.3 to 216.6) | 159 (86.9 to 205) | 154.9 (87.3 to 199.4) | 150.5 (84.7 to 193.3) | 146.2 (85.8 to 186.3) | 141.8 (82.5 to 177.6) | 137.8 (81.8 to 170.7) | 134.5 (81 to 165.9) | 131.2 (80.2 to 161.1) | 127.9 (79 to 155.2) | 124.8 (78.6 to 150.6) | 121.8 (76.6 to 146.7) | 119.2 (75.8 to 143.1) | 117.1 (74.7 to 141.4) | 117.2 (75.5 to 141) | 116.4 (75.9 to 138.4) | 116 (77.3 to 137.1) | 116.6 (76.8 to 138) | 117.7 (78 to 138.7) | 118.6 (79.6 to 139.6) | 117.8 (79.3 to 137.8) | 117.5 (80.6 to 135.9) | 117.7 (80.8 to 135.6) | 119.9 (81.9 to 136.9) | 112.2 (78.8 to 126.6) | 114.2 (81 to 129.5) | 101.5 (74.6 to 116.2) | 96.8 (71.4 to 112.1) | 91.3 (67 to 107.6) | 86.7 (63.2 to 103.6) | 73.8 (54.9 to 86.2) | 64.4 (48.3 to 75.5) |
|  |  | Nutritional deficiencies | 237.2 (186.9 to 326) | 200.5 (151.6 to 280.3) | 178.6 (139.1 to 250.2) | 161 (125 to 222.1) | 145.3 (115.8 to 194.7) | 130 (103.5 to 166.5) | 113.8 (90.4 to 146.5) | 104.1 (83.3 to 131.8) | 95.2 (77 to 116.9) | 89 (72.9 to 108) | 82.9 (69 to 99.7) | 78.3 (65.2 to 92.8) | 72 (60.4 to 85) | 67.7 (56.2 to 80) | 63.4 (52.5 to 75.6) | 61.2 (51.3 to 72.2) | 59.8 (50 to 70.2) | 60.3 (50.8 to 71.3) | 63.2 (52.8 to 75.5) | 62.8 (53.4 to 74.6) | 61.1 (52 to 71.5) | 57.3 (49.6 to 66.5) | 54.6 (47.2 to 63.1) | 53.1 (46 to 60.6) | 47.7 (41.6 to 54.9) | 38.8 (33.8 to 44.8) | 29.7 (23.9 to 34.8) | 24.3 (18.7 to 29) | 20.7 (15.5 to 25) | 18 (13.2 to 22.1) | 12.1 (9.2 to 14.6) | 8.9 (6.7 to 10.6) |
|  |  | Other infectious diseases | 1168.6 (612.1 to 2286.8) | 923.2 (515 to 1720.5) | 731.3 (441.6 to 1314.6) | 616.5 (388.5 to 1033.5) | 530.4 (362.5 to 816.1) | 470.2 (330.6 to 736.8) | 428.1 (307.9 to 646.5) | 389.5 (284.6 to 581) | 366.6 (269.3 to 535.8) | 335.5 (256.6 to 467.1) | 320.4 (249.8 to 436.1) | 298.3 (232 to 405.4) | 283.9 (223 to 382.5) | 275.3 (220.5 to 361.4) | 264.1 (211.9 to 346.3) | 263.4 (215 to 339.4) | 266.6 (212.8 to 340.3) | 274.9 (220.8 to 359.3) | 283.4 (221.1 to 372) | 291.4 (227.8 to 374.4) | 289.8 (223.6 to 385) | 288 (224.1 to 388.7) | 288.3 (227.8 to 378.4) | 290.3 (226.8 to 388.2) | 256.1 (188.9 to 366) | 223.5 (154.5 to 325.8) | 185.9 (124.6 to 272) | 164.6 (111 to 245.7) | 147 (100 to 216.8) | 131.3 (90 to 201) | 101.1 (67.4 to 179.5) | 79.2 (53.9 to 126) |
|  |  | Other non-communicable diseases | 9068.1 (5115.6 to 11359.7) | 8229.4 (4769.8 to 10312.4) | 7658 (4549.2 to 9595.4) | 7170.6 (4454.4 to 9053.7) | 6669.4 (4205 to 8463.3) | 6158.8 (4003.6 to 7810.6) | 5711.1 (3850.3 to 7163.5) | 5346 (3700.9 to 6664.5) | 5035.2 (3589.1 to 6264.4) | 4775.7 (3530.1 to 5914.9) | 4573.4 (3453.2 to 5610.9) | 4457.6 (3425.3 to 5478.8) | 4392.7 (3489.4 to 5388.1) | 4330.2 (3458.6 to 5288.8) | 4257.3 (3454.7 to 5128.7) | 4174.9 (3455.4 to 4979.2) | 4192.8 (3461.2 to 4982.1) | 4310.5 (3608.5 to 5063.4) | 4503.3 (3761.5 to 5249.1) | 4654.3 (3953.5 to 5409.5) | 4704.7 (4003.8 to 5409.4) | 4685.1 (4015.3 to 5384.7) | 4648.7 (4021.6 to 5318.6) | 4552.3 (4015.5 to 5172.5) | 4267.1 (3817.6 to 4879.5) | 3740.9 (3399.1 to 4270.9) | 2983 (2677.4 to 3445.4) | 2544.8 (2228.8 to 3028.8) | 2215 (1884.1 to 2689) | 1956 (1649.6 to 2402.8) | 1259.9 (1078.5 to 1552.9) | 879.9 (727.9 to 1120.9) |
|  |  | Respiratory infections and tuberculosis | 3077.3 (2534.7 to 4054.1) | 2651.2 (2142.4 to 3499.5) | 2353.6 (1898 to 3103.1) | 2115.7 (1716.2 to 2766.1) | 1914.1 (1550.6 to 2434.3) | 1735.6 (1422.6 to 2178.2) | 1585 (1299.3 to 1947.1) | 1467.1 (1202.7 to 1792.7) | 1363.8 (1128.4 to 1642.9) | 1272.2 (1060 to 1518.5) | 1189.3 (1000.8 to 1414.8) | 1129.8 (954.1 to 1335.3) | 1101.7 (934.4 to 1304.6) | 1086.1 (925.1 to 1281.9) | 1084.2 (925 to 1278.7) | 1078.9 (922.4 to 1263.1) | 1089.7 (930.2 to 1274.2) | 1113.6 (942.8 to 1309.4) | 1141.7 (979.1 to 1343.4) | 1150.9 (1000.6 to 1359.6) | 1129.3 (986.9 to 1326.2) | 1092.9 (961.1 to 1255.6) | 1062.6 (936.3 to 1209) | 1014.2 (899.1 to 1145.7) | 910 (810.9 to 1019.6) | 747.8 (664.2 to 841.7) | 566.6 (475.3 to 648.7) | 467.5 (373.4 to 548.4) | 396.1 (309.2 to 473.2) | 343.8 (264.6 to 417.7) | 460.3 (371.5 to 551.9) | 392.2 (334.9 to 438.7) |
|  |  | Self-harm and interpersonal violence | 628 (559.6 to 681.6) | 578.4 (498.5 to 627.9) | 521.2 (462.1 to 568) | 514.7 (448.2 to 563.3) | 513.9 (456.2 to 559.7) | 482.4 (424.4 to 530.3) | 495.1 (438.4 to 540.1) | 496.9 (435.3 to 541.5) | 508 (448.3 to 555) | 530.9 (468.3 to 574.9) | 527.9 (470.9 to 571.7) | 560.5 (499.9 to 605.9) | 542 (475.1 to 588.6) | 571 (502.7 to 615.3) | 594.3 (528.3 to 638.2) | 568.3 (505.2 to 612.3) | 572.8 (512.8 to 618.5) | 567 (507 to 610.9) | 584.4 (527.3 to 631.6) | 576.6 (523.2 to 623.2) | 544.4 (491.6 to 586.4) | 497.5 (449.8 to 538.3) | 478.1 (435.3 to 514.3) | 477.5 (437.2 to 514) | 439.9 (407.2 to 471.4) | 417.9 (386.7 to 448.4) | 418.1 (388.6 to 446.5) | 393.9 (363.8 to 425.1) | 354.9 (321.8 to 387) | 310.1 (279.9 to 343.5) | 357.2 (328.2 to 389.1) | 327 (293.3 to 359.8) |
|  |  | Skin and subcutaneous diseases | 13.1 (7.7 to 18) | 12.2 (7.4 to 16.3) | 11.7 (7.5 to 15.3) | 11.2 (7.4 to 14.4) | 10.9 (7.4 to 13.8) | 10.3 (7.1 to 13) | 9.8 (6.9 to 12.2) | 9.4 (6.8 to 11.6) | 9.2 (6.7 to 11.3) | 9 (6.9 to 10.9) | 8.7 (6.8 to 10.7) | 8.4 (6.5 to 10.4) | 8.4 (6.5 to 10.3) | 8.4 (6.6 to 10.4) | 8.4 (6.7 to 10.3) | 8.3 (6.6 to 10.3) | 8.4 (6.7 to 10.4) | 8.6 (6.9 to 10.5) | 8.8 (7 to 10.8) | 9 (7.2 to 10.9) | 9 (7.4 to 10.9) | 9 (7.6 to 10.7) | 9.1 (7.7 to 10.6) | 9 (7.8 to 10.4) | 8.6 (7.6 to 9.7) | 8.2 (7.1 to 9.7) | 7.1 (5.9 to 8.8) | 6.5 (5.2 to 8.4) | 5.9 (4.5 to 8) | 5.5 (4.1 to 7.6) | 4.5 (3.5 to 6) | 3.9 (3 to 5.3) |
|  |  | Substance use disorders | 36.8 (28 to 48.1) | 37.3 (27.2 to 47.9) | 38.8 (29.9 to 50.6) | 39.4 (29.6 to 51.1) | 41.1 (31.6 to 52.8) | 43.7 (33.9 to 55.2) | 47 (37.4 to 60.4) | 51 (40.3 to 65.1) | 54.5 (43.6 to 69.3) | 57.6 (46.3 to 73) | 60.4 (48.7 to 75) | 62.8 (50.3 to 77.9) | 64.8 (53.1 to 79.9) | 66.8 (55.5 to 82.2) | 68.3 (56.8 to 83.5) | 69.1 (57.9 to 83.6) | 69.9 (58.8 to 84.6) | 69 (57.8 to 83.6) | 66.9 (56.4 to 79.4) | 62.6 (53.5 to 75.3) | 58.3 (49.5 to 68.7) | 54.6 (46.8 to 63.9) | 51.1 (44.1 to 59.8) | 46.6 (40 to 54.2) | 44.5 (37.5 to 51.9) | 41.7 (35.5 to 48.7) | 40.6 (34.2 to 48.1) | 40.4 (33.7 to 49.3) | 40.2 (32.3 to 49.5) | 40.4 (32.2 to 50.2) | 39 (30.9 to 48.9) | 39.1 (30.2 to 50.7) |
|  |  | Transport injuries | 4901.3 (4274.1 to 5844.9) | 4677.2 (4070.3 to 5497) | 4528.8 (3975.4 to 5266.8) | 4385.2 (3867.5 to 5065.3) | 4263.6 (3762.6 to 4868.2) | 4124.7 (3663.2 to 4706.9) | 4009.9 (3584.1 to 4547) | 3932.9 (3533.5 to 4377.3) | 3852.4 (3500.2 to 4256.2) | 3796.1 (3474.2 to 4162.9) | 3733.9 (3428.8 to 4057) | 3691.9 (3386.2 to 4003.2) | 3677.1 (3404.6 to 3997.2) | 3655.3 (3389.8 to 3966.3) | 3607.2 (3360.1 to 3912.8) | 3497.8 (3254.7 to 3814.8) | 3397.6 (3171.3 to 3690.9) | 3295.3 (3071.7 to 3585.8) | 3209.6 (2997.9 to 3496.3) | 3100.2 (2889 to 3377.7) | 2903.4 (2718.7 to 3166.4) | 2782.3 (2609.1 to 3011.7) | 2698.6 (2529.7 to 2907.4) | 2611.5 (2458.6 to 2797.2) | 2277.8 (2164.9 to 2416.8) | 1841.1 (1722.5 to 1971.3) | 1600.6 (1478.4 to 1716.4) | 1483.7 (1363.3 to 1600.1) | 1369.8 (1242.1 to 1494) | 1284 (1157.7 to 1411.1) | 1221 (1116.1 to 1328.9) | 1135.2 (1019.4 to 1258) |
|  |  | Unintentional injuries | 8816.2 (8094.6 to 9588.5) | 3121.5 (2602.1 to 3637.5) | 2906 (2456.9 to 3388.4) | 2871.2 (2432.5 to 3287.6) | 2672.4 (2238.6 to 3058.6) | 2557.4 (2149.3 to 2938.3) | 2449 (2057.6 to 2817.3) | 2672.8 (2286.1 to 3006.4) | 2318.4 (1957.8 to 2611.6) | 2250.4 (1895.2 to 2531.9) | 2180.2 (1835.6 to 2430.1) | 2193.9 (1863.1 to 2446.7) | 2095 (1767.3 to 2330.6) | 4673.6 (4297 to 5035.7) | 1994.7 (1716.9 to 2213.3) | 1918.2 (1655.4 to 2129.4) | 1776.7 (1536.9 to 1961.6) | 1712 (1489.6 to 1896.3) | 1664.4 (1458.4 to 1838.2) | 1590.4 (1414 to 1758.2) | 1515 (1360.8 to 1679.8) | 1421.8 (1279.3 to 1578.2) | 1412.2 (1279.2 to 1566.7) | 1342.9 (1218.7 to 1488.9) | 1217.4 (1113.3 to 1350.3) | 1051 (967.2 to 1169) | 894.4 (822.4 to 1009.4) | 894.1 (824.4 to 1004.2) | 746.3 (666.8 to 844.4) | 705.9 (628 to 806.1) | 642.8 (580.4 to 735.7) | 559.3 (492.4 to 649.6) |
